# Fluorescent Isostere (*Fluostere*) of the Carboxylate: Design of *h*DHODH Fluorescent Inhibitors as Proof of Concept

**DOI:** 10.1021/acs.jmedchem.5c00348

**Published:** 2025-06-18

**Authors:** Stefano Sainas, Elena Martino, Claudio Garino, Paola Circosta, Anna Luganini, Marta Giorgis, Francesco Bavo, Marco Piccinini, Cristina Ramondetti, Marta Alberti, Riccardo Miggiano, Giorgio Gribaudo, Donatella Boschi, Bente Frølund, Marco Lucio Lolli

**Affiliations:** † 9314 Department of Drug Science and Technology. University of Torino, Via Pietro Giuria 9, Torino 10125, Italy; ‡ Department of Chemistry, University of Torino, Via Pietro Giuria 7, Torino 10125, Italy; § Department of Clinical and Biological Sciences, University of Torino, Regione Gonzole 10, Orbassano, Torino 10043, Italy; ∥ Molecular Biotechnology Center, University of Torino, Via Nizza 52, Torino 10126, Italy; ⊥ Department of Life Sciences and Systems Biology, University of Torino, Via Accademia Albertina 13, Torino 10123, Italy; # Department of Drug Design and Pharmacology, Faculty of Health and Medical Sciences, University of Copenhagen, Universitetsparken 2, Copenhagen DK-2100, Denmark; ∇ Department of Oncology, University of Torino, Via Michelangelo 27/B, Torino 10125, Italy; ○ Department of Pharmaceutical Sciences, 19050 University of Piemonte Orientale, Via G. Bovio 6, Novara 28100, Italy

## Abstract

Fluorescent probes targeting proteins are used to investigate biological processes, requiring strong binding affinity and favorable fluorescence. In this study, we present the first *fluostere* with optimized fluorescence properties. We started exploring the fluorescence of acidic pyrazolo­[1,5-*a*]­pyridin-2-ol, and, by the introduction of EWGs, π-conjugation, incorporation of push–pull systems and rigid structures, we optimized emission profiles and QY, providing a first *Structure–Fluorescence relationship* (SFR) of the system. To provide proof of concept in biological applications, the established SFR was integrated with *h*DHODH*i*, an important oncology target, enabling the SAR designing fluorescent *h*DHODH*i* **11a** and **14**, with **11a** being the most potent IC_50_ = 170 nM. These inhibitors were validated in vitro for their antileukemic and antiviral activity. As they are both environmentally sensitive fluorescent probes that can highlight their binding to the target, their fluorescence was found to colocalize in the mitochondria, where *h*DHODH is located, in cellular experiments.

## Graphical abstract

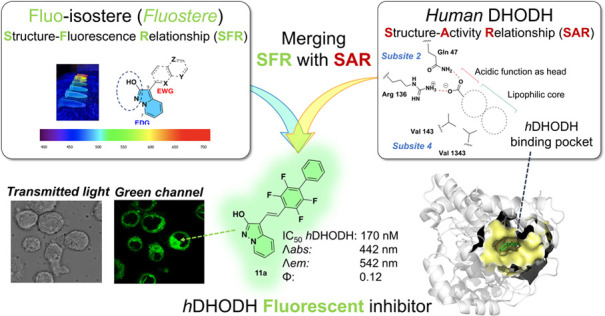

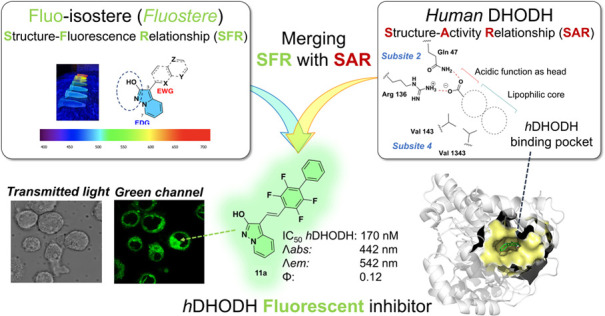

## Introduction

Protein-targeted fluorescent probes have emerged as a promising and fascinating strategy to study and visualize biological processes within living organisms.^1^ Numerous applications are associated with this technique, including studying overexpressed proteins in cancer therapy,^2,3^ investigating the biodistribution of unidentified targets, and early disease evaluation.^4^ The effectiveness of this strategy depends on the development of fluorescent probes endowed with a strong binding affinity to specific targets.^3^ Often fluorescent probes are developed by linking a fluorescent tag to a known bioactive ligand.^5,6^

Recently, merging the pharmacophoric and fluorescent features in a single small molecular structure yielding *bioactive intrinsically fluorescent ligands* (BIFL) has emerged as a new intriguing approach.^7^ However, the retention of the fluorescence profile during the modulation of the structure responsible for target affinity still presents a significant challenge in this approach. Thus, the final compound must have an absorption-emission profile with larger Stokes shift*s* to eliminate spectral overlap while reducing self-quenching and enhancing the imaging signal-to-noise ratio.^4^ High emission quantum yield and physicochemical properties compatible with the biological target are also required. In this regard, Sotelo et al.^7^ recently reported the design of a green-emitting BIFL (compound **55** in ref 7 with strong cannabinoid receptor subtype 2 (CB2R) affinity, high selectivity, and an agonist profile. ^7^ In the compound **55** structure, a fluorescent nitrobenzoxadiazole (NBD) moiety contributes to compound binding by forming π–π interactions with Phe183 and Phe117, introducing favorable pharmacological properties in the CB2R profile.

Harris Friedman introduced the term “bio-isosteres”^8^ in the 1950s to describe a subclass of isosteres that can produce a similar biological effect.^8^ Since then, bioisosteric replacement has been recognized as an effective strategy for increasing potency, enhancing selectivity, modulating physical properties, improving metabolic stability, removing or modifying toxicophores, and obtaining new intellectual property.^9^ In a further development of the BIFL as well as the bioisosteric fields, in this study, we take a ground-breaking step and present a first example of fluorescent isostere of carboxylic acid as a novel principle for designing intrinsically fluorescent ligands. To our knowledge, this is the first case of introducing “fluo-isosteres” (*fluosteres*) as a subclass of isosteres with an optimized fluorescence profile. Among the chemical groups used in bioisosteric replacements for carboxylic function and other acidic moieties are the acidic hydroxyazoles. The heterocyclic nature of these systems, acidic due to lactim-lactam tautomerism, is beneficial as it provides, by strategically altering the ring heteronucleus and substituents, the ability to fine-tune p*K*_a_, lipophilicity-polarity balance and improve affinity to a target protein.^10,11^ Within the hydroxyazole portfolio that we and others have developed for bioisosteric modulations in recent years,^12–21^ the pyrazolo­[1,5*-a*]­pyridin-2-ol (**1**, Figure 1)^22^ is a fluorescent acidic system (p*K*_a_ around 5).^23^ Unfortunately, this system is unsuitable for *in vitro*/*in vivo* applications when unsubstituted, due to its unfavorable excitation/emission wavelengths. In the first part of this study (Figure 1, Steps 1–4), the pyrazolo­[1,5-*a*]­pyridin-2-ol was systematically modulated to identify the structural features responsible for the fluorescent characteristics of the molecule. In other words, initially we focused on constructing a *Structure Fluorescence Relationship* (SFR, Figure 1, Step 5) on the system, looking for improvements in *quantum yield* and emission spectral profile.

**Figure 1:**
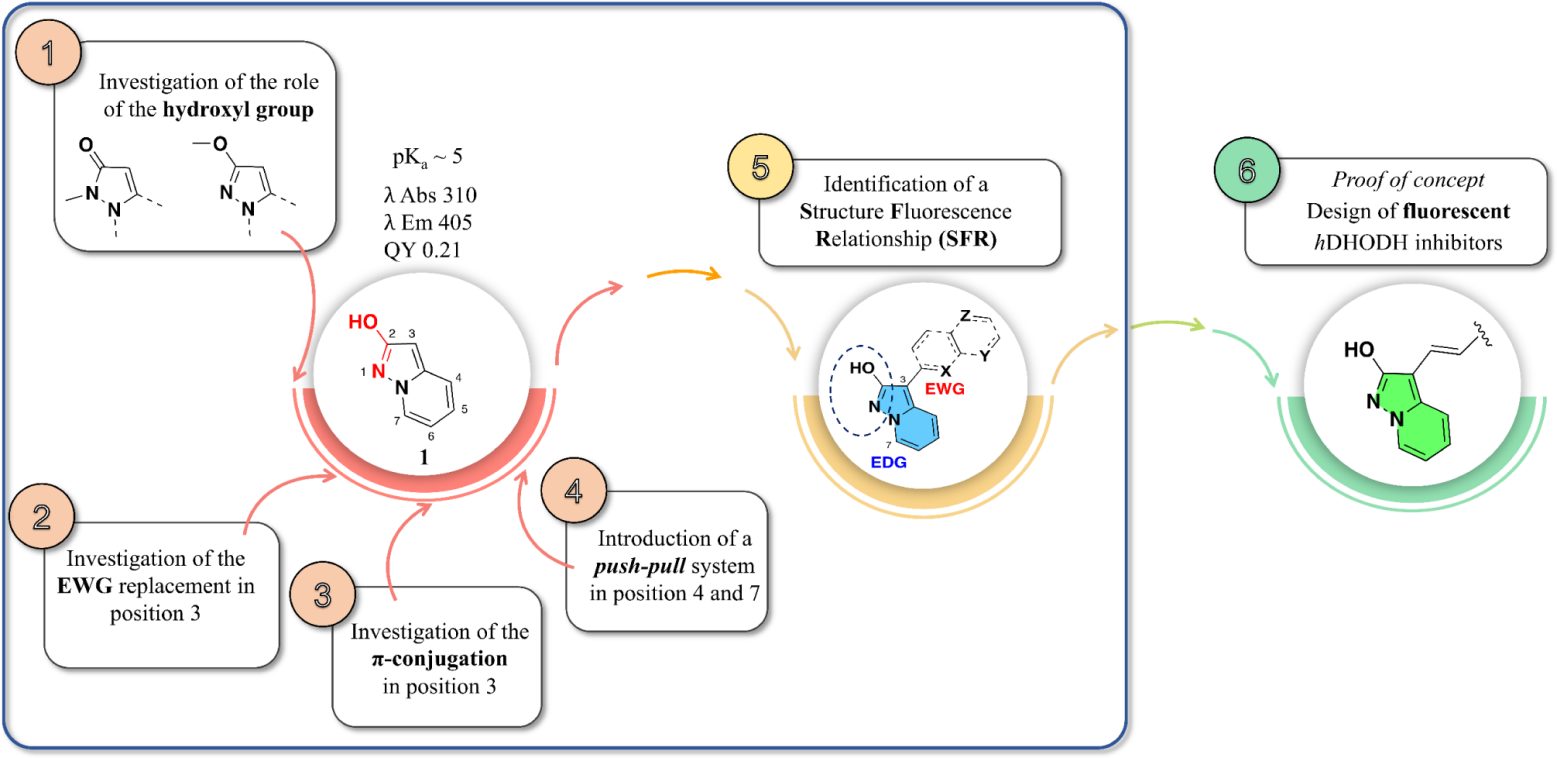
Starting from the structure of pyrazolo­[1,5*-a*]­pyridin-2-ol (**1**), the pathway followed to improve the fluorescence features and the final proof of concept in the design of fluorescent *h*DHODH inhibitors.

In the second part (Figure 1, Step 6), after clearing the preliminary SFR of the developed acid fluosteres, we obtained the first *proof of concept* by applying this new approach to the design of fluorescent bioactive ligands. In this regard, we decided to use our knowledge in the design of *human* dihydroorotate dehydrogenase (*h*DHODH) inhibitors.

The central role of the mitochondrial membrane-bound *h*DHODH in *de novo* pyrimidine biosynthesis has sparked engagement by the academic and pharmaceutical industries communities.^24–33^ *h*DHODH is considered a consolidated target for treating diseases involving cellular proliferation, like autoimmune diseases and cancer (e.g., Acute Myeloid Leukemia (AML)),^34–37^ as well as viral infections.^25,38–44^

Moreover, high levels of *h*DHODH expression in solid and pediatric tumors also indicate the significance of this enzyme as a prognostic marker,^45–47^ highlighting the need for *in vivo* imaging probes to explore inhibitor efficacy further. The design of the first example of *fluorescent h*DHODH inhibitors took advantage of the fact that the structure of potent phase I/II *h*DHODH inhibitors (see example in Figure 2 as *brequinar*,^48^ *vidofludimus*,^49^ *AG-636*,^50^ *ASLAN-003* ^51,52^ and Rhizen RP7214 ^31,32^ often contains a carboxylic group that plays a crucial role in the interaction with Arg136 within the so-called *lipophilic patch* of the enzyme.^49^ After merging the *Structure Fluorescence Relationship* (SFR, Figure 1, Step 5) with the well-known *h*DHODH *Structure Activity Relationship* (SAR), we *fine-tuned* the *fluostere* molecule to engage *h*DHODH Arg136 while retaining optimal optical properties. All the newly developed fluorescent compounds were assayed for *h*DHODH inhibition activity and validated at the cellular level for two main biological effects of *h*DHODH inhibitors: the induction of apoptosis in an AML model and the antiviral effect against coronavirus (*h*CoV-OC43). Then, the most promising fluorescent *h*DHODH inhibitors were further validated through fluorescence microscopy analyses in different types of target cells, which verified their mitochondrial colocalization with the protein target.

**Figure 2:**
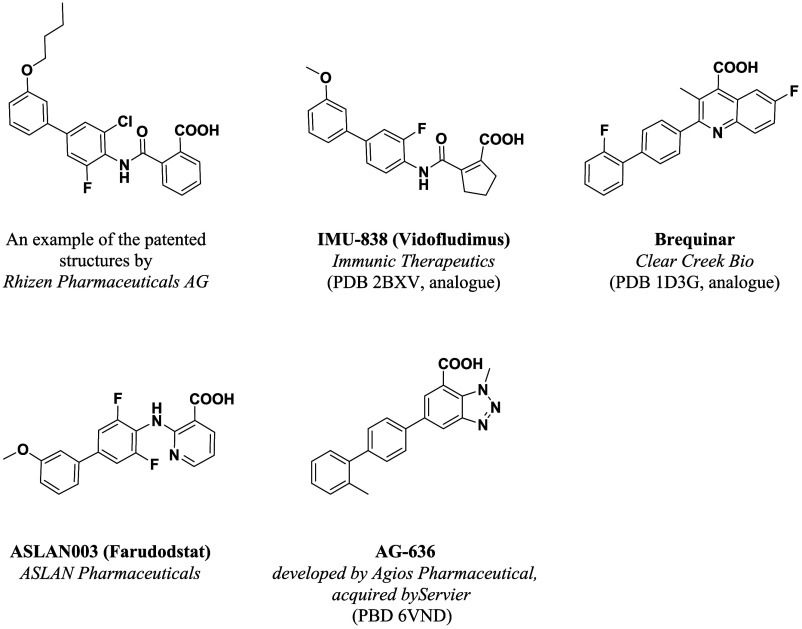
Structure of *h*DHODH inhibitors bearing an acidic moiety. The PDB IDs of the structures of the inhibitors in complex with *h*DHODH are indicated where available. The structure of Rhizen Pharmaceutical AG’s compound RP7214, which is involved in clinical trials, has not yet been disclosed.^31,32,53^ Adapted with permission from reference 33 (Copyright © 2022 by American Chemical Society).

## Results and Discussion

### Design of Acidic Fluorescent Isosteres

Hydroxyazoles are hydroxylated heterocycles characterized by the presence of a hydroxy group in position 2 or 3 with respect to an endocyclic nitrogen atom in a single or fused five-membered heterocycle.^10^

This combination renders these systems slightly acidic, being the negative charge originates from the deprotonation of the hydroxy group delocalized over the oxygen and nitrogen atoms due to lactim-lactam tautomerism. One member of this class, pyrazolo­[1,5-*a*]­pyridin-2-ol (Figure 1, compound **1**), is characterized by a nitrogen-bridged fused 5- and 6-membered ring in which the nitrogen in position 1 exhibits a pyrrolic property. Like the parent compounds indolizine and aza-indolizine, this heterocycle has an aromatic nature with 10 π-electrons. With an acidic p*K*_*a*_ value (the experimental p*K*_*a*_ measured on compound **2** is 5.39, Figure 3),^22^ compound **1** can be considered deprotonated at physiological pH and, thus a carboxylic acid isostere.

**Figure 3:**
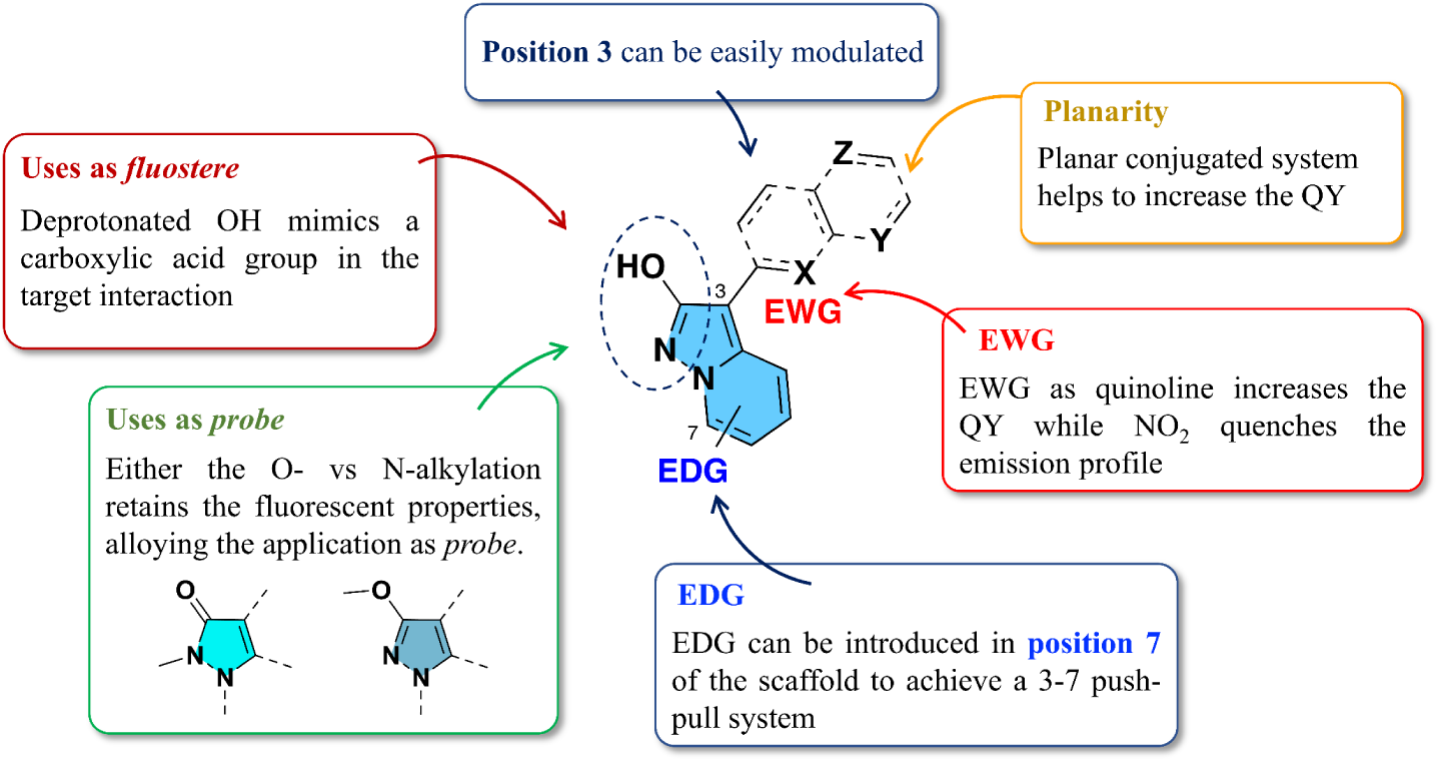
Summary of modifications applied to modulate the emission profile of the pyrazolo­[1,5*-a*]­pyridin-2-ol during the acquisition of the Structure Fluorescence Relationship (SFR).

Since the discovery of its fluorescent nature by Rangnekar et al.,^23^ the chemistry of this system has been poorly investigated. When designing an effective fluorescent probe with this system, it is essential to consider several key features. Specifically, to reduce interference from biological background signals, an optimal fluorescent dye should emit light at wavelengths (λ_em_) longer than 425 nm and exhibit a high Stokes shift. This feature helps prevent the reabsorption of emitted photons, thereby minimizing energy loss and ensuring signal integrity is maintained. Additionally, a high luminescence quantum yield (QY > 0.1), indicating the conversion efficiency of absorbed photons into emitted light, is crucial. We started by analyzing these parameters for **1**, finding a promising starting point with a good QY (0.21) and a high Stokes shift (7567 cm^–1^). However, these features are associated with an excessively short emission wavelength (λ_em_ 405 nm), making it unsuitable as a cell-based probe. In the following chapters, we describe the steps we took to systematically investigate the structure of compound **1** to obtain a fluorescent ligand with optimized properties while enriching the SFR of the pyrazolo­[1,5-*a*]­pyridin-2-ol system.

#### Step 1. Pyrazolo­[1,5*-a*]­pyridine: Investigating the Role of the Hydroxy Group on the Fluorescence Profile

A series of compounds was designed to investigate the role of the acidic hydroxyl group, located in position 2 of the pyrazolo­[1,5*-a*]­pyridine system, on the fluorescence properties.

The acidity introduced by this functional group is crucial for its application as an isosteric replacement of acidic moieties. In azoles, the presence and behavior of lactam-lactim tautomerism strongly depend on the nature of the ring substituents. Besides the fact that spectroscopic techniques suggested that, within lactam-lactim tautomerism, the enol form (OH tautomer) is predominant in solution among the two tautomeric species, alkylation of **1** occurs, although in different ratios, in both the O- and *N*- positions. Starting from **1**, we design and synthesized the nonionizable O-Me (**1a**) and N-Me (pyrazolone, **1b**) analogs. The incorporation of an ester moiety to compound **1** resulted in a slight hypsochromic shift in the violet region (**2**); thus, we considered this replacement in the following modulations, synthesizing also neutral O-alkyl (**2a** and **2c**) and *N*-alkyl (pyrazolone, **2b** and **2d**) analogues (Scheme 1).

**Scheme 1:**
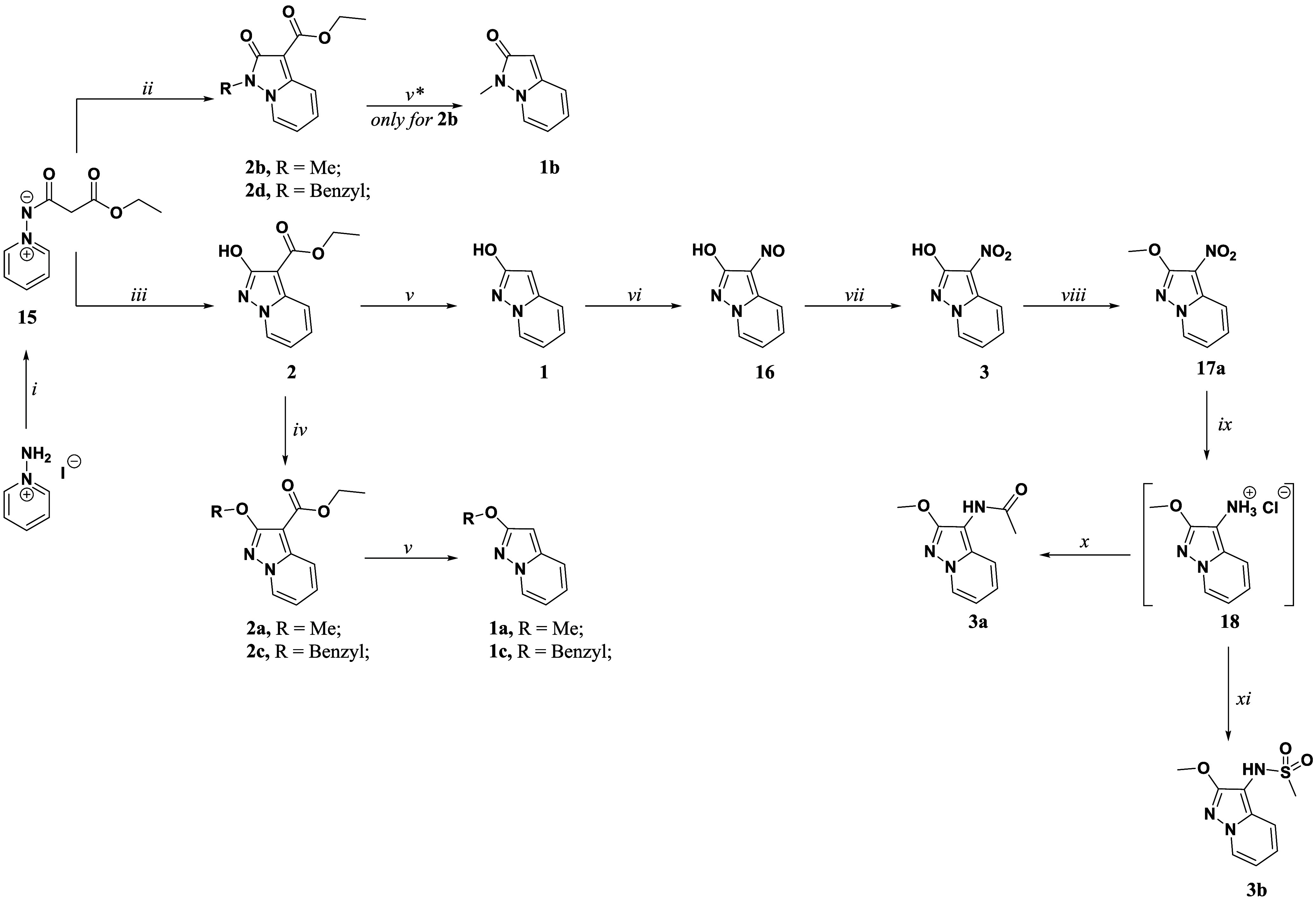
Synthetic Methodologies for the Synthesis of the Targets Compounds **1**, **1ac**, **2**, **2a-d**, **3**, **3ab** ^a^

a (i) K_2_CO_3_, diethylmalonate, EtOH abs, 50 °C; (ii) K_2_CO_3_, MeI or benzyl bromide, dry ACN reflux; (iii) *t*-BuO^–^ K^+^, dry THF; (iv) Cs_2_CO_3_, MeI or benzyl bromide, dry DMF; (v) (a) 5 M NaOH, EtOH, 70 °C, (b) 37% w/w HCl, 70 °C, or * 37% w/w HCl, 0 °C for **1b**. (vi) NaNO_2_, CH_3_COOH; (vii) H_2_O_2_, CH_3_COOH; (viii) Cs_2_CO_3_, MeI, dry DMF; (ix) SnCl_2_, 37% w/w HCl, dioxane; (x) TEA, acyl chloride, dry THF; (xi) TEA, methane sulfonyl chloride, dry THF.

##### Chemistry

For preparing target compounds **1**, **1a**–**b**, **2**, **2a–d**, the synthetic scheme began from a slightly modified procedure adopted in our previous works. In this case, the synthesis started from commercial 1-aminopyridinium iodide (Scheme 1), which was reacted with diethyl malonate in the presence of K_2_CO_3_ and EtOH as a solvent to yield the common intermediate **15**.^54^ Subsequently, intermediate **15** was either treated with *t-*BuO^–^K^+^ as a base in dry THF, to afford compound **2** through cyclization or refluxed with an alkylating agent (benzyl bromide or methyl iodide) and a mild base condition (K_2_CO_3_) in acetonitrile as a solvent, to achieve target compounds **2b** and **2d**. Moving forward in the scheme, compound **2** was protected on the hydroxyl group with a methyl or benzyl group to afford compounds **2a** and **2c,** respectively. Moreover, the ester moiety of compounds **2**, **2a–c** was hydrolyzed in basic conditions to obtain the corresponding acids and immediately decarboxylated in strong acid conditions to afford compounds **1** and **1a–c** in good yields.

##### Fluorescence

The examination of the fluorescence spectra reveals that the pyrazolone analogues (*N*-alkylated; **1b**, **2b**, and **2d**) present the most favorable emission characteristics in the blue section of the spectrum, along with a significant Stokes shift (Table 1). However, they also exhibit lower *quantum yield* (QY) than the parent hydroxylated compounds (**1** and **2**) and the O-alkylated analogues (**1a**, **2a**, and **2c**, see Table **1**). Because the alkylation in both **1** and **2** did not result in major modifications in the luminescence, we focused on assessing for subsequent modifications, when possible, on the alkylated series, as these compounds are easier to handle.

**Table 1:** Spectral Properties of **1**, **2**, **3**, **1a**–**b** and **2a**–**d** in ACN^a^

Compound	λ_abs_ (nm)	λ_em_ (nm)	Stokes shift (cm^–1^)	QY	Lifetime (ns)
**1**	310	405	7567	0.21	12
**1a**	305	400	5942	n.d.	n.d.
**1b**	335	465	6514	n.d.	n.d.
**2**	313	373	5139	0.42	7
**2a**	310	380	5942	0.45	9
**2b**	345	445	6514	0.26	11
**2c**	310	380	5942	0.43	9
**2d**	348	448	6414	0.30	12

a n.d. = Not determined.

#### Step 2. Pyrazolo­[1,5*-a*]­pyridine: Investigating the Role of the EWGs in Position 3 on the Fluorescence Profile

To keep exploring the role of the EWGs in position 3 of pyrazole­[1,5-*a*]­pyridine, other substituents beside the ester were investigated. The Hammett substituent constant (σ_*p*_, Table 2) was used as a parameter for judging the moieties nitro (**3**), acetamido (**3a**) and sulphonamido (**3b**) in terms of electronic effects (Scheme 1).

**Table 2:** Spectral Properties of Compounds **3a**–**b** in ACN^a^

Compound	λ_abs_ (nm)	λ_em_(nm)	Stokes shift (cm^–1^)	QY	Lifetime (ns)	*σ*_*p*_
**3**	361	n.e.^a^	-	-	-	0.78 ^56^
**3a**	312	457	10169	<0.01	-	0.00 ^56^
**3b**	315	468	10379	<0.01	2	0.03 ^56^

a n.e. = No emission observed.

##### Chemistry

The synthesis of compounds **3, 3a** and **3b** is outlined in Scheme 1. The synthesis of compound **3** started from **1**. After failing the direct nitration, although following the condition described in the literature,^55^ compound **1** was first nitrosylated in the 3-position using NaNO_2_ in CH_3_COOH as a solvent. In the following, **16** was oxidized with H_2_O_2_, ending with compound **3** in good yield. The hydroxy function of compound **3** was protected with methyl group using the same procedure outlined in Scheme 1, obtaining both O- (**17a**) or exocyclic nitrogen (**17b** see chemistry characterization) isomers as we earlier described. The nitro group of **17a** was then reduced using SnCl_2_ as a reducing agent in the presence of hydrochloric acid using dioxane as a solvent, giving intermediate **18** in good yield.

Due to the instability of compound **18** to moisture when isolated as a free base, the latter was isolated as hydrochloride salt and immediately used in the following steps. Compounds **3a** and **3b** were achieved by a coupling reaction using acetyl chloride or methane sulfonyl chloride, respectively, in the presence of TEA in dry THF.

##### Fluorescence

Although the nitro group is present in some fluorescent dyes, when used as an EWG on 2-hydroxy-pyrazole­[1,5-*a*]­pyridine it acts as a fluorescence quencher, leading to a complete loss of emission in **3**. On the other hand, a significant bathochromic shift of the emission for compounds **3a** and **3b** compared to compound **2a** is observed, but the QY drops to almost zero.

#### Step# 3. Pyrazolo­[1,5*-a*]­pyridine: Investigating the Role of π-conjugation in Position 3 on the Fluorescence Profile

It is widely recognized that increasing π-conjugation could improve the emission characteristics. To investigate how the increase of the conjugation impacts the fluorescence of the pyrazolo­[1,5*-a*]­pyridin-2-ol, a set of compounds based on the O-methylated scaffold **1a** was then designed (Scheme 2). Compounds **4a** and **4b** were designed to evaluate the impact of a combination of a strong EWG and an increase in the π-conjugation of the system. In contrast, compound **5a** was designed to evaluate only the effect of the π-conjugation on the luminescence. Moreover, starting from the structure of compound **5a**, an *electron-donating group* (EDG) group (−SCH_3_) was introduced (**5b**) to promote electron donation, while three different EWG groups were incorporated to enhance charge delocalization (**5c**–**e**).

**Scheme 2:**
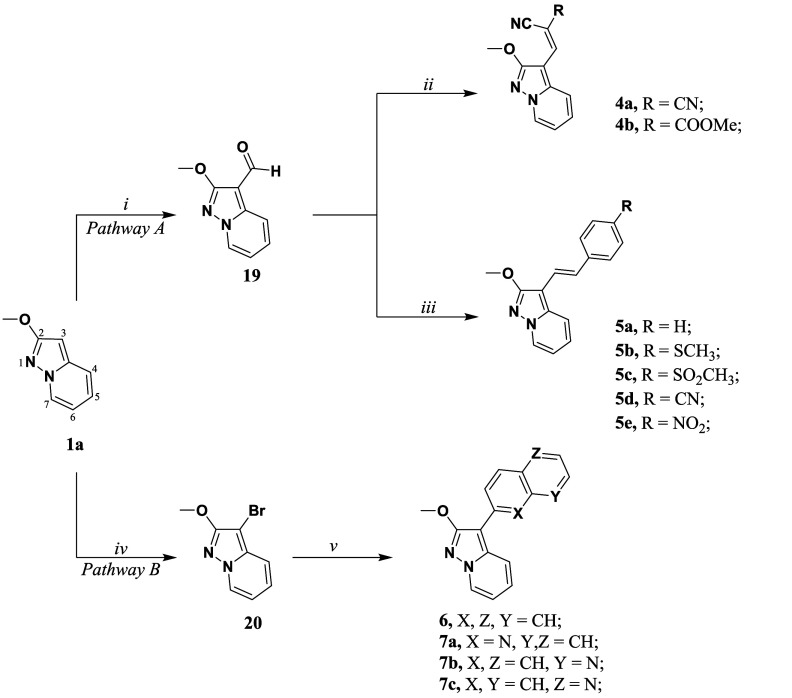
Synthetic Methodologies for the Synthesis of the Targets Compounds **4a–b, 5a–e, 6, 7a–c** ^a^

a (i) POCl_3_, dry DMF, 0 °C; (ii) malononitrile or methyl 2-cyanoacetate, ammonium acetate, CH_3_COOH; (iii) phosphonium salt **35 a–e**, LiHMDS, dry THF, reflux; (iv) NBS, dry CH_2_Cl_2_, −10 °C; (v) [Pd­(PPh_3_)_4_], Cs_2_CO_3_, boronic acid, dioxane/water 8:2 v/v.

##### Chemistry

Starting from compound **1a**, we followed two different synthetic pathways to afford **4a–b, 5a–e, 6, 7a–c** dedicated to modulation of position 3 (Scheme 2). In *pathway A*, **1a** underwent a *Vilsmeier–Haack* reaction at the electron rich position 3 to obtain the corresponding aldehyde **19**. Subsequently, this latter was involved in a *Knoevenagel* reaction to achieve compounds **4a** and **4b**, with a good yield (75–80%). Furthermore, aldehyde **19** was used as the starting material for synthesizing compounds **5a–e** afforded by a *Wittig* reaction. In the synthetic *pathway B,* **1a** was brominated with NBS and PPh_3_ in dry dichloromethane affording **20**. Subsequently, a *Suzuki-Miyaura* cross-coupling was carried out on **20**, which resulted in good yields (70–85%) of the target compounds **6** and **7a–c**.

##### Fluorescence

Compounds **4a** and **4b** were found able to emit yellow light already in the solid state. According to a study conducted by Tigreros et al.^57^ on the closely related pyrazolo­[1,5*-a*]­pyrimidine system, the packing of these heterocycles is predominantly driven by *van der Waals* forces. Additionally, in the absence of bulky substituents in the core structure, they observed the phenomenon known as the *aggregation-induced emission effect* (AIE). It is reasonable to assume that the luminescence of **4a** and **4b** may be attributed to the high level of planarity in the system. Moving to the derivatives **5a–e**, the fluorescence falls in the visible region of the spectra. Thus, compounds **5c** and **5d** show cyan emission, **5a** and **5b** have a bathochromic shift toward green/yellow, and **5e** shows red emission (Table 3). Based on the significant bathochromic emission shift observed in compound **5a** compared to the *lead* compound **1**, we can conclude that the extension of π-conjugation with a single double bond and a phenyl ring could be sufficient to obtain an emission suitable for biological studies. Interestingly, the absorbance shifts significantly when EWGs of different strengths are introduced in the *para* position of the terminal phenyl ring. This suggests that the pyrazolo­[1,5*-a*]­pyridine scaffold would exhibit a more favorable fluorescence profile when acting as a donor group, being conjugated to an EWG.

**Table 3:** Spectral Properties of Compounds **4a–b**, **5a–e** and **6** and **7a–c** in ACN^a^

Compound	λ_abs_ (nm)	λ_em_ (nm)	Stokes shift (cm^–1^)	QY	Lifetime (ns)
**4a**	393	n.e.	-	-	-
**4b**	395	n.e.	-	-	-
**5a**	335	500	9851	<0.01	3.5
**5b**	350	510	8964	<0.01	3.5
**5c**	370	470	5750	<0.01	3.5
**5d**	378	470	5178	<0.01	3.5
**5e**	426	662	8368	<0.01	<0.1
**6**	345	465	7480	0.13	4.3
**7a**	370	422	3330	0.52	2.8
**7b**	356	450	5868	0.42	5.5
**7c**	350	460	6832	0.24	5.3

a n.e. = No emission observed.

Unfortunately, a drastic reduction in quantum yield makes these series unsuitable for biological studies. This decline may be associated with an increase in the internal conversion process, leading to energy dissipation through nonradiative pathways when the molecule returns to the ground state. This behavior may particularly relate to molecular flexibility due to the free rotation around the sigma bonds near the olefinic part.

A common strategy^58^ to enhance the brightness or QY of a fluorescent dye is limiting the rotation by elevating the rotational barrier or increasing rigidification while inhibiting quenching via nonradiative relaxation or intersystem crossing. In this sense, ideally starting with compound **5a**, the substructure was rigidified by substituting the double bond with a naphthalene or quinoline group.^58^ In compound **6**, the alkene substructure of **5a** was rigidified by incorporating the double bond into a naphthalene moiety. Additionally, based on the inherent fluorescent quantum yield of the “*azarene*”” heterocycles, a quinoline ring was used to replace the naphthalene substructure, leading to the design of three compounds: **7a–c**, where the pyrazolo­[1,5-*a*]­pyridine group was introduced at positions 2, 6, and 7 on the quinoline ring (Scheme 3).^59^ The complete rigidification of the scaffold in compound **6** resulted in a modestly diminished blue shift in its emission to a wavelength of 465 nm. Nonetheless, it shows a satisfactory Stokes shift and recovery of the quantum yield. This observation suggests that the reduction in QY in the previous compounds could indeed be due to free rotation around the σ bond. Replacing the naphthalene substructure with a quinoline ring in compounds **7a–c** resulted in emission wavelengths ranging from 422 to 460 nm. Significantly, all compounds had improved QY values, ranging from 0.24 to 0.52 (Table 3).

**Scheme 3:**
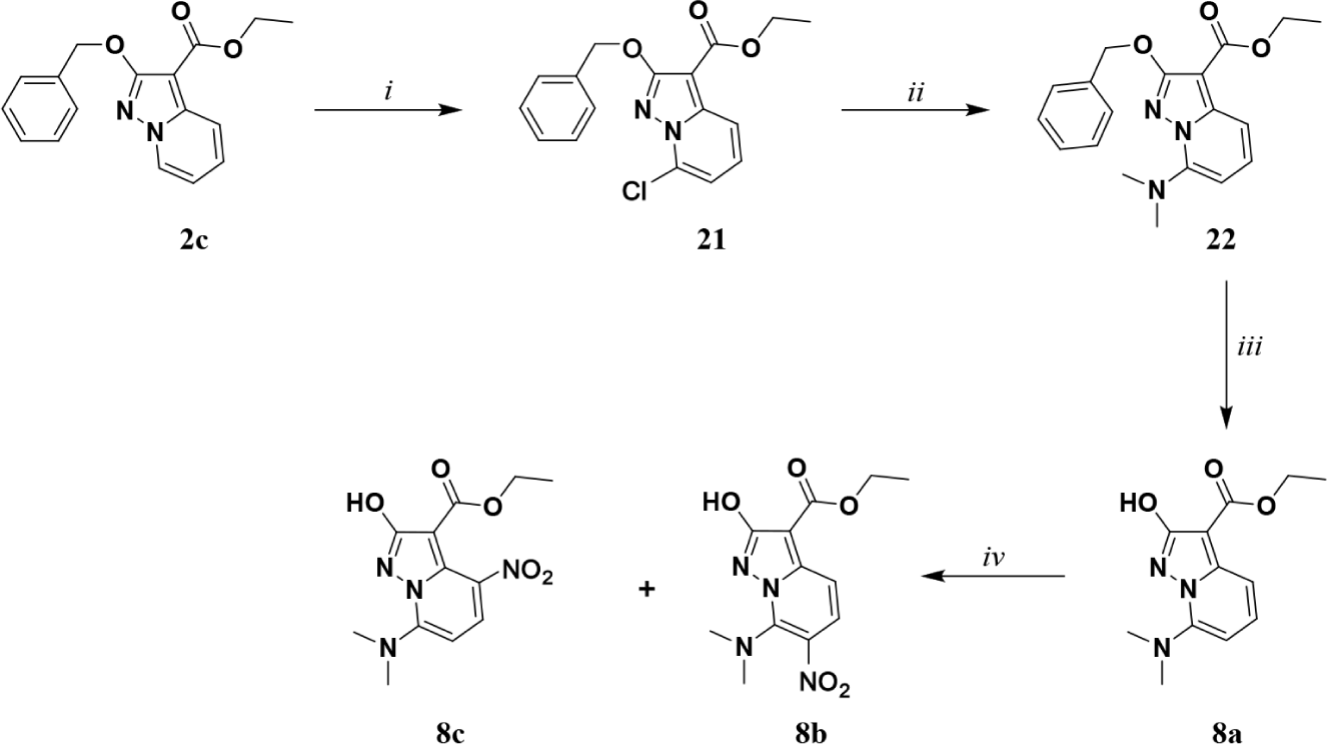
Synthetic Methodologies for the Synthesis of the Targets Compounds **8ac** ^a^

a (i) (a) 1.0 M Lithium Hexamethyldisilazane (LiHMDS), Dry THF, −78 °C, Nitrogen Atmosphere, 1 h, (b) Hexachloroethane, Dry THF, r.t., Nitrogen Atmosphere; (ii) 40% w/w Dimethylamine in MeOH, Dry DMF 80 °C; (iii) H_2_, Pd/C, EtOH; *iv*) NaNO_2_, CH_3_COOH.

#### Step 4. Pyrazolo­[1,5*-a*]­pyridine: Introducing a Push–Pull System

Push–pull fluorophores are recognized as prime candidates for developing effective fluorescence.^60,61^ By functionalizing the core of a molecule with appropriate electron-donating and electron-withdrawing groups, a *push–pull* dye could be created, ideally capable of emitting light at wavelengths longer than 400 nm, which is necessary to prevent overlap with biological autofluorescence. A *push–pull* system based on a pyrazolo­[1,5-*a*]­pyridine core falls under a category of *π-conjugated* molecules featuring an electron donor and an electron acceptor linked by a *π-conjugated* segment. Starting with compound **2**, we examined how the *push–pull* system at positions 7 (acting as the push) and 3 (serving as the pull) influences the fluorescence characteristics of pyrazolo­[1,5-*a*]­pyridine. We initially selected the dimethylamine group as the EDG and identified an ester group as the EWG at position 3 (compound **8a**, Scheme 3). As for compound **2**, we further investigated the role of the nitro group as EWG pull system by inserting this group in positions 6 (**8b**) and 4 (**8c**). Because compound **7c,** characterized by a quinoline scaffold as the EWG, showed optimal λ_em_ and QY, we also designed **9b**, where a dimethylamine group serves as the EDG.

##### Chemistry

The scheme for the synthesis of compounds **8a–c** (Scheme 3), starts from compound **2c** that was converted by lithium hexamethyldisilazane (LiHMDS) into the lithium salt by selectively deprotonating position 7. This latter was then quenched with hexachloroethane, affording compound **21** in good yield. A nucleophilic aromatic substitution reaction, using dimethylamine 40% w/w in MeOH as a nucleophile, was employed to achieve compound **22**. The latter was subsequently hydrogenated to remove the benzyl-protecting group to afford compound **8a**. The application of the nitrosation conditions (NaNO_2_ in acetic acid) to compound **8a**, surprisingly afforded directly the nitro compound as a mixture of **8b** and **8c**, obtained the spontaneous oxidation of the nitrous to the nitro group. The mixture was then well resolved by preparative-HPLC (for structure elucidation, see SI).

Compound **9a** was synthesized from compound **2a** (Scheme 4), which was chlorinated at position 7 according to the procedure described in Scheme 4 affording compound **23**. The ester of compound **23** was then hydrolyzed in basic environments and the resulting acid was decarboxylated under strongly acidic conditions to give **24**. In the following, **24** was iodinated in 3 position using NIS in dry DCM to afford **25**. A *Suzuki-Miyaura* cross-coupling reaction with the quinolin-6-ylboronic acid was applied to **25** affording **9a** in good yield. Finally, **9b** was afforded through a SNAr reaction using dimethylamine (40% w/w) in MeOH.

**Scheme 4:**
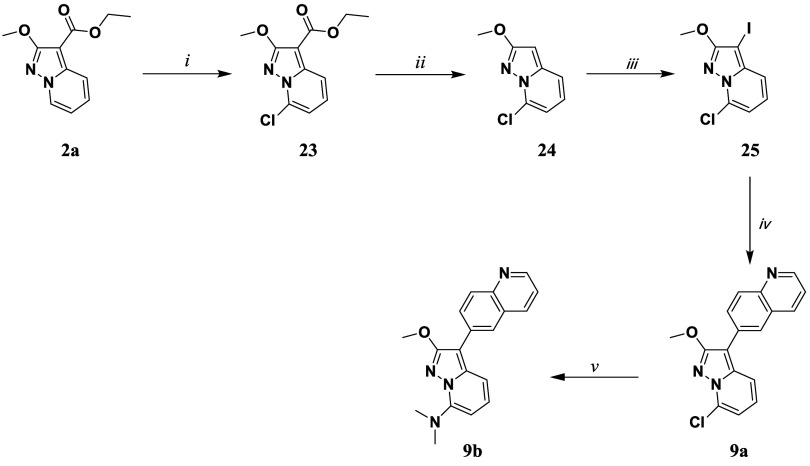
Synthetic Methodologies for the Synthesis of the Targets Compounds **9a** and **9b** ^a^

a (i) (a) Nitrogen Atmosphere, 1.0 M LHMDS, Dry THF, −78 °C, 1 h, (b) Nitrogen Atmosphere, Hexachloroethane r.t.; (ii) (a) 5 M NaOH, EtOH, 70 °C, (b) 37% w/w HCl, 70 °C; (iii) NIS, Dry DCM, −10 °C; (iv) [Pd­(PPh_3_)_4_], Cs_2_CO_3_, Quinolin-6-ylboronic Acid, Dioxane/Water 8/2 v/v; *v*) Dimethylamine 40% in MeOH, Dry DMF 80 °C (Seal Tube).

##### Fluorescence

When comparing **8a** (Table 4) with **2** (Table 1), it is possible to observe a shift to the red region of the spectrum, while an acceptable quantum yield was retained. Moving to **8b** and **8c**, also in this case, the nitro group quenches the fluorescence. A similar beneficial effect due to the presence of a dimethylamino EDG in position 7 could be seen comparing **9a** (Table 4) with **7c** (Table 3) as a shift to the red region is observed, while the QY is acceptable. Compound **9a**, an intermediate for synthesizing **9b**, was also included to investigate the retro-donating effect of the chlorine in position 7. Introducing a chlorine atom in **9a** also shifts the emission into the red region of the spectrum, although at the cost of a significant reduction in QY, which can be understood considering the heavy atom effect.^62^

**Table 4:** Spectral Properties of Compounds **8a–c** and **9a**–**b** in ACN^a^

Compound	λ_abs_ (nm)	λ_em_ (nm)	Stokes shift (cm^–1^)	QY	Lifetime (ns)
**8a**	338	411	5255	0.06	3.7
**8b**	402	n.e.	-	-	-
**8c**	415	n.e.	-	-	-
**9a**	350	460	6832	<0.01	5.1
**9b**	362	552	9508	0.10	2.7

a n.e. = No emission observed.

#### Step 5. Pyrazolo­[1,5*-a*]­pyridine: *Structure Fluorescence Relationship* (SFR)

The data gathered from this investigation were utilized to establish what we refer to as the SFR for the pyrazolo­[1,5-*a*]­pyridine-2-ol core scaffold (Figure 3). While still preliminary and source of continuum expansion, this SFR here proposed could already guide future applications in designing fluorescent ligands.

Because O-/*N*-alkylated or free–OH derivatives show comparable fluorescent properties, optimized pyrazolo­[1,5-*a*]­pyridin-2-ol derivatives can be used either as isosteres of carboxylic acids or as a probe to be attached to an already existing bioactive ligand. In the second application, considering the two isomers obtained after scaffold alkylation, the *N*-alkylated series shows a better emission profile than the O-alkylated. However, both are suitable for biological applications when correctly modulated. While incorporating the -NO_2_ group in different positions of the backbone is not allowed due to fluorescence quenching, a small EWG in position 3 is tolerated but causes the loss of QY. Nevertheless, when strategically employed within a π-extended system, it is able to induce a red shift in the emission. To achieve a red shift while retaining acceptable QY, it is also advantageous to introduce an EWG characterized by a rigid structure, such as naphthalene or quinoline. Other groups, such as pyridine or heterocycles, would also be reasonable to suggest and will be soon investigated. Moreover, to achieve a red shift while retaining the QY, it is beneficial to introduce an EWG characterized by a rigid structure in that position. Lastly, to enhance electron density and thus the effectiveness of the electron-donating strength of pyrazolo­[1,5-*a*]­pyridine, an additional EDG can be introduced at position 7 of the core scaffold as a disubstituted amino group. It must be emphasized that this SFR on the pyrazolo­[1,5-*a*]­pyridin-2-ol system has to be regarded as provisional and will therefore be expanded in the future. However, it contains sufficient evidence to establish *proof of concept* in developing fluorescent ligands as well as all the synthetic strategies helpful for easily modulating the core scaffold during the development.

### Design of Fluorescent *h*DHODH Inhibitors

#### Role and Targeting *h*DHODH

*Human* dihydroorotate dehydrogenase (*h*DHODH) is located in the inner mitochondrial membrane and is involved in *de novo* pyrimidine biosynthesis. This function links *h*DHODH activity to rapid cell growth in autoimmune diseases, cancer, and viral infections, where the need for pyrimidines cannot be met by the salvage pathway alone. *h*DHODH consists of two domains: the C-terminal catalytic domain (Met78-Arg396), which contains the active site where dihydroorotate (DHO) is oxidized into orotate, and an N-terminal domain (Met30-Leu68), which anchors the enzyme to the inner mitochondrial membrane. The N-terminal domain forms a hydrophobic tunnel (termed a *lipophilic patch* by Baumgartner et al.^49^ within the membrane and harbors the FMN binding site, where FMN is reduced to FMNH_2_ simultaneously with the oxidation of DHO. This tunnel provides access to the second cofactor, ubiquinone (CoQ), to reach FMN and triggers the second step of the catalytic reaction.

Due to the peculiar *ping pong mechanism* that characterizes *h*DHODH, the *h*DHODH inhibitors described in the literature are designed to bind to the ubiquinone binding site, the cofactor that connects *h*DHODH to the mitochondrial respiratory chain. The effect of blocking the access of CoQ to FMN, disrupt the ping-pong enzyme mechanism and by reflex block the enzyme activity. Baumgartner et al.^49^ first described the topography of the lipophilic patch, subdividing it into five subsites, as shown in Figure 4. Subsite 1 is highly lipophilic and represents the entrance of the lipophilic patch (Met43, Leu42, Leu46, Ala59, Phe62, Phe98, Leu68, Leu359, and Pro364), which harbors Subsite 2, characterized by two polar amino acids, Arg136 and Gln47. The common structural features of *h*DHODH inhibitors listed in Figure 2, the best known of which is brequinar, include a bulky lipophilic tail required for interaction with Subsite 1 and an acidic polar head interacting with Arg136 in polar Subsite 2, the latter being considered a key interaction.

**Figure 4:**
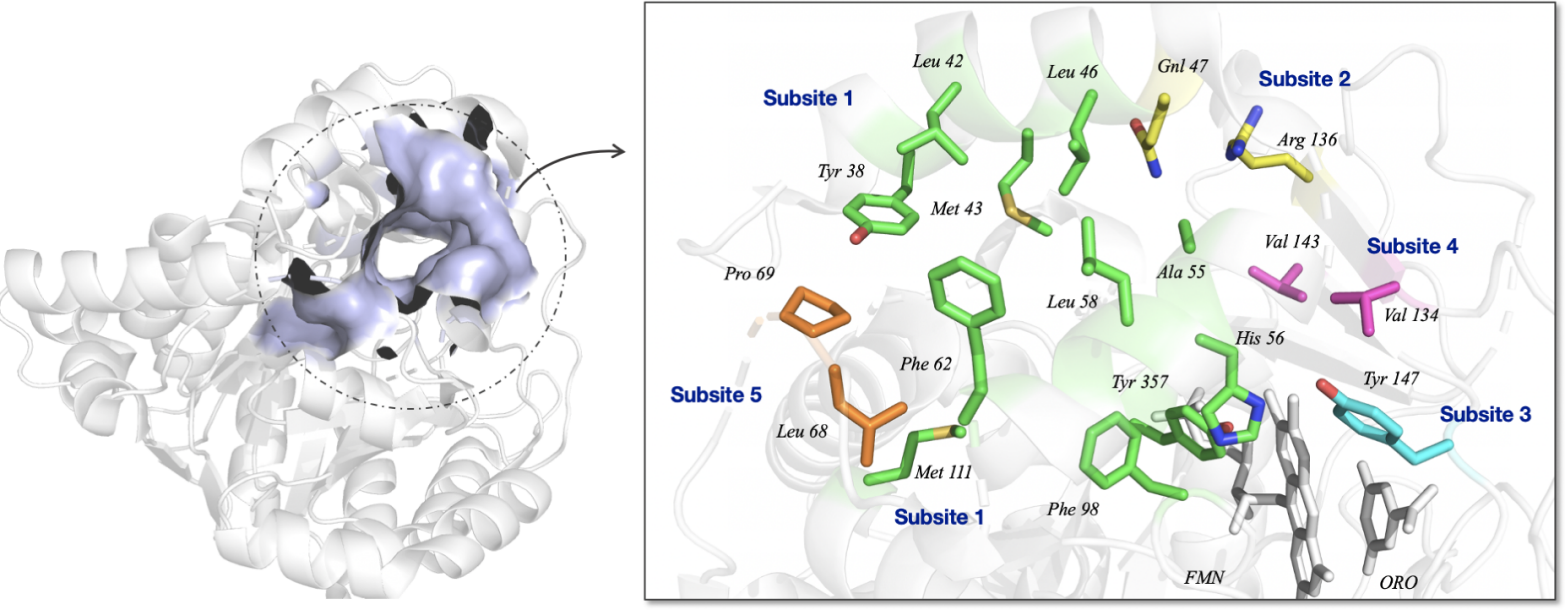
Representation of *h*DHODH CoQ binding site and subsites classification. On the left image is the representation of the pocket surface in which the *h*DHODH inhibitors are bound. Zoom-in on right shows the amino acids forming *Subsite* 1 (green), *Subsite 2* (yellow), *Subsite* 3 (cyan), *Subsite* 4 (pink), *Subsite* 5 (orange). ORO and FMN are gray. The images were created using PyMOL starting from PDB ID: 6FMD.^63^

Since 2012, our group at the University of Turin (IT) introduced a new generation of *h*DHODH inhibitors^64,65^ designed using scaffold-hopping replacement of brequinar’s acidic moiety with various acidic hydroxylated azoles.^22,27,33,64,65^ While different azoles successfully play this role (1,2,5-oxadiazole, thiadiazole, triazole, and pyrazole, Figure 5), giving inhibitors with IC_50_ in the nM range,^64^ the pyrazolo­[1,5-*a*]­pyridine is the most effective being also able to engage contacts with Val134/Val143 (Subsite 4) through its pyridine submoiety. MEDS433 is an orally active best-in-class *h*DHODH inhibitor (IC_50_ *h*DHODH 1.2 nM) currently in advanced preclinical studies (intellectual property owned by the UniTo spinoff Drug Discovery and Clinic s.r.l.).^66^ Unfortunately, the pyrazolo­[1,5-*a*]­pyridin-2-ol moiety in MEDS433 has a poor fluorescence, falling outside the above-described SFR requirements.

**Figure 5:**
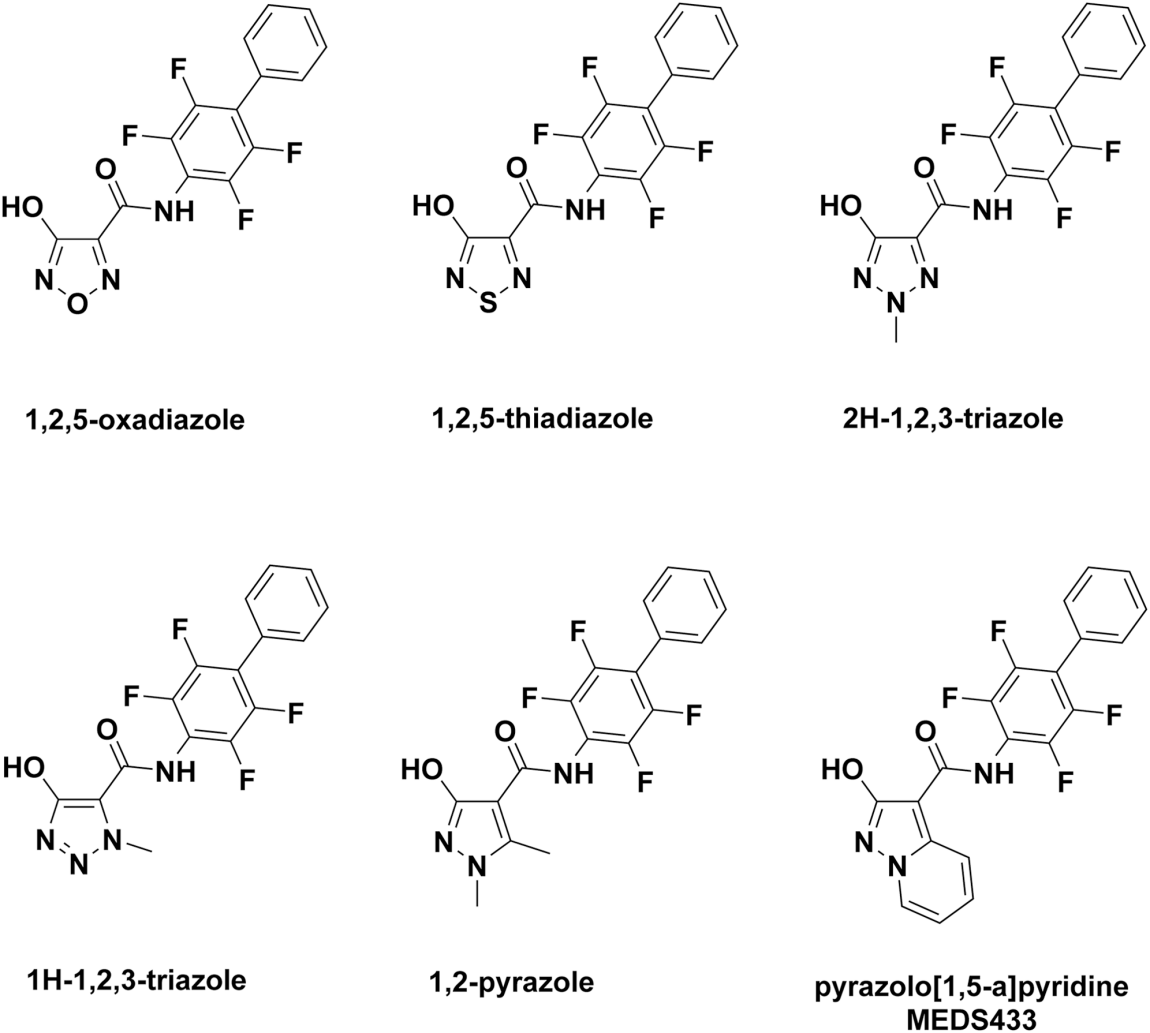
Structures of *h*DHODH inhibitors based on hydroxyazole.

#### Design of Fluorescent *h*DHODH Inhibitors: Merging SFR and SAR

With *h*DHODH inhibitor SAR principles in hand, the next step was to apply them at the initial compound optimization steps. From the SFR/SAR comparison shown in Figure 6, the first two design steps are pretty obvious: 1) the azole OH group must be maintained because it is required in the interactions with Arg136 and Gln47 inside the *Subsite 2*, and 2) no suitable EDG substituents, although beneficial for SFR, can be placed in position 7 of pyrazole­[1,5*-a*]­pyridine because it is known from the SAR that this position does not tolerate substitutions.^22,27,33,64^

**Figure 6:**
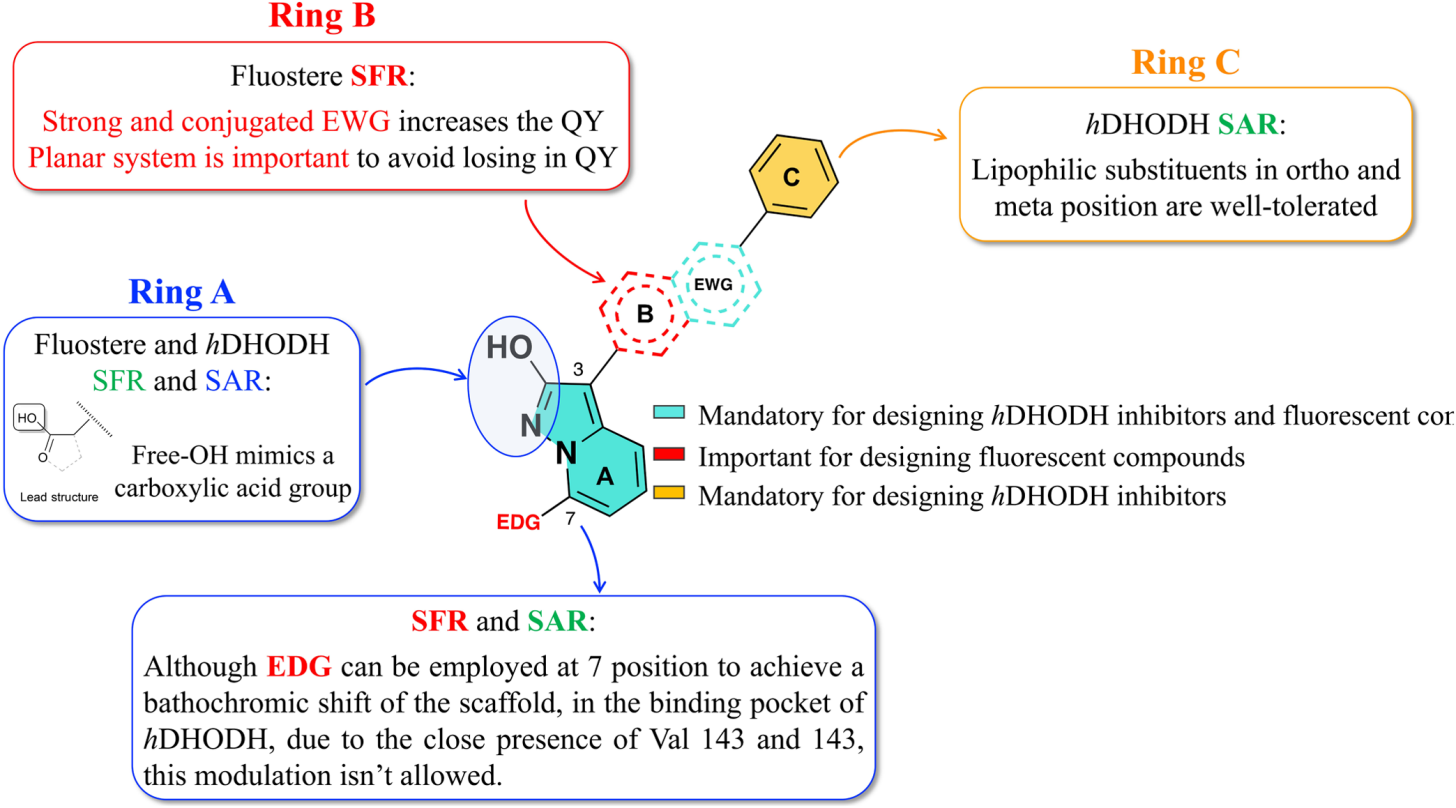
Merging between *h*DHODH SAR and pyrazolo­[1,5*-a*]­pyridin-2-ol core SFR for the design of fluorescent *h*DHODH inhibitors.

Since MEDS433 has a central amide that does not appear to be crucial in the interactions within the binding pocket, the design was focused on its replacement with a substructure able to enhance the conjugation of the system toward a biphenylic scaffold tail while retaining the correct group orientation. The olefinic substructure of compound **11a–b**, which could be seen as a close relative of **5a**, was first employed to mimic the trans conformation of the amide moiety. The biphenyl was perfluorinated in the internal ring to optimize the dihedral angle as well as introducing a push–pull system with the pyrazolo­[1,5*-a*]­pyridine acting as an EDG and the tetrafluorobiphenylic system acting as a strong EWG group. As shown by docking experiments, compound **11a** well superimposes on the crystallographic pose of MEDS433 (Figure 8A), retaining the key binding interactions with the pocket. In addition, a good overlap between the biphenyl structure can be observed.

Furthermore, although H and F can be considered as isosteres,^67–69^ the difference in *van der Waals* radius and, consequently, in the volume they occupy could be beneficial for reducing the free rotation of biphenylic substructure, and potentially restore the QY value, totally lost in the compound **5a**–**e** series. The following design was focused on introducing planarity and reducing free rotation, according to what was learned from SFR. Compound **12** was designed based on compound **7a** due to the high QY and good overlap with MEDS433 observed in docking analysis (Figure 8B). In detail, the position of the nitrogen atom of the quinoline ring of compound **12** could mimic the nitrogen atom of the amide moiety. In addition, the 6-phenyl quinoline substructure fits well into the hydrophobic *Subsite 1* pocket, substantially overlapping with the biphenyl moiety (Figure 8B). Considering the favorable emission and QY of compound **7b**, this latter was employed as a basis for developing compounds **13** and **14** (Figures 7 and 8C–D). In molecules **12** and **13**, we investigated how different positions of the quinoline nitrogen can influence the overall *h*DHODH inhibitory activity.

**Figure 7:**
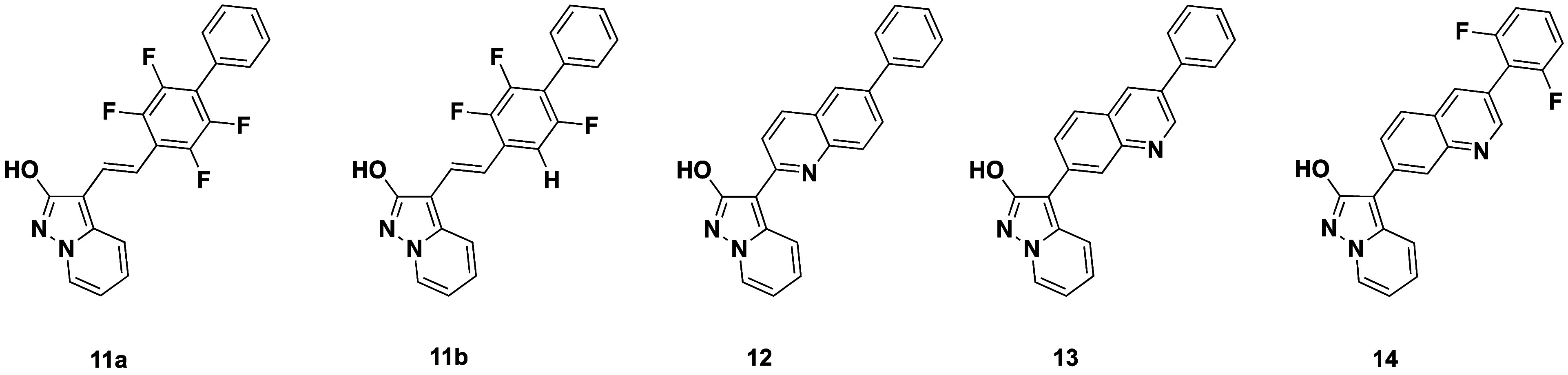
Structures of the designed fluorescent *h*DHODH inhibitors.

**Figure 8:**
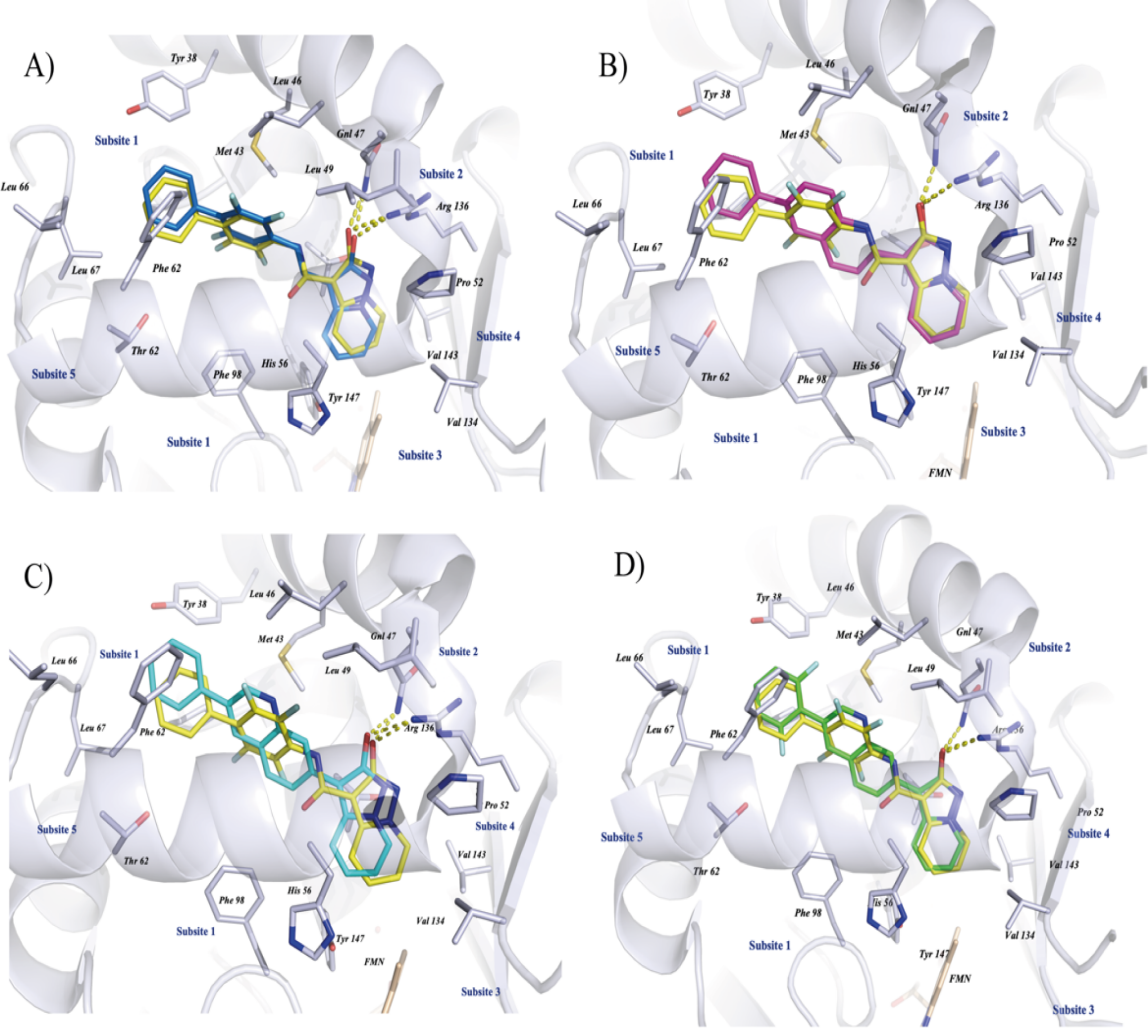
Ubiquinone binding site of *h*DHODH (PDB ID code: 6FMD). Docking poses of target compounds overlapped with the crystallographic pose of MEDS433. The main amino acidic residues contributing to the binding are shown in the stick representation (carbon backbone is in light gray, nitrogen atoms in blue, oxygen in red, and sulfur in yellow). A yellow dish represents polar interactions. In panel A, the docking pose for compound **11a** is blue. Panel B shows the docking pose for compound **12** in pink. Panel C shows the docking pose for compound **13** in cyan. Panel D shows the docking pose for compound **14** in green. The image was created using PyMOL.^63^

Moreover, by analyzing the crystallographic pose of MEDS433, it is possible to observe that the presence of two fluorine atoms in the *ortho* position imposes a rotation of approximately 50° in the terminal ring configuration. Compound **14** was, therefore, designed to mimic the molecular geometry observed in the biphenyl ring of MEDS433.

##### Chemistry

The synthesis of the target compounds as the first example of fluorescent *h*DHODH inhibitors is described in Schemes 5 and 6. To synthesize the final compounds **11a** and **11b**, the procedure starts from the intermediate **1.** After the protection of the hydroxy group with *p*-methoxybenzyl bromide to afford **26**, the latter was converted into the corresponding aldehyde **27** via *Vilsmeier–Haack formylation*. This latter was involved in a *Wittig reaction* with the corresponding phosphonium salts (for the synthetic procedure, see SI). In both cases, a mixture of Z and E isomers was isolated. The E isomer **28a**, identified by J coupling constant (J ≥ 16 Hz), was isolated together with the defluorinated analogue **28b**. Both compounds were deprotected using TFA and thioanisole to afford the desired target compounds **11a** and **11b** in quantitative yield.

**Scheme 5:**
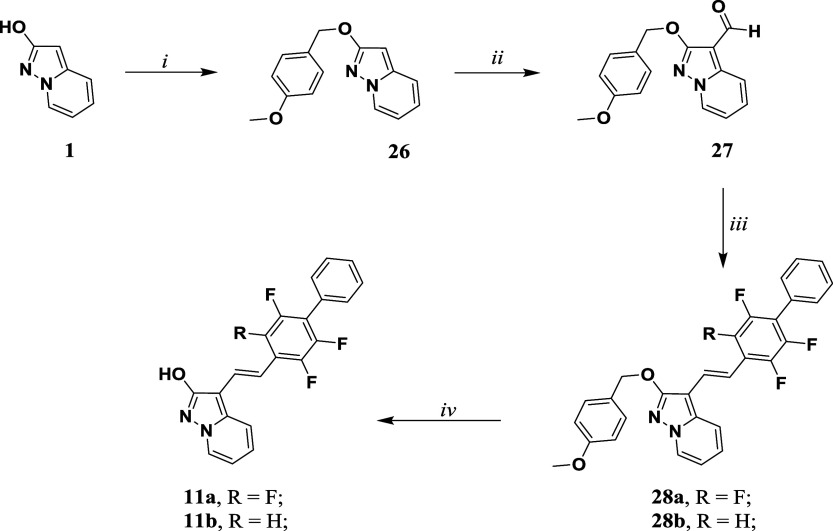
Synthetic Methodologies for the Synthesis of the target **11a, b** [Fn sch5-fn2]

a (i) Cs_2_CO_3_, 4-methoxybenzyl chloride, dry DMF; (ii) POCl_3_, dry DMF; (iii) LiHMDS, dry THF, **41a** and **b** reflux; (iv) TFA, thioanisole, 0 °C.

**Scheme 6:**
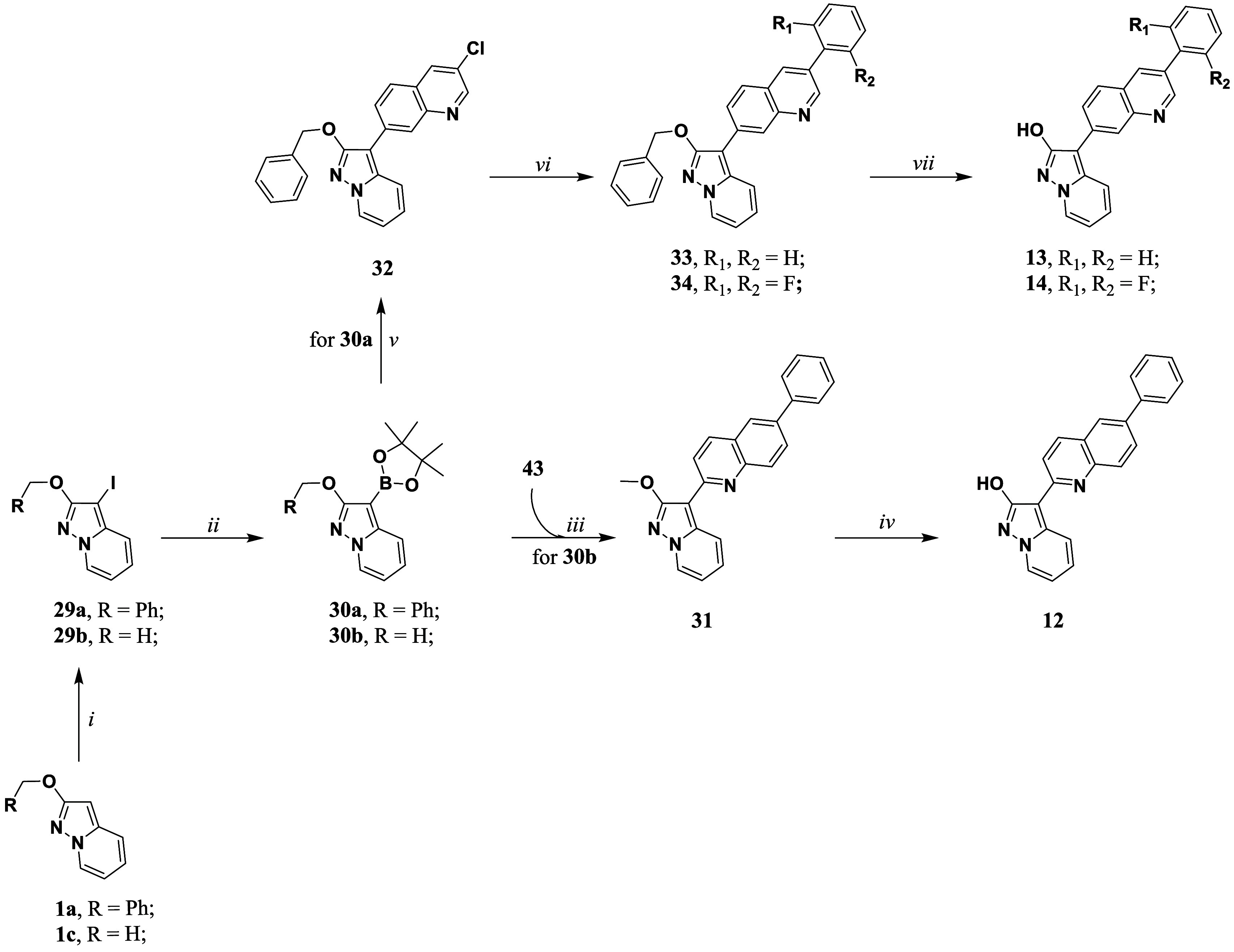
Synthetic Methodologies for the Synthesis of the Target Compounds **12–14** [Fn sch6-fn3]

a (i) NIS, PPh_3_, dry DCM; (ii) *Turbo Grignard*, 2-isopropoxy-4,4,5,5-tetramethyl-1,3,2-dioxaborolane, dry THF, 0 °C; (iii) and (v) [Pd­(PPh_3_)_4_], **30b** (for iii) or **30a** (for v), Cs_2_CO_3_, dioxane/water, 8:2 v/v; (iv) 48% w/w HBr, reflux; (vi) [Pd­(OAc)_2_], K_3_PO_4_, boronic acid, X-PHOS, dioxane/water 8:2 v/v; (vii) TFA, thioanisole, 0 °C.

To achieve target compounds **12–14**, compounds **1a** and **1c** were iodinated using NIS and PPh_3_ in dry dichloromethane, to achieve intermediates **29a–b** in a good yield. In the following, compounds **29a–b** were allowed to react with 2-isopropoxy-4,4,5,5-tetramethyl-1,3,2-dioxaborolane and *turbo Grignard* reagent in dry THF, leading to the formation of intermediates pinacolic boronic ester **30a–b** in good yield. Due to high reactivity, these intermediates tend to undergo deborylation over time. So, they were immediately coupled with compound **43** (see Supporting Information) *via Suzuki-Miyaura* coupling to afford compound **31**. Compounds **33** and **34** were obtained by two consecutive *Suzuki cross-coupling* reactions starting from intermediate **30a**. Subsequently, the final compounds **12–14** were obtained by deprotection of the hydroxy group by heating under reflux in 48% w/w aqueous HBr solution for compound **12** and using the TFA/thioanisole system for compounds **13** and **14**.

#### *h*DHODH Enzyme Activity Inhibition

The features of the *h*DHODH inhibitors just described prompted the selection of compounds **11a** and **12–14** for the evaluation of their inhibitory activity. These compounds were compared with **MEDS433** and Teriflunomide the only FDA/EMA-approved *h*DHODH inhibitor (Table 5).

**Table 5:** Enzyme Inhibition by MEDS433,^22^ Teriflunomide, and **11a** and **12–14**, with Relative IC_50_ Values Shown

Compound	*h*DHODH^*a*^ IC_50_ (C.L. 95%) μM
Teriflunomide	0.39 (0.25–0.52)
MEDS433	0.0012 (0.0010–0.0021)
**11a**	0.17 (0.14–0.21)
**12**	16 (7–25)
**13**	33 (23–42)
**14**	22 (10–53)

As seen in Table 5, all the designed compounds act as *h*DHODH inhibitors presenting IC_50_ values in the low μM range. The most interesting compound in the series is **11a**, which reaches the nM range with an activity 2-fold superior to *Teriflunomide*.

#### *h*DHODH Related AML Cellular Activities

In 2016, a breakthrough discovery showed the association of *h*DHODH activity with myeloid differentiation in acute myeloid leukemia (AML)^35,36^ cells, opening new possibilities for addressing a severe pathology with a poor prognosis. In AML, *h*DHODH is required to maintain the undifferentiated state of leukemia blasts, which are immature cells that cannot mature into adult white blood cells. These blasts proliferate in the blood and target organs, while their accumulation in the bone marrow disrupts normal blood cell production, causing the disease. By inhibiting *h*DHODH and blocking *de novo* pyrimidine biosynthesis, AML cells are forced into a state known as “*pyrimidine starvation,*” forcing them to differentiate and then go to apoptosis. We initially investigated the proapoptotic activity of our compounds in THP1 AML cell lines, a system we frequently explored during the MEDS433 optimization. Table 6 shows the biological activity of compounds **11a** and **14** compared to the control MEDS433.

**Table 6:** Analysis of the Biological Activity (Inhibitory Activity on the Enzyme, ,Apoptosisand Viability) of Compounds **11a** and **14**, Compared to MEDS433^a^

Compound	*h*DHODH^b^ IC_50_ μM (C.L. 95%)	Apoptosis EC_50_ THP1 (nM) (C.L. 95 %)	Viability EC_50_ K562 (nM) (C.L. 95 %)	Viability EC_50_ A549 (nM) (C.L. 95 %)	MRC-5 CC_50_ (μM) (C.L. 95 %)^b^
**MEDS433**	0.0012 (0.001 – 0.0021)	72 (42 – 124)	32 ^70^	n.d.	104800 ^41^
**11a**	0.17 (0.14–0.21)	693 (567–873)	114 (57 – 186)	423 (375 - −476)	7000 (3000 – 10000)
**14**	22 (10–53)	n.d.	n.d.	n.d.	n.d.

a The apoptotic and viability data are expressed as EC_50_ on indicated cell lines. “n.d.” Indicates that the compound was not tested in that specific assay.

b CC_50_, compound concentration producing 50% cytotoxicity, as determined by cell viability assays performed in MRC-5 cells. Reported values are the means (C.L. 95%) of each group.

The antileukemic pro-apoptotic activity requires inhibitors with IC_50_ in the one-digit nM range for the isolated enzyme and for this reason, the relatively low inhibitory activity on recombinant *h*DHODH *in vitro* could explain the absence of pro-apoptosis efficacy of compound **14**. Although **11a** is not as potent as MEDS433 on isolated enzymes (0.17 μM and 0.0012 μM, respectively), it still shows significant pro-apoptotic activity on the AML cell line, falling within the nM range. As shown in Table 6, the antileukemic activity of **11a** is nearly 10-fold lower than that of our designed best-in-class MEDS433, despite the latter being 140 times more potent on the isolated enzyme. The pro-apoptotic activity of **11a** was completely reversed by adding exogenous uridine^27^ (100 μM), suggesting that the induction of apoptosis is mainly due to *h*DHODH inhibition (see Figure S4). To gain a deeper insight into the antitumor potential of the most promising compound **11a**, we extended our viability assays to another leukemic cell line and a solid tumor cell line. Compound **11a** demonstrated potent antiproliferative activity against K562 chronic myeloid leukemia cells, with an EC_50_ value of 114 nM, superior to that observed in THP-1 cells. Moreover, it showed activity against the A549 lung adenocarcinoma cell line, albeit with a slightly higher EC_50_ of 423 nM.

### Antiviral Activity of Target Compounds

Efficient virus replication depends on the availability of pyrimidine nucleotides in infected cells. Therefore, compounds targeting the cellular pathways responsible for providing an appropriate supply of pyrimidines, such as the *de novo* biosynthetic pathway, have the potential to be used as effective host-acting antiviral (HTA) agents.^71^ Moreover, inhibitors of enzymes of the pyrimidine biosynthesis pathway, including *h*DHODH, can overcome the emergence of viral drug resistance. In addition, being independent of virus-specific replication strategies, they may also be effective against various viruses from different families, acting as broad-spectrum antivirals (BSAs).^71^ The emergence of new respiratory virus infections in humans with epidemic or pandemic potential in the last two decades has highlighted the urgent need for effective BSAs to be deployed against future respiratory tract virus infections, with novel coronaviruses and influenza viruses being the most likely to have pandemic potential.^72–74^ To contribute to this antiviral field, in the past few years, we have characterized the potent *h*DHODH inhibitor MEDS433 as a BSA candidate effective against several human respiratory viruses, such as coronaviruses, including SARS-CoV-2, as well as influenza A and B viruses, and the respiratory syncytial virus.^40,41,44,75^ On the other hand, these findings further validate the antiviral activity of *h*DHODH inhibitors as an inherent biological property of such molecules. Therefore, it is interesting to investigate the antiviral activity of compounds **11a** and **14** compared to MEDS433 against the representative human endemic beta-coronavirus *h*CoV-OC43. To this end, FFRAs were performed in HCT-8 cells exposed to target compounds before, during, and after infection with *h*CoV-OC43 (full treatment).

As shown in Figure 9, all the target compounds exert a concentration-dependent inhibitory effect on coronavirus replication.

**Figure 9:**
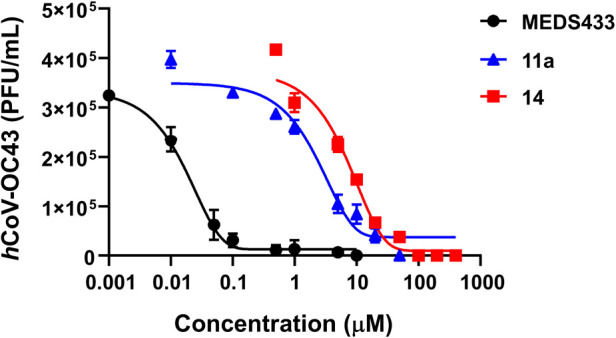
Compounds **11a** and **14** inhibit the replication of *h*CoV-OC43. HCT-8 cell monolayers were infected with the *h*CoV-OC43 (50 PFU/well), and, where indicated, the cells were treated with increasing concentrations of compounds 1 h before, during virus adsorption, and postinfection. Compounds remained in the culture medium throughout the experiment. *h*CoV-OC43 replication was quantified at 72 h p.i. by FFRA, and numbers of viral foci microscopically counted were converted into viral titers (PFU/mL). The compounds concentrations producing 50% and 90% reductions of viral replication (EC_50_ and EC_90_, respectively) were determined by GraphPad Prism. The data are the means ± SDs (error bars) of *n* = 5 independent experiments performed in triplicate.

As expected, the calculated EC_50_ value for MEDS433 is in the low-nanomolar range,^44^ while that of **11a** is in the low-micromolar range (see Table 7). Compound **14** showed a higher EC_50_ value, albeit in the one-digit micromolar range. Notably, the low 50% cytotoxic concentration (CC_50_) of **11a** and **14,** as determined in uninfected HCT-8 cells, confirms that their antiviral activity did not stem from a nonspecific cytotoxicity. Indeed, the Selectivity Index (SI) is about 50 for **14** and 150 for **11a**, respectively (Table 7). Together, these results confirm the retention of antiviral activity in the low-micromolar range for both the fluorescent *h*DHODH inhibitors **11a** and **14**, even though compound **11a** showed a greater anticoronavirus potency than **14**, consistent with its lower IC_50_ value in the inhibition of *h*DHODH activity (Table 5). While **11a** is not toxic to uninfected HCT-8 cells (301 ± 9 μM), it shows low μM toxicity on the MRC-5 cell line (7 μM (3 – 10, C.L. 95%, Table 6)).

**Table 7:** Antiviral Activity of Compounds **11a** and **14** Against *h*CoV-OC43 Replication

Compound	EC_50_ (μM)^a^	EC_90_ (μM)^b^	HCT-8 CC_50_ (μM)^c^	SI^d^
**MEDS433**	0.0142 ± 0.0004	0.08 ± 0.01	84 ± 5	5915
**11a**	2.3 ± 0.5	23.3 ± 0.4	301 ± 9	131
**14**	8.4 ± 0.8	48 ± 4	360 ± 7	43

a EC_50_, the Compound Concentration Inhibiting 50% of Virus replication, as Determined Against *h*CoV-OC43 by FFRAs in HCT-8 Cells. Reported Values are the Means ± SD of Data Derived from Five Experiments Performed in Triplicate.

b EC_90_, the Compound Concentration Inhibiting 90% of Virus replication, as Determined Against *h*CoV-OC43 by FFRAs in HCT-8 Cells. Reported Values are the Means ± SD of Data Derived from Five Experiments Performed in Triplicate.

c CC_50_, Compound Concentration Producing 50% Cytotoxicity, as Determined by Cell Viability Assays Performed in HCT-8 Cells. Reported Values are the Means ± SD of Data Derived from Five Experiments Performed in Triplicate.

d SI, Selectivity Index Determined as the Ratio of CC_50_ to EC_50_.

### Photophysical Characterization

To prepare the following cell-based investigation for assessing the subcellular localization of the target compound, fluorescence properties were initially investigated in acetonitrile solution at room temperature (Table 8). Compounds **13** and **14**, derived from the compound **7** series, show absorbance and emission comparable to the lead SFR-optimized compound **7b**. Additionally, they display generally acceptable fluorescence features, making them suitable for an initial investigation in a biological system. Compounds **11a** and **11b**, ideally developed from compound **5**, exhibit yellow emission at 535 and 542 nm, respectively. Surprisingly, the QY value is restored in these two molecules, with compound **11a** showing the most favorable outcome. In this case, the insertion of a strong EWG, such as a tetrafluorobiphenyl group, together with the substitution of fluorine atoms in the ortho position relative to the olefin group has significant effects on the photophysical properties of compounds **11a**–**b**. In contrast to the compounds of the 5 series, the strong EWG tetrafluorobiphenyl group can stabilize the excited state by lowering its energy, making the fluorescence more competitive against nonradiative decay processes.^76^ In addition, the steric hindrance provided by the *diortho-fluoro* substituents on the olefin group increases the rigidity of the molecular structure. It restricts free rotation of the substituents around the carbon–carbon double bond, a common pathway for nonradiative decay.

**Table 8:** Spectral Properties of Compounds **11a–b,** and **12, 13,** and **14** in ACN

Compound	λ_abs_ (nm)	λ_em_ (nm)	Stokes shift (cm^–1^)	QY	Lifetime (ns)
**11a**	442	542	4174	0.12	<1
**11b**	450	535	3531	0.08	<1
**12**	390	430	2385	<0.01	2.3
**13**	370	460	5288	0.74	3.7
**14**	390	465	4136	0.64	3.7

By preventing this rotation, the molecule is less likely to dissipate its energy through nonradiative means such as internal conversion or vibrational relaxation.

Due to an emission profile compatible with biological visualization and a good QY value, the fluorescent properties of **11a** and **14** were studied in PBS solution to evaluate the fluorescence in a polar system. As outlined in Figure 10, the emission of both compounds is completely quenched in polar protic solvent following a behavior that can sometimes be observed in so-called *environmentally sensitive fluorescent probes*. Fluorescent molecules often lose their fluorescence in polar environments for several reasons related to solvent interactions, but they can maintain or even enhance their fluorescence when bound to proteins.^200^ This differential behavior can be explained through various photophysical and structural mechanisms such as *twisted intramolecular charge transfer* (TICT), quenching resulting from aggregation, or *intramolecular proton transfer to the excited state* (ESIPT).^77–80^ Nevertheless, this feature can be highly advantageous when the design aims to apply the probe within a biological system. Indeed, in most cases, when the ligand binding site is located in a lipophilic environment, the probe can recover its fluorescence once it reaches the target. This feature makes these fluorescent probes “*smart probes*” able to highlight the binding of a molecule to its target or act as a sensor to detect the local biological environment.

**Figure 10:**
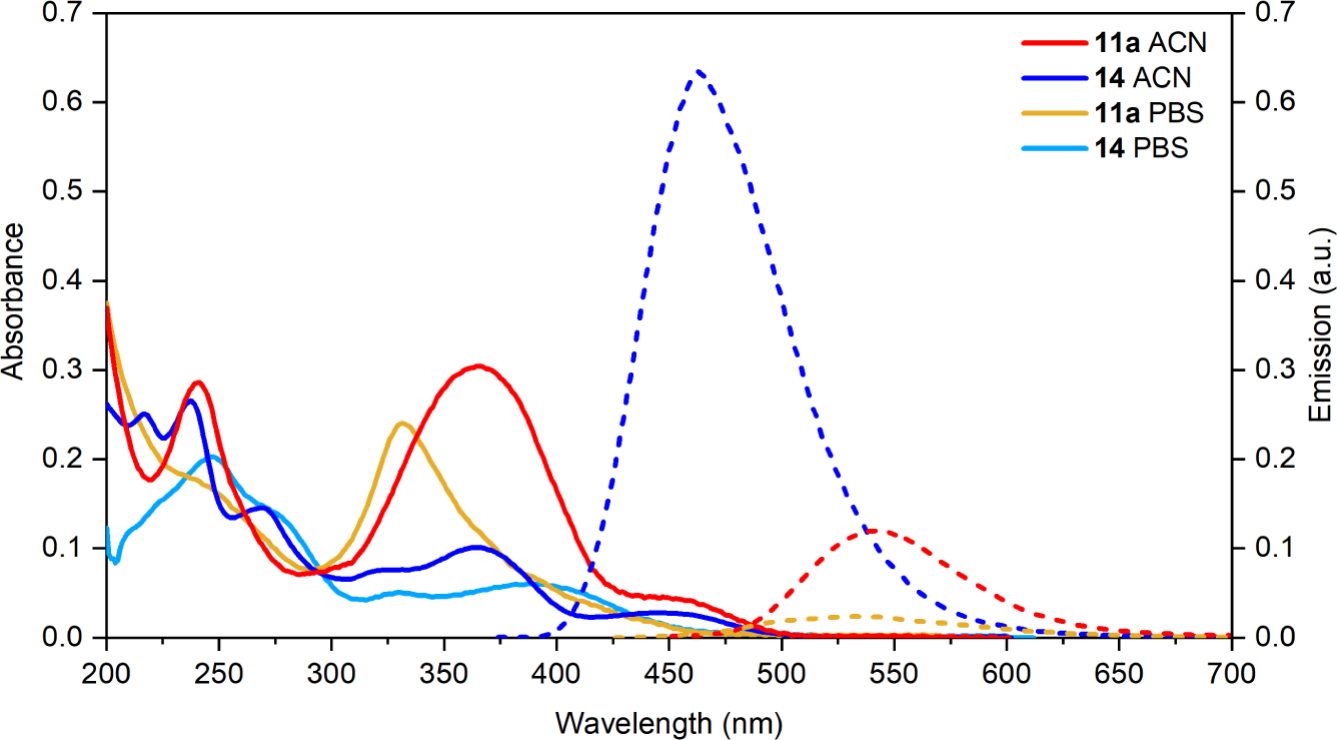
Absorption and emission spectra of compounds **11a** and **14** in ACN and PBS solution. Plain lines represent the absorption profiles, dashed lines correspond to the emission profiles, **14** is not emissive in PBS.

To assess whether the developed compounds are able to restore their fluorescence in a biological context and to evaluate their potential as environmentally sensitive fluorescent probes, we examined the fluorescence response of compound **11a**, the most potent inhibitor within the series, in the presence of a target protein. We evaluated the fluorescence of compound **11a** in condition of *h*DHODH enzymatic inhibition assay, confirming the absence of emission even in TRIS buffer (pH 8), and then observing the restoration of fluorescence in the presence of the recombinant protein. In particular, we observed an increase in fluorescence intensity in correlation with the growing amount of protein, as shown in Figure 11.

**Figure 11:**
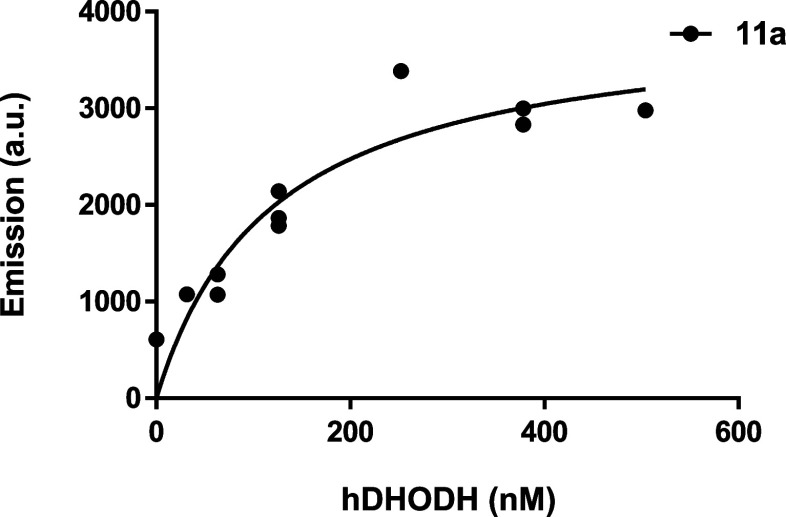
Variation of in the fluorescence emission of compound **11a** (1 μM) in the presence of different amount of *h*DHODH.

Because the isolated *h*DHODH used for the previous measurements, and in inhibition enzymatic assay, is the GST-tagged protein, we decided to confirm that the observed fluorescence enhancement resulted from specific binding of inhibitor **11a** to the target protein and not from nonspecific interactions with the GST portion. When in a control experiment an unrelated GST-conjugated-protein was used, no restoration of fluorescence was observed supporting the conclusion that fluorescence is restored solely by the binding of compound **11a** to its specific target protein (Figure S5).

### Intracellular Localization of Target Compounds **11a** and **14**

The results presented in the previous sections highlight that compounds **11a** and **14** behave as environmentally sensitive ligands endowed with a sufficient affinity for the target and, importantly, exert two different biological activities typical of *h*DHODH inhibitors (Tables 5 and 6, and Figure 9). Thus, compounds **11a** and **14** were subsequently examined in the context of living cells to determine whether they retained their fluorescence profile after entry into target cells. First, both compounds were incubated for 24 h with THP-1 cells at a concentration of 10 μM. After washing with PBS buffer, cells were analyzed under confocal microscopy using an appropriate laser stimulation (λ_ex_: 405 nm, λ_em_: 415 and 500 nm for compounds **11a** and **14**, respectively) to excite the molecules. As shown in Figure 12, both compounds *recovered* a strong fluorescence, confirming that they exhibited typical features of *environment-sensitive fluorescent* probes. Moreover, it was possible to observe that both compounds localize in the cytosol of THP-1 cells.

**Figure 12:**
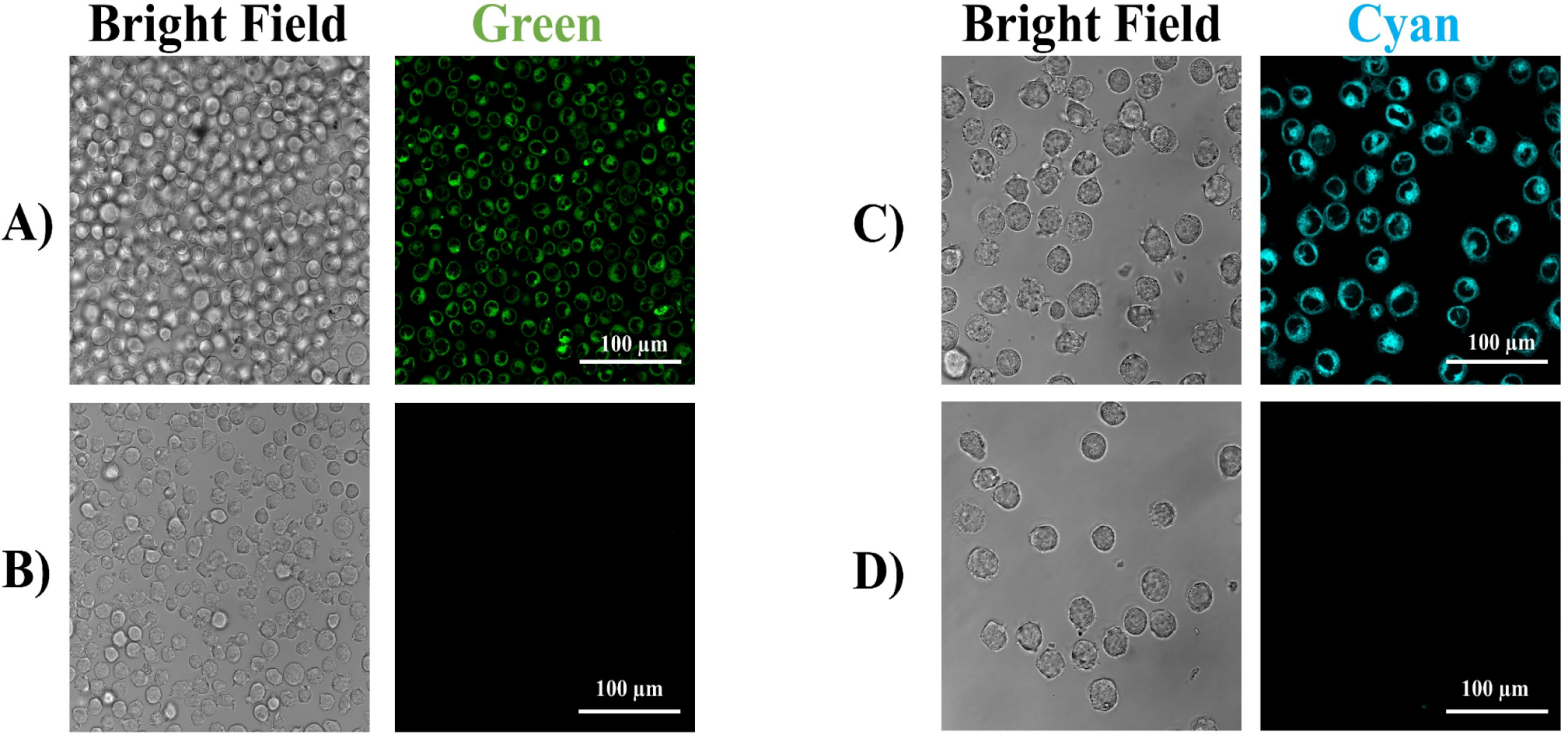
Fluorescence microscopy imaging of THP-1 cells incubated with 10 μM of **11a** and **14**. Images were collected Leica TCS SP8 confocal system (Leica Microsystems) and acquired with an HCX PL APO 63×/1.4 NA oil-immersion. (A) THP-1 cells incubated with **11a** (green); (B) THP-1 cells not treated; (C) THP-1 incubated with **14** (cyan); (D) THP-1 not treated. White scale bar = 100 μm.

Co-localization experiments were performed to examine whether compound **11a** may reach the cellular compartment where *h*DHODH is located. For this, a specific mitochondrial stain marker in the two cell lines in which the biological activities of **11a** were observed, namely THP-1 and HCT-8 cells, was used. The compound was therefore incubated with THP-1 cells (Figure 13) or HTC-8 cells (Figure 14) at 37 °C for 24 h. Then, cells were stained with *Red-MitoTracker* (a specific fluorescent probe for mitochondrial targeting) for 30 min at 37 °C. In both THP-1 and HCT-8 cells, merging images obtained using an appropriate laser to excite the molecules confirms the ability of compound **11a** to localize into mitochondria, where its protein target is present.

**Figure 13:**
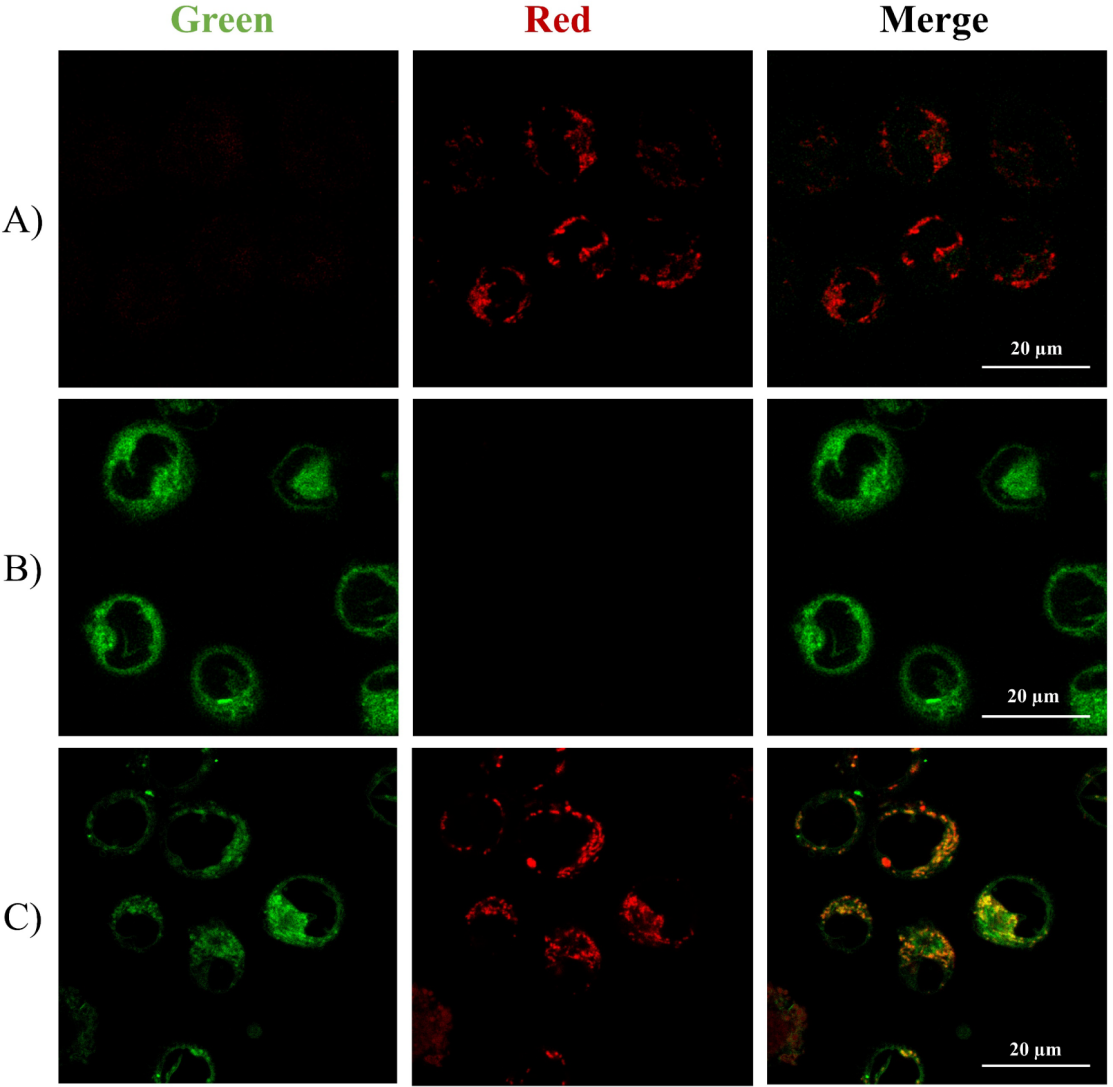
Intracellular localization of the fluorescent inhibitor **11a** and *Red-MitoTracker* (mitochondria-specific dye) in THP-1 cells. Images were collected with a Leica TCS SP8 confocal system (Leica Microsystems) and acquired with a HCX PL APO 63×/1.4 NA oil-immersion: (A) THP-1 cells were incubated with DMSO for 24 h as negative control, and then staining with *Red-MitoTracker* for cellular localization of mitochondria (30 min at 37 °C); (B) THP-1 cells were treated with 10 μM of **11a** for 24 h to assess its cellular localization; (C) THP-1 cells were treated compound **11a** for 24 h, and then staining with *Red-MitoTracker* (30 min at 37 °C). Images were collected with excitation of ligand **11a** at λ_ex_ = 405 nm and emission at λ_em_ = 500 nm, *Red-MitoTracker* at λ_ex_ = 581 nm, and emission at λ_em_ = 605 nm. White scale bar = 20 μm.

**Figure 14:**
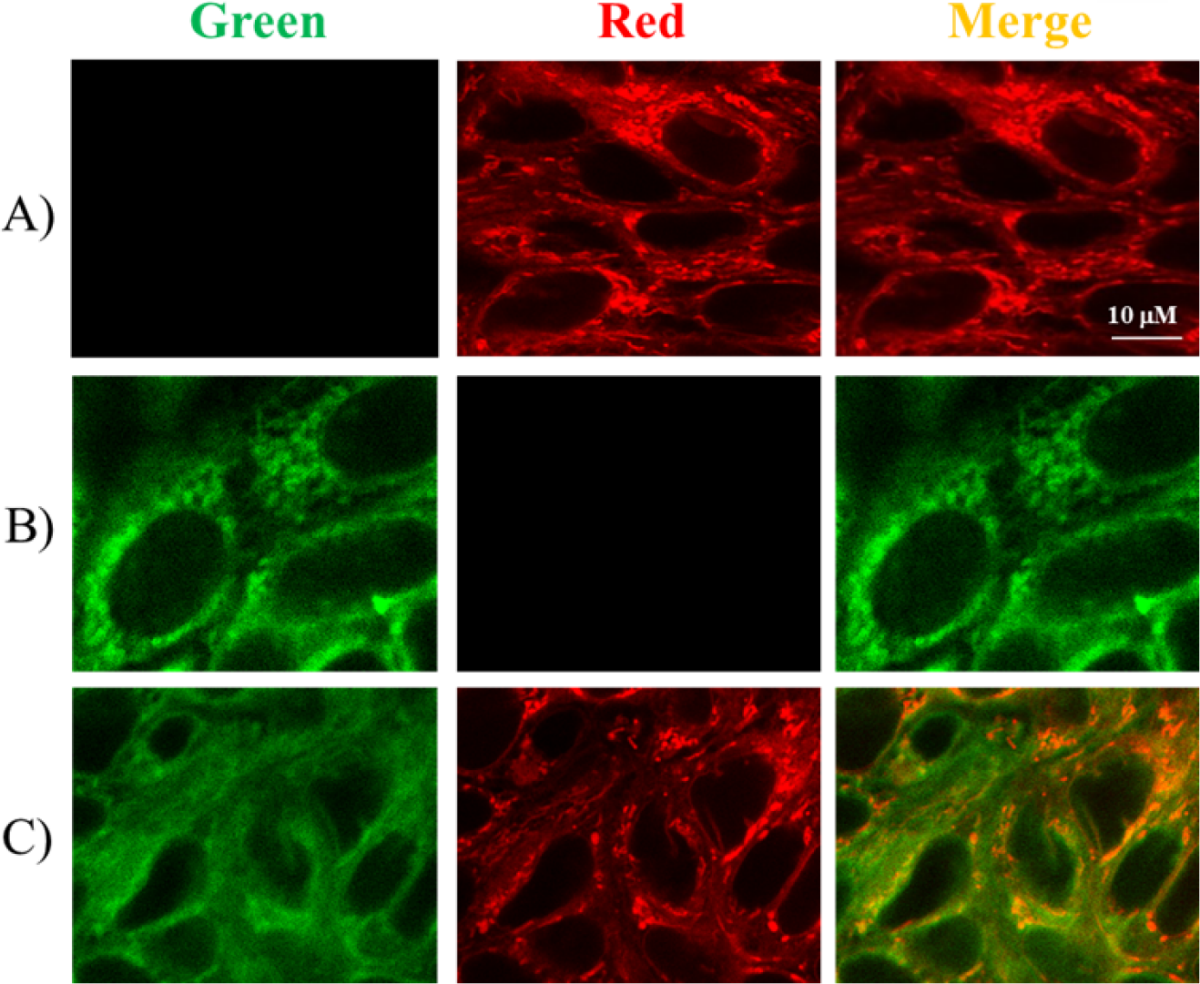
Intracellular localization of the fluorescent inhibitor **11a** and *Red-MitoTracker* (mitochondria-specific dye) in HTC-8 cells. (A) HTC-8 cells were treated with DMSO for 24 h as a negative control and then stained with *Red-MitoTracker* for intracellular localization of mitochondria (30 min at 37 °C); (B) HTC-8 cells were treated with 10 μM of **11a** for 24 h; (C) HTC-8 cells were treated with 10 μM of **11a** for 24 h, and then stained with *Red-MitoTracker* (30 min at 37 °C). Images were acquired by a Leica TCS SP5 multiphoton-inverted confocal microscope (Leica Microsystems) equipped with an HCX PL APO 63×/1.4 NA oil-immersion. Images were acquired with excitation for ligand **11a** at λex = 405 nm and emission at λem = 500 nm, for *Red-MitoTracker* at λex = 581 nm and emission at λem = 605 nm. White scale bar = 10 μm.

To confirm the mitochondrial localization of compound **11a**, we performed a competition assay involving a well-known *h*DHODH inhibitor (**Bay2402234)** with higher affinity for the target (one digit nM) compared to **11a**. THP-1 cells were then incubated with compound **11a**, either alone or in combination with **Bay2402234**, followed by *Red-MitoTracker* staining to label mitochondria and subsequent analysis by fluorescence microscopy (Figure 15A-C). At the specific wavelength for compound **11a**, it colocalized with the mitochondria, while **Bay2402234** showed no detectable fluorescence (Figure 15A-B). Colocalization between *h*DHODH inhibitors and mitochondria was quantified using Manders coefficient (Figure 15D). In the presence of **Bay2402234**, the colocalization signal of **11a** with the mitochondria decreased slightly but statistically significantly, as indicated by the Welch’s *t* test (*p* = 0.0024). Altogether, the data demonstrate that **Bay2402234** competitively displaced compound **11a** from the target, confirming both the mitochondria localization and *h*DHODH binding specificity of compound **11a.**

**Figure 15:**
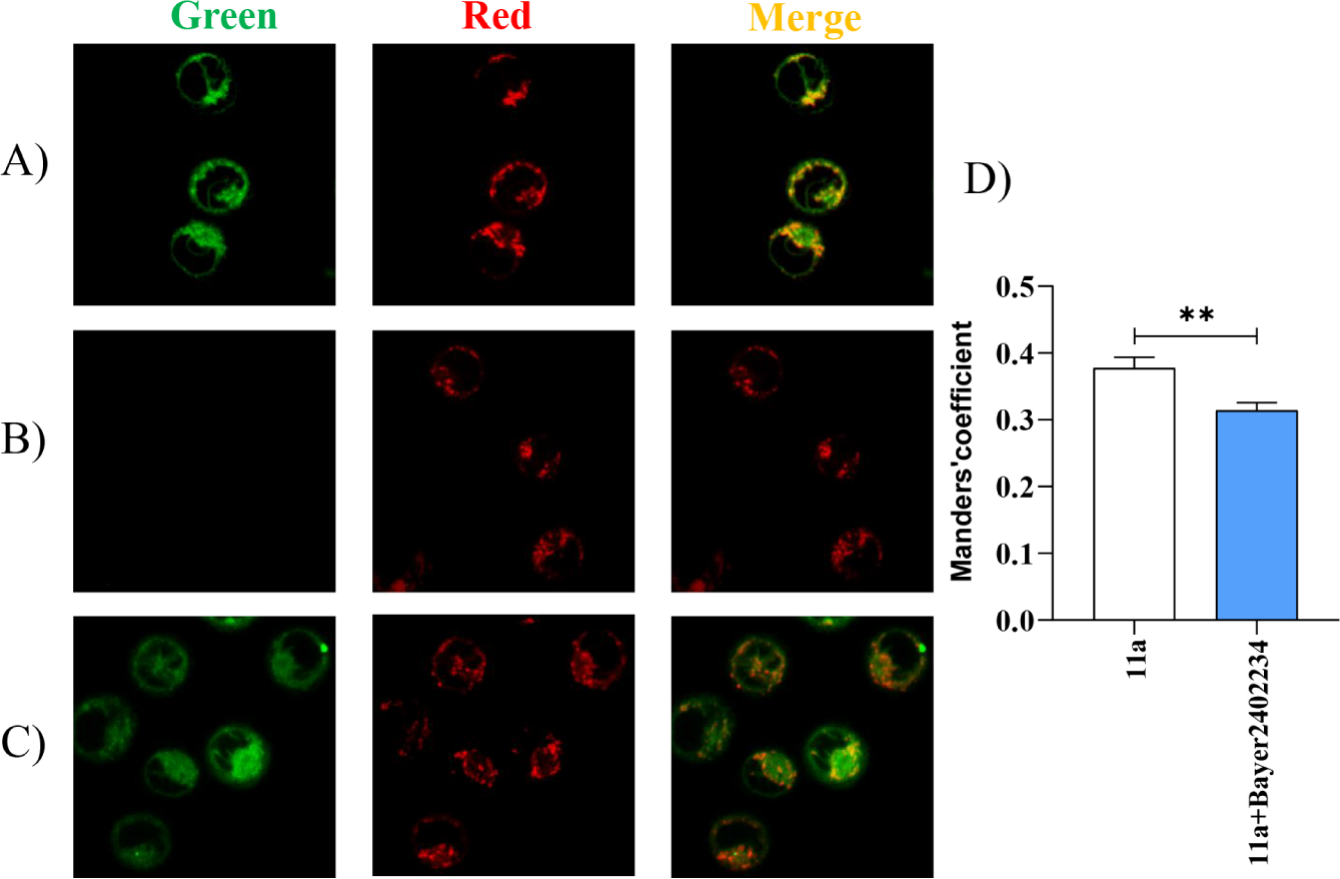
Colocalization analysis of fluorescent inhibitor **11a** with mitochondria of THP-1 cells. THP-1 cells were treated with *h*DHODH inhibitors at 10 μM for 3 h and then staining with *Red-MitoTracker* for 30 min at 37 °C. Images were collected with a Leica TCS SP8 confocal system (Leica Microsystems) and acquired with an HCX PL APO 63×/1.4 NA oil-immersion. Scale bar = 10 μm. (A) THP-1 cells were treated with **11a**; (B) THP-1 cells were treated with **Bayer2402234;** (C) THP-1 cells were treated with **11a** and **Bayer2402234;** (D) Manders colocalization coefficient of **11a** with mitochondria labeled by *Red-MitoTracker*. Statistical significance was tested with Welch’s *t* test (*n* = 3).

### Conclusions

This study introduces the first fluorescent isostere of carboxylic acid, establishing “*fluo-isosteres*” (*fluosteres*) as a novel subclass of isosteres with optimized fluorescence. The successful design of the first fluorescent *h*DHODH inhibitors, compounds **11a** and **14**, provides *proof-of-concept* for the *fluostere* technology, demonstrating its ability to create potent inhibitors optimized for fluorescent properties and able to prove cellular-based activities, as antiviral agents in the micromolar range. Interestingly, both **11a** and **14** were identified as *environmentally sensitive probes*, losing their emission in protic solvents but recovering fluorescence in biological systems by interacting with the target in a lipophilic environment. In colocalization experiments with the *Red-MitoTracker* in THP-1 and HTC-8 cell lines, compound **11a** confirmed its ability to reach the mitochondria, where its biological target *h*DHODH is situated.

Quite interestingly, the possibility to alkylate the OH group of the pyrazolo­[1,5*-a*]­pyridine without losing the optimal fluorescent profile allows the possibility to use such a moiety also as *probe*. In the near future, the *fluostere* technology will be applied to explore additional SFR and enhance its precision as a research tool.

## Experimental Section

### General Methods

All chemical reagents were obtained from commercial sources (Sigma-Aldrich, Alfa Aesar, FluoroChem and BLD Pharma), and used without further purification. Analytical-grade solvents (acetonitrile [ACN], diisopropyl ether, diethyl ether, dichloromethane [DCM], dimethylformamide [DMF], ethanol 99.8% v/v, ethyl acetate [EtOAc], hexane, methanol [MeOH], *petroleum ether* bp 40–60 °C [*petroleum ether*], toluene), were used without further purification. When needed, solvents were dried over 4 Å molecular sieves. Tetrahydrofuran [THF] was distilled from Na and benzophenone under N_2_ immediately prior to use. Anhydrous THF, DCM and DMF were obtained from a Glass Contour Solvent System (SG Water USA). Anhydrous MeOH was obtained by distillation over CaCl_2_ and storage over activated 3 Å molecular sieves for a minimum of 24 h. Thin layer chromatography (TLC) was conducted on silica gel on 5 × 20 cm plates at a 0.25 mm layer thickness were used and visualized using UV-light (254 nm) or by TLC visualization reagents (KMnO_4_, ninhydrin, iodine and 2,4-dinitrophenylhydrazine). Anhydrous Na_2_SO_4_ was used as a drying agent for the organic phases. Compound purification was either achieved using flash column chromatography on silica gel (Merck Kieselgel 60, 230–400 mesh ASTM), and the eluents indicated in the procedures for each compound or using CombiFlash Rf 200 (Teledyne Isco) with 5–200 mL/min, 200 psi (with an automatic injection valve), and RediSep Rf Silica columns (Teledyne Isco), with the eluents indicated in the procedures for each compound. Compounds synthesized in our laboratory generally varied between 90% and 99% purity. Purity was assessed by analytical HPLC on an UltiMate HPLC system (Thermo Scientific) consisting of an LPG-3400A pump (1 mL/min), a WPS-3000SL autosampler, and a DAD- 3000D diode array detector using a Gemini- NX C18 column (4.6 × 250 mm, 3 μm, 110 Å); gradient elution 0 to 100% B (ACN/H_2_O/TFA, 90:10:0.1) in solvent A (H_2_O/TFA, 100:0.1) over 15 min. Data were acquired and processed using Chromeleon Software v. 6.80. Analytical purity is ≥95% unless stated otherwise; retention times (tR) are indicated. Preparative HPLC purification was carried out on a Dionex Ultimate 3000 HLPC system consisting of an LPG-3200BX pump (20 mL/min), a Rheodyne 9725i injector, a 10 mL loop, an MWD-3000SD detector (200, 210, 254, and 281 nm), and an AFC-3000SD automated fraction collector using a Gemini-NX C18 column (21.2 ´ 250 mm, 5 μm, 110 Å); gradient elution was 0 to 80% B (ACN/H_2_O/TFA, 90:10:0.1) in solvent A (H_2_O/TFA, 100:0.1) over 12 min. Data were acquired and processed using Chromeleon Software v. 6.80. Melting points (m.p.), were measured on the capillary apparatus (Büchi 540). Final mp determination was achieved by placing the sample at a temperature that was 10 °C below the mp and applying a heating rate of 1 °C min^1^. All compounds were routinely checked by ^1^H- and ^13^C NMR spectroscopy and mass spectrometry. MS spectra were recorded on a Waters Micromass ZQ equipped with an ESCi source for electrospray ionization mass spectra or using either a LC-MS system built from an Agilent 1200 series solvent delivery system equipped with an autoinjector coupled to a DAD and an Agilent 6130A series quadrupole electrospray ionization detector, or a Waters acquity UPLC-MS equipped with a dual-wavelength PDA (214 and 254 nm) combined with electrospray ionization. Gradients of H_2_O/ACN/HCOOH (95:5:0.1 v/v/v) (solvent A), and ACN/HCOOH (100:0.1 v/v/v) (solvent B) were used. ^1^H–^13^C NMR and 2D-NMR spectra were performed on a JEOL JNM-ECZR 600 spectrometer (^1^H NMR operating frequency 600 MHz) or on a Bruker Avance II spectrometer equipped with a 5 mm broad band probe (BBFO) (^1^H NMR operating frequency 400 MHz) or a Bruker Avance III HD spectrometer equipped with a cryogenically cooled 5 mm dual probe (^1^H NMR operating frequency 600 MHz). Chemical shifts are reported relative to TMS (δ = 0) and referenced against solvent residual peaks. The following abbreviations are used for coupling patterns: *br* = broad, *s* = singlet, *d* = doublet, *dd* = doublet of doublets, *t* = triplet, *q* = quartet, *m* = multiplet. In this work, protons and carbons are labeled (*a, b, c, d, e, f, g, h, l, m, n*, *o, p, q, r, and s*). Values marked with an asterisk (*, ** and ***) are interchangeable. To characterize the E isomer of the double bond, we compare the J coupling constants with those reported in other manuscripts.^81,82^ The HRMS spectra of the final compounds were recorded on a ZenoTOF 7600 System (Sciex, Framingham, MA, U.S.A.) equipped with an ESI ionization source working in positive mode. LC method: direct infusion flow 0.1 mL/min, mobile phase H_2_O (1% TFA)/MeOH 50/50. MS method: spray capillary voltage: 5500 V, declustering potential 40 V, collision energy 10 V TOF mass range scan 100–1000 Da. Compounds **5a**–**d** were obtained according to previously described procedures.^22^ Biological experiments were performed on compounds with a purity of at least 95%.

### (Ethoxycarbonyl)­(pyridin-1-Ium-1-yl)­amide (**15**)

A solution of 1-aminopyridinium iodide (3.0 g, 0.013 mol), diethyl malonate (4.16 g, 0.026 mol), and K_2_CO_3_ (5.39 g, 0.039 mol) in 130 mL of EtOH was stirred at 50 °C overnight. The reaction was then filtered to remove insoluble inorganic salts and the filtrate was concentrated under reduced pressure. The crude mixture was purified by flash chromatography on Al_2_O_2_ (eluent: first diethyl ether, then DCM) to afford the title compound as a colorless sticky solid. Yield 68%. ^1^H NMR (600 MHz, *Chloroform-d*): δ 1.29 (*t*, 3H, J = 7.1 Hz, -OCH_2_C*H*_3_), 3.37 (*s*, 2H), 4.21 (*q*, 2H, J = 7.1 Hz, -OC*H*_2_CH_3_), 7.63 – 7.68 (*m*, 1H), 7.92 (*tt*, 1H, J = 7.8, 1.3 Hz,), 8.67 – 8.70 (*m*, 1H). ^13^C NMR (151 MHz, *Chloroform-d*): δ 14.4, 43.6, 60.9, 126.2, 137.5, 143.3, 169.9, 170.7. MS (ES^+^): 209 (M+1).

### Ethyl 2-hydroxypyrazolo­[1,5-a]­pyridine-3-carboxylate (**2**)

Potassium tert-butoxide (4.88 g, 0.043 mol) was added portion-wise to a solution of **15** (6.0 g, 0.029 mol) in dry THF (300 mL) at 0 °C. The resulting dark-orange suspension was stirred at room temperature for some minutes until complete conversion was observed, after which it was concentrated under vacuum. The residue was taken with water (200 mL) and acidified to pH 2 using 6 M HCl and then extracted with EtOAc (6 × 150 mL). The organic layers were collected, dried over Na_2_SO_4_, and evaporated under reduced pressure to afford a yellowish crude oil that was purified by flash chromatography (eluent: *petroleum ether*/EtOAc 60/40 v/v), to afford the desired compound as white solid. (mp 150.0–151.3 °C, from MeOH). Yield 70%. ^1^H NMR (600 MHz, *Chloroform-d*): δ 1.44 (*t*, 3H, J = 7.0 Hz, −CH_2_C*H*_3_), 4.42 (*q*, 2H, J = 7.0 Hz, −C*H*_2_CH_3_), 6.88 (*t*, 1H, J = 6.7 Hz, *H-b*), 7.39 (*t*, 1H, J = 7.5 Hz, *H-c*), 7.75 (*d*, 1H, J = 8.8 Hz, *H-d*), 8.34 (*d*, 1H, J = 6.7 Hz, *H-a*), 9.06 (*br s*, 1H, −OH). ^13^C NMR (151 MHz, *Chloroform-d*): δ 14.7 (−OCH_2_ *C*H_3_), 60.6 (−O*C*H_2_CH_3_), 86.4 (*C-f*), 113.3 (*C-b*), 117.4 (*C-d*), 128.3 (*C-c*), 129.5 (*C-a*), 140.4 (*C-e*), 160.3 (*C-h*) *, 167.2 (*C-g*)*. MS (ES^+^): 207 (M+1).

### General Procedure for the Synthesis of Target Compounds **2a** and **2c**

The correspondent alkylating agent (3.20 mmol) was added dropwise to a mixture of **2** (2.91 mmol) and Cs_2_CO_3_ (8.73 mmol) in dry DMF (15 mL). The reaction mixture was stirred overnight at room temperature, and then water (100 mL) was added. The mixture was extracted with EtOAc (3 × 25 mL), and the combined organic layers were dried over Na_2_SO_4_ and evaporated under reduced pressure. Crude compounds were purified following the conditions below.

#### Ethyl 2-methoxypyrazolo­[1,5-a]­pyridine-3-carboxylate (**2a**)

The mixture showed two spots on TLC (eluent: *petroleum ether*/EtOAc 80/20 v/v), ascribed to the two methylated pyrazolo­[1,5-*a*]­pyridine regio-isomers. The desired regio-isomer was achieved after flash chromatography (eluent: *petroleum ether*/EtOAc 80/20 v/v) as a white solid (mp 128.9–129.4 °C, from trituration with diisopropyl ether). Yield 85%. ^1^H NMR (600 MHz, *DMSO-d*_6_): δ 1.29 (*t*, 3H, J = 7.1 Hz, −OCH_2_C*H*_3_), 4.01 (*s*, 3H, −OC*H*_3_) 4.24 (*q*, 2H, J = 7.1 Hz, −OC*H*_2_CH_3_), 7.03 (*td*, 1H, J = 6.9 Hz, 1.4 Hz, *H-b*), 7.54 (*ddd*, 1H, J = 8.8, 7.0, 1.1 Hz, *H-c*), 7.90 (*ddd*, 1H, J = 8.9, 1.4, 1.0 Hz, *H-d*), 8.67 (*dt*, 1H, J = 6.8, 1.1 Hz, *H-a*). ^13^C NMR (151 MHz, *DMSO-d*_6_): δ 14.5 (−OCH_2_ *C*H_3_), 56.5 (−O*C*H_3_) *, 59.0 (−O*C*H_2_CH_3_)*, 86.7 (*C-f*), 113.3 (*C-b*), 117.2 (*C-d*), 128.8 (*C-c*), 129.6 (*C-a*), 142.1 (*C-e*), 161.9 (*C-h*)*, 165.0 (*C-g*)*. MS (ES^+^): 221 (M+1).

#### Ethyl 2-(Benzyloxy) Pyrazolo­[1,5-a]­pyridine-3-carboxylate (**2c**)

The mixture showed two spots on TLC (eluent: *petroleum ether*/EtOAc 80/20 v/v), ascribed to the two benzylated pyrazolo­[1,5-*a*] pyridine regio-isomers. The desired regio-isomer was achieved after flash chromatography (eluent: *petroleum ether*/EtOAc 80/20 v/v) purification as a pale-yellow solid (mp 100.0 – 100.8 °C, from trituration with diisopropyl ether). Yield 75%. ^1^H NMR (600 MHz, *DMSO-d*_6_): δ 1.30 (*t*, J = 7.1 Hz, 3H, OCH_2_C*H*_3_), 4.24 (*q*, J = 7.1 Hz, 2H, −OCH_2_CH_3_), 5.44 (*s*, 2H, −OC*H*_2_Ph), 7.04 (*t*, J = 6.9 Hz, 1H, *H-b*), 7.29–7.45 (*m*, 3H), 7.47–7.59 (*m*, 3H), 7.91 (*d*, J = 8.8 Hz, 1H, *H-d*), 8.67 (*d*, J = 6.8 Hz, 1H, *H-a*). ^13^C NMR (151 MHz, *DMSO-d*_6_): δ 14.4 (−OCH_2_ *C*H_3_), 59.0 (-O*C*H_2_CH_3_), 70.2 (−O*C*H_2_Ph), 87.0 (*C-f*), 113.3 (*C-b*), 117.2 (*C-d*), 127.4, 127.9, 128.3, 128.9, 129.6, 136.6, 142.0 (*C-e*), 161.9 (*C-h*) *, 164.3 (*C-g*)*. MS (ES^+^): 297 (M+1).

### General Procedure for the Synthesis of Target Compounds **2b** and **2d**

A solution of compound **15** (6.58 mmol), K_2_CO_3_ (19.74 mmol), and the correspondent alkylating agent (7.89 mmol) in dry ACN (100 mL) were warm at reflux upon complete conversion of starting material was observed by TLC (eluent: DCM/MeOH 9:1 v/v). The reaction was then filtered to remove insoluble inorganic substances and the filtrate was concentrated under reduced pressure. The crude mixture was purified following the conditions above

#### Ethyl 1-methyl-2-oxo-1,2-dihydropyrazolo­[1,5-a]­pyridine-3-carboxylate (**2b**)

The mixture was solubilized in MeOH (10 mL), and cooled diethyl ether (100 mL) was added. The pale-yellow precipitate was filtered off, affording the desired product as a pale-yellow solid (mp 217.8–224.2 °C dec, from diethyl ether). Yield 90%. ^1^H NMR (600 MHz, *Chloroform-d*): δ 1.38 (*t*, 3H, J = 7.1 Hz, −OCH_2_C*H*_3_), 3.65 (*s*, 3H, −NC*H*_3_), 4.36 (*q*, 2H, J = 7.0 Hz, −OC*H*_2_CH_3_), 6.89 (*td*, 1H, J = 7.0, 1.3 Hz, *H-b*), 7.45 (*ddd*, 1H, J = 8.7, 7.1, 1.0 Hz, *H-c*), 7.89 (*d*, 1H, J = 6.8 Hz, *H-a*), 8.10 (*d*, 1H, J = 8.9 Hz, *H-d*). ^13^C NMR (151 MHz, *Chloroform-d*): δ 14.8 (−CH_2_ *C*H_3_), 28.1 (−N*C*H_3_), 59.7 (−*C*H_2_CH_3_), 85.6 (*C-f*), 112.3 (*C-b*), 117.9 (*C-d*), 122.2, 131.1, 143.5 (*C-e*), 160.5, 164.3. MS (ES^+^): 221 (M+1).

#### Ethyl 1-benzyl-2-oxo-1,2-dihydropyrazolo­[1,5-a]­pyridine-3-carboxylate (**2d**)

Crude mixture was purified by using RediSep Gold Silica Gel disposable flash column, 40 g of silica (eluent: *petroleum ether*/EtOAc from 50/50 to 10/90 v/v) to afford the tile compound as a white solid (mp 172.3–174.0 °C; from trituration with EtOAc/diisopropyl ether 1/1 v/v). Yield 70%. ^1^H NMR (600 MHz, *DMSO-d*_6_): δ 1.28 (*t*, J = 7.1 Hz, 3H, −OCH_2_C*H*_3_), 4.22 (*q*, J = 7.1 Hz, 2H, −OC*H*_2_CH_3_), 5.43 (*s*, 2H, −NC*H*_2_Ph), 6.95 (*td*, J = 7.1, 1.0 Hz, 1H, *H-b*), 7.18–7.38 (*m*, 5H), 7.59 (*t*, J = 8.0 Hz, 1H, *H-c*), 7.92 (*d*, J = 8.8 Hz, 1H, *H-d*), 8.41 (*d*, J = 6.9 Hz, 1H, *H-a*). ^13^C NMR (151 MHz, *DMSO-d*_6_): δ 14.6 (−OCH_2_ *C*H_3_), 43.6 (−N*C*H_2_Ph), 58.5 (−O*C*H_2_C*H*_3_) 84.4 (*C-f*), 112.5 (*C-b*), 116.3 (*C-d*), 125.2 (*C-a*), 127.1, 128.0, 128.9, 132.5, 134.0, 142.7 (*C-e*), 160.0 (*C-g*) *, 163.2 (*C-h*)*. MS (ES^+^): 297 (M+1).

### General Procedure for the Synthesis of Target Compounds **1, 1a–C**

Five M NaOH (10.0 equiv) was added to the correspondent pyrazolo­[1,5-*a*] pyridine analogue solution (2.40 mmol) in EtOH (20 mL). The reaction mixture was stirred at reflux until complete conversion of the starting material was observed by TLC (eluent: *petroleum ether*/EtOAc 60/40 v/v for **1a** and **1c** and DCM/MeOH 9:1 v/v for **1** and **1b**). Subsequently, EtOH was removed under reduced pressure. The white solid was taken up with water and warmed at reflux (for **1**, **1a** and **1c**) or stirred at 0 °C (for **1b**). Then 37% w/w HCl was slowly added until pH 1 was reached. Subsequently, the mixture was extracted with EtOAc (3 × 20 mL for **1**, **1a** and **1c**) and DCM (7 × 20 mL for **1b**). The combined organic layers were dried under Na_2_SO_4_ and evaporated under reduced pressure to afford a crude oil purified following the above-mentioned conditions.

#### Pyrazolo­[1,5-a]­pyridin-2-ol (**1**)

Crude material was purified via flash chromatography (eluent: *petroleum ether*/EtOAc 9/1 v/v) to afford the title compound as a white solid (mp 126.3–126.6 °C, trituration from diisopropyl ether). Yield: 90%. ^1^H NMR (600 MHz, *DMSO-d*_6_): δ 5.73 (*s*, 1H, *H-f*), 6.62 (*td*, 1H, J = 6.8, 1.2 Hz, *H-b*), 7.04–7.10 (*m*, 1H, *H-c*), 7.35 (*d*, 1H, J = 8.8 Hz, *H-d*), 8.33 (*d*, 1H, J = 6.9 Hz, *H-a*), 10.40 (*s*, 1H, -O*H*). ^13^C NMR (151 MHz, *DMSO-d*_6_): δ 79.9 (*C-f*), 109.4 (*C-b*), 115.7 (*C-d*), 123.7 (*C-c*)*, 128.1 (*C-a*)*, 141.0 (*C-e*), 163.9 (*C-g*). MS (ES^+^): 135 (M+1).

#### 2-Methoxypyrazolo­[1,5-a]­pyridine (**1a**)

The crude material was purified by combiflash using RediSep Gold Silica Gel disposable flash column, 24 g (eluent: *petroleum ether*/EtOAc from 100 to 80/20 v/v) to afford the title compound as a colorless oil. Yield 90%. ^1^H NMR (400 MHz, *Chloroform-d*): δ 4.00 (*s*, 3H, −OC*H*_3_), 5.83 (*s*, 1H, *H-f*), 6.59 (*td*, 1H, J = 6.9, 1.3 Hz, *H-b*), 7.04 (*ddd*, 1H, J = 8.8, 6.8, 1.0 Hz, *H-c*), 7.29 (*d*, 1H, J = 8.9 Hz, *H-d*), 8.24 (*d*, 1H, J = 7.0 Hz, *H-a*). ^13^C NMR (100 MHz, *Chloroform-d*): δ 56.8 (−O*C*H_3_), 79.6 (*C-f*), 109.8 (*C-b*), 116.5 (*C-d*), 124.1 (*C-c*) *, 128.5 (*C-a*)*, 141.9 (*C-e*), 166.4 (*C-g*). MS (ES^+^): 149 (M+1).

#### 1*-*Methylpyrazolo­[1,5-a]­pyridin-2­(1*H*)-one (**1b**)

The crude material was purified by combiflash using RediSep Gold Silica Gel disposable flash column (12 g, eluent: DCM/MeOH 95/5 v/v) to afford the title compound as a pale-pink solid. Yield 75%. ^1^H NMR (400 MHz, *DMSO-d*_6_): δ 3.53 (*s*, 3H, −NC*H*_3_), 5.13 (*s*, 1H, *H-f*), 6.60 (*td*, 1H, J = 6.7, 1.7 Hz, *H-b*), 7.16–7.22 (*m*, 2H, *H-c and d*) 8.25 (*d*, 1H, J = 6.9 Hz, *H-a*). ^13^C NMR (100 MHz, *DMSO-d*_6_): δ 27.7 (−N*C*H_3_), 77.6 (*C-f*), 107.5 (*C-b*), 114.2 (*C-d*), 124.2 (*C-c*) *, 128.3 (*C-a*)*, 142.2 (*C-e*), 162.2 (*C-g*). MS (ES^+^): 149 (M+1).

#### 2*-*Benzyloxy-pyrazolo­[1,5-a]­pyridine (**1c**)

This compound was synthesized according to the general procedure and the specific procedure outlined below. Benzyl bromide (0.701 g, 4.10 mmol) was added to a mixture of pyrazolo­[1,5-*a*] pyridin-2-ol (**1**, 0.500 g, 3.73 mmol) and Cs_2_CO_3_ (3.64 g, 11.2 mmol) in dry DMF (10 mL). The reaction mixture was stirred overnight at room temperature, and then water (100 mL) was added. The mixture was extracted with EtOAc (4 × 50 mL), then the combined organic layers were dried over Na_2_SO_4_ and evaporated under reduced pressure to afford a yellow oil. The mixture was purified using flash chromatography (eluent: *petroleum ether*/EtOAc 2/1 v/v) to afford the title compound as a white solid (mp 140.8–142.8 °C, trituration from diisopropyl ether). Yield 61%. ^1^H NMR (600 MHz, *Chloroform-d*) δ: 5.34 (*s*, 2H, −OC*H*_2_Ph), 5.88 (*s*, 1H, *H-f*), 6.60 (*t*, J = 6.8 Hz, 1H, *H-b*), 7.01–7.07 (*m*, 1H, *H-c*), 7.29 (*d*, 1H, J = 8.9 Hz, *H-d*), 7.33 (*t*, 1H, J = 7.3 Hz), 7.39 (*t*, 2H, J = 7.5 Hz), 7.50 (*d*, 2H, J = 7.4 Hz), 8.25 (*d*, 1H, J = 6.9 Hz, *H-a*).^13^C NMR (151 MHz, *Chloroform-d*) δ: 71.1 (-O*C*H_2_Ph), 80.4 (*C-f*), 109.9 (*C-b*), 116.6 (*C-d*), 124.1, 127.9, 128.2, 128.5, 128.6, 137.0, 141.8 (*C-e*), 165.6 (*C-g*). MS (ES^+^): 225 (M+1).

### 3-Nitrosopyrazolo­[1,5-a]­pyridin-2-ol (**16**)

To a stirred solution of **1** (0.200 g, 1.49 mmol) in acetic acid (2.0 mL) cooled at 0 °C, a solution of NaNO_2_ (0.123 g, 1.78 mmol) in water (3.0 mL) was added dropwise. The reaction was then stirred at room temperature until a yellow precipitate was observed, then filtrated and the filtrate dried under vacuum. The resulting solid crude was triturated using a mixture of diisopropyl ether and methanol to afford the title compound as a yellow solid. Yield 84%.^1^H NMR (600 MHz, *DMSO-d*_6_): δ 7.66 (*td*, 1H, J = 6.3, 3.2 Hz, *H-b*), 7.84 (*td*, 1H, J = 7.9, 1.1 Hz, *H-c*), 8.22 (*ddd*, 1H, J = 7.9, 1.6, 0.7 Hz, *H-d*), 8.59 (*d*, 1H, J = 6.1 Hz, *H-a*). ^13^C NMR (151 MHz, *DMSO-d*_6_) δ: 123.7, 127.2, 130.4, 131.1, 134.3, 142.9, 169.2. MS (ES^+^): 164 (M+1).

### 3-Nitropyrazolo­[1,5-a]­pyridin-2-ol (**3**)

A solution of H_2_O_2_ (30% w/w in water) was added dropwise to a cooled suspension of compound **16** (0.150 g, 0.915 mmol) in acetic acid (2.0 mL). The reaction mixture was stirred at room temperature upon complete conversion of starting material was observed. The result suspension was filtered, and the solid was washed with water and dried under vacuum, to afford the title compound as a pale orange solid. Yield 84%.^1^H NMR (600 MHz, *DMSO-d*_6_): δ 7.27 (*td*, 1H, J = 7.1, 1.3 Hz, *H-b*), 7.79 (*ddd*, 1H, J = 8.5, 7.3, 0.9 Hz, *H-c*), 8.09 (*d*, 1H, J = 8.7 Hz, *H-d*), 8.73 (*dd*, 1H, J = 6.7, 1.1 Hz, *H-a*), 12.62 (*s*, 1H, -O*H*).^13^C NMR (151 MHz, *DMSO-d*_6_): δ 110.1 (*C-f*), 116.1, 116.6, 130.2, 132.0, 136.9 (*C-e*), 159.7 (*C-g*). MS (ES^+^): 180 (M+1).

### 2-Methoxy-3-nitropyrazolo­[1,5-a]­pyridine (**17a**) and 1-Methyl-3-nitropyrazolo­[1,5-a]­pyridine-2­(1H)-one (**17b**)

Methyl iodide (4.4 mmol) was added dropwise to a mixture of **3** (0.400 g, 2.2 mmol) and Cs_2_CO_3_ (2.15 g, 6.6 mmol) in dry DMF (15.0 mL). The reaction mixture was stirred overnight at room temperature, and then water (100 mL) was added. The reaction mixture was extracted with EtOAc (8 × 25 mL), and the combined organic layers were dried over Na_2_SO_4_ and evaporated under reduced pressure. The resulting crude mixture showed two spots on TLC (eluent: petroleum DCM/MeOH 90/10 v/v), ascribed to the two pyrazolo­[1,5-*a*]-pyridine regio-isomers. Both regio-isomers were achieved after flash chromatography (eluent: DCM/MeOH from 95/5 to 80/20 v/v) as a pale-yellow solid (**17a**) and green solid (**17b**).

**17a**) First isomer eluted. Yield 70%. ^1^H NMR (400 MHz, *DMSO-d*_6_): δ 4.12 (*s*, 3H, −OC*H*_3_), 7.33 (*dt*, 1H, J = 7.0, 1.4 Hz, *H-b*), 7.87 (*ddd*, 1H, J = 8.7, 7.2, 1.1 Hz, *H-c*), 8.15 (*ddd*, 1H, J = 8.8, 1.4, 1.0 Hz, *H-d*), 8.87 (*dt*, 1H, J = 6.7, 1.0 Hz, *H-a*). ^13^C NMR (151 MHz, *DMSO-d*_6_): δ 57.4 (−O*C*H_3_), 110.1 (*C-f*), 116.3 (*C-b*), 116.8 (*C-d*), 130.8 (*C-a*), 132.8 (*C-c*), 137.5 (*C-e*), 160.1 (*C-g*). MS (ES^+^): 194 (M+1).

**17b**) Second isomer eluted. Yield 30%. ^1^H NMR (600 MHz, *DMSO-d*_6_): δ 3.61 (*s*, 3H, −NC*H*_3_), 7.41 (*td*, 1H, J = 7.4, 1.4 Hz, *H-b*), 7.95 (*ddd*, 1H, J = 8.6, 7.4, 1.0 Hz, *H-c*), 8.21 (*ddd*, 1H, J = 8.7, 1.3, 0.8 Hz, *H-d*), 8.79 (*d*, 1H, J = 6.8 Hz, *H-a*).^13^C NMR (151 MHz, *DMSO-d*_6_): δ 28.5\(−N*C*H_3_), 108.7 (*C-f*), 115.9, 116.1, 125.9, 134.9, 136.4 (*C-e*), 154.4 (*C-g*). MS (ES^+^): 194 (M+1).

### *N*-(2-methoxypyrazolo­[1,5-a]­pyridine-3-yl)­acetamide (**3a**)

Acetyl chloride (0.108 g, 1.38 mmol), triethylamine (0.379 g, 3.75 mmol), and dry DMF (7.0 μL) were solubilized under N_2_ atmosphere in 10 mL of dry THF. The solution was cooled down at 0 °C and then, **18** (0.250 g, 1.25 mmol) solubilized in 5.0 mL of dry THF was slowly added to the mixture, keeping the temperature below 0 °C. The reaction mixture was stirred at room temperature upon complete conversion of starting material was observed by TLC (eluent: *petroleum ether*/EtOAc 6:4 v/v). The mixture was then quenched in NH_4_Cl saturated solution (100 mL) and extracted with EtOAc (3 × 25 mL). The combined organic layers were collected, dried over Na_2_SO_4_ and concentrated under reduced pressure. The crude material was purified by flash chromatography (eluent*: petroleum ether*/acetone from 100 to 70/30 v/v) to afford the title compound as a white solid. Yield 75%. ^1^H NMR (600 MHz, *DMSO-d*_6_): δ 2.02 (*s*, 3H, −CONHC*H*_3_), 3.94 (*s*, 3H, −OC*H*_3_), 6.69 (*t,* 1H, J = 6.5 Hz, *H-b*), 7.09–7.15 (*m*, 1H, *H-c*), 7.20 (*d*, 1H, J = 8.9, *H-d*), 8.42 (*d*, 1H, J = 6.8 Hz, *H-a*). 9.21 (s, 1H, – CON*H*CH_3_). ^13^C NMR (151 MHz, *DMSO-d*_6_): δ 22.5 (−CONH*C*H_3_), 56.1 (−O*C*H_3_), 92.9 (*C-f*), 109.8 (*C-b*), 115.5 (*C-d*), 123.9, 128.7, 136.6 (*C-e*), 159.2, 169.0 (*C-g*). MS (ES^+^): 206 (M+1).

### *N*-(2-methoxypyrazolo­[1,5-a]­pyridin-3-yl)­methanesulfonamide (**3b**)

Methane sulfonyl chloride (0.233 g, 2.04 mmol), triethylamine (0.561 g, 5.55 mmol) and dry DMF (7.0 μL) were solubilized under N_2_ atmosphere in 10 mL of dry THF. The solution was cooled down and then, **18** (0.370 g, 1.85 mmol) solubilized in 5 mL of dry THF was slowly added to the mixture, keeping the temperature below 0 °C. The reaction was stirred at room temperature upon complete conversion of starting material was observed by TLC (eluent: *petroleum ether*/EtOAc 6:4 v/v). Then, the mixture was quenched in NH_4_Cl saturated solution (100 mL) and extracted with EtOAc (3 × 25 mL). The combined organic layers were dried over Na_2_SO_4_ and concentrated under reduced pressure. The crude was purified by flash chromatography (eluent*: petroleum ether*/acetone from 100 to 50/50 v/v) to afford the title compound as a white solid. Yield 78%.^1^H NMR (600 MHz, *DMSO-d*_6_): δ 2.94 (*s*, 3H, −SO_2_C*H*_3_), 3.98 (*s,* 3H, −OC*H*_3_), 6.77 (*td*, 1H, J = 6.8, 1.4 Hz, *H-b*), 7.25 (*ddd*, 1H, J = 8.9, 6.8, 1.1 Hz, *H-c*), 7.37–7.40 (*m*, 1H, *H-d*), 8.48 (*dt*, 1H, J = 6.9, 1.0 Hz, *H-a*), 8.91 (*s*, 1H, −SO_2_N*H*).^13^C NMR (151 MHz, *DMSO-d*_6_): δ 40.1 (−SO_2_ *C*H_3_), 56.4 (-O*C*H_3_), 90.9 (*C-f*), 110.7, 114.7, 125.3, 128.9, 139.0 (*C-e*), 160.8 (*C-g*). MS (ES^+^): 242 (M+1).

### 2-Methoxypyrazolo­[1,5-a]­pyridine-3-carbaldehyde (**19**)

A solution of POCl_3_ (2.61 g, 17.04 mmol) in dry DMF (20 mL) was stirred for 30 min at rt under a nitrogen atmosphere. After cooling the mixture to 0 °C, a solution of **1a** (1.0 g, 5.7 mmol) in dry DMF (10 mL) was added dropwise. The reaction mixture was stirred until complete conversion of the starting material was observed by TLC (eluent: *petroleum ether*/EtOAc 8:2 v/v). The reaction mixture was quenched with 1 M NaOH (300 mL), and the precipitate obtained was filtered off. The obtained solid was triturated with diisopropyl ether to afford a white fluffy solid (mp 123.1–124.9 °C, from trituration with diisopropyl ether). Yield 94%. ^1^H NMR (600 MHz, *Chloroform-d*): δ 4.11 (*s*, 3H, −OC*H*_3_), 6.93 (*td*, 1H, J = 6.9, 1.4 Hz, *H-b*), 7.45 (*ddd*, 1H, J = 8.5, 7.1, 1.0 Hz, *H-c*), 8.11 (*d*, 1H, J = 8.7 Hz, *H-d*), 8.31 (*dt*, 1H, J = 6.8 Hz, *H-a*), 9.90 (*s*, 1H, −CO*H*).^13^C NMR (151 MHz, *Chloroform-d*): δ 56.8 (−O*C*H_3_), 98.8 (*C-f*), 114.1 (*C-b*), 118.3 (*C-d*), 129.2 (*C-c*)*, 129.8 (*C-a*)*, 141.5 (*C-e*), 167.8 (*C-g*), 182.2 (*C-h*). MS (ES^+^): 177 (M+1).

### 2-((2-Methoxypyrazolo­[1,5-a]­pyridin-3-yl)­methylene) (**4a**)

Malonitrile (0.082 g, 1.2 mmol) and ammonium acetate (0.174 g, 2.26 mmol) were dissolved in 5.0 mL of acetic acid. The solution was stirred at r.t. for 30 min and then a solution of **19** (0.200 mg, 1.13 mmol) in acetic acid (2.0 mL) was slowly added. The reaction mixture was heated at reflux upon complete conversion of the starting material was observed. The precipitate formed during the reaction was filtered and washed several times with water to afford the title compound as a pale-yellow solid (mp 206.2 – 208.3 °C, from water). Yield 80%.^1^H NMR (600 MHz, *Chloroform-d*): δ 4.13 (*s*, 3H, −OC*H*_3_), 7.05 (*td*, 1H, J = 7.0, 1.2 Hz, *H-b*), 7.56 (*ddd*, 1H, J = 8.6, 7.2, 1.1 Hz, *H-c*), 7.67 (*s*, 1H, −C*H*C­(CN)_2_), 8.18 (*d*, 1H, J = 8.9 Hz, *H-d*), 8.37 (*dt*, 1H, J = 6.8, 1.1 Hz, *H-a*). ^13^C NMR (151 MHz, *Chloroform-d*): δ 57.3 (-O*C*H_3_), 70.5, 94.4 (*C-f*), 115.1 (*C-b*), 116.0, 116.6, 119.1 (*C-d*), 130.5 (*C-a*), 130.6 (*C-c*), 140.7 (*C-e*), 147.2, 166.7 (*C-g*). MS (ES^+^): 225 (M+1).

### Methyl (e)-2-cyano-3-(2-methoxypyrazolo­[1,5-a]­pyridin-3-yl)­acrylate (**4b**)

Methyl 2-cyanoacetate (0.092 g, 0.93 mmol) and ammonium acetate (0.131 g, 1.7 mmol) were dissolved in 5.0 mL of acetic acid. The solution was stirred at r.t. for 30 min and then a solution of 2-methoxypyrazolo­[1,5-*a*] pyridine-3-carbaldehyde (0.150 g, 0.85 mmol) in acetic acid (2.0 mL) was slowly added. The mixture was stirred at reflux upon complete conversion of starting material was observed. The reaction mixture was cooled to room temperature and the observed precipitate was filtered off, washed several times with water, and dried in desiccator to afford the title compound as a pale-yellow solid (mp 209.4 – 210.0 °C, from water). Yield 75%. ^1^H NMR (600 MHz, *Chloroform-d*): δ 3.89 (*s*, 3H, −COOC*H*_3_)*, 4.12 (*s*, 3H, −OC*H*_3_)*, 6.97 (*t*, 1H, J = 6.9 Hz, *H-b*), 7.49 (*t*, 1H, J = 8.0 Hz, *H-c*), 8.29–8.33 (*m*, 2H,) 8.34 (*d*, 1H, J = 6.7, *H-a*).^13^C NMR (151 MHz, *Chloroform-d*): δ 52.9 (−COO*C*H_3_)**, 57.1 (−O*C*H_3_)**, 92.1 (*C-f*), 93.5, 114.3 (*C-b*), 118.5, 119.5 (*C-d*), 129.5 (*C-c*), 130.2 (*C-a*), 140.8 (*C-e*), 144.1 (C-*h*), 165.3 (*C-g*)***, 167.1 (*−C*OOCH_3_) ***. MS (ES^+^): 258 (M+1).

### General Procedure for the Synthesis of Compounds **5a–5e**

To a stirred suspension of appropriate benzyl bromide (1.0 mmol) in dry toluene, triphenylphosphine (1.0 mmol) was added under nitrogen atmosphere. The reaction mixture was stirred at 40 °C for 48 h until complete conversion of the starting material was observed. Then, the reaction mixture was cooled to room temperature and the precipitate was filtered under nitrogen atmosphere to afford the appropriate phosphonium salt as a white solid (see supporting info for detailed synthesis and characterization).

#### General Wittig Reaction Procedure for Synthesis of Compounds **5a–e**

A suspension of the correspondent phosphonium salt (**35 a–e** 1.14 mmol) in dry THF (7.0 mL) was cooled at −10 °C with ice/salt bath under nitrogen atmosphere. Then, 1.0 eq. of 1 M solution of LiHMDS in dry THF (1.14 mmol) was added dropwise. The reaction mixture was stirred for 30 min at −10 °C and then, a solution of *2-methoxypyrazolo­[1,5-a]­pyridine-3-carbaldehyde* (1.14 mmol), in dry THF (1.0 mL) was slowly added. The reaction mixture was stirred at reflux until completely conversion of the starting material was observed. The mixture was quenched in a saturated solution of NH_4_Cl (100 mL) and extracted with EtOAc (3 × 50 mL). The combined organic layers were dried under Na_2_SO_4_ and evaporated under reduced pressure to afford a dark oil. The crude product was purified by flash chromatography (see below the conditions).

##### (E)-2-methoxy-3-styrylpyrazolo­[1,5-a]­pyridine (**5a**)

The crude material was purified by flash chromatography (eluent*: petroleum ether*/EtOAc 95/5 v/v) to afford the title compound as a pale-yellow solid (mp 117.5–118.3 °C, from trituration with diisopropyl ether). Yield 45%.^1^H NMR (600 MHz, *Chloroform-d*): δ 4.15 (*s*, 3H, −OC*H*_3_), 6.63 (*td*, 1H, J = 6.8, 1.2 Hz, *H-b*), 7.06–7.16 (*m*, 3H, *H-h, H-i, H-c*) 7.20 (*t*, 1H, J = 7.3 Hz), 7.34 (*d*, 2H, J = 7.7 Hz), 7.50 (*d*, 2H, J = 7.3 Hz), 7.53 (*d*, 1H, J = 8.9 Hz, *H-d*), 8.22 (*d*, 1H, J = 6.9 Hz, *H-a*). ^13^C NMR (151 MHz, *Chloroform-d*): δ 56.6 (−O*C*H_3_), 94.0 (*C-f*), 110.2 (*C-b*), 115.9, 117.0 (*C-d*), 124.8, 125.0, 125.7, 126.5, 128.7, 128.8, 138.9, 139.1, 163.9 (*C-g*). MS (ES^+^): 251 (M+1).

##### (E)-2-methoxy-3-(4-(methylthio)­styryl)­pyrazolo­[1,5-a]­pyridine (**5b**)

The crude material was purified by flash chromatography (eluent*: petroleum ether*/EtOAc 90/10 v/v) to affords a mixture of cis–trans isomer. The pure trans isomer was obtained after purification using preparative HPLC (Method: 20 min, H_2_O 100% MeOH from 0 to 100%) to afford the title compound as a pale-yellow solid (mp 134.8–136.1 °C, from water). Yield 55%. ^1^H NMR (600 MHz, *Chloroform-d*): δ 2.50 (*s*, 3H, −SC*H*_3_), 4.14 (*s*, 3H, −OC*H*_3_), 6.63 (1H, *t*, J = 6.8 Hz, *H-b*), 7.00–7.16 (*m*, 3H), 7.23 (*d*, 2H, J = 8.2 Hz), 7.41 (*d*, 2H, J = 8.2 Hz), 7.52 (*d*, 1H, J = 8.9 Hz, *H-d*), 8.22 (*d*, 1H, J = 6.9 Hz, *H-a*). ^13^C NMR (151 MHz, *Chloroform-d*): δ 16.4 (−S*C*H_3_), 56.7 (−O*C*H_3_), 94.0 (*C-f*), 110.2 (*C-b*), 115.9 (*C-d*), 116.6, 124.4, 124.8, 126.2, 127.3, 128.8, 136.16, 136.17, 139.0 (*C-e*), 163.9 (*C-g*). MS (ES^+^): 297 (M+1).

##### (E)-2-methoxy-3-(4-(methylsulfonyl)­styryl)­pyrazolo­[1,5-a]­pyridine (**5c**)

The crude material was purified by flash chromatography (eluent*: petroleum ether*/EtOAc 95/5 v/v) to afford the title compound as a white solid (mp 195.1–196 °C, from trituration with diisopropyl ether). Yield 56% ^1^H NMR (600 MHz, *Chloroform-d*): δ 3.06 (*s*, 3H, −SO_2_C*H*_3_), 4.16 (*s*, 3H, -OC*H*_3_), 6.69 (*t*, 1H, J = 6.3 Hz, *H-b*), 7.08 (*d*, J = 16.3 Hz, 1H) *, 7.21 (*t*, 1H, J = 7.8 Hz, *H-c*), 7.29 (*d*, 1H, J = 16.3 Hz) *, 7.55 (*d*, 1H, J = 8.8 Hz, *H-d*), 7.62 (*d*, 2H, J = 8.2 Hz) 7.86 (*d*, 2H, J = 8.2 Hz,), 8.24 (*d*, 1H, J = 6.8 Hz, *H-a*). ^13^C NMR (151 MHz, *Chloroform-d*): δ 44.8 (−SO_2_ *C*H_3_), 56.7 (-O*C*H_3_), 93.7 (*C-f*), 110.8 (*C-b*), 115.7 (*C-d*), 121.0, 122.3, 125.7, 126.0, 127.9, 129.0, 137.2, 139.5, 144.7, 164.3 (*C-g*). MS (ES^+^): 329 (M+1).

##### (E)-4-(2-(2-methoxypyrazolo­[1,5-a]­pyridin-3-yl)­vinyl)­benzonitrile (**5d**)

The crude material was purified by flash chromatography (eluent*: petroleum ether*/EtOAc 93/7 v/v) to afford the title compound as a yellow solid (mp 175.7–177 °C, from trituration with diisopropyl ether). Yield 45%. ^1^H NMR (600 MHz, *Chloroform-d*): δ 4.15 (*s*, 3H, −OC*H*_3_), 6.68 (*t*, 1H, J = 6.8 Hz, *H-b*), 7.03 (*d*, 1H, J = 16.3 Hz), 7.16–7.21 (*m*, 1H, *H-c*), 7.24 (*d*, 1H, J = 16.3 Hz), 7.50–7.54 (*m*, 3H), 7.55–7.59 (*m*, 2H), 8.23 (*d*, 1H, J = 6.9 Hz, *H-a*). ^13^C NMR (151 MHz, *Chloroform-d*): δ 56.7 (−O*C*H_3_), 93.7 (*C-f*), 109.0 (*−C*N), 110.8 (*C-b*), 115.6 (*C-d*), 119.6, 120.8, 122.5, 125.6, 125.9, 129.0, 132.5, 139.5, 143.7, 164.3 (*C-g*). MS (ES^+^): 276 (M+1).

##### (E)-2-methoxy-3-(4-nitrostyryl) Pyrazolo­[1,5-a]­pyridine (**5e**)

The crude material was purified by flash chromatography (eluent*: petroleum ether*/EtOAc 95/5 v/v) to afford the title compound as red solid (mp 211.5–213.6 °C, from trituration with diisopropyl ether). Yield: 27%. ^1^H NMR (600 MHz, *Chloroform-d*): δ 4.17 (*s*, 3H, −OC*H*_3_), 6.71 (*t*, 1H, J = 6.6 Hz, *H-b*), 7.10 (*d*, 1H, J = 16.3 Hz), 7.19–7.25 (*m*, 1H, *H-c*), 7.32 (*d*, 1H, J = 16.3 Hz), 7.53–7.60 (*m*, 3H), 8.18 (*d*, 1H, J = 8.6 Hz), 8.25 (*d*, 1H, J = 6.7 Hz, *H-a*). ^13^C NMR (151 MHz, *Chloroform-d*): δ 56.8 (-O*C*H_3_), 93.9 (*C-f*), 111.0 (*C-b*), 115.7 (*C-d*), 121.9, 122.1, 124.3, 125.7, 125.9, 129.2, 139.6, 145.8, 145.9, 164.4 (*C-g*). MS (ES^+^): 296 (M+1).

### 3-Bromo-2-methoxypyrazolo­[1,5-a]­pyridine (**20**)

A solution of 2-methoxypyrazolo­[1,5-*a*] pyridine (0.340 g, 2.29 mmol) in dry DCM (10 mL) was cooled at 0 °C by an ice bath. Then a solution of NBS (0.448 g, 2.52 mmol) in dry DCM (2.0 mL) was slowly dropped into the reaction mixture that was stirred until complete conversion of starting material as observed by TLC (eluent: *petroleum ether*/EtOAc 9:1 v/v). The solvent was removed then under reduced pressure and the reaction crude was purified by flash chromatography (eluent*: petroleum ether*/EtOAc 90/10 v/v) to afford the title compound as a white solid (mp 55.1–55.6 °C, from trituration with diisopropyl ether). Yield 95%. ^1^H NMR (600 MHz, *Chloroform-d*): δ 4.09 (*s*, 3H, −OC*H*_3_), 6.63 (*td*, 1H, J = 6.9, 1.3 Hz, *H-b*), 7.14 (*ddd*, 1H, J = 8.9, 6.7, 1.0 Hz, *H-c*), 7.27–7.30 (*m*, 1H, *H-d*), 8.20 (*dt*, 1H, J = 7.0, 0.9 Hz, *H-a*). ^13^C NMR (151 MHz, *Chloroform-d*): δ 57.0 (−O*C*H_3_), 67.4 (*C-f*), 110.3 (*C-b*), 115.2 (*C-d*), 125.1 (*C-c*) *, 128.9 (*C-a*)*, 139.7 (*C-e*), 162.2 (*C-g*). MS (ES^+^): 227, 229 (M+1).

### General Procedure for the Synthesis of Compounds **6, 7a–c**

[Pd­(PPh_3_)_4_] (4.4 mg, 0.0038 mmol) was added to a solution of 3-bromo-2-methoxypyrazolo­[1,5-*a*]­pyridine (0.086 mg, 0.38 mmol) and Cs_2_CO_3_ (0.371 g, 1.14 mmol,) in dioxane/water (8:2 v/v, 10 mL) solution. After stirring the resulting mixture under nitrogen atmosphere for 1 h, the corresponding boronic acid (0.760 mmol) was added. The reaction mixture was stirred at reflux until complete conversion of starting material as observed by TLC (eluent: *petroleum ether*/EtOAc 8:2 v/v). Then, the reaction mixture was cooled to room temperature and was concentrated under reduced pressure to afford a crude material. The latter was *taken-up* with water (50 mL), and the aqueous solution was extracted with EtOAc (3 × 10 mL). The combined organic layers were dried over Na_2_SO_4_ and concentrated under reduced pressure. The crude material was purified by flash chromatography (see below the conditions).

#### 2-Methoxy-3-(naphthalen-2-yl)­pyrazolo­[1,5-a]­pyridine (**6**)

The mixture was purified by flash chromatography (eluent*: petroleum ether*/EtOAc from 90/10 to 80/20 v/v) to afford the title compound as a white solid (mp 126.8 – 127.5 °C, from trituration with diisopropyl ether). Yield 76%. ^1^H NMR (600 MHz, *Chloroform-d*): δ 4.17 (*s*, 3H, −OC*H*_3_), 6.66 (*td*, 1H, J = 6.8, 1.3 Hz, *H-b*), 7.16 (*ddd*, 1H, J = 8.9, 6.8, 1.1 Hz, *H-c*), 7.42–7.51 (*m*, 2H), 7.71 (*dt*, 1H, J = 9.0, 1.0 Hz, *H-d*), 7.82–7.88 (*m*, 3H), 7.90 (*d*, 1H, J = 8.5 Hz), 8.08 (*d*, 1H, J = 0.6 Hz), 8.31 (*dt*, 1H, J = 6.9, 1.0 Hz, *H-a*). ^13^C NMR (151 MHz, *Chloroform-d*): δ 56.7 (−O*C*H_3_), 95.6 (*C-f*), 110.2 (*C-b*), 116.0 (*C-d*), 125.0, 125.4, 125.6, 126.2, 126.5, 127.78, 127.82, 128.2, 128.9, 129.9, 131.8, 134.0, 139.2 (*C-e*), 163.1 (*C-g*). MS (ES^+^): 275 (M+1).

#### 2-(2-Methoxypyrazolo­[1,5-a]­pyridin-3-yl)­quinoline (**7a**)

The mixture was purified by Combiflash using RediSep Gold Silica Gel disposable flash column, 40 g of silica (eluent*: petroleum ether*/EtOAc from 95/5 v/v to 60/40 v/v) to afford the title compound as a pale-yellow solid (mp 129.2–130.3 °C, from trituration with diisopropyl ether). Yield 78%. ^1^H NMR (600 MHz, *Chloroform-d*): δ 4.22 (*s*, 3H, −OC*H*_3_), 6.79 (*t*, J = 6.7 Hz, 1H, *H-b*), 7.34 (*t,* J = 7.8 Hz, 1H), 7.43 (*t*, J = 7.4 Hz, 1H, *H-c*), 7.66 (*t*, J = 7.6 Hz, 1H), 7.75 (*d*, J = 8.0 Hz, 1H), 8.08 (*t*, J = 8.1 Hz, 2H), 8.16 (*d*, J = 8.7 Hz, 1H, H-d), 8.31 (*d*, J = 6.8 Hz, 1H, *H-a*), 8.89 (*d*, J = 8.9 Hz, 1H). ^13^C NMR (151 MHz, *Chloroform-d*): δ 56.8 (−O*C*H_3_), 95.5 (*C-f*), 111.7 (*C-b*), 119.9 (*C-d*), 120.3, 124.9, 126.0, 126.4, 127.6, 128.5, 128.7, 129.2, 135.7, 141.1, 148.4, 153.7, 164.4 (*C-g*). MS (ES^+^): 276 (M+1).

#### 7-(2-Methoxypyrazolo­[1,5-a]­pyridin-3-yl)­quinoline (**7b**)

The mixture was purified by flash chromatography (eluent*: petroleum ether*/EtOAc from 90/10 v/v to 70/30 v/v) to afford the title compound as a pale-yellow solid (mp 148.1–151.1 °C, from trituration with diisopropyl ether). Yield 70%. ^1^H NMR (600 MHz, *Chloroform-d*): δ 4.17 (*s*, 3H, −OC*H*_3_), 6.70 (*td*, 1H, J = 6.8, 1.2 Hz, *H-b*), 7.19 (*ddd*, 1H, J = 8.9, 6.8, 1.0 Hz, *H-c*), 7.35 (*dd*, 1H, J = 8.2, 4.2 Hz), 7.82 (*d*, 1H, J = 9.0 Hz, *H-d*), 7.85 (*d*, 1H, J = 8.5 Hz), 7.98 (*dd*, 1H, J = 8.5, 1.6 Hz), 8.14 (*d*, 1H, J = 7.4 Hz), 8.31 (*d*, 1H, J = 6.9 Hz, *H-a*), 8.37 (s, 1H), 8.90 (*dd*, 1H, J = 4.1, 1.5 Hz). ^13^C NMR (151 MHz, *Chloroform-d*): δ 56.7 (−O*C*H_3_), 94.9 (*C-f*), 110.6 (*C-b*), 116.1 (*C-d*), 120.5, 125.5, 126.1, 126.5, 127.0, 128.0, 129.0, 134.1, 135.8, 139.3, 148.9, 150.7, 163. (*C-g*). MS (ES^+^): 276 (M+1).

#### 6-(2-Methoxypyrazolo­[1,5-a]­pyridin-3-yl)­quinoline (**7c**)

The mixture was purified by Combiflash using RediSep Gold Silica Gel disposable flash column, 40 g of silica (eluent*: petroleum ether*/EtOAc from 95/5 v/v to 70/30 v/v) to afford the title compound as a pale-yellow solid (mp 147.5–148.5 °C, from trituration with diisopropyl ether). Yield 85%. ^1^H NMR (600 MHz, *Chloroform-d*): δ 4.16 (*s*, 3H, -OC*H*_3_), 6.68 (*td,* 1H, J = 6.9, 1.2 Hz, *H-b*), 7.18 (*ddd*, 1H, J = 8.9, 6.8, 1.0 Hz, *H-c*), 7.38 (*dd*, 1H, J = 8.2, 4.2 Hz, *H-c*), 7.71 (*d*, 1H, J = 9.0 Hz, *H-d*), 8.04 (*d*, 1H, J = 1.8 Hz,), 8.08 (*dd*, 1H, J = 8.7, 1.9 Hz,), 8.11–8.18 (*m*, 2H), 8.30 (*d*, 1H, J = 6.9 Hz, *H-a*), 8.86 (*d,* 1H, J = 3.1 Hz). ^13^C NMR (151 MHz, *Chloroform-d*): δ 56.7 (−O*C*H_3_), 94.9 (*C-f*), 110.4 (*C-b*), 115.8, 121.4, 125.0, 125.4, 128.9, 129.0, 129.7, 129.9, 130.8, 135.8, 139.2, 146.8, 149.8, 163.2 (*C-g*). MS (ES^+^): 276 (M+1).

#### Ethyl 2-(benzyloxy)-7-chloropyrazolo­[1,5-a]­pyridine-3-carboxylate (**21**)

LiHMDS (1.0 M THF solution: 1.48 mL, 1.48 mmol) was added dropwise to a solution of **2c** (0.400 g, 1.35 mmol) in dry THF (10 mL), previously cooled to a −78 °C with dry ice and acetone. The mixture was stirred at −78 °C for 1 h, and then a solution of hexachloroethane (0.383 g, 1.62 mmol) in dry THF was added. The mixture was left slowly to back at room temperature and, subsequently, the reaction was quenched with saturated solution of NH_4_Cl (100 mL). The water phase was extracted with EtOAc (3 × 25 mL). The combined organic layers were dried over Na_2_SO_4_, filtered, and evaporated to dryness under vacuum. The crude product was purified by flash chromatography (eluent: *petroleum ether*/ethyl acetate 80:20 v/v) to afford the title compound as a white solid. Yield 81%. ^1^H NMR (600 MHz, *DMSO-d*_6_): δ 1.31 (*t*, 3H, J = 7.1 Hz, −OCH_2_C*H*_3_), 4.26 (*q*, 2H, J = 7.1 Hz, -OC*H*_2_CH_3_), 5.48 (*s*, 2H, −C*H*_2_Ph), 7.30–7.37 (*m*, 2H), 7.38–7.44 (*m*, 2H), 7.50–7.60 (*m*, 3H), 7.92 (*dd*, J = 8.8, 0.9 Hz, 1H, *H-d*). ^13^C NMR (151 MHz, *DMSO-d*_6_) δ 14.3 (−OCH_2_ *C*H_3_), 59.3 (−O*C*H_2_CH_3_), 70.4 (−*C*H_2_Ph), 88.8 (*C-f*), 113.5 (*C-b*), 115.8 (*C-d*), 127.6, 127.9 (*C-c*), 128.3, 129.2, 129.5 (*C-a*), 136.4, 143.7 (*C-e*), 161.6 (C-h), 163.9 (*C-g*). MS (ES^+^): 331 (M+1).

#### Ethyl 2-(Benzyloxy)-7-(dimethylamino)­pyrazolo­[1,5-a]­pyridine-3-carboxylate (**22**)

To a stirred solution of compound **21** (0.460, 1.39 mmol) in DMF (5.0 mL), a 30% aqueous solution of dimethylamine (0.35 mL) was added. The reaction mixture was stirred until complete conversion of starting material was observed by TLC. Then, the mixture was diluted with water (50 mL) and extracted with EtOAc (3 × 10 mL). The organic layers were collected, dried over anhydrous MgSO_4_, and filtered and concentrated under reduced pressure. The crude obtained was crystallized from methanol to afford the title compound as a white solid. Yield 75%. ^1^H NMR (400 MHz, *DMSO-d*_6_): δ 1.30 *(t*, 3H, J = 7.1 Hz, −OCH_2_C*H*_3_), 3.02 (*s,* 6H, −N­(C*H*_3_)_2_), 4.24 (*q*, 2H, J = 7.1 Hz, −OC*H*_2_CH_3_), 5.49 (*s*, 2H, −OC*H*_2_Ph), 6.43 (*dd*, 1H, J = 7.5, 1.6 Hz, *H-b*), 7.33 (t, 1H, J = 7.3 Hz), 7.37–7.55 (*m*, 6H).^13^C NMR (101 MHz, *DMSO-d*_6_): δ 14.4 (−OCH_2_ *C*H_3_), 40.8 (−N­(*C*H_3_)_2_), 58.8 (−O*C*H_2_CH_3_), 70.0 (−O*C*H_2_Ph), 86.3 (*C-f*), 99.1 (*C-b*), 108.3 (*C-d*), 127.3, 127.7, 128.3, 130.0, 136.9, 144.1 (*C-e*), 147.4 (*C-a*), 162.1 (*C-h*), 163.1 (*C-g*). MS (ES^+^): 340 (M+1).

#### Ethyl 7-(Dimethylamino)-2-hydroxypyrazolo­[1,5-a]­pyridine-3-carboxylate (**8a**)

Palladium on carbon (Pd/C, 8% w/w), was added to a solution of compound **22** (0.105 g, 0.40 mmol), in absolute EtOH (5.0 mL). The resulting mixture was vigorously stirred under a hydrogen atmosphere for 6 h. The suspension was filtered through a cake of Celite, that was washed with methanol. The overall filtrate was concentrated under reduced pressure to afford the crude compound. The latter was triturated with diisopropyl ether to afford the title compounds as a pale-yellow solid. Yield 93%.^1^H NMR (400 MHz, *DMSO-d*_6_): δ 1.29 (t, 3H, J = 7.1 Hz, −OCH_2_C*H*_3_), 3.00 (*s*, 6H, −N­(C*H*_3_)_2_), 4.24 (*q*, 2H, J = 7.1 Hz, −OC*H*_2_CH_3_), 6.41 (*dd*, 1H, J = 7.4, 1.5 Hz, *H-b*), 7.39–7.48 (*m*, 2H, *H-c and H-d*), 10.94 (*s*, 1H, −O*H*).^13^C NMR (101 MHz, *DMSO-d*_6_): δ 14.5 (−OCH_2_ *C*H_3_), 40.9 (−N­(*C*H_3_)_2_), 58.8 (−O*C*H_2_CH_3_), 85.8 (*C-f*), 99.3 (*C-b*), 108.3 (*C-d*), 129.3 (*C-c*), 143.5 (*C-e*), 147.2 (*C-a*), 163.0 (*C-g*), 163.5 (*C-h*). MS (ES^+^): 250 (M+1).

#### Ethyl 7-(Dimethylamino)-2-hydroxy-4-nitropyrazolo­[1,5-a]­pyridine-3-carboxylate (**8b**) and Ethyl 7-(Dimethylamino)-2-hydroxy-6-nitropyrazolo­[1,5-a]­pyridine-3-carboxylate (**8c**)

To a stirred solution of **8a** (0.050 g, 0.2 mmol) in acetic acid (2.0 mL) at 0 °C, a solution of NaNO_2_ (0.016 g, 0.24 mmol) in water (1.0 mL) was added dropwise. The reaction was then stirred at room temperature until complete conversion was observed by LC-MS. Then, the solvent was removed under reduced pressure. The crude was diluted with water (3.0 mL) and extracted with dichloromethane (3 × 2 mL). The organic layers were collected and concentrated under reduced pressure to obtain a dark brown oil. The residue was dissolved in water/acetonitrile (5 mL, 1:1 v/v) and purified by preparative HPLC (Method: 20 min, H_2_O 100% MeOH from 0 to 100%). Both fractions were freeze-dried providing two solids, which were triturated using diisopropyl ether to afford compound **8b** as a green solid and compound **8c** as a red solid.

**8b**) Yield 14%. ^1^H NMR (600 MHz, *DMSO-d*_6_): δ 1.30 (*t*, 3H, J = 6.8 Hz, −CH_2_C*H*_3_), 3.06 (*s*, 6H, −N­(C*H*_3_)_2_), 4.26 (*q*, 2H, J = 6.8 Hz, −OC*H*_2_CH_3_), 7.49 (*d*, 1H, J = 9.2 Hz, *H-b*), 7.98 (*d*, 1H, J = 9.5 Hz, *H-c*), 11.66 (*s*, 1H, −O*H*).^13^C NMR (101 MHz, *DMSO-d*_6_): δ 14.4 (−OCH_2_ *C*H_3_), 41.7 (−N­(*C*H_3_)_2_), 59.4 (−O*C*H_2_CH_3_), 89.7 (*C-f*), 107.9 (*C-b*), 125.9 (*C-d*), 128.0 (*C-c*), 143.8 (*C-e*), 145.0 (*C-a*), 162.1, 164.5 (*C-g*). MS (ES^+^): 295 (M+1).

**8c**) Yield 8%. ^1^H NMR (600 MHz, *DMSO-d*_6_): δ 1.30 (*t*, 3H, J = 7.1 Hz, −OCH_2_C*H*_3_), 3.32 (*s*, 6H, −N­(C*H*_3_)_2_), 4.16 (*q*, 2H, J = 7.1 Hz, −OC*H*_2_CH_3_), 6.34 (*d*, 1H, J = 9.2 Hz, *H-c*), 8.17 (*d*, 1H, J = 9.2 Hz, *H-d*), 11.62 (*s*, 1H, −O*H*). ^13^C NMR (101 MHz, *DMSO-d*_6_): δ 14.2 (−OCH_2_ *C*H_3_), 42.0 (−N­(*C*H_3_)_2_), 59.9 (−O*C*H_2_CH_3_), 91.1 (*C-f*), 95.5, 127.3, 128.9, 135.9, 149.9, 162.5, 162.6. MS (ES^+^): 295 (M+1).

#### Ethyl 7-Chloro-2-methoxypyrazolo­[1,5-a]­pyridine-3-carboxylate (**23**)

LiHMDS (1.0 M THF solution: 2.17 mL, 2.17 mmol) was added dropwise to a solution of **2a** (0.400 g, 1.82 mmol) in dry THF (10 mL), previously cooled to a −78 °C. The mixture was stirred at −78 °C for 1 h, and then a solution of hexachloroethane (0.572 g, 2.17 mmol) in dry THF was added. The mixture was left slowly to relieve at room temperature and, subsequently, the reaction was quenched with an aqueous saturated solution of NH_4_Cl (50 mL). The water phase was extracted with EtOAc (3 × 10 mL). The combined organic phases were dried over Na_2_SO_4_, filtered, and evaporated to dryness under vacuum. The crude product was purified by flash chromatography (eluent: *petroleum ether*/ethyl acetate 8:2 v/v) to afford the title compound as a white solid. Yield 81%. ^1^H NMR (600 MHz, *Chloroform-d*): δ 1.41 (*t*, 3H, J = 7.1 Hz, −CH_2_C*H*_3_), 4.20 (*s*, 3H, −OC*H*_3_), 4.39 (*q*, 2H, J = 7.1 Hz, −C*H*_2_CH_3_), 6.96 (*dd*, 1H, J = 7.5, 1.3 Hz, *H-b*), 7.30 (*dd*, 1H, J = 8.9, 7.5 Hz, *H-c*), 7.96 (*dd*, 1H, J = 8.9, 1.3 Hz, *H-d*). ^13^C NMR (151 MHz, *Chloroform-d*): δ 14.7 (−CH_2_ *C*H_3_), 57.3 (−O*C*H_3_), 60.1 (−*C*H_2_CH_3_), 89.8 (*C-f*), 112.7, 116.5, 127.9, 130.7, 144.6 (*C-e*), 163.1, 165.7 (*C-g*). MS (ES^+^): 255 (M+1).

#### 7-Chloro-2-methoxypyrazolo­[1,5-a]­pyridine (**24**)

Five M NaOH (10.0 equiv) was added to compound **23** (1.00 mmol) in EtOH (20 mL). The reaction mixture was stirred at reflux until complete conversion of the starting material was observed by TLC (eluent: *petroleum ether*/EtOAc 8:2 v/v). Then, EtOH was removed under reduced pressure. The white solid was taken up with water and warmed at reflux. Then 37% w/w HCl was slowly added until pH 1 was reached. Subsequently, the mixture was extracted with EtOAc (3 × 20 mL), dried under Na_2_SO_4_, and evaporated under reduced pressure to afford crude oil. The latter was purified by flash chromatography (eluent: *petroleum ether*/ethyl acetate 90:10 v/v) to afford the title compound as a white solid. Yield 51%. ^1^H NMR (400 MHz, *Chloroform-d*): δ 4.05 (*s*, 3H, −OC*H*_3_), 5.97 (*s*, 1H, *H-f*), 6.74 (*dd*, 1H, J = 7.3, 1.2 Hz, *H-b*), 7.01 (*dd*, 1H, J = 8.8, 7.3 Hz, *H-c*), 7.27 (*dd*, 1H, J = 8.8, 1.2 Hz, *H-d*). ^13^C NMR (400 MHz, *Chloroform-d*): δ 57.0 (−O*C*H_3_), 81.7 (*C-f*), 109.9 (*C-b*), 114.7 (*C-d*), 124.2 (*C-c*), 129.7 (*C-a*), 143.5 (*C-e*), 166.4 (*C-g*). MS (ES^+^): 183 (M+1).

#### 7-Chloro-3-iodo-2-methoxypyrazolo­[1,5-a]­pyridine (**25**)

A solution of compound **24** (0.113 g, 0.618 mmol) in dry DCM (5.0 mL) was cooled at 0 °C by an ice bath. Successively, NIS (0.121 mg, 0.680 mmol) was slowly dropped into the reaction mixture that was then stirred until complete conversion of starting material as observed by TLC (eluent: *petroleum ether*/EtOAc 9:1 v/v). Then, the solvent was removed under reduced pressure and the crude residue was purified by flash chromatography (eluent*: petroleum ether*/EtOAc 95/5 v/v) to afford the title compound as a white solid. Yield 90%. ^1^H NMR (600 MHz, *Chloroform-d*): δ 3.98 (*s*, 3H, −OC*H*_3_), 6.62 (*dd*, 1H, J = 7.3, 1.2 Hz, *H-b*), 6.94 (*dd*, 1H, J = 8.9, 7.3 Hz, *H-c*), 7.06 (*dd*, 1H, J = 8.9, 1.2 Hz, *H-d*). ^13^C NMR (151 MHz, *Chloroform-d*): δ 34.4 (C-*f*), 57.2 (−O*C*H_3_), 110.6 (*C-b*), 114.7 (*C-d*), 125.5 (*C-c*), 130.3 (*C-a*), 143.8 (*C-e*), 165.4 (*C-g*). MS (ES^+^): 309 (M+1).

#### 6-(7-Chloro-2-methoxypyrazolo­[1,5-a]­pyridin-3-yl)­quinoline (**9a**)

[Pd­(PPh_3_)_4_] (1.9 mg, 0.0016 mmol) was added to a solution of 7-chloro-3-iodo-2-methoxypyrazolo­[1,5-*a*]­pyridine (0.050 g, 0.162 mmol) and Cs_2_CO_3_ (0.154 mg, 0.486 mmol) in dioxane/water (8:2 v/v, 10 mL) solution. After stirring the resulting mixture under nitrogen atmosphere for 1 h, quinolin-6-ylboronic acid (28 mg, 0.16 mmol) was added and the reaction mixture was stirred at reflux, until the complete conversion of starting material was observed by TLC (eluent: *petroleum ether*/EtOAc 9:1 v/v). Then, the reaction was cooled to room temperature and was concentrated under reduced pressure to afford a crude material. The latter was *taken-up* with water (30 mL), and the aqueous solution was extracted with EtOAc (3 × 5 mL). The combined organic layers were dried over Na_2_SO_4_ and concentrated under reduced pressure. The crude material was purified by flash chromatography (eluent*: petroleum ether*/EtOAc from 90/10 to 70/30 v/v) to afford the title compound as a pale-yellow solid. Yield 80%. ^1^H NMR (600 MHz, *Chloroform-d*): δ 4.23 (*s*, 3H, −OC*H*_3_), 6.84 (*d*, 1H, J = 7.2, *H-b*), 7.15 (*t*, 1H, J = 8.0 Hz, *H-c*), 7.42 (*dd*, 1H, J = 8.1, 4.1 Hz), 7.67 (*d*, 1H, J = 8.8 Hz), 7.97 – 8.12 (*m*, 2H), 8.20 (*t*, 2H, J = 7.6 Hz), 8.68 – 9.06 (*m*, 1H). ^13^C NMR (151 MHz, *Chloroform-d*): δ 57.0 (−O*CH*_3_), 96.7 (C-*f*), 110.6 (C-*b*), 114.0 (C-*d*), 121.5, 125.4, 125.5, 128.9, 129.4, 130.3, 130.5, 130.6, 136.4, 140.8, 146.5 (C-*e*), 149.6, 163.2 (C-*g*). MS (ES^+^): 310 (M+1).

#### 2-Methoxy-*N*,*N*-dimethyl-3-(quinolin-6-yl)­pyrazolo­[1,5-a]­pyridin-7-amine (**9b**)

To a stirred solution of compound **9a** (0.020 g, 0.064 mmol), in DMF (1.0 mL), a 30% aqueous solution of dimethylamine (1.0 mL) was added. The reaction was stirred until complete conversion of starting material was observed by TLC (eluent: *petroleum ether*/EtOAc 5:5 v/v). Then, the mixture was concentrated under reduced pressure. The crude obtained was purified by flash chromatography (eluent*: petroleum ether*/EtOAc from 50/50 to 20/80 v/v) to afford the title compound as a pale orange solid. Yield 75%. ^1^H NMR (600 MHz, *Chloroform-d*): δ 3.13 (*s*, 6H, −N­(C*H*_3_)_2_), 4.23 (*s*, 3H, −O*CH*_3_), 6.15 (*d*, 1H, J = 7.4, 1H, *H-b*), 7.20 (*ddd*, 1H, J = 9.0, 7.4, 1.6 Hz, *H-c*), 7.39 (*d*, 1H, J = 8.8 Hz, *H-d*), 7.41–7.46 (*m*, 1H), 8.08 (*s*, 1H), 8.15 (*dt*, 1H, J = 8.8, 2.0 Hz), 8.18–8.26 (*m*, 2H), 8.88 (*s*, 1H). ^13^C NMR (151 MHz, *Chloroform-d*): δ 41.4 (−N­(*C*H_3_)_2_), 56.7 (−O*C*H_3_), 94.2 (C-*f*), 96.8 (C-*b*), 108.0 (C-*d*), 121.3, 124.9, 126.8, 128.5, 129.1, 130.8, 131.8, 136.9, 141.3, 145.6, 148.1, 148.6, 162.6 (C-*g*). MS (ES^+^): 319 (M+1).

#### 2-((4-Methoxybenzyl)­oxy)­pyrazolo­[1,5-a]­pyridine (**26**)

4-Methoxybenzyl chloride (0.840 g, 5.36 mmol) was added to a mixture of pyrazolo­[1,5-*a*]­pyridin-2-ol (0.600 g, 4.47 mmol) and Cs_2_CO_3_ (4.370 g, 13.41 mmol) in dry DMF (15 mL). The reaction mixture was stirred overnight at room temperature, and then water (100 mL) was added. The mixture was extracted with EtOAc (4 × 70 mL), and the combined organic layers were dried over Na_2_SO_4_ and evaporated under reduced pressure to afford a crude yellow oil. The crude compound was purified by flash chromatography (eluent: *petroleum ether*/EtOAc 2/1 v/v) to afford the title compound as a pale pink solid (mp 110.2–111.3 °C, trituration from diisopropyl ether). Yield 61%. ^1^H NMR (600 MHz, *Chloroform-d*): δ 3.81 (*s*, 3H, −OC*H*_3_), 5.26 (*s*, 2H, −OC*H*_2_Ar), 5.85 (*s*, 1H, *H-f*), 6.59 (*td*, 1H, J = 6.9, 0.9 Hz, *H-b*), 6.92 (*d*, 2H, J = 8.7 Hz), 7.02–7.07 (*m*, 1H, *H-c*), 7.29 (*d*, 1H, J = 8.9 Hz, *H-d*), 7.43 (*d*, 2H, J = 8.7 Hz), 8.24 (*dd*, 1H, J = 6.9, 0.9 Hz, *H-a*). ^13^C NMR (151 MHz, *Chloroform-d*): δ 55.4 (−O*C*H_3_), 70.9 (−O*C*H_2_Ar), 80.4 (*C-f*), 109.8 (*C-b*), 114.0, 116.5 (*C-d*), 124.0, 128.5, 129.1, 129.8, 141.8 (*C-e*), 159.6, 165.6 (*C-g*). MS (ES^+^): 255 (M+1).

#### 2-((4-Methoxybenzyl)­oxy)­pyrazolo­[1,5-a]­pyridine-3-carbaldehyde (**27**)

A solution of POCl_3_ (1.08 g, 7.07 mmol) in dry DMF (20.0 mL), was stirred for 30 min at room temperature under a nitrogen atmosphere. After cooling the mixture to 0 °C, a solution of 2-((4-methoxybenzyl)­oxy)­pyrazolo­[1,5-*a*]­pyridine (0.600 g, 2.35 mmol) in dry DMF (5.0 mL) was added dropwise. The reaction mixture was stirred until complete disappearance of the starting material was observed by TLC. The reaction mixture was quenched with 1 M NaOH (200 mL) and the observed precipitate was filtered off. The solid was triturated with diisopropyl ether to afford a white fluffy solid (mp 149.0 – 151.0 °C, trituration from diisopropyl ether). Yield 90%. ^1^H NMR (600 MHz, *Chloroform-d*): δ 3.82 (*s*, 3H, -OC*H*_3_), 5.40 (*s*, 2H, -OC*H*_2_Ar), 6.90–6.97 (*m*, 3H), 7.43–7.48 (*m*, 3H), 8.14 (*d*, 1H, J = 8.7 Hz, *H-d*), 8.33 (*d*, 1H, J = 6.8 Hz, *H-a*), 9.92 (*s*, 1H, −C*H*O). ^13^C NMR (151 MHz, *Chloroform-d*): δ 55.4 (−O*C*H_3_), 71.2 (−O*C*H_2_Ar), 99.0 (*C-f*), 114.1, 114.1, 118.5, 128.2, 129.1, 129.8, 130.2, 141.4 (*C-e*), 159.9, 167.2 (*C-g*), 182.4 (*-C*HO). MS (ES^+^): 283 (M+1).

### General Wittig Reaction Procedure for Synthesis of Compounds **28 a–B**

A suspension of the correspondent phosphonium salt (1.14 mmol) in dry THF (7.0 mL), was cooled at −10 °C under a nitrogen atmosphere, then, a 1 M solution of LiHMDS in THF (1.14 mmol, 1.14 mL) was added dropwise. The reaction mixture was stirred for 30 min at −10 °C and then, a solution of *2-((4-methoxybenzyl)­oxy)­pyrazolo­[1,5-a]­pyridine-3-carbaldehyde* (1.14 mmol), in dry THF (1.0 mL) was slowly added. The reaction was stirred at reflux until complete conversion of the starting material was observed by TLC. The mixture was quenched in a saturated solution of NH_4_Cl (100 mL) and extracted with EtOAc (3 × 50 mL). The combined organic layers were dried over Na_2_SO_4_ and evaporated under reduced pressure to afford a dark oil. The crude product was purified by flash chromatography (see below the conditions).

#### (E)-2-((4-Methoxybenzyl)­oxy)-3-(2-(2,3,5,6-tetrafluoro-[1,1’-biphenyl]-4-yl)­vinyl)­pyrazolo­[1,5-a]­pyridine (**28a**)

The crude mixture was purified by flash chromatography (eluent*: petroleum ether*/acetone from 100 to 95/5 v/v) to afford a mixture Z/E isomer (10:90) as yellow solid. The mixture Z/E was solubilized in CHCl_3_ and cooled hexane was added. The yellow precipitate was filtered off, affording the desired E product as yellow solid. Yield 55%. ^1^H NMR (600 MHz, *Chloroform-d*): δ 3.84 (*s*, 3H, −OC*H*_3_), 5.46 (*s*, 2H, −OC*H*_2_Ar), 6.70 (*td*, 1H, J = 6.9, 1.3 Hz, *H-b*), 6.96 (*d*, 2H, J = 8.7 Hz), 7.14–7.24 (*m*, 2H), 7.39–7.47 (*m*, 1H), 7.47–7.56 (*m*, 7H), 7.59 (*d*, J = 16.7 Hz, 1H), 8.25 (*d*, 1H, J = 6.9 Hz, *H-a*). ^13^C NMR (151 MHz, *Chloroform-d*): δ 55.5 (−O*C*H_3_), 70.9 (−O*C*H_2_Ar), 94.5 (*C-f*), 110.0, 111.0, 114.1, 115.6, 116.7 (*t*, J = 17.2 Hz), 117.6 (*t*, J = 13.5 Hz), 125.57, 125.63, 125.7, 128.1, 128.6, 128.9, 129.0, 129.4, 130.4, 139.6 (*C-e*), 144.1 (*dd*, J = 241.3, 16.1 Hz), 144.5 (*dd*, J = 246.7, 12.7 Hz), 159.6, 163.6 (*C-g*). ^19^F NMR (565 MHz, *Chloroform-d*): δ −146.18 (*dd*, J = 21.6, 10.5 Hz), −144.81 (*dd*, J = 21.6, 10.4 Hz). MS (ES^+^): 527 (M+Na).

#### (E)-2-((4-Methoxybenzyl)­oxy)-3-(2-(2,3,6-trifluoro-[1,1’-biphenyl]-4-yl)­vinyl)­pyrazolo­[1,5-a]­pyridine (**28b**)

The crude mixture was purified by flash chromatography (eluent*: petroleum ether*/acetone from 100 to 95/5 v/v) to afford a crude compound still slightly impure of the Z isomer that was removed by trituration with diisopropyl ether to afford the title compound as a yellow solid. Yield 22%. ^1^H NMR (600 MHz, *Chloroform-d*): δ 3.84 (*s*, 3H, −OC*H*_3_), 5.45 (*s*, 2H, −OC*H*_2_Ar), 6.70 (*t*, 1H, J = 6.8 Hz, *C-b*), 6.97 (*d*, 2H, J = 8.5 Hz), 7.13–7.16 (*m*, 1H), 7.18–7.29 (*m*, 3H), 7.41 (*t*, 1H, J = 6.9 Hz), 7.43–7.56 (*m*, 6H), 7.56 (*d*, 1H, J = 8.8 Hz, *H-d*), 8.26 (*d*, 1H, J = 6.8 Hz, *H-a*). ^13^C NMR (151 MHz, *Chloroform-d*): δ 55.5 (−O*C*H_3_), 71.0 (−O*C*H_2_Ar), 94.0 (*C-f*), 106.5 (*d*, J = 26.0 Hz), 110.9, 114.1, 114.6 (*d*, J = 2.2 Hz), 115.8, 116.9 (*dd*, J = 21.1, 15.2 Hz), 121.4 (*d*, J = 4.3 Hz), 125.7, 127.9 (*dd*, J = 12.8, 8.0 Hz), 128.50, 128.52, 128.9, 129.0, 129.7, 130.4, 139.4, 144.9 (*d*, J = 259.9 Hz), 146.6 (*d*, J = 246.6 Hz), 155.2 (*d*, J = 242.7 Hz), 159.7, 163.6. ^19^F NMR (565 MHz, *Chloroform-d*): δ −148.56 (*t*, J = 17.3 Hz), −139.32 (*d*, J = 19.8 Hz), −121.18 (*t*, J = 13.0 Hz). MS (ES^+^): 487 (M+1).

### General Procedure Followed for Synthesis of Target Compounds **29a** and **29b**

A solution of *O-protected* hydroxy pyrazolo­[1,5-*a*]­pyridine (4.70 mmol) in dry DCM (10 mL) was cooled at 0 °C by an ice bath. Sub sequentially, NIS (5.17 mmol) was slowly dropped into the reaction. The reaction mixture was stirred until complete conversion of starting material was observed by TLC (eluent: *petroleum ether*/EtOAc 9:1 v/v). The solvent was removed under reduced pressure and the crude mixture was purified by flash chromatography (see below the conditions).

#### 2-Benzyloxy-3-iodopyrazolo­[1,5-a]­pyridine (**29a**)

The mixture was purified by flash chromatography (eluent*: petroleum ether*/EtOAc 95/5 v/v) to afford a crude compound. The latter was triturated with diisopropyl ether, to afford the title compound as a white solid. Yield 93%.^1^H NMR (600 MHz, *Chloroform-d*): δ 5.45 (*s*, 2H, −C*H*_2_OAr), 6.64 (*td*, 1H, J = 6.9, 1.4 Hz, *H-b*), 7.14 (*ddd*, 1H, J = 8.9, 6.8, 1.1 Hz, *H-c*), 7.22–7.27 (*m*, 1H,), 7.33 (*t*, 1H, J = 7.4 Hz), 7.40 (*t,* 2H, J = 7.5 Hz), 7.52 (*d*, 2H, J = 7.0 Hz), 8.21 (*dt*, 1H, J = 6.9, 1.0 Hz, *H-a*). ^13^C NMR (151 MHz, *Chloroform-d*): δ 32.7 (*C-f*), 71.2 (−*C*H_2_OAr), 110.7 (*C-b*), 116.4 (*C-d*), 125.5, 127.8, 128.1, 128.6, 129.2, 136.9, 142.5 (*C-e*), 164.6 (*C-g*). MS (ES^+^): 351 (M+1).

#### 3-Iodo-2-methoxypyrazolo­[1,5-a]­pyridine (**29b**)

The mixture was purified by flash chromatography (eluent*: petroleum ether*/EtOAc 90/10 v/v) to afford a crude compound. The latter was triturated with diisopropyl ether, to afford the title compound as a white solid (mp 50.3–51.9 °C, from trituration with diisopropyl ether). Yield 90%. ^1^H NMR (400 MHz, *Chloroform-d*): δ 4.12 (*s*, 3H, −C*H*_3_), 6.66 (*td*, 1H, J = 6.9, 1.4 Hz, *H-b*), 7.17 (*ddd*, 1H, J = 8.9, 6.8, 1.0 Hz, *H-c*), 7.27 (*dt*, 1H, J = 8.9, 1.1 Hz, *H-d*), 8.24 (*d*, 1H, J = 6.9 Hz, *H-d*). ^13^C NMR (400 MHz, *Chloroform-d*): δ 32.0 (*C-f*), 57.0 (*−C*H_3_), 110.6 (*C-b*), 116.4 (*C-d*), 125.5 (*C-c*), 129.2 (*C-a*), 142.5 (*C-e*), 165.3 (-O*C*H_3_). MS (ES^+^): 275 (M+1).

### General Procedure for the Synthesis of Compounds **30a** and **30b**

A solution of 3-Iodo-pyrazolo­[1,5-*a*]­pyridine (1.09 mmol) and 2-isopropoxy-4,4–5,5-tetramethyl-1,3,2-dioxaborolane (0.33 mL, 1.6 mmol) in dry THF (5 mL) was cooled down at −10 °C with an ice salt bath and stirred under nitrogen atmosphere. Isopropyl magnesium chloride, lithium chloride complex solution 1.3 M in THF (2.18 mmol) was added dropwise to the stirred solution over a period of 10 min. Upon completion, the reaction was quenched in a saturated solution of NH_4_Cl, and the water phase was extracted with EtOAc (3 × 25 mL). The combined organic layers were dried over Na_2_SO_4_ and concentrated under reduced pressure. The crude mixture was purified by flash chromatography (see below the conditions).

#### 2-Benzyloxy-3-(4,4,5,5-tetramethyl-1,3,2-dioxaborolan-2-yl)­pyrazolo­[1,5-a]­pyridine (**30a**)

The mixture was purified by Combiflash using RediSep Gold Silica Gel disposable flash column, 24 g of silica (eluent: Heptane/EtOAc from 100 to 85/15 v/v) to affords the title compound as a white solid. This latter was immediately used in the next step. MS (ES^+^): 351 (M+1).

#### 2-Methoxy-3-(4,4,5,5-tetramethyl-1,3,2-dioxaborolan-2-yl)­pyrazolo­[1,5-a]­pyridine (**30b**)

The mixture was purified by Combiflash using RediSep Gold Silica Gel disposable flash column, 24 g of silica (eluent: Heptane/EtOAc from 100 to 80/20 v/v) to affords the title compound as a white solid. This latter was immediately used in the next step. MS (ES^+^): 275 (M+1).

#### 2-(2-Methoxypyrazolo­[1,5-a]­pyridin-3-yl)-6-phenylquinoline (**31**)

[Pd­(PPh_3_)_4_] (5.4 mg, 0.0047 mmol) was added to a solution of 2-chloro-6-phenylquinoline (0.112 g, 0.47 mmol) and Cs_2_CO_3_ (0.459 g, 1.41 mmol,) in dioxane/water (8:2 v/v, 10 mL) solution. After stirring the resulting mixture under nitrogen atmosphere for 1 h, *2-methoxy-3-(4,4,5,5-tetramethyl-1,3,2-dioxaborolan-2-yl)­pyrazolo­[1,5-a]­pyridine* (0.130 g, 0.47 mmol) was added. The reaction mixture was refluxed for 2 h, then was cooled to room temperature and concentrated under reduced pressure. The crude material was *taken-up* with water (100 mL) and the mixture was extracted with EtOAc (3 × 25 mL). The combined organic layers were dried over Na_2_SO_4_ and concentrated under reduced pressure. The crude mixture was purified by Combiflash using RediSep Gold Silica Gel disposable flash column 24 g of silica (eluent:Heptane/EtOAc from 100 to 80/20 v/v)) to afford the title compound as a white solid (mp 182.6–183.3 °C, from trituration with diisopropyl ether). Yield 82%. ^1^H NMR (600 MHz, *Chloroform-d*): δ 4.26 (*s*, 3H, −OC*H*_3_), 6.82 (*t*, 1H, J = 6.8 Hz, *H-b*), 7.35–7.44 (*m*, 2H), 7.52 (*t*, 2H, J = 7.6 Hz), 7.77 (*d*, 2H, J = 7.5 Hz), 7.95–7.97 (*m*, 2H), 8.15–8.16 (*m*, 2H), 8.21 (*d*, 1H, J = 8.7 Hz, *H-d*), 8.34 (*d*, 1H, J = 6.7 Hz, *H-a*), 8.94 (*d*, 1H, J = 8.9 Hz). ^13^C NMR (151 MHz, *Chloroform-d*): δ 56.8 (−O*C*H_3_), 95.6 (*C-f*), 111.8 (*C-b*), 120.0, 120.6 (*C-d*), 125.3, 126.1 (*C-c*), 126.4, 127.42, 127.47, 128.49 (*C-a*), 128.8, 129.0, 129.2, 135.9, 137.6 (*C-e*), 140.9, 141.1, 147.9, 153.7, 164.4 (*C-g*). MS (ES^+^): 352 (M+1).

#### 7-(2-Benzyloxy-pyrazolo­[1,5-a]­pyridin-3-yl)-3-chloroquinoline (**32**)

[Pd­(PPh_3_)_4_] (4.6 mg, 0.004 mmol) was added to a solution of 7-bromo-3-chloroquinoline (0.104 g, 0.43 mmol) and Cs_2_CO_3_ (0.420 g, 1.29 mmol,) in dioxane/water (8:2 v/v 10 mL) solution. After stirring the resulting mixture under a nitrogen atmosphere for 1 h, *2-benzyloxy-3-(4,4,5,5-tetramethyl-1,3,2-dioxaborolan-2-yl)­pyrazolo­[1,5-a]­pyridine* (0.150 g, 0.43 mmol) was added. The reaction mixture was warm at reflux for 2 h, then was cooled to room temperature and concentrated under reduced pressure. The crude material was *taken-up* with water (50 mL) and the mixture was extracted with EtOAc (3 × 10 mL). The combined organic layers were dried over Na_2_SO_4_ and concentrated under reduced pressure. The crude was purified by Combiflash using RediSep Gold Silica Gel disposable flash column, 13 g (eluent*: petroleum ether*/EtOAc from 100 to 80/20 v/v) to afford the title compound as a white solid (mp 160.9–161.5 °C, from trituration with diisopropyl ether). Yield 58%.^1^H NMR (600 MHz, *Chloroform-d*): δ 5.55 (*s*, 2H, −C*H*_2_OAr), 6.72 (*td*, 1H, J = 6.8, 1.3 Hz, *H-b*), 7.21 (*ddd*, 1H, J = 9.0, 6.8, 1.1 Hz, *H-c*), 7.30–7.35 (*m*, 1H), 7.39 (*t*, 2H, J = 7.5 Hz), 7.52–7.55 (*m*, 2H), 7.76 (*d*, 1H, J = 8.5 Hz), 7.82 (*dt*, 1H, J = 9.0, 1.1 Hz, *H-d*), 8.06 (*dd*, 1H, J = 8.5, 1.7 Hz), 8.09 (*d*, 1H, J = 2.2 Hz), 8.32 (*dt*, 1H, J = 6.9, 1.1 Hz, *H-a*), 8.33–8.35 (*m*, 1H), 8.80 (*d*, 1H, J = 2.4 Hz). ^13^C NMR (151 MHz, *Chloroform-d*): δ 71.1 (−*C*H_2_OAr), 94.9 (*C-f*), 110.8 (*C-b*), 116.1 (*C-d*), 125.7, 125.8, 126.5, 127.2, 127.6, 127.9, 128.1, 128.3, 128.7, 129.1, 133.8, 134.4, 137.1, 139.3, 146.9, 149.9, 162.6 (*C-g*). MS (ES^+^): 386 (M+1).

### General Procedure for the Synthesis of Compounds **33** and **34**

[Pd­(OAc)_2_] (4.6 mg, 0.021 mmol) was added to a solution of 7-(2-benzyloxy-pyrazolo­[1,5-*a*]­pyridin-3-yl)-3-chloroquinoline (0.080 g, 0.21 mmol), X-PHOS (11 mg, 0.042 mmol) and K_3_PO_4_ (0.133 g, 0.63 mmol) in dioxane/water (8:2 v/v 5 mL) solution. After stirring the resulting mixture under a nitrogen atmosphere for 1 h, the correspondent boronic acid (0.42 mmol) was added and the reaction mixture was stirred at reflux until complete conversion of starting material was observed by TLC. Then, the reaction mixture was cooled to room temperature and concentrated under reduced pressure. The crude material was *taken up* with water (50 mL) and the aqueous layer was extracted with EtOAc (3 × 20 mL). Then the combined organic layers were dried over Na_2_SO_4_ and concentrated under reduced pressure. The crude material was purified by flash chromatography (see below the conditions).

#### 7-(2-Benzyloxy-pyrazolo­[1,5-a]­pyridin-3-yl)-3-phenylquinoline (**33**)

The crude was purified by Combiflash using RediSep Gold Silica Gel disposable flash column, 13 g (eluent*: petroleum ether*/EtOAc from 100 to 80/20 v/v) to afford the title compound as a white solid. Yield 96%. ^1^H NMR (600 MHz, *Chloroform-d*): δ 5.56 (*s*, 2H, C*H*_2_OAr), 6.72 (*td*, 1H, J = 6.9, 1.3 Hz, *H-b*), 7.21 (*ddd*, 1H, J = 9.0, 6.8, 1.1 Hz, *H-c*), 7.31–7.35 (*m*, 1H), 7.40 (*t*, 2H, J = 7.5), 7.42–7.45 (*m*, 1H), 7.52–7.56 (*m*, 4H), 7.74 (*dd*, 2H, J = 8.3, 1.2 Hz), 7.87 (*dd*, 1H, J = 9.0, 1.0 Hz), 7.90 (*d*, 1H, J = 8.8 Hz), 8.07 (*dd*, 1H, J = 8.5, 1.7 Hz), 8.28 (*d*, 1H, J = 2.1 Hz), 8.33 (*dd*, 1H, J = 6.9, 1.0 Hz, *H-a*), 8.39–8.40 (*m*, 1H), 9.17 (*d*, 1H, J = 2.3 Hz). ^13^C NMR (151 MHz, *Chloroform-d*): δ 71.1 (*C*H_2_OAr), 95.2 (*C-f*), 110.7 (*C-b*), 116.3 (*C-d*), 125.5, 125.8, 126.2, 127.5, 127.7, 127.8, 128.07, 128.08, 128.2, 128.6, 129.0, 129.3, 133.0, 133.2, 134.1, 137.2, 138.2, 139.3 (*C-e*), 148.0, 150.2, 162.6 (*C-g*). MS (ES^+^): 428 (M+1).

#### 7-(2-Benzyloxy-pyrazolo­[1,5-a]­pyridin-3-yl)-3-(2,6-difluorophenyl)­quinoline (**34**)

The crude mixture was purified by Combiflash using RediSep Gold Silica Gel disposable flash column, 12 g of silica (eluent*: petroleum ether*/EtOAc from 100 to 85/15 v/v) to affords the title compound as a pale-yellow solid. Yield 90%. ^1^H NMR (600 MHz, *Chloroform-d*): δ 5.56 (*s*, 2H, −C*H*_2_OAr), 6.71 (*td*, 1H, J = 6.8, 1.3 Hz, *H-b*), 7.02–7.11 (*m*, 2H), 7.21 (*ddd,* 1H, J = 9.0, 6.8, 1.1 Hz, *H-c*), 7.30–7.37 (*m*, 2H), 7.40 (*t*, 2H, J = 7.5 Hz), 7.55 (*d*, 2H, J = 6.9 Hz), 7.84–7.89 (*m*, 2H), 8.08 (*dd*, 1H, J = 8.5, 1.7 Hz), 8.25 (*s*, 1H), 8.32 (*dt*, 1H, J = 6.9, 1.0 Hz, *H-a*), 8.40–8.42 (*m*, 1H), 8.98 (*d*, 1H, J = 1.5, 1.0 Hz). ^13^C NMR (151 MHz, *Chloroform-d*): δ 71.1 (−*C*H_2_OAr), 95.2 (*C-f*), 110.8 (*C-b*), 112.03 (*d*, J = 21.1 Hz), 112.06 (*d*, J = 21.0 Hz), 115.5 (*t*, J = 18.8 Hz), 116.2, 121.9, 125.62, 125.69, 125.7, 127.6, 127.8, 128.1, 128.3, 128.6, 129.0, 129.8 (*t,* J = 10.2 Hz), 134.8, 137.1, 137.2, 139.3 (*C-e*), 148.0, 151.5, 160.4 (*d*, J = 249.3 Hz), 160.5 (*d*, J = 250.0 Hz), 162.6 (*C-g*).^19^F NMR (565 MHz, *Chloroform-d*) δ: −114.19. MS (ES^+^): 464 (M+1).

### General Procedure for the Synthesis of Compounds **11a, 11b, 13** and **14**

TFA (3.0 mL) was added to a 0 °C cooled suspension of the corresponding starting material (0.37 mmol) in thioanisole (436 μL, 3.70 mmol). The reaction mixture was stirred at room temperature, until a complete conversion of starting material was observed by TLC. The mixture was concentrated, and the resulting solid was purified following a different procedure (see details above).

#### (E)-3-(2-(2,3,5,6-Tetrafluoro-[1,1’-biphenyl]-4-yl)­vinyl)­pyrazolo­[1,5-a]­pyridin-2-ol (**11a**)

The result sticky solid was triturated with EtOH to afford the title compound as a yellow solid. Yield: quantitative. ^1^H NMR (600 MHz, *DMSO-d*_6_): 6.85 (*td*, 1H, J = 6.9, 1.3 Hz, *H-b*), 7.03 (*d*, 1H, J = 16.6 Hz), 7.34 (*ddd*, 1H, J = 8.7, 6.9, 1.0 Hz, *H-c*), 7.47 7.55 (*m*, 1H), 7.55 (*d*, 2H, J = 5.7 Hz), 7.59 (*d*, 1H, J = 16.6 Hz), 7.74 (*dt*, 1H, J = 8.8, 1.2 Hz, *H-d*), 8.48 (*dt*, 1H, J = 6.9, 1.1 Hz, *H-a*), 11.71 (*br*, 1H, −O*H*). ^13^C NMR (151 MHz, *DMSO-d*_6_) δ 92.7, 107.2, 111.4, 115.0, 115.9 (*t*, J = 17.3 Hz), 117.3 (*t*, J = 13.7 Hz), 126.1, 126.9 (*t*, J = 8.68 Hz), 127.05, 128.7, 128.9, 129.2, 130.1, 138.8, 142.7, 144.3, 162.9 (*C-g*).^19^F NMR (565 MHz, *DMSO-d*_6_): δ −145.79 (*d*, J = 13 Hz), −145.08 (*d*, J = 12.5 Hz). MS (ES^+^): 385 (M + 1). ESI-HRMS (*m*/*z*): [M + H]^+^calcd. for C_21_H_13_F_4_N_2_O 385.0959; obsd. 385.0959.

#### E)-3-(2-(2,3,6-Trifluoro-[1,1’-biphenyl]-4-yl)­vinyl)­pyrazolo­[1,5-a]­pyridin-2*-*ol (**11b**)

The resulting oil crude was purified by flash chromatography (eluent: DCM/MeOH from 100 to 90/10 v/v) to afford a title compound as a yellow solid. Yield: 95%. ^1^H NMR (600 MHz, *DMF-d*_7_): δ 6.86 (*t*, J = 6.7 Hz, 1H, *H-b*), 7.32–7.38 (*m*, 2H), 7.48–7.53 (*m*, 1H), 7.55–7.60 (*m*, 4H), 7.75–7.86 (*m*, 2H), 7.97 (*d*, J = 8.8 Hz, 1H, *H-d*), 8.48 (d, J = 6.7 Hz, 1H, *H-a*). ^13^C NMR (151 MHz, *DMF-d*_7_): δ 93.4 (C-*f*), 106.7 (*d*, J = 26.6 Hz), 111.4 (C-*b*), 112.5, 115.7 (C-*d*), 116.5 (*dd*, J = 21.7, 15.7 Hz), 123.6 (*d*, J = 2.06 Hz), 125.6, 125.8 (*C-c*), 128.5, 128.9, 128.96, 128.99, 129.1, 129.3, 130.6, 140.0, 155.3 (*d*, J = 255.51 Hz), 155.5 (*d*, J = 252.52 Hz), 163.6 (C-*g*). ^19^F NMR (565 MHz, *DMF-d*_7_): δ −149.88 (*t*, J = 17.5 Hz), −139.83 (*d*, J = 21.6 Hz), −120.76 (*t*, J = 12.5 Hz). MS (ES^+^): 367 (M + 1). ESI-HRMS (*m*/*z*): [M + H]^+^calcd for C_21_H_14_F_3_N_2_O 367.1053; obsd. 367.1053.

#### 3-(3-Phenylquinolin-7-yl)­pyrazolo­[1,5-a]­pyridin-2-ol (**13**)

The crude material was purified by Combiflash using RediSep Gold Silica Gel disposable flash column, 4 g of silica (eluent: DCM/MeOH from 100 to 85/15 v/v) to achieve a crude compound. This latter was solubilized in MeOH and precipitated with water to afford the title compound as an orange solid (mp 281.1–283 °C, dec from water). Yield 75%.^1^H NMR (600 MHz, *DMSO-d*_6_): δ 6.80 (*t*, 1H, J = 6.4 Hz, *H-b*), 7.29 (*t*, 1H, J = 7.8 Hz), 7.41 (*t,* 1H, J = 7.0 Hz), 7.52 (*t*, 2H, J = 7.3 Hz) 7.79–7.89 (*m*, 3H), 8.06 (*m*, 2H), 8.27 (*s*, 1H), 8.49 (*d*, 1H, J = 6.4 Hz, *H-a*), 8.56 (*s*, 1H), 9.19 (*s*, 1H), 11.39 (*br*, 1H, −O*H*). ^13^C NMR (600 MHz, *DMSO-d*_6_): δ 93.1 (*C-f*), 110.9 (*C-b*), 115.3 (*C-d*), 124.4, 125.4, 125.8, 126.7, 127.0, 128.0, 128.4, 129.0, 129.2, 131.8, 132.4, 134.2, 137.3, 138.1, 147.5, 149.6, 161.8 (*C-g*). MS (ES^+^): 338 (M+1). ESI-HRMS (*m*/*z*): [M + H]^+^calcd for C_22_H_16_N_3_O 338.1288; obsd. 338.1287.

#### 3-(3-(2,6-Difluorophenyl)­quinolin-7-yl)­pyrazolo­[1,5-a]­pyridin-2-ol (**14**)

The crude compound was purified using a Dowex 50W-X8 (200–400 mesh, capacity 1.7 mequiv/mL wet bed volume) ion-exchange resin, affording the title compound as an orange solid. The resin activation was performed according to the following method. The resin was washed with water (three volumes of resin), 10% w/w HCl (up to acidic pH), water (up to neutral pH), 10% w/w aqueous ammonia (up to basic pH), water (up to neutral pH), and then 10% w/w HCl until acidic pH was reached. The resin was then washed with water until the neutrality of the eluate, and then a solution of the TFA salt, dissolved in slightly acidic water to help solubility, was loaded on the top of the column. The column was eluted with water until neutral pH and then with 10% w/w aqueous ammonia solution to recover the desired compound in a neutral form (mp 295.0–296.6 °C, from water). Yield 20%. ^1^H NMR (600 MHz, *DMSO-d*_6_): δ 6.85 (*td*, 1H, J = 6.8, 1.3 Hz, *H-b*), 7.27–7.39 (*m*, 3H), 7.53–7.61 (*m*, 1H), 7.89 (*d*, 1H, J = 8.09 Hz, *H-d*), 8.04–8.15 (*m*, 2H), 8.32 (*s*, 1H), 8.49 (*s*, 1H), 8.54 (*d*, 1H, J = 6.9 Hz, *H-a*), 8.92 (*s*, 1H), 11.44 (*br*, 1H, -O*H*). ^13^C NMR (151 MHz, *DMSO-d*_6_): δ 92.9 (*C-f*), 111.0, 112.27 (*d*, J = 21.0 Hz). 112.30 (*d*, J = 21.2 Hz), 114.7 (*t*, J = 18.9 Hz), 115.3, 121.0, 124.3, 124.8, 126.0, 126.7, 128.4, 129.0, 130.8, 135.1, 136.9, 138.1, 147.5, 151.1, 159.61 (*d*, J = 246.3 Hz), 159.65 (*d*, J = 246.4 Hz), 161.9 (*C-g*). ^19^F NMR (565 MHz, *DMSO-d*_6_): −114.79. MS (ES^+^): 374 (M+1). ESI-HRMS (*m*/*z*): [M + H]^+^calcd for C_22_H_14_F_2_N_3_O 374.1099; obsd. 374.1100.

#### 3-(6-Phenylquinolin-2-yl)­pyrazolo­[1,5-a]­pyridin-2-ol (**12**)

2-(2-Methoxypyrazolo­[1,5-*a*]­pyridin-3-yl)-quinoline (0.01 mmol) was suspended in 1 mL of 48% w/w HBr aqueous solution. The suspension was warm at reflux until complete conversion of starting material was observed by TLC. The resulting solid was filtered off and washed several times with water. The crude material was crystallized with EtOH to afford a yellow solid (mp 230–231.6 °C, from EtOH). Yield 86%. ^1^H NMR (600 MHz, DMSO): δ 7.10 (*t*, 1H, J = 6.8 Hz, *H-b*), 7.44 (*t*, 1H, J = 7.3 Hz, *H-c*), 7.54 (*t*, 2H, J = 7.6 Hz), 7.61 (*t*, 1H, J = 7.9 Hz), 7.84 (*d*, 2H, J = 7.9 Hz), 8.19 (*m*, 2H), 8.24 (*d*, 1H, J = 8.9 Hz), 8.37 (*s*, 1H), 8.40 (*d*, 1H, J = 8.8 Hz) 8.64–8.70 (*m*, 2H). ^13^C NMR (151 MHz, DMSO): δ 90.7 (*C-f*), 113.7 (*C-b*), 117.3, 120.2, 124.0, 125.5, 126.9, 128.1, 128.6, 129.2, 129.7, 130.8, 137.8, 138.7 (*C-e*), 139.3, 140.7, 150.5, 164.1 (*C-g*). MS (ES^+^): 338 (M+1). ESI-HRMS (*m*/*z*): [M + H]^+^ calcd for C_22_H_16_N_3_O 338.1288; obsd. 338.1288.

### Molecular Modeling Docking

The structure of *h*DHODH complexed with MEDS433 (PDB ID: 6FMD) was used to rationally design the new compounds and to perform *in-silico* simulations. Before docking studies, the protein was prepared by adding hydrogens (all the His residues were considered as protonated on their e_2_ nitrogen according to the crystallographic data) and keeping the FMN and ORO cofactors. According to the experimental data,^33^ the compounds were simulated in the deprotonated form, bearing the negative charge on the oxygen atom, as this is the most populated tautomer at physiological pH. Compounds were submitted to docking with Glide. Poses were scored with the Chemscore function and ranked accordingly. In order to, validate the docking procedure, the self-docking of the ligand cocrystallized within *h*DHODH (PDB: 6FMD) was performed, obtaining a pose similar to the crystallographic one.

### *h*DHODH Inhibition Assay

Following the procedure described in Sainas et al., 2018,^22^ Inhibitory activity was assessed by monitoring the reduction of 2,6-dichloroindophenol (DCIP), which is associated with the oxidation of dihydroorotate catalyzed by the *h*DHODH enzyme GST-tagged. The enzyme (around 60 nM) was preincubated for 5 min at 37 °C in Tris-buffer solution (pH 8.0) with coenzyme Q10 (100 μM), tested compounds at different concentrations (final DMSO concentration 0.1% v/v), and DCIP (50 μM). The reaction was initiated by addition of DHO (500 μM) and the reduction was monitored at λ = 650 nm. The initial rate was measured in the first 5 min (ε = 10400 M^–1^cm^–1^) and the IC_50_ value was calculated starting from Vi/V0 (i = inhibitor; 0 = control) values using GraphPad Prism 7 software. Values are means ± SE of three independent experiments.

### Cell-Based Assays Cell Lines

The AML human cell lines THP1 (acute monocytic leukemia), K562 (chronic myeloid leukemia), A549 (lung adenocarcinoma) supplemented with 10% heat-inactivated fetal bovine serum (FBS) and 1% penicillin/streptomycin (GIBCO, Invitrogen, Milan, Italy) were purchased. MRC-5 cell line from ATCC were cultured in EMEM medium (LonzaBioWhittaker) supplemented with 10% heat-inactivated fetal bovine serum (FBS) and 1% penicillin/streptomycin (GIBCO, Invitrogen, Milan). *h*DHODH inhibitors were solubilized in DMSO (Sigma-Aldrich, Milan, Italy) and final dilutions of the drugs were made in culture medium.

### Annexin Assay

For the determination of EC_50_, 1 × 10^4^ THP1 cells were plated in 96-well round-bottom plates and treated with increasing doses of *h*DHODH inhibitors from 0.001 μM to 10 μM. After 3 days of culture, the apoptotic assay was performed using the Annexin V-FITC Kit (Miltenyi Biotec, Italy), according to the manufacturer’s instructions. The apoptotic cells were acquired on FacsVerse and analyzed using Kaluza software version 2.1 (Beckman Coulter, Fullerton, CA).

### Cell Viability Assay

For the determination of EC_50_, 1 × 10^4^ K562 cells or 3 × 10^3^ A549 were plated in 96-well plates and treated with increasing doses of *h*DHODH inhibitors from 0.001 μM to 10 μM. After 3 days of culture, the percentage of live cells was determined using the CellTiter-Glo Luminescent assay (Promega, Milan, Italy), following the manufacturer’s instructions.

### Cells and Viruses

The human ileocecal adenocarcinoma cell line HCT-8 (ATCC CCL-244) was purchased from the American Type Culture Collection (ATCC, USA) and cultured in RPMI (Euroclone, Pero (MI), Italy), supplemented with 10% (v/v) fetal bovine serum (FBS), 2 mM glutamine, 1 mM sodium pyruvate, 100 U/mL penicillin, and 100 μg/mL streptomycin sulfate (Euroclone). Human coronavirus OC43 (*h*CoV-OC43, ATCC VR-1558) was purchased from ATCC, and propagated and titrated as previously described in HCT-8 cells.^83^ The AML human cell lines THP1 (acute monocytic leukemia), was cultured in complete RPMI 1640 (Invitrogen Life Technologies, Gaithersburg, MD), supplemented with 10% heat-inactivated fetal bovine serum (FBS) and 1% penicillin/streptomycin (GIBCO, Invitrogen, Milan, Italy). *h*DHODH inhibitors were solubilized in DMSO (Sigma-Aldrich, Milan, Italy) and final dilutions of the drugs were made in culture medium.

### Antiviral Assay

The antiviral activity of **11a** and **14** against hCoV-OC43 was evaluated by means of the focus-forming reduction assay (FFRA) procedure as described by Sibille et al.^83^ Briefly, HCT-8 cells were seeded on 96-well plates (30,000 cells/well) and, after 24 h, treated with different concentrations of the **11a** or **14**, or with the vehicle (DMSO, as negative control) or with **MEDS433** (as positive control) 1 h prior to and during infection with *h*CoV-OC43 (50 PFU/well). After virus adsorption (2 h at 33 °C), the infected cell cultures were incubated in medium containing the corresponding compounds plus 1% (w/v) methylcellulose (Merck) and 1% (v/v) FBS. At 72 h post infection (p.i.), cell monolayers were fixed and subjected to indirect immunoperoxidase staining (IPA) with a mAb against the *h*CoV-OC43 nucleoprotein (N) (Millipore, clone 542-D7; Burlington, MA, USA) (diluted 1:100). Viral foci were microscopically counted, and the mean plaque counts for each drug concentration was converted in viral titer (PFU/mL). GraphPad Prism software version 8.0 was used to determine the concentration of compounds producing 50 and 90% reductions in viral titers (EC_50_ and EC_90_).

### Cytotoxicity Assay

HCT-8 cells (20,000 cells/well) or MRC-5 (6000 cells/well) cells were seeded in 96-well plates and, after 24 h, exposed to increasing concentrations of MEDS433, **11a**, **14,** or vehicle (DMSO) as a control. After 72 h of incubation, the number of viable cells was determined using the CellTiter-Glo Luminescent assay (Promega, Milan, Italy). The cytotoxicity of the tested compounds was expressed as cytotoxic concentration (CC_50_).

### Fluorescence Analysis and Live Cell Mitochondrial Imaging

For fluorescence analysis, 10 μM of target compounds were incubated with THP-1 for 24 h. After washing the cells with a PBS buffer solution, they were analyzed under confocal microscopy imaging technique using the appropriate laser to excite the molecule. For the colocalization experiments, HCT-8 cells (55,000 cells/well) were plated on μ-Slide 8 well high glass bottom (IBIDI, Gräfelfing, Germany) and treated for 24 h with 10 μM of **11a** at 37 °C. Then, cells were stained with *MitoTracker Red CMXRos* (ThermoFischer, Carlsbad, CA, USA) (1:4000) for 30′ at 37 °C and after washing with PBS buffer solution, cells were visualized with a Leica TCS SP5 multiphoton-inverted confocal microscope equipped with LAS AF matrix software. Live cells were imaged using an HCX PL APO 63×/1.4 NA oil immersion objective. Images were collected with excitation of ligand **11a** at λ_ex_ = 405 nm and emission at λ_em_ = 500 nm, *Red-MitoTracker* at λ_ex_ = 581 nm and emission at λ_em_ = 605 nm.

### Co-Localization Analysis

THP-1 cells were incubated for 3 h with *h*DHODH*i* **11a** and **Bay240223410** at 10 μM, either alone or in combination. After incubation, the cells were washed and stained with *Red-MitoTracker* CMXRos (Thermo Fisher, Carlsbad, CA, USA) at a concentration of 100 nM for 30 min at 37 °C. Cells were imaged with a Leica TCS SP8 confocal system (Leica Microsystems) equipped with 4 excitation lasers (405 Diode, Argon, DPSS561, HeNe633). Images were acquired with a HCX PL APO 63×/1.4 NA oil-immersion objective with a resolution of 81 nm × 81 nm and were processed and analyzed with ImageJ software (Rasband, W.S., U.S. National Institutes of Health, Bethesda, MA). In particular, colocalization was analyzed with JACoP plugin^84^ and Manders’ coefficient was calculated

### Statistical Analysis

Statistical analyses were performed on Prism software, version 8.0 (GraphPad Software, San Diego, CA). All data are reported as means ± SD. A Student’s *t* test was used as a significance test of different groups. A Wilcoxon test was performed for data not normally distributed. For colocalization analysis, a Welch’s *t* test was applied. For the determination of EC_50_, a nonlinear regression model was applied. Moreover, a p-value < 0.05 was considered significant.

### Photophysical Characterization

UV–Vis absorption spectra were recorded on a Cary60 spectrometer. Photoemission spectra, luminescence lifetimes, and quantum yields were acquired with a HORIBA Jobin Yvon IBH Fluorolog-TCSPC spectrofluorometer equipped with a Quanta-φ integrating sphere. Luminescence lifetimes were determined by time-correlated single-photon counting; excitation was achieved with nanosecond pulses of light generated by NanoLED pulsed diodes (operating wavelength 295, 370, or 450 nm). Emission-decay data were collected in 2048 channels to 10000 counts in the peak channel. IRF was measures using colloidal silica suspension in H_2_O to scatter light. Data were analyzed with the software DAS6 (TCSPC decay-analysis software) using a nonlinear least-squares method to fit of the decay to a sum of exponentials. The value of χ^2^, residuals and the autocorrelation function (Durbin-Watson parameter) were used to determine the quality of the fit. For measurements in the presence of hDHODH, 11a was incubated, at 1 μM concentration in TRIS Buffer 0.1 mM (pH 8) with 0.1% DMSO, alone or with different concentrations of recombinant protein ranging from 31.5 to 504 nM concentration. After 5 min incubation UV–Vis absorption spectra were recorded on a Cary60 spectrophotometer and photoemission spectra were acquired with a HORIBA Jobin Yvon IBH Fluorolog-TCSPC spectrofluorometer with excitation λ of 366 nm and maximum of emission at 500 nm; a.u. data were analyzed with GraphPad Prism using One Site – Specific Binding equation.

## Supplementary Material







## Notes

Synthesis of intermediates, ^1^H NMR, ^13^C NMR, and UHPLC (PDF)
The docking poses (PDB)
Molecular formula strings and biological data (CSV)

## Abbreviations

AML: acute myeloid leukemia
AIE: aggregation-induced emission effect
BIFL: bioactive intrinsically fluorescent ligands
CB2R: cannabinoid receptor subtype 2
CFSE: carboxyfluorescein diacetate succinimidyl ester
DCIP: dichloroindophenol
DHO: dihydroorotate
EDG: electron-donating group
ETC: electron transport chain
EWGs: electron-withdrawing groups
FMN: flavin mononucleotide
hDHODH: human dihydroorotate dehydrogenase
LiHMDS: lithium hexamethyldisilazane
NBD: nitrobenzoxadiazole
ORO: orotate
QY: quantum yield
SFR: Structure Fluorescence Relationship
SAR: Structure Activity Relationship
TFA: trifluoroacetic acid.
