# Supplementary material for: Fluorescent Isostere (Fluostere) of the Carboxylate: Design of hDHODH Fluorescent Inhibitors as Proof of Concept
Source: J Med Chem. 2025 Jun 18;68(13):13562–90. doi: 10.1021/acs.jmedchem.5c00348 (PMC12257544; doi:10.1021/acs.jmedchem.5c00348)
Supplement: Supplementary file 1 [file jm5c00348_si_001.pdf]

Fluorescent isostere (*Fluostere*) of the carboxylate: design of *h*DHODH fluorescent inhibitors as proof of concept

Stefano Sainas[1#], Elena Martino[1#], Claudio Garino[2], Paola Circosta[3,4], Anna Luganini[5], Marta Giorgis[1], Francesco Bavo[6], Marco Piccinini[7], Cristina Ramondetti[7], Marta Alberti[8], Riccardo Miggiano[8], Giorgio Gribaudo[5], Donatella Boschi[1], Bente Frølund[6] and Marco Lucio Lolli[1]*

[1] Department of Drug Science and Technology. University of Torino, Via Pietro Giuria 9, Torino 10125, Italy.
[2] Department of Chemistry, University of Torino, Via Pietro Giuria 7, Torino 10125, Italy.
[3] Department of Clinical and Biological Sciences, University of Torino, Regione Gonzole 10, Orbassano, Torino 10043, Italy.
[4] Molecular Biotechnology Center, University of Torino, Via Nizza 52, Torino 10126, Italy.
[5] Department of Life Sciences and Systems Biology, University of Torino, Via Accademia Albertina 13, Torino 10123, Italy.
[6] Department of Drug Design and Pharmacology, Faculty of Health and Medical Sciences, University of Copenhagen, Universitetsparken 2, DK-2100 Copenhagen, Denmark.
[7] Department of Oncology, University of Torino, Via Michelangelo 27/B, Torino 10125, Italy.
[8] Department of Pharmaceutical Sciences, University of Piemonte Orientale, Via G. Bovio 6, Novara 28100, Italy.
[#]Both authors contributed equally to this work.
Corresponding Authors
*Marco Lucio Lolli
E-mail: marco.lolli@unito.it.
Phone: +39 0116707180
Fax: +39 0116707162 (M.L.L.).

Supplementary materials

Table of Content	
Pages S2 – S9	Synthesis of compounds **35** – **47** and characterization
Page S10	Structural elucidation of intermediate **38b**
Page S11	Structural elucidation of intermediates **8b** and **8c**
Page S12	Apoptosis test rescue with uridine, and fluorescent analysis of **11a** with GST
Page S13 – S17	UHPLC analysis of final compounds **11** – **14**
Pages S18 - S120	^{1}H-NMR, ^{13}C-NMR and ^{19}F-NMR and 2D-NMR
Page S121	References

Synthesis and characterization of intermediate compounds:

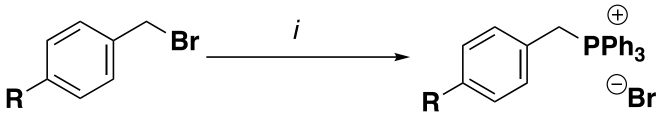

Scheme S1. Synthesis of intermediate compounds **35 a – e**. *i*) PPH_3, toluene, reflux.

General Procedure: the synthesis of phosphonium salt **35 a – e**.

To a stirred suspension of appropriate benzyl bromide (1 mmol) in dry toluene, triphenylphosphine (1 mmol) was added under a nitrogen atmosphere. The reaction mixture was stirred at 40°C for 48 hours until complete conversion of the starting material was observed. Then, the reaction mixture was cooled to room temperature and the precipitate was filtered under a nitrogen atmosphere to afford the appropriate phosphonium salt as a white solid, characterized only by mass spectroscopy.

Benzyltriphenylphosphonium salt (**35a**). Yield 90%. MS (ES^+): 354 (M+1).

(4-Mercaptobenzyl)triphenylphosphonium salt (**35b**). Yield 77%. MS (ES^+): 386 (M+1).

(4-(Methylsulfonyl)benzyl)triphenylphosphonium salt (**35c**). Yield 95%. MS (ES^+): 432 (M+1).

(4-Cyanobenzyl)triphenylphosphonium salt (**35d**). Yield 80%. MS (ES^+): 379 (M+1).

(4-Nitrobenzyl)triphenylphosphonium salt (**35e**). Yield 75%. MS (ES^+): 398 (M+1).

Synthesis of intermediates **41a** and **b**:

The synthesis of target compounds **41a** and **b** (Schemes S2 and S3), started from commercially available 4-bromo-2,3,5,6-tetrafluorobenzoic acid, which was protected in the carboxylic acid function by employing MeI in basic environment, leading compound **36**. This intermediate was then involved in a Suzuki cross-coupling reaction to yield compound **37**. Subsequently, this latter underwent reduction using $LiAlH_4$ in dry THF as the solvent. Since harsh reaction conditions were required (5 eq of $LiAlH_4$ and refluxed conditions) unexpectedly, this led to the formation of compound **38b** (for more info about the structure elucidation see the S10). To synthesize intermediate **38a**, the synthetic scheme was improved and modified starting from 4-bromo-2,3,5,6-tetrafluorobenzaldehyde as a starting material, which by a Suzuki cross-coupling reaction was converted into compound **39**. Subsequently, the latter was reduced using $NaBH_4$ in dry MeOH, resulting in the formation of compound **39a** in excellent yield. [1] The synthetic route was followed up by the bromination of intermediates **38a - b** using NBS and PPh_3, to afford compounds **39a - b**. These latter were then involved in the synthesis of a phosphonium salt by employing PPh_3 in dry toluene at reflux.

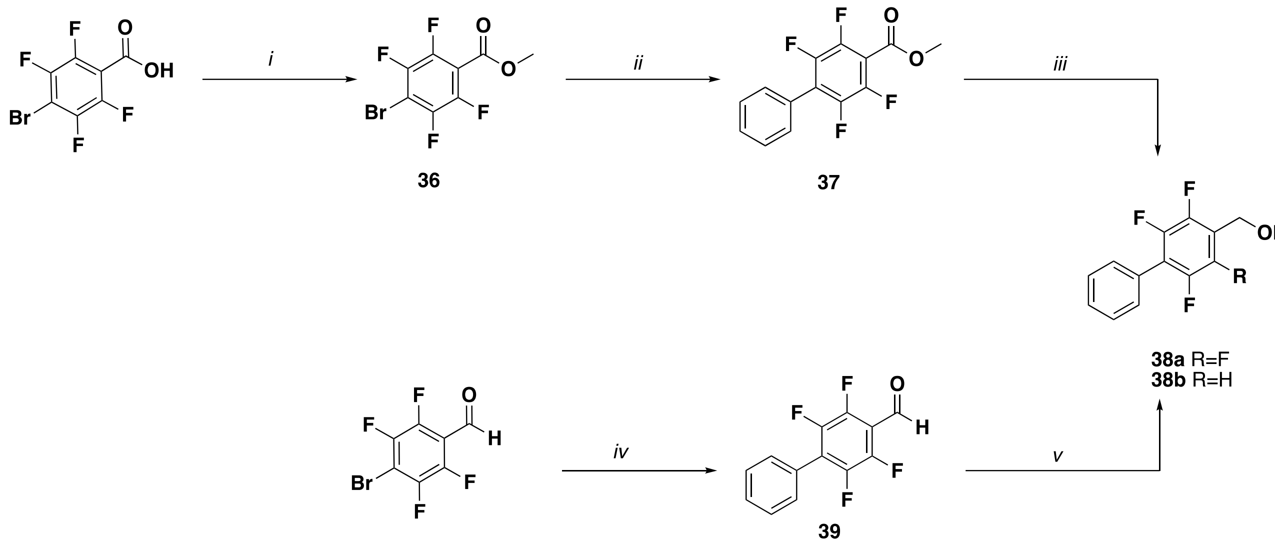

Scheme S2. Synthesis of the compounds **38 a – b.** *i*) MeI, K_2CO_3, dry DMF; *ii*) and *iv*) $Pd(PPH_3)_4$, K_2CO_3 (for *ii*) and Cs_2CO_3 (for *iv*), boronic acid, toluene/water 8:2 (for *ii*) and dioxane/water (for i*v*); *iii*) $LiAlH_4$, dry THF; *v*) $NaBH_4$, dry MeOH.

Methyl 4-bromo-2,3,5,6-tetrafluorobenzoate (**36**). Methyl iodide (4.25 g, 1.86 mL, 0.03 mol) was added dropwise to a mixture of 4-bromo-2,3,5,6-tetrafluorobenzoic acid (3.00 g, 0.01 mmol) and K_2CO_3 (4.14 g, 0.03 mmol) in dry DMF (20 mL). The reaction mixture was stirred for 1 hour at room temperature, then water (100 mL) was added. The mixture was extracted with diethyl ether (3 × 70 mL), the combined organic layers were dried over Na_2SO_4 and evaporated under reduced pressure to afford a yellow oil. The crude compound was purified using flash chromatography (eluent: *petroleum ether*/EtOAc 95/5 v/v) to afford the title compound a white solid (m.p. 57.6 – 58.1 °C, triturated from hexane). Yield 75 %. ^{1}H NMR (600 MHz, *Chloroform-d*): δ 3.99 (*s*, 1H).^{13}C NMR (151 MHz, *Chloroform-d*): δ 53.6 (-COOCH_3), 104.0 (*t*, *J* = 22.0 Hz, -*C*F), 112.2 (*t*, *J* = 15.9 Hz, -*C*F), 144.9 (*dd*, *J* = 259.7, 15.5 Hz, -*C*F), 145.2 (*dd*, *J* = 249.6, 14.1 Hz, -*C*F), 159.9.^{19}F NMR (565 MHz, *Chloroform-d*): δ -137.66 (*d*, J = 23.6, 12.8 Hz), -131.34 (*dd*, J = 22.4, 11.3 Hz). MS (ES$^+$): 288 (M+1).

Methyl 2,3,5,6-tetrafluoro-[1,1'-biphenyl]-4-carboxylate (**37**). $Pd[(P(Ph)_3)]_4$ (0.040 g, 0.03 mmol) was added to a solution of methyl 4-bromo-2,3,5,6-tetrafluorobenzoate (1 g, 3.48 mmol) and K_2CO_3 (1.44 g, 10.44 mmol,) in toluene/water (8:2 v/v 55 mL). After stirring the resulting mixture under a nitrogen atmosphere for 1 h, phenylboronic acid (6.96 mmol) was added; the reaction mixture was then stirred at reflux overnight. The day after, the reaction was cooled to room temperature and concentrated under reduced pressure. The crude material was *taken-up* with water (100 mL) and the mixture was extracted with EtOAc (3 x 60 mL). The combined organic layers were dried over Na_2SO_4 and concentrated under reduced pressure. The mixture was purified by flash chromatography (eluent*: petroleum ether*/EtOAc from 100 to 95/5 v/v) to afford the title compound as a white solid (m.p. 116.2 – 117.1 °C, triturated from diisopropyl ether). Yield 63 %.^{1}H NMR (600 MHz, *Chloroform-d*): δ 4.01 (*s*, 3H, -OC*H*$_3$), 7.39 – 7.74 (*m*, 5H).^{13}C NMR (151 MHz, *Chloroform-d*): δ 53.5 (-OCH$_3$), 111.4 (*t*, *J* = 16.0 Hz), 123.9 (*t*, *J* = 15.68 Hz), 126.7, 128.9, 129.9, 130.2, 144.0 (*d*, *J* = 250.35 Hz, -

*C*F), 145.1 (*d*, *J* = 256.10 Hz, -*C*F), 160.4 (-*C*OOMe). F^{19} NMR (565 MHz, *Chloroform-d*) δ: -139.5 (*dd*, *J* = 22.6, 11.7 Hz), -142.4 (*dd*, *J* = 22.6, 11.8 Hz).

2,3,5,6-Tetrafluoro-[1,1'-biphenyl]-4-carbaldehyde (**39**). $Pd[(P(Ph)_3)]_4$ (0.045 g, 0.039 mmol) was added to a solution of 4-bromo-2,3,5,6-tetrafluorobenzaldehyde (1 g, 3.89 mmol) and Cs_2CO_3 (3.80 g, 11.67 mmol,) in dioxane/water (8:2 v/v 40 mL) solution. After stirring the resulting mixture under a nitrogen atmosphere for 1 h, phenylboronic acid (7.78 mmol) was added and the reaction mixture was stirred at reflux. After 3h, the reaction is cooled to room temperature and concentrated under reduced pressure. The crude material was *taken-up* with water (100 mL) and the mixture was extracted with EtOAc (3 x 60 mL). The combined organic layers were dried over Na_2SO_4 and concentrated under reduced pressure. The mixture was purified by flash chromatography (eluent*: petroleum ether*/EtOAc from 100 to 90/10 v/v) to afford the title compound as a white solid (m.p. 98.5 – 100.2 °C, trituration from diisopropyl ether). ^1H NMR (600 MHz, *Chloroform-d*): δ 7.56 – 7.43 (*m*, 5H), 10.36 (*s*, 1H). ^{13}C NMR (151 MHz, *Chloroform-d*): δ 114.1 (*t*, *J* =9.52), 126.5, 126.9 (*t*, *J* =16.22), 129.0, 130.1, 130.2, 144.0 (*d*, *J* = 250.7 Hz, -*C*F), 147.1 (*d*, *J* = 262.3 Hz, -*C*F), 182.7. ^{19}F NMR (565 MHz, *Chloroform-d*): δ -145.11 – -145.71 (*m*), -142.37 – - 142.54 (*m*). MS (ES^+): 255 (M+1).

(2,3,5,6-Tetrafluoro-[1,1'-biphenyl]-4-yl)methanol (**38a**). A solution of **37** (0.350 g, 1.38 mmol) in dry MeOH (15 mL) was cooled to 0 °C. Then $NaBH_4$ (0.057 g, 1.5 mmol) was added portion-wise. The reaction mixture was stirred at room temperature until complete conversion of the starting material was observed. The mixture was quenched with water (200 mL) and the observed precipitate was isolated by filtration affording the title compound a white solid (m.p. 85.5 – 86.5 °C, from water). Yield 85 %. ^1H NMR (600 MHz, *Chloroform-d*): δ 2.02 (*t*, 1H, *J* = 6.5 Hz, -CH_2OH), 4.88 (*d*, 2H, *J* = 5.9 Hz, CH_2OH), 7.42 – 7.53 (*m*, 5H). ^{13}C NMR (151 MHz, *Chloroform-d*): δ 53.2 (-CH_2OH), 117.6 (*t*, *J* = 17.7 Hz), 120.9 (*t*, *J* = 17.30 Hz), 127.4, 128.8, 129.4, 130.2, 143.8 (*d*, *J* = 247.8 Hz), 145.4 (*d*, *J* = 245.6 Hz). F^{19} NMR (565 MHz, *Chloroform-d*): δ -145.23 (*dd*, *J* = 22.0, 13.3 Hz), -143.81 (*dd*, *J* = 23.4, 12.6 Hz).

2,3,6-Trifluoro-[1,1'-biphenyl]-4-yl)methanol **(38b**). A solution of **39** (0.500 g, 1.76 mmol) in dry THF (20 mL) was cooled to 0 °C. Then $LiAlH_4$ (0.333 g, 8.8 mmol) was added portion-wise. The reaction mixture was stirred at room temperature until complete conversion of starting material was observed. The mixture was quenched with 6 mL of water/NaOH 1 N (1:1). The water phase was extracted with EtOAc (3x 70 ml), dried over Na_2SO_4 and concentrated under reduced pressure. The mixture was purified by flash chromatography (eluent*: petroleum ether*/EtOAc from 100 to 80/20 v/v) to afford the title compound as a white solid (m.p. 76.1 – 77.6 °C, from trituration with diisopropyl ether). ^1H NMR (600 MHz, *Chloroform-d*): δ 1.92 (*t*, 1H, *J* = 6.1 Hz, -CH_2OH), 4.83 (*d*, 2H, *J* = 4.9 Hz, -CH_2OH), 7.05 – 7.14 (*m*, 1H), 7.35 – 7.38 (*m*, 5H). ^{13}C NMR (151 MHz, *Chloroform-d*): δ 58.6 (-CH_2OH), 110.1 (*td*, J= 3.59, 26 Hz, -*C*F), 119.2 (*d*, *J* = 16.8 Hz), 128.5, 128.6, 128.8, 130.3, 145.0 (*d*, *J* = 253.31 Hz, -*C*F), 145.1 (*d*, *J* = 258.94 Hz, -*C*F), 155.2 (*d*, *J* = 249.3 Hz, -*C*F). ^{19}F NMR (565 MHz, *Chloroform-d*). δ -148.3 (*t*, *J* = 18.1 Hz), -138.4 (*d*, *J* = 21.3 Hz), -119.5 (*t*, *J* =12.63 Hz).

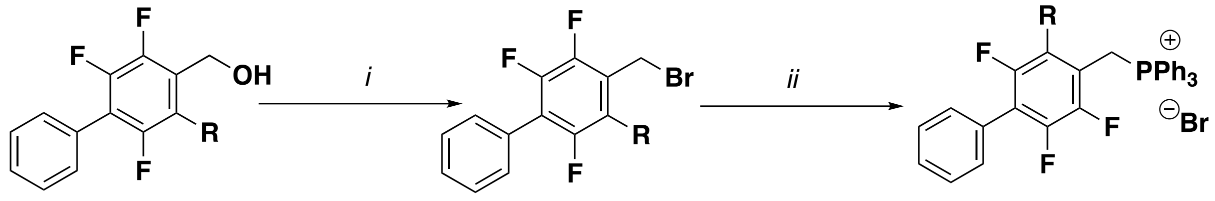

38a R= F
38b R= H

40a R= F
40b R= H

41a R= F
41b R= H

Scheme S2. Synthesis of the compounds **41 a – b.** *i*) NBS, PPH_3, dry toluene; *ii*) PPH_3, toluene, reflux.

4-(Bromomethyl)-2,3,5,6-tetrafluoro-1,1'-biphenyl (**40a**). A solution of (2,3,5,6-tetrafluoro-[1,1'-biphenyl]-4-yl)methanol (0.320 g, 1.25 mmol) and triphenylphosphine (0.360, 1.37 mmol) in dry dichloromethane (10 mL) was cooled at -10 °C. In the following, NBS (0.243 g, 1.37 mmol) in dry DCM (2 mL) was slowly dropped into the reaction mixture. The mixture was stirred until the end of the reaction was observed by TLC, and then the solvent was removed under reduced pressure. The mixture was purified by flash chromatography (eluent*: petroleum ether*) to afford the title compound as a white solid (m.p. 123.6 – 124.4 °C, triturated from diisopropyl ether). Yield 83 %.^{1}H NMR (600 MHz, *Chloroform-d*): δ 4.58 (*s*, 2H, -C*H*$_2$Br), 7.25 - 8.10 (*m*, 5H).^{13}C NMR (151 MHz, *Chloroform-d*): δ 16.8 (-*C*H$_2$Br), 115.8 (*t*, J=16.28 Hz), 121.5 (*t*, J=17.16 Hz), 127.2, 128.8, 129.5, 130.2, 143.8 (*d*= 252.01 Hz, -*C*F), 145.0 (*d*= 248.26 Hz, -*C*F).^{19}F (565 MHz, *Chloroform-d*): δ -143.31 (*dd*, *J* = 22.4, 11.9 Hz), -143.13 (*dd*, *J* = 21.6, 12.9 Hz).

4-(Bromomethyl)-2,3,6-trifluoro-1,1'-biphenyl (**40b**). A solution of (2,3,6-tetrafluoro-[1,1'-biphenyl]-4-yl)methanol (0.300 g, 1.26 mmol) and triphenylphosphine (0.363, 1.38 mmol) in dry dichloromethane (10 mL) was cooled at -10 °C. In the following, NBS (0.245 g, 1.38 mmol) in dry DCM (2 mL) was slowly dropped into the reaction mixture. The mixture was stirred until the end of the reaction was observed by TLC, and then the solvent was removed under reduced pressure. The mixture was purified by flash chromatography (eluent*: petroleum ether*) to afford the title compound as a white solid (m.p. 84.7 – 85.9 °C, triturated from diisopropyl ether). Yield 70 %. ^{1}H NMR (600 MHz, *Chloroform-d*): δ 4.51 (*d*, 2H, *J* = 1.0 Hz, -C*H*$_2$Br), 7.69 – 7.07 (*m*, 1H), 7.35 – 7.56 (*m*, 5H). ^{19}F NMR (565 MHz, *Chloroform-d*): δ -145.1 (*t*, *J* = 17.8 Hz), -136.8 (*d*, *J* = 20.7 Hz), -118.6 – 119.3 (*m*).

Triphenyl((2,3,5,6-tetrafluoro-[1,1'-biphenyl]-4-yl)methyl)phosphonium salt (**41a**).
Triphenylphosphine (0.230 g, 0.877 mmol) was added to a stirred suspension of 4-(bromomethyl)-2,3,5,6-tetrafluoro-1,1'-biphenyl (0.280 g, 0.877 mmol) in dry toluene, under nitrogen atmosphere. The reaction mixture was stirred at 40 °C for 48 hours until completely the conversion of the starting material was observed. The reaction mixture was cooled to room temperature and the resulting precipitate was filtered under a nitrogen atmosphere, to afford the phosphonium salt a white solid. Yield 90 %. ^{1}H NMR (600 MHz, *Chloroform-d*): *δ* 5.59 (*d*, 2H, J = 14.1 Hz, -C*H*$_2$P(Ph)$_3$), 7.34 (*d*, 2H, J = 7.1 Hz), 7.40 – 7.46 (*m*, 3H), 7.64 – 7.69 (*m*, 6H), 7.77 – 7.87 (*m*, 9H). ^{13}C NMR (565 MHz, *Chloroform-d*): *δ* 21.8 (*d*, J = 51.0 Hz, -*C*H$_2$P(Ph)$_3$), 107.1, 117.7 (*d*, J = 86.1 Hz), 121.83, 126.6, 128.8, 129.7 (*d*, J = 57.7 Hz), 130.5 (*d*, J = 12.8 Hz), 134.1 (*d*, J = 10.2 Hz), 135.6 (*d*, J = 2.7 Hz), 143.73 (d, J = 239.6 Hz), 145.39 (d, J = 239.9 Hz).

Triphenyl(3,5,6-tetrafluoro-[1,1'-biphenyl]-4-yl)methyl)phosphonium salt (**41b**). Triphenylphosphine (0.222 g, 0.849 mmol) was added to a suspension of 4-(bromomethyl)-3,5,6-tetrafluoro-1,1'-biphenyl (0.270 g, 0.849 mmol) in dry toluene, stirred under nitrogen atmosphere. The reaction mixture was stirred at 40 °C for 48 hours until complete conversion of the starting material was observed. Then, the reaction mixture was cooled to room temperature and the precipitate was filtered under a nitrogen atmosphere to afford the phosphonium salt as a white solid. Yield 89 %. ^{1}H NMR (600 MHz, *Chloroform-d*): δ 5.77 (*d*, 2H, J = 14.6 Hz -CH_2P(Ph_3)), 7.23 – 7.28 (*m*, 1H), 7.35 (*d*, 2H, J = 7.1 Hz), 7.38 – 7.46 (*m*, 3H), 7.66 – 7.71 (*m*, 6H), 7.78 – 7.84 (*m*, 3H), 7.85 – 7.91 (*m*, 6H). ^{13}C NMR (565 MHz, *Chloroform-d*): δ 23.77 (*d*, J = 49.9 Hz), 114.9 (*d*, J = 25.1 Hz), 117.1, 117.7, 127.7, 128.6, 129.1, 130.2, 130.5 (*d*, J = 12.7 Hz), 134.3 (*d*, J = 10.1 Hz), 135.5 (*d*, J= 2.6 Hz). ^{31}P NMR (243 MHz, *Chloroform-d*): 23.77.

Synthesis of intermediate **43**:

The synthesis of target compound 43 (Scheme S3), started from the commercially available 6-bromoquinolin-2-ol witch through a Suzuki-Miyaura coupling was converted into compound 42. This latter was treated with refluxed $POCl_3$, to replace the hydroxyl group with a chlorine atom, resulting in compound 43.

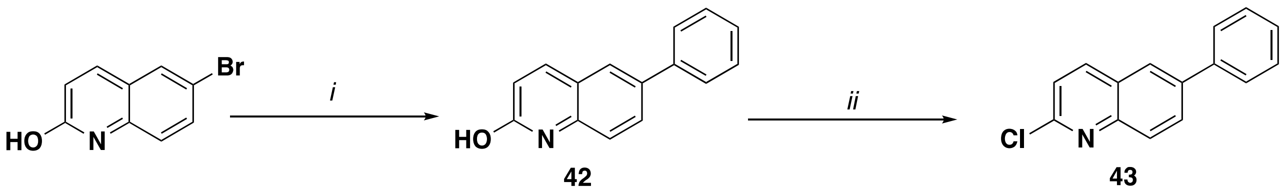

Scheme S3. Synthesis of intermediate compound **42**. *i*) Pd(PPH_3)$_4$, Cs_2CO_3, phenylboronic acid, dioxane/water 8/2 v/v, reflux; *ii*) $POCl_3$, reflux.

6-Phenylquinolin-2-ol (**42**). Pd(PPh_3)$_4$ (0.003 mmol) was added to a solution of 6-bromoquinolin-2-ol (0.301 mmol) and Cs_2CO_3 (0.905 mmol) in dioxane/water (8:2 v/v 10 mL) solution. After stirring the resulting mixture under a nitrogen atmosphere for 1 h, phenylboronic acid (0.301 mmol) was added and the reaction mixture was stirred at reflux. Upon completion, the reaction was cool down, the crude material was *taken-up* with water (50 mL) and the mixture was extracted with EtOAc (3 x 15 mL). The combined organic layers were dried over Na_2SO_4 and concentrated under reduced pressure. The crude was purified by RediSep Gold® RP Silica Gel disposable flash column, 24 g RP silica (eluent: ACN/Water from 100 to 95/5 v/v) to afford a crude compound. The latter was crystallized from dioxane to afford the title compound as a white solid (m.p. 279.2 – 281.8°C, decomposition, from dioxane). Yield 89%.^{1}H NMR (400 MHz, *DMSO-d$_6$*): δ 6.50 (*d*, 1H, *J* = 9.4 Hz), 7.29 – 7.41 (*m*, 2H), 7.46 (*t*, 2H, *J* = 7.7 Hz), 7.69 (*dd*, 2H, *J* = 8.3, 1.1 Hz), 7.78 (*dd*, 1H, *J* = 8.6, 2.1 Hz), 7.91 (*d*, 1H, *J* = 9.5 Hz), 7.93 (*d*, 1H, *J* = 2.0 Hz).^{13}C NMR (101 MHz, *DMSO-d$_6$*): δ 116.8, 119.7, 122.2, 125.4, 126.3, 127.0, 128.5, 128.9, 132.8, 139.6, 139.7, 140.0, 163.1 (-*C*O). MS (ES+): 222 (M+1).

2-Chloro-6-phenylquinoline (**43**). 6-Phenylquinolin-2-ol (2.25 mmol) was suspended in 2 mL of $POCl_3$. The suspension was warm at reflux until complete conversion of starting material was

observed by TLC. The mixture was quenched with water and basified until pH 8. The resulting solid was filtered and washed several times with water to afford the title compound as a white solid (m.p. 109.3 – 110.1°C, from water). Yield 73%.^{1}H NMR (400 MHz, *Chloroform-d*): δ 7.37 – 7.46 (*m,* 2H), 7.50 (*t*, *J* = 7.6 Hz, 2H), 7.70 (*d*, 2H, *J* = 7.3 Hz), 7.94 – 8.04 (*m*, 2H), 8.09 (*d*, 1H, *J* = 9.2 Hz), 8.15 (*d*, 1H, *J* = 8.6 Hz).^{13}C NMR (101 MHz, *Chloroform-d*): δ 122.9, 125.4, 127.2, 127.6, 128.1, 129.1, 129.2, 130.5, 139.2, 140.0, 140.1, 147.4, 150.7. MS (ES+): 240 (M+1).

Synthesis of intermediate **44**:

To achieve intermediate **44**, 4-bromo-2-nitrobenzaldehyde was first selectively reduced on the -NO_2 function using Fe in CH_3COOH to afford the corresponding 2-amino-4-bromobenzaldehyde, which was then immediately used in a cyclization reaction in the presence of 2-chloro-1,1-dimethoxyethane and a catalytic amount of 96 % w/w H_2SO_4 to afford intermediate **44** in good yield.

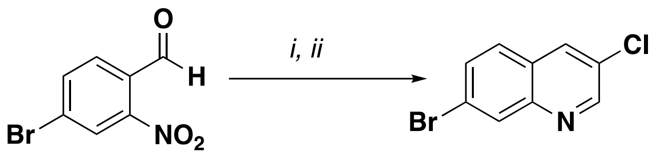

44

Scheme S4. Synthesis of intermediate compound **44**. *i*) Fe, CH_3COOH, reflux; *ii*) 2-chloro-1,1-dimethoxyethane, H_2SO_4 cat., toluene.

7-Bromo-3-chloroquinoline (**44**). 2-Nitro-4-bromobenzaldehyde (2 g, 8.69 mmol) was suspended in EtOH/Acetic acid (1:1 v/v 40 mL) solution under a nitrogen atmosphere. The suspension was cooled down at 0° and then, Fe powder was added (1.4 g, 26.07 mmol). The reaction was stirred at rt until complete conversion of starting material was observed by TLC. Insoluble was filtered off and the filtrate was concentrated under vacuum. The residue was taken with ethyl acetate (50 mL) and washed several times with sodium bicarbonate before drying with Na_2SO_4 and concentrated under reduced pressure. The crude product was purified by Combiflash using RediSep Gold® Silica Gel disposable flash column, 40 g of silica (eluent: *petroleum ether*/EtOAc from 100 to 80/20 v/v) to affords the title compound as a pale yellow solid. Yield 64%. The latter was immediately used in the next step of reaction.

A mixture of 2-Chloro-1,1-diethoxyethane (1.428 g, 9 mmol), 2-amino-4-bromobenzaldehyde (0.900 g, 4.5 mmol), and *p*-toluene sulfonic acid (0.109 g, 0.45 mmol) in toluene (10 mL) was heated at 110° using a Dean/Stark apparatus. Upon complete conversion of starting material was observed by TLC, the solvent was removed under reduced pressure. The crude material was *taken up* with $NaHCO_3$ (100 mL) and the mixture was extracted with EtOAc (3 x 60 mL). The combined organic layers were dried over Na_2SO_4 and concentrated under reduced pressure. The crude mixture was purified by Combiflash using RediSep Gold® Silica Gel disposable flash column, 12 g of silica (eluent: *petroleum ether*/EtOAc from 100 to 95/5 v/v) to affords the title compound as white solid (m.p. 115.2 – 116.9°C, triturated from hexane). Yield 83%.^{1}H NMR (600 MHz, *Chloroform-d*): δ 7.62 (*d*, 1H, J = 8.6 Hz), 7.67 (*d*, 1H, J = 8.5 Hz), 8.11 (*s*, 1H), 8.28 (*s*, 1H), 8.82 (*s*, 1H). ^{13}C NMR (151 MHz, *Chloroform-d*): δ 123.8, 127.1, 128.3, 129.0, 131.4, 132.0, 134.0, 146.9, 150.8. MS (ES+): 242, 244 (M+1).

Alternative synthesis for target compound 13:

For the synthesis of target compound **13**, an alternative pathway has been explored. The synthesis started with an iron-mediated domino reductive cyclization reaction between 4-bromo-2-nitrobenzaldehyde and (E)-(2-nitrovinyl)benzene in acetic acid to afford the 3-arylquinoline **45**. [2] The resulting intermediate **45** was then subjected to a Suzuki-Miyaura cross-coupling reaction to yield pinacol boronic ester compound **46**. Subsequently, the latter was subjected to another *Suzuki-Miyaura* cross-coupling reaction, this time with compound **20**. This reaction afforded the pre-final compound **47** in good yield. The final step involved the deprotection of methyl group by refluxing compound47 in 48 % w/w HBr, leading the target compound **13** in high yield.

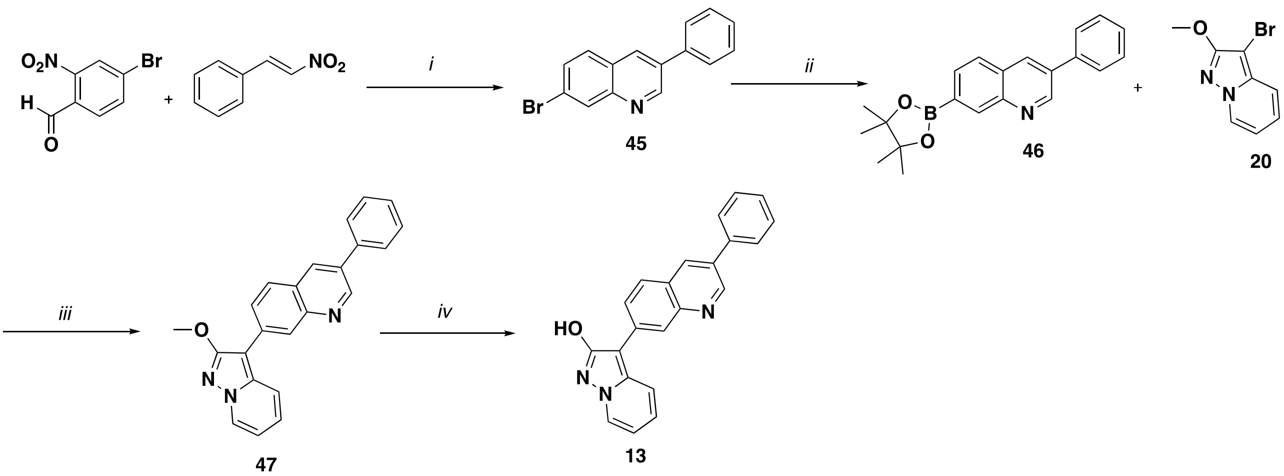

Scheme S6. Alternative synthesis of target compound **13** *i*) Fe, CH_3COOH, reflux; *ii*) Bis(pinacolato)diboron, Pd(dppf)Cl_2, NaOAc, dioxane, reflux; *iii*) Pd[P(Ph)$_3$]$_4$, Cs_2CO_3, dioxane/water 8/2 v/v, reflux; *iv*) HBr, reflux.

7-Bromo-3-phenylquinoline (**45**). Fe (1.12 g, 20.11 mmol) was added to a solution of *E*-(2-nitrovinyl)benzene (1.00 g, 6.70 mmol) and 4-bromo-2-nitrobenzaldehyde (1.54 g, 6.70 mmol) in AcOH (25 mL). The reaction was stirred at reflux until complete conversion of the starting material was observed. Then, the reaction was cooled at room temperature and quenched in water (100 mL). The obtained suspension was filtered off and then the filtrate was neutralized until pH 7 with 5M NaOH solution. The water phase was extracted with EtOAc (3 x 50 mL). The combined organic layers were dried over Na2SO4 and concentrated under reduced pressure. The crude material was purified by Combiflash using RediSep Gold Silica Gel disposable 40 g of silica, (eluent*: petroleum ether*/EtOAc from 95/5 v/v to 80/20 v/v) to afford the title compound as a pale-yellow solid (m.p. 123.4 – 124.6 °C). Yield 10.8 %.^{1}H NMR (600 MHz, *Chloroform-d*): δ 7.46 (*t*, 1H, J = 7.4 Hz), 7.54 (*t*, 2H, J = 7.6 Hz), 7.67 (*dd*, 1H, J = 8.7, 1.7 Hz), 7.71 (*d*, 2H, J = 7.3 Hz), 7.76 (*d*, 1H, J = 8.7 Hz), 8.27 (*d*, 1H, J = 1.8 Hz), 8.33 (*s*, 1H), 9.19 (*d*, 1H, J = 2.0 Hz). ^{13}C NMR (151 MHz, *Chloroform-d*): δ 123.5, 126.8, 127.5, 128.5, 129.38, 129.42, 130.8, 131.7, 133.2, 134.4, 137.6, 148.0, 151.0. MS (ES^+): 284, 285, 286 (M+1).

3-Phenyl-7-(4,4,5,5-tetramethyl-1,3,2-dioxaborolan-2-yl)quinoline (**46**). NaOAc (0.173 g, 2.11 mmol), compound **45** (0.200 g, 0.704 mmol), and PdCl_2(dppf), (0.005 mg, 0.007 mmol) were dissolved in 5 mL of dioxane, under a nitrogen atmosphere. The reaction mixture was stirred for 40

minutes at room temperature, and then bis(pinacolato)diboron (0.214 g, 0.844 mmol) was added. The reaction was stirred at 100 °C for 48 hours. Upon completion, the solution was cooled at room temperature, and the solvent was evaporated under reduced pressure. The mixture was purified by Combiflash using RediSep Gold Silica Gel disposable 24 g of silica (eluent: DCM/EtOAc 70/30 v/v), to afford a crude compound. This latter was then triturated with diisopropyl ether to obtain the title compound as a white solid. Yield 51 %.^{1}H NMR (600 MHz, *Chloroform-d*): δ 1.41 (*s*, 12H, -CH_3), 7.44 (*t*, 1H, J = 7.4 Hz), 7.53 (*t*, 2H, J = 7.7 Hz), 7.73 (*dd*, 2H, J = 8.3, 1.2 Hz), 7.86 (*d*, 1H, J = 8.0 Hz), 7.93 (*dd*, 1H, J = 8.1, 1.1 Hz), 8.29 (*d*, 1H, J = 2.0 Hz), 8.63 (*s*, 1H), 9.21 (*d*, 1H, J = 2.3 Hz). ^{13}C NMR (151 MHz, *Chloroform-d*): δ 25.1, 84.3, 127.3, 127.6, 128.4, 129.3, 129.9, 131.8, 133.1, 134.6, 137.3, 138.0, 147.0, 150.2. MS (ES$^+$): 332 (M+1).

7-(2-Methoxypyrazolo[1,5-a]pyridin-3-yl)-3-phenylquinoline (**47**). Pd(PPh_3)$_4$ (3.46 mg, 0.003 mmol) was added to a solution of 3-bromo-2- methoxypyrazolo[1,5-*a*]pyridine (0.068 g, 0.301 mmol) and Cs_2CO_3 (0.294 g, 0.905 mmol) in dioxane/water (8:2 v/v, 10 mL) solution. After stirring the resulting mixture under a nitrogen atmosphere for 1 h, 3-phenyl-7-(4,4,5,5-tetramethyl-1,3,2- dioxaborolan-2-yl)quinoline (0.100 g, 0.301 mmol) was added and the reaction mixture was heated at reflux. Upon completion, the reaction was cool down, the crude material was *taken-up* with water (50 mL) and the mixture was extracted with EtOAc (3 x 15 mL). The combined organic layers were dried over Na_2SO_4 and concentrated under reduced pressure. The crude mixture was purified by Combiflash using RediSep Gold RP Silica Gel disposable flash column, 12 g of RP silica (eluent: Water/ACN from 95/5 to 70/30 v/v) to afford a solid crude. This latter was triturated with diisopropyl ether to obtain the title compound as a white solid (m.p. 161.3 - 162.1 °C, from trituration with diisopropyl ether). Yield 92 %.^{1}H NMR (600 MHz, *Chloroform-d*): δ 4.18 (*s*, 3H, -OCH_3), 6.71 (*t*, 1H, *J* = 6.8, *H-b*), 7.20 (*t*, 1H, *J* = 7.9, *H-c*), 7.44 (t, 1H, *J* = 7.4 Hz), 7.53 (*t*, 2H, *J* = 7.7 Hz), 7.74 (*d*, 2H, *J* = 7.2 Hz), 7.84 (*d*, 1H, *J* = 9.0 Hz, *H-d*), 7.91 (*d*, 1H, *J* = 8.5 Hz), 8.01 (*dd*, 1H, *J*= 8.5, 1.5 Hz), 8.29 (*s*, 1H), 8.32 (*d*, 1H, *J* = 6.9 Hz, *H-a*), 8.40 (*s*, 1H), 9.17 (*d*, 1H, *J* = 2.3 Hz). ^{13}C NMR (600 MHz, *Chloroform-d*): 56.7 (-O*C*H_3), 95.0, 110.6 (*C-b*), 116.2 (*C-d*), 125.5 (*C-c*), 125.9, 126.2, 127.5, 128.1, 128.2, 129.1, 129.3, 133.0, 133.2, 134.1, 138.2, 139.3 (*C-e*), 148.1, 150.2, 163.4 (*C-g*). MS (ES$^+$): 352 (M+1).

3-(3-Phenylquinolin-7-yl)pyrazolo[1,5-a]pyridin-2-ol (**13**). 7-(2-methoxypyrazolo[1,5-*a*]pyridin-3-yl)-3-phenylquinoline (1 mmol) is suspended in 4 mL of HBr. The suspension is warm at reflux until the complete conversion of the starting material is observed by TLC. The resulting solid is filtered and washed several times with water to afford an orange solid. Yield 85%.^{1}H NMR (600 MHz, *DMSO-d$_6$*): δ 6.98 (*t*, 1H, *J* = 6.5, *H-b*), 7.51 (*t*, 1H, *J* = 7.7, 1H), 7.55 (*t*, 1H, *J* = 7.1 Hz, 1H), 7.62 (*t*, 2H, *J* = 7.3 Hz), 8.02 (*d*, 2H, *J* = 7.5 Hz), 8.04 (*d*, 1H, *J* = 9.1 Hz, *H-d*), 8.38 (*d*, 1H, *J* = 8.7 Hz), 8.46 (*d*, 1H, *J* = 8.6), 8.61 (*d*, 1H, *J* = 6.6 Hz, *H-a*), 8.63 (*s*, 1H), 9.47 (*s*, 1H), 9.64 (*d*, 1H, *J* = 2.3 Hz). ^{13}C NMR (600 MHz, *DMSO-d$_6$*): δ 92.3 (*C-f*), 112.2 (*C-b*), 113.7,115.5 (*C-d*), 126.1, 127.2, 127.4 (*C-c*), 128.5, 129.3, 129.4, 129.5, 129.6, 131.8, 134.1, 137.8, 138.5, 139.5, 141.9, 143.3, 162.73. MS (ES$^+$): 338 (M+1).

Structural elucidation of intermediate 37b:

^{13}C chemical shift and heteronuclear ^{1}H-^{13}C HMBC correlations have been proved to be diagnostic analyses for the characterization of compound **38b.** In detail, a $^{3}J_{H-C}$ correlation between the H_3 and the C_1 can be observed. This data, in addition with ^{19}F analysis of the two intermediate **38a** and **38b** confirmed the chemical structure of compound **38b**.

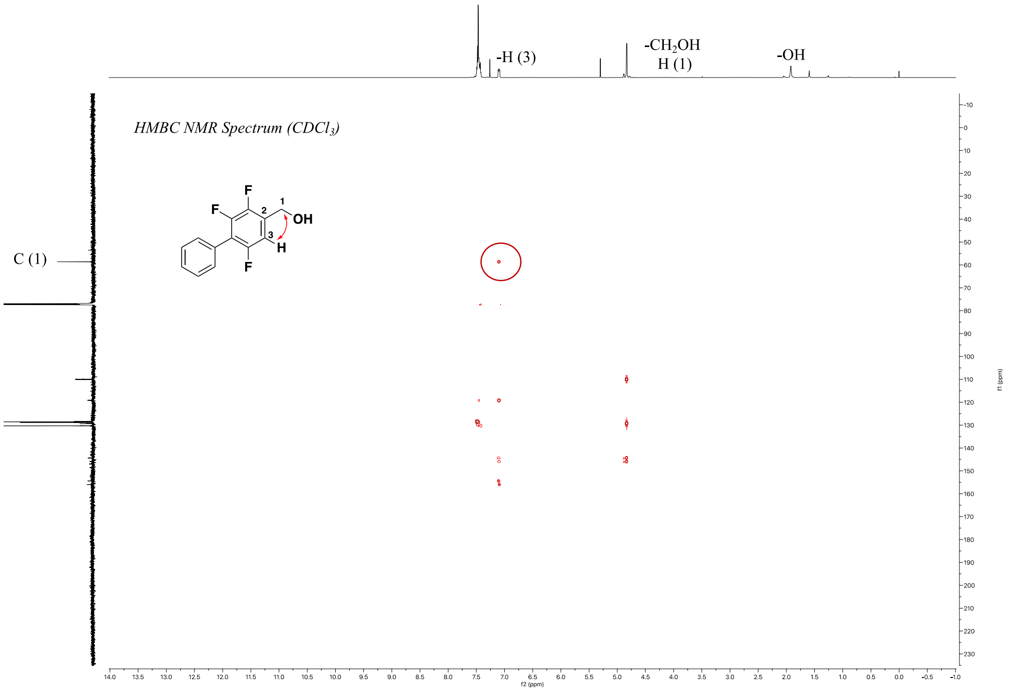

S1. HMBC analysis of intermediate **38b**.

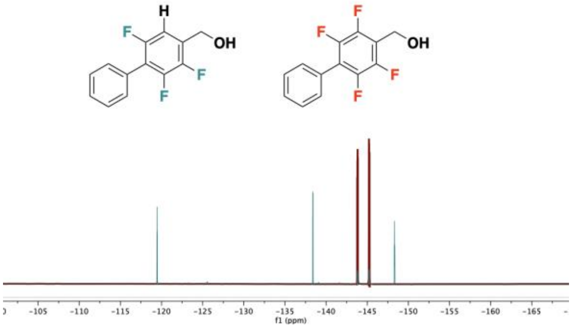

S2. ^{19}**F** spectrum of intermediates **38a** and **38b**.

Structural elucidation of target compounds 8b and 8c

The structure of the target compounds **8b** and **8c** was confirmed by ROESY analysis. In detail, in compound **8b**, a cross-coupling between proton **c** and proton **d** can be observed, together with cross-coupling between -NMe_2 and the mobile -OH proton.

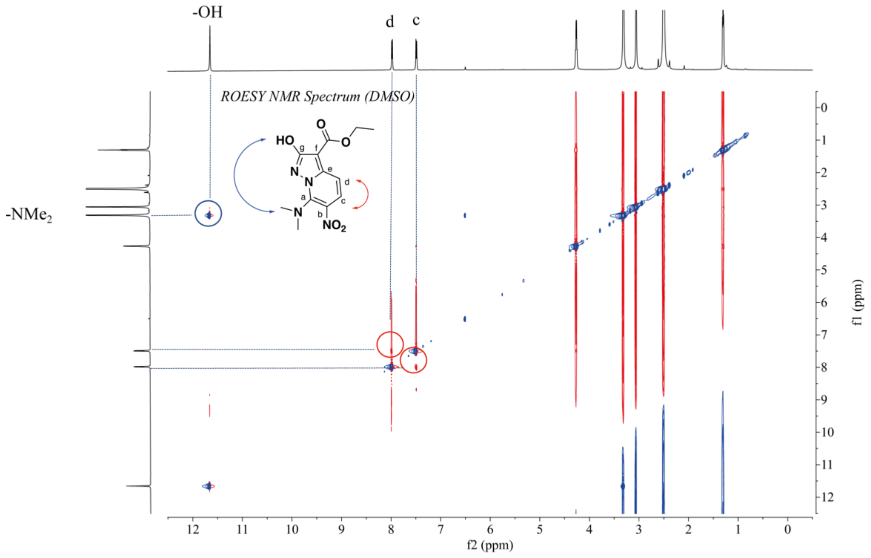

S3. ROESY analysis of target compound **8b**

When analyzing the spectrum of compound **8c** instead, proton **b** shows a cross-coupling with proton **c** and a cross-coupling with the -NMe_2 group. Moreover the -NMe_2 group shows cross-coupling with the mobile -OH group.

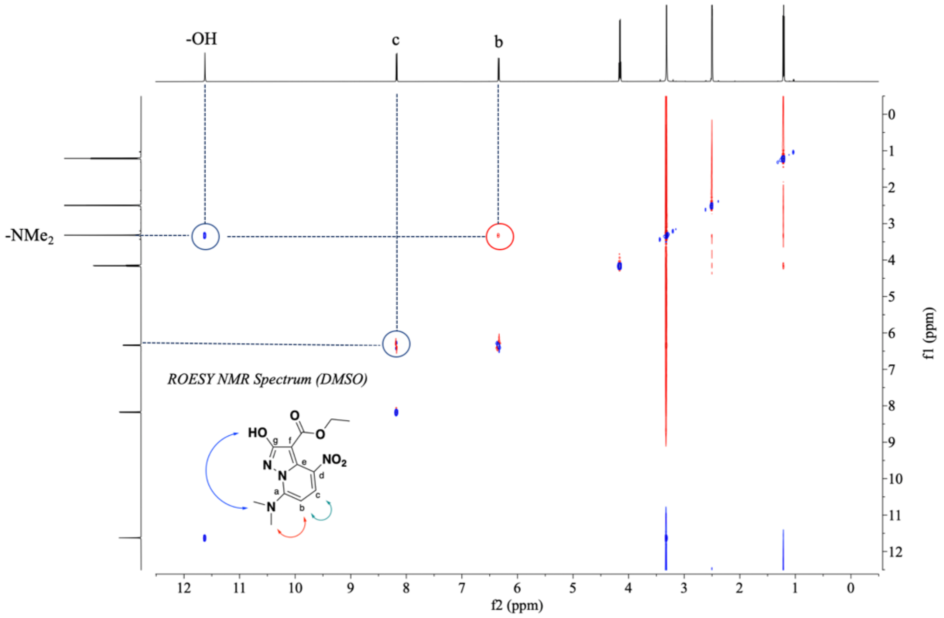

S3. ROESY analysis of target compound **8c**.

Apoptosis test rescue with uridine:

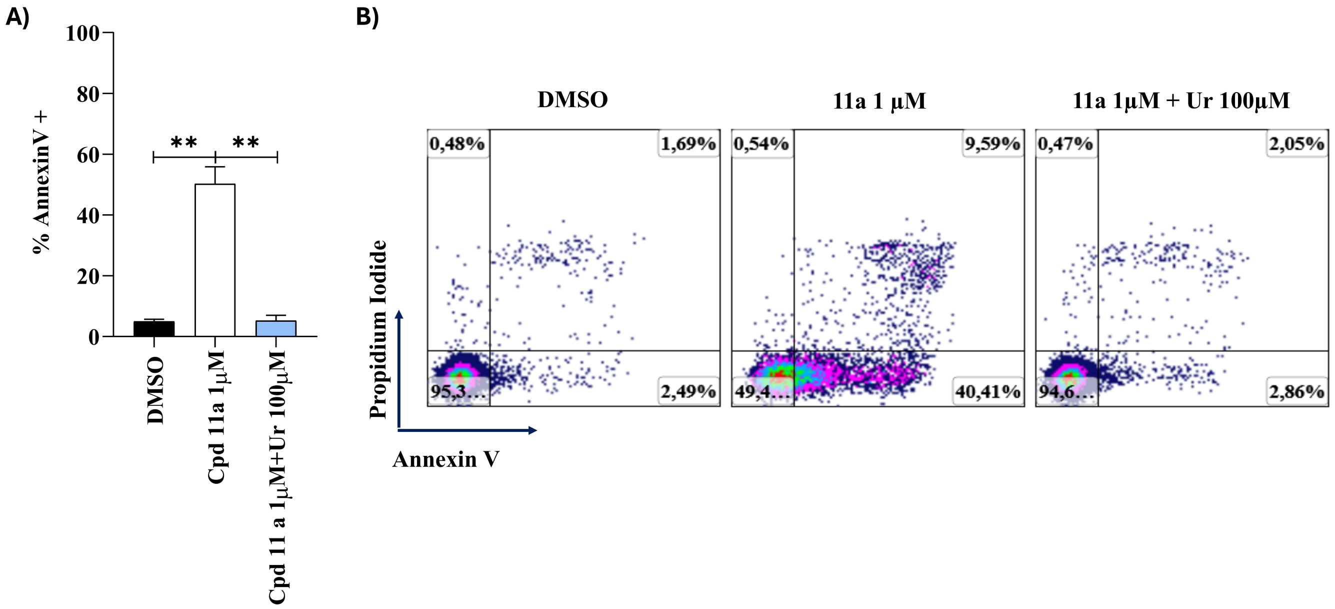

Figure S4. A) Analysis of the apoptotic rate induced by compound **11a**, on THP-1 cell line (n = 3) with or without exogenous uridine at 100 µM. Compounds were utilized at 1 µM; apoptotic was evaluated after 3 days of treatment. DMSO: dimethyl sulfoxide. Cpd: compound. Ur: uridine. Statistical significance: Student's t test: **p<0.01. B) Representative Annexin V-FITC/PI flow cytometry dot plots of THP-1 cells after 3 days of treatment in presence of vehicle (left), compound **11a** (middle), compound **11a** and uridine 100 µM. Data are representative of 3 independent experiments.

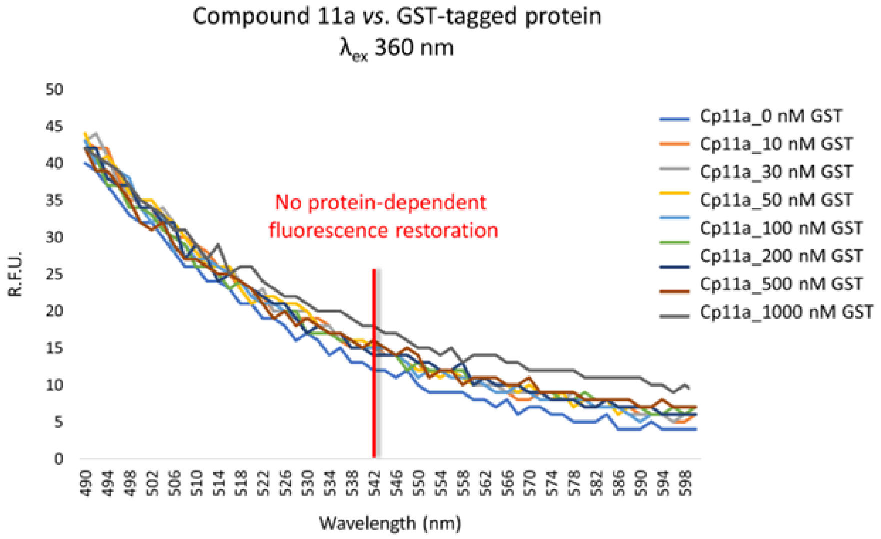

Figure S5. Fluorescence-based analysis of the potential contribution of GST-tag to the emission restoration of compound **11a**. As showed in the plot, a GST-conjugated protein has been tested at increasing concentration versus a fixed amount of compound **11a.** All the tested conditions did not show protein-dependent fluorescence restoration.

UHPLC analysis of target compounds:

Compound **12**:

4/14/2023 2:26:27 PM EM-422 MS 7 ELENA

Report UHPLC

Acquisition Date/Time	3/7/2023 1:01:14 PM	Sample Name	EM-422
Acquisition Method	MS 7 ELENA	Injection Volume	4
Instrument Name	UHPLC		

EM-422 : 254:10:360:10 : 1

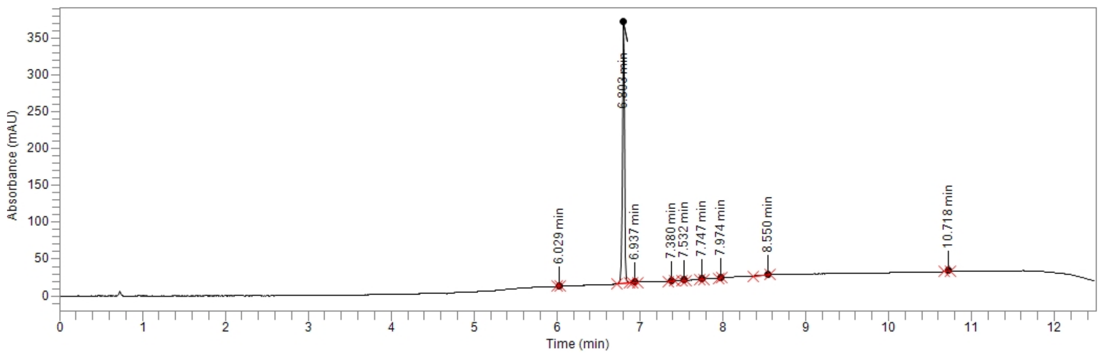

Channel Name **254:10:360:10**

Component Name	Time	Height	Area	Area %	Peak Purity	Wavelength Maximum
	6.029	259.9	387.0	0.06	1.16Pass	202
	6.803	355,313.0	604,479.7	97.96	1.36Pass	206
	6.937	577.9	953.8	0.15	1.45Pass	202
	7.380	1,511.8	2,426.6	0.39	1.35Pass	202
	7.532	923.5	1,321.6	0.21	1.08Pass	202
	7.747	518.1	589.6	0.10	1.16Pass	202
	7.974	380.7	372.8	0.06	1.10Pass	204
	8.550	394.7	2,996.8	0.49	1.23Pass	204
	10.718	1,613.4	3,562.5	0.58	1.09Pass	204
Total			617,090.4	100.00		

Compound **13**:

Report UHPLC

Acquisition Date/Time	3/7/2023 12:43:20 PM	Sample Name	EM-412
Acquisition Method	MS 7 ELENA	Injection Volume	4
Instrument Name	UHPLC		

EM-412 : 254:10:360:10 : 1

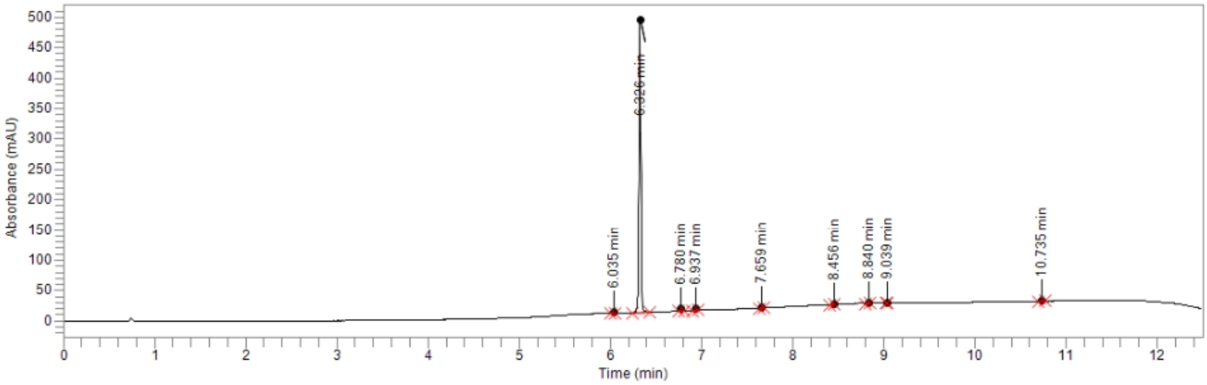

Channel Name **254:10:360:10**

Component Name	Time	Height	Area	Area %	Peak Purity	Wavelength Maximum
	6.035	414.2	756.9	0.10	1.17Pass	202
	6.326	482,416.1	757,875.1	97.81	1.45Pass	230
	6.780	3,144.0	4,772.8	0.62	1.30Pass	202
	6.937	2,987.9	4,829.5	0.62	1.19Pass	202
	7.659	1,150.3	1,646.1	0.21	1.19Pass	202
	8.456	170.8	711.1	0.09	1.19Pass	204
	8.840	162.0	392.2	0.05	1.11Pass	204
	9.039	114.3	106.7	0.01	1.10Pass	204
	10.735	1,630.3	3,761.5	0.49	1.20Pass	204
Total			774,851.9	100.00		

Compound **14**:

Report UHPLC

Acquisition Date/Time	3/7/2023 12:25:28 PM	Sample Name	EM-562
Acquisition Method	MS 7 ELENA	Injection Volume	4
Instrument Name	UHPLC		

EM-562 : 254:10:360:10 : 1

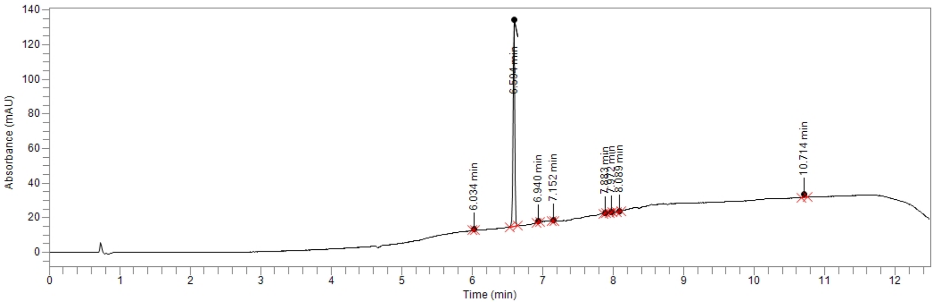

Channel Name **254:10:360:10**

Component Name	Time	Height	Area	Area %	Peak Purity	Wavelength Maximum
	6.034	261.6	412.2	0.21	1.24Pass	202
	6.594	119,792.9	189,664.0	95.67	1.36Pass	202
	6.940	540.6	744.3	0.38	1.38Pass	202
	7.152	528.1	609.7	0.31	1.15Pass	202
	7.883	412.9	538.9	0.27	1.14Pass	202
	7.972	533.1	690.6	0.35	1.13Pass	202
	8.089	459.9	784.6	0.40	1.34Pass	202
	10.714	1,906.9	4,806.4	2.42	1.32Pass	204
Total			198,250.7	100.00		

Compound **11a**:

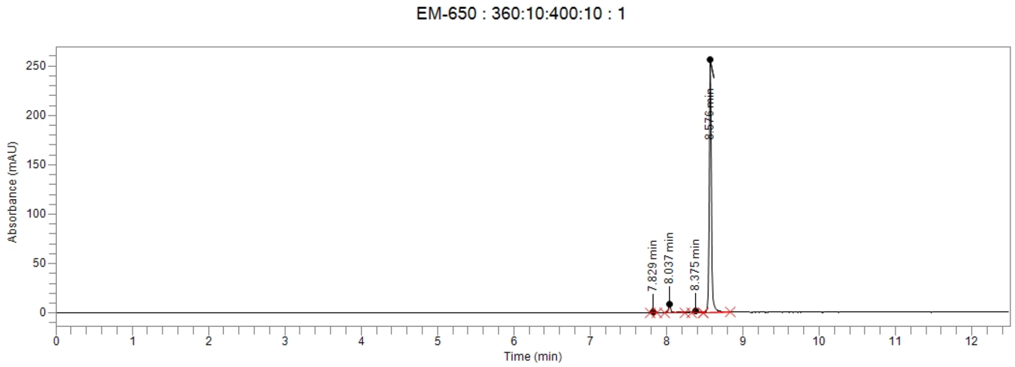

Channel Name **360:10:400:10**

Component Name	Time	Height	Area	Area %	Peak Purity	Wavelength Maximum
	7.829	567.8	1,170.0	0.23	1.25Pass	201
	8.037	8,208.0	15,148.7	2.97	1.20Pass	201
	8.375	2,005.6	3,929.7	0.77	1.29Pass	201
	8.576	256,553.1	489,864.1	96.03	1.12Pass	202
Total			510,112.5	100.00		

Compound **11b**:

Report UHPLC

Acquisition Date/Time	2/9/2022 1:44:09 PM	Sample Name	M564
Acquisition Method	MS3 564	Injection Volume	1
Instrument Name	UHPLC		

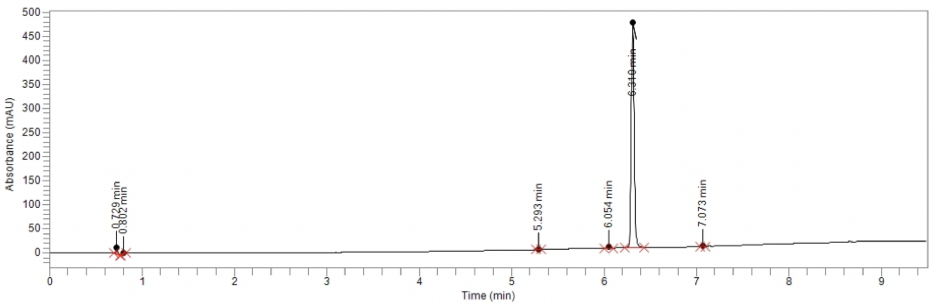

Channel Name **254:10:600:10**

Component Name	Time	Height	Area	Area %	Peak Purity	Wavelength Maximum
	0.729	13,717.8	18,048.2	1.62	76.55Fail	201
	0.802	2,725.3	7,011.5	0.63	8.52Fail	195
	5.293	1,093.5	2,084.0	0.19	1.87Fail	201
	6.054	3,080.3	6,392.1	0.57	1.18Pass	201
	6.310	468,267.2	1,080,956.7	96.73	1.20Pass	204
	7.073	1,646.5	3,006.0	0.27	1.18Pass	202
Total			1,117,498.4	100.00		

(Ethoxycarbonyl)(pyridin-1-ium-1- yl)amide (**15**)

^{1}H NMR Spectrum (*CDCl$_3$*)

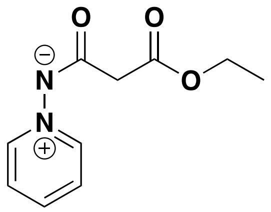

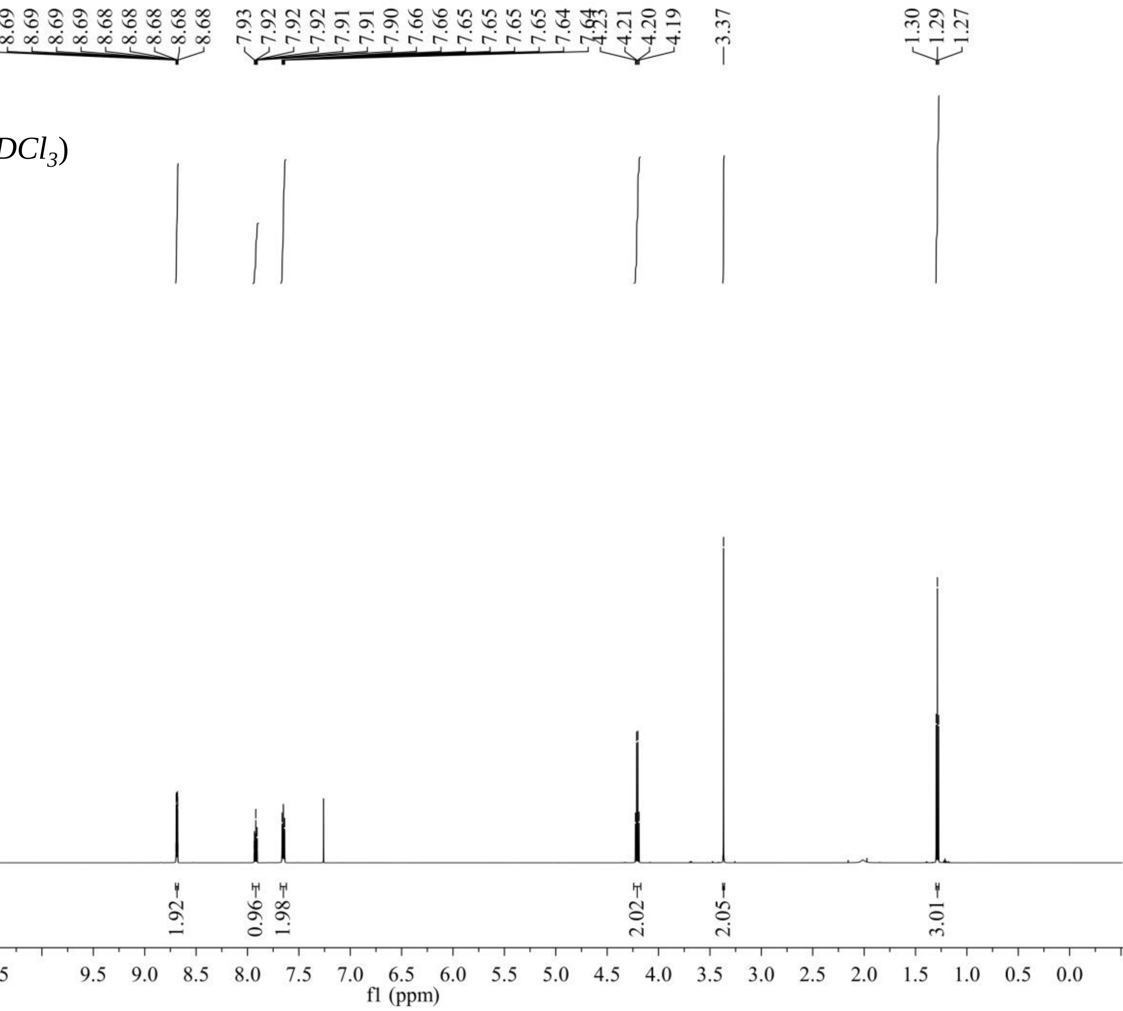

^{13}C NMR Spectrum (*CDCl$_3$*)

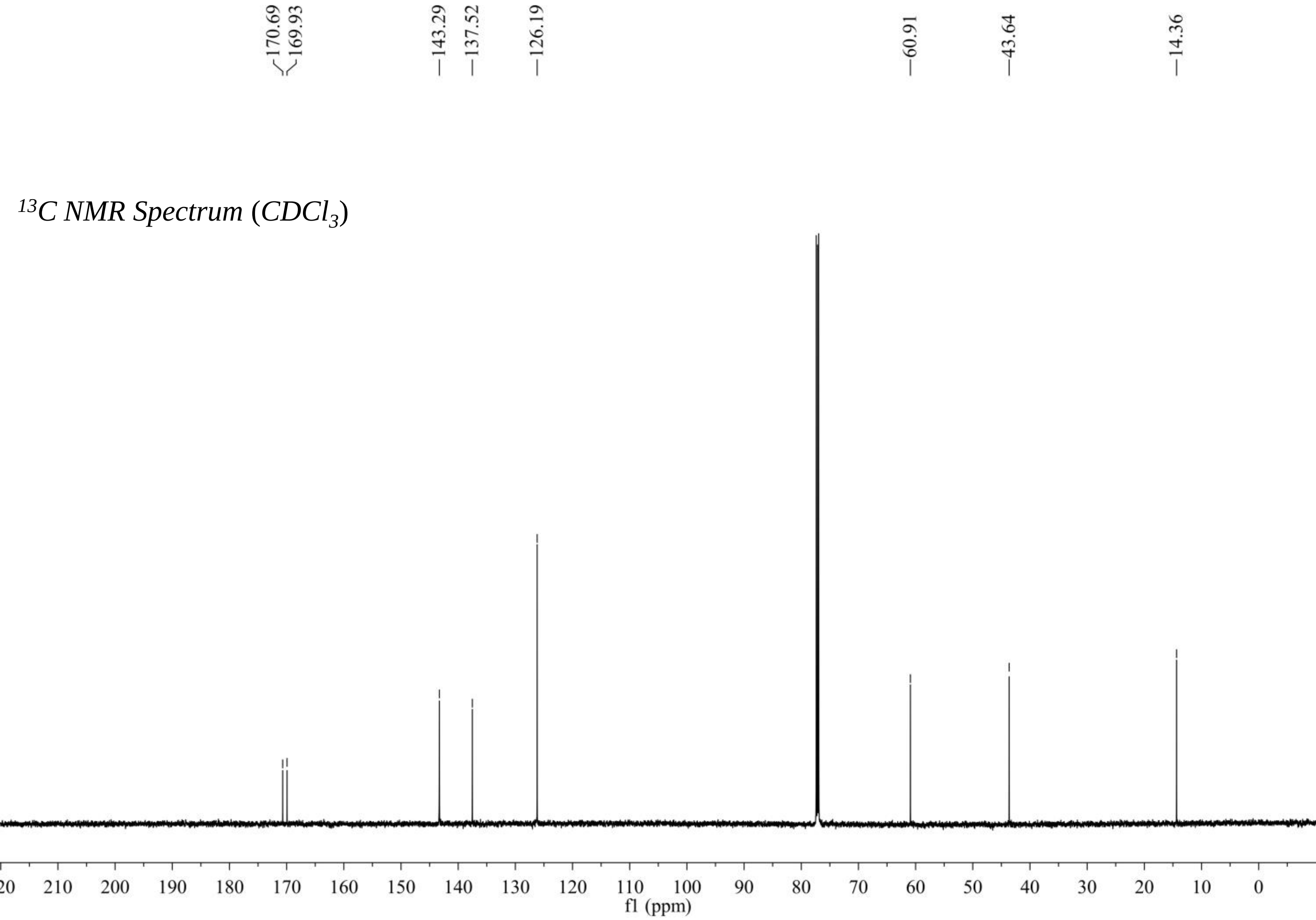

(Ethoxycarbonyl)(pyridin-1-ium-1- yl)amide (**15**)

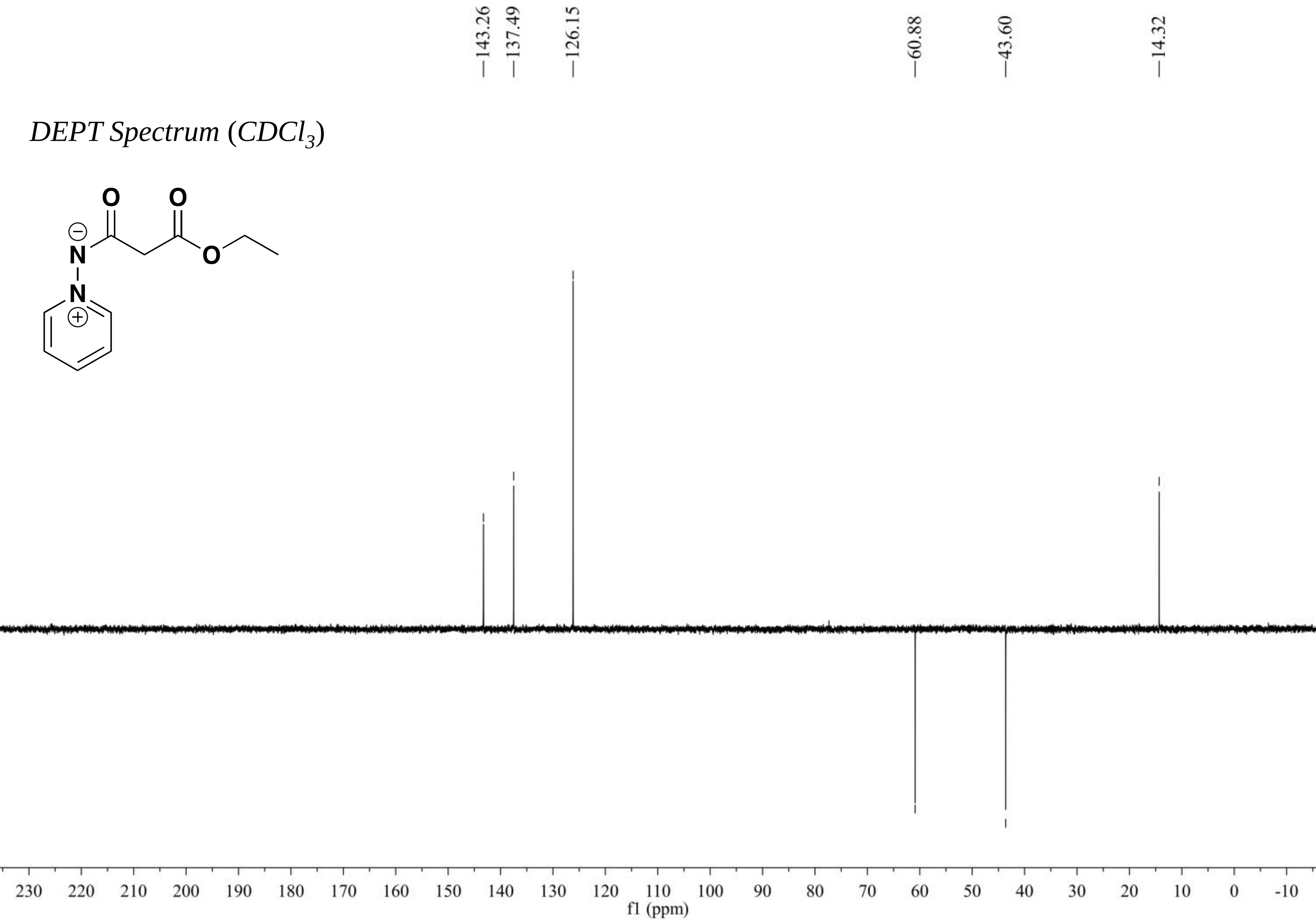

Ethyl 2-methoxypyrazolo[1,5-a]pyridine-3-carboxylate (**2a**)

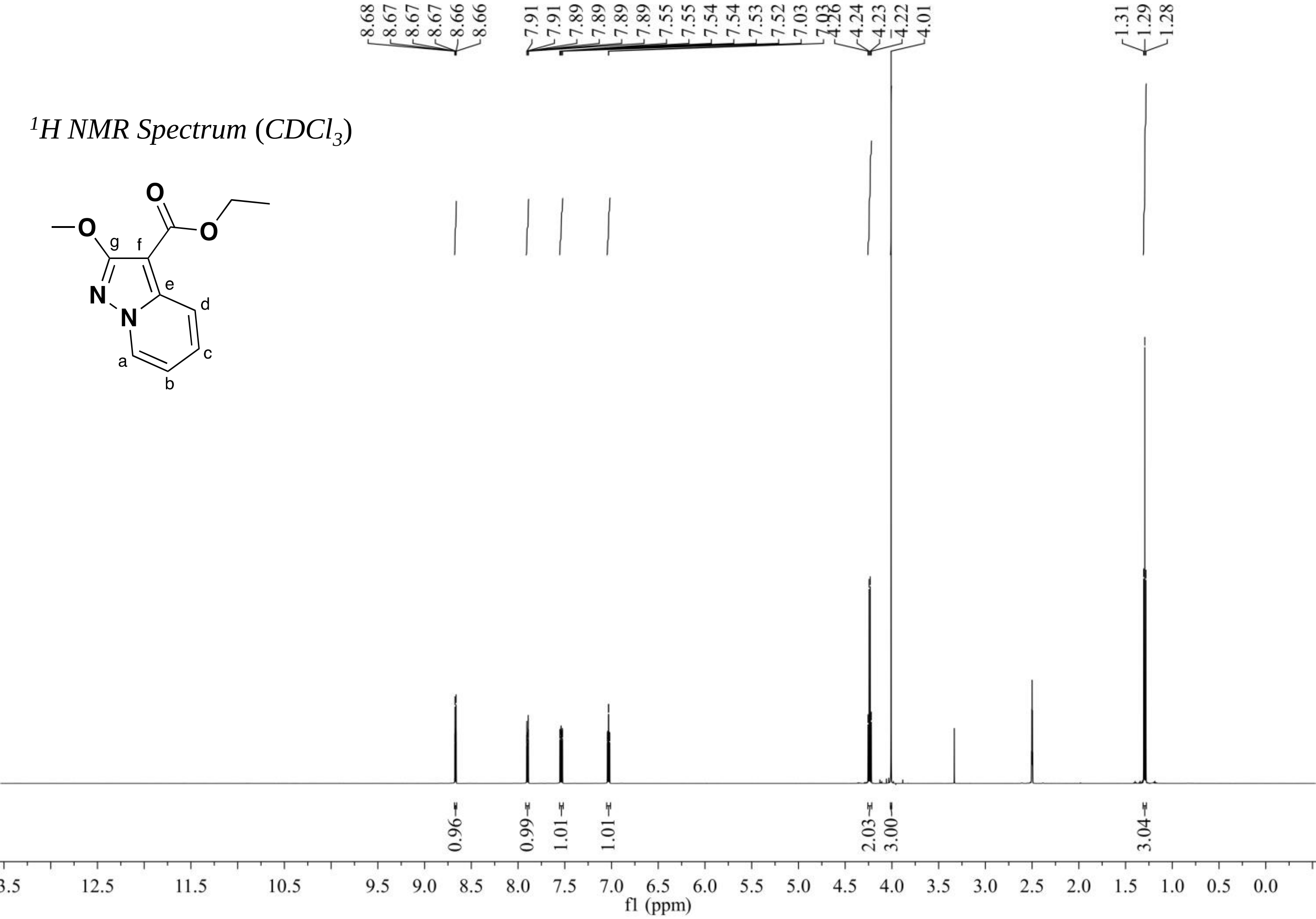

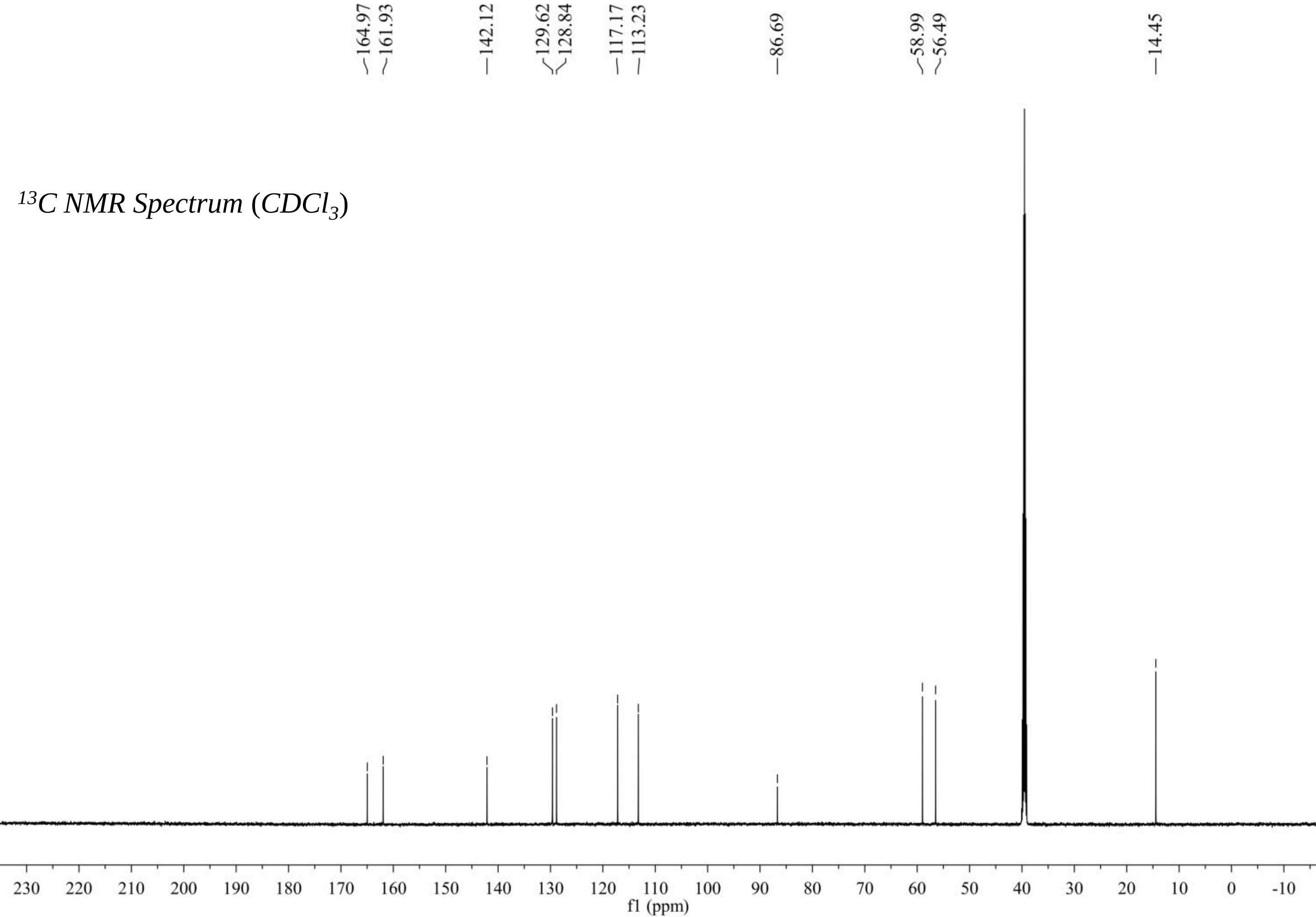

Ethyl 1-methyl-2-oxo-1,2-dihydropyrazolo[1,5-a]pyridine-3-carboxylate (**2b**)

^{1}H NMR Spectrum (*$CDCl_3$*)

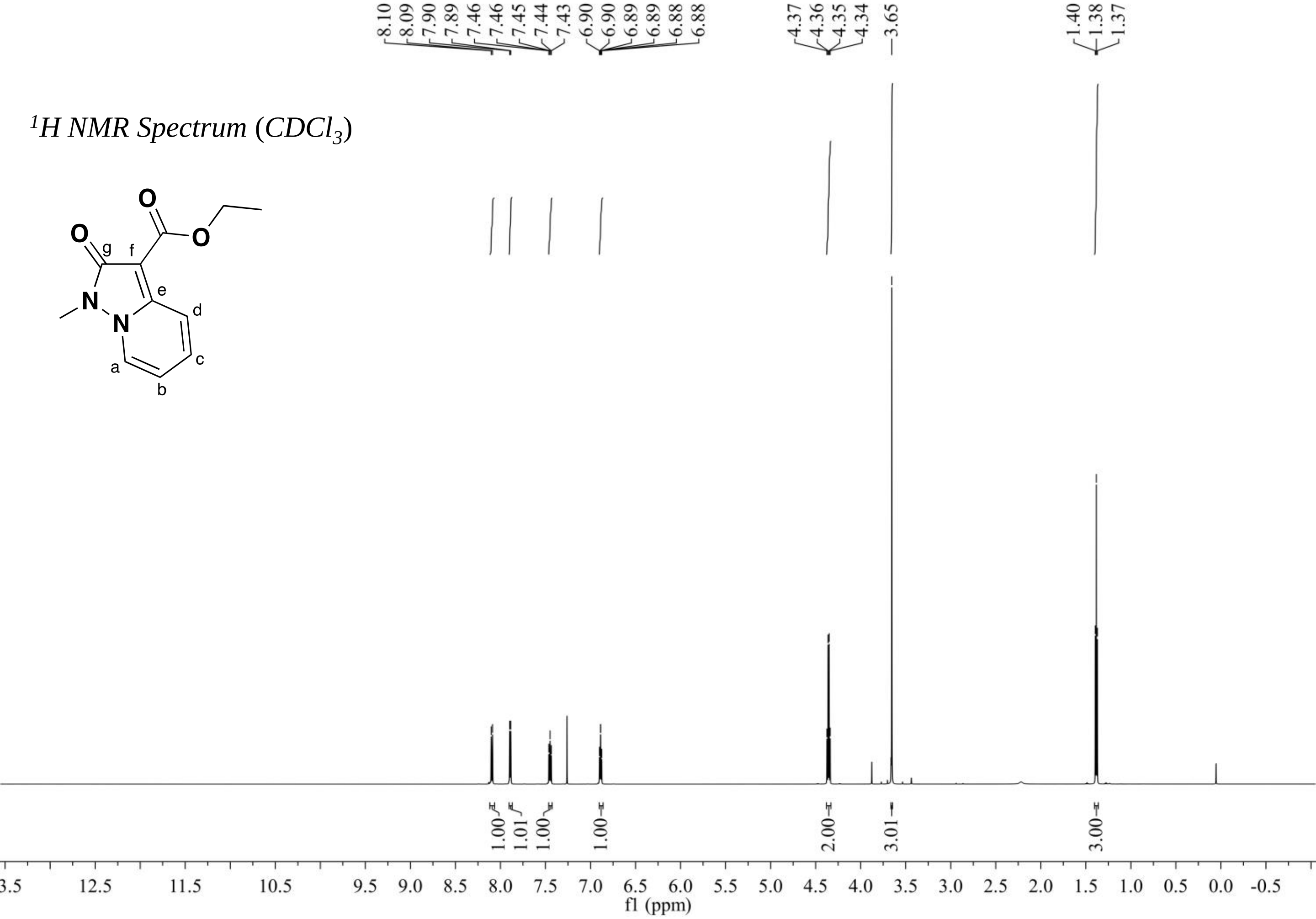

^{13}C NMR Spectrum (*$CDCl_3$*)

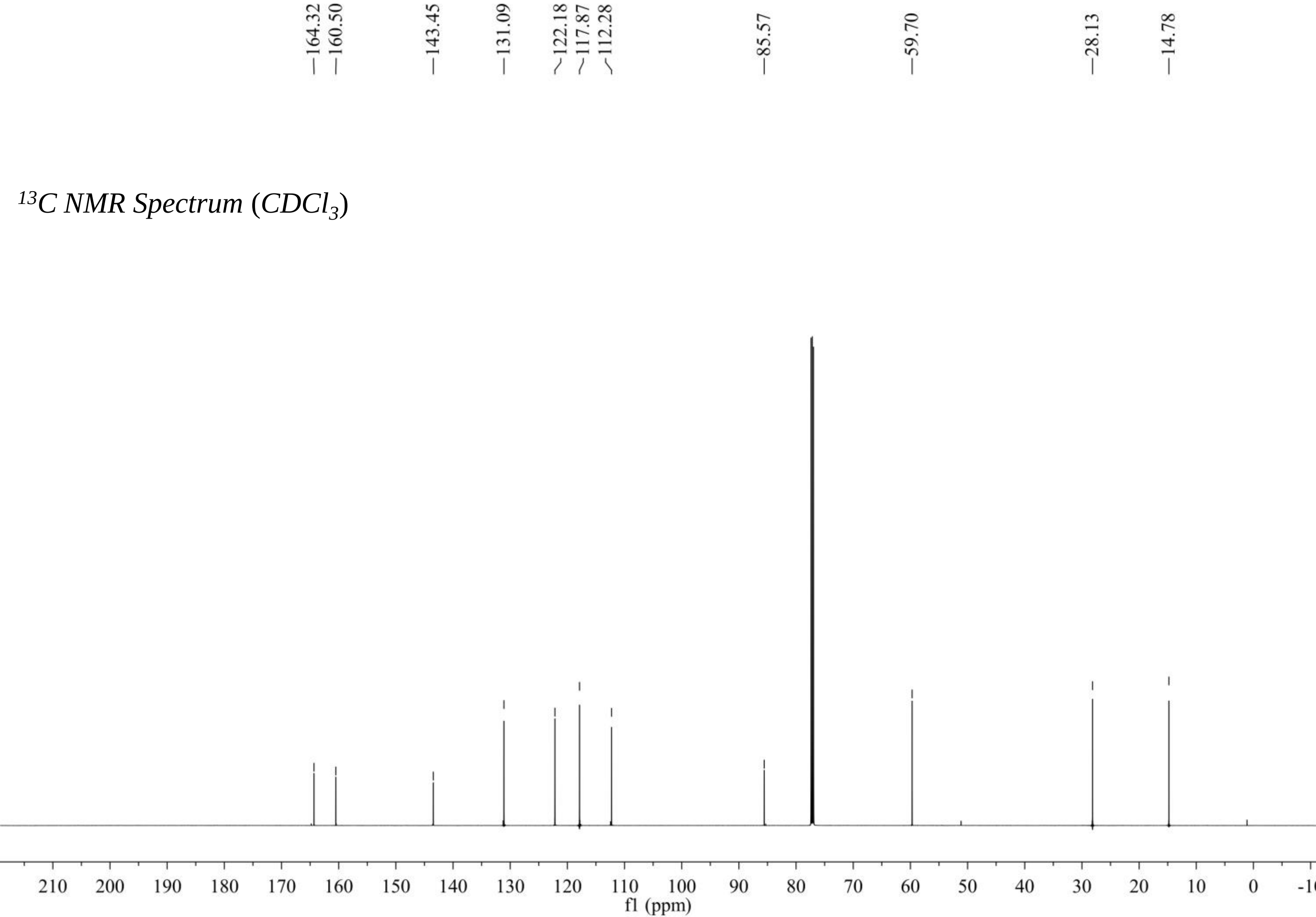

Pyrazolo[1,5-a]pyridin-2-ol (**1**)

^{1}*H NMR Spectrum* (*DMSO*)

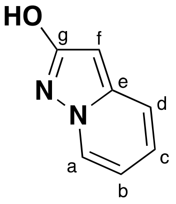

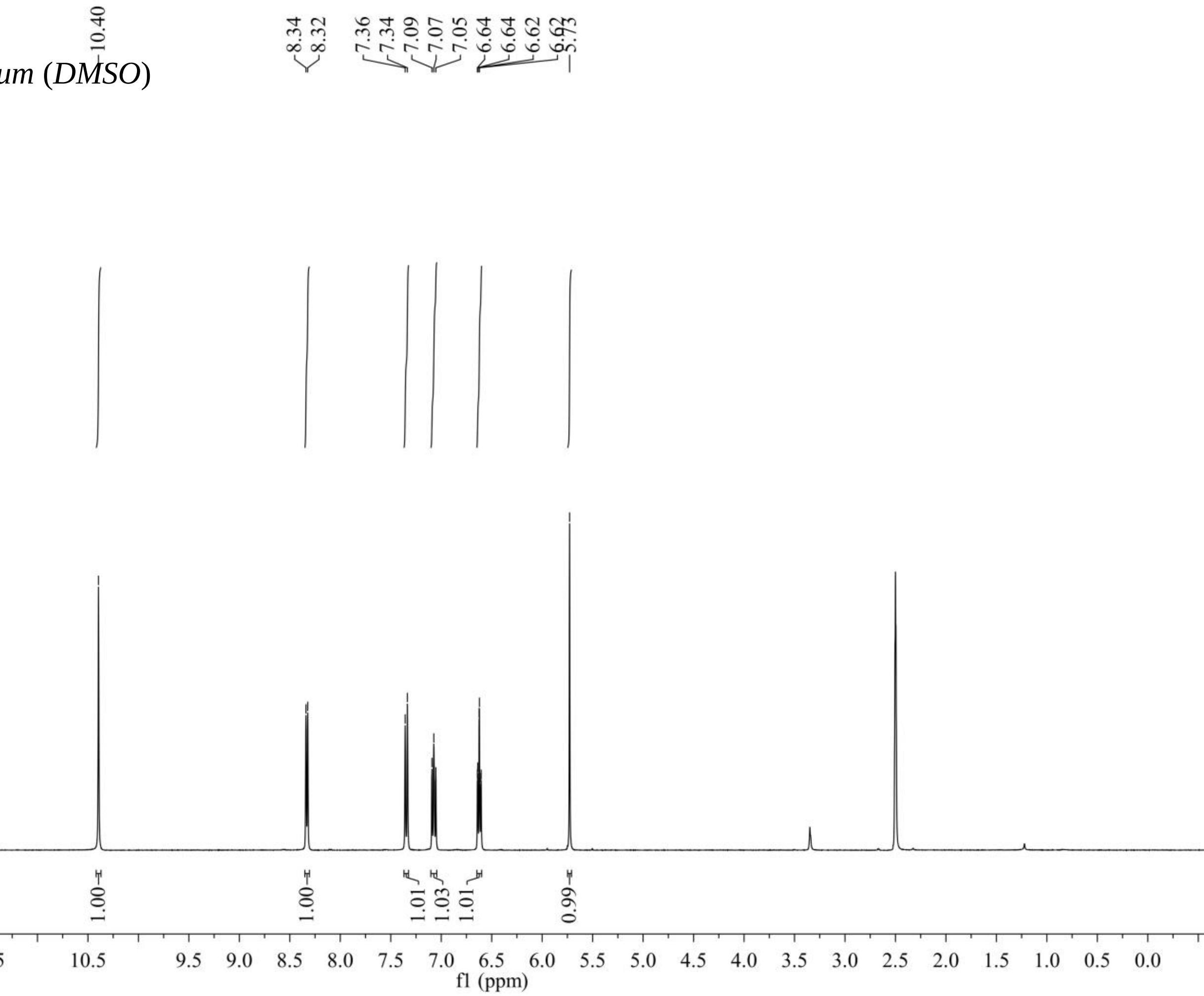

^{13}*C NMR Spectrum* (*DMSO*)

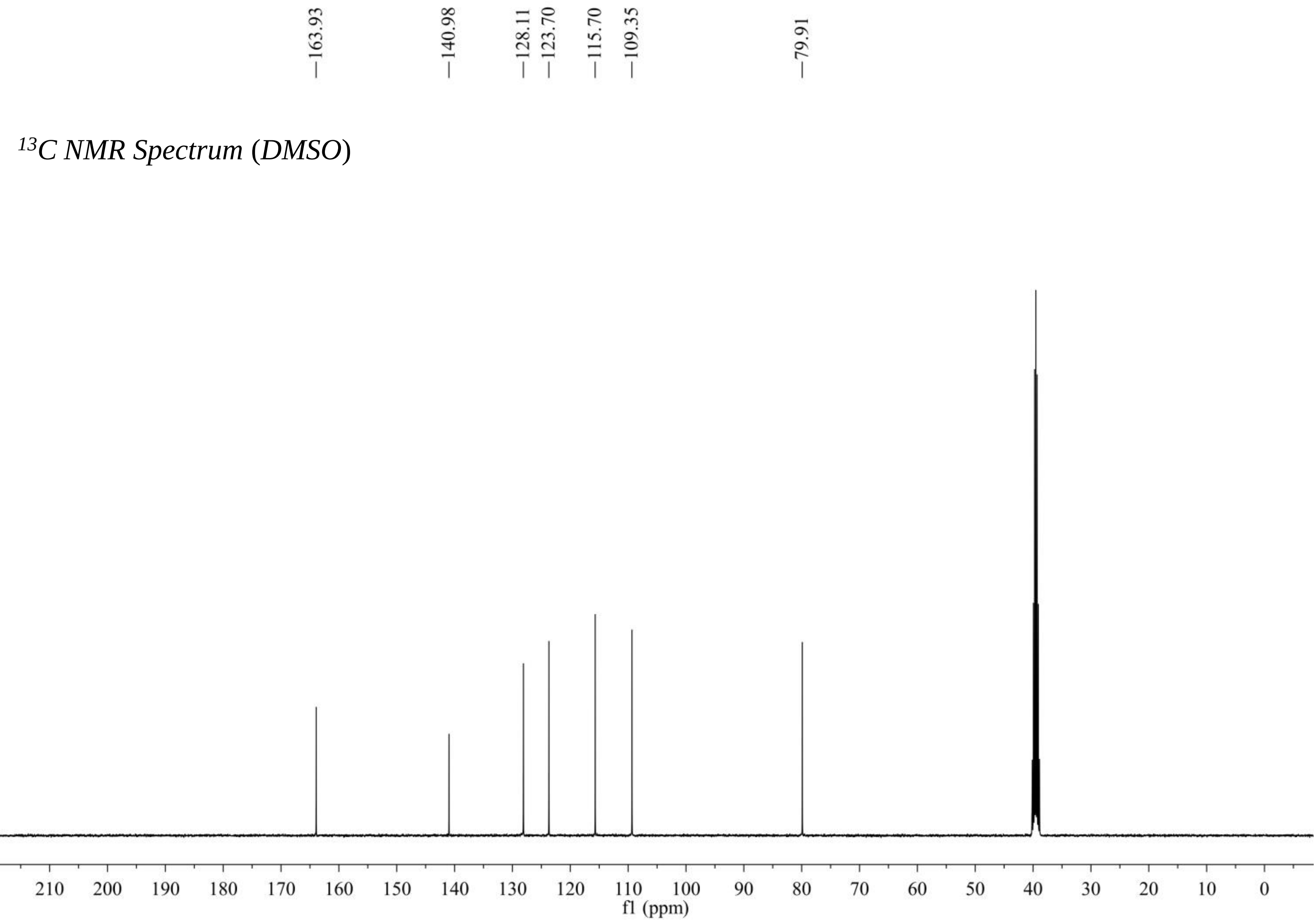

2-*Methoxypyrazolo[1,5-a]pyridine* (**1a**)

^{1}H NMR Spectrum (*CDCl$_3$*)

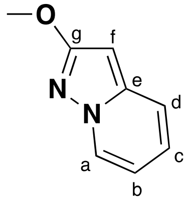

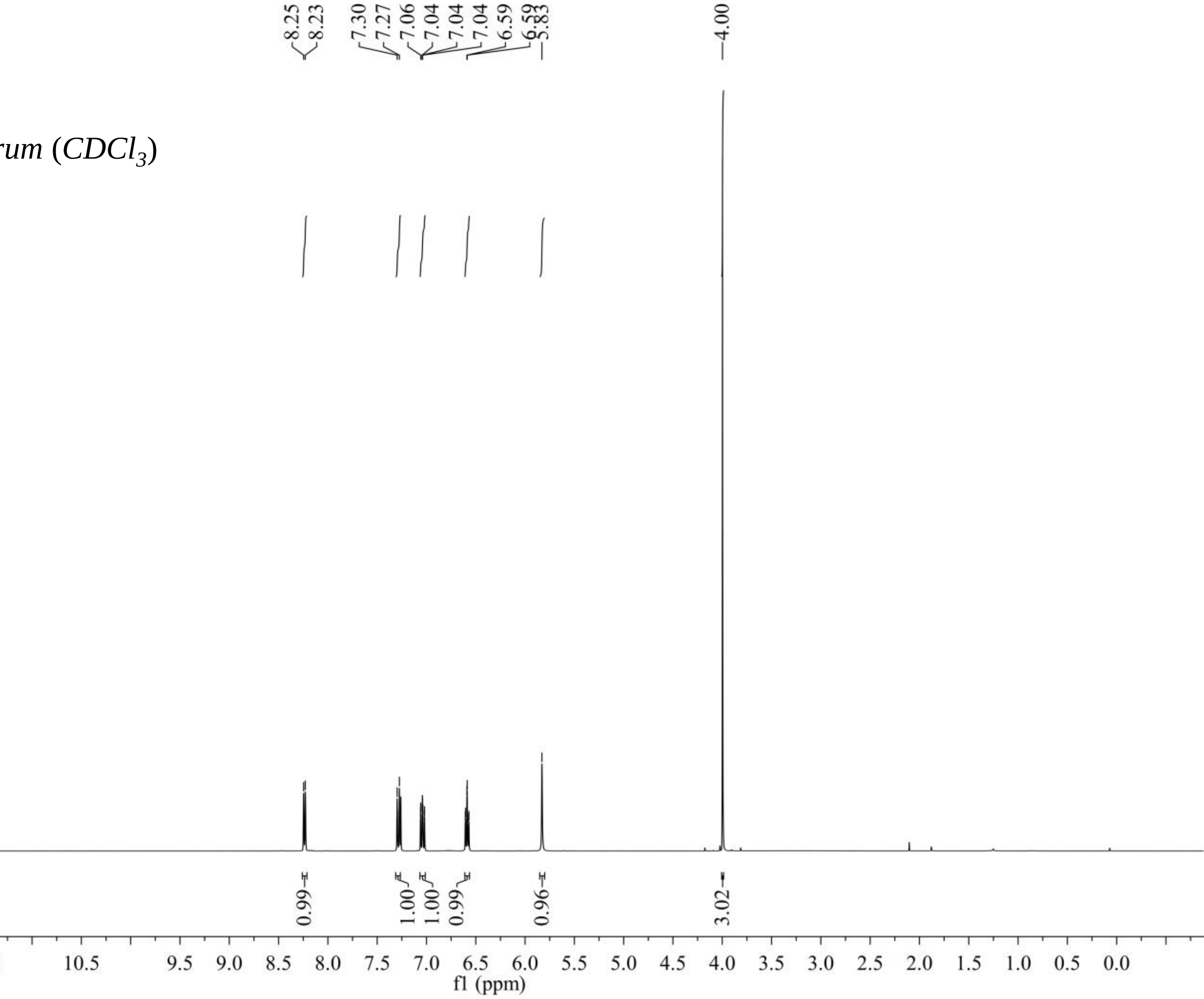

^{13}C NMR Spectrum (*CDCl$_3$*)

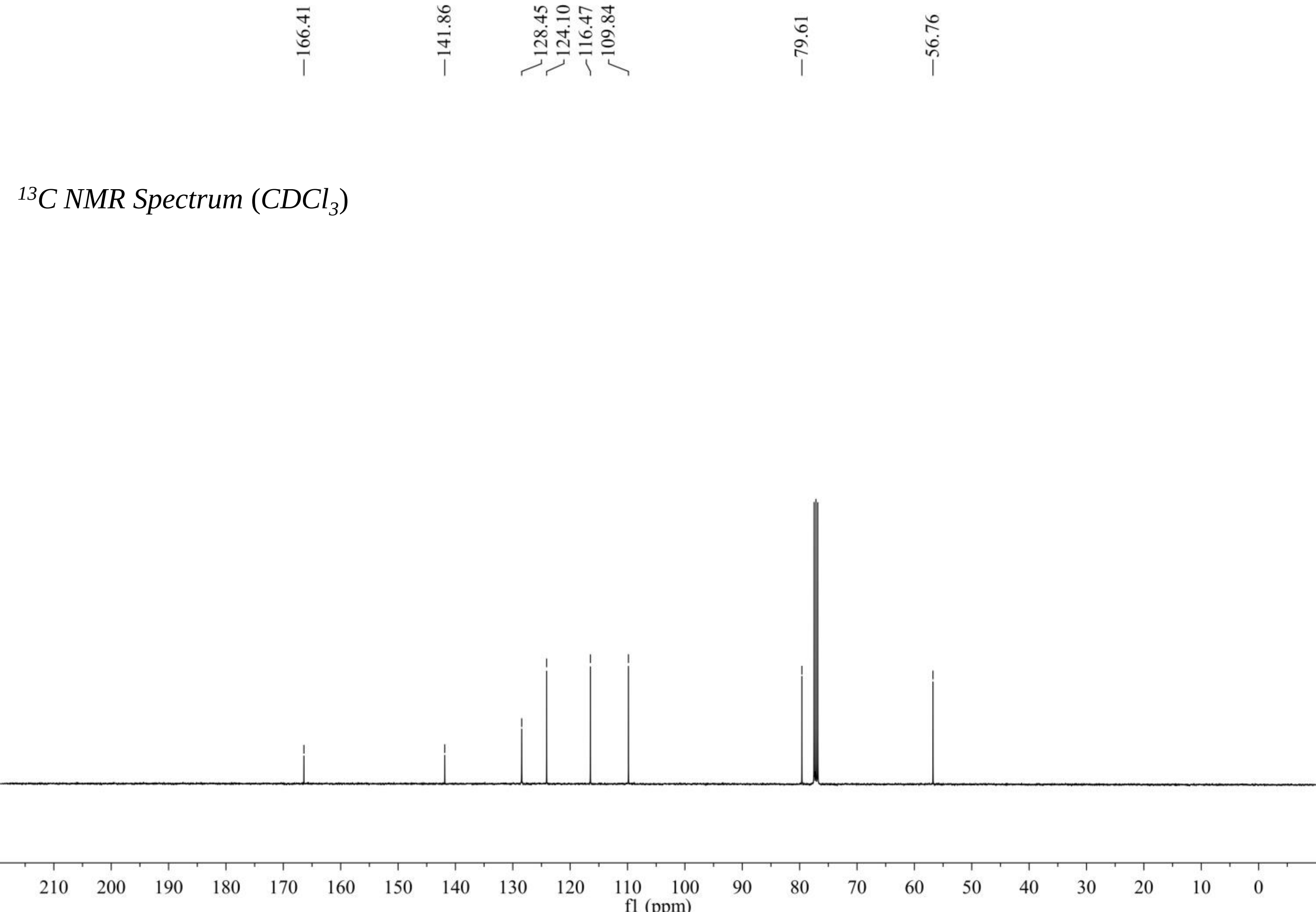

1-Methylpyrazolo[1,5-a]pyridin-2(1H)-one (**1b**)

^{1}H NMR Spectrum (*DMSO*)

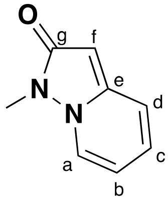

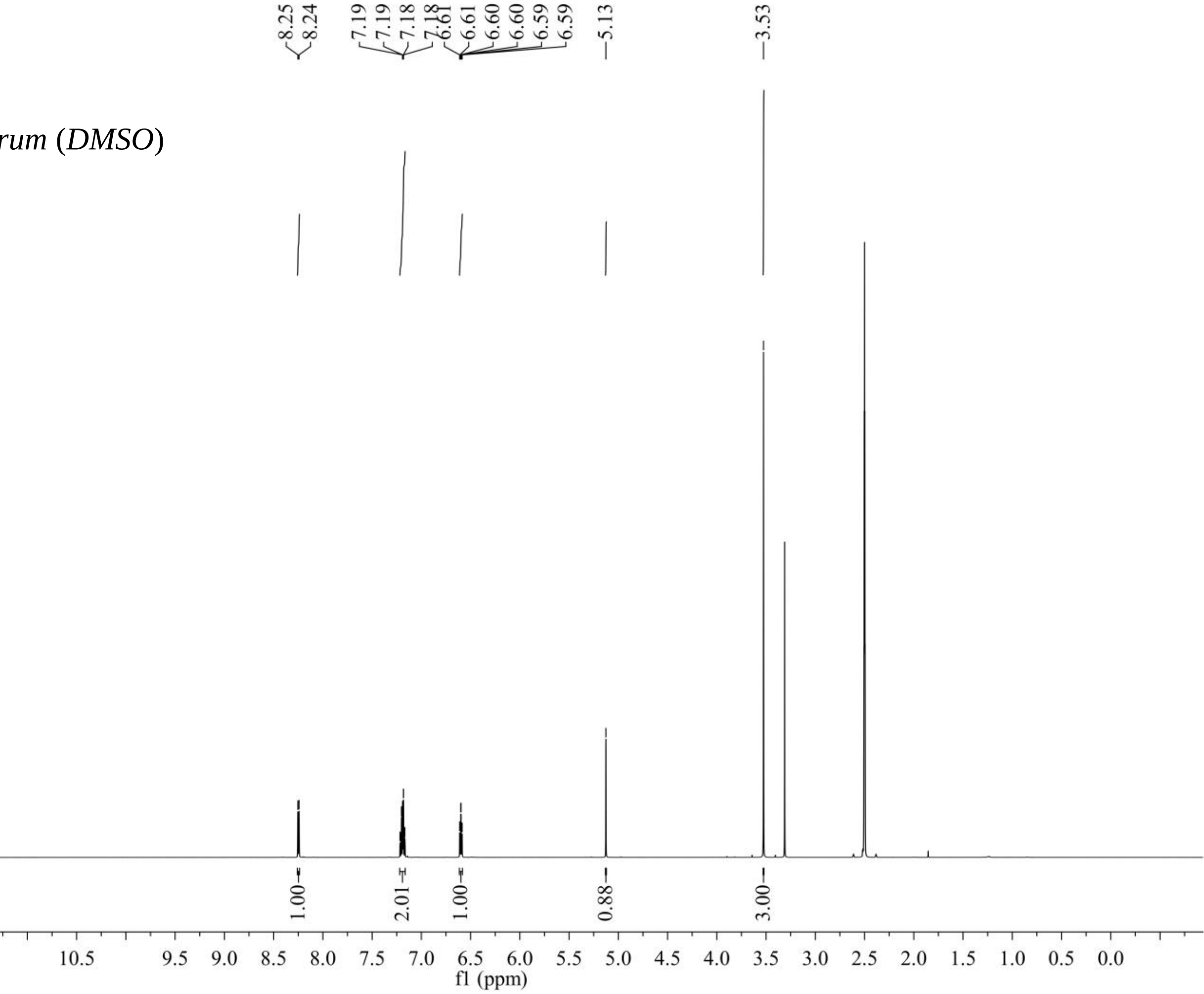

^{13}C NMR Spectrum (*DMSO*)

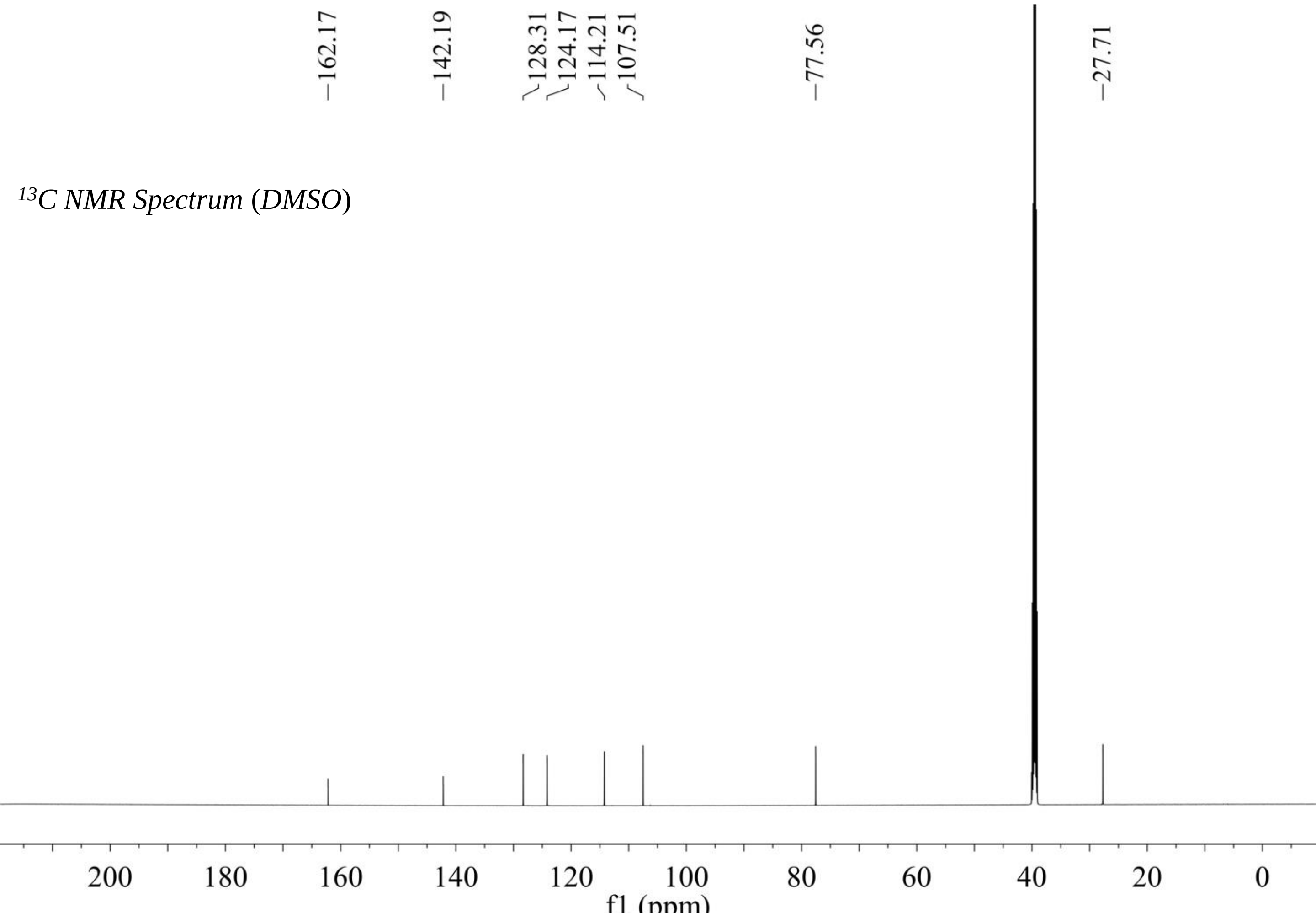

2-(Benzyloxy)pyrazolo[1,5-a]pyridine (**1c**)

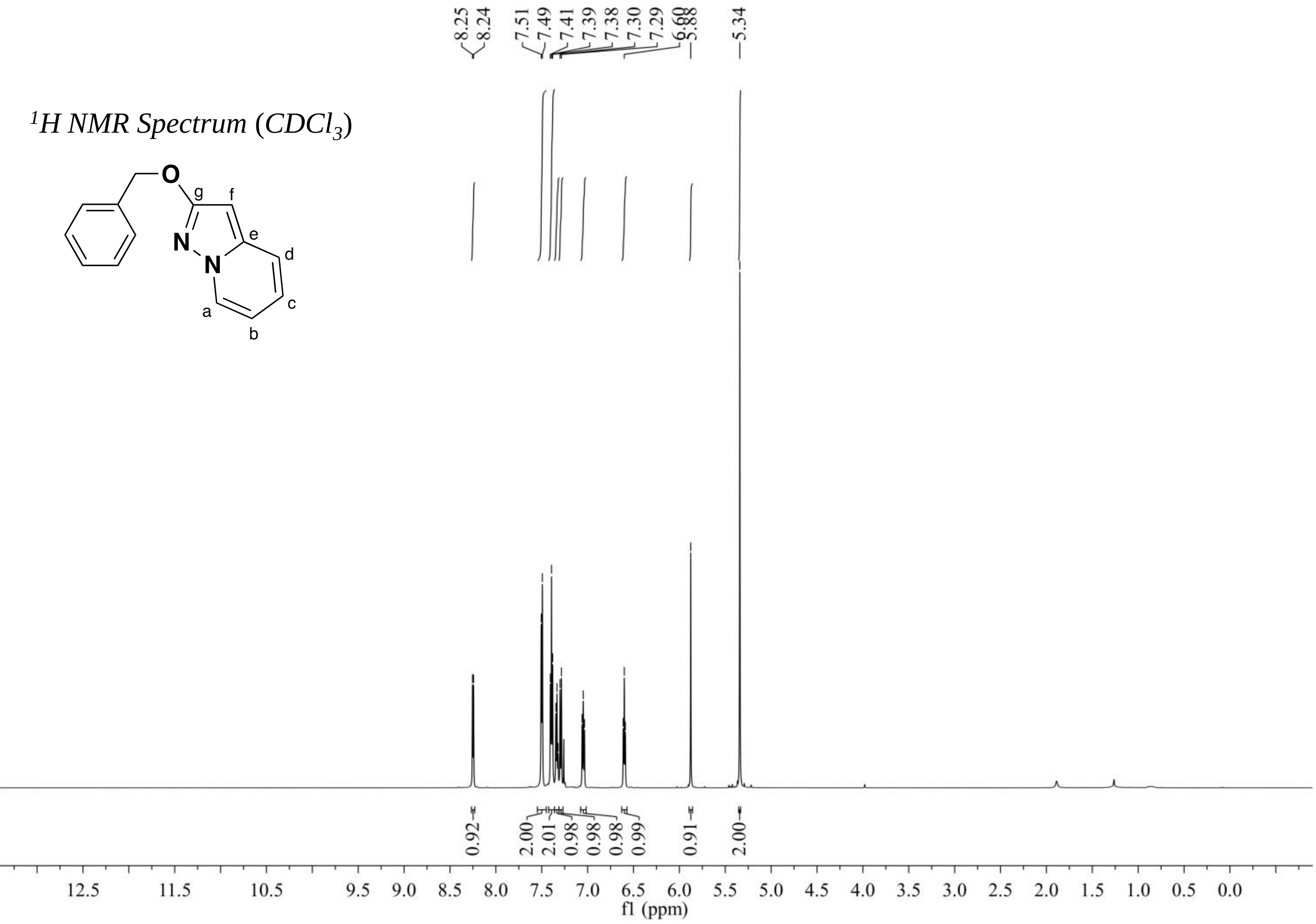

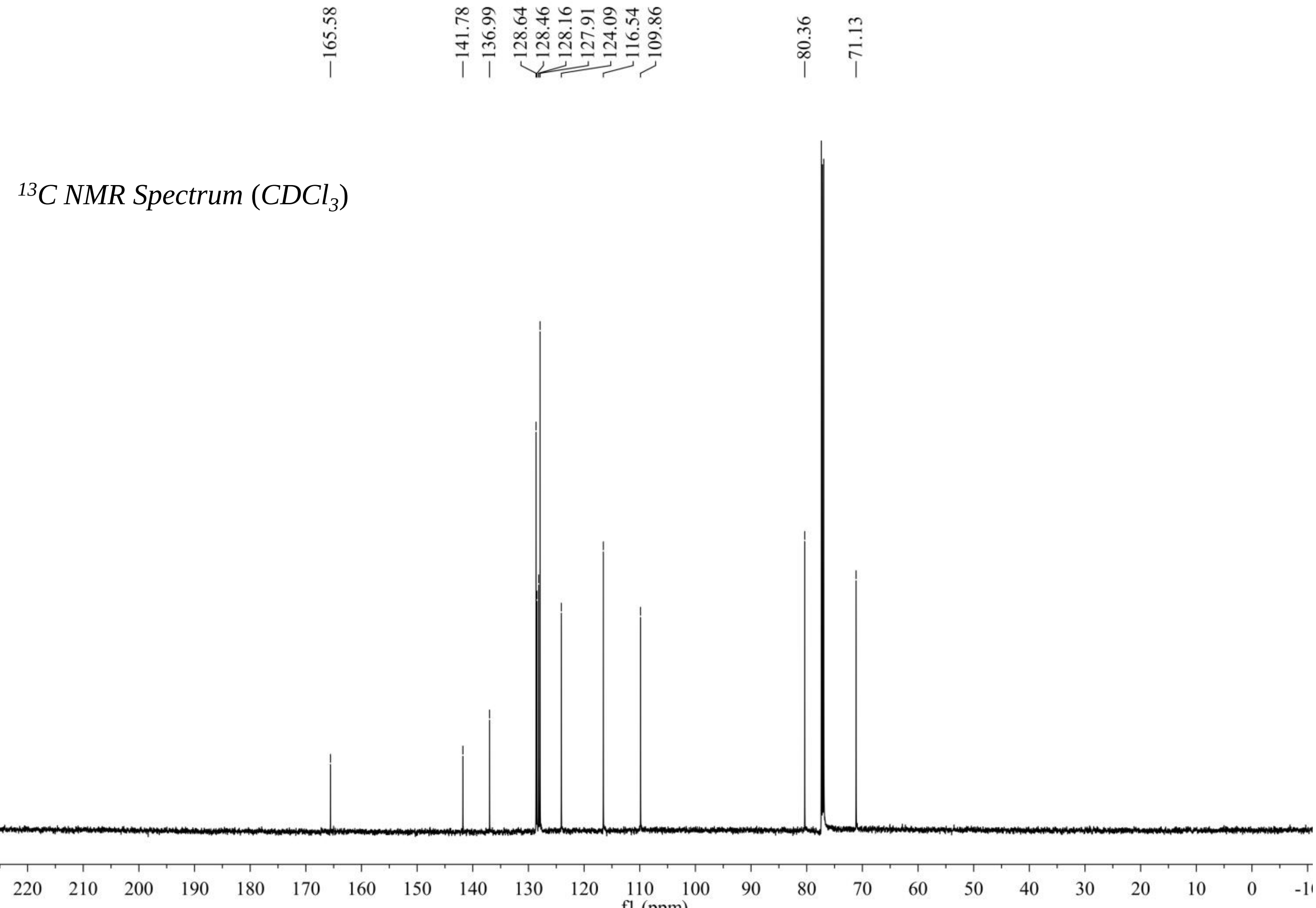

3-Nitrosopyrazolo[1,5-a]pyridin-2-ol (**16**)

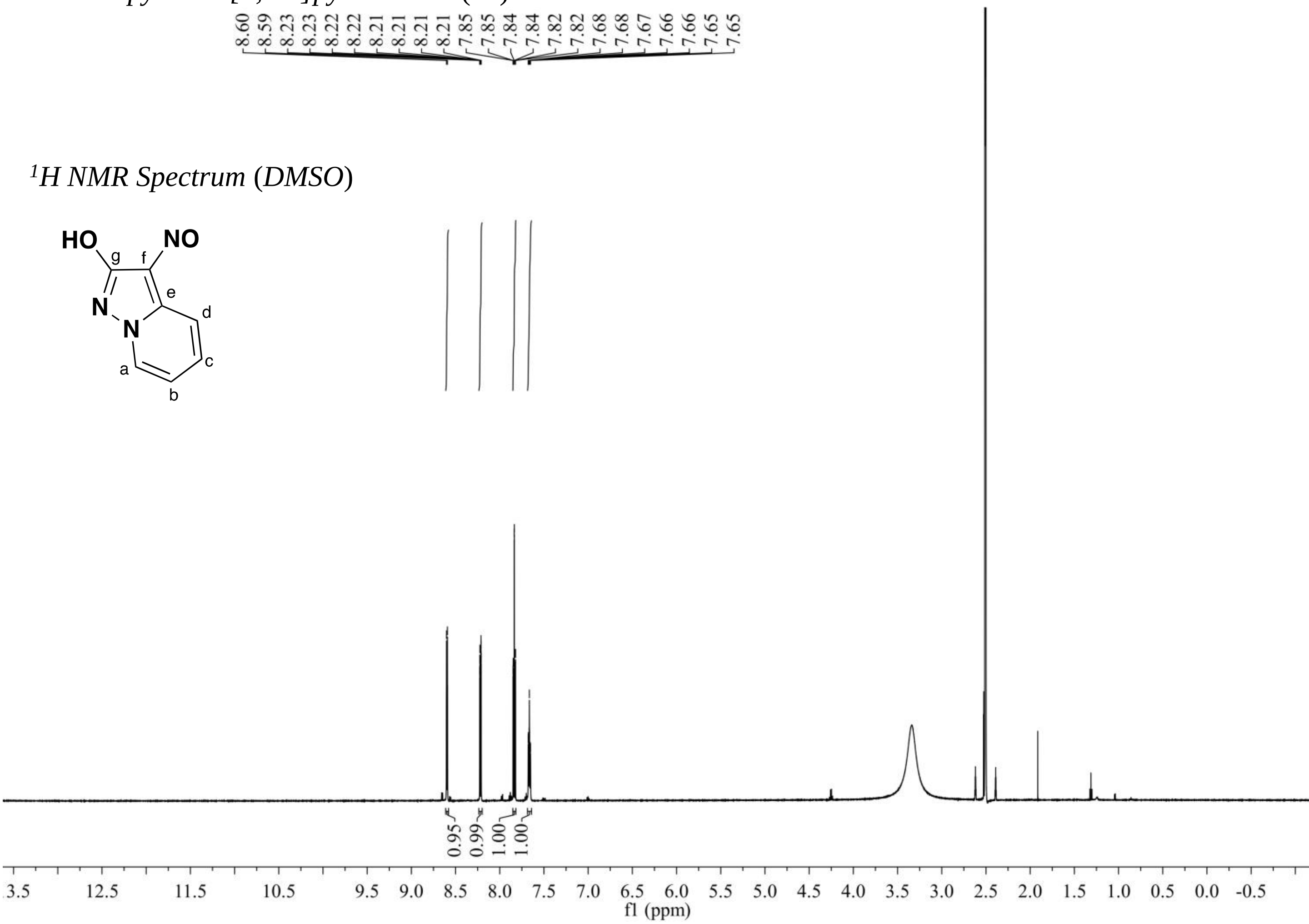

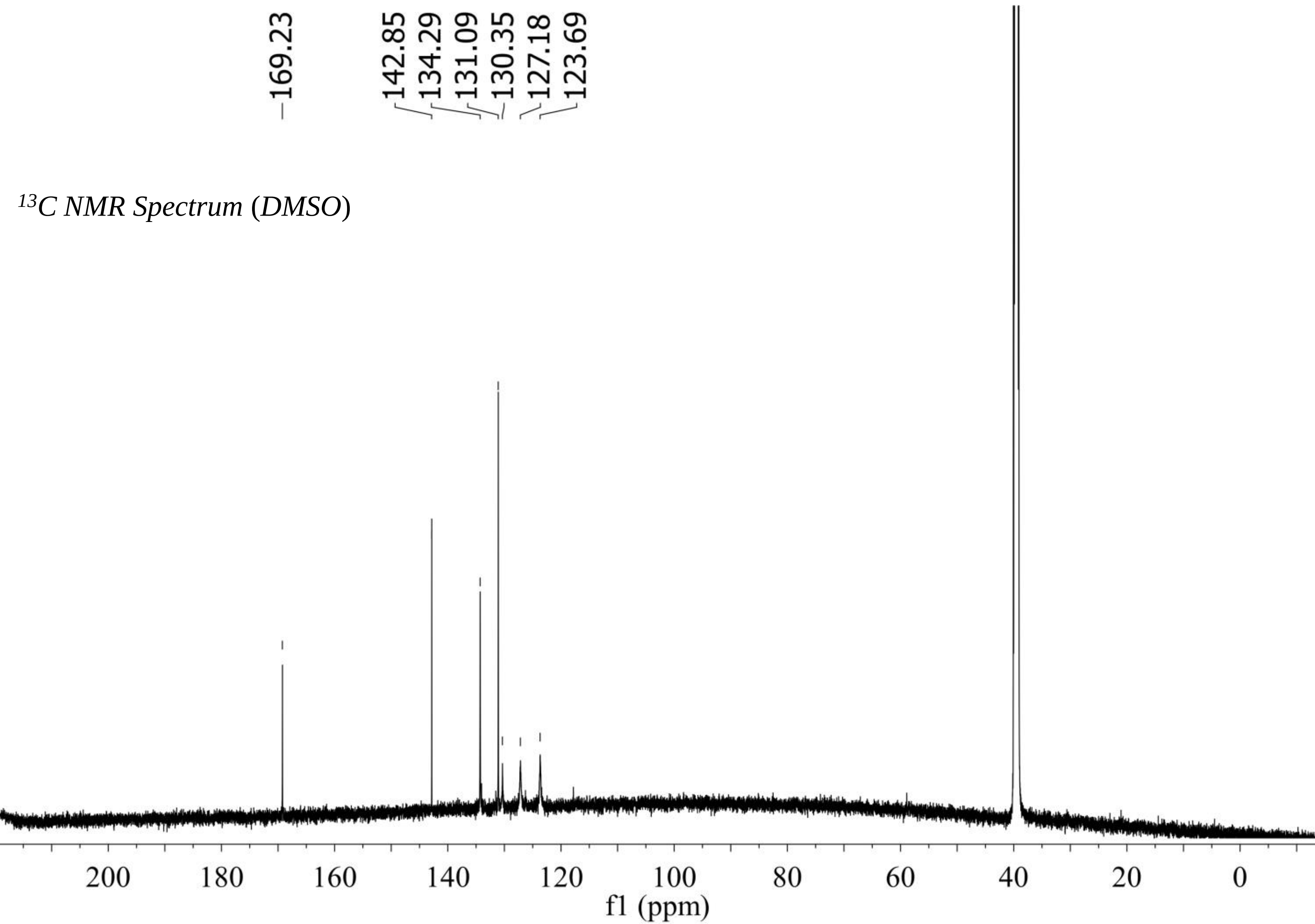

3-Nitropyrazolo[1,5-a]pyridin-2-ol (**3**)

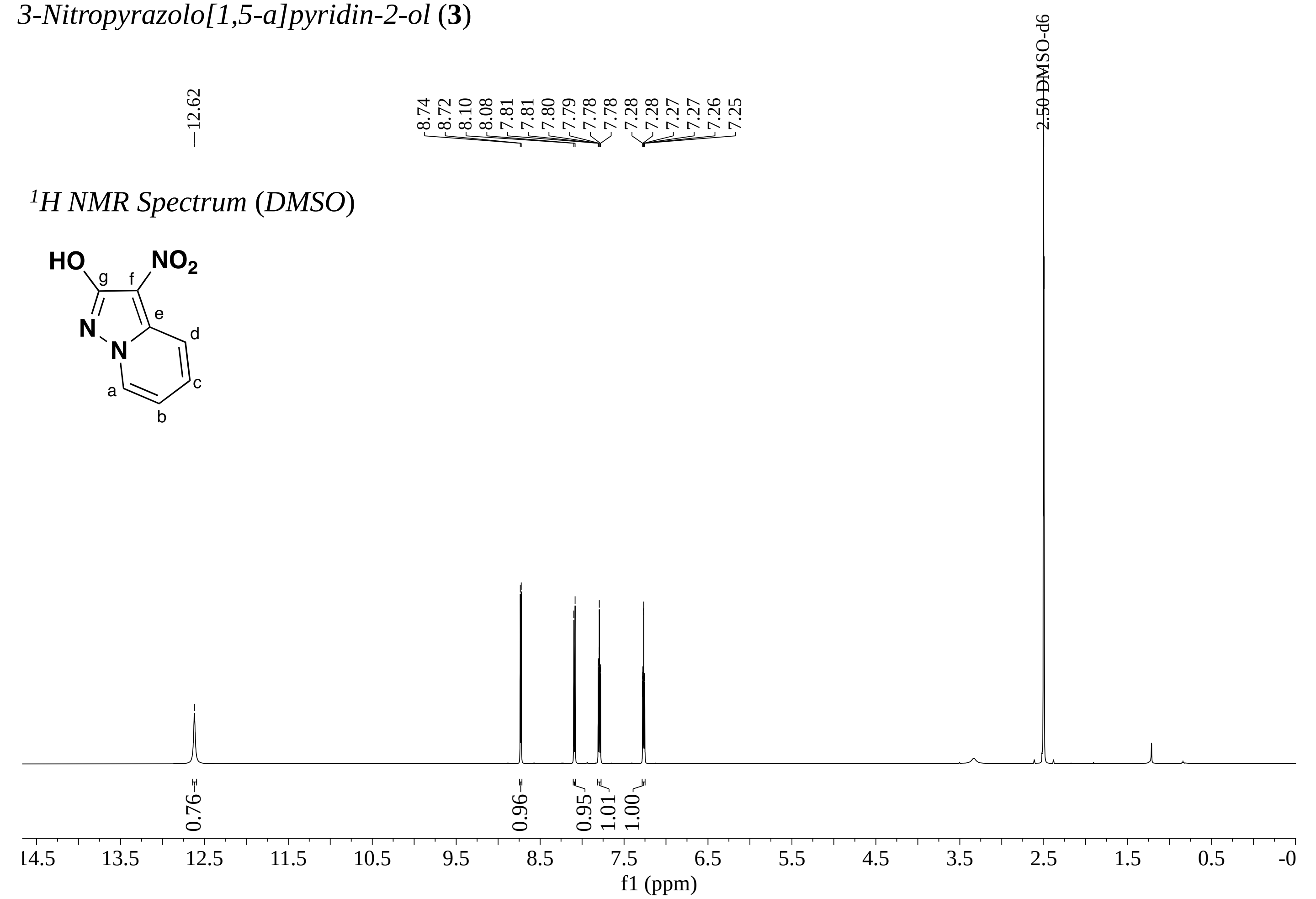

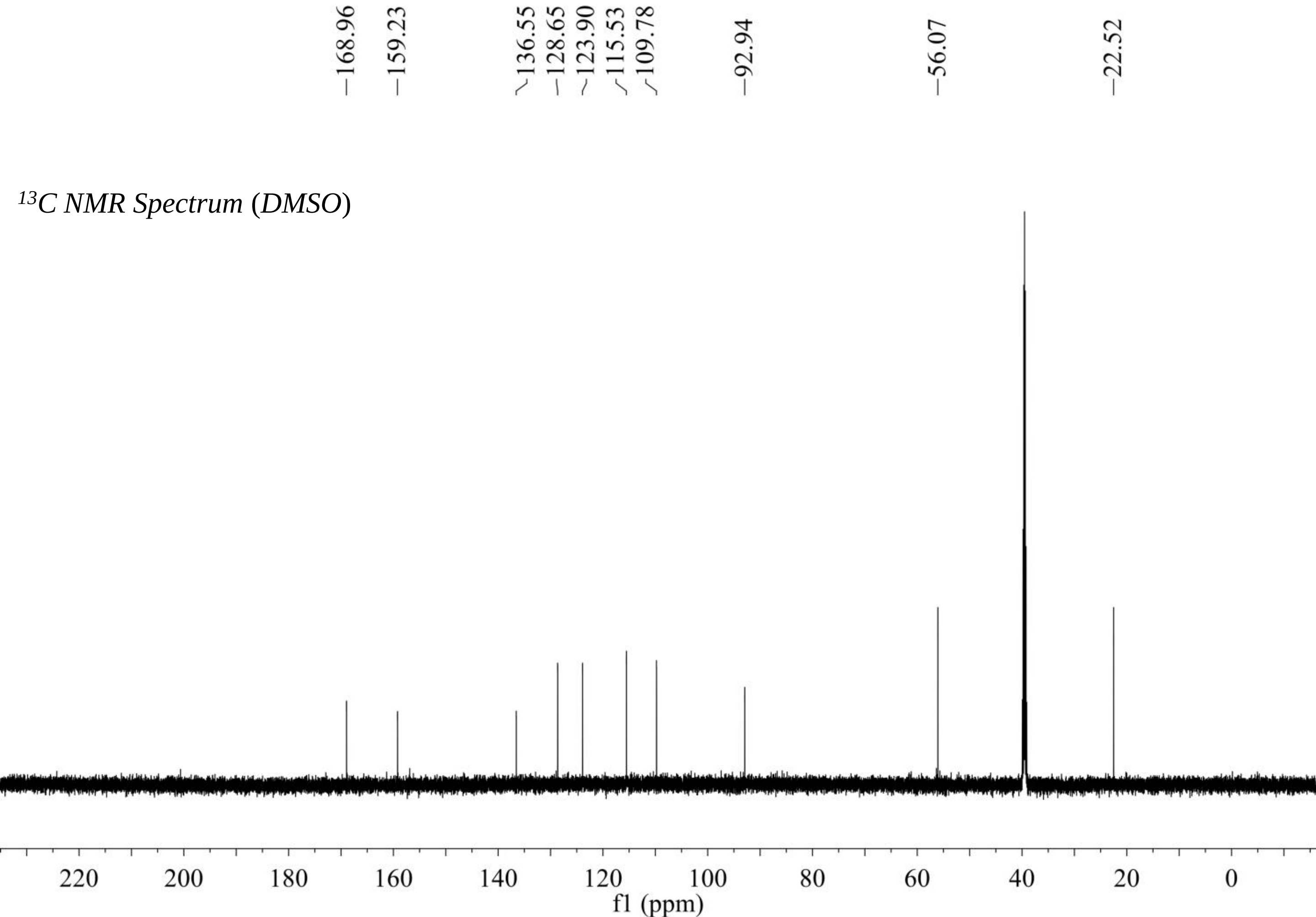

2-Methoxy-3-nitropyrazolo[1,5-a]pyridine (**17a**)

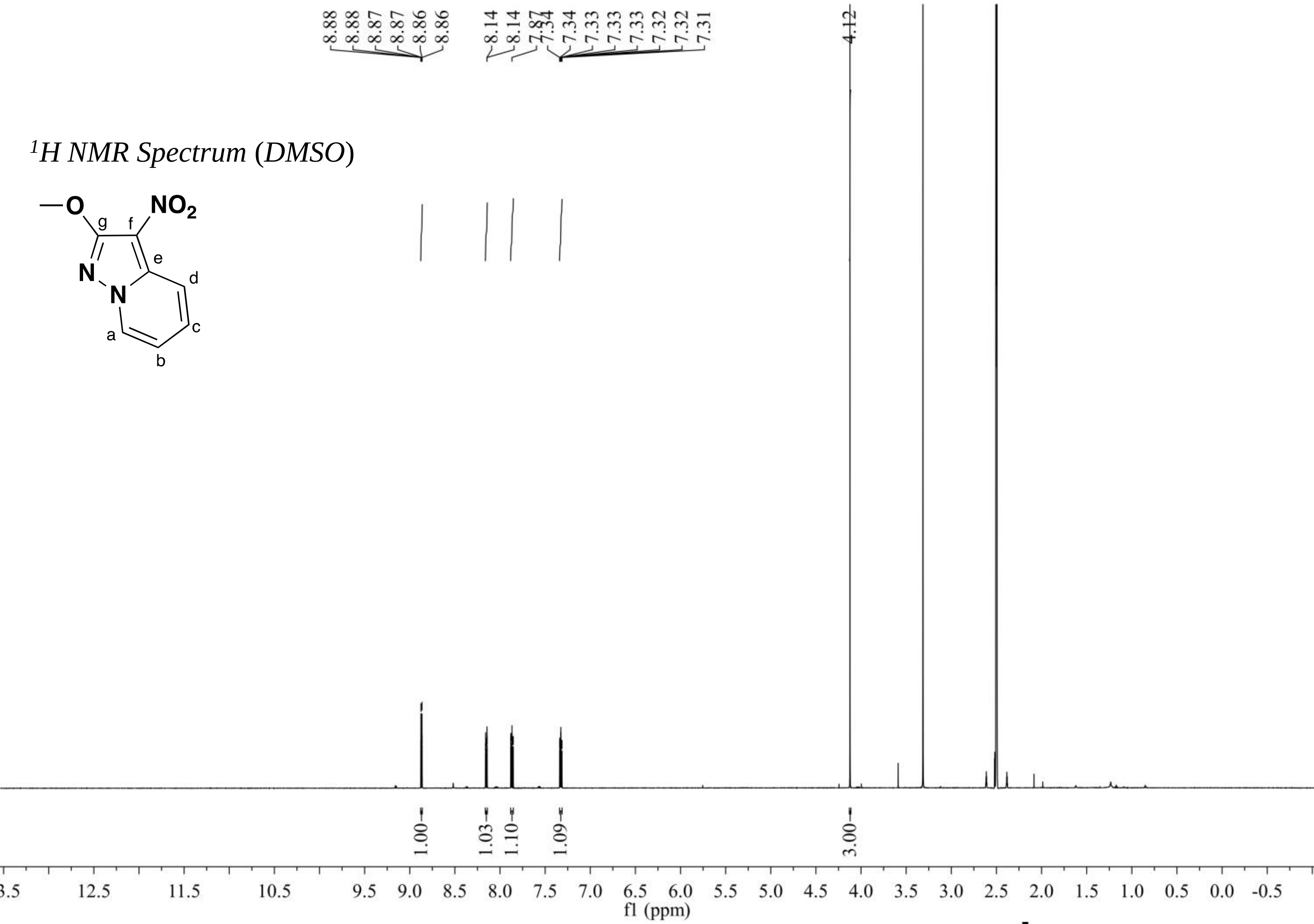

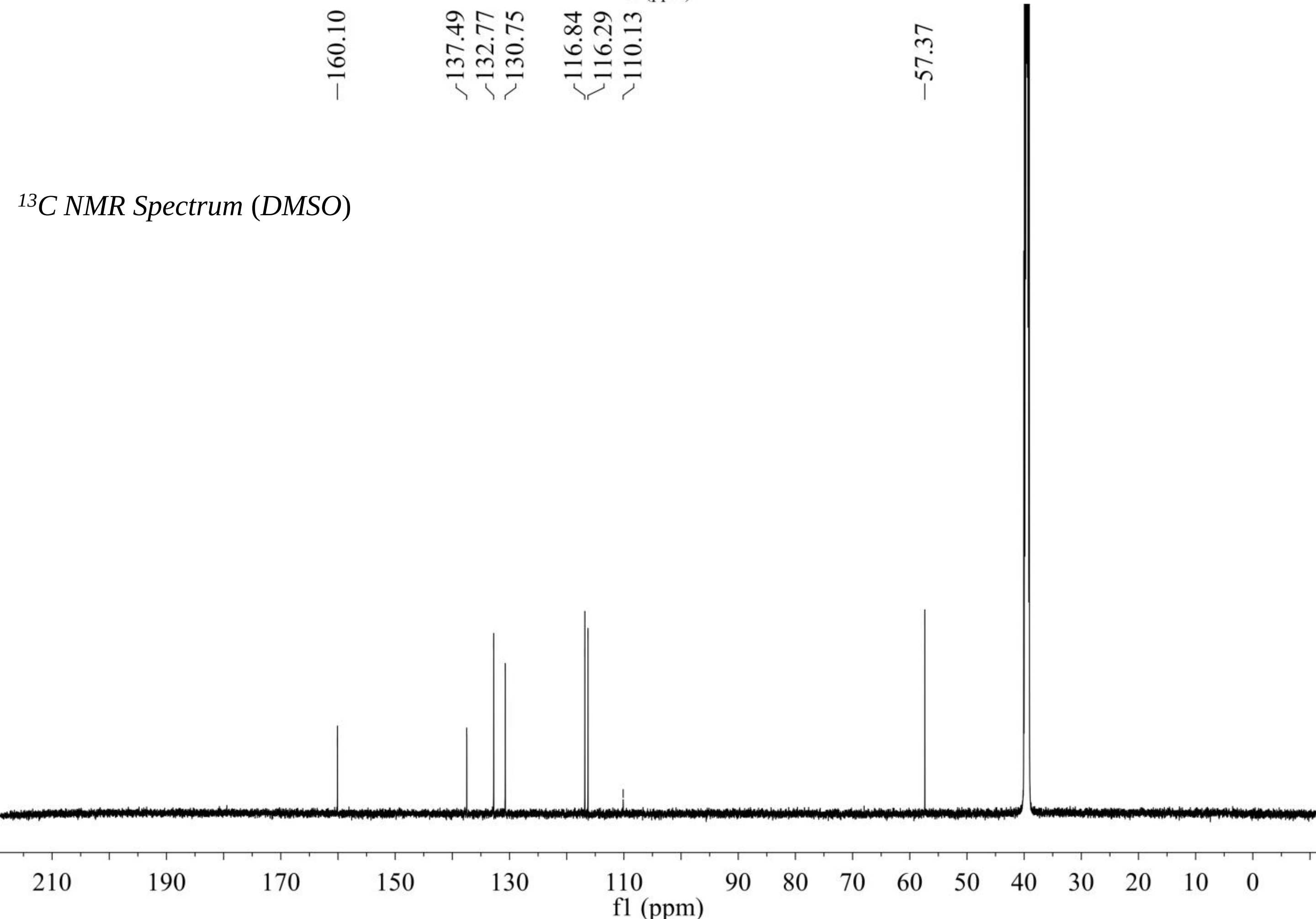

2-Methoxy-3-nitropyrazolo[1,5-a]pyridine (**17a**)

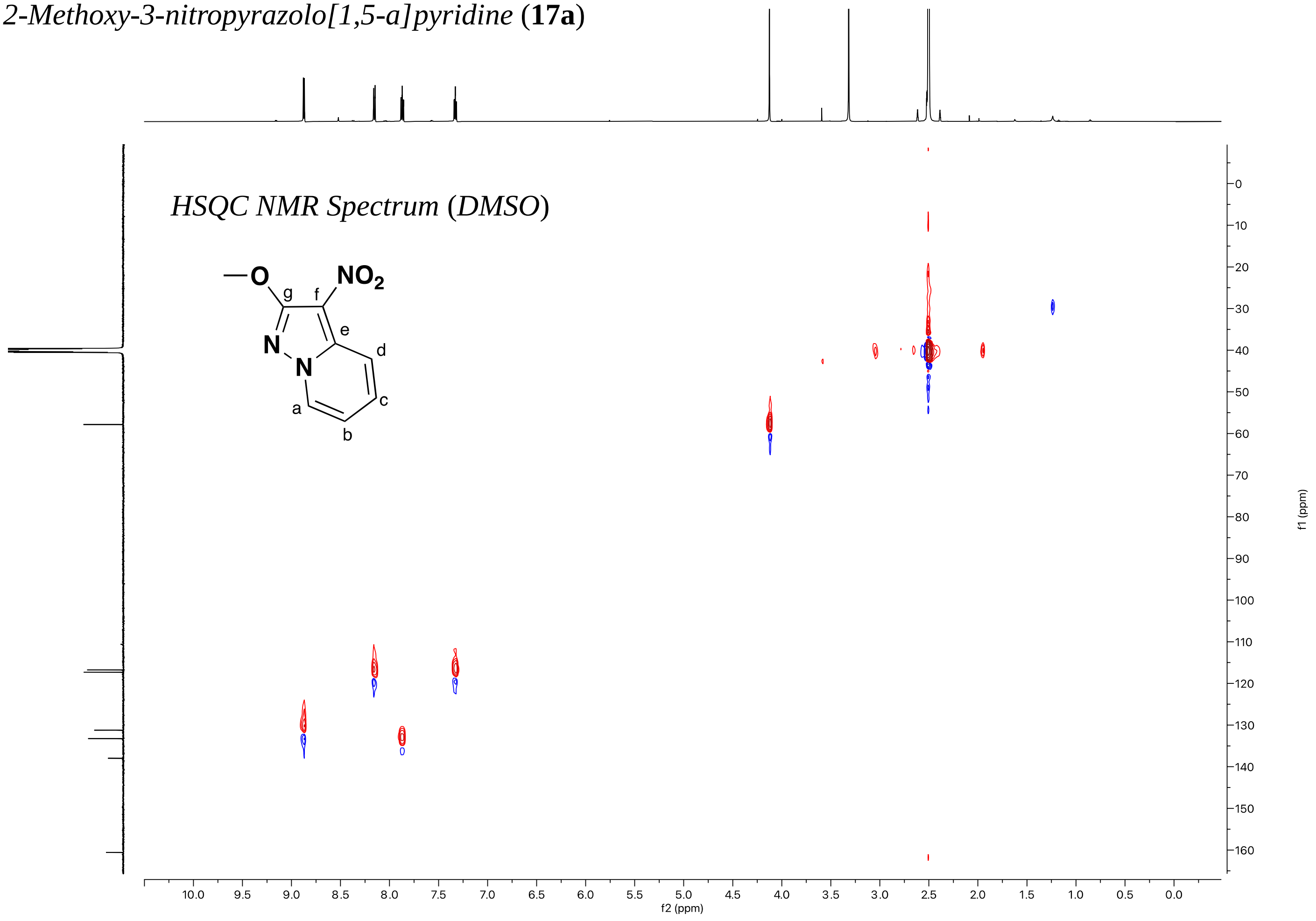

N-(2-Methoxypyrazolo[1,5-a]pyridin-3-yl)acetamide (**3a**)

^{1}H NMR Spectrum (*DMSO*)

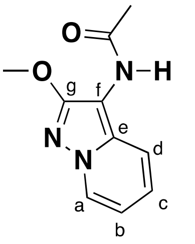

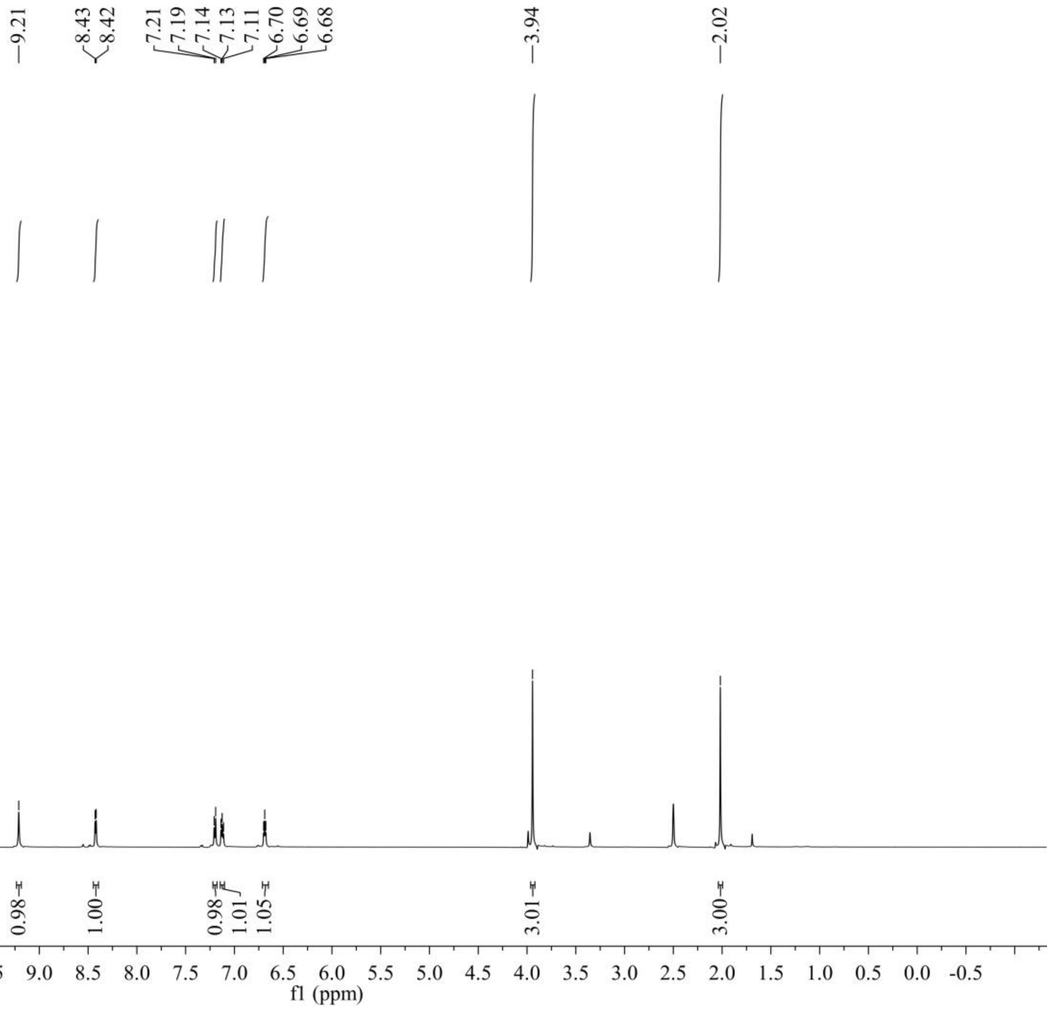

^{13}C NMR Spectrum (*DMSO*)

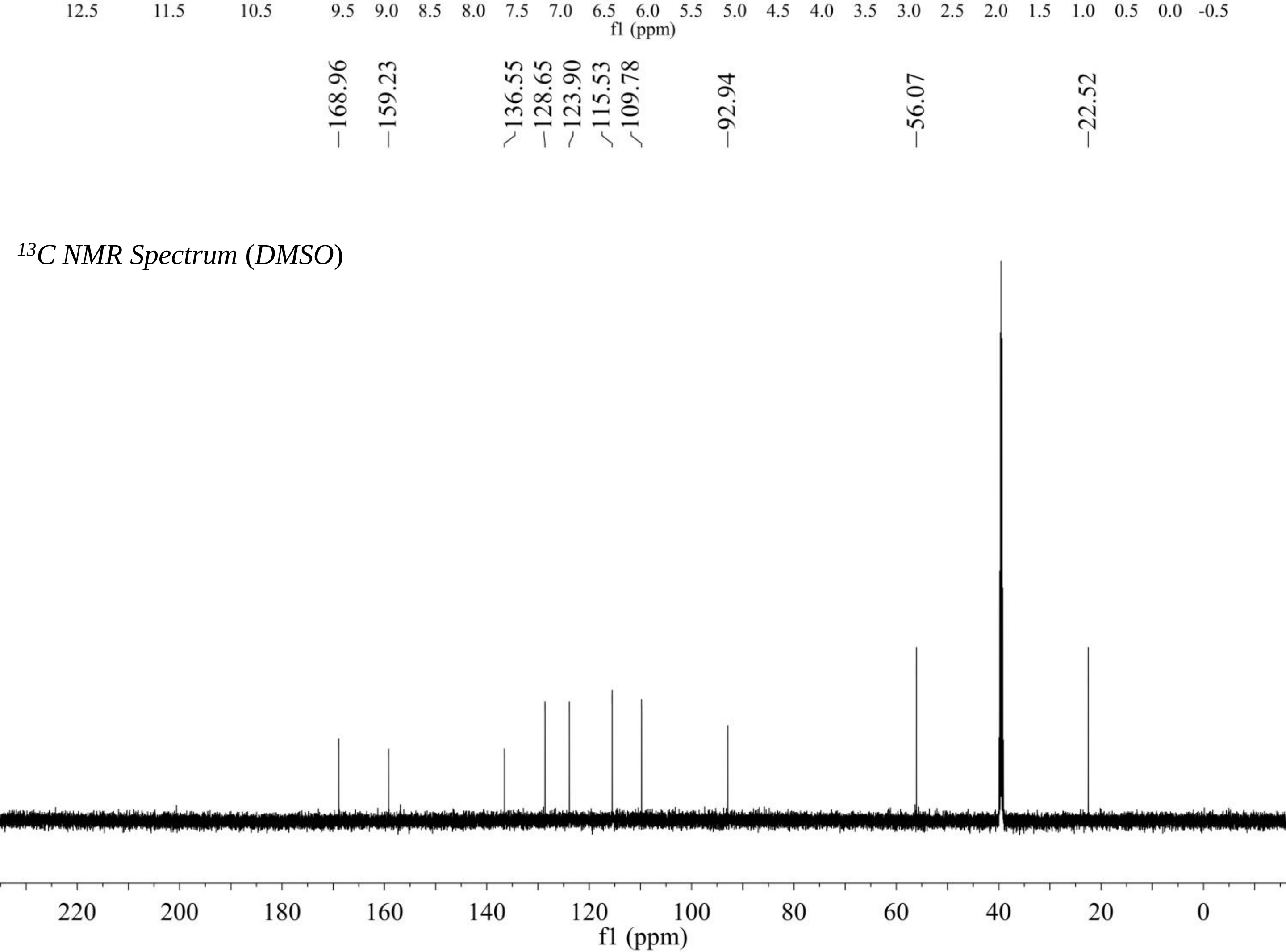

N-(2-Methoxypyrazolo[1,5-a]pyridin-3-yl)methanesulfonamide (**3b**)

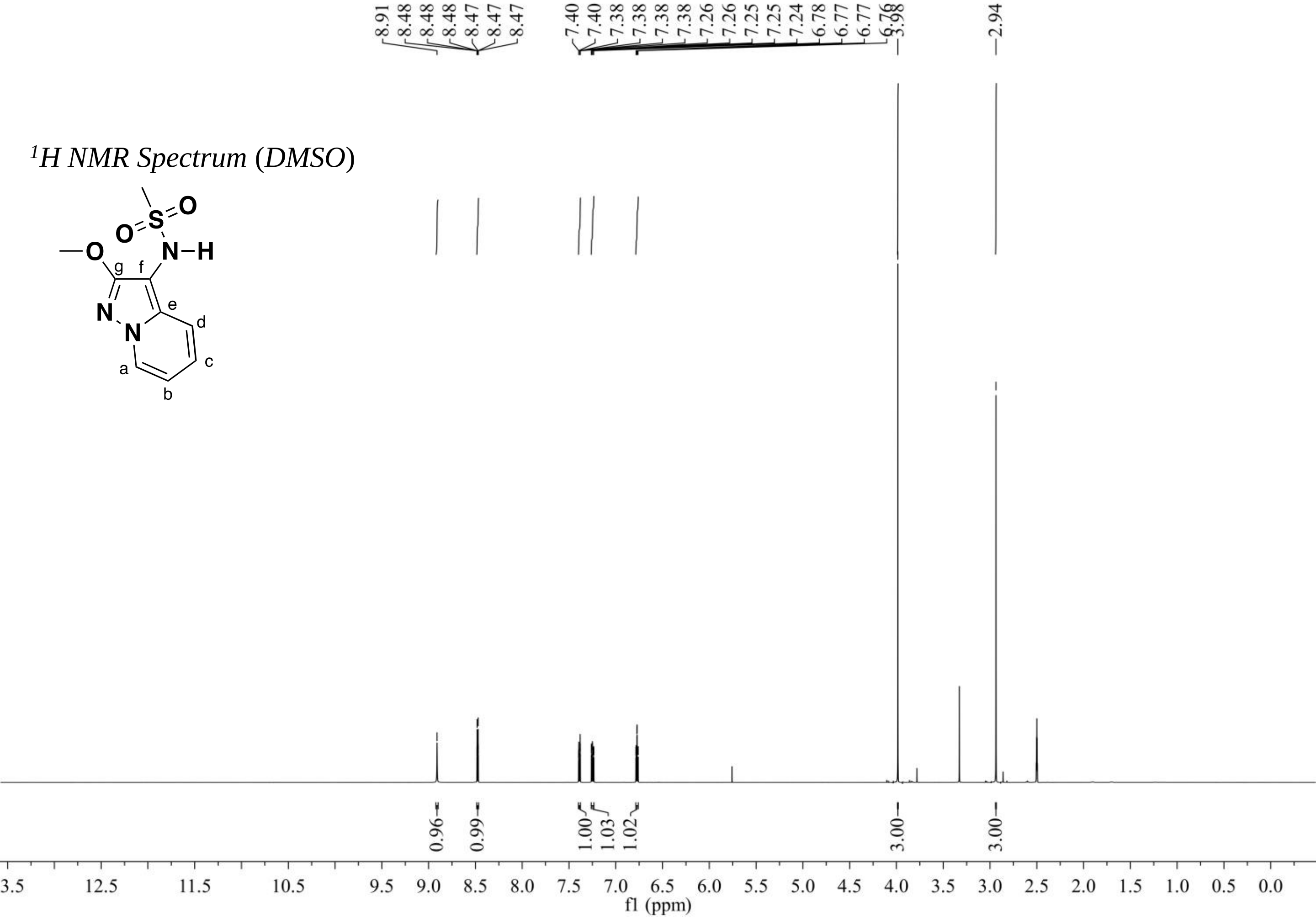

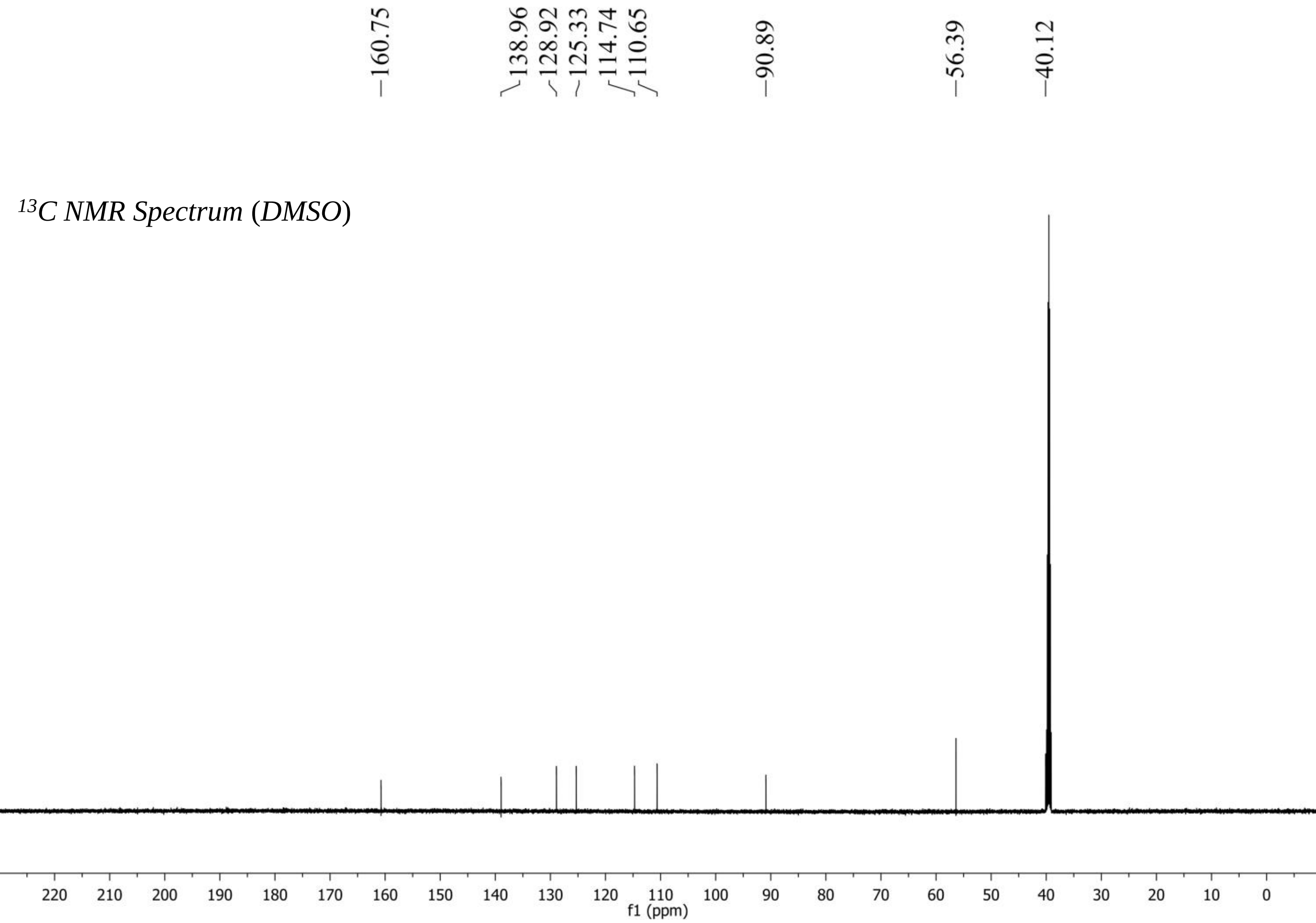

2-methoxypyrazolo[1,5-a]pyridine-3-carbaldehyde (**19**)

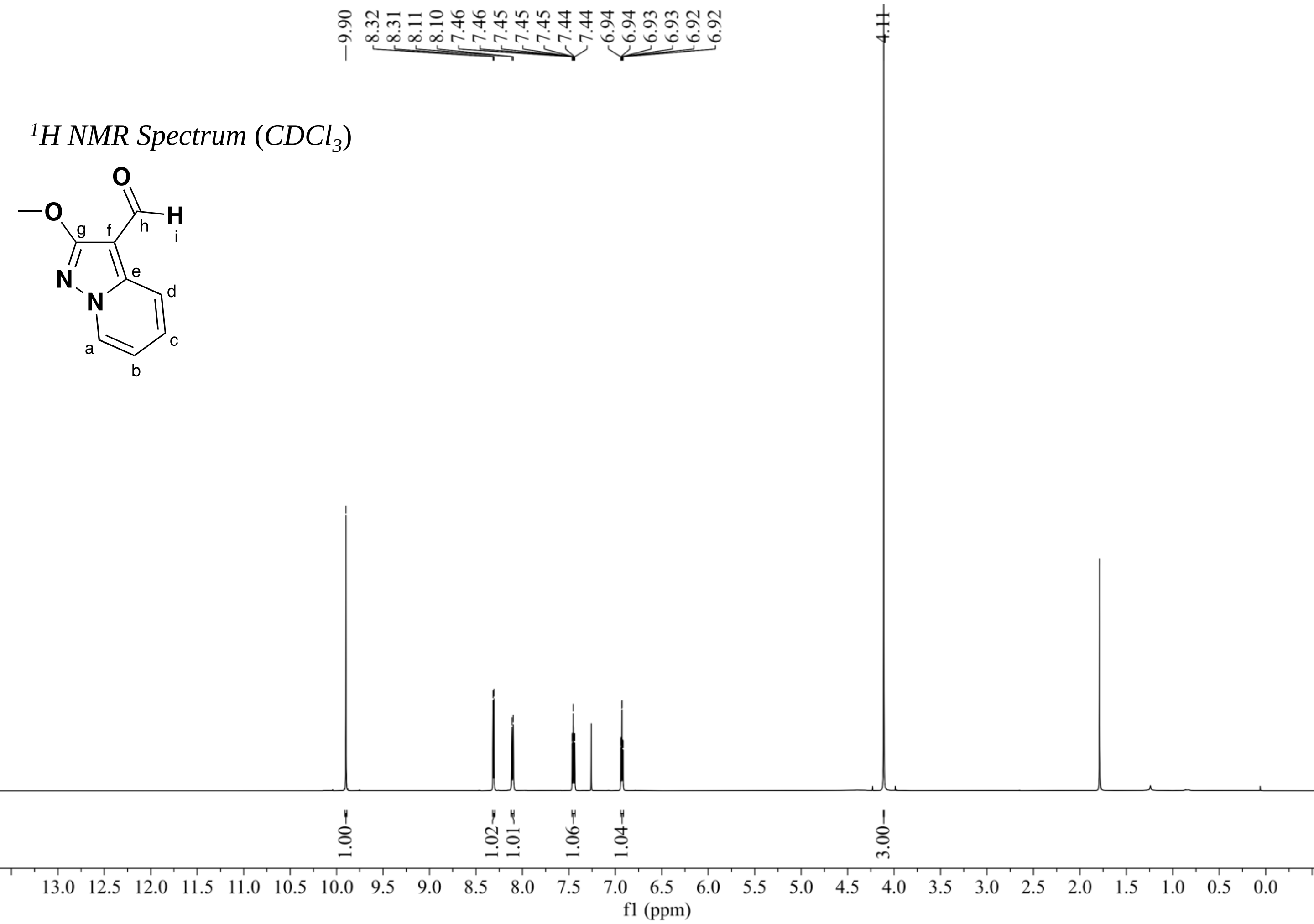

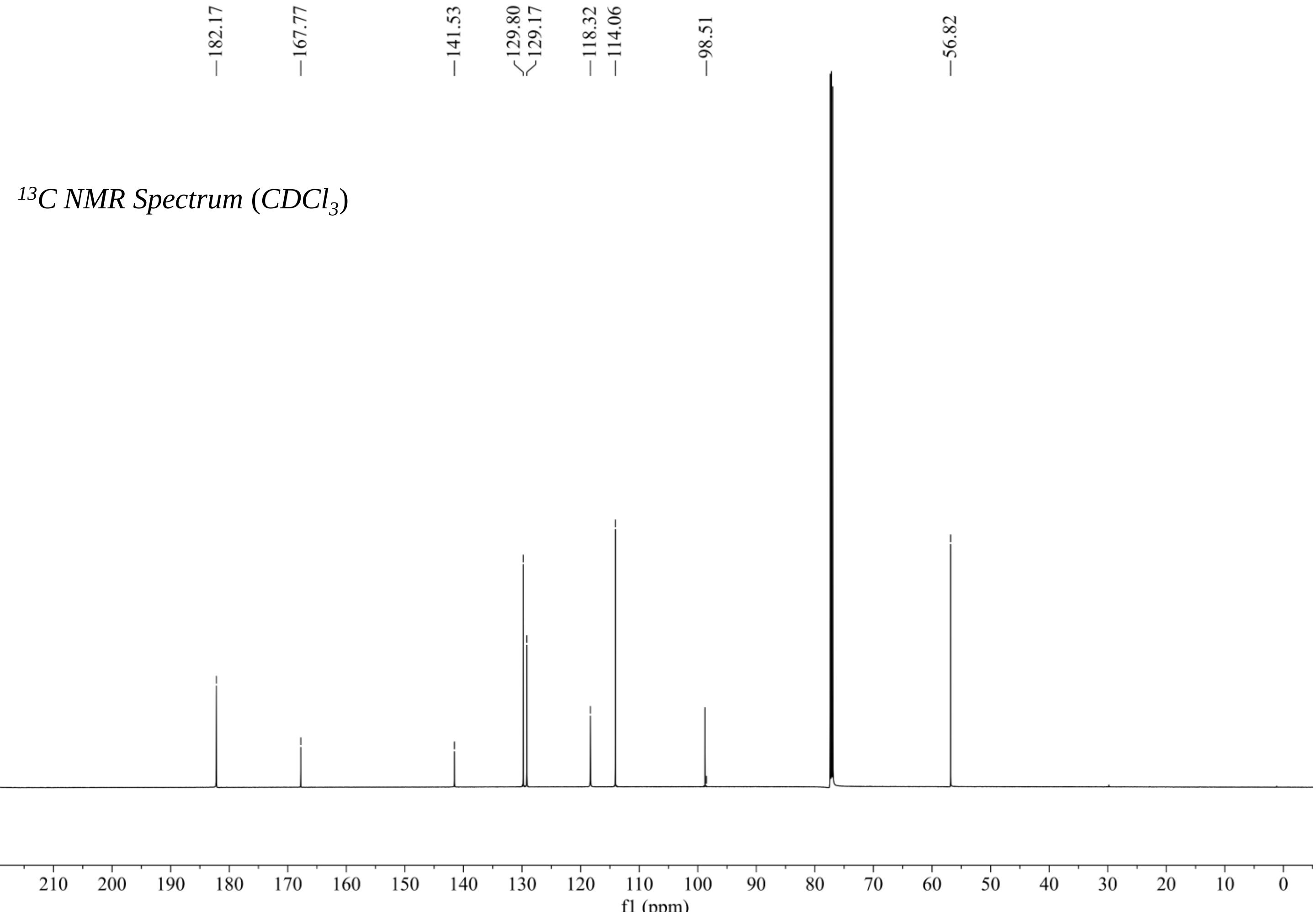

(E)-2-(2-methoxypyrazolo[1,5-a]pyridin-3-yl)vinyl)malononitrile (**4a**)

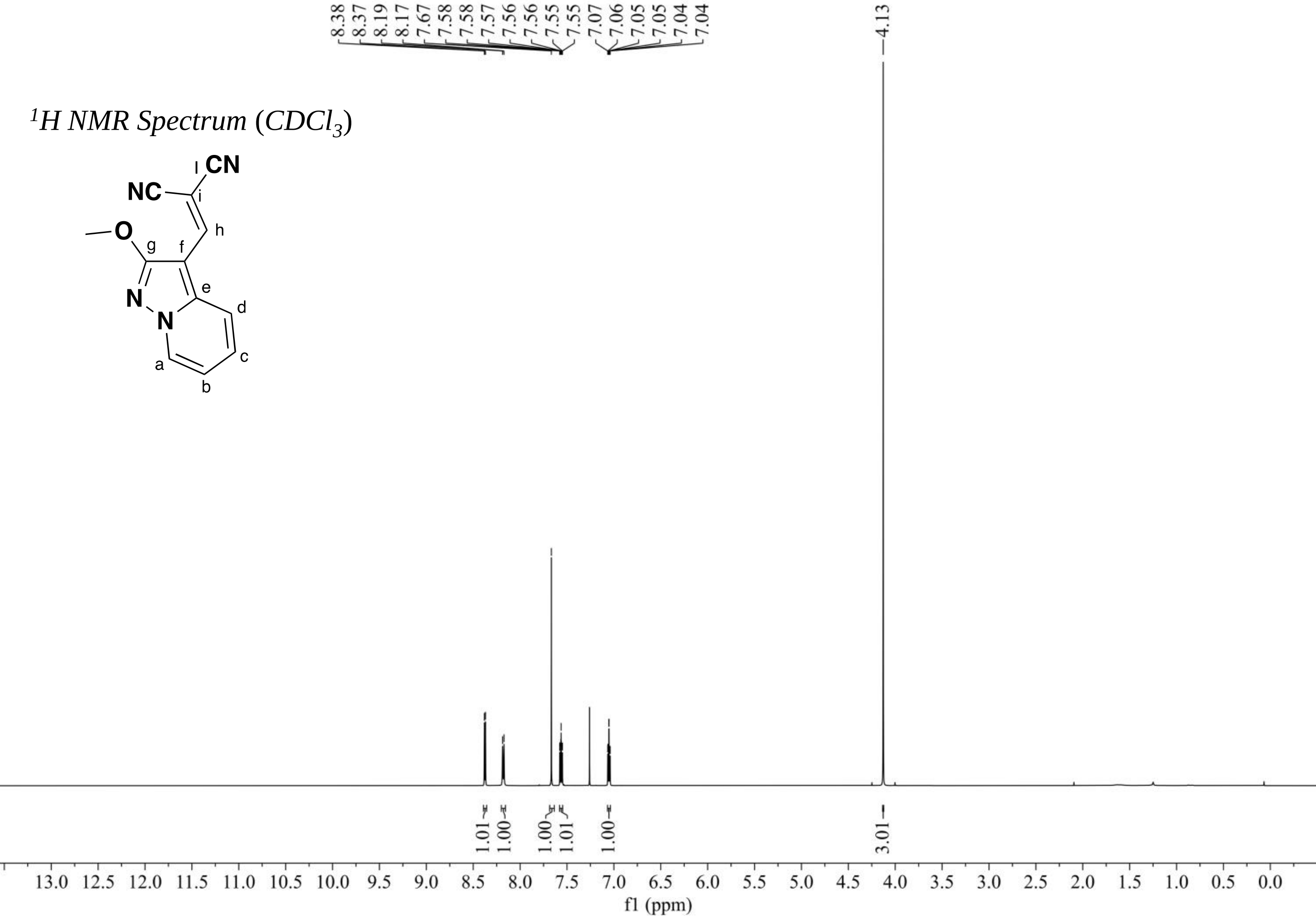

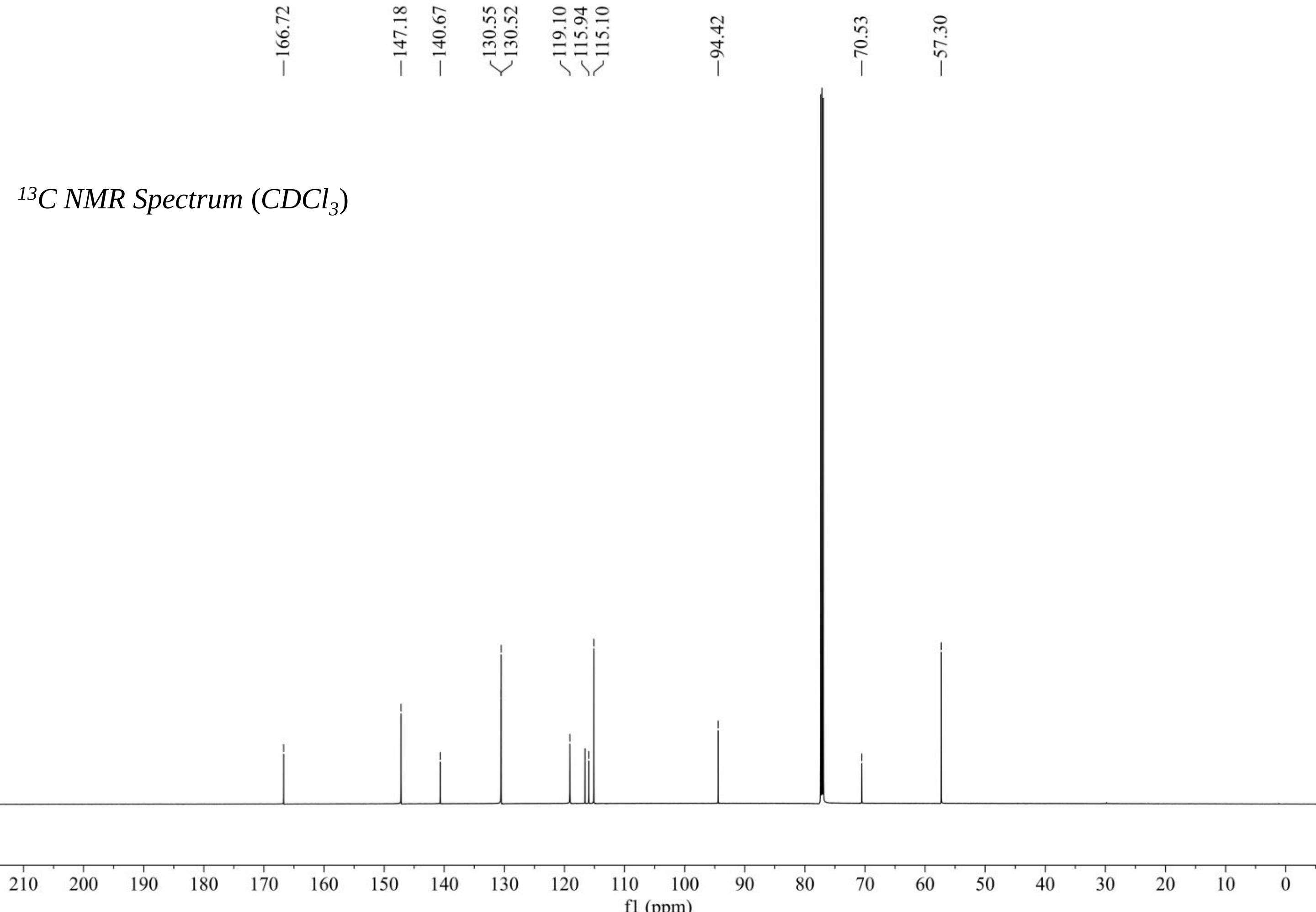

(E)-2-(2-methoxypyrazolo[1,5-a]pyridin-3-yl)vinyl)malononitrile (**4a**)

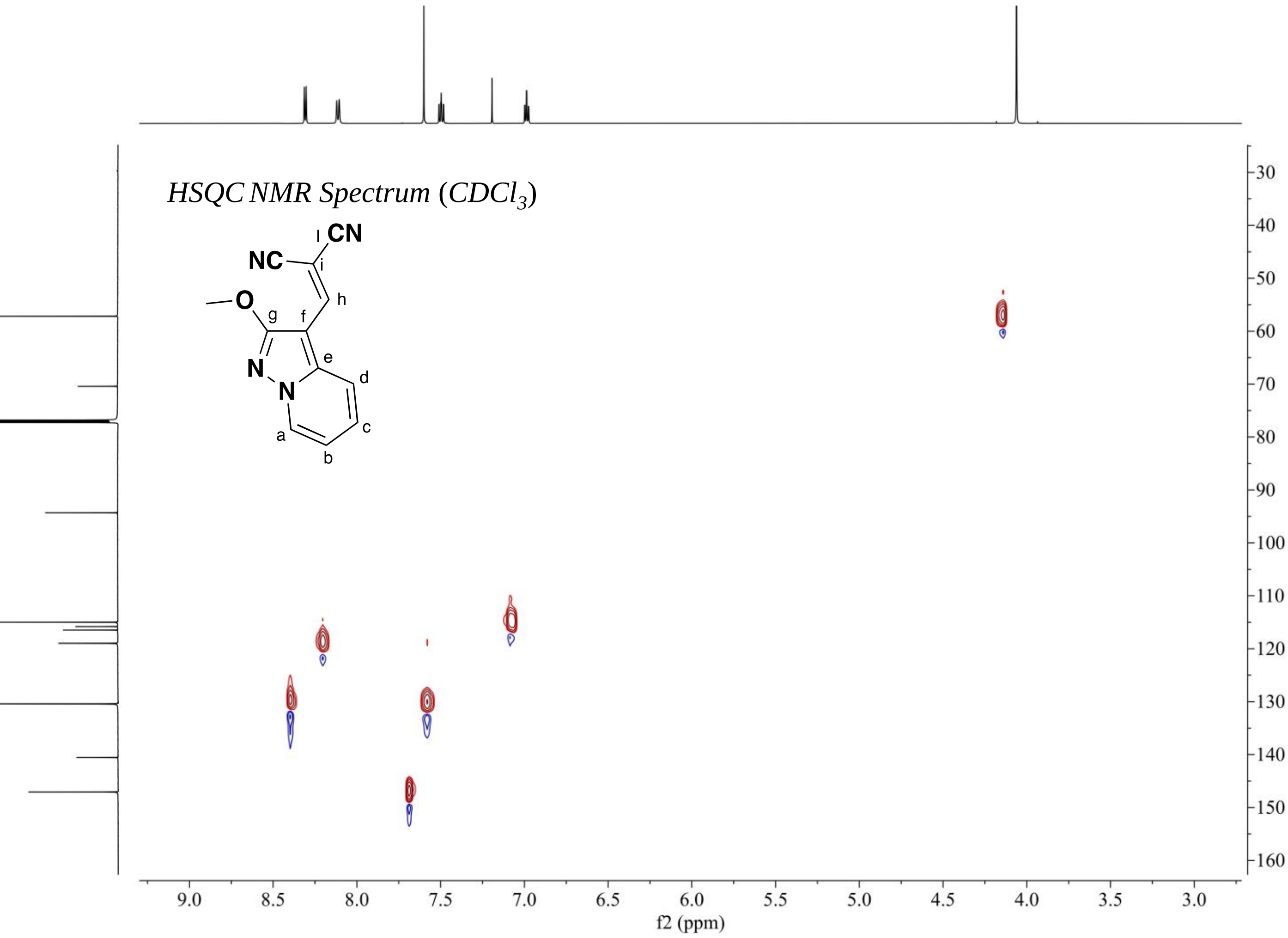

Ethyl (E)-2-cyano-4-(2-methoxypyrazolo[1,5-a]pyridin-3-yl)but-3-enoate (**4b**)

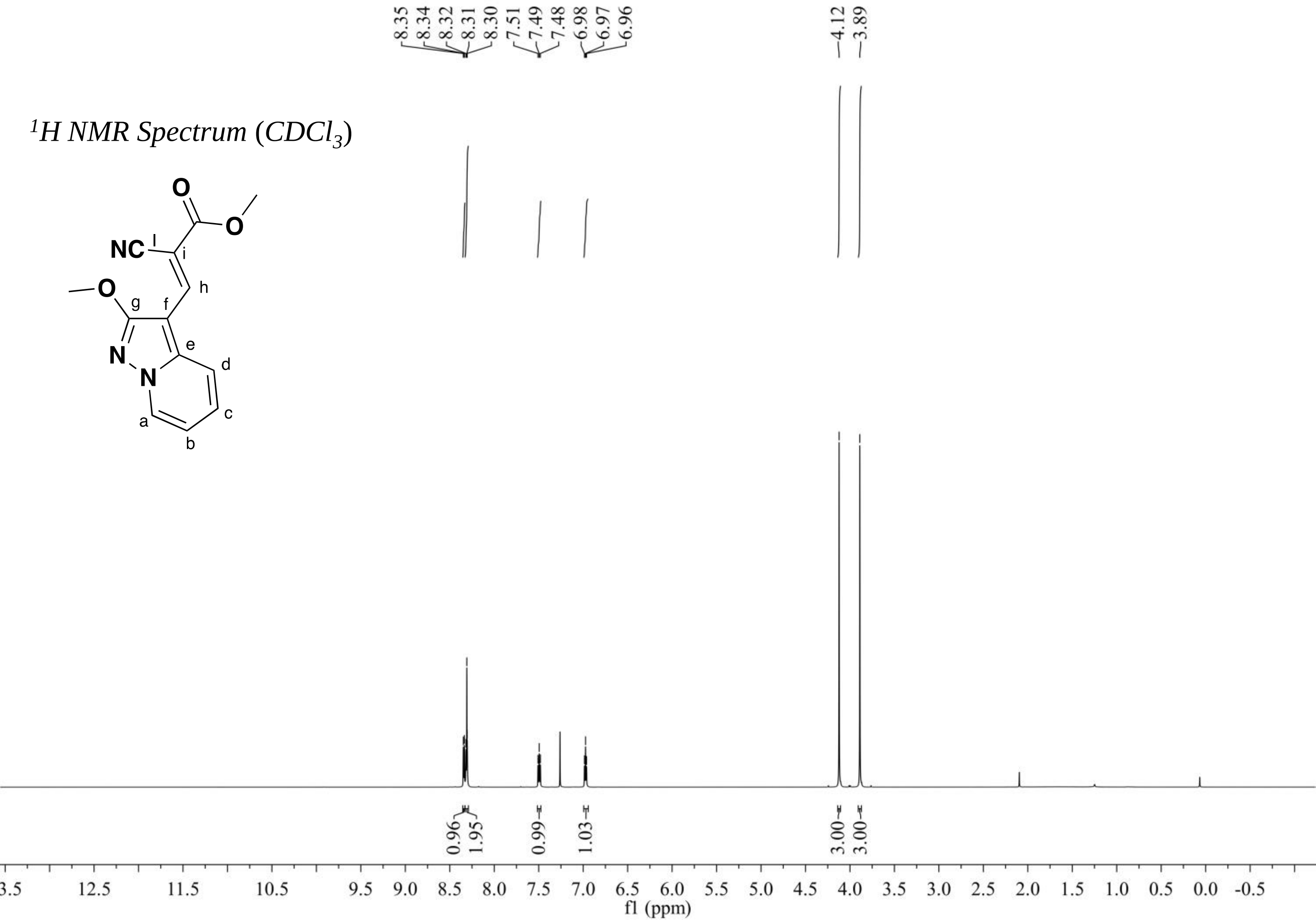

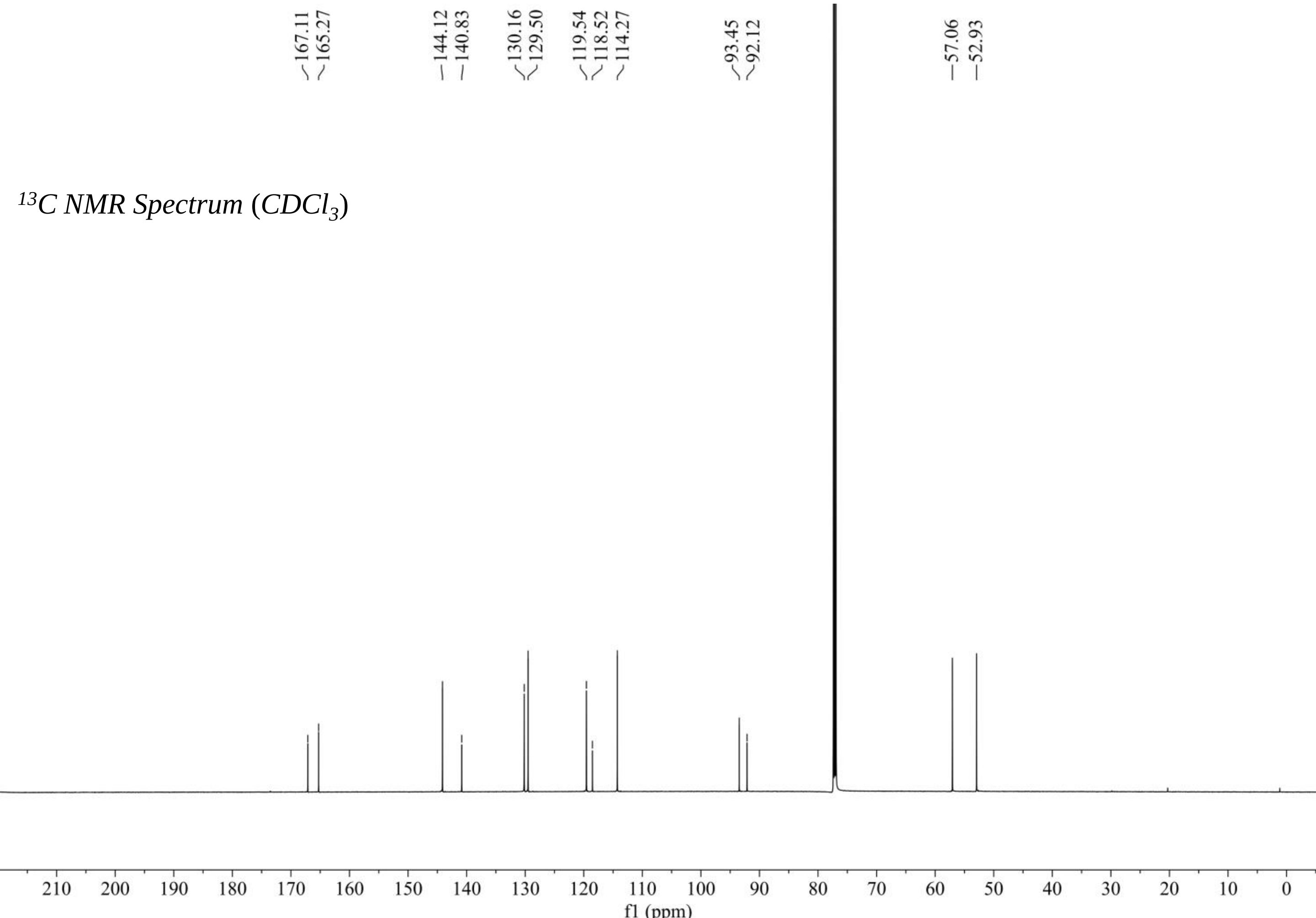

Ethyl (E)-2-cyano-4-(2-methoxypyrazolo[1,5-a]pyridin-3-yl)but-3-enoate (**4b**)

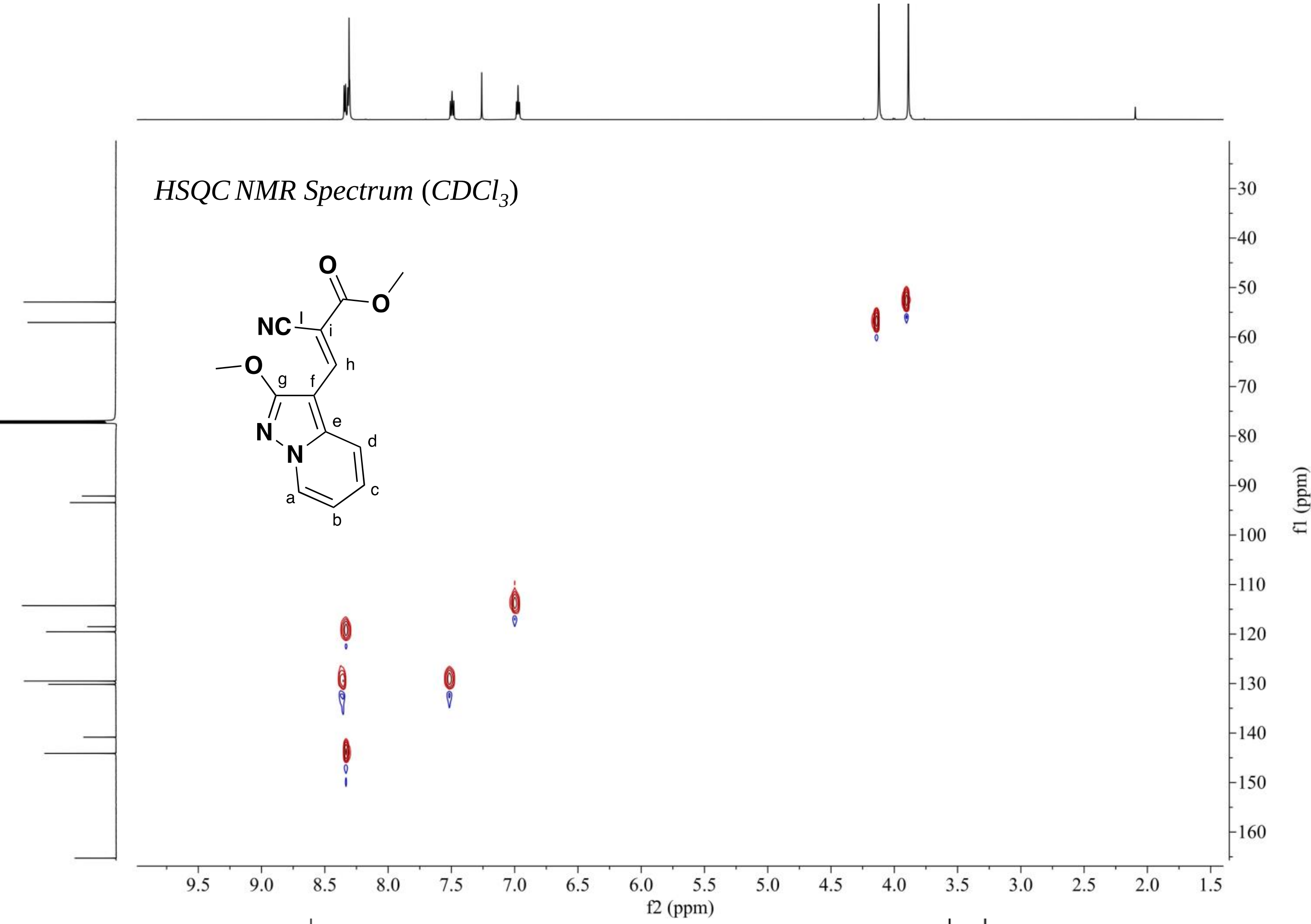

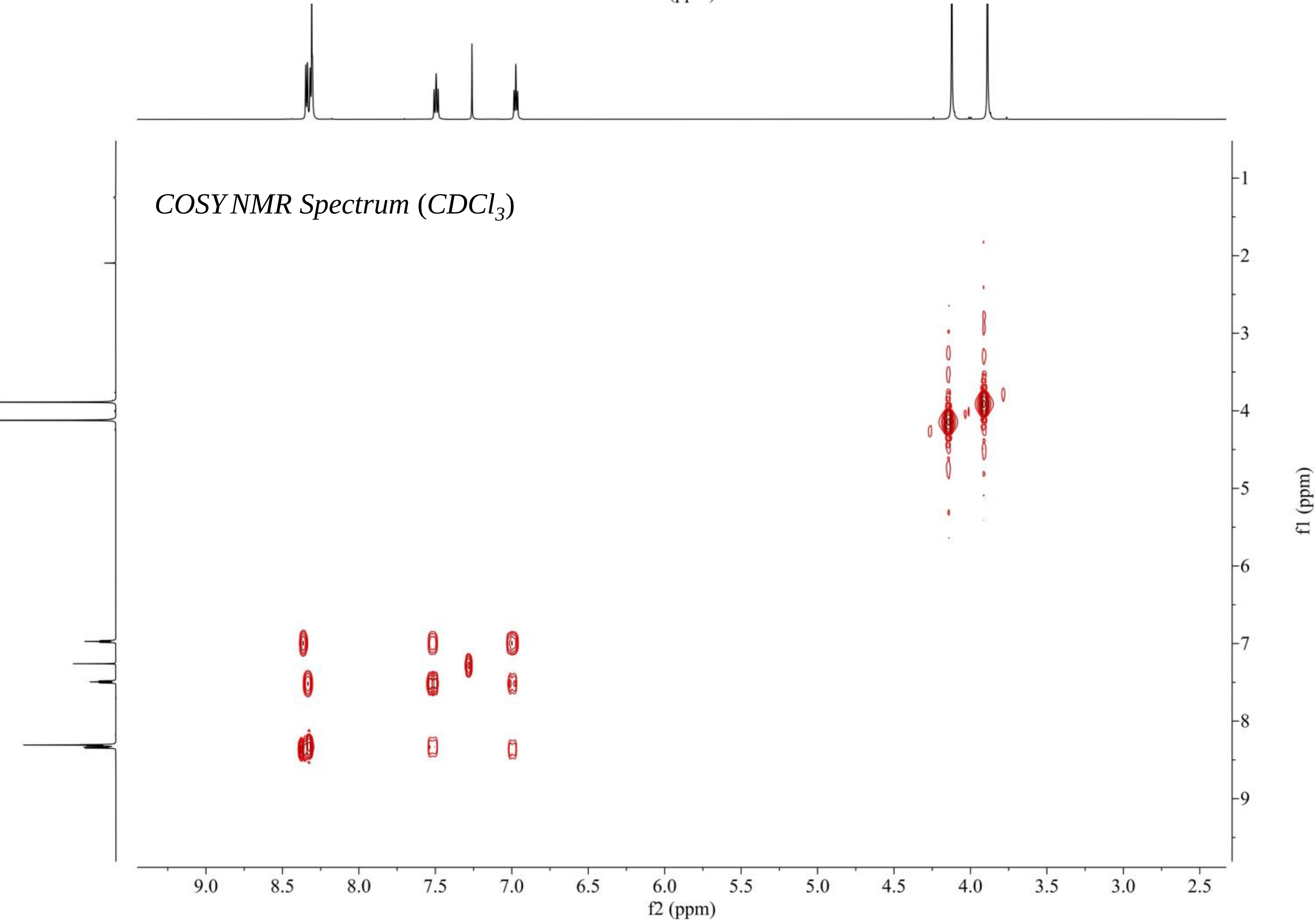

Ethyl (E)-2-cyano-4-(2-methoxypyrazolo[1,5-a]pyridin-3-yl)but-3-enoate (**4b**)

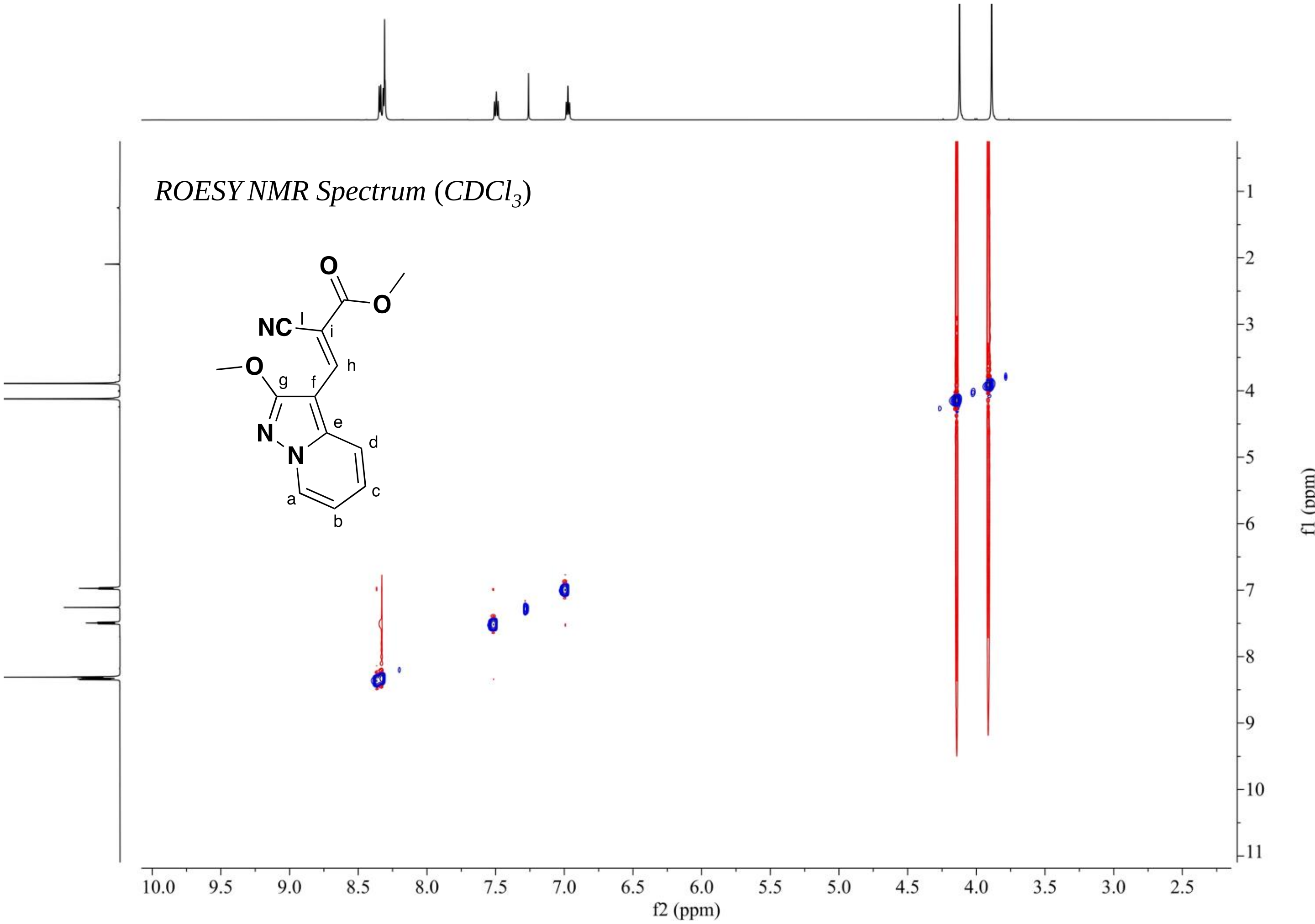

(E)-2-methoxy-3-styrylpyrazolo[1,5-a]pyridine (**5a**)

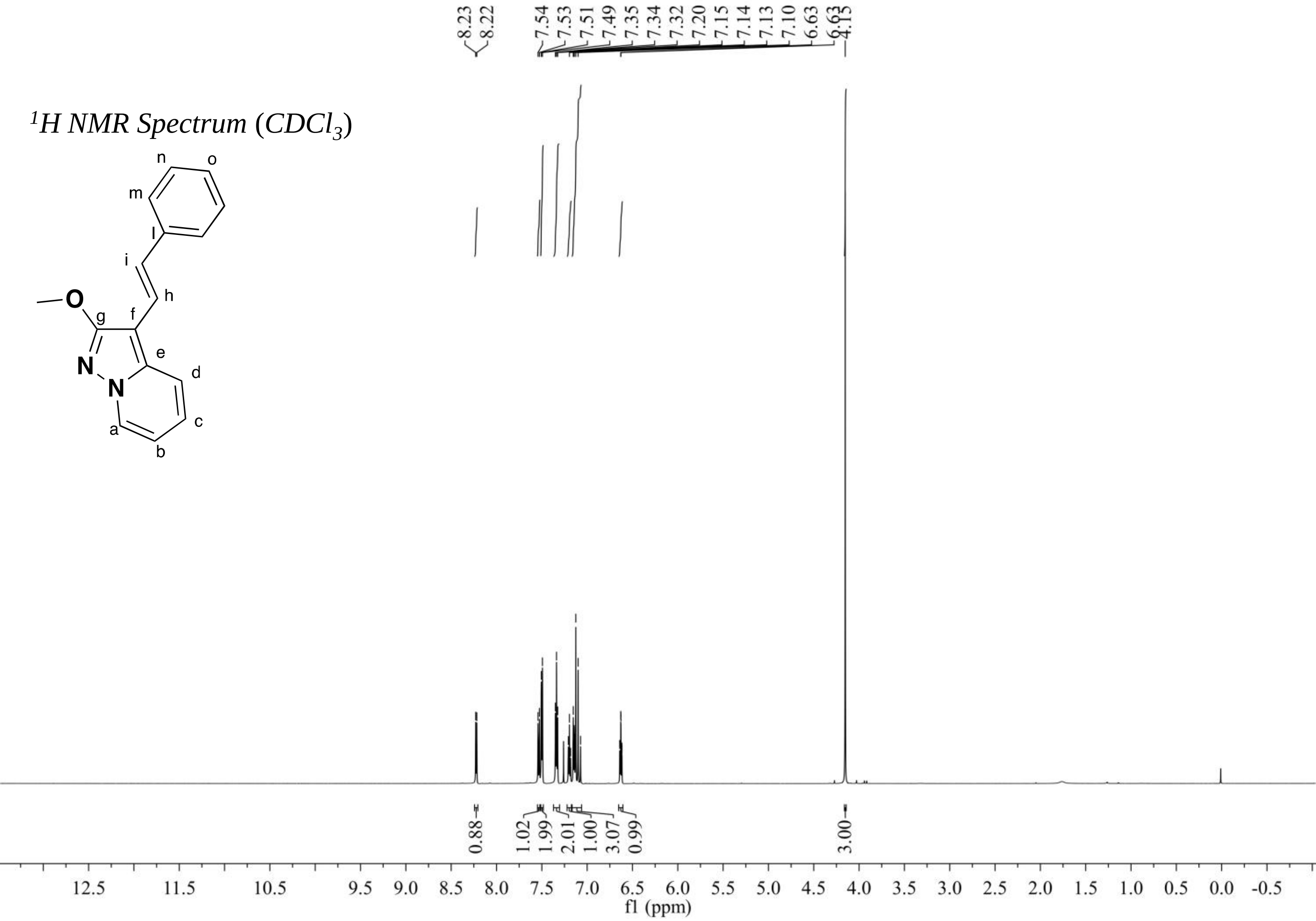

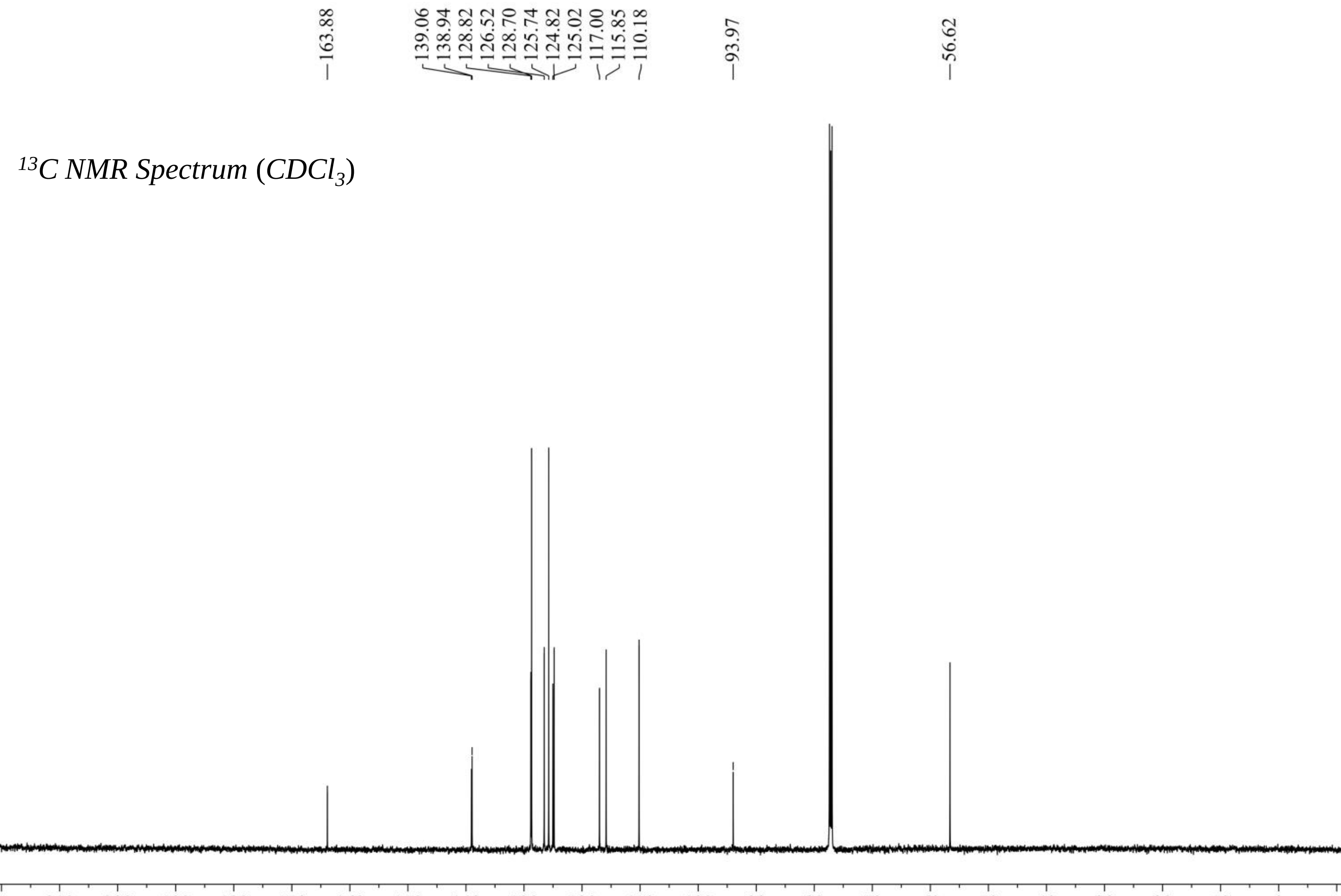

(E)-2-methoxy-3-(4-(methylthio)styryl)pyrazolo[1,5-a]pyridine (**5b**)

^{1}H NMR Spectrum (*CDCl$_3$*)

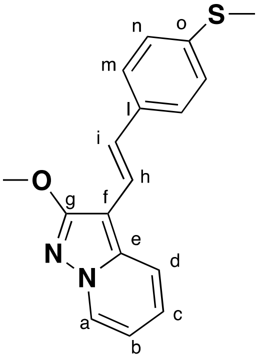

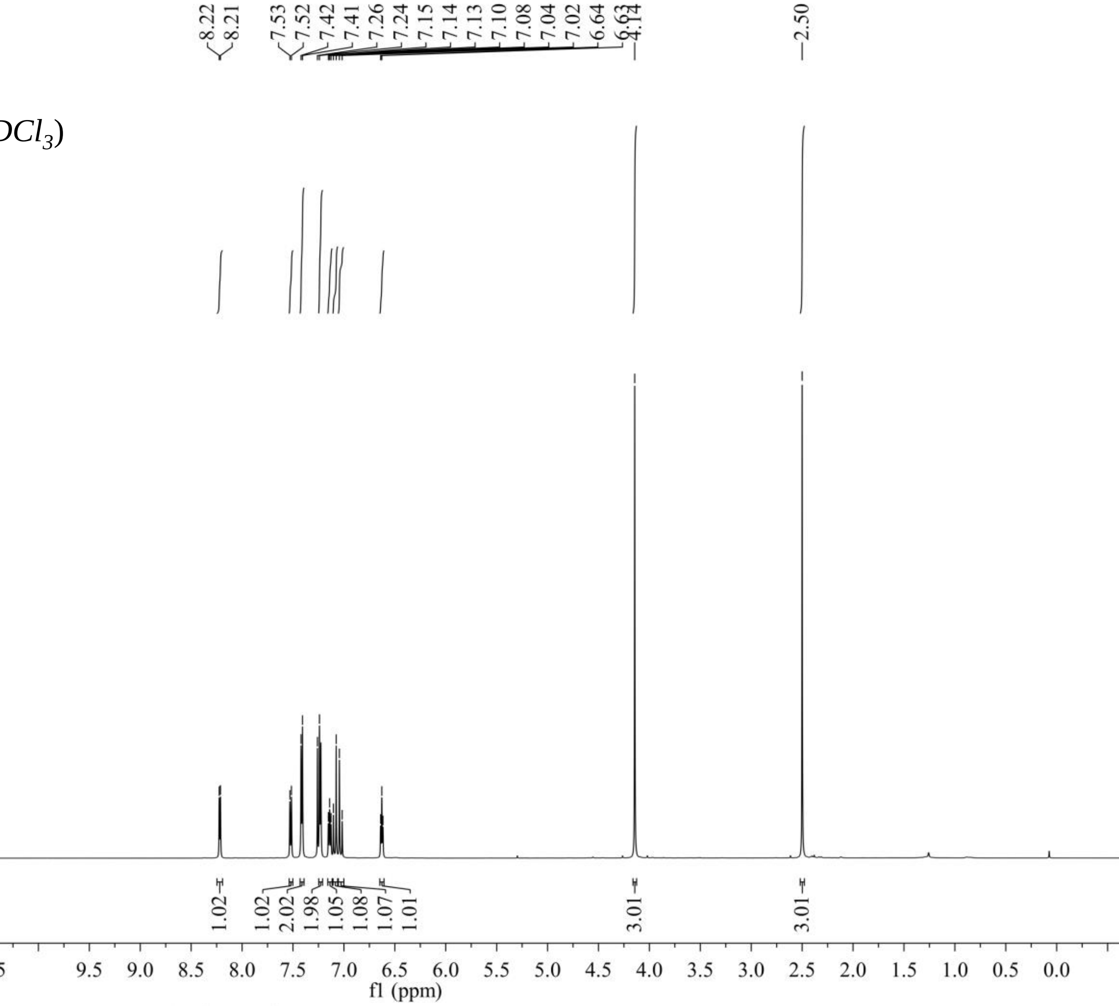

^{13}C NMR Spectrum (*CDCl$_3$*)

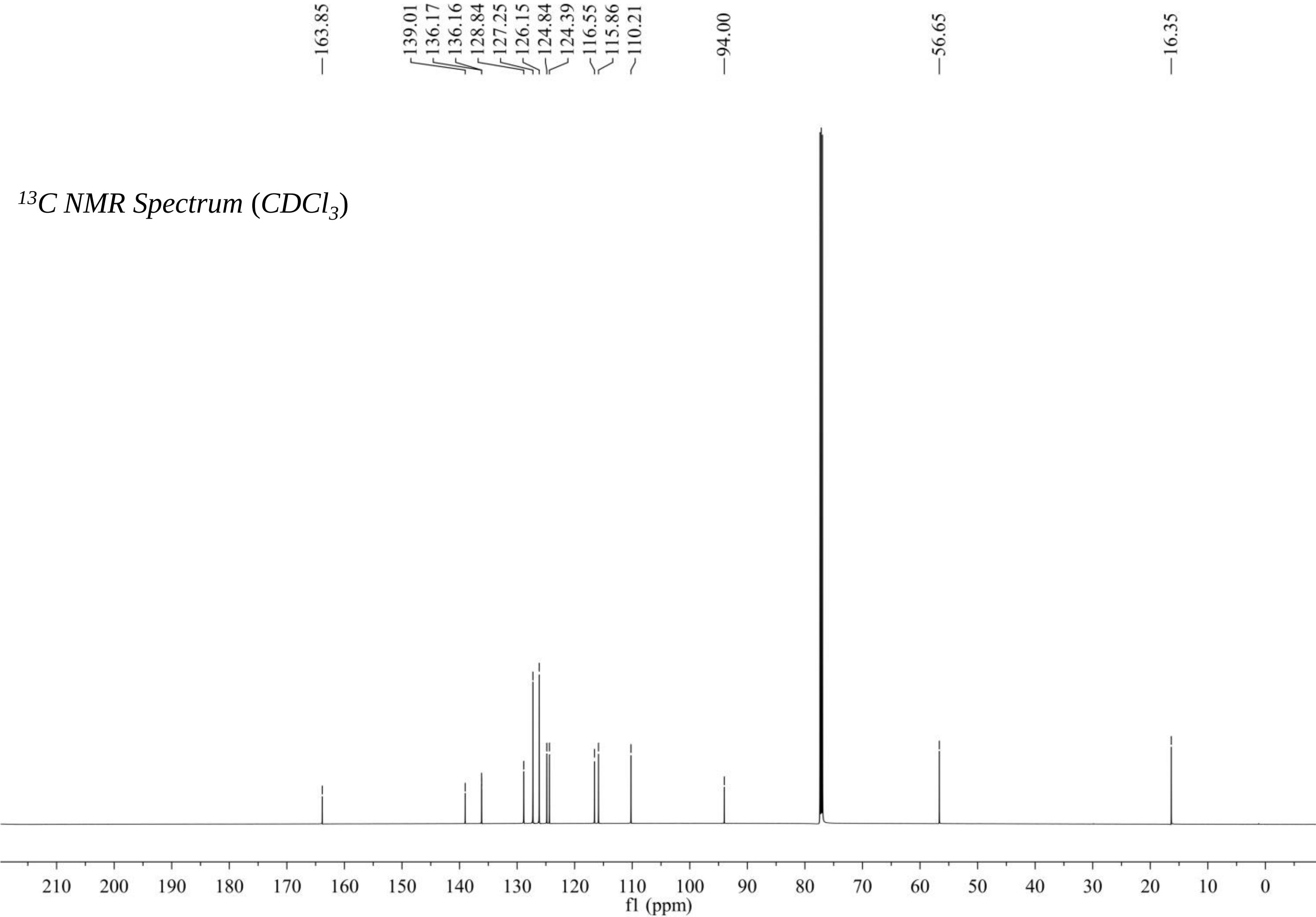

(E)-2-methoxy-3-(4-(methylsulfonyl)styryl)pyrazolo[1,5-a]pyridine (**5c**)

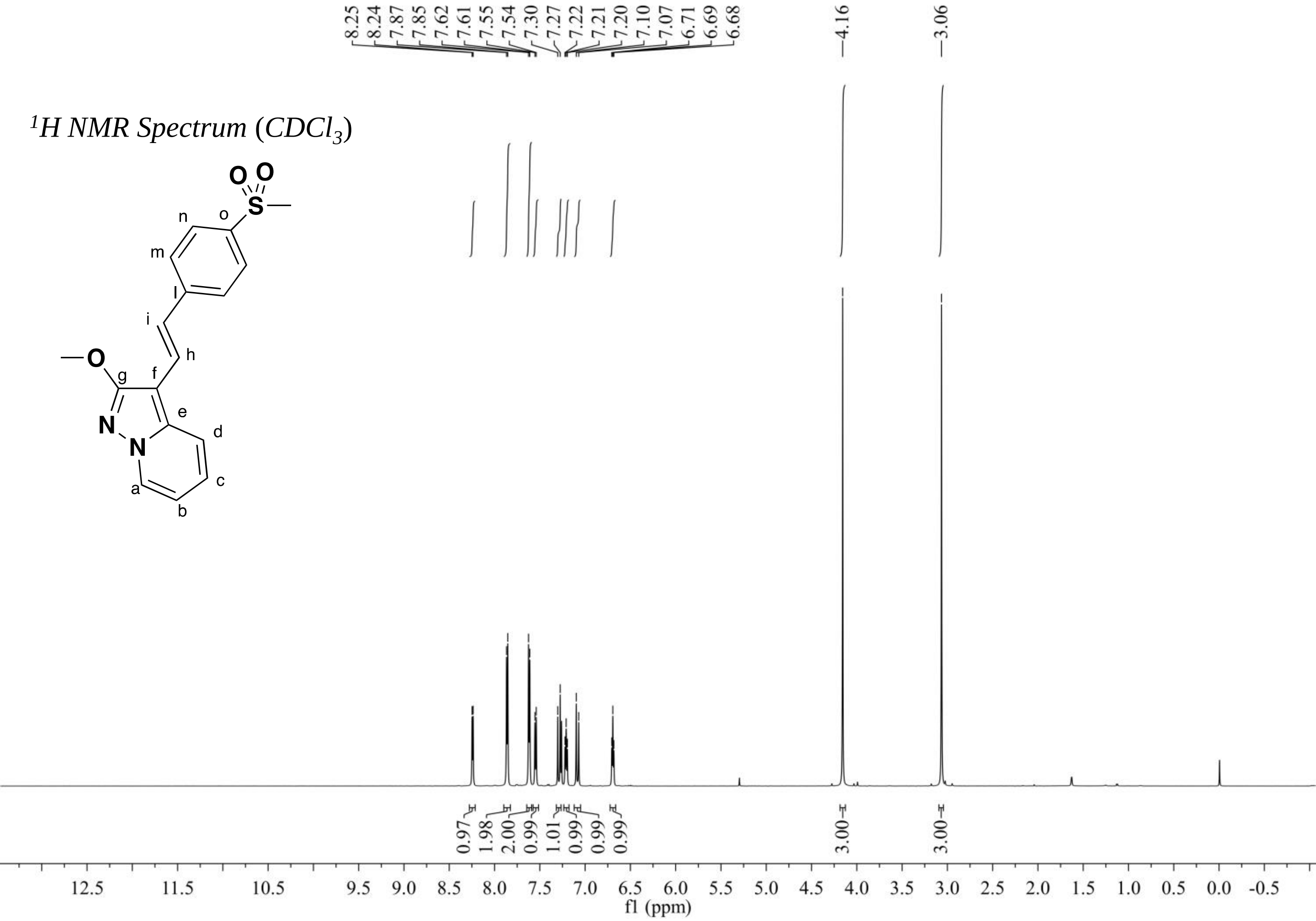

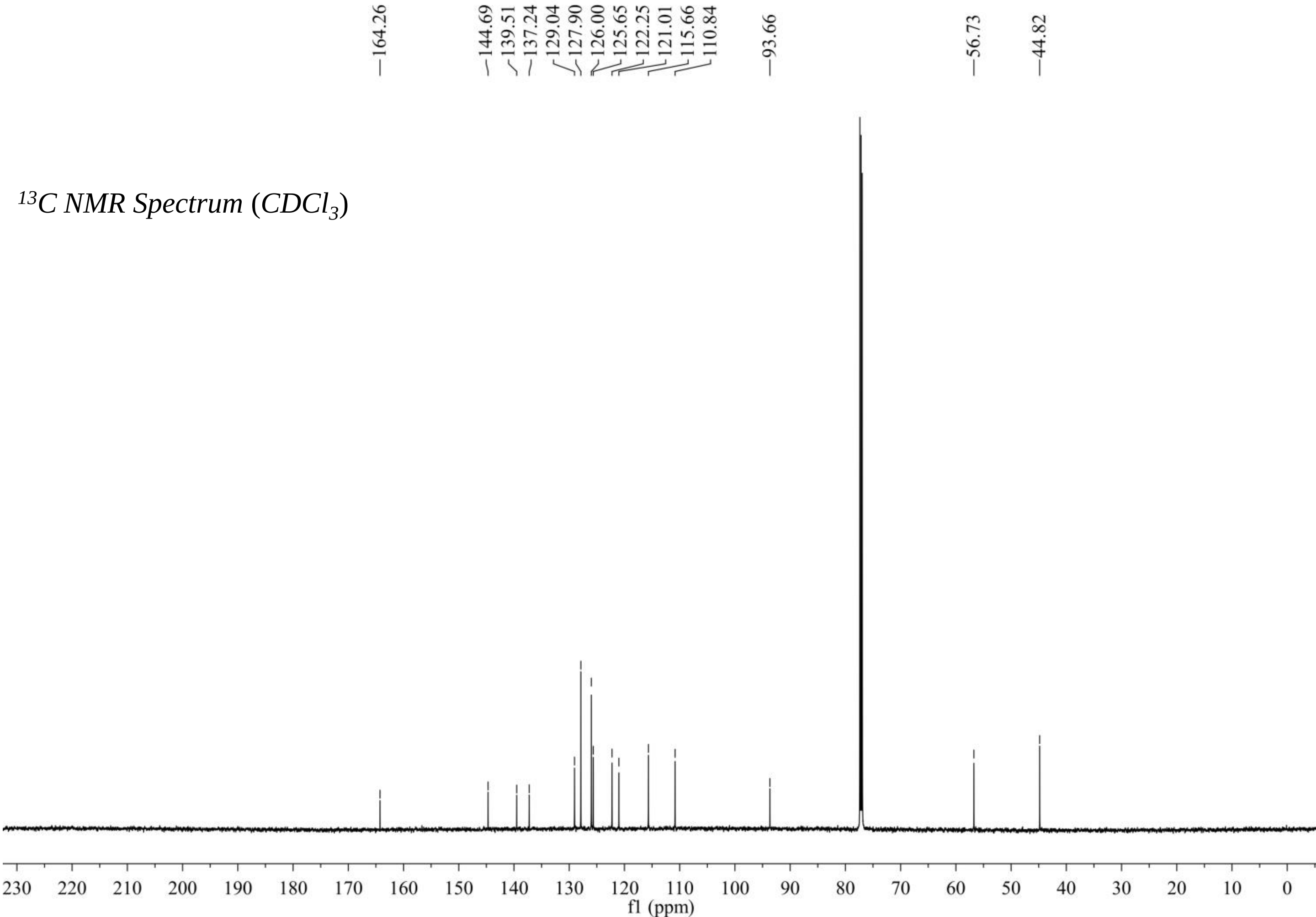

(E)-4-(2-(2-methoxypyrazolo[1,5-a]pyridin-3-yl)vinyl)benzonitrile (**5d**)

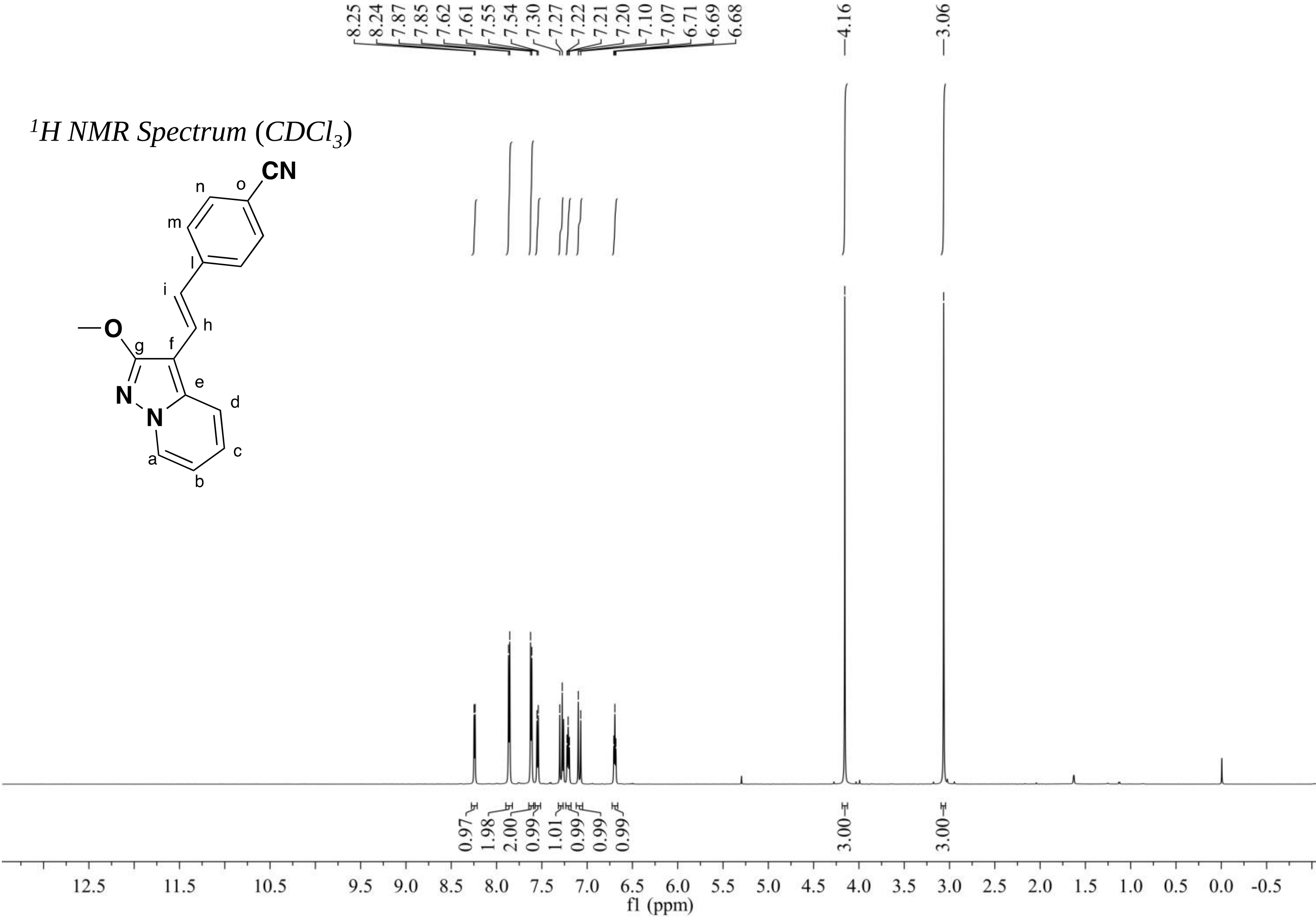

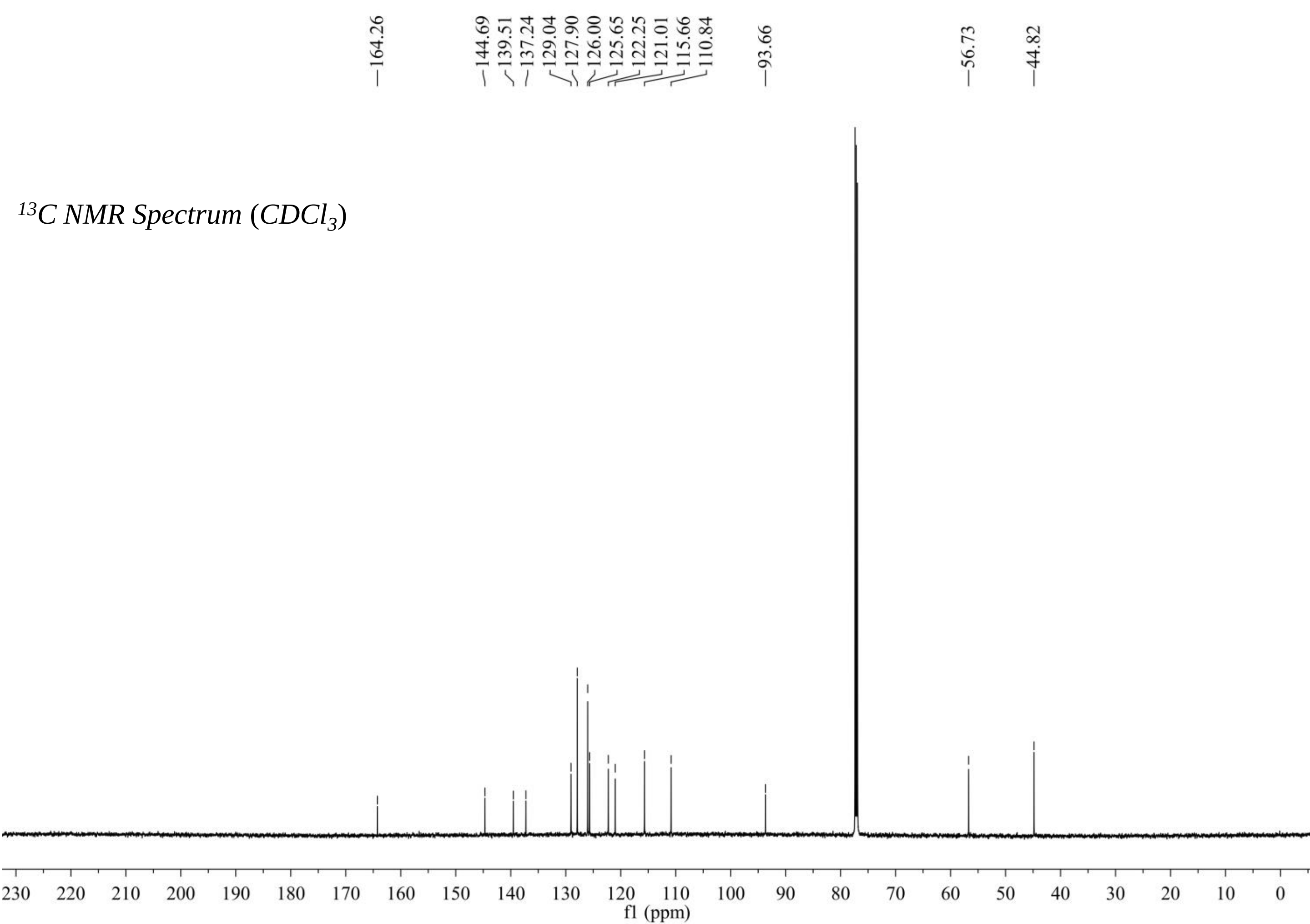

(E)-2-methoxy-3-(4-nitrostyryl)pyrazolo[1,5-a]pyridine (**5e**)

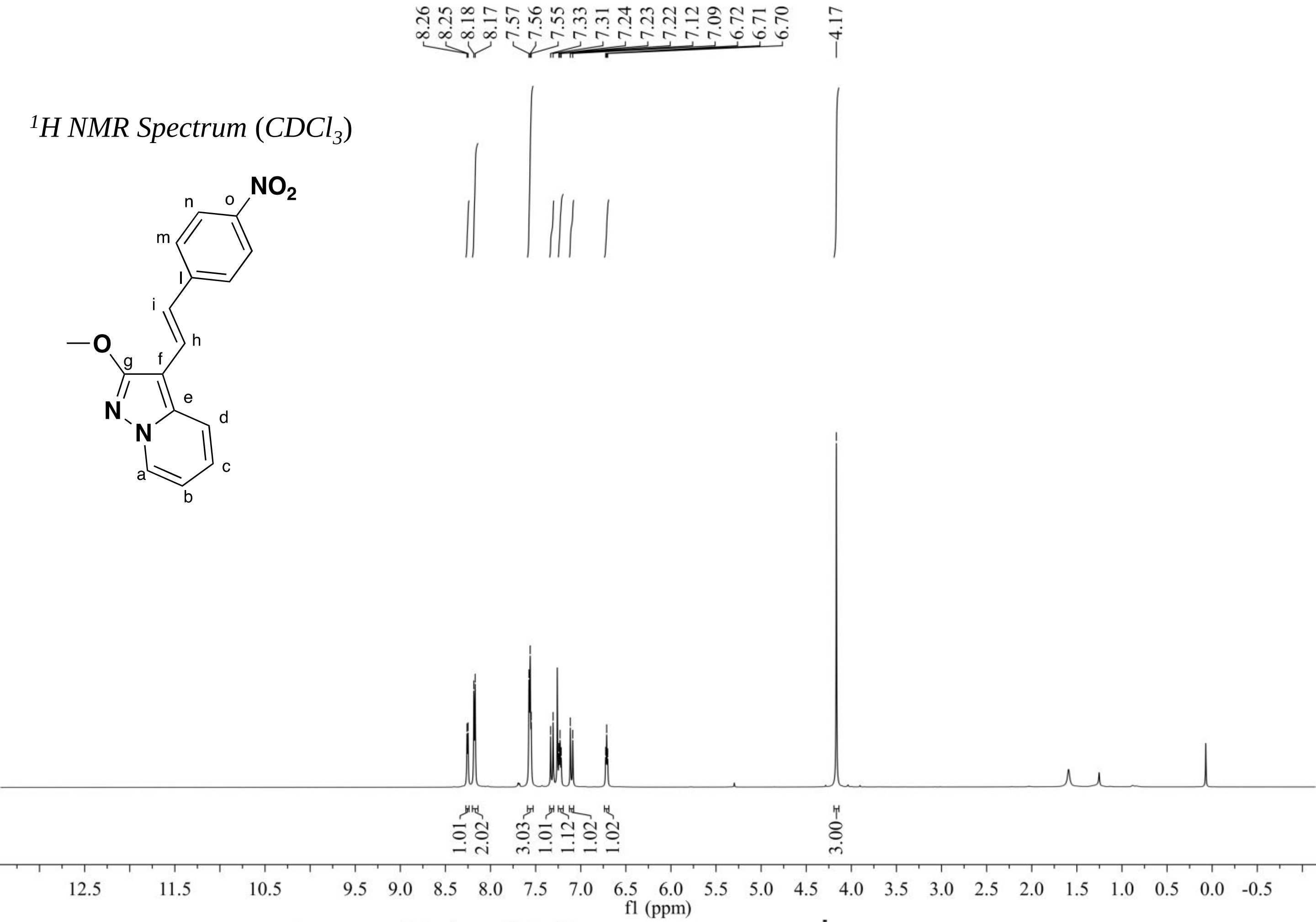

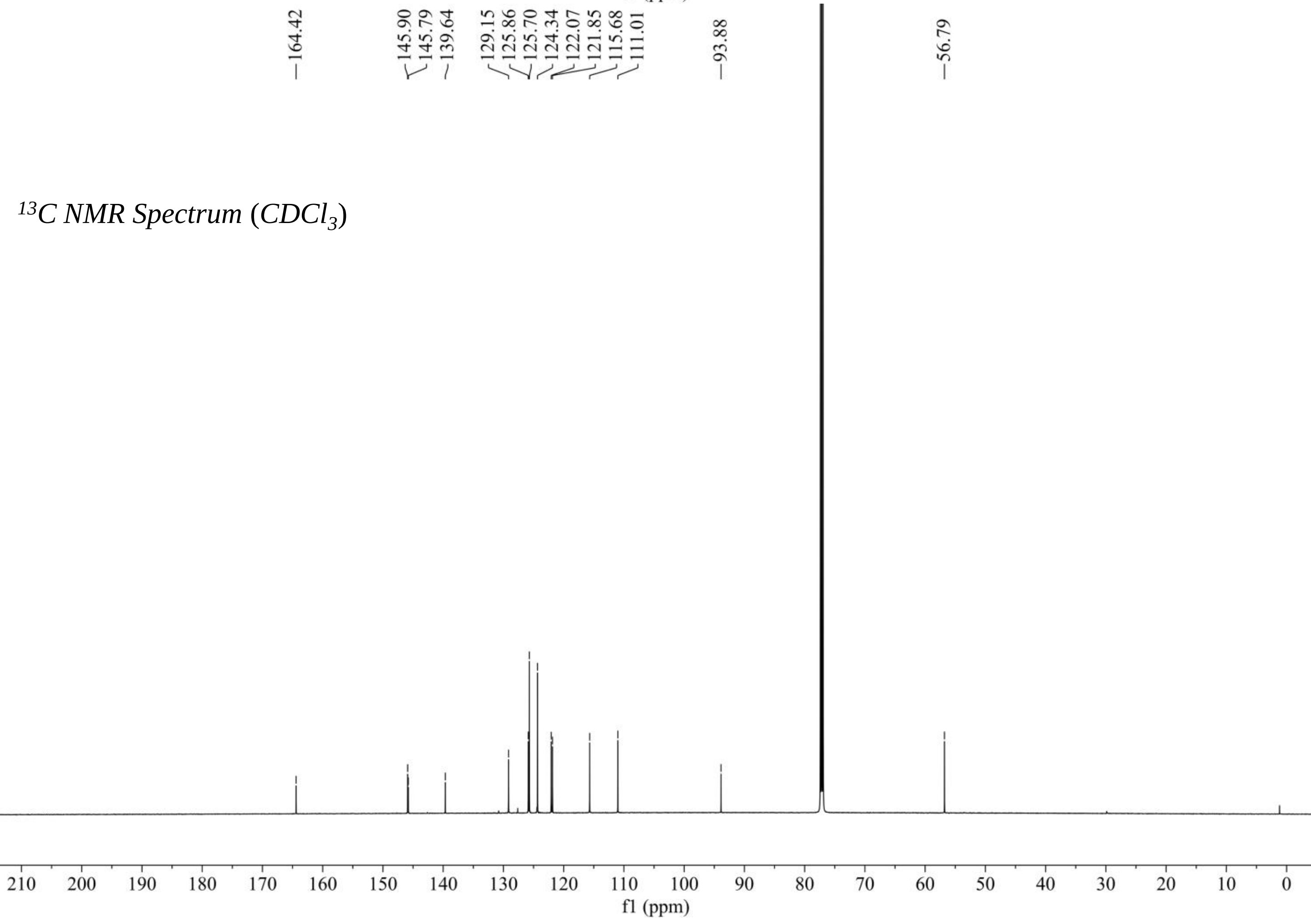

(E)-2-methoxy-3-(4-nitrostyryl)pyrazolo[1,5-a]pyridine (**5e**)

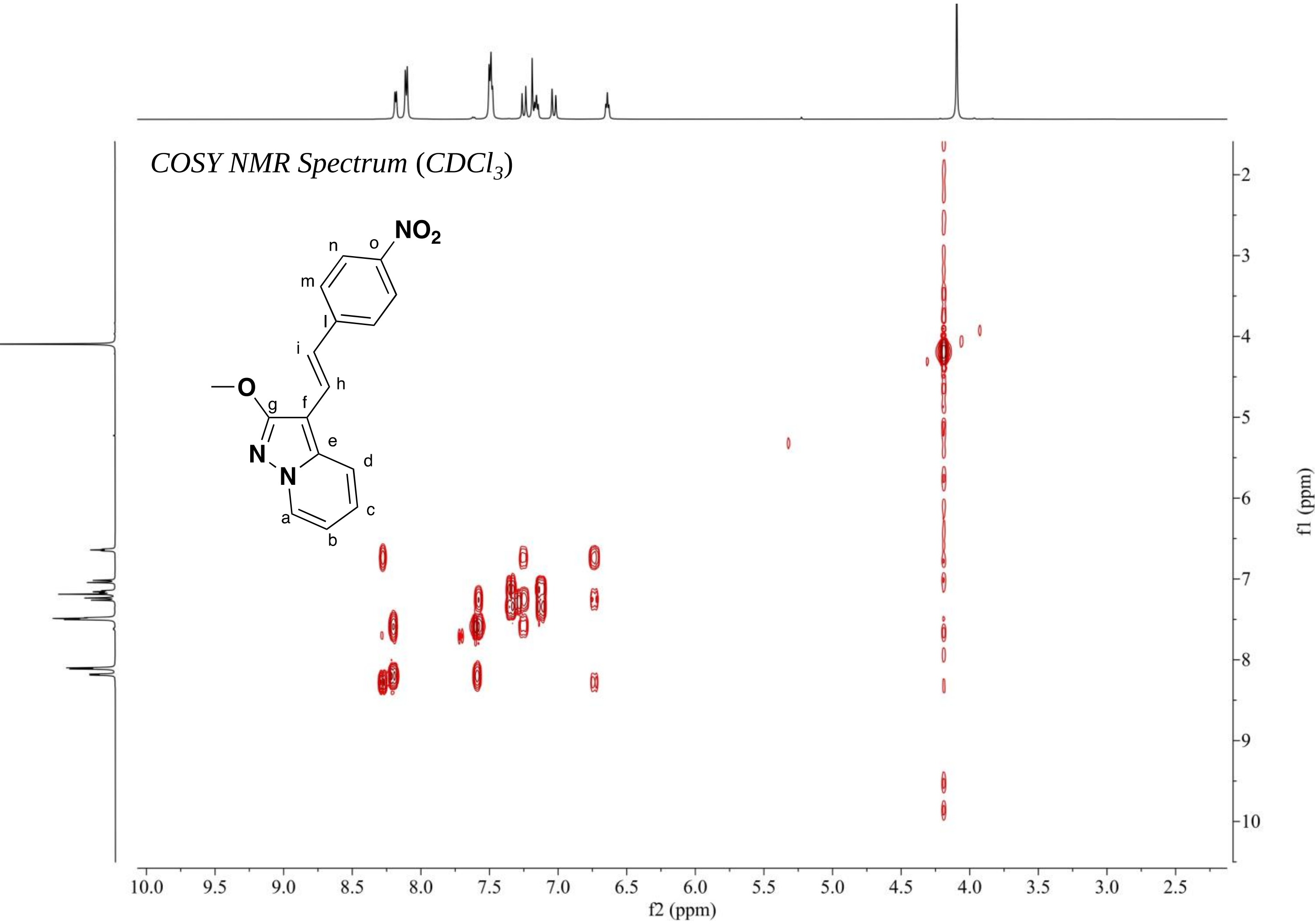

3-bromo-2-methoxypyrazolo[1,5-a]pyridine (**20**)

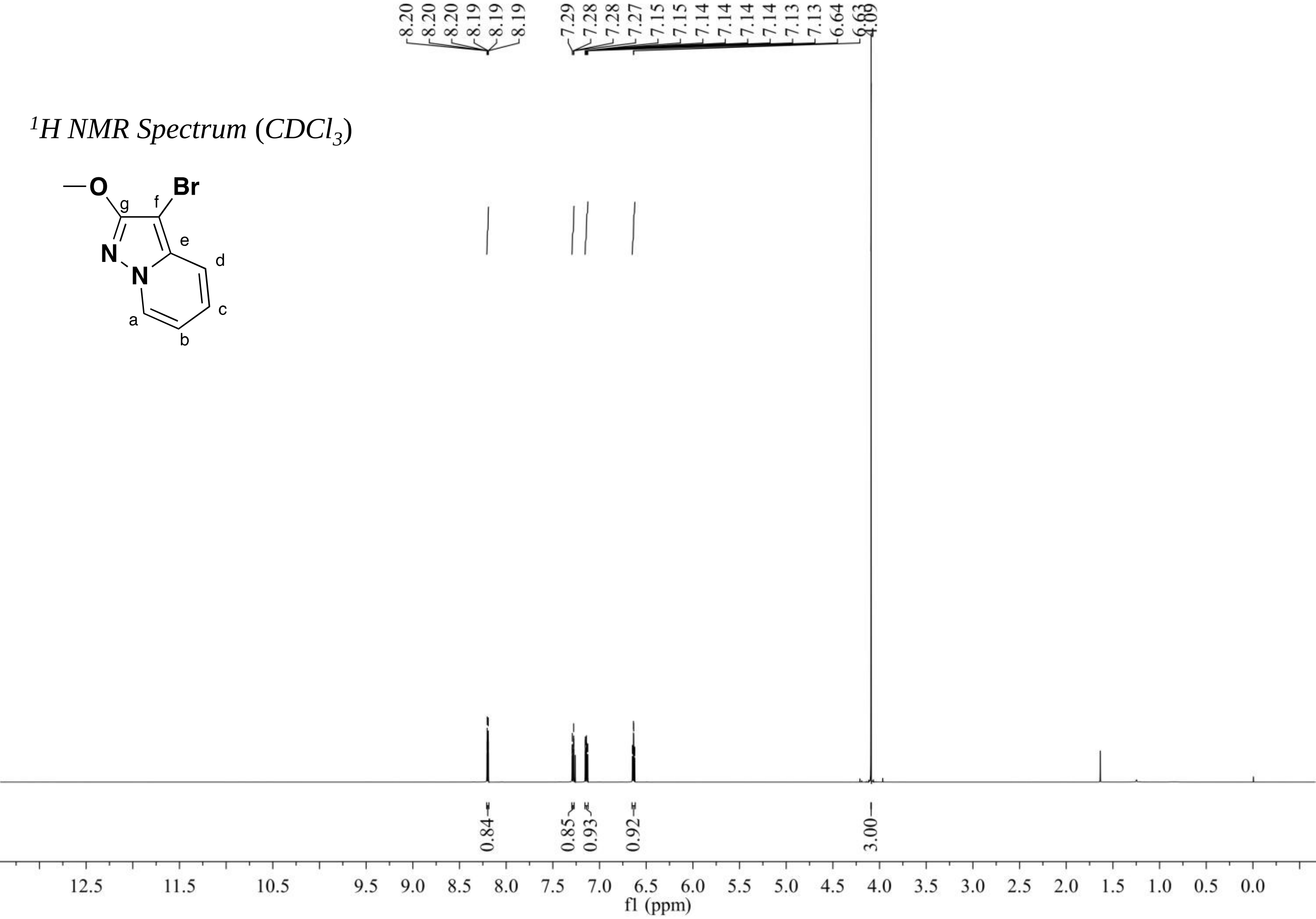

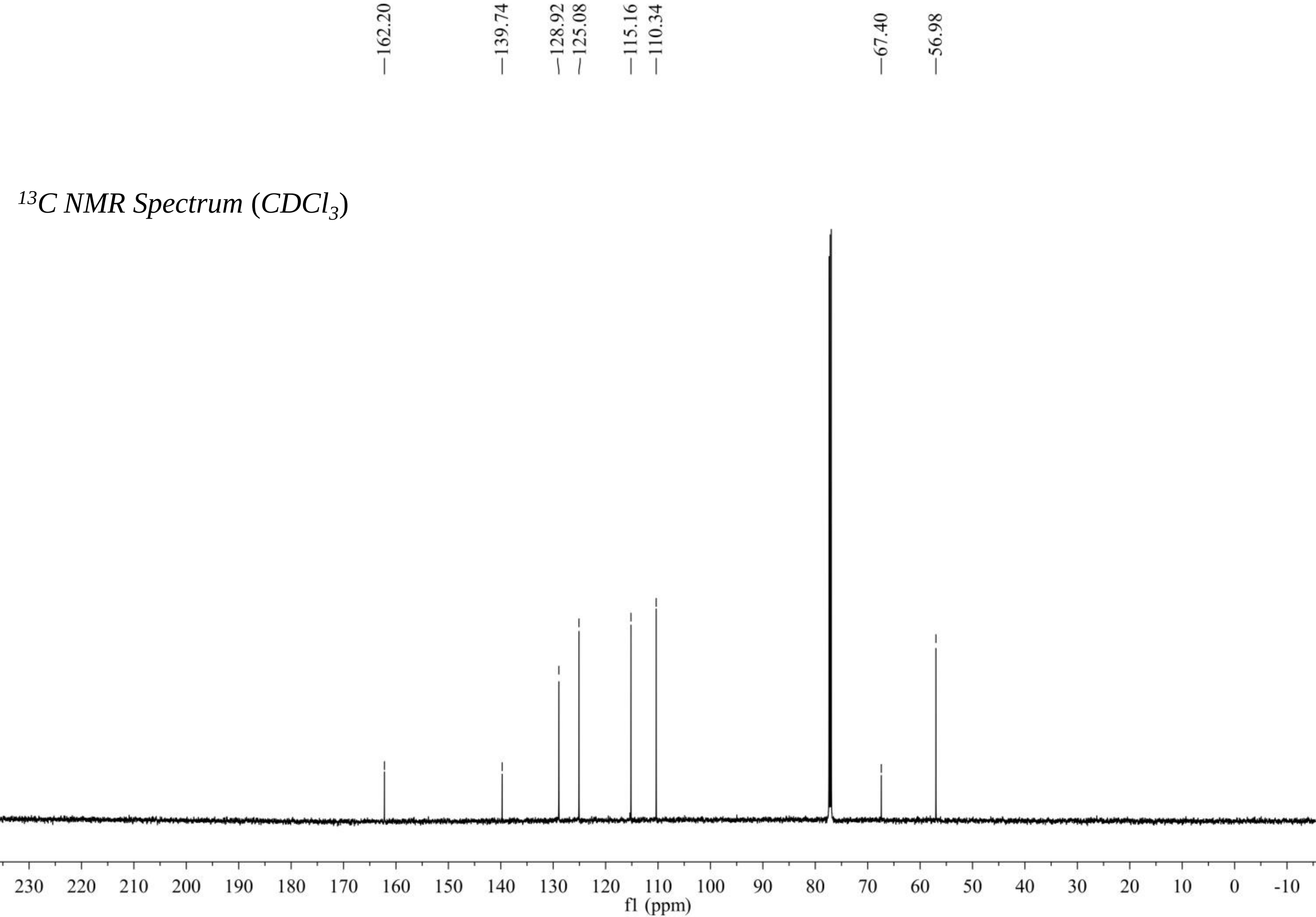

2-methoxy-3-(naphthalen-2-yl)pyrazolo[1,5-a]pyridine (**6**)

^{1}H NMR Spectrum (*$CDCl_3$*)

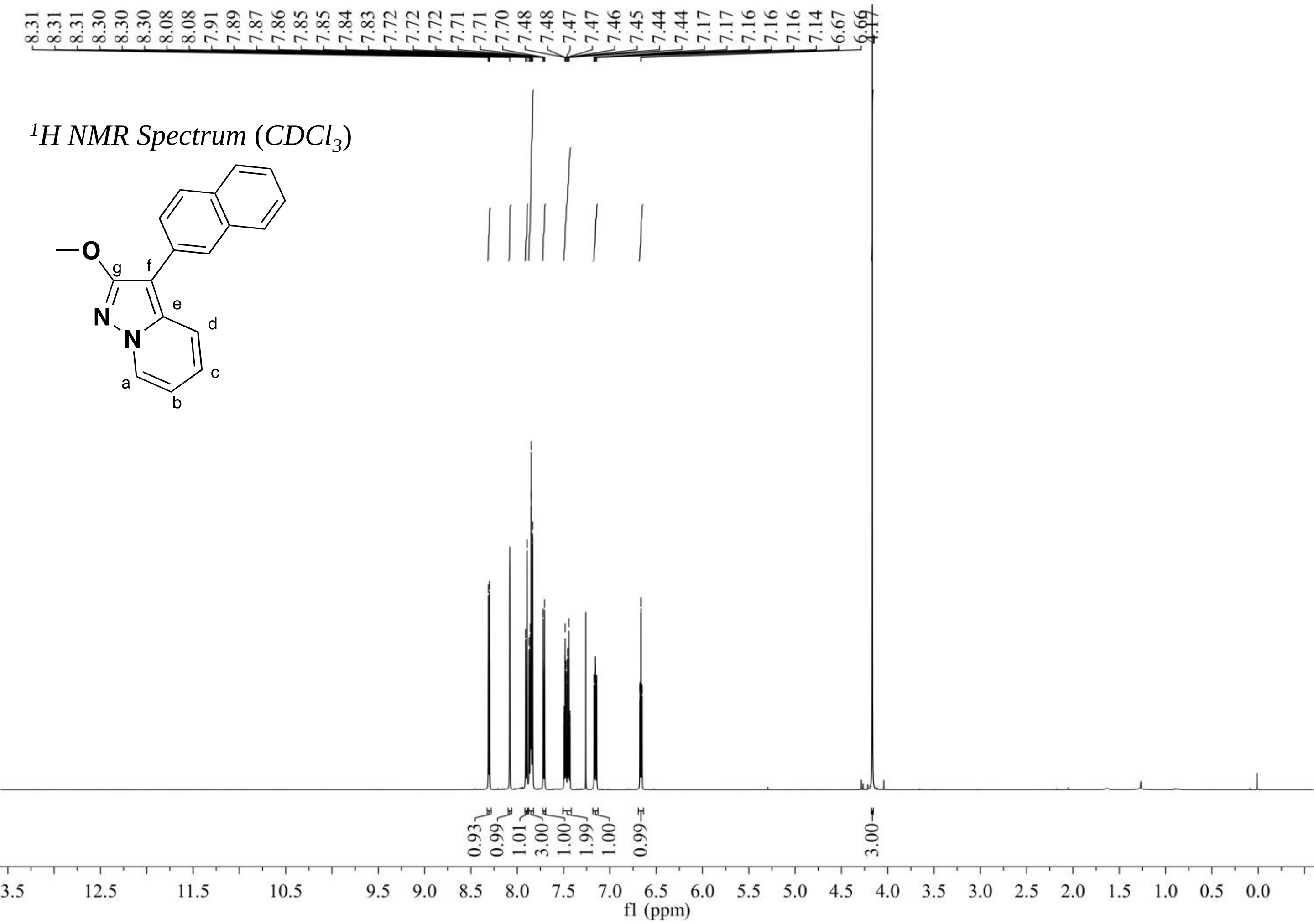

^{13}C NMR Spectrum (*$CDCl_3$*)

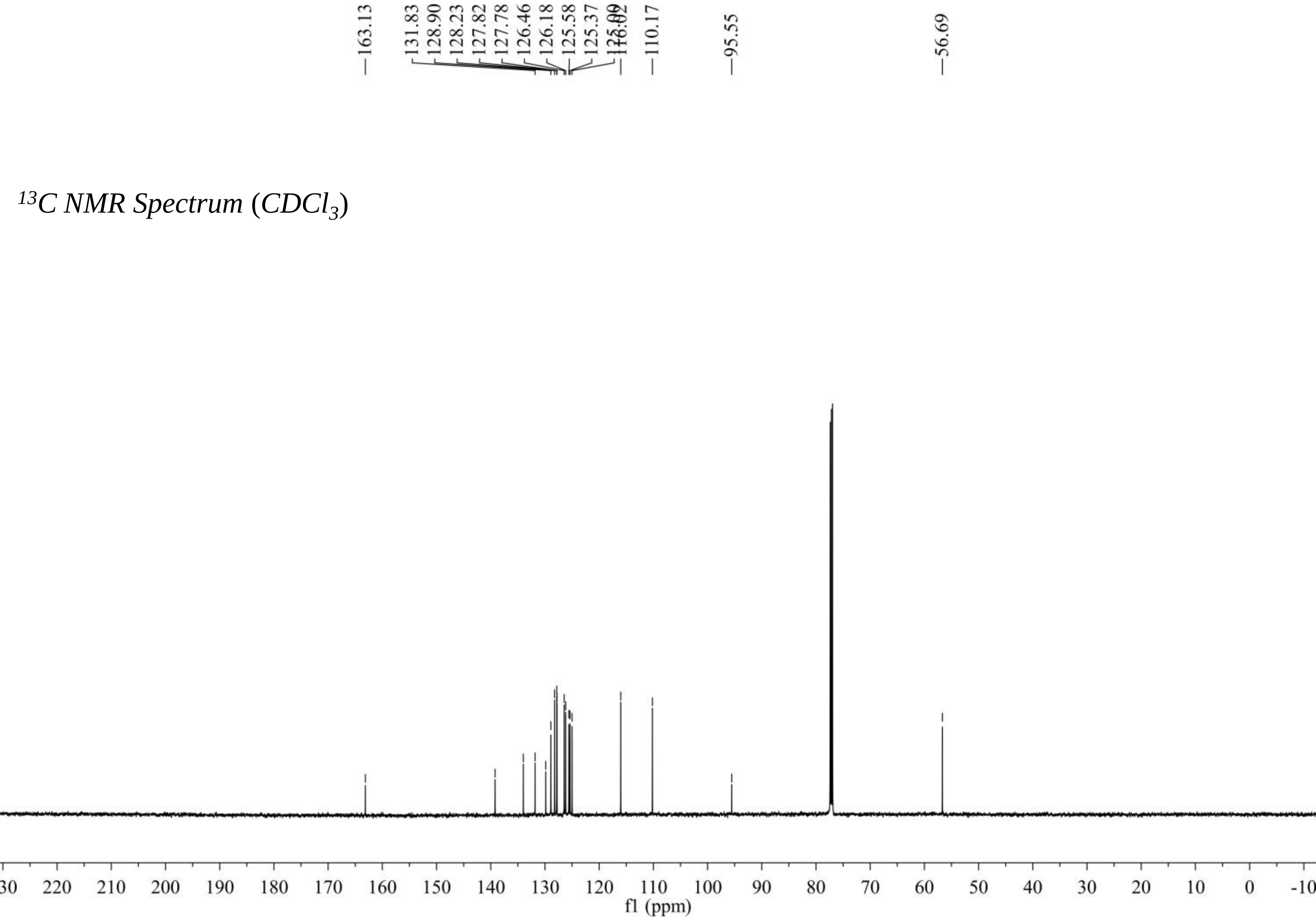

2-(2-methoxypyrazolo[1,5-a]pyridin-3-yl)quinoline (**7a**)

^{1}H NMR Spectrum (*CDCl$_3$*)

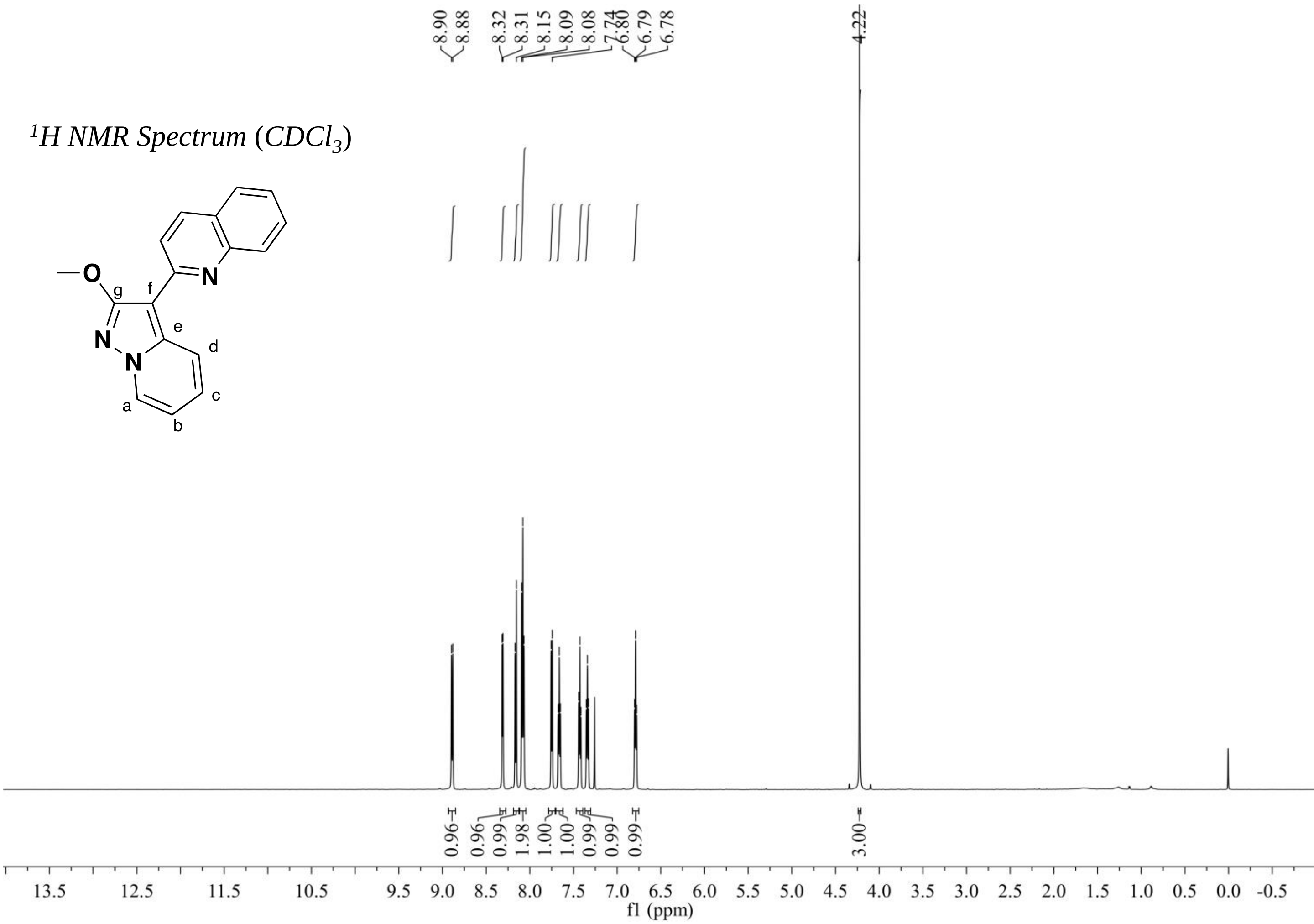

^{13}C NMR Spectrum (*CDCl$_3$*)

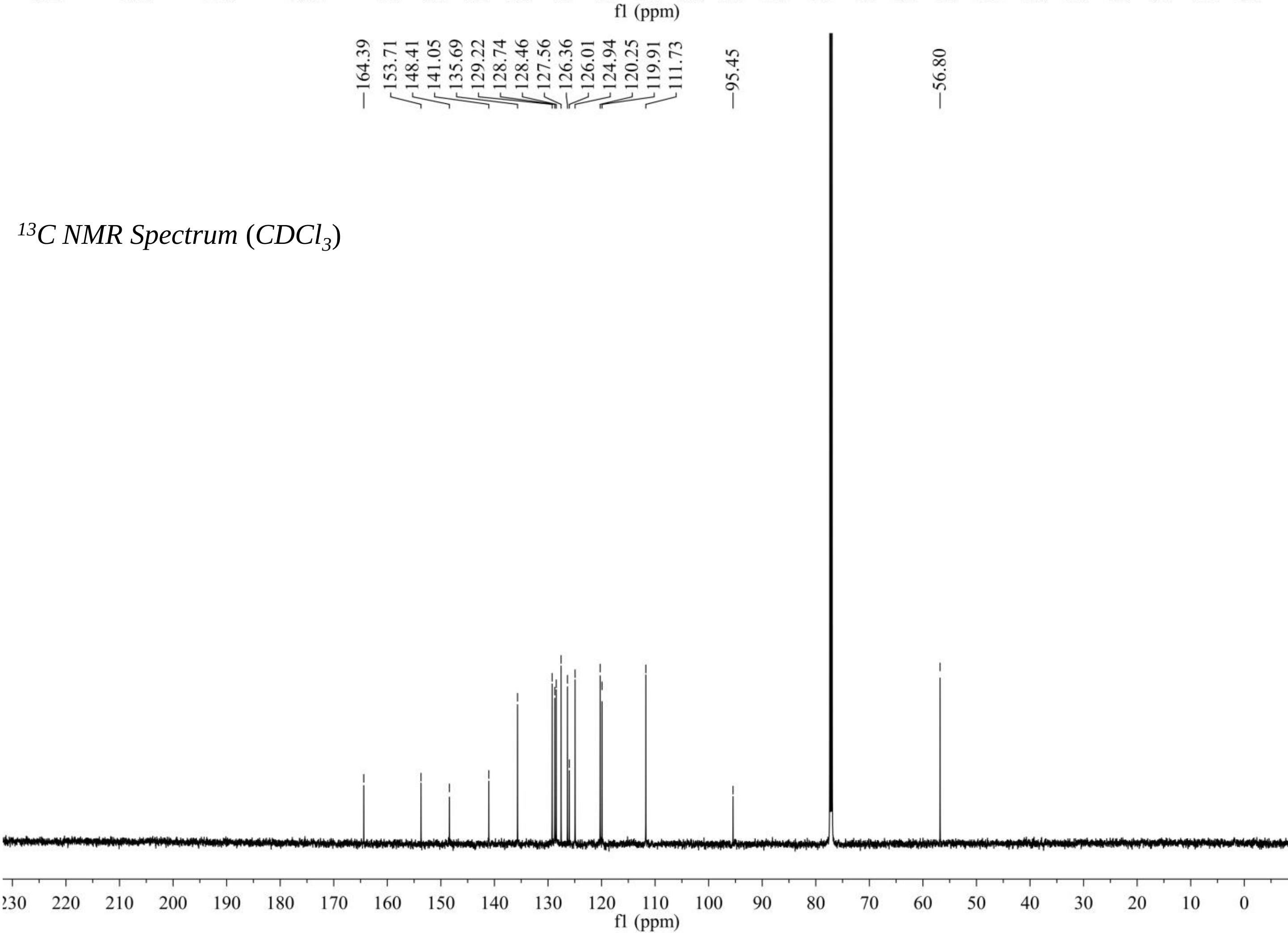

7-(2-methoxypyrazolo[1,5-a]pyridin-3-yl)quinoline (**7b**)

^{1}H NMR Spectrum ($CDCl_3$)

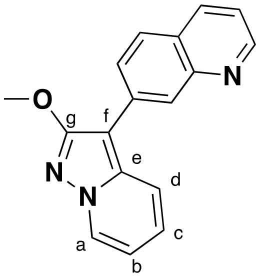

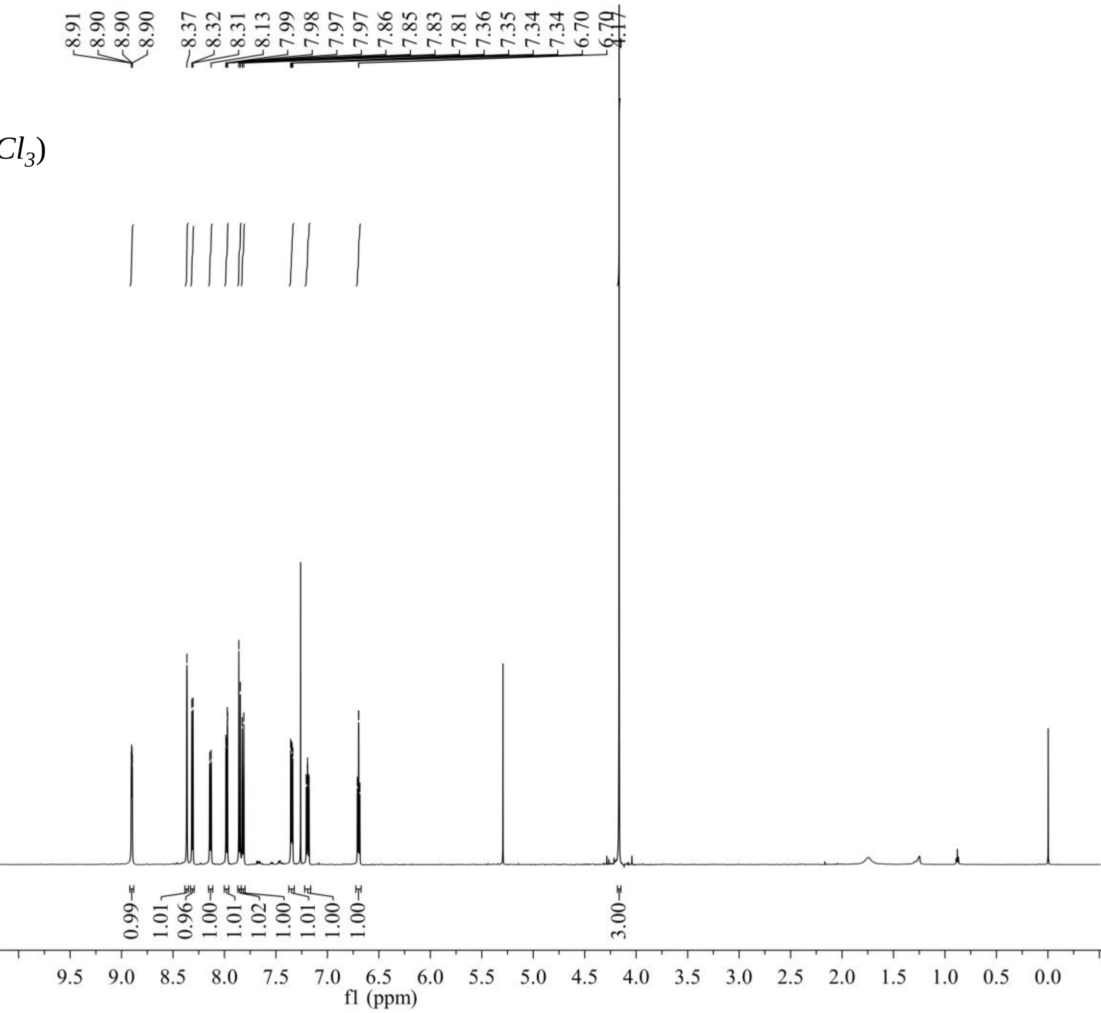

^{13}C NMR Spectrum ($CDCl_3$)

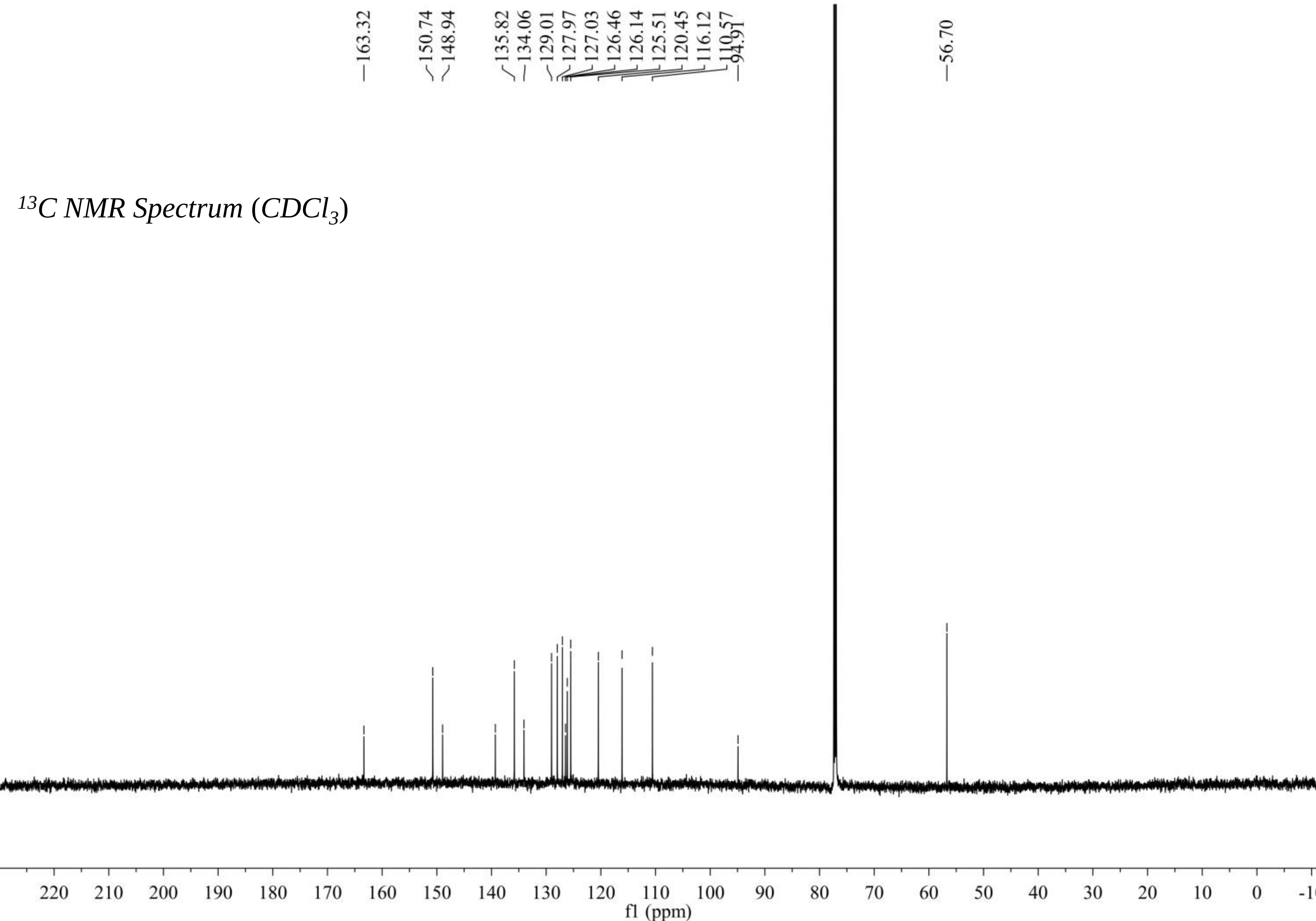

6-(2-methoxypyrazolo[1,5-a]pyridin-3-yl)quinoline (**7c**)

^{1}H NMR Spectrum ($CDCl_3$)

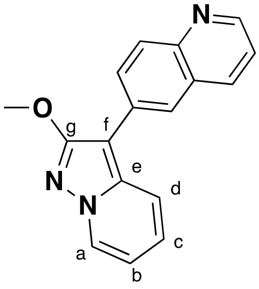

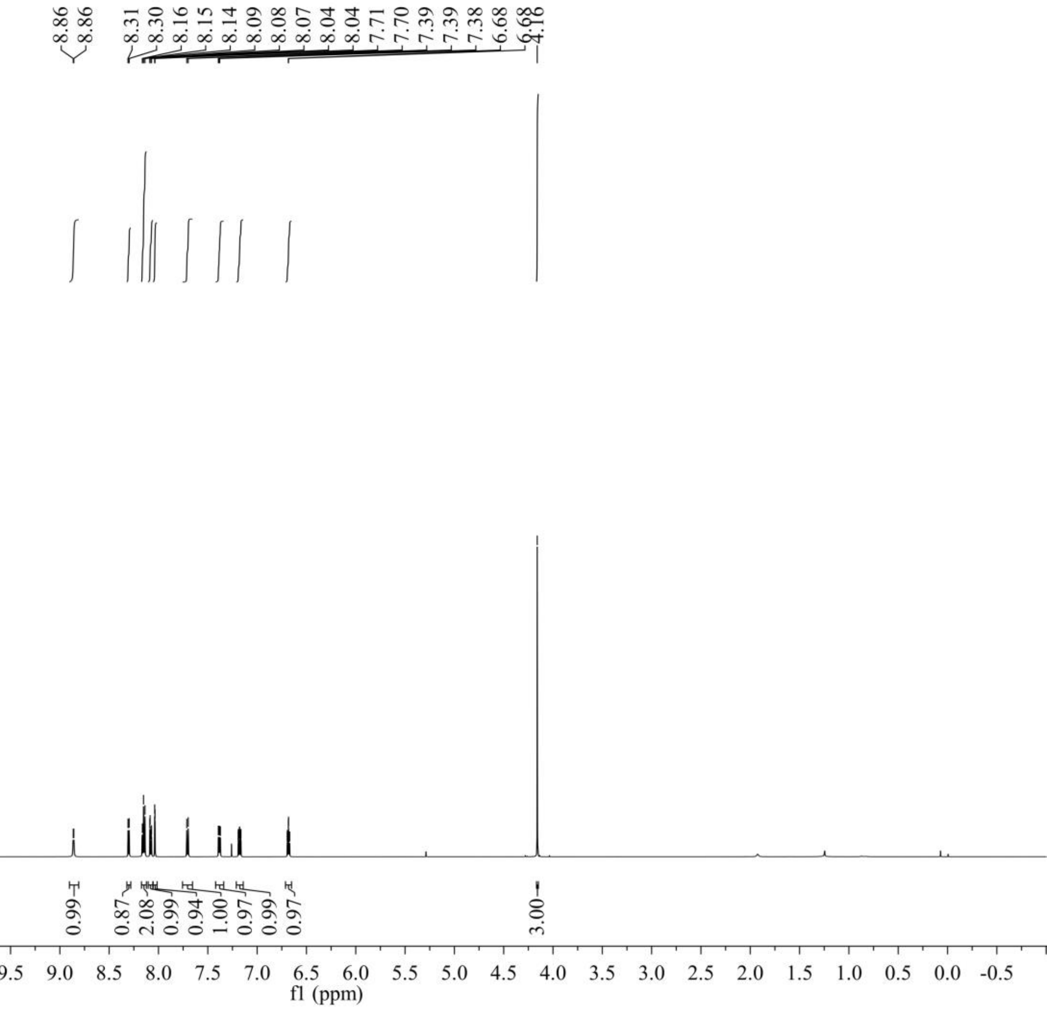

^{13}C NMR Spectrum ($CDCl_3$)

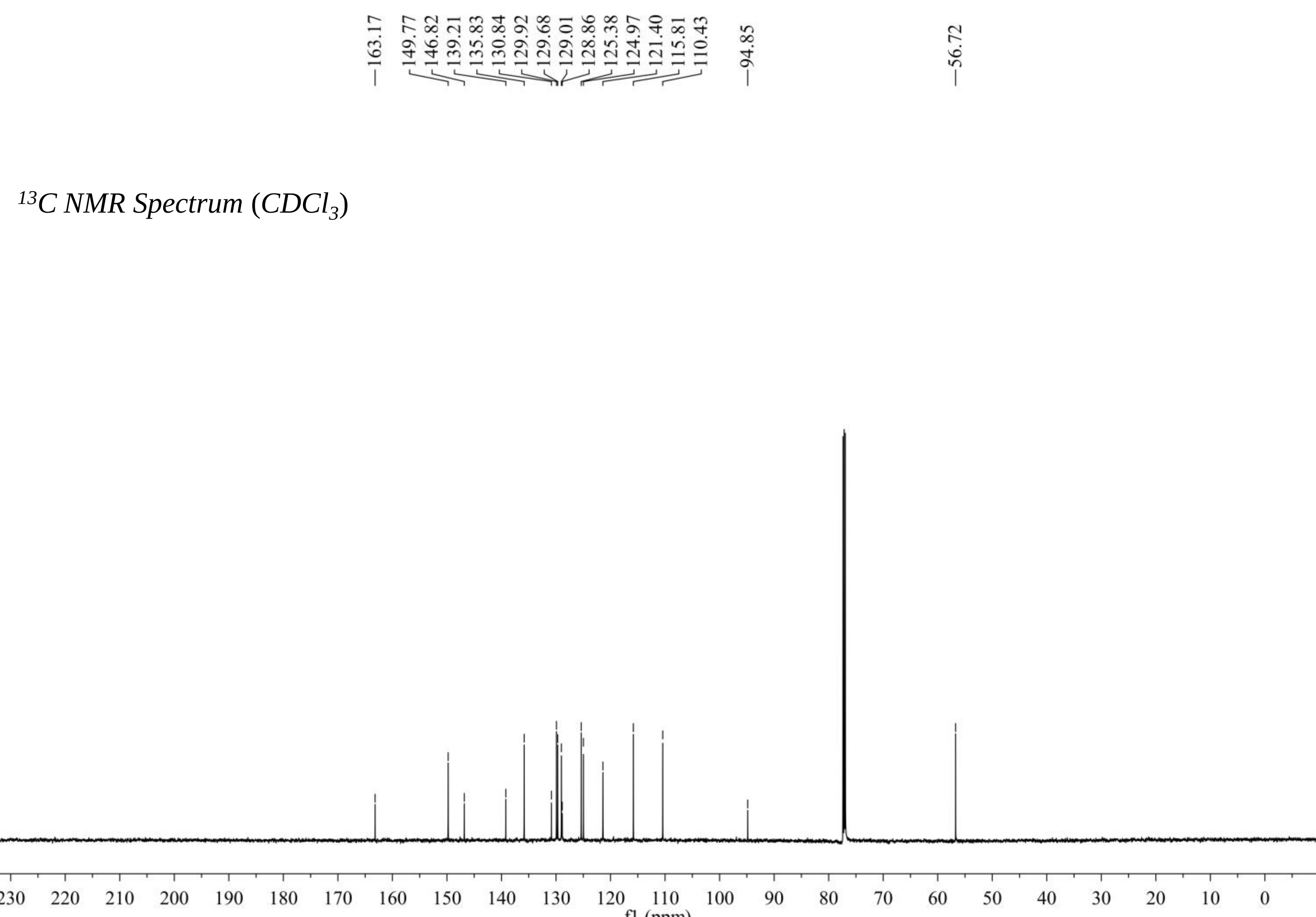

Ethyl 2-(benzyloxy)-7-chloropyrazolo[1,5-a]pyridine-3-carboxylate (**21**)

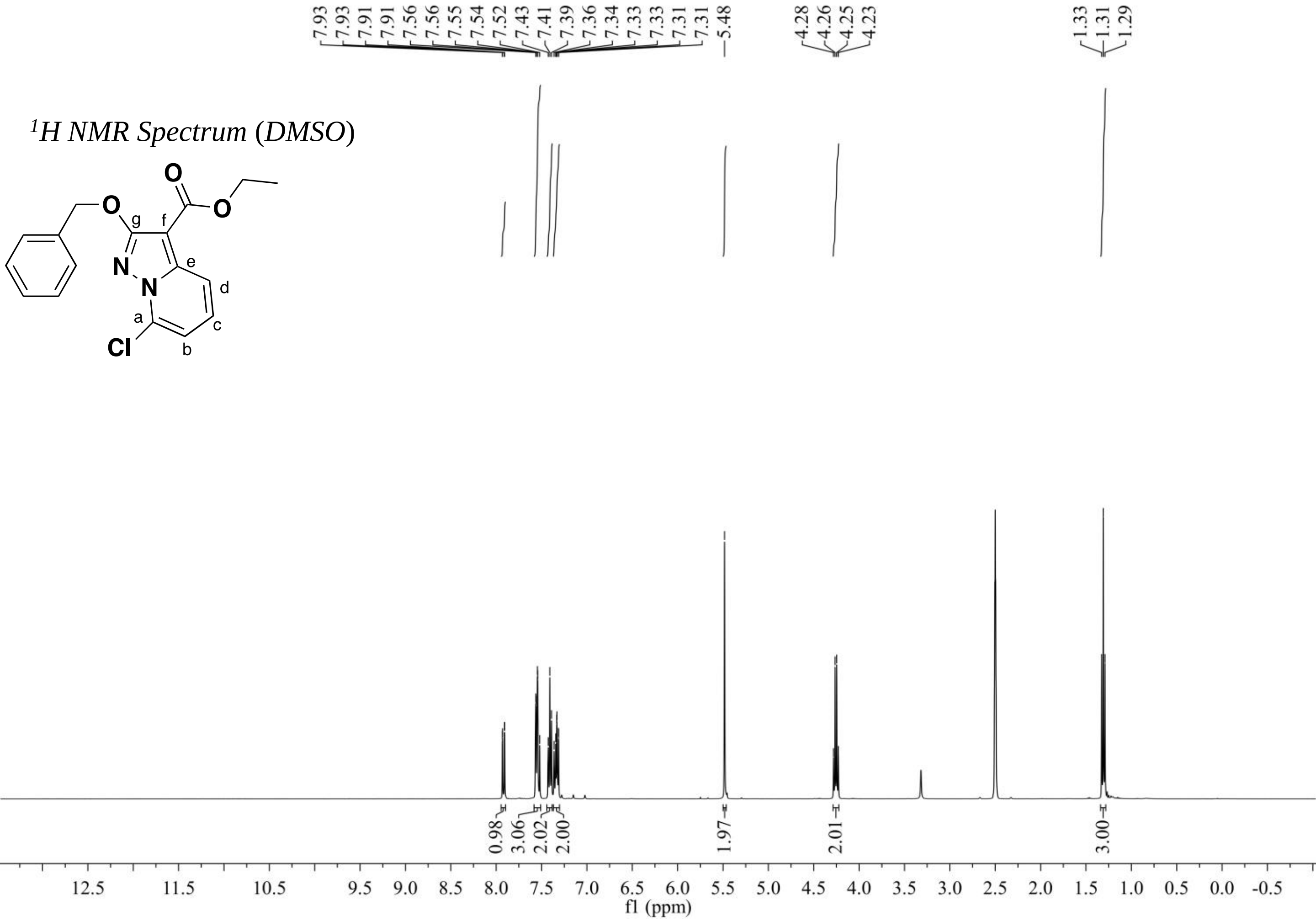

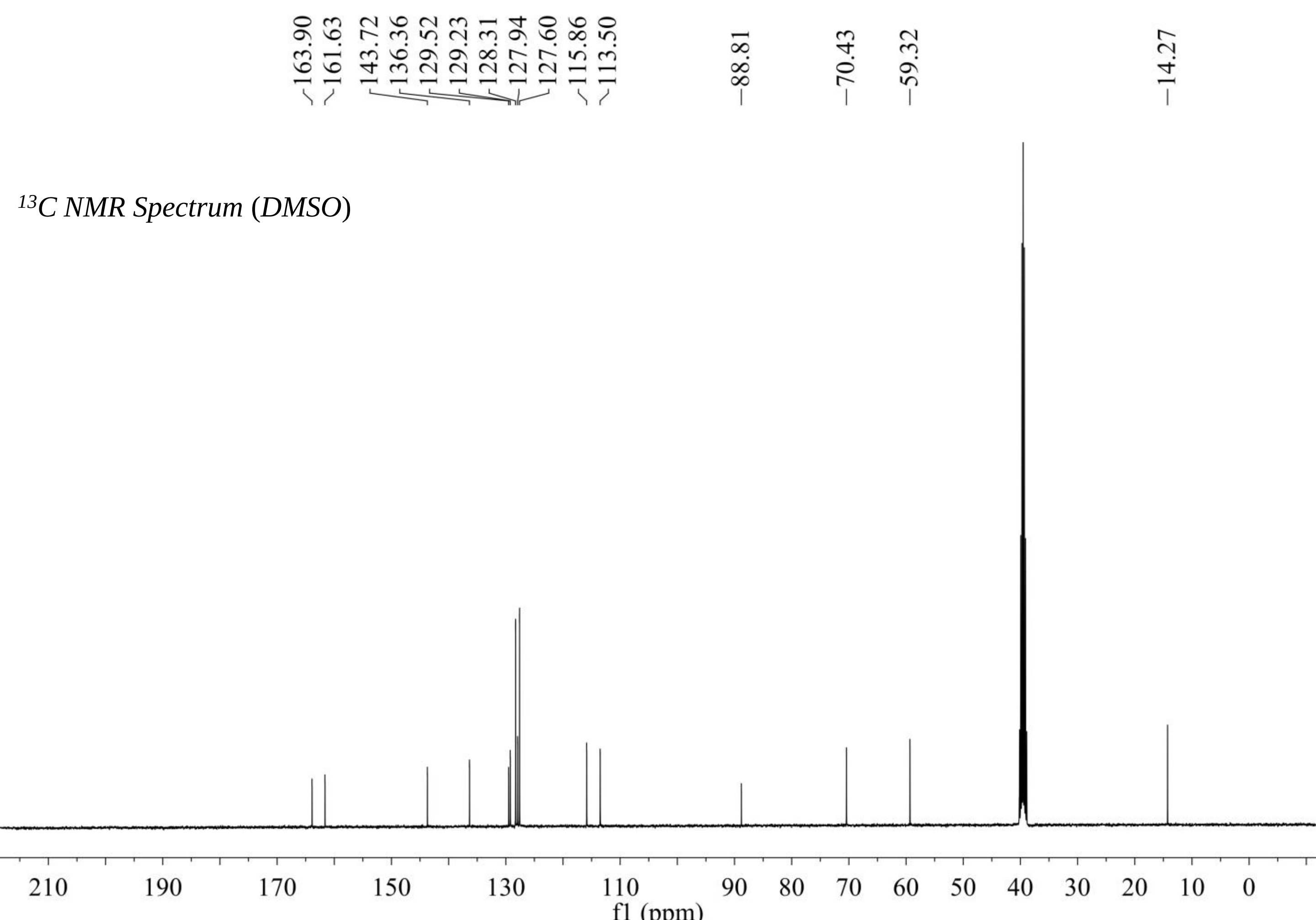

Ethyl 2-(benzyloxy)-7-chloropyrazolo[1,5-a]pyridine-3-carboxylate (**21**)

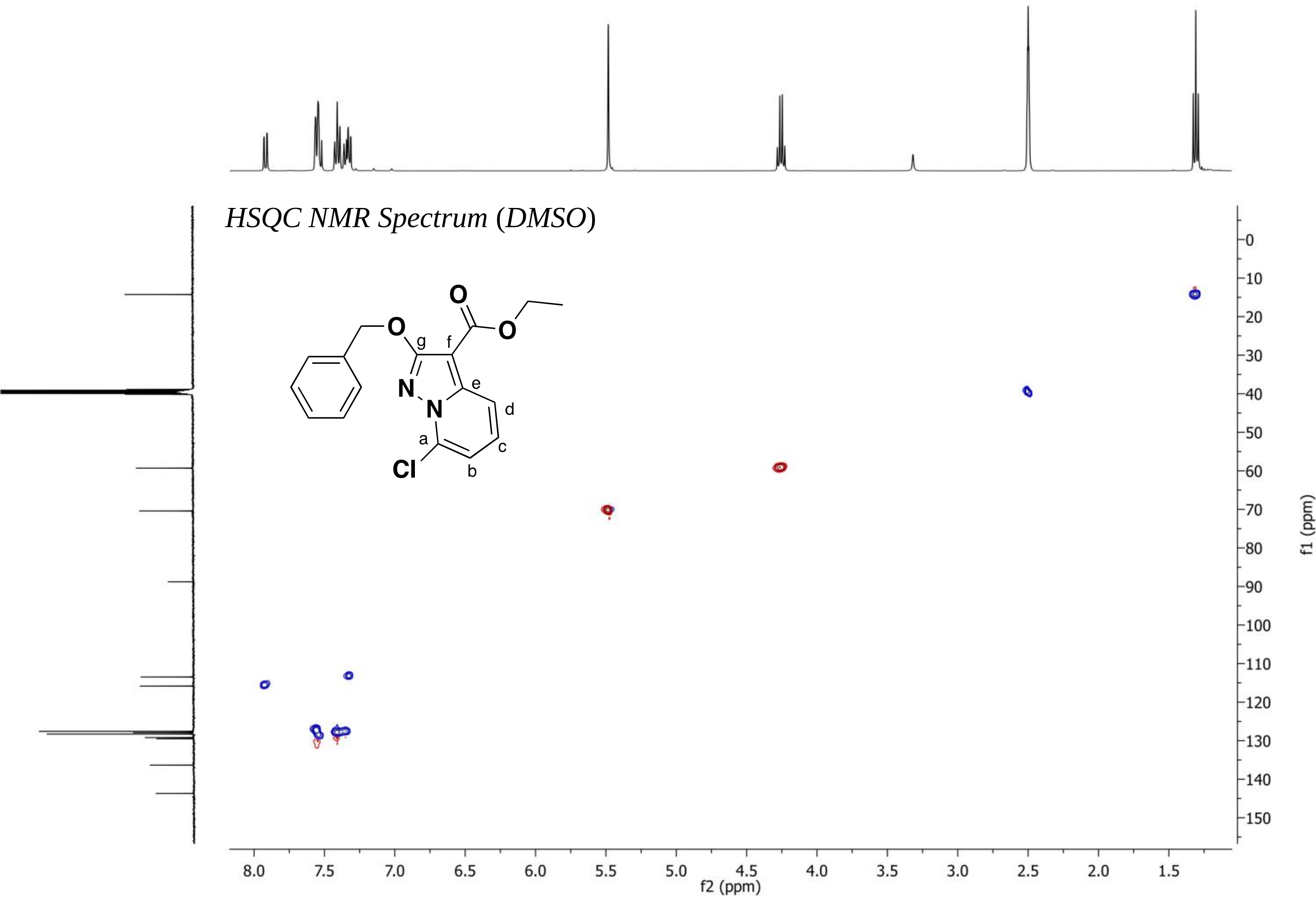

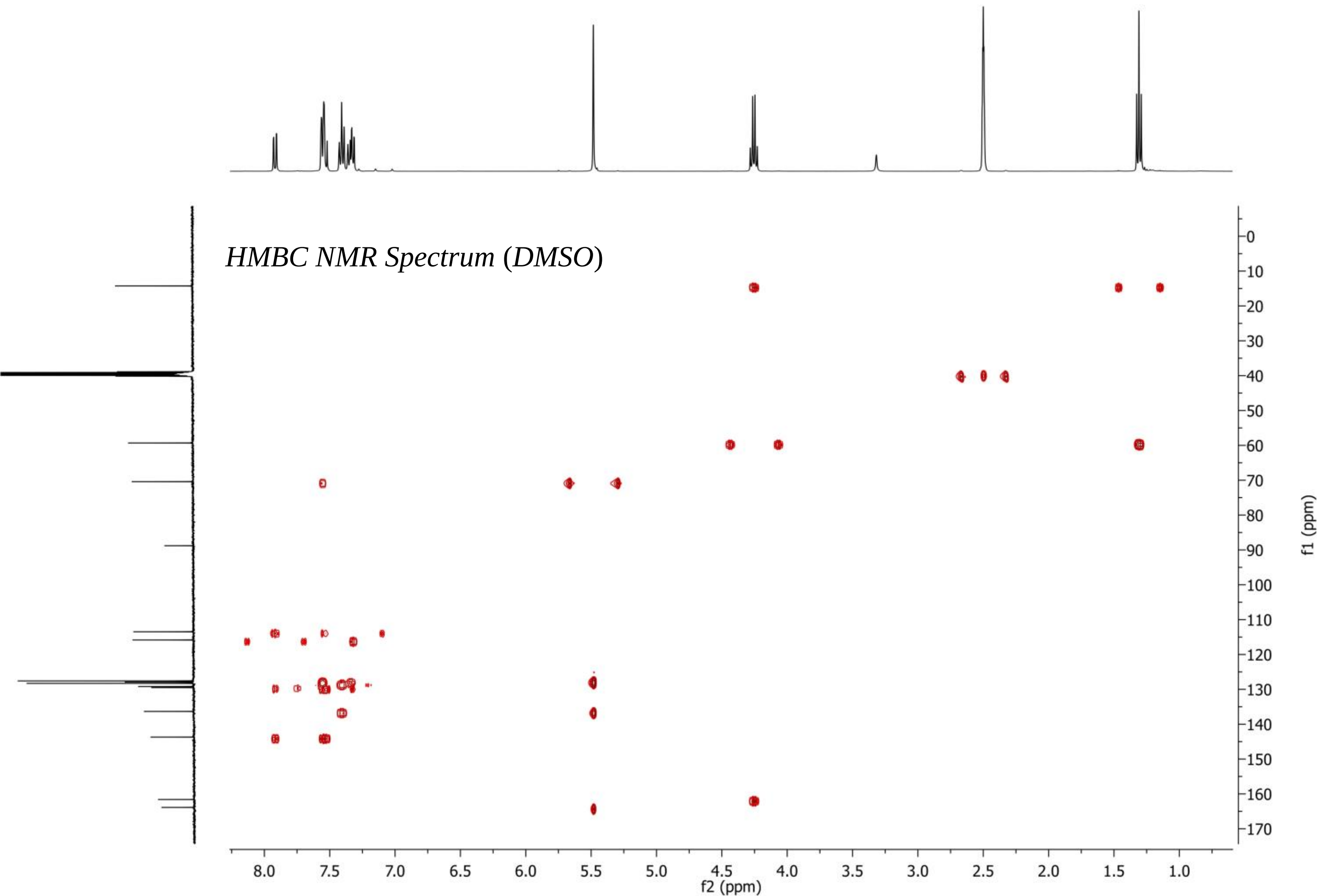

Ethyl 2-(benzyloxy)-7-(dimethylamino)pyrazolo[1,5-a]pyridine-3-carboxylate (**22**)

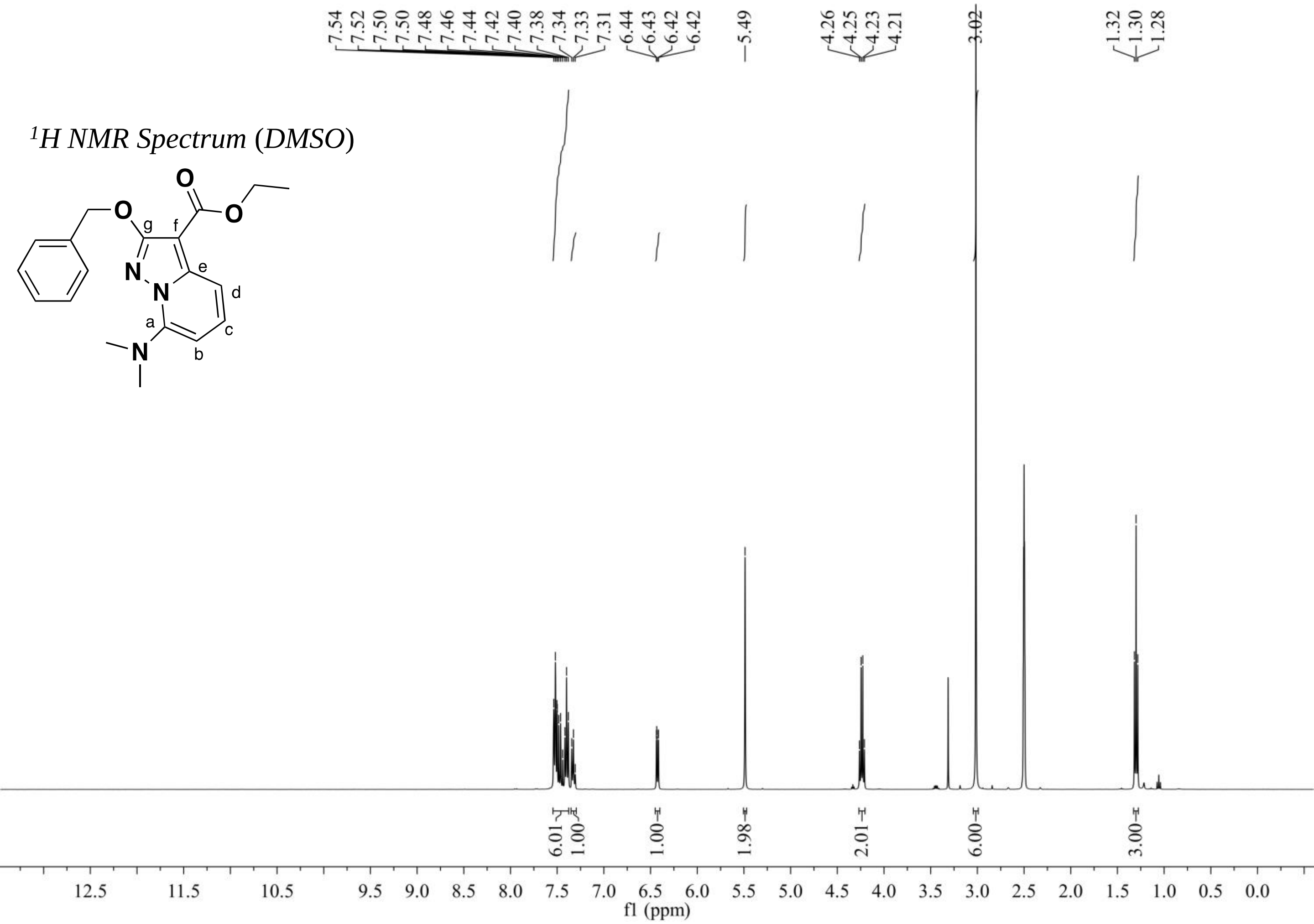

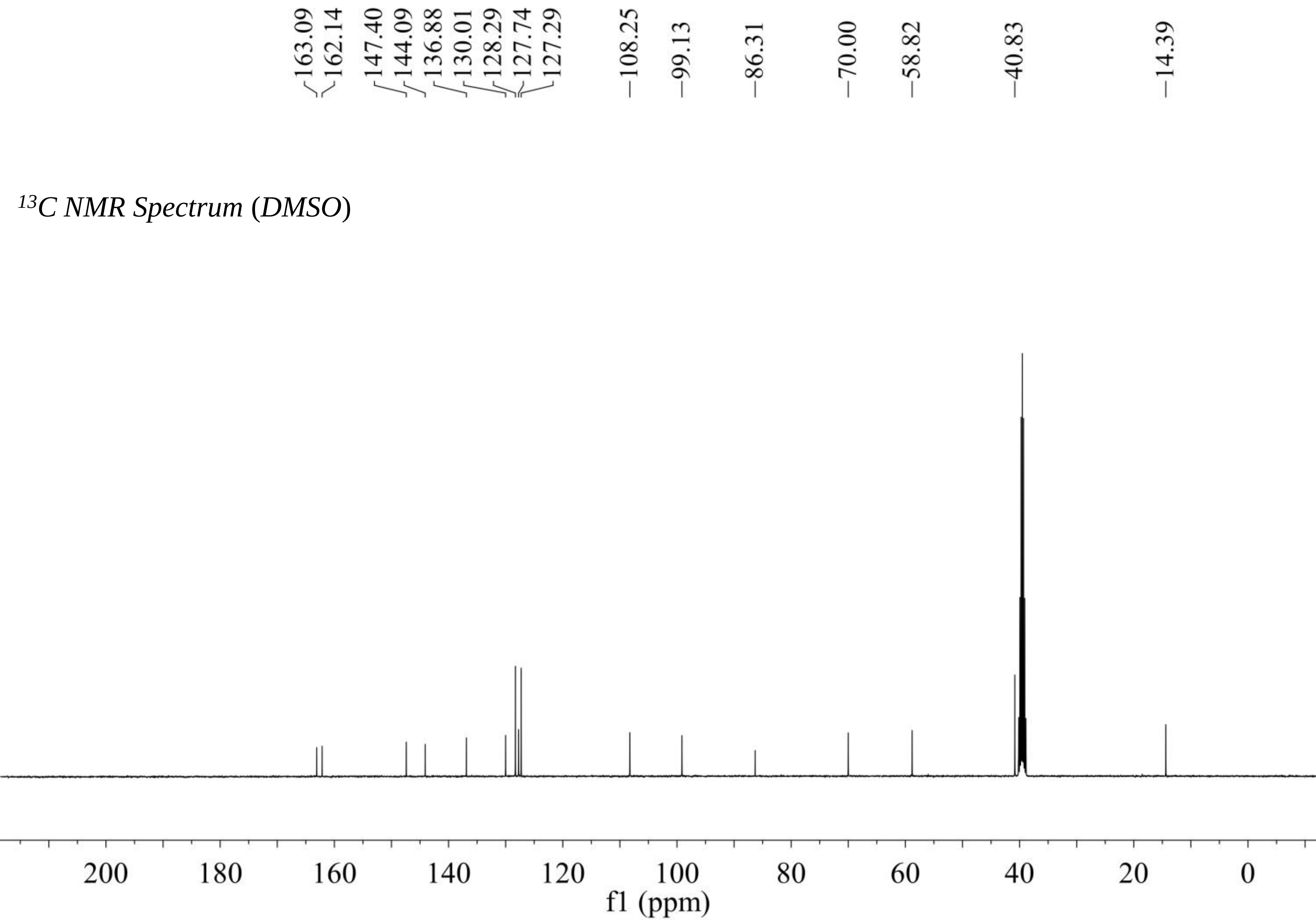

Ethyl 2-(benzyloxy)-7-(dimethylamino)pyrazolo[1,5-a]pyridine-3-carboxylate (**22**)

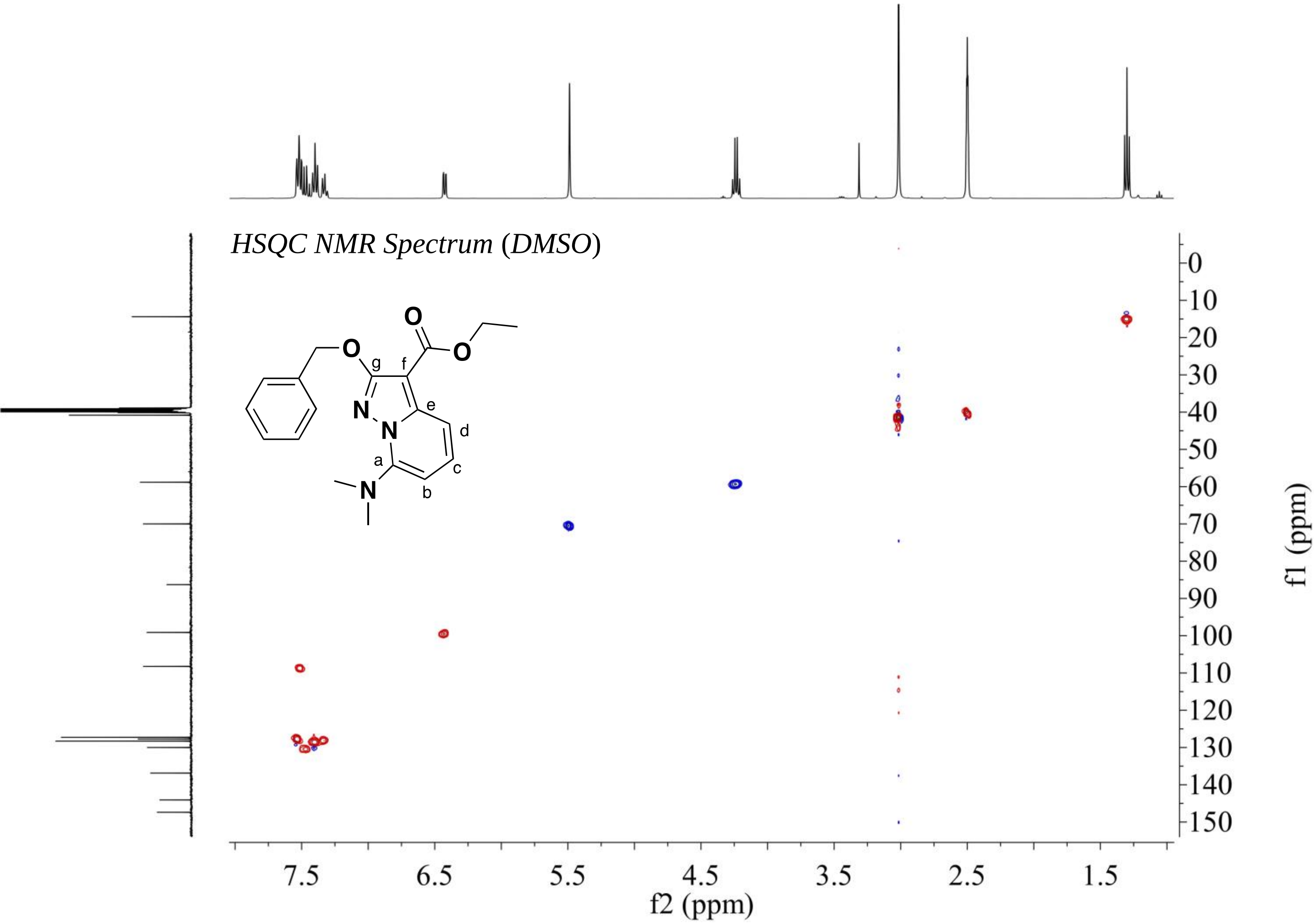

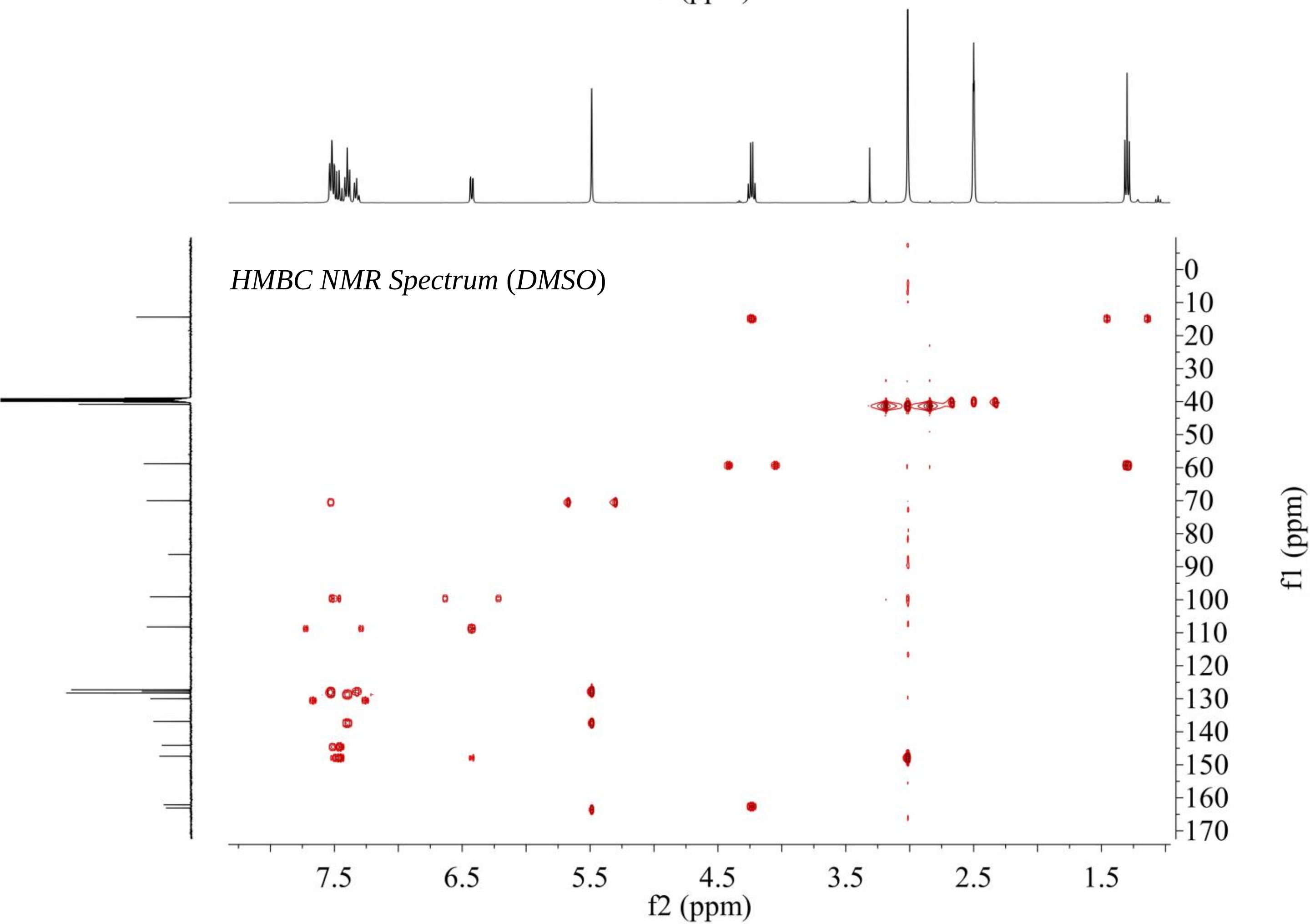

Ethyl 7-(dimethylamino)-2-hydroxypyrazolo[1,5-a]pyridine-3-carboxylate (**8a**)

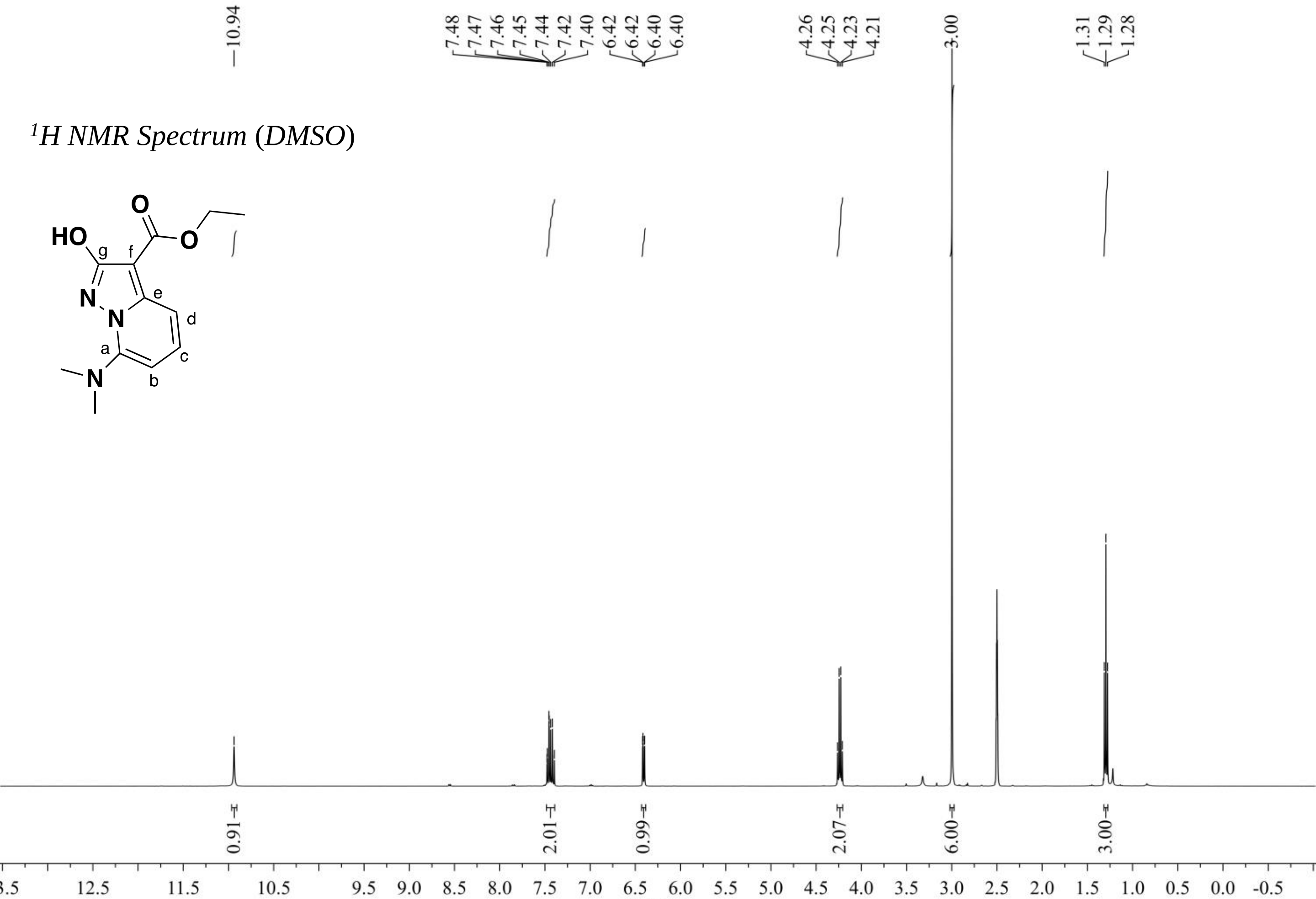

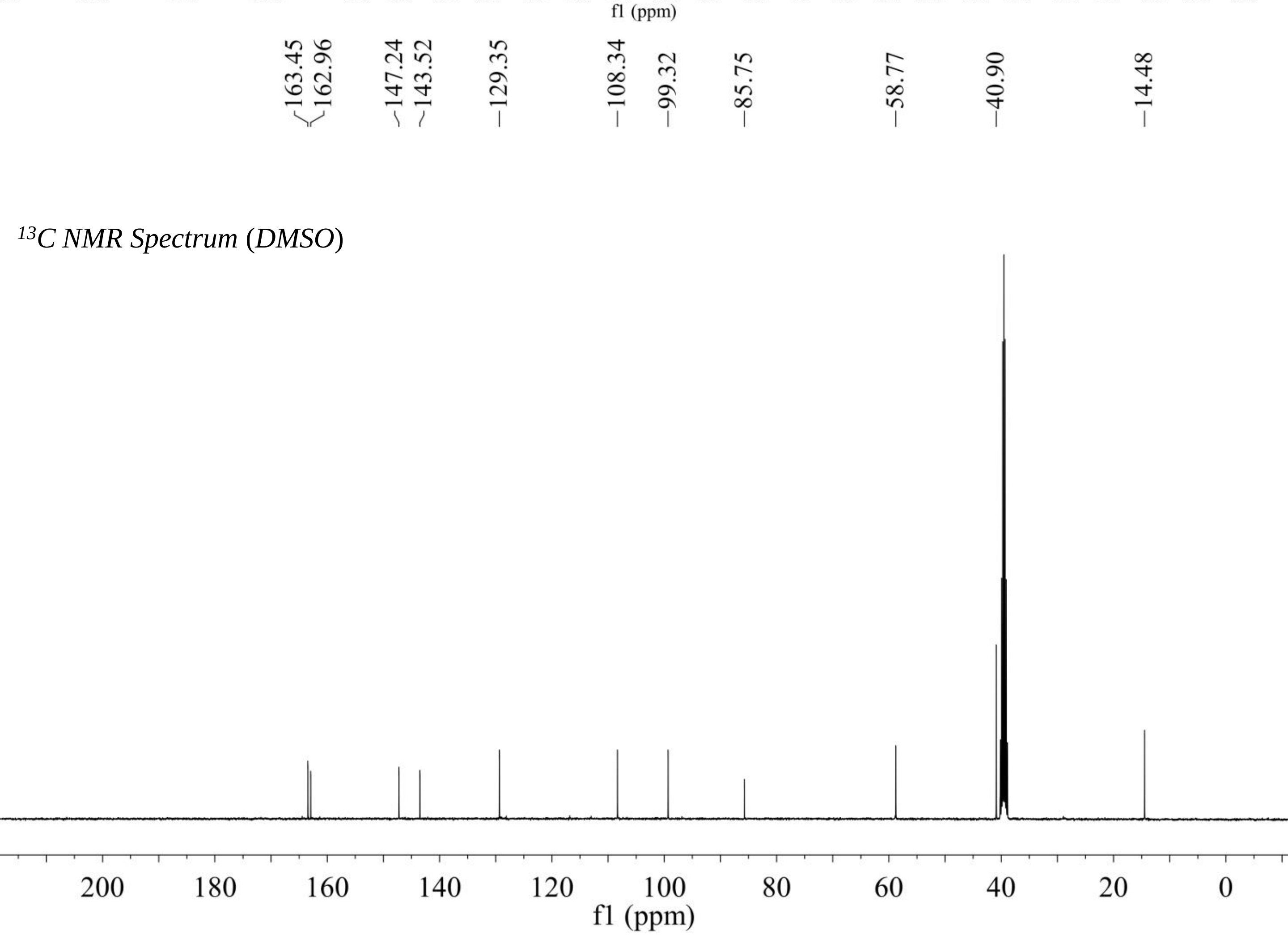

Ethyl 7-(dimethylamino)-2-hydroxypyrazolo[1,5-a]pyridine-3-carboxylate (**8a**)

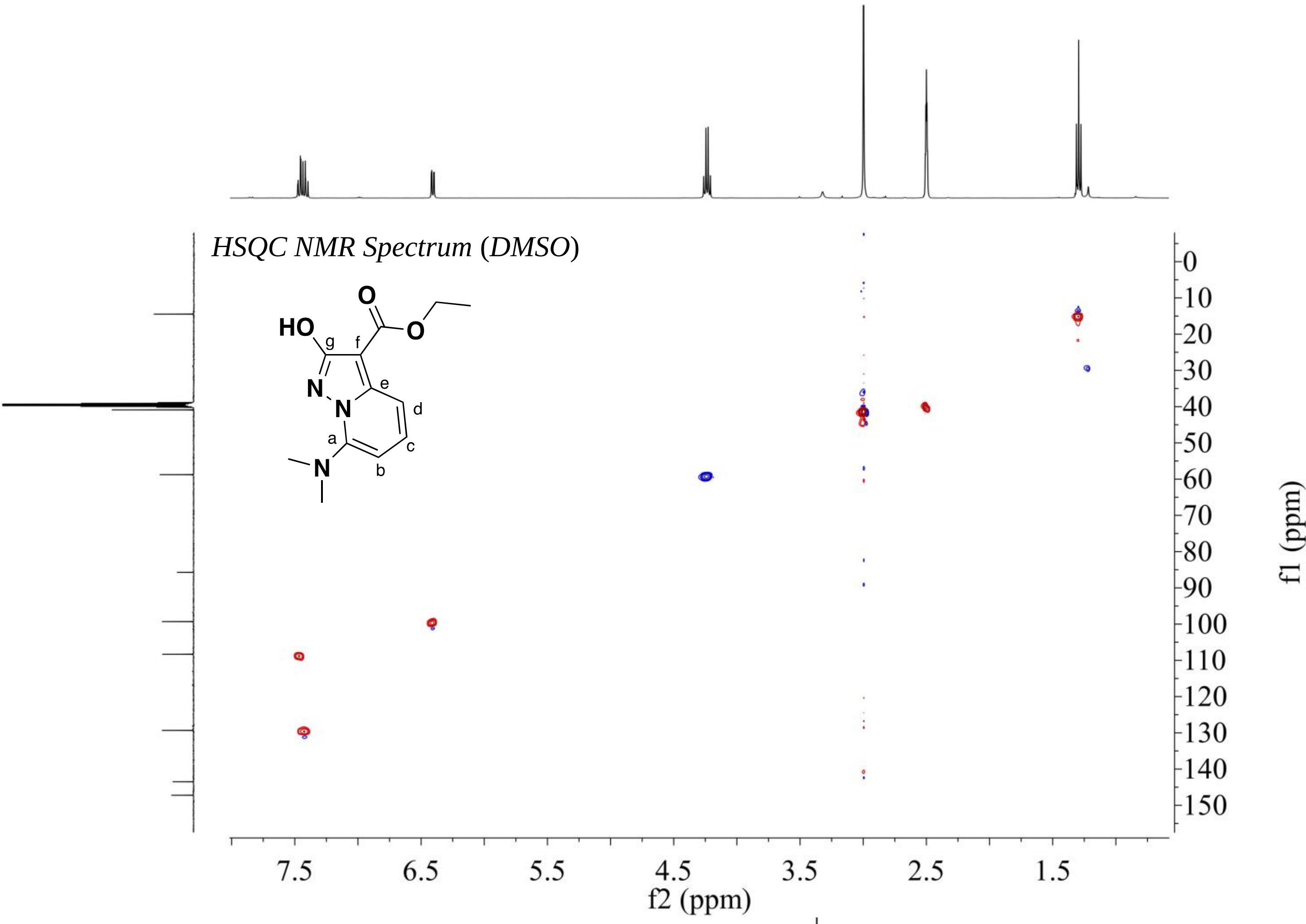

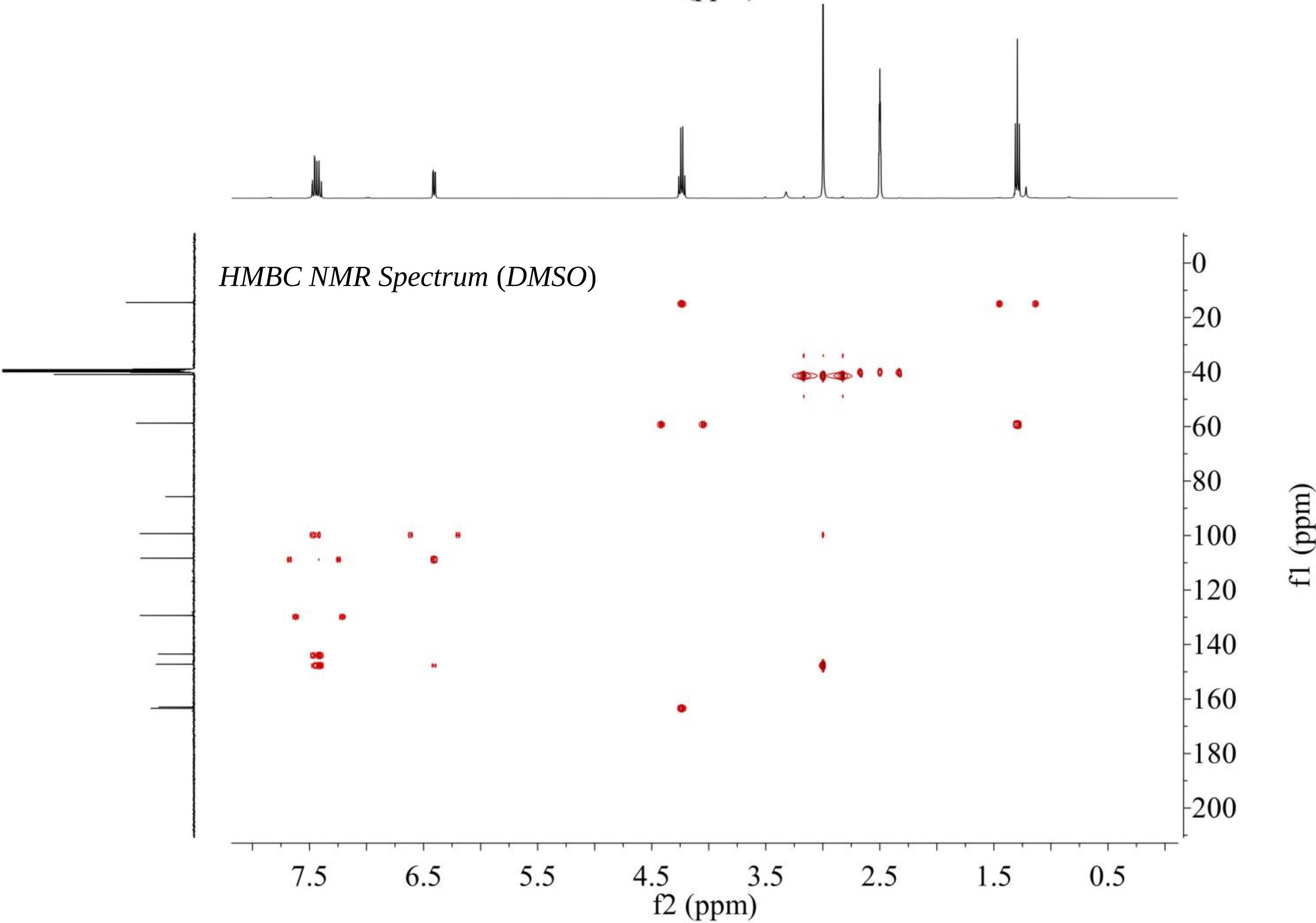

Ethyl 7-(dimethylamino)-2-hydroxy-6-nitropyrazolo[1,5-a]pyridine-3-carboxylate (**8b**)

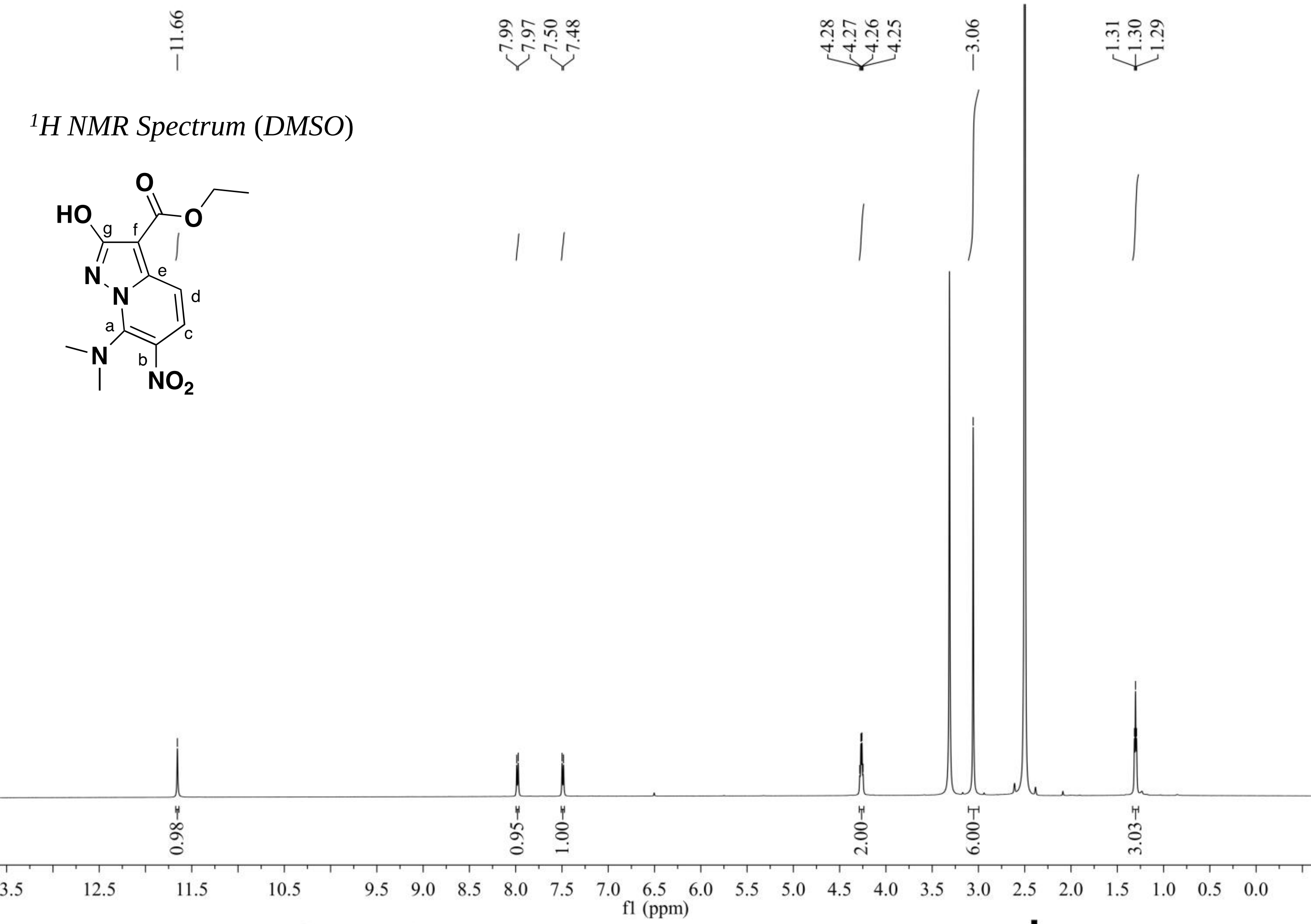

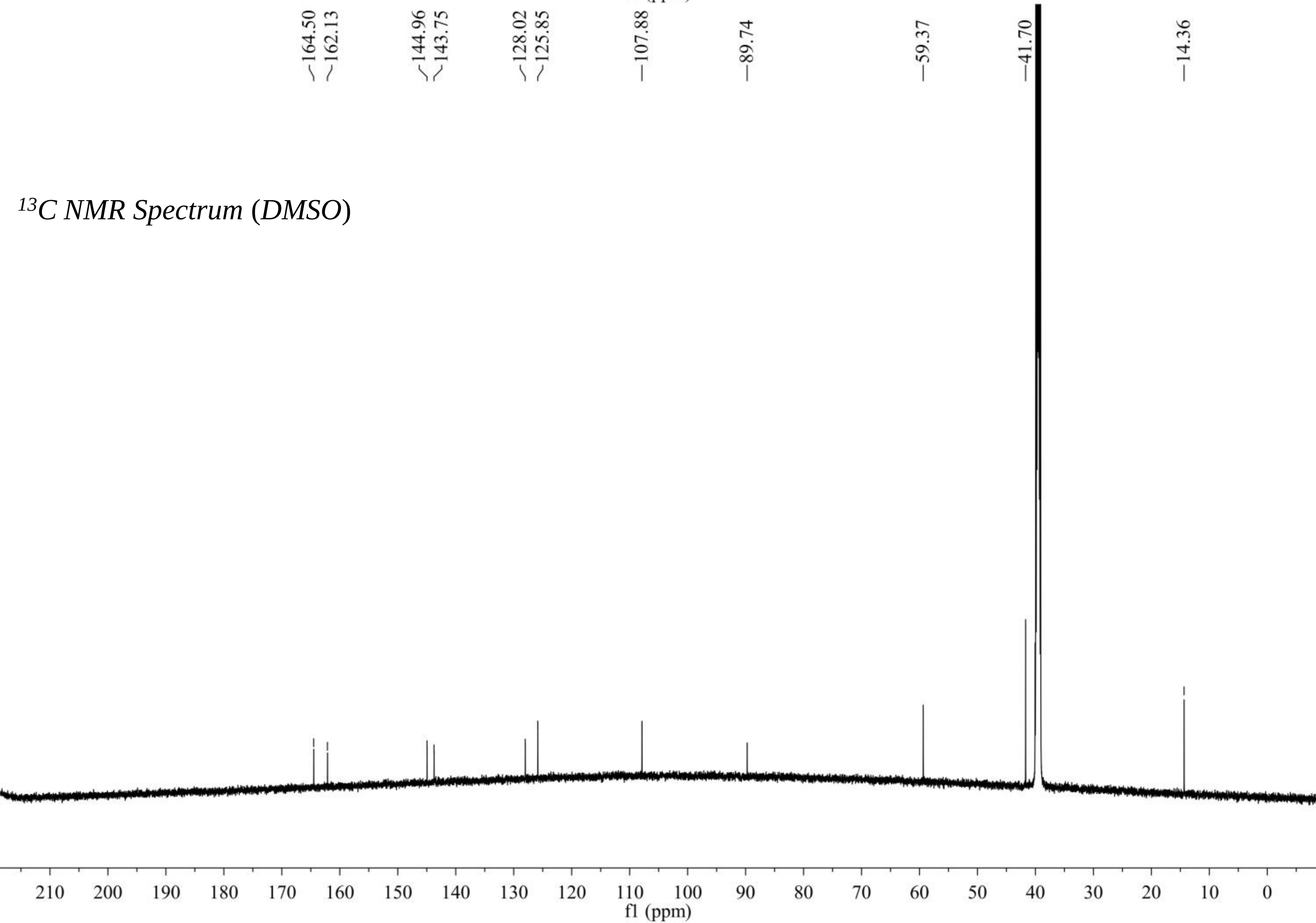

Ethyl 7-(dimethylamino)-2-hydroxy-6-nitropyrazolo[1,5-a]pyridine-3-carboxylate (**8b**)

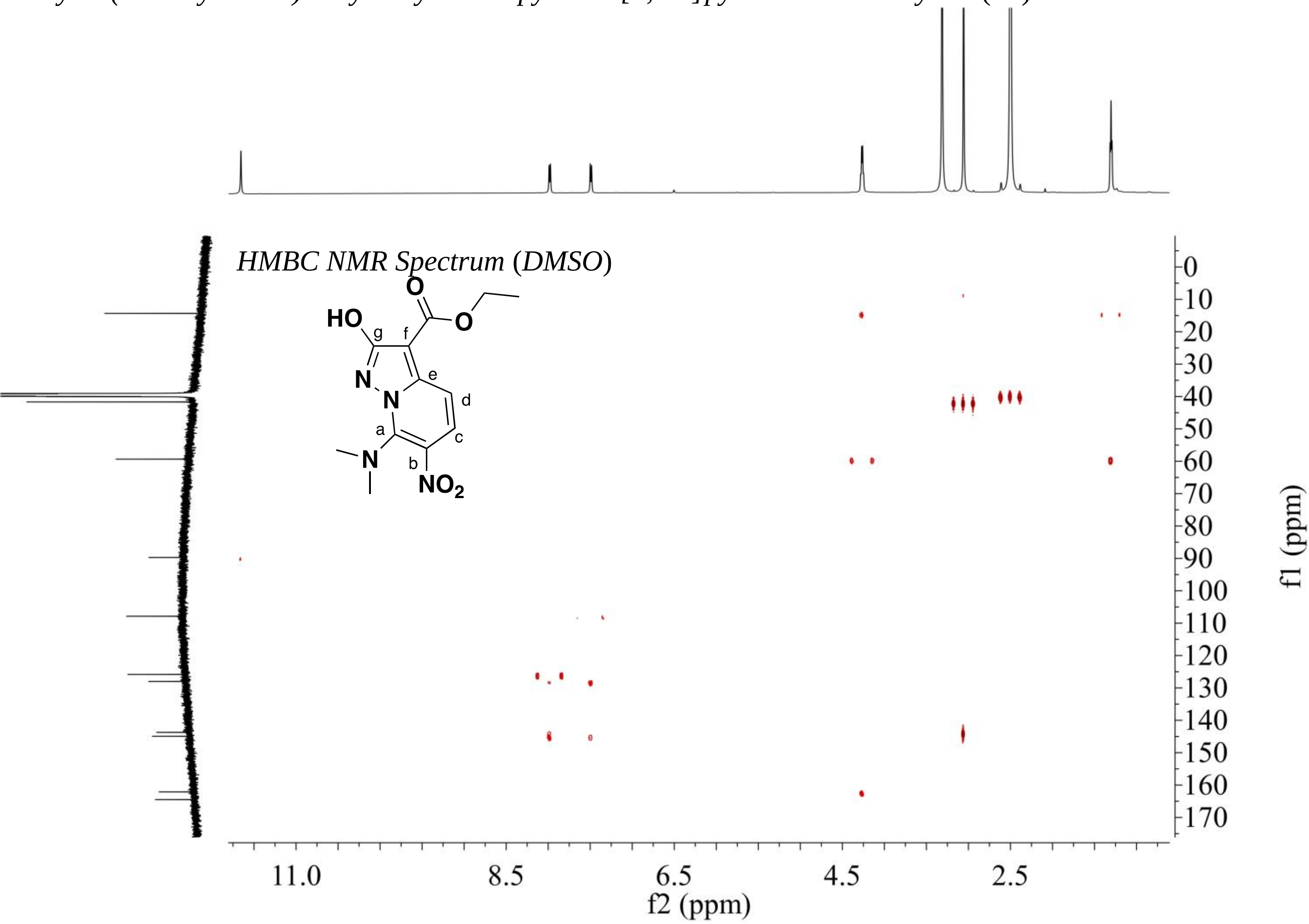

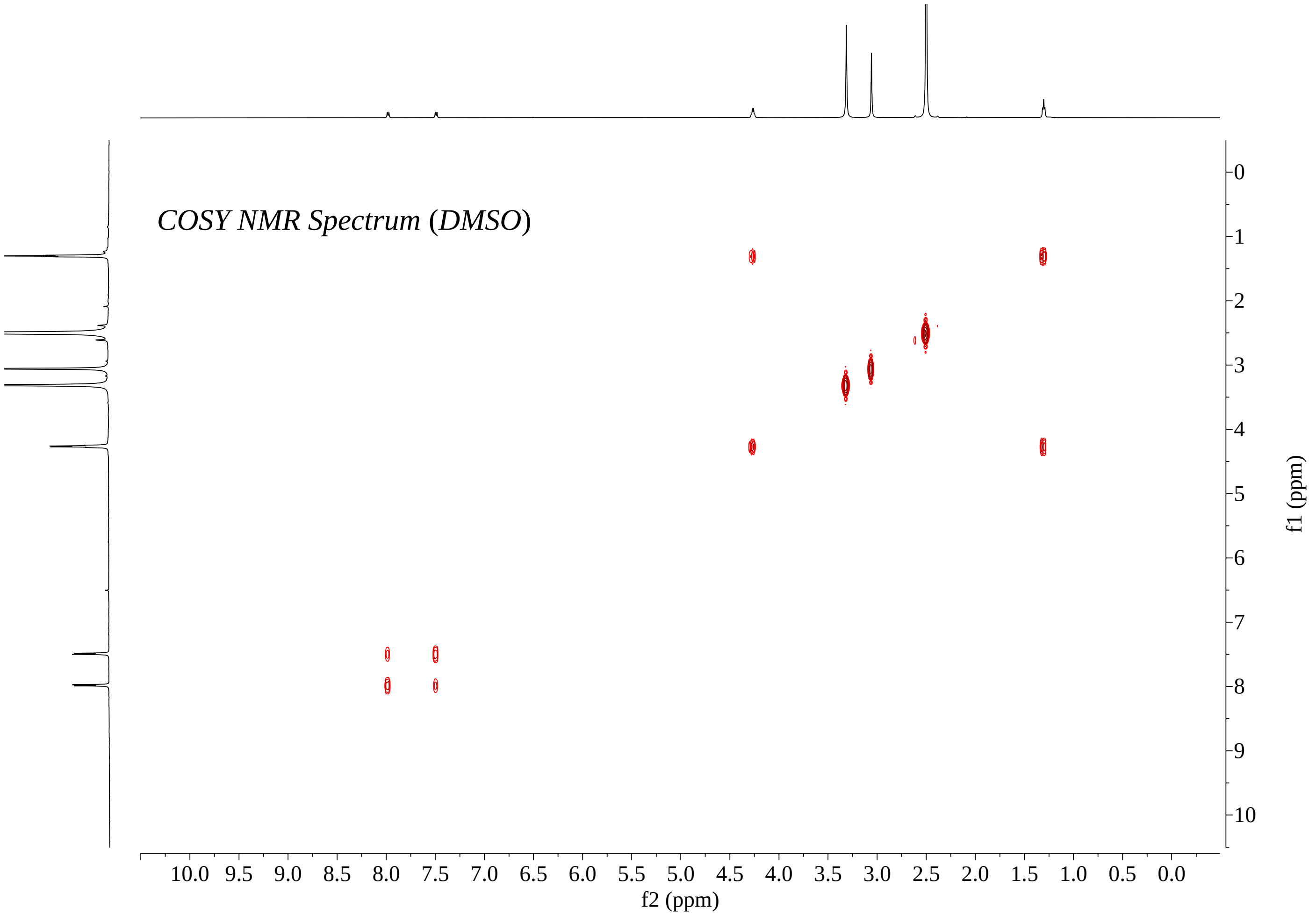

Ethyl 7-(dimethylamino)-2-hydroxy-6-nitropyrazolo[1,5-a]pyridine-3-carboxylate (**8b**)

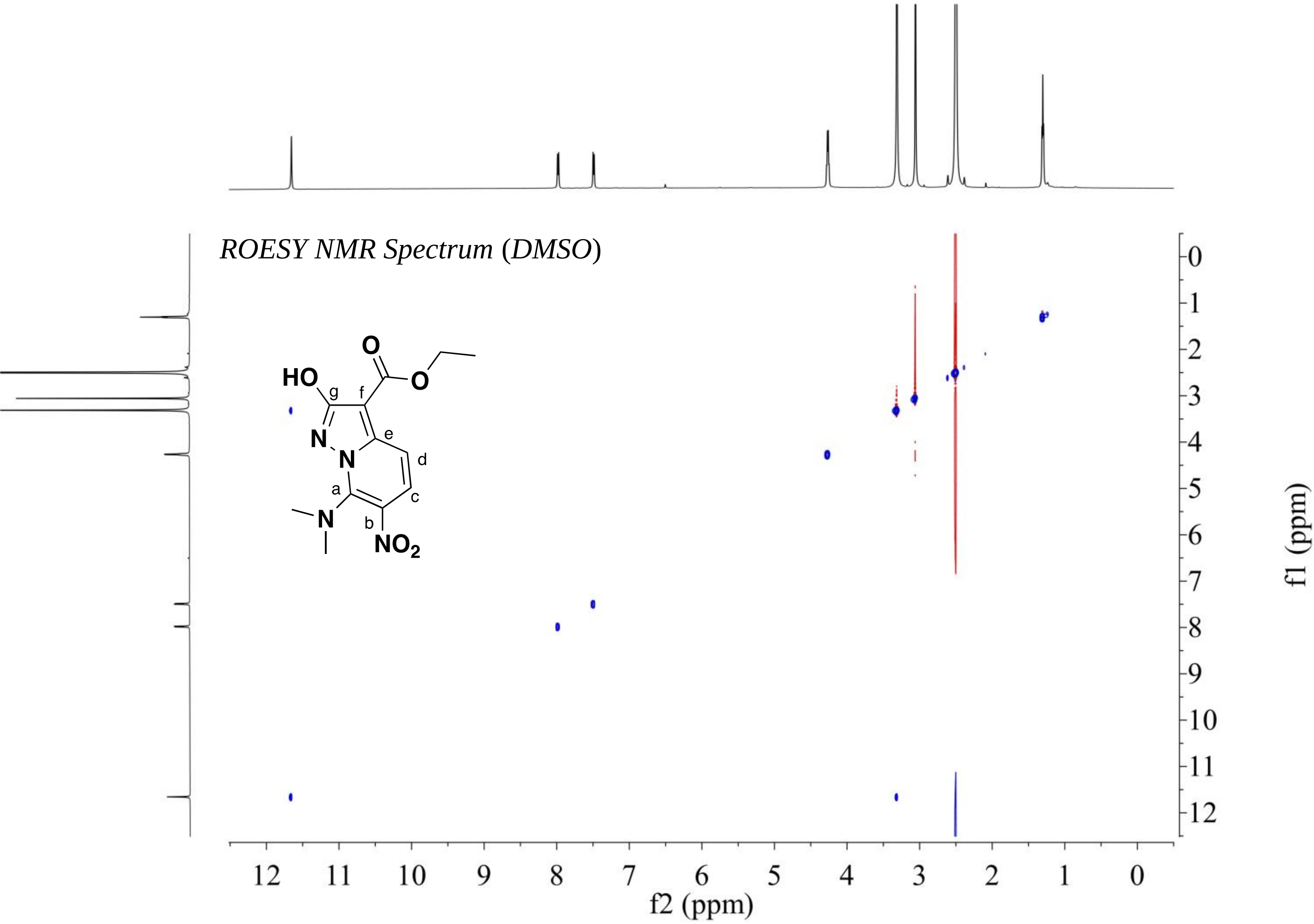

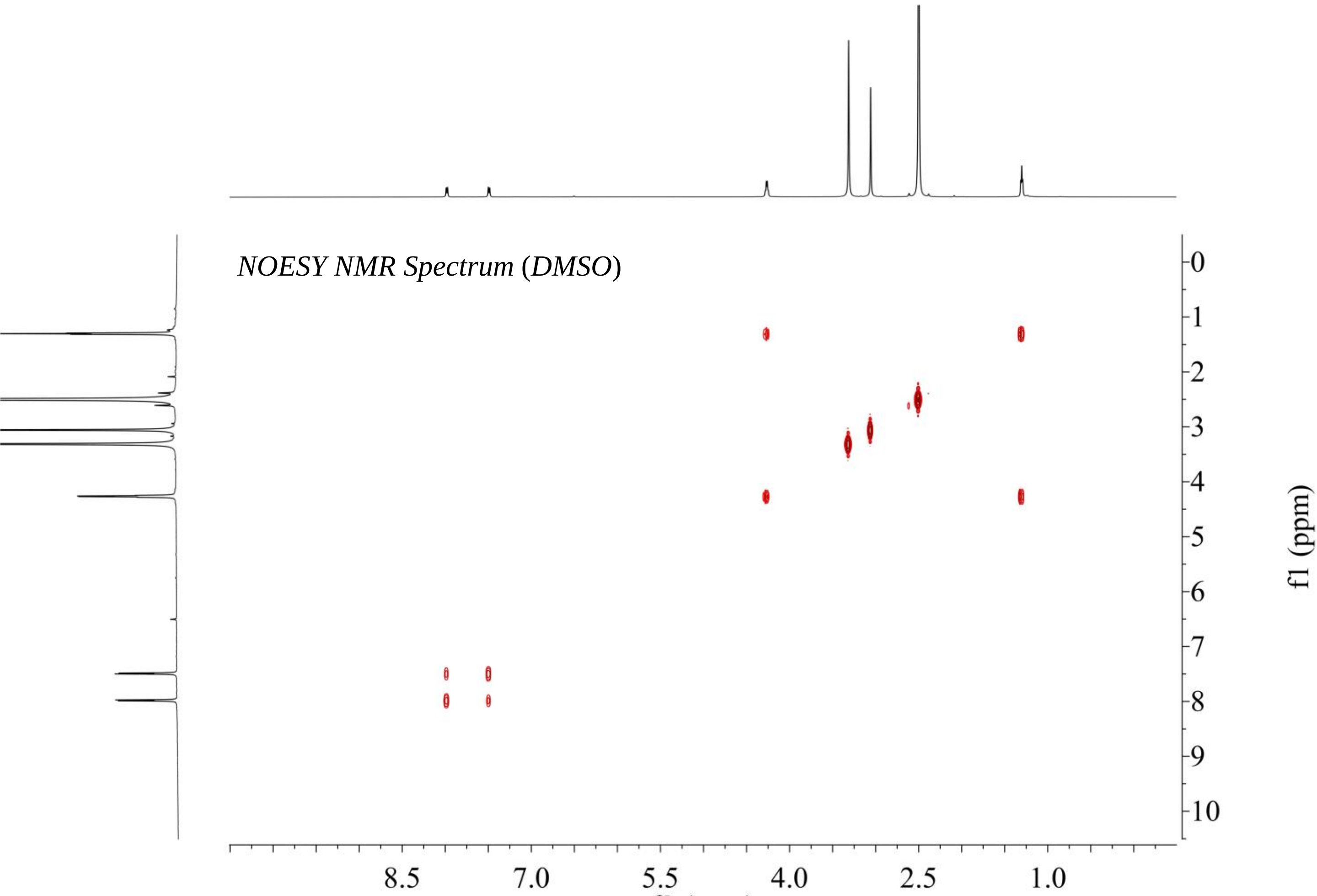

Ethyl 7-(dimethylamino)-2-hydroxy-4-nitropyrazolo[1,5-a]pyridine-3-carboxylate (**8c**)

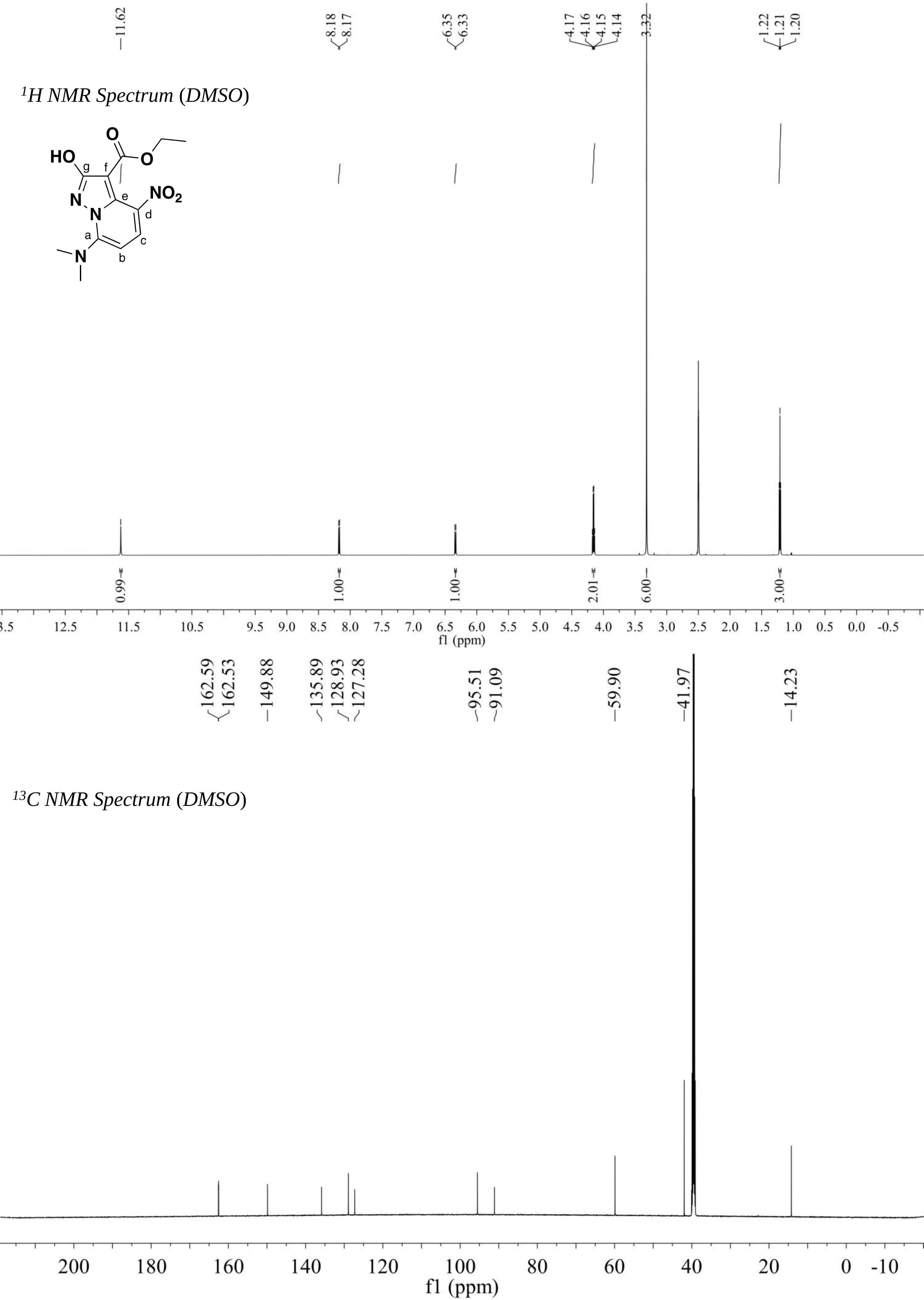

Ethyl 7-(dimethylamino)-2-hydroxy-4-nitropyrazolo[1,5-a]pyridine-3-carboxylate (**8c**)

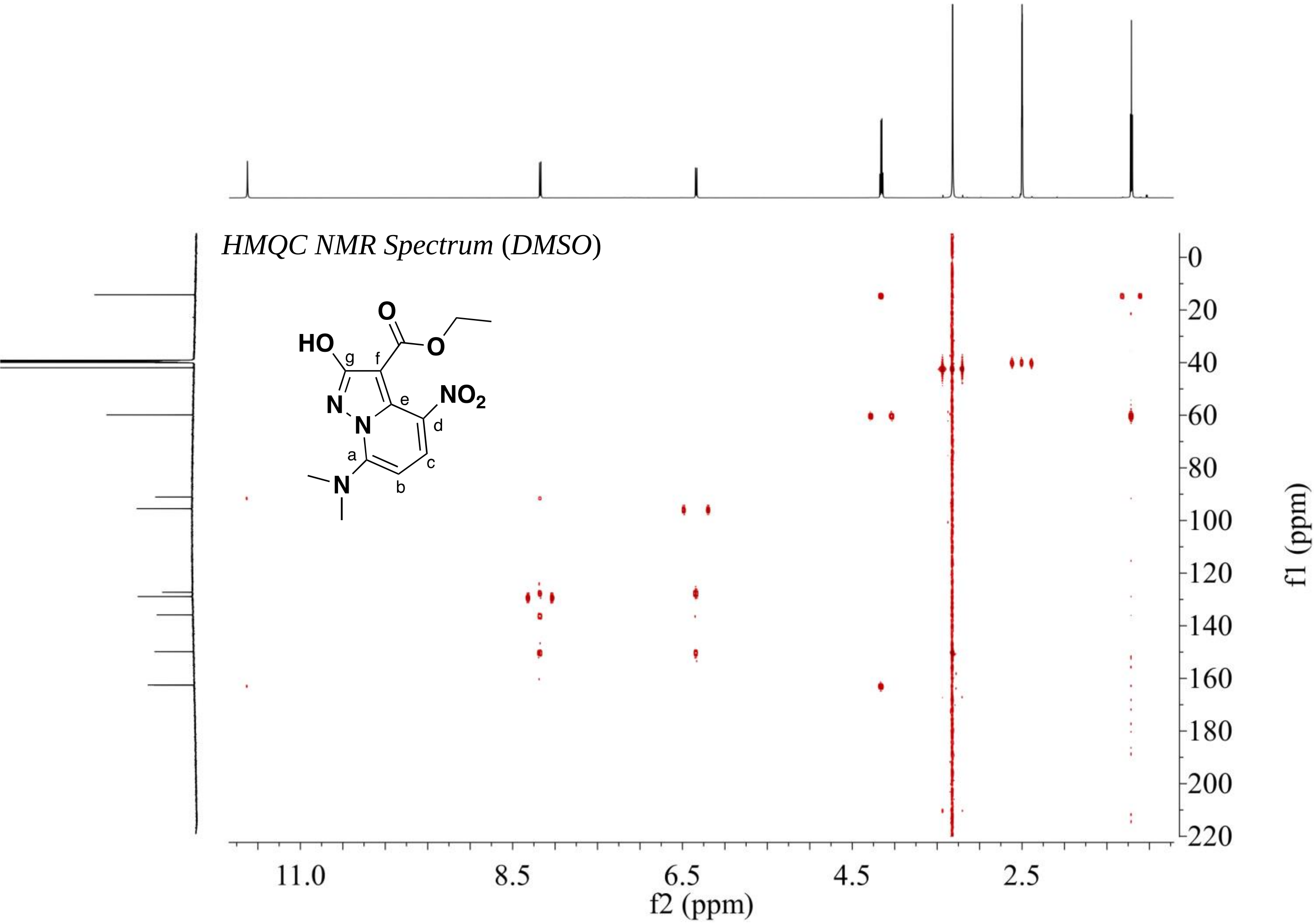

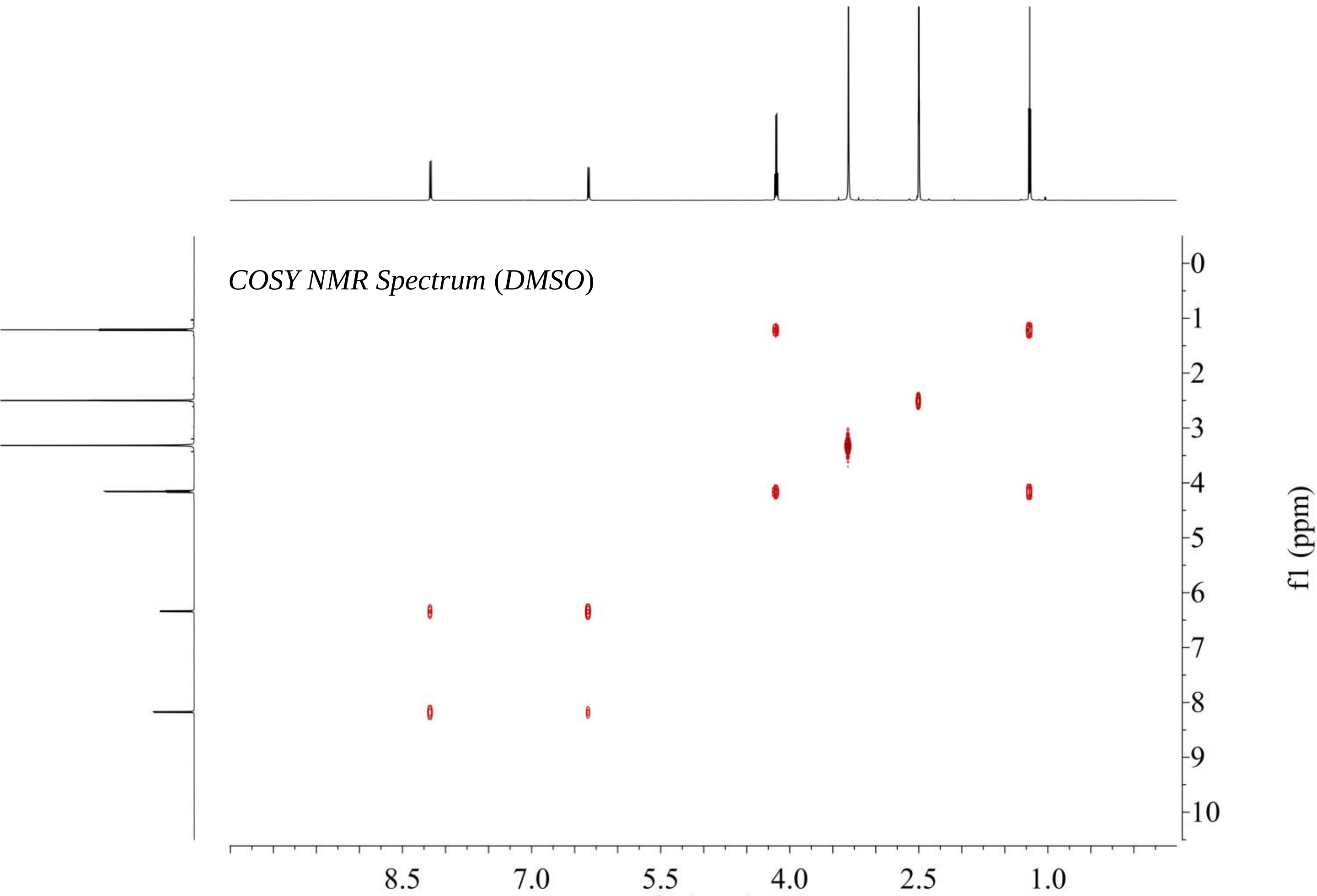

Ethyl 7-(dimethylamino)-2-hydroxy-4-nitropyrazolo[1,5-a]pyridine-3-carboxylate (**8c**)

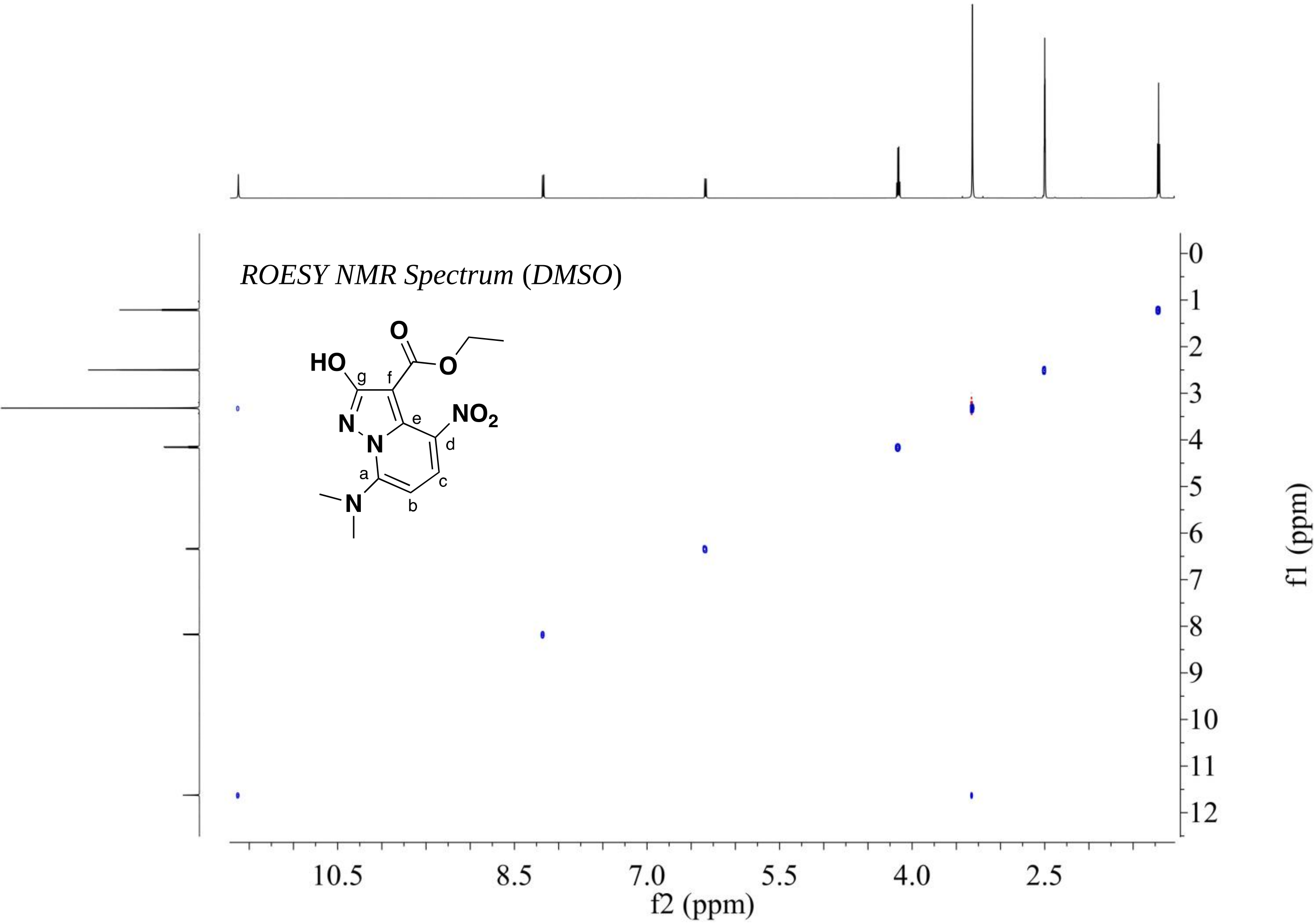

Ethyl 7-chloro-2-methoxypyrazolo[1,5-a]pyridine-3-carboxylate (**23**)

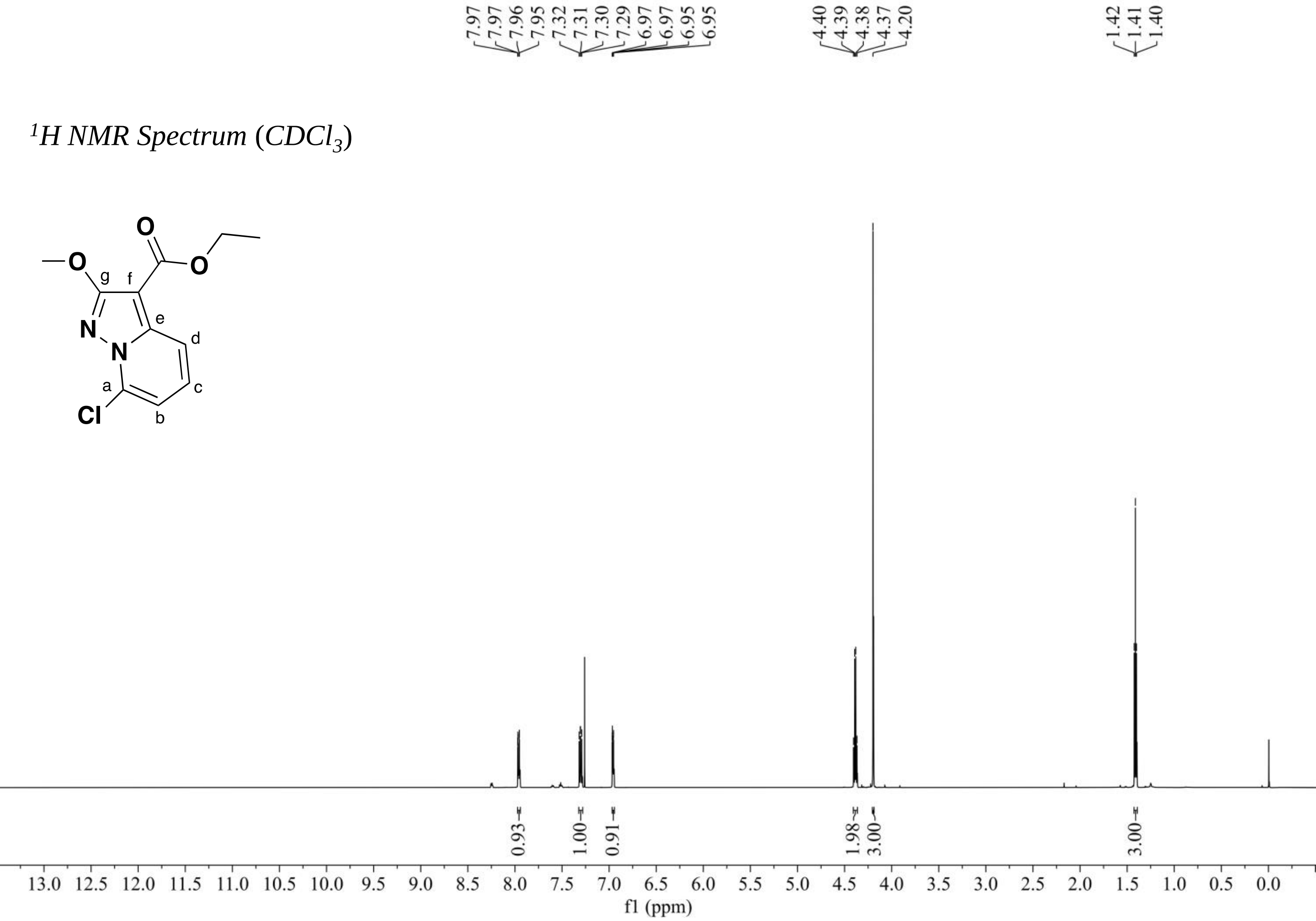

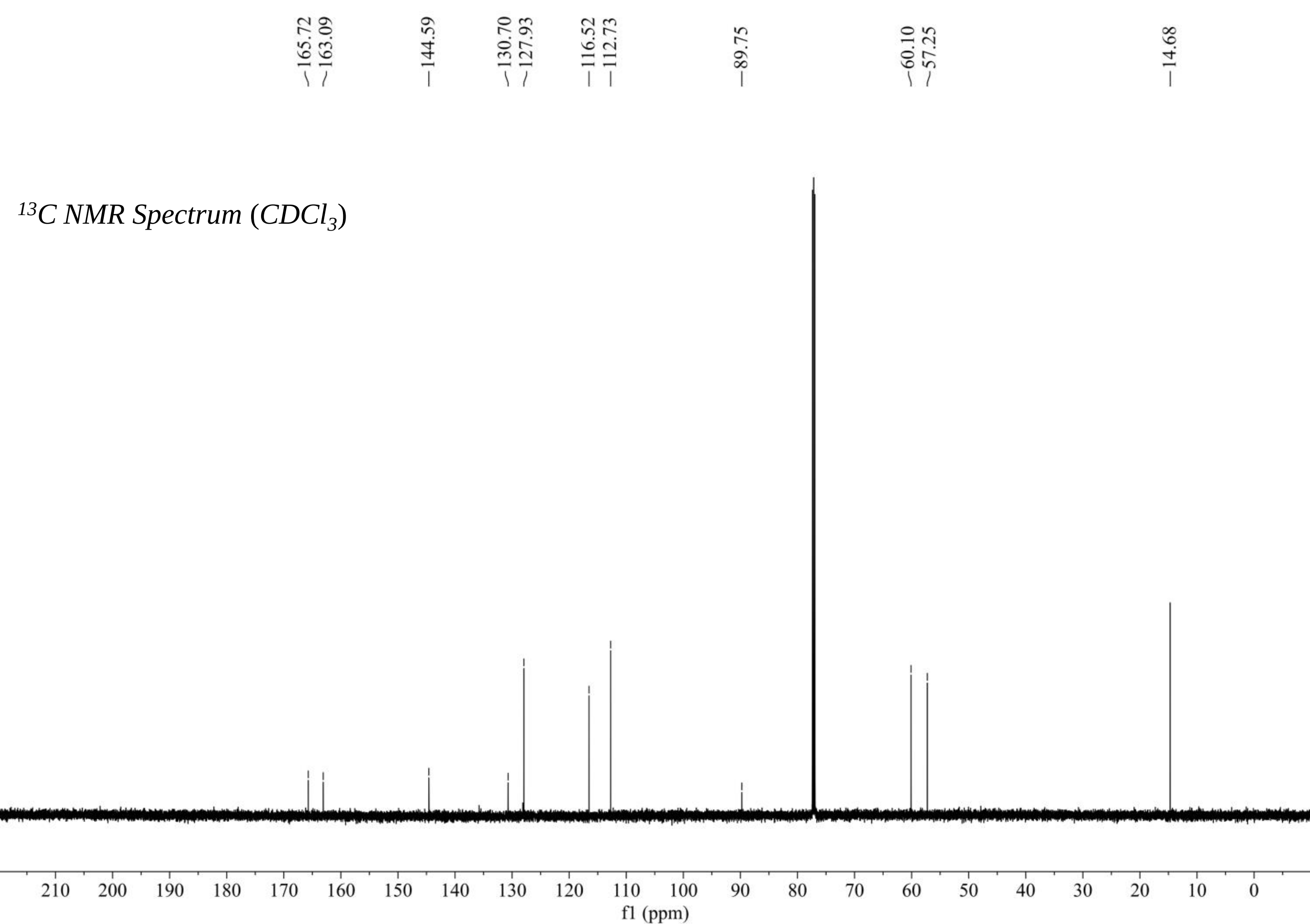

7-Chloro-2-methoxypyrazolo[1,5-a]pyridine (**24**)

^{1}H NMR Spectrum ($CDCl_3$)

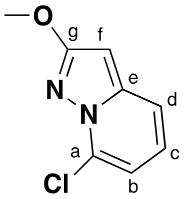

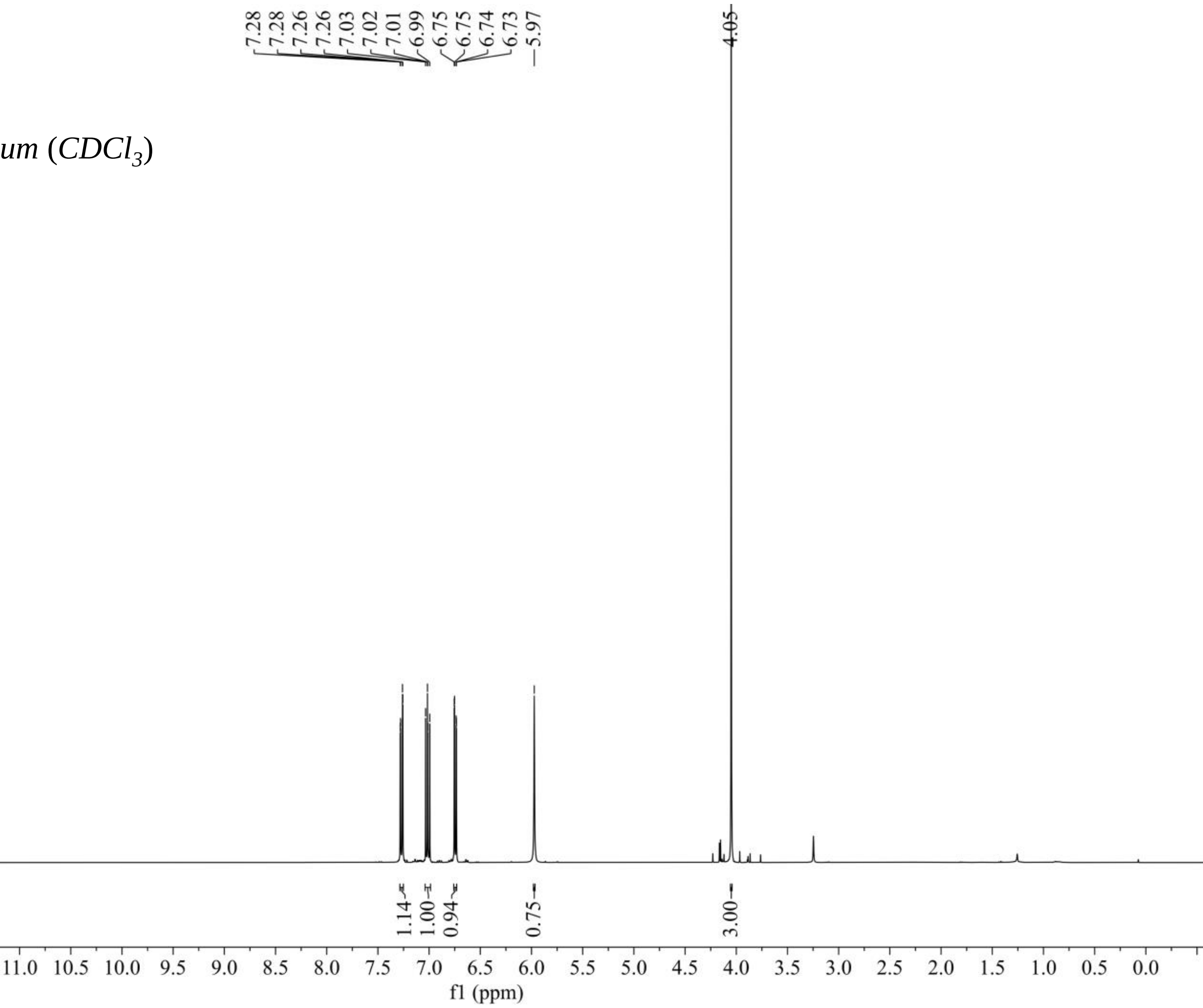

^{13}C NMR Spectrum ($CDCl_3$)

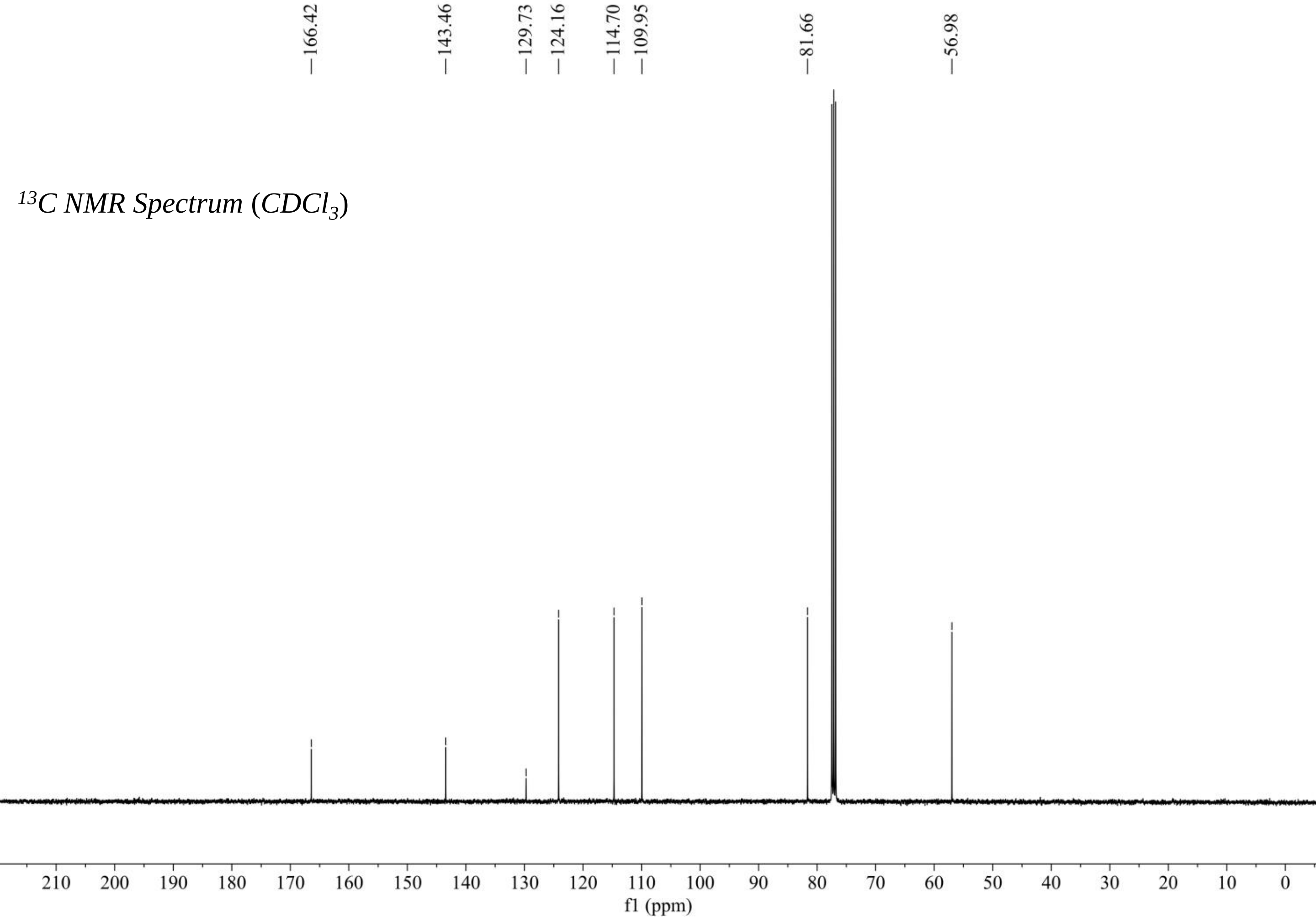

7-Chloro-2-methoxypyrazolo[1,5-a]pyridine (**24**)

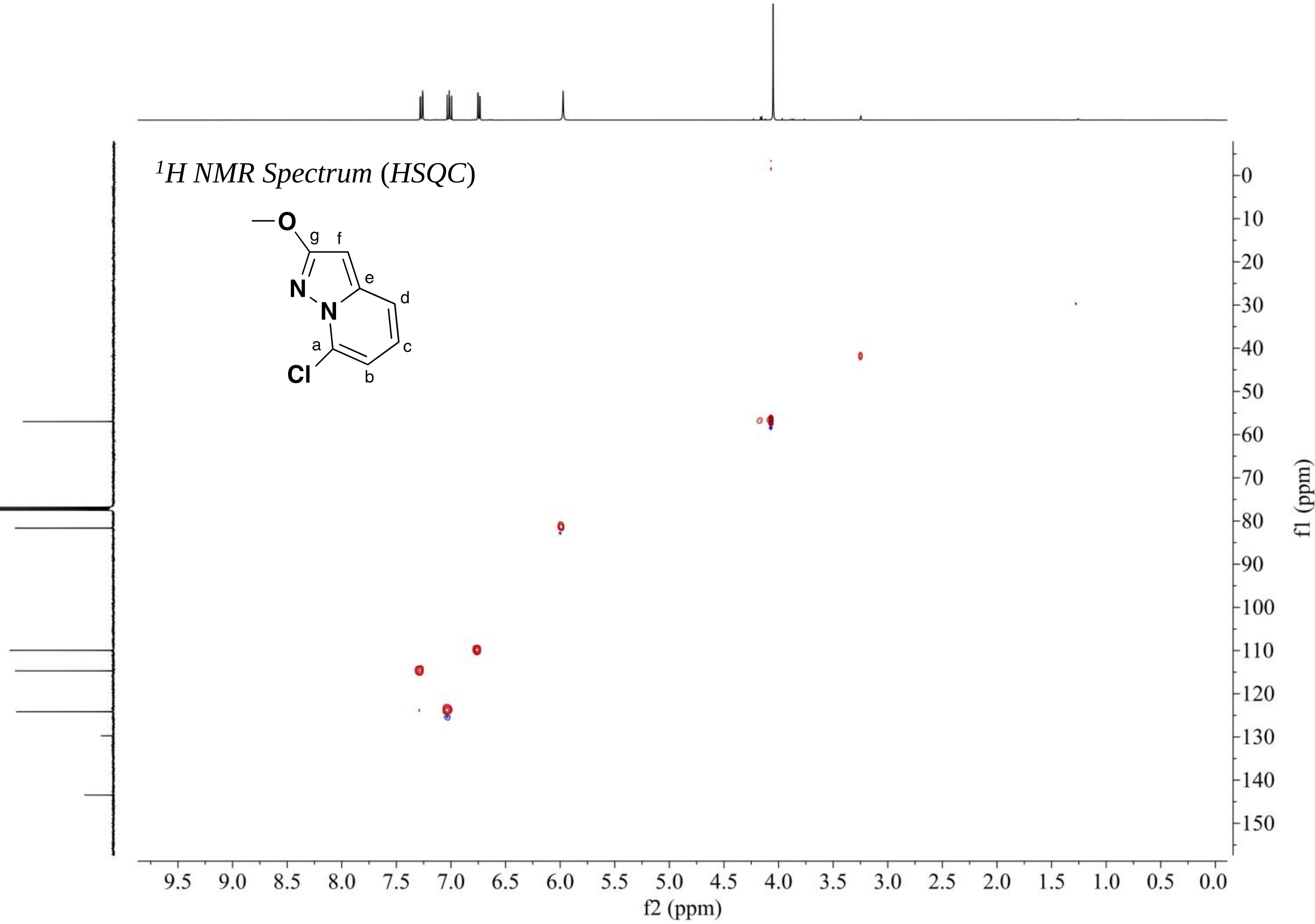

7-Chloro-2-methoxypyrazolo[1,5-a]pyridine (**25**)

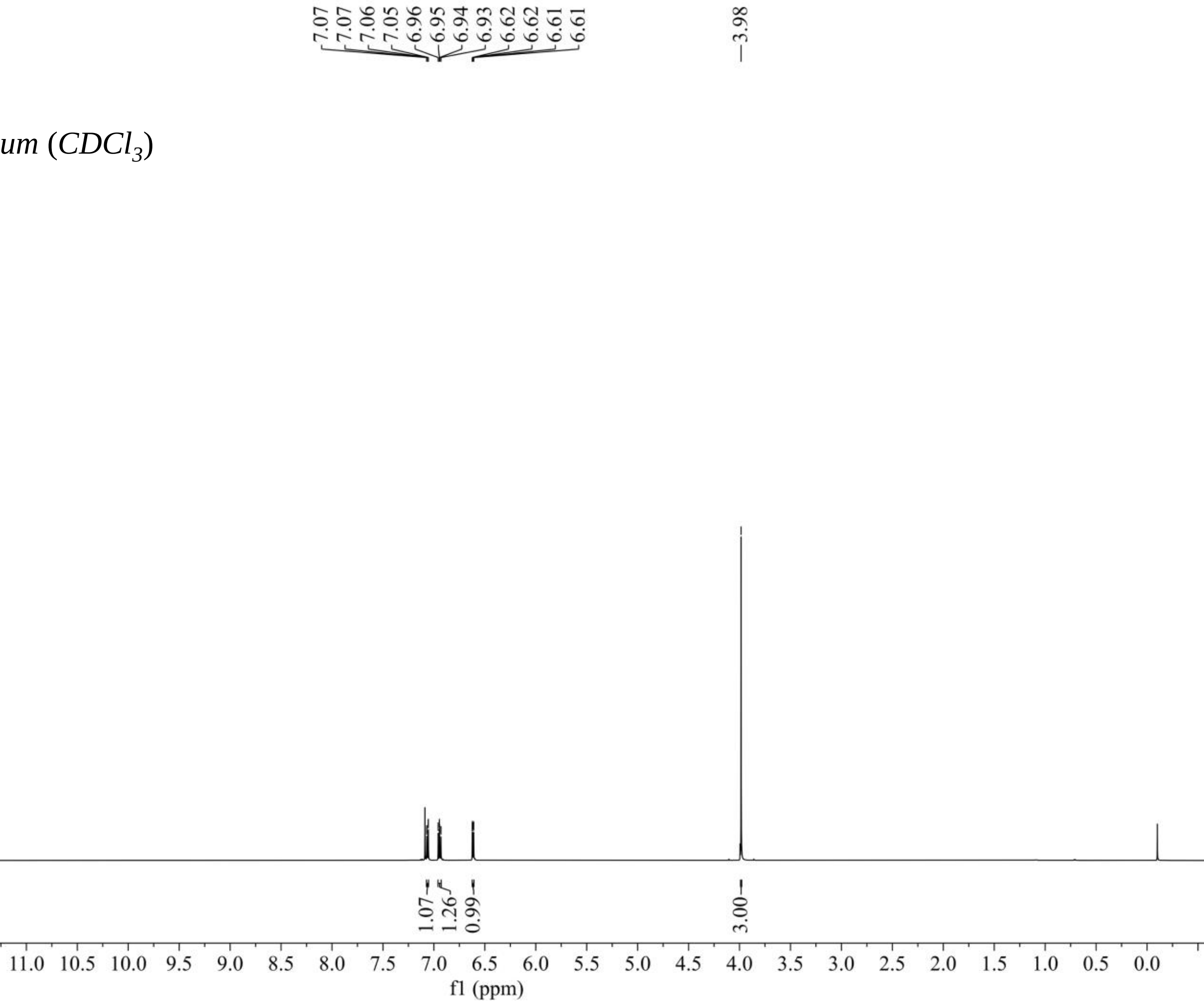

^{1}H NMR Spectrum ($CDCl_3$)

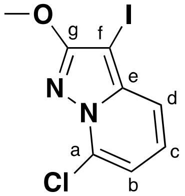

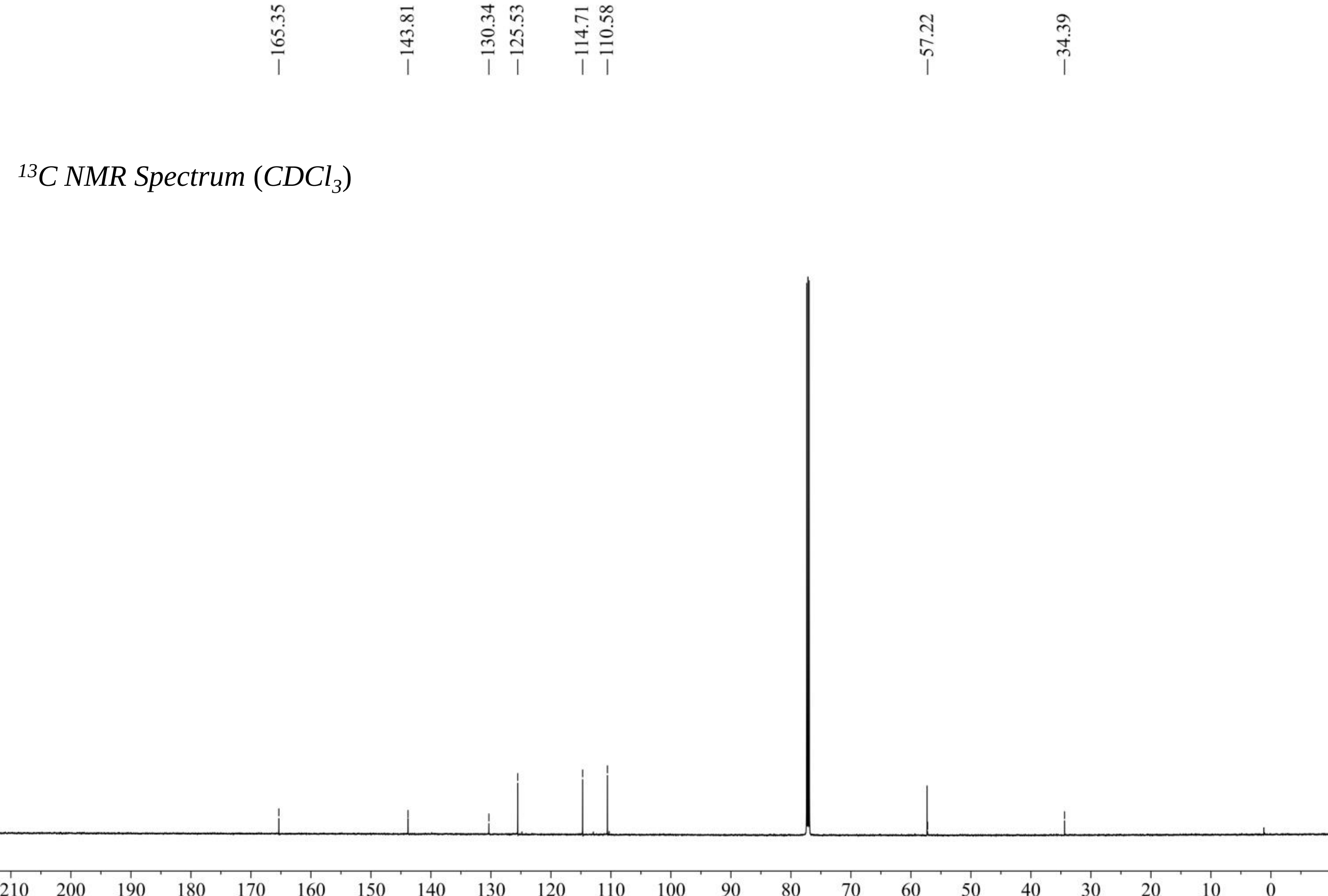

^{13}C NMR Spectrum ($CDCl_3$)

7-Chloro-2-methoxypyrazolo[1,5-a]pyridine (**25**)

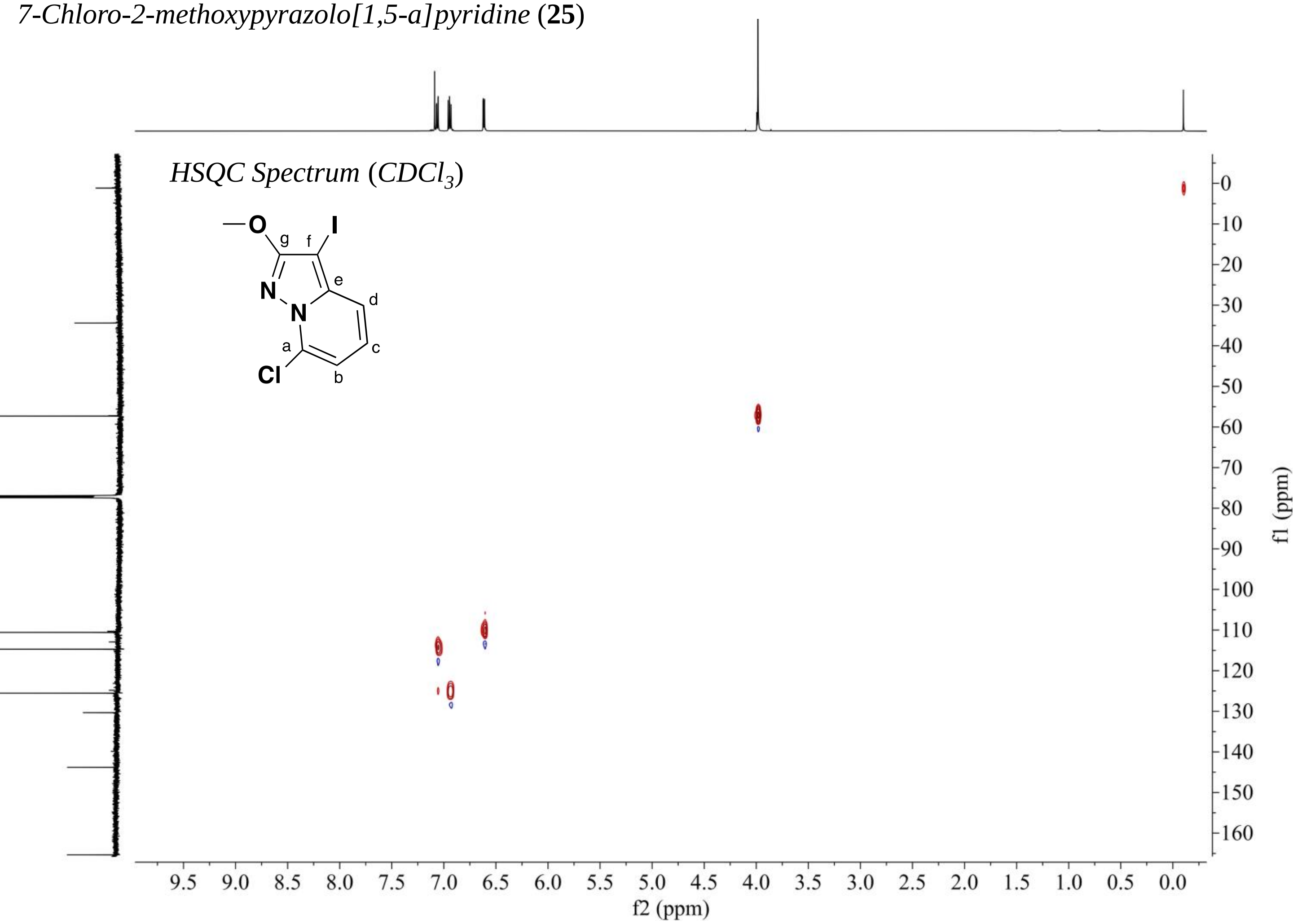

6-(7-Chloro-2-methoxypyrazolo[1,5-a]pyridin-3-yl)quinoline (**9a**)

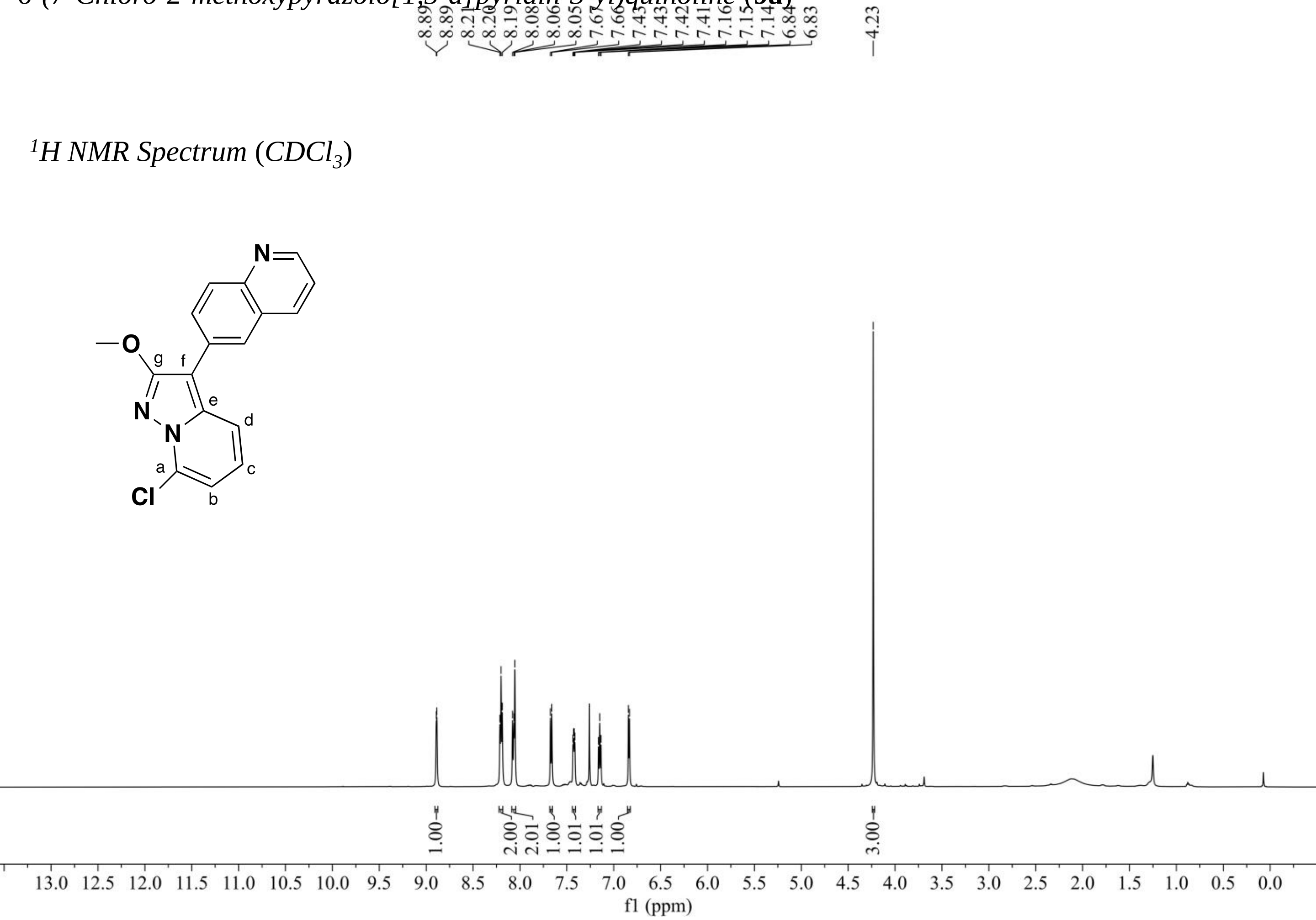

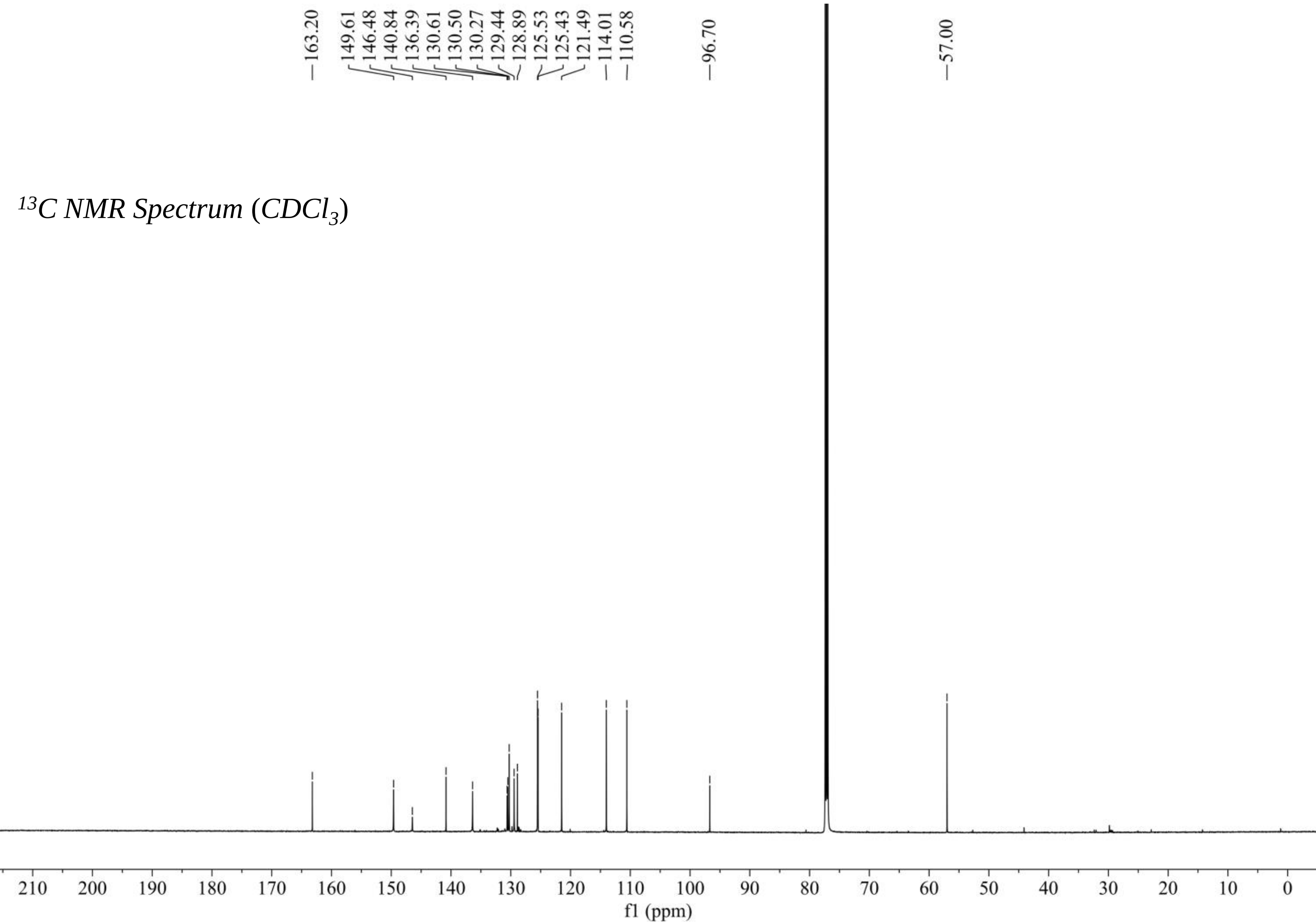

6-(7-Chloro-2-methoxypyrazolo[1,5-a]pyridin-3-yl)quinoline (**9a**)

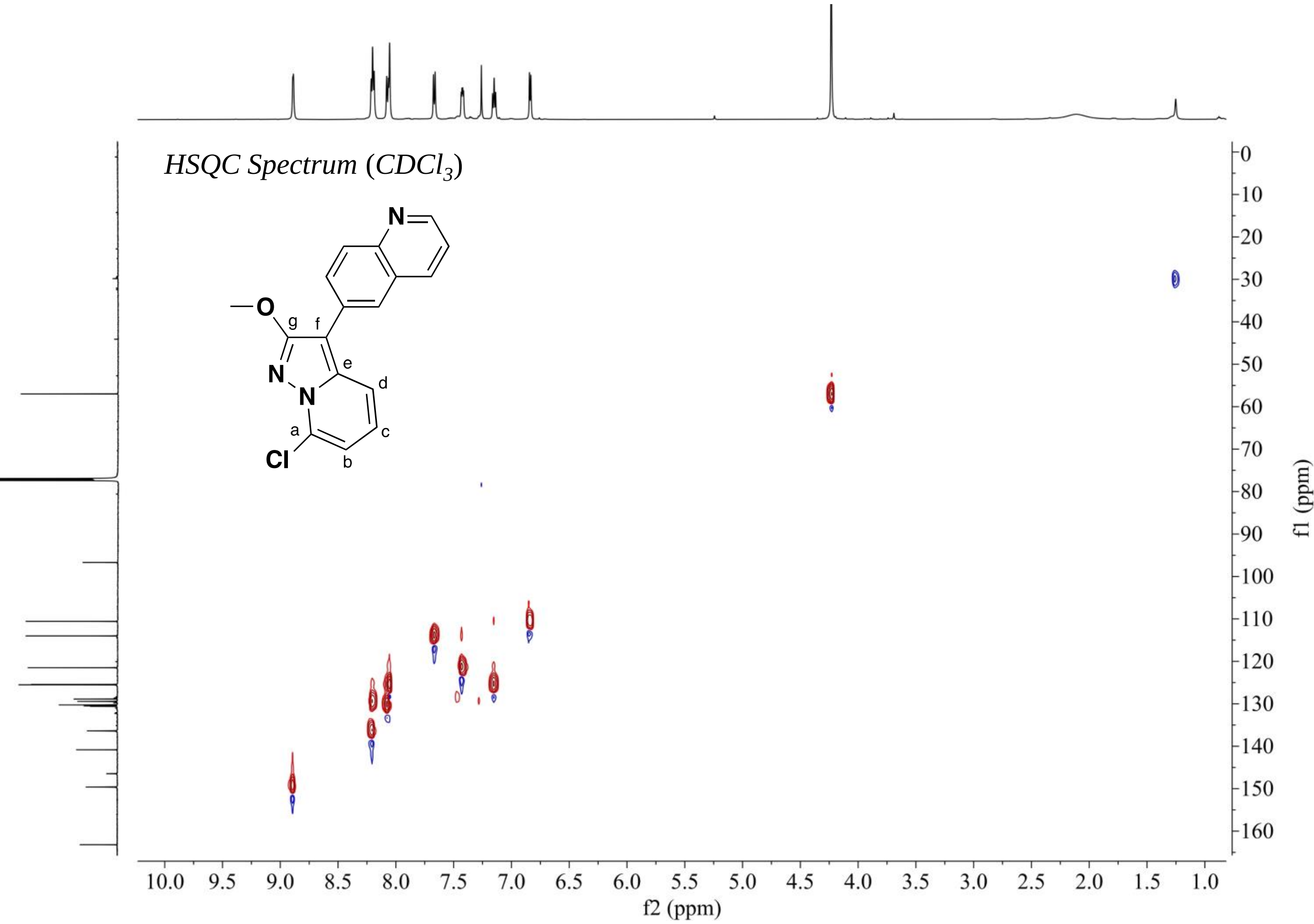

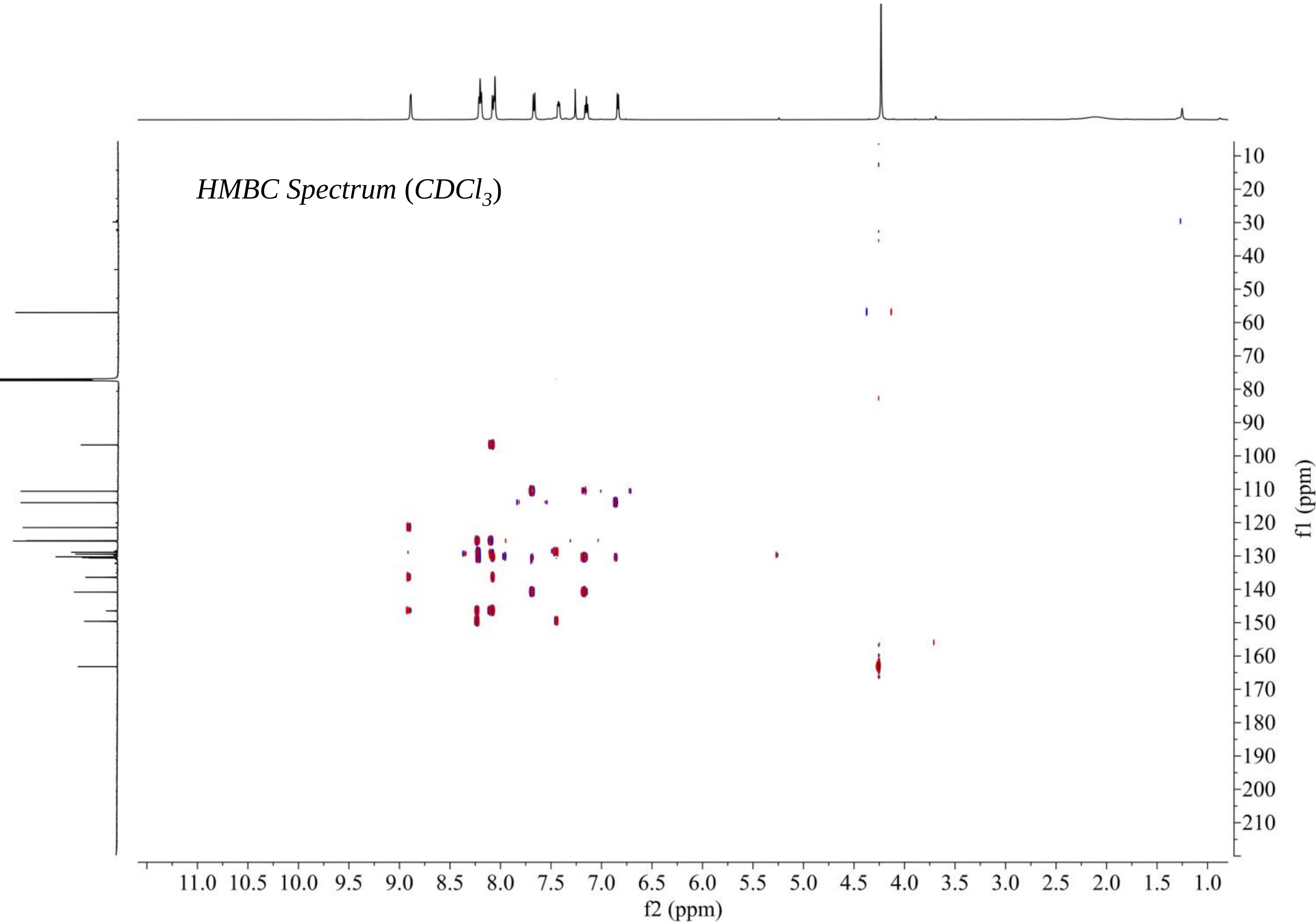

2-Methoxy-N,N-dimethyl-3-(quinolin-6-yl)pyrazolo[1,5-a]pyridin-7-amine (**9b**)

^{1}H NMR Spectrum (CDCl$_3$)

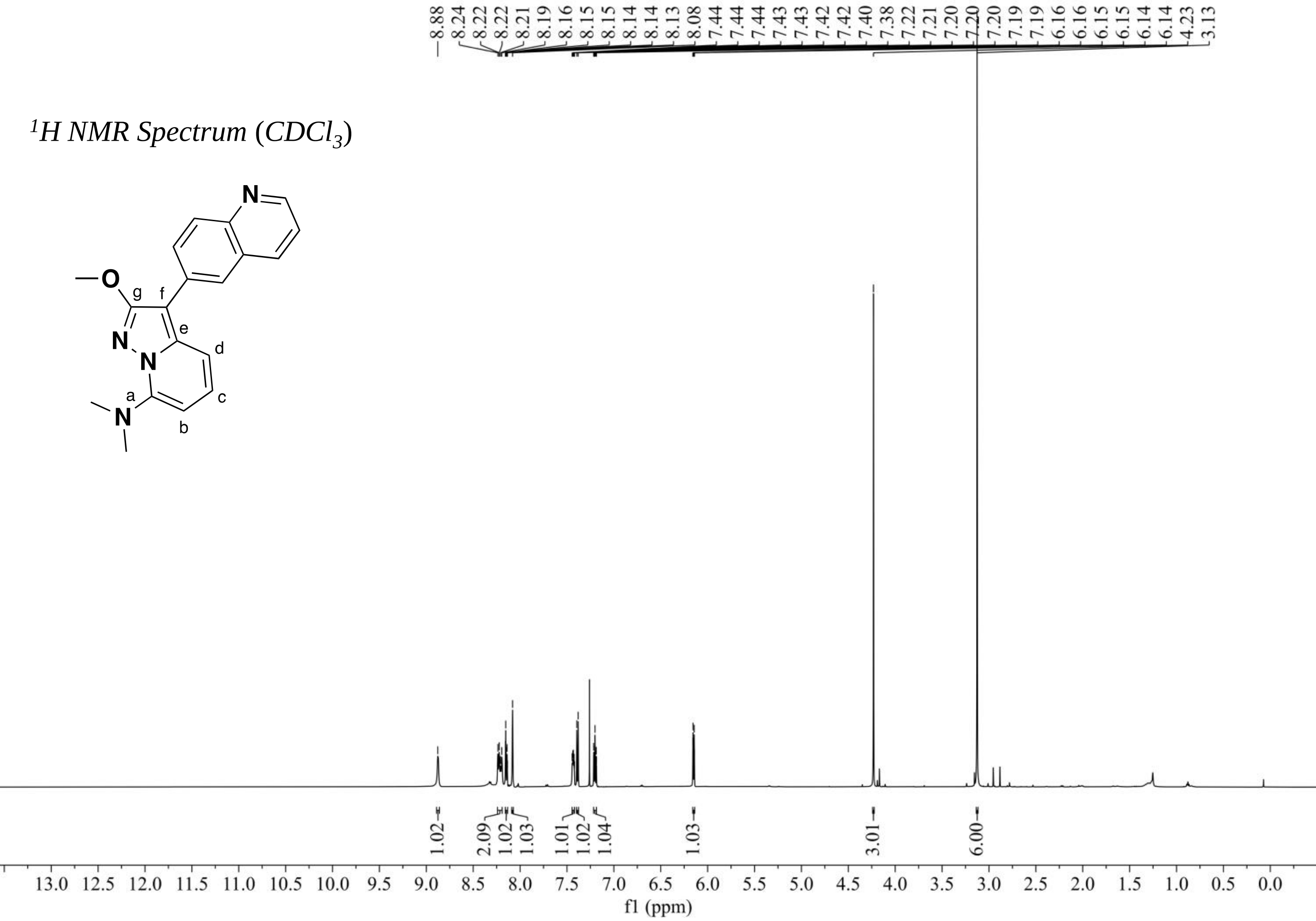

^{13}C NMR Spectrum (CDCl$_3$)

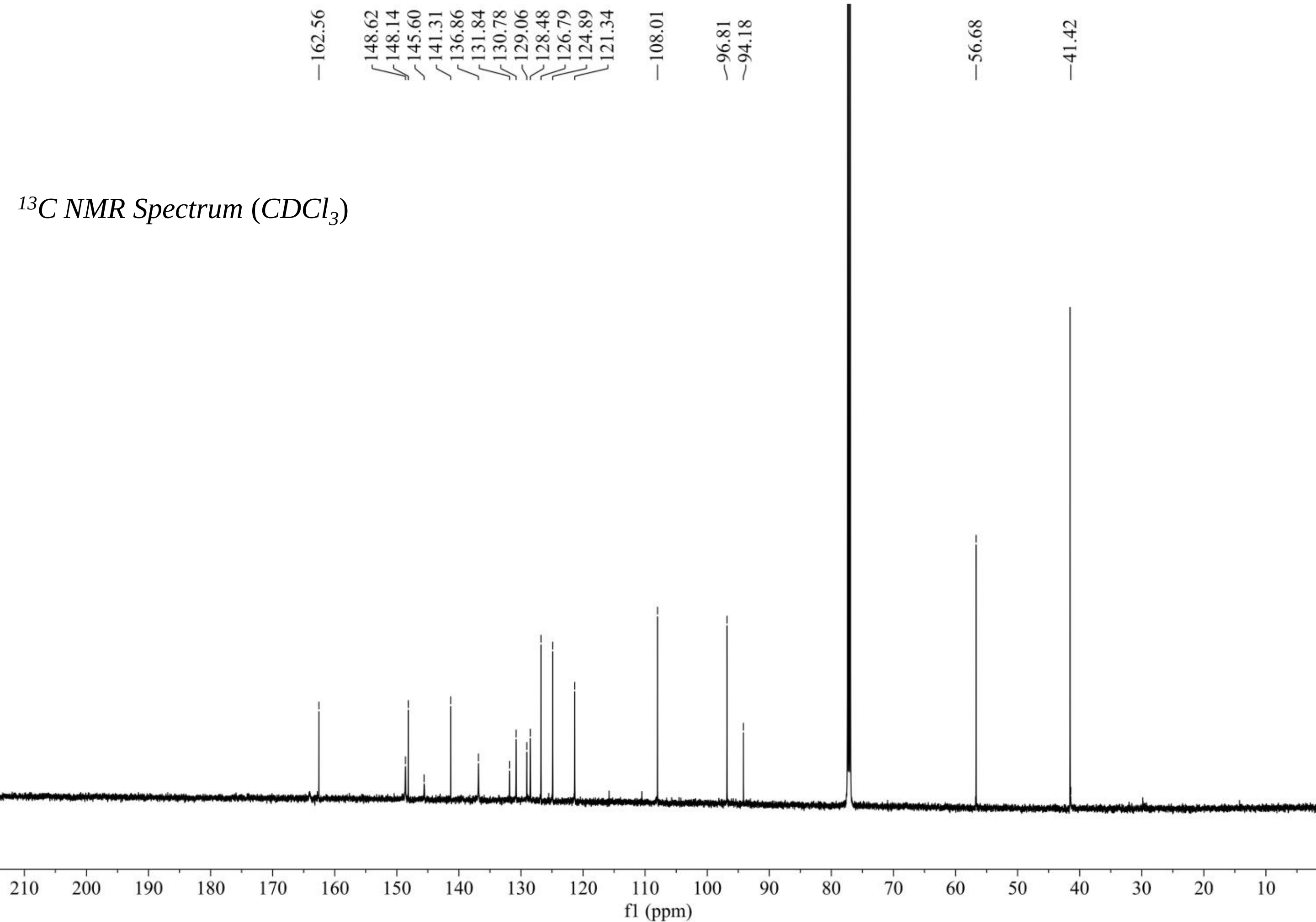

2-Methoxy-N,N-dimethyl-3-(quinolin-6-yl)pyrazolo[1,5-a]pyridin-7-amine (**9b**)

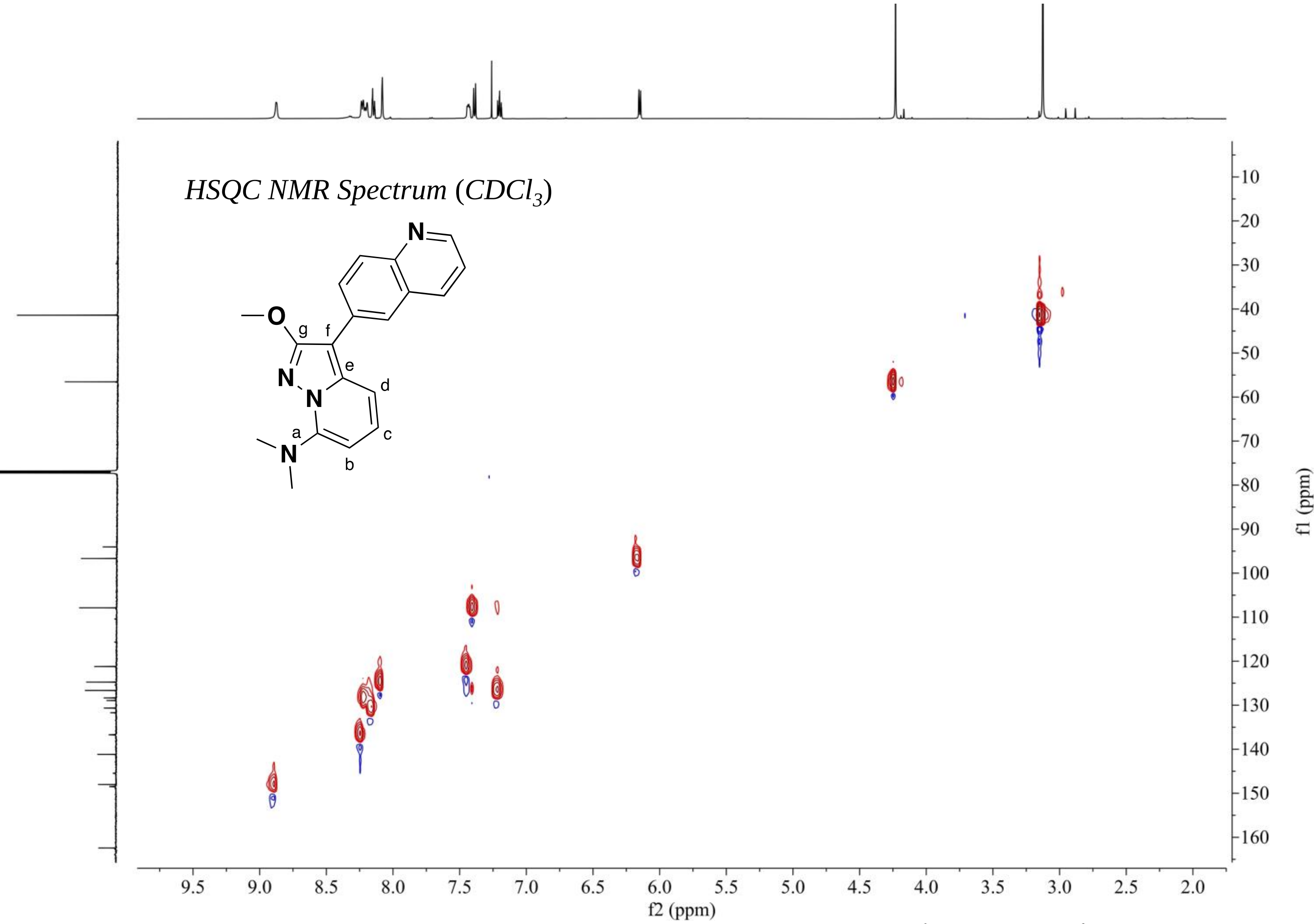

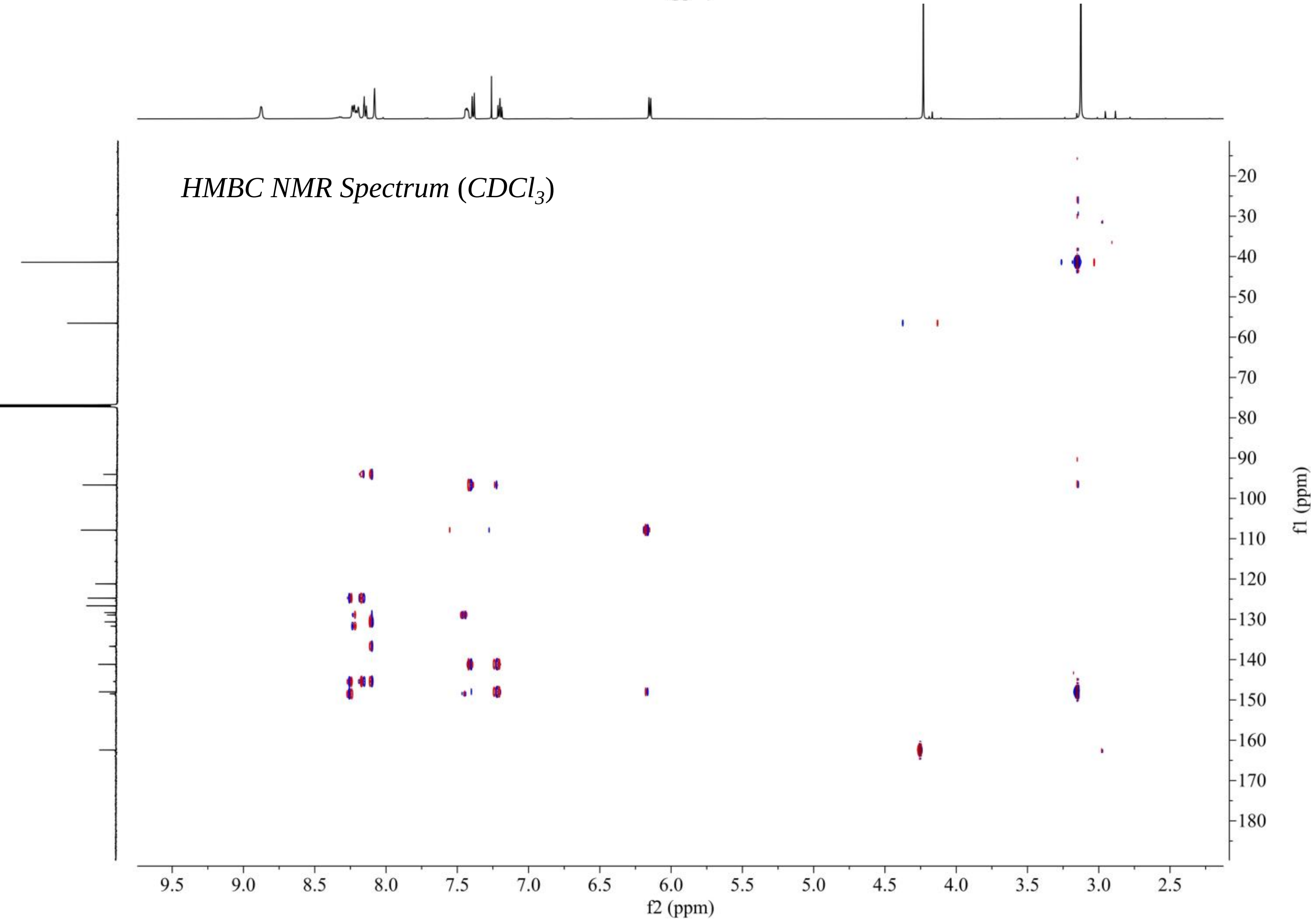

2-((4-Methoxybenzyl)oxy)pyrazolo[1,5-a]pyridine-3-carbaldehyde (**26**)

^{1}H NMR Spectrum (*CDCl$_3$*)

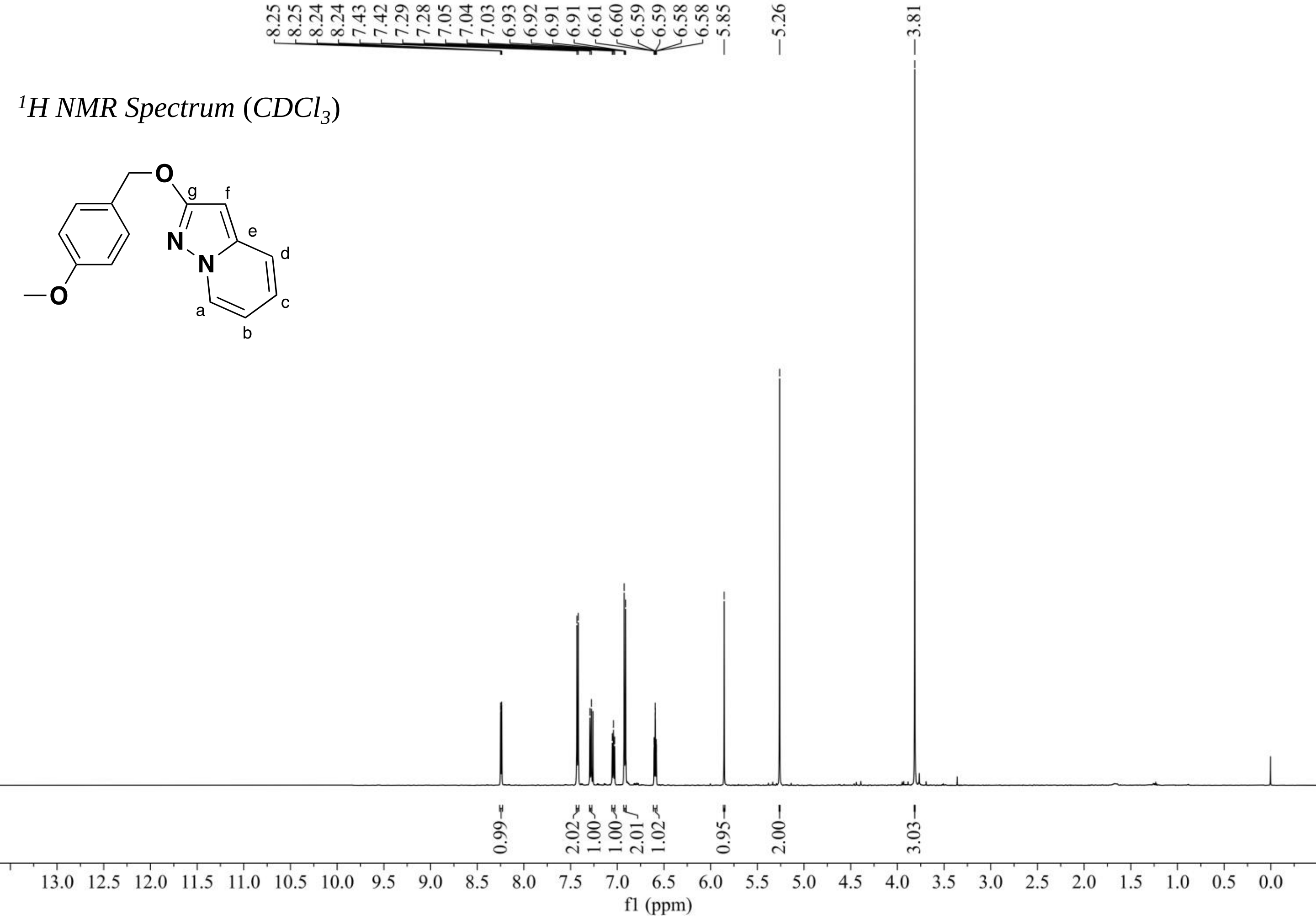

^{13}C NMR Spectrum (*CDCl$_3$*)

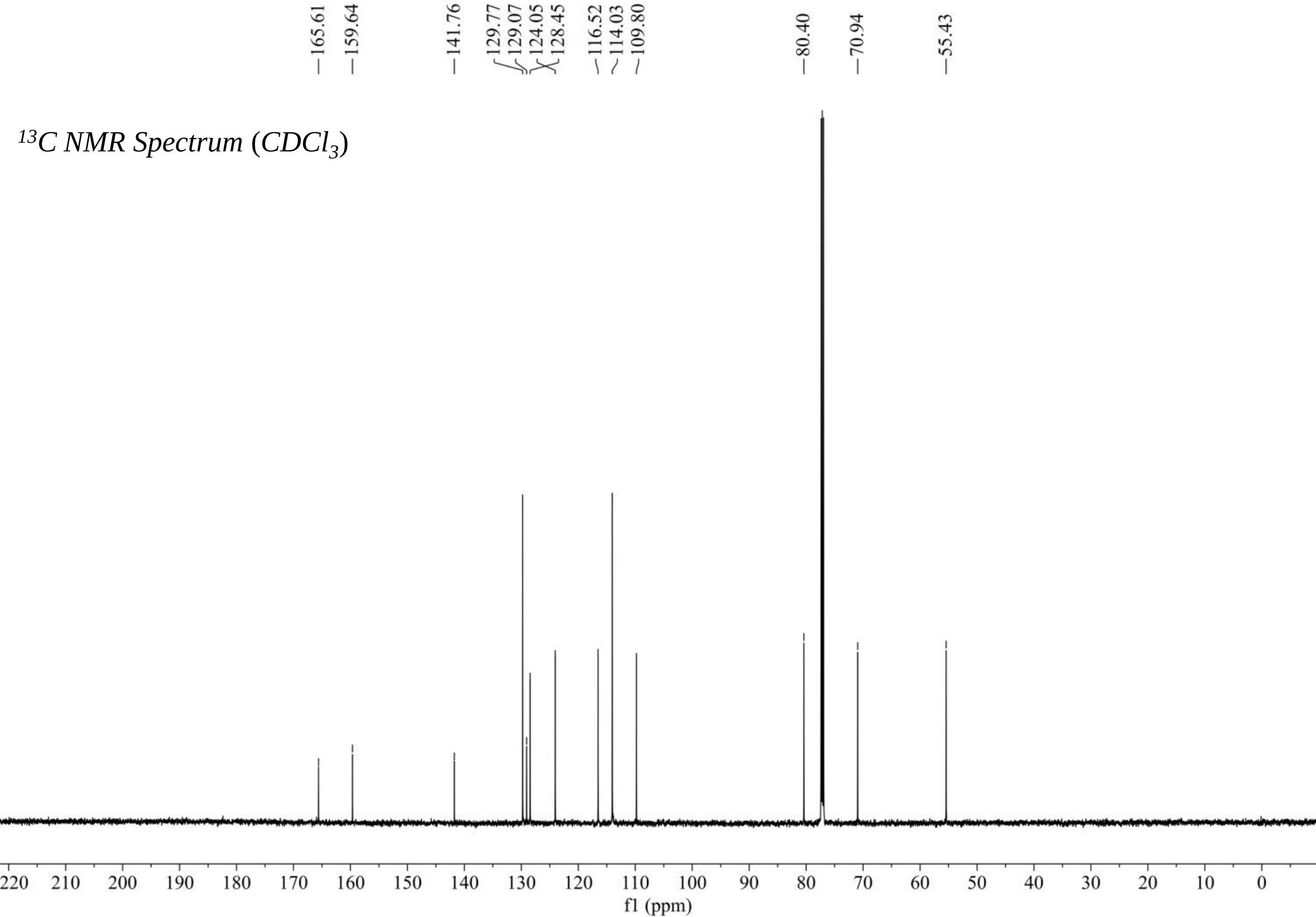

2-((4-Methoxybenzyl)oxy)pyrazolo[1,5-a]pyridine-3-carbaldehyde (**27**)

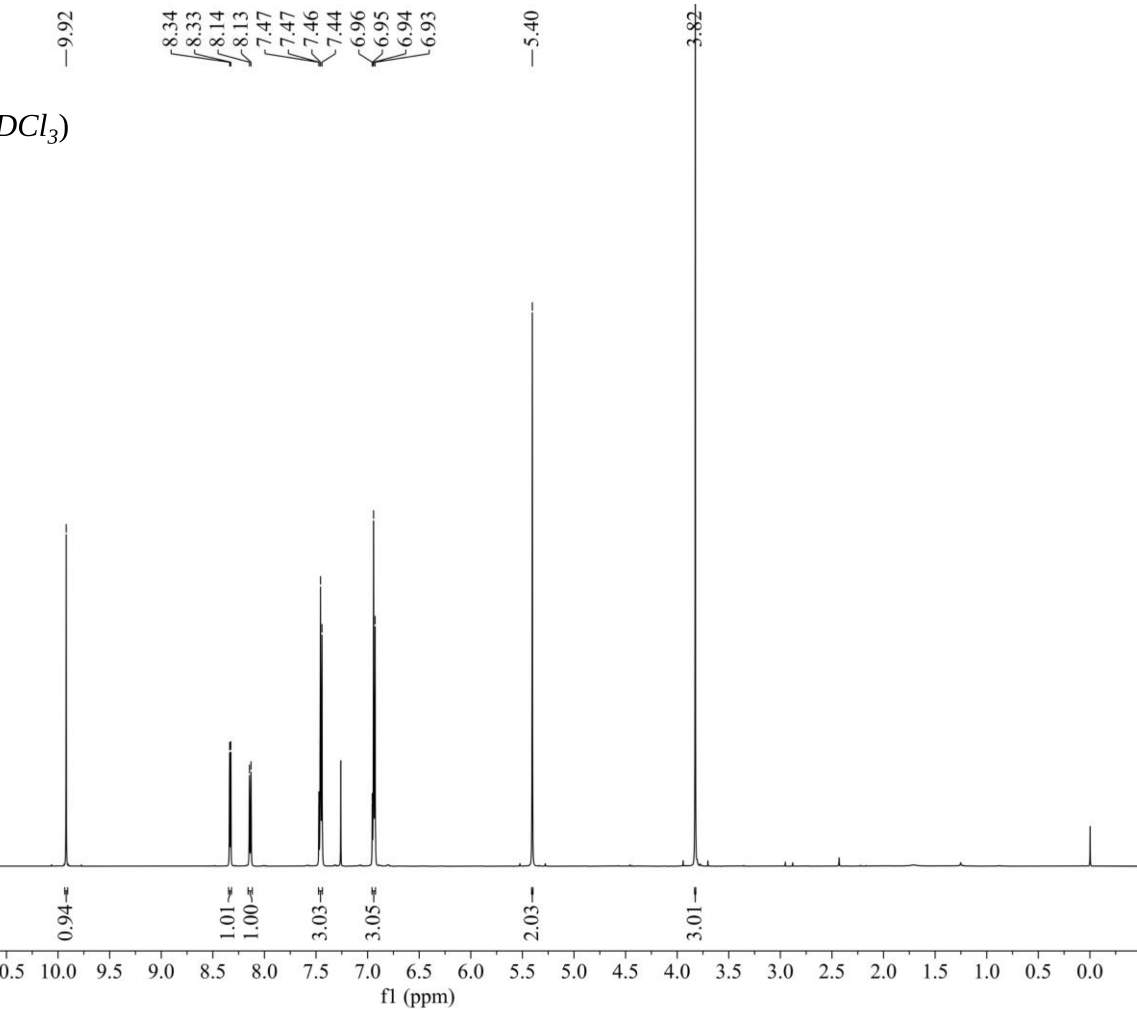

^{1}H NMR Spectrum ($CDCl_3$)

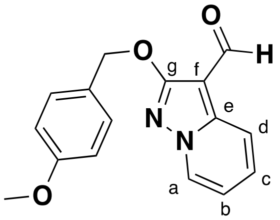

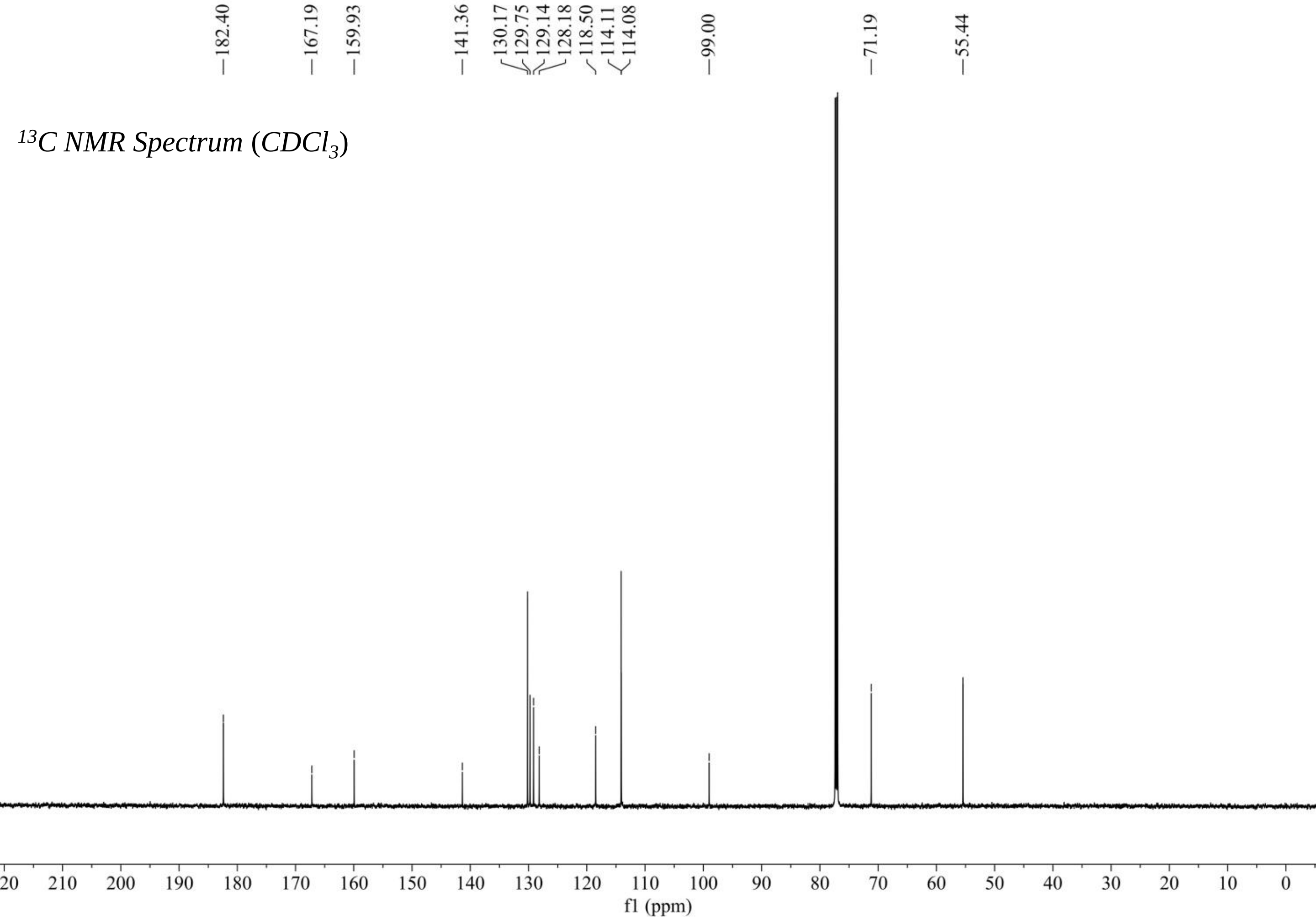

^{13}C NMR Spectrum ($CDCl_3$)

methyl 4-bromo-2,3,5,6-tetrafluorobenzoate (**36**)

^{1}H NMR Spectrum ($CDCl_3$)

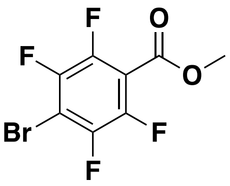

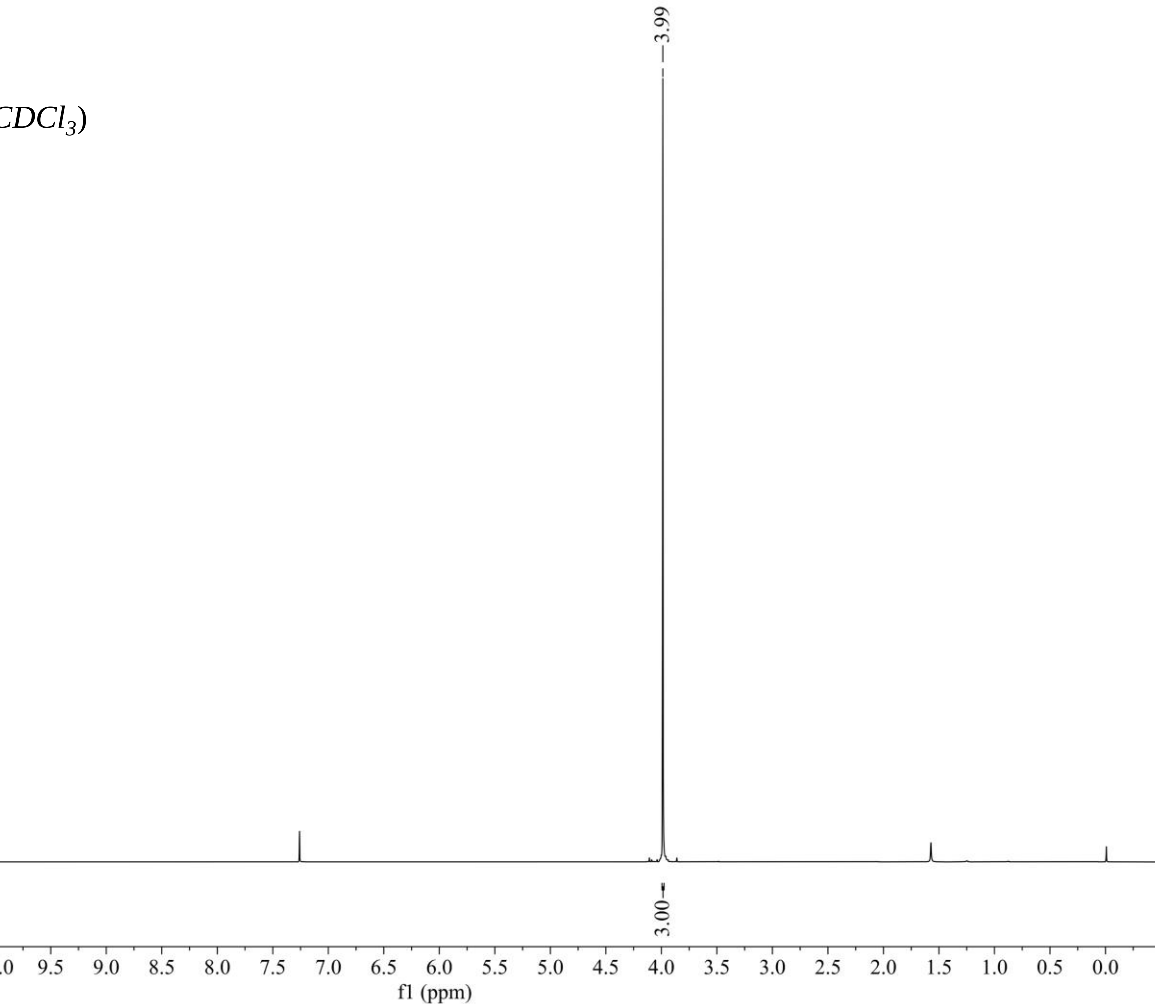

^{13}C NMR Spectrum ($CDCl_3$)

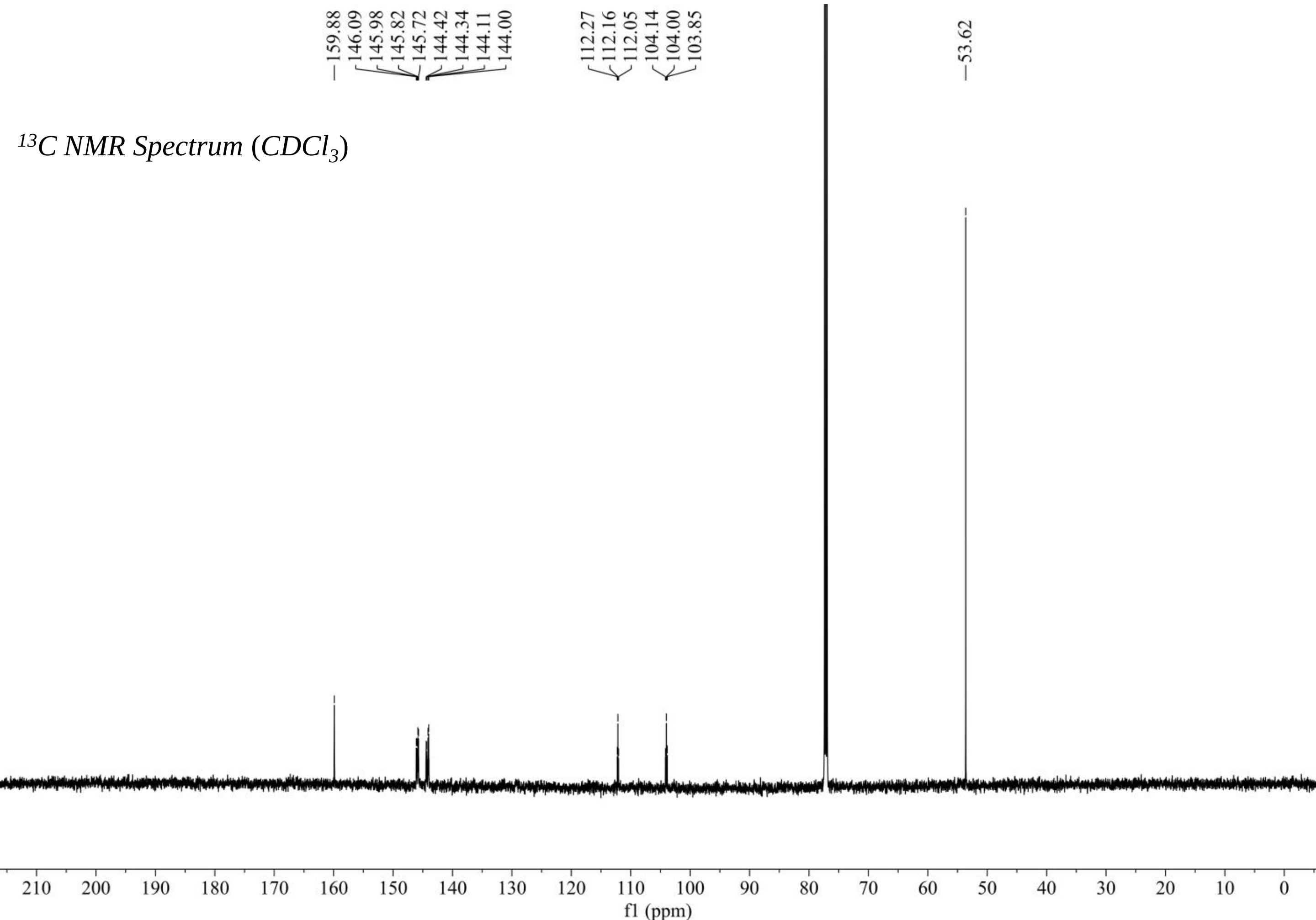

methyl 4-bromo-2,3,5,6-tetrafluorobenzoate (**36**)

^{19}F NMR Spectrum (CDCl$_3$)

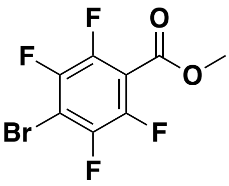

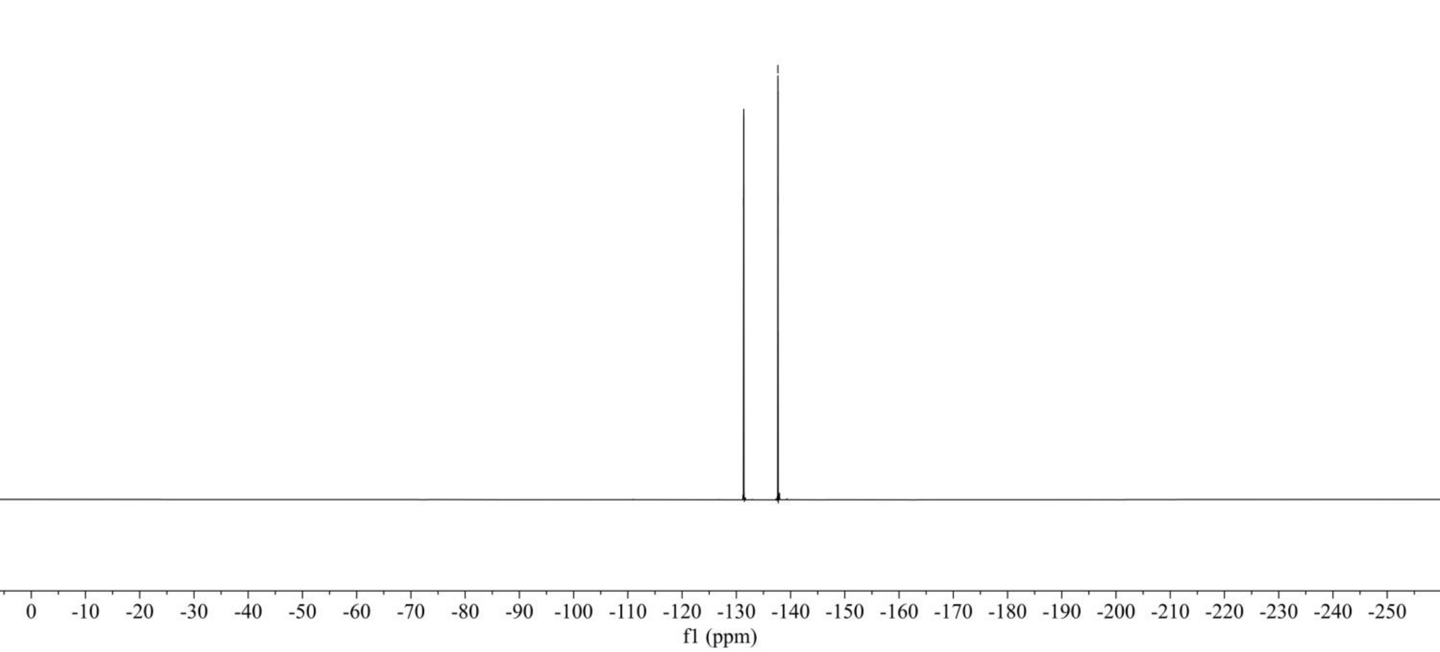

methyl 2,3,5,6-tetrafluoro-[1,1'-biphenyl]-4-carboxylate (**37**)

^{1}H NMR Spectrum ($CDCl_3$)

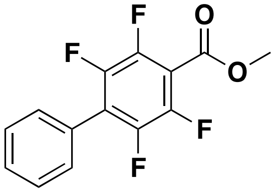

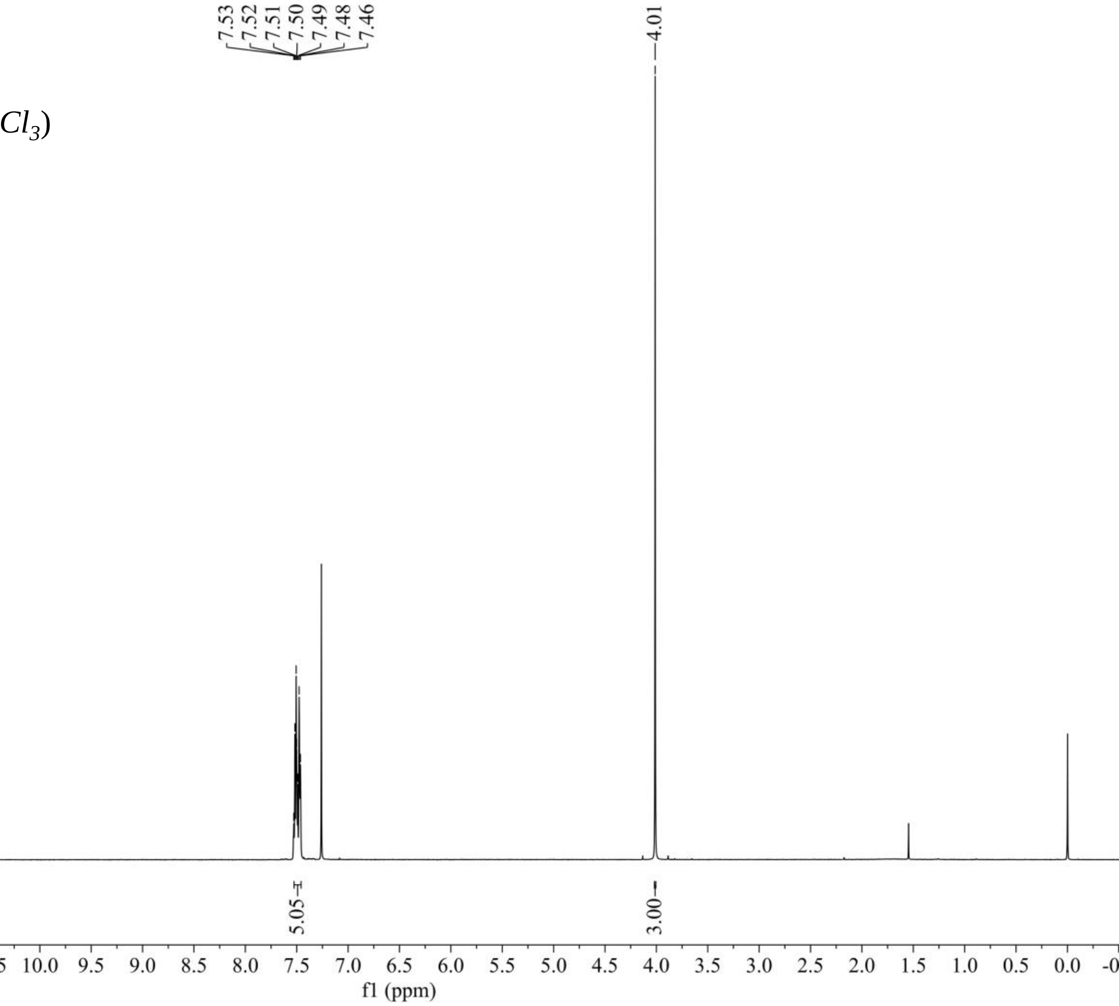

^{13}C NMR Spectrum ($CDCl_3$)

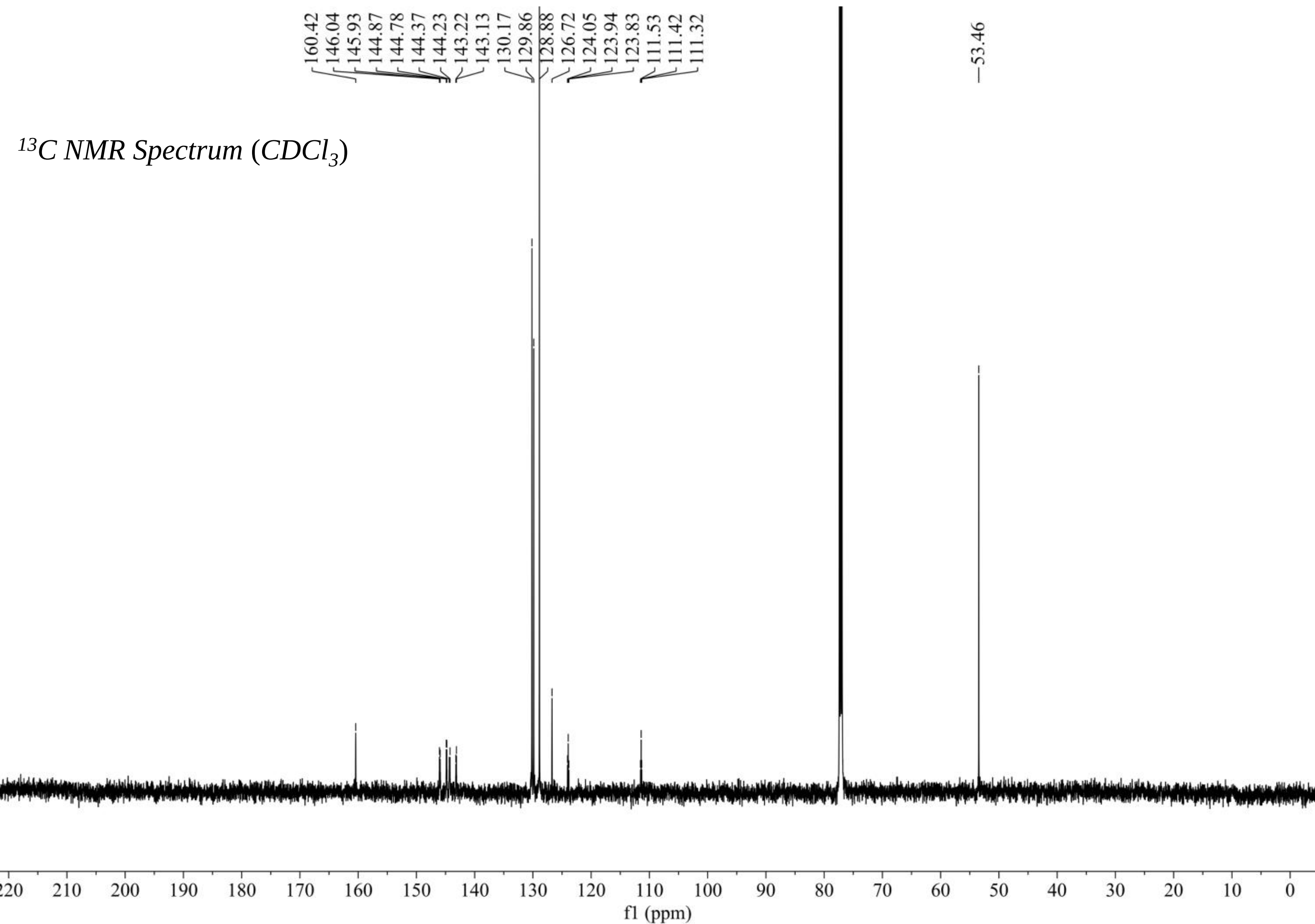

methyl 2,3,5,6-tetrafluoro-[1,1'-biphenyl]-4-carboxylate (**37**)

^{19}*F NMR Spectrum* (*CDCl$_3$*)

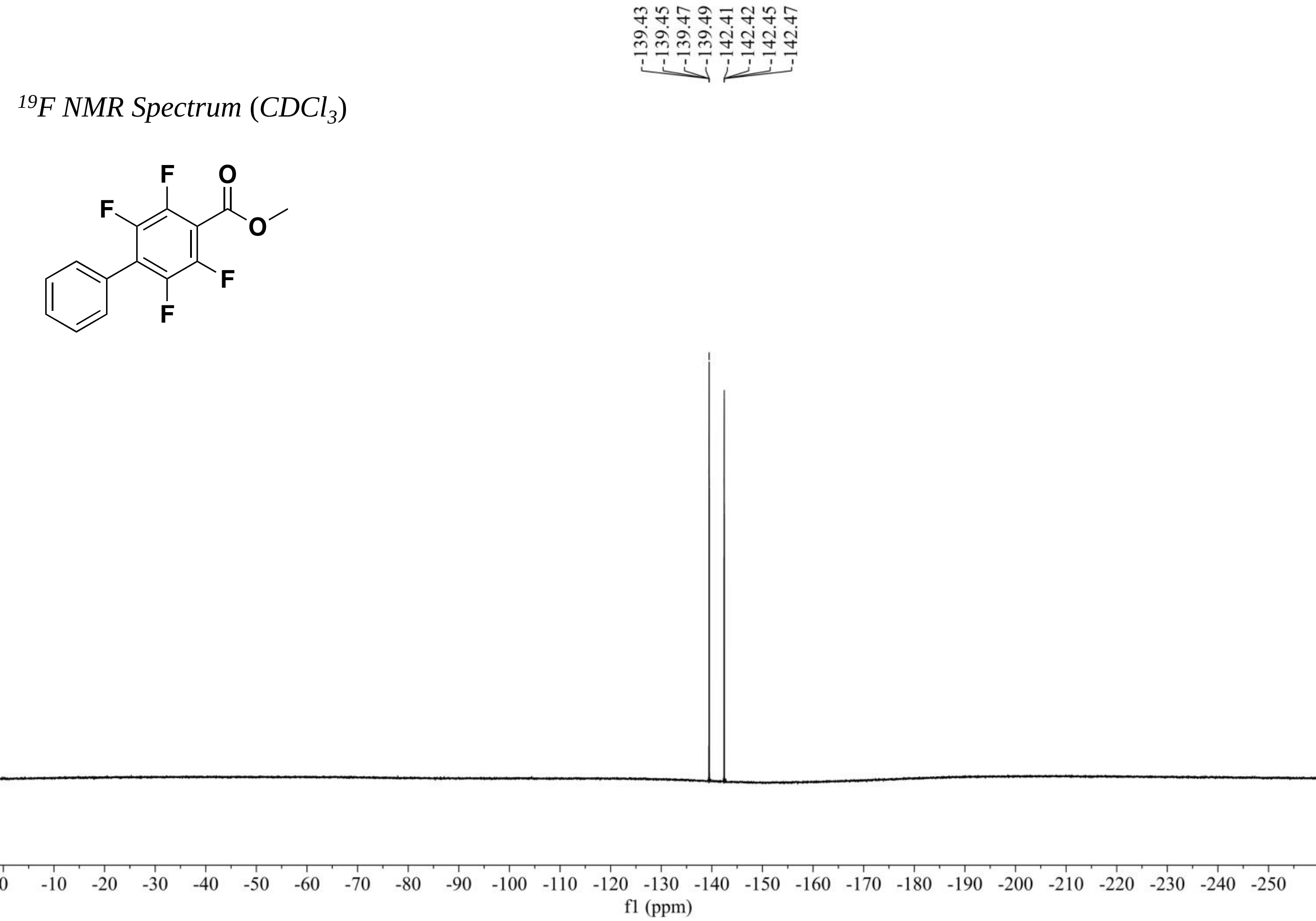

2,3,6-Trifluoro-[1,1'-biphenyl]-4-yl)methanol (**38b**)

¹H NMR Spectrum (*CDCl₃*)

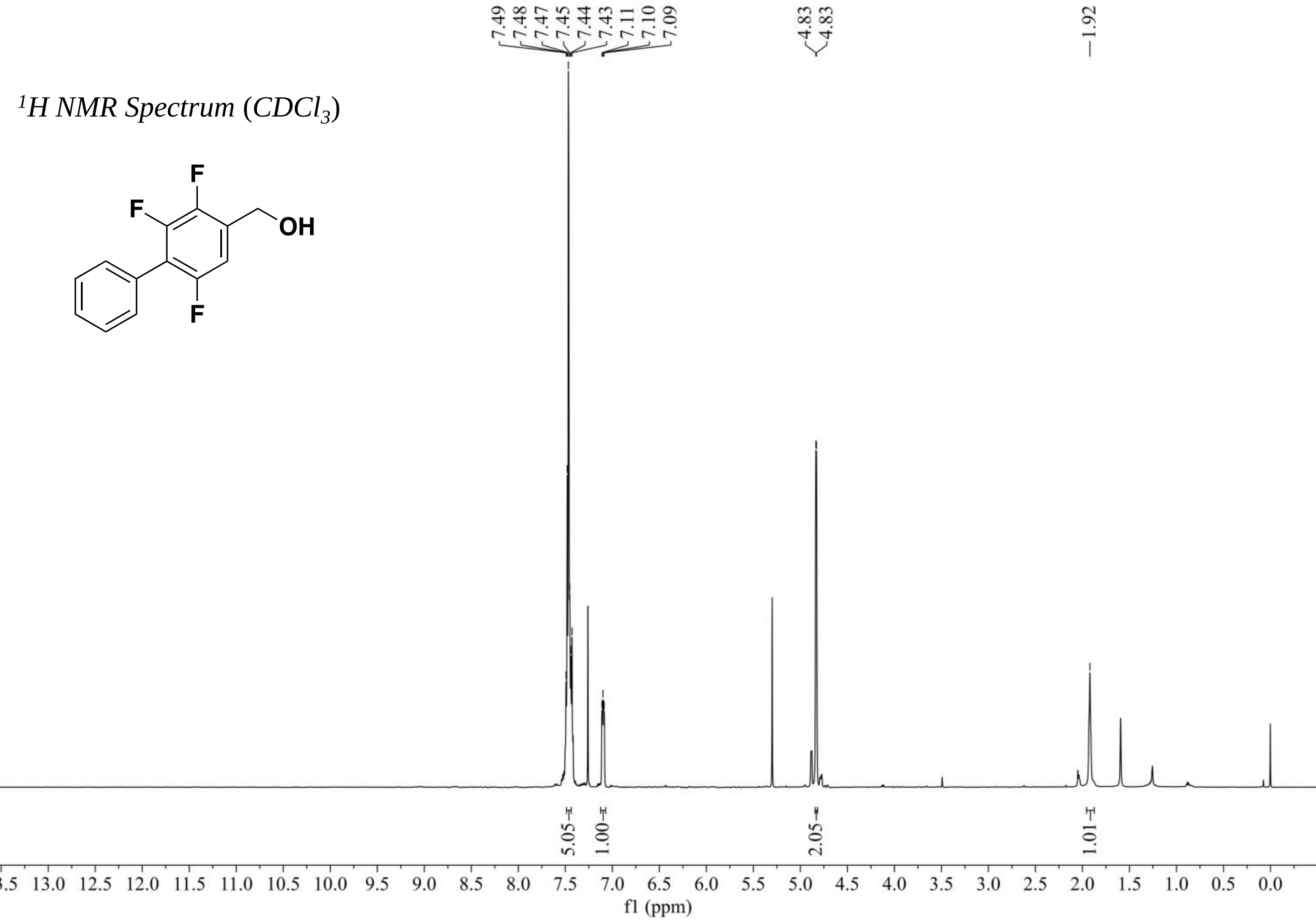

¹³C NMR Spectrum (*CDCl₃*)

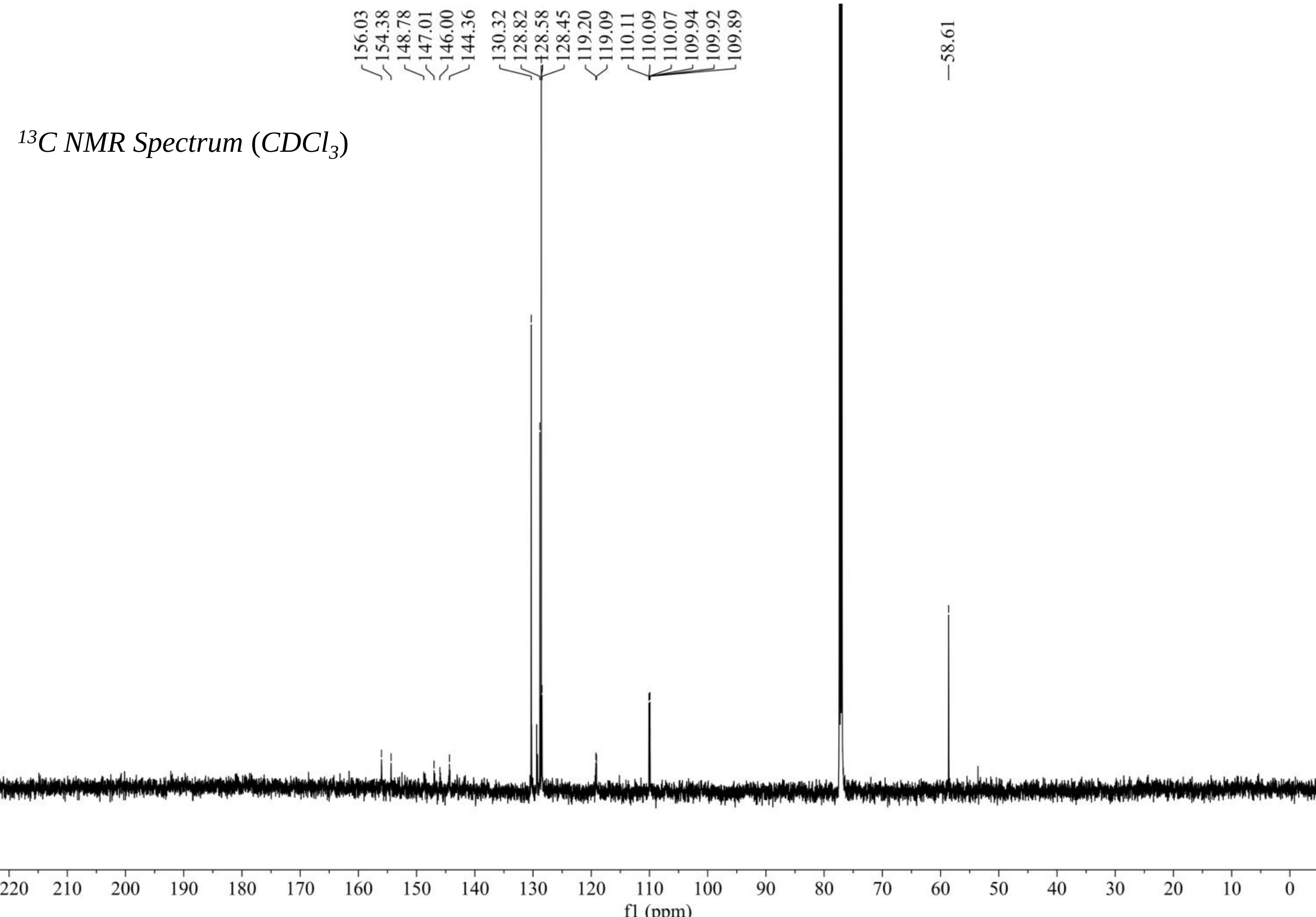

2,3,6-Trifluoro-[1,1'-biphenyl]-4-yl)methanol (**38b**)

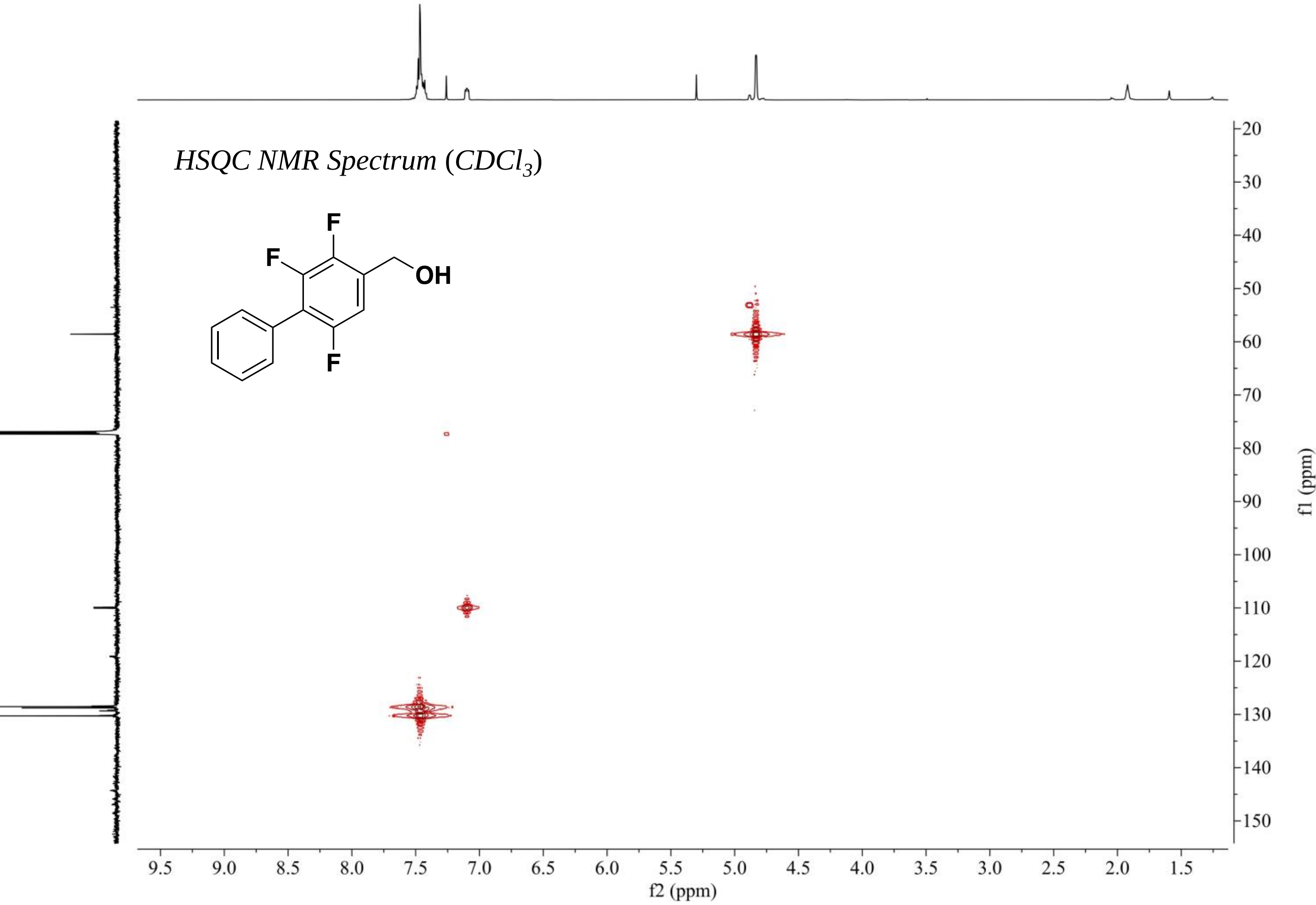

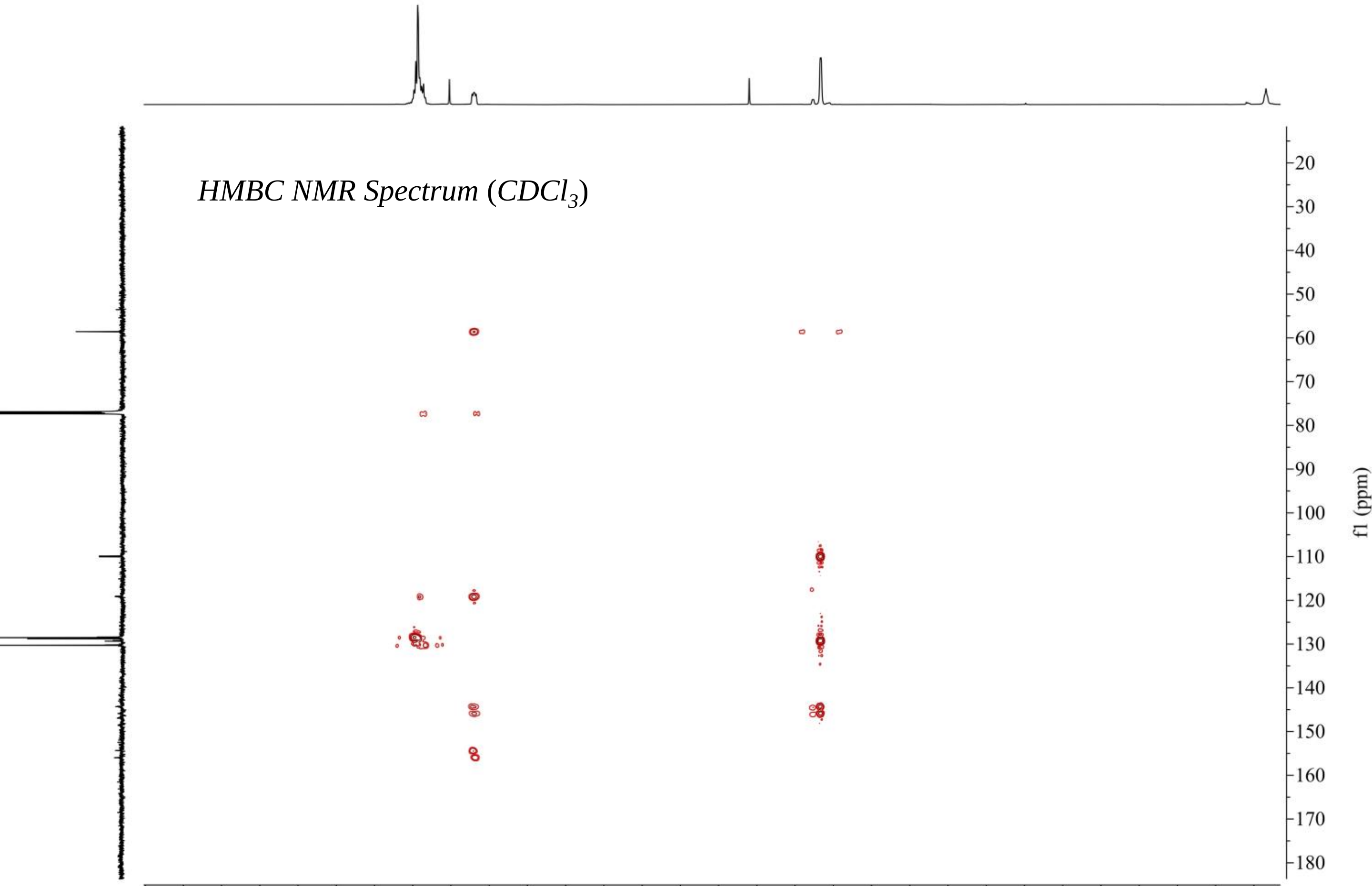

2,3,6-Trifluoro-[1,1'-biphenyl]-4-yl)methanol (**38b**)

^{19}F NMR Spectrum (*$CDCl_3$*)

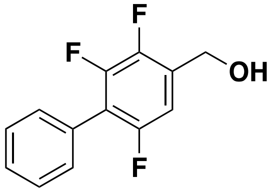

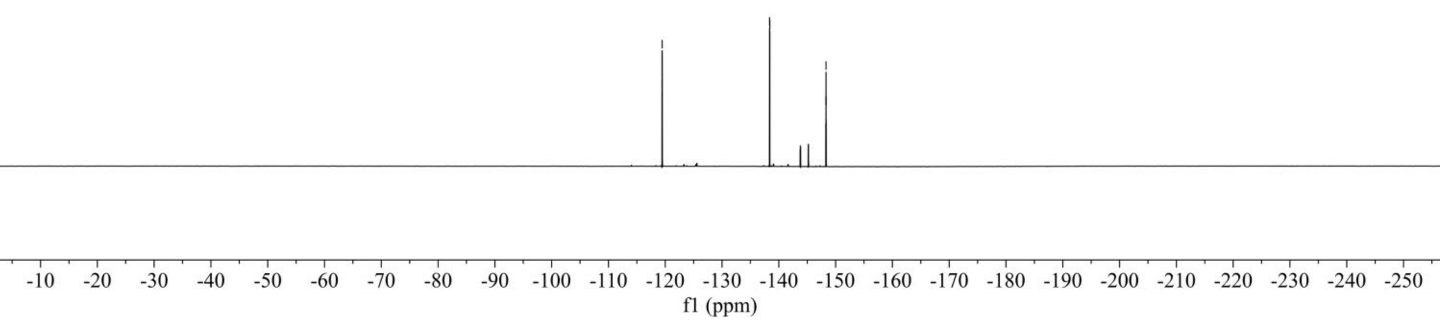

4-(Bromomethyl)-2,3,6-trifluoro-1,1'-biphenyl (**40b**)

^{1}H NMR Spectrum (*$CDCl_3$*)

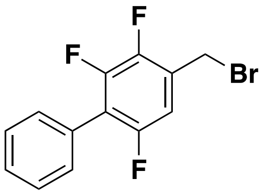

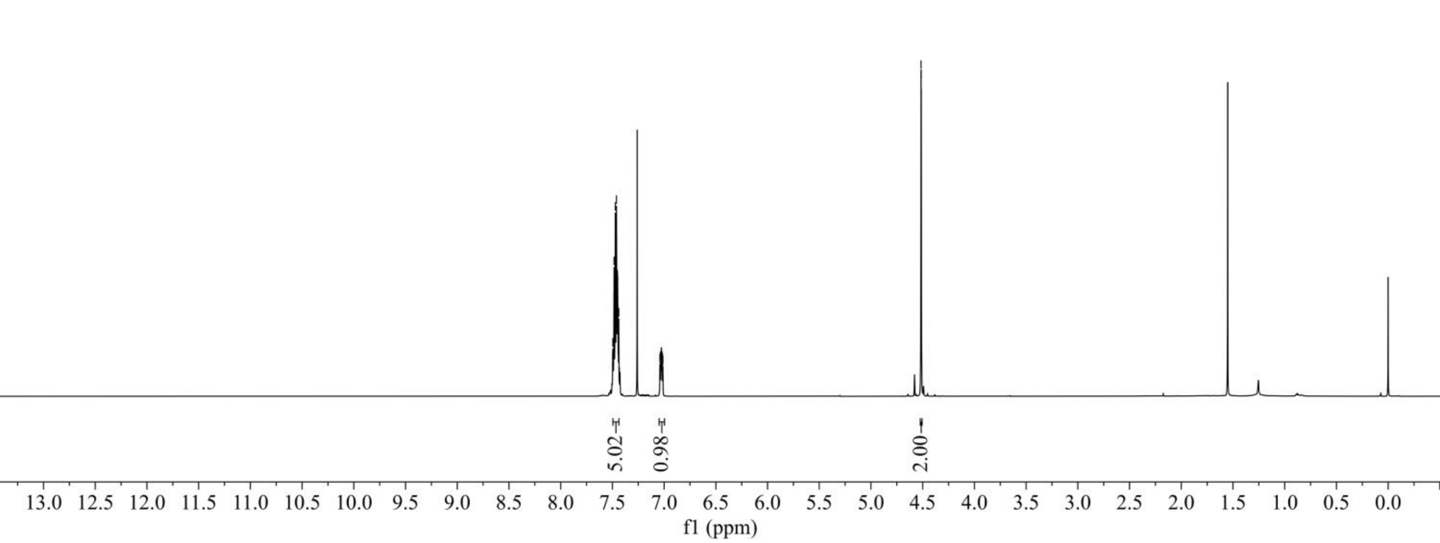

^{19}F NMR Spectrum (*$CDCl_3$*)

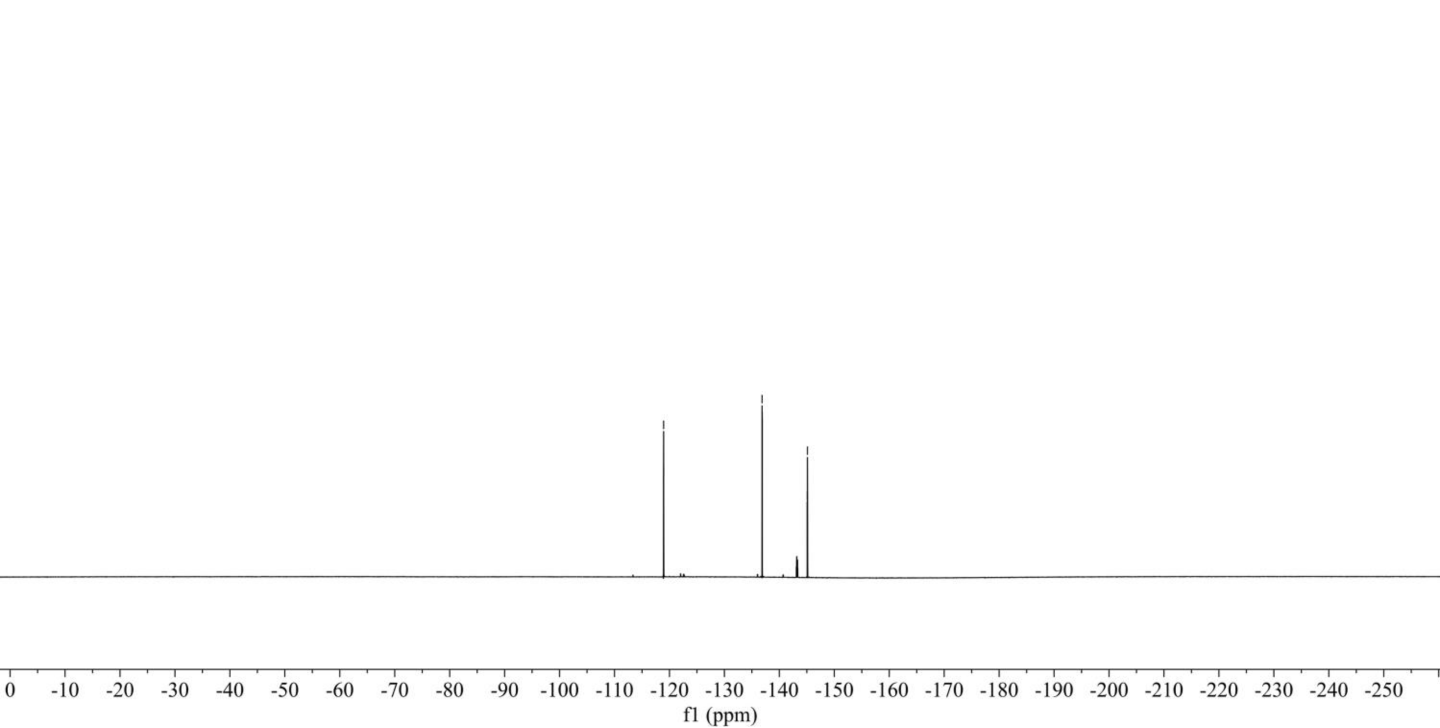

Triphenyl(3,5,6-tetrafluoro-[1,1'-biphenyl]-4-yl)methyl)phosphonium salt (**41b**)

^{1}H NMR Spectrum (*$CDCl_3$*)

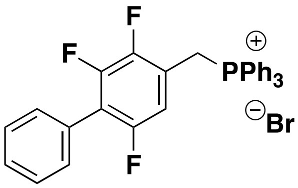

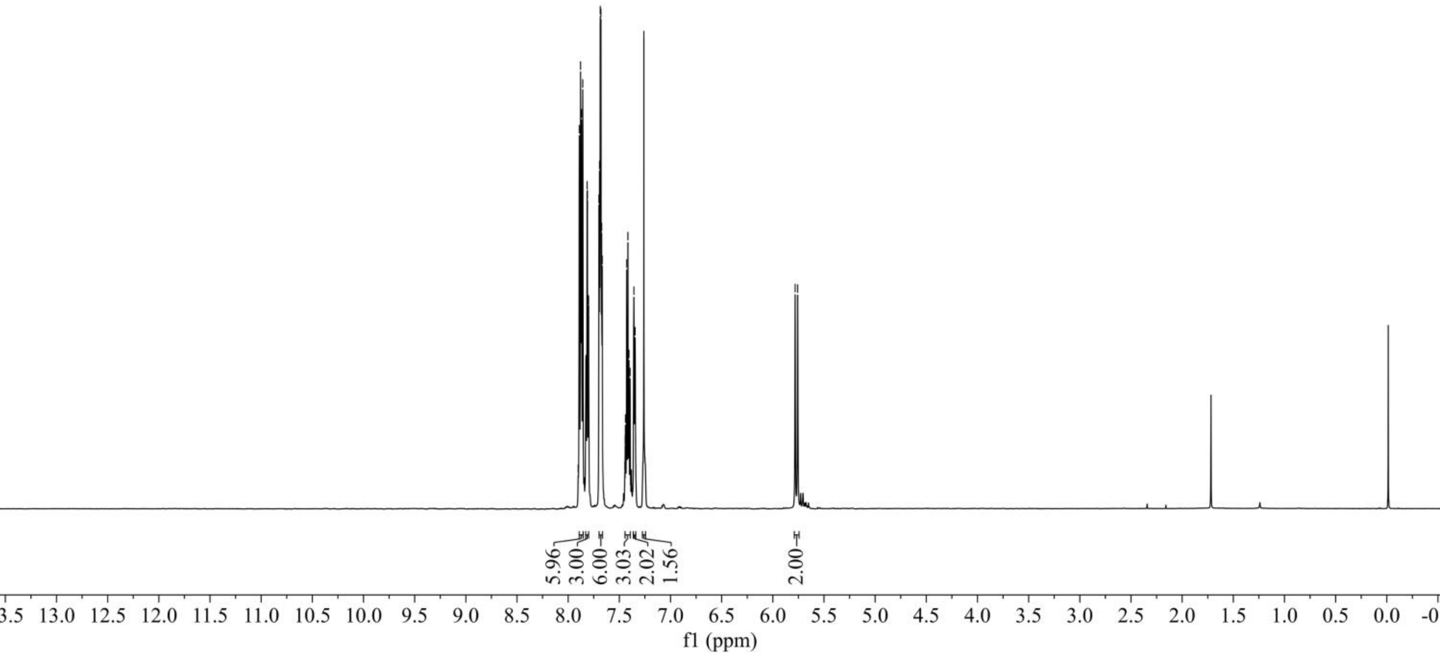

^{19}F NMR Spectrum (*$CDCl_3$*)

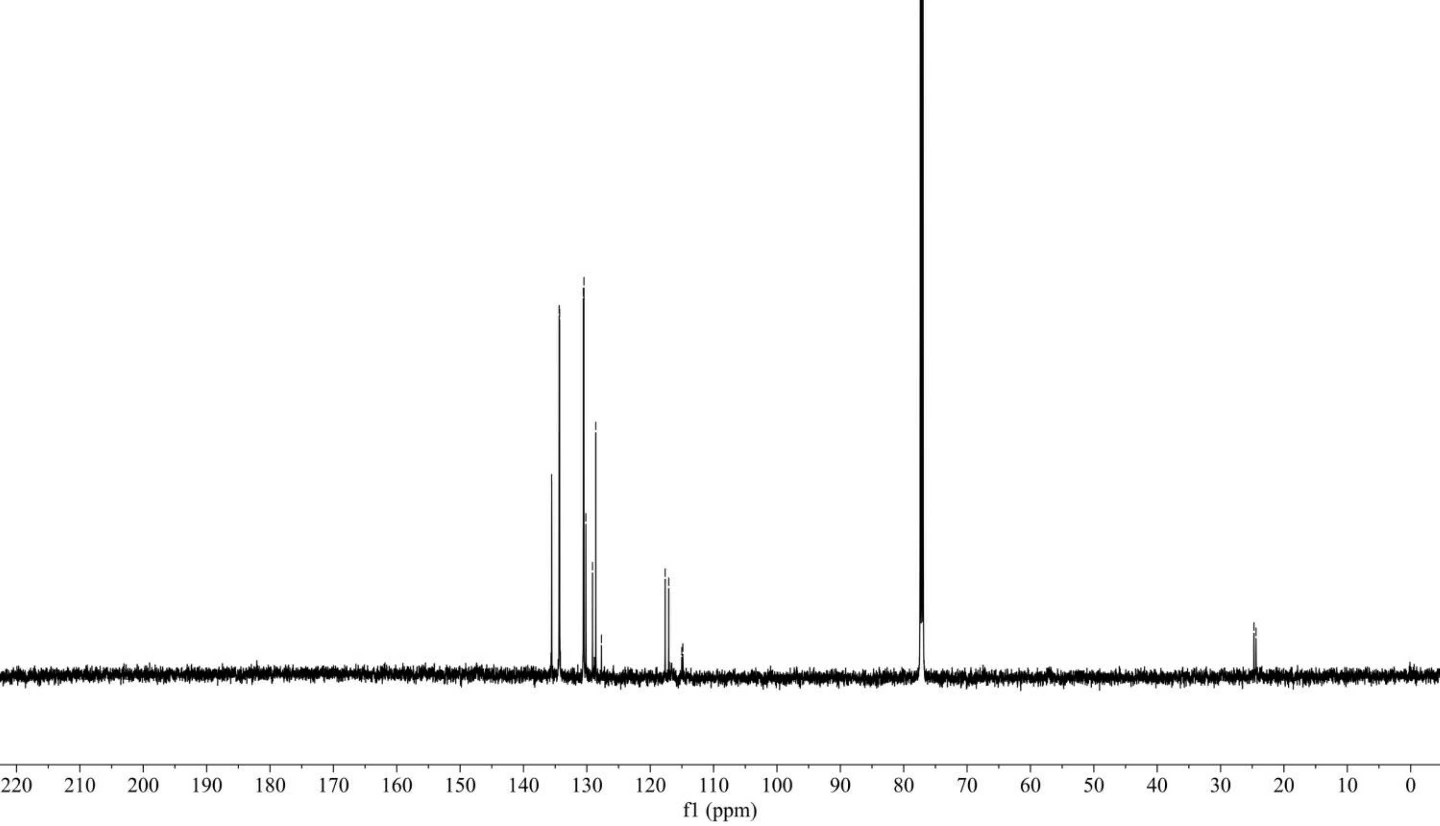

Triphenyl(3,5,6-tetrafluoro-[1,1'-biphenyl]-4-yl)methyl)phosphonium salt (**41b**)

^{31}P NMR Spectrum (*$CDCl_3$*)

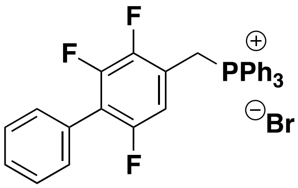

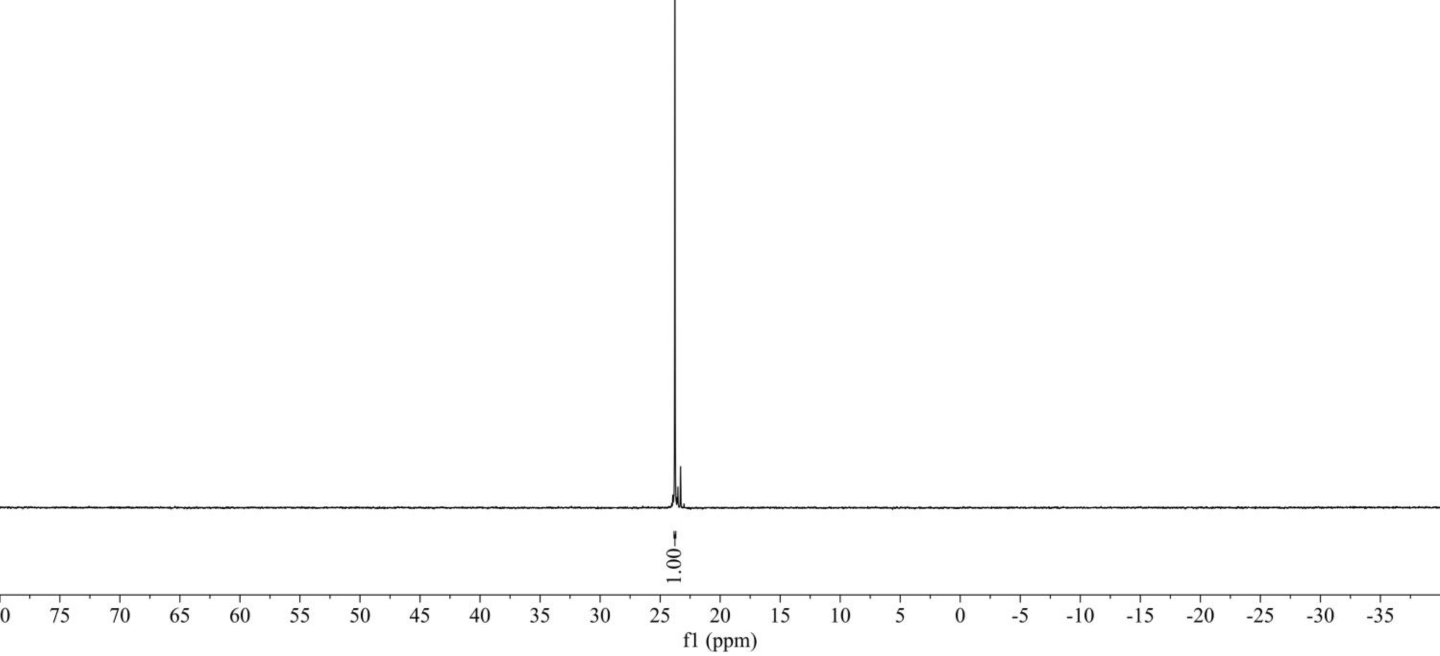

(*E*)-2-((4-methoxybenzyl)oxy)-3-(2-(2,3,6-trifluoro-[1,1'-biphenyl]-4-yl)vinyl)pyrazolo[1,5-*a*]pyridine (**28b**)

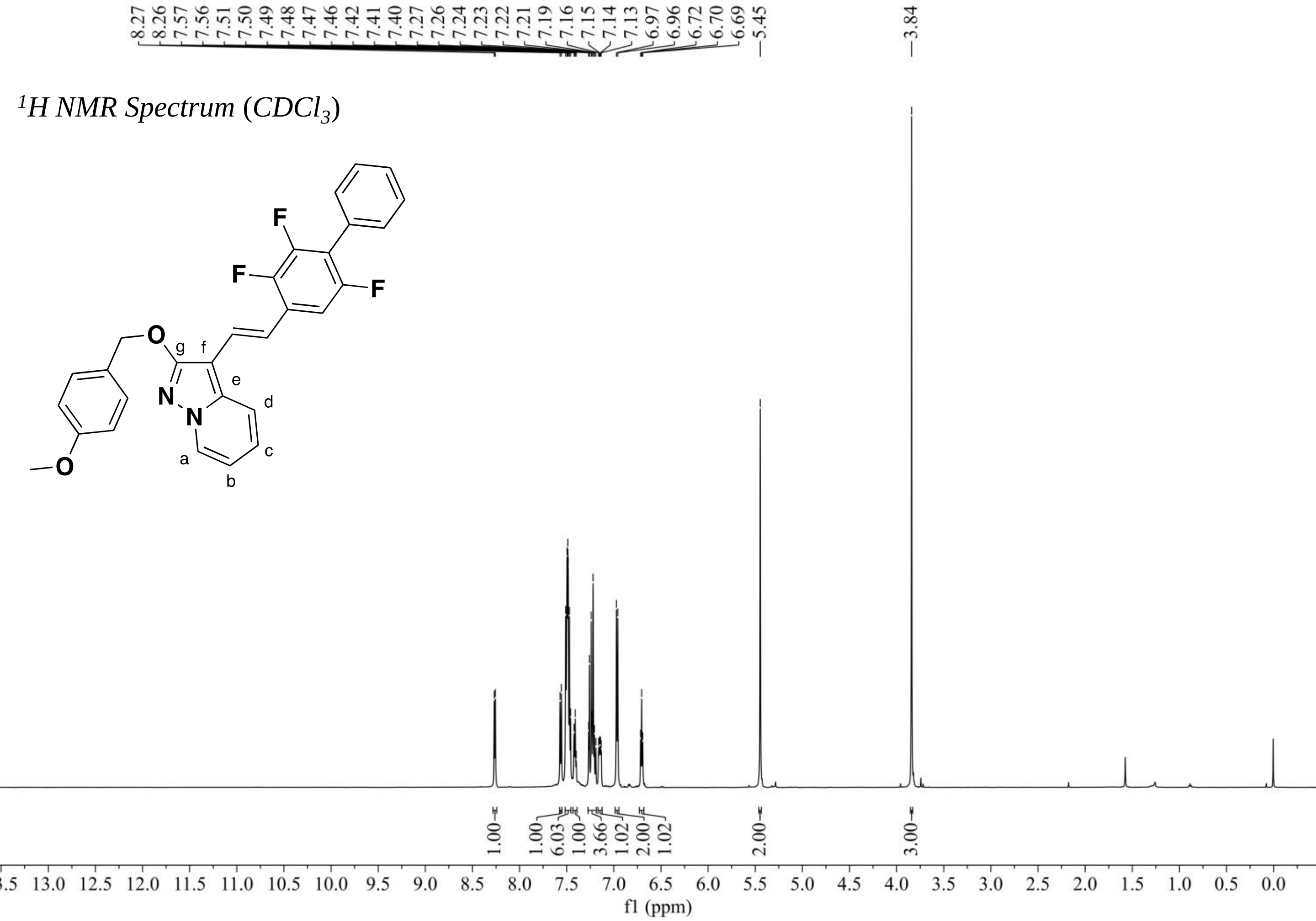

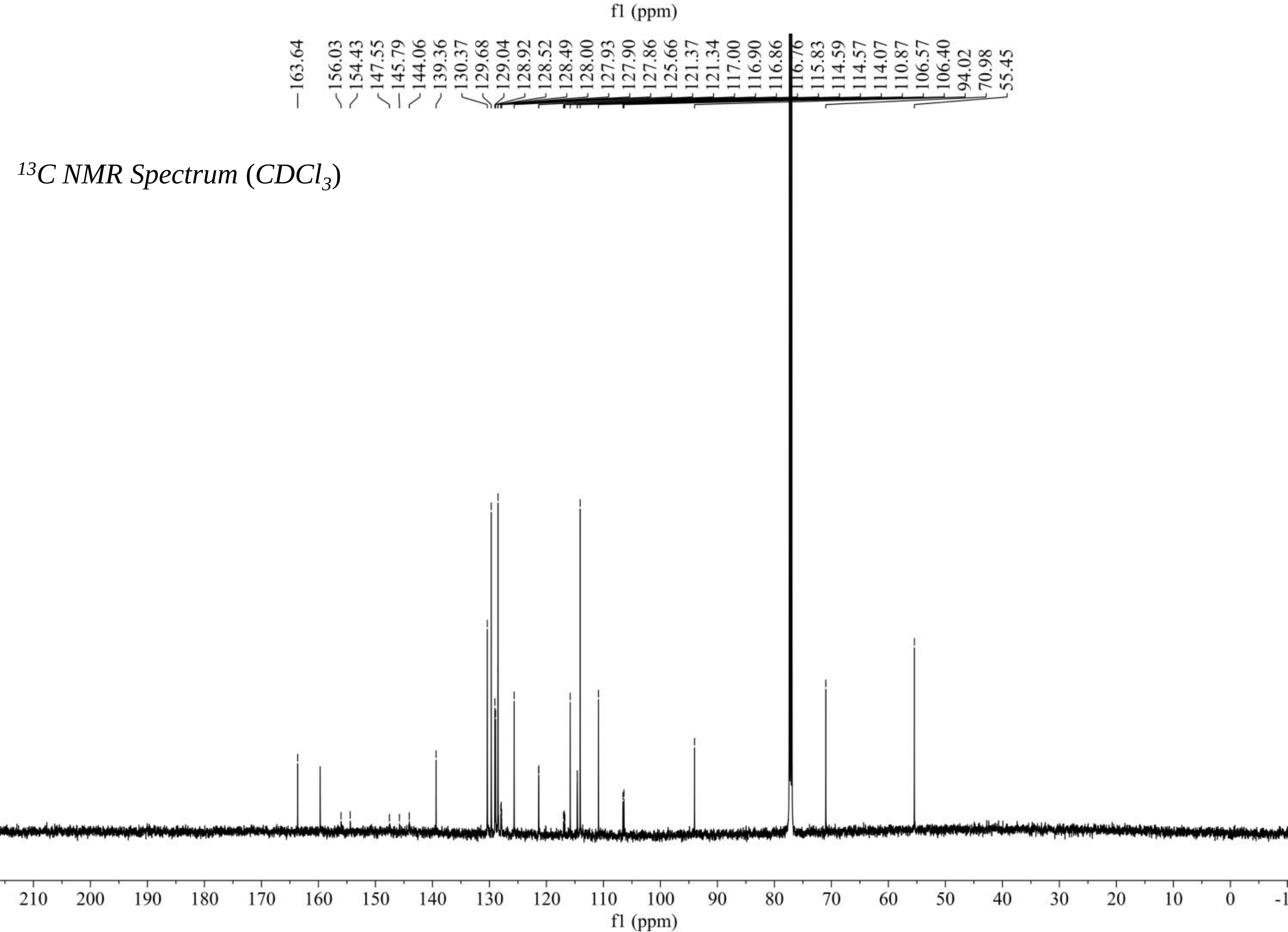

(E)-2-((4-methoxybenzyl)oxy)-3-(2-(2,3,6-trifluoro-[1,1'-biphenyl]-4-yl)vinyl)pyrazolo[1,5-a]pyridine (**28b**)

^{19}F NMR Spectrum (*$CDCl_3$*)

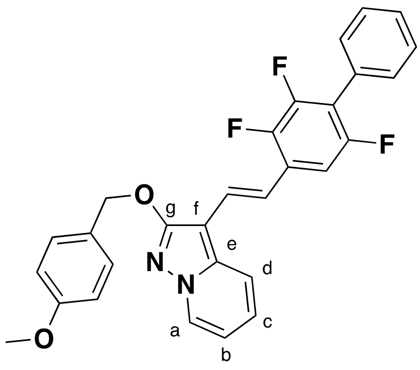

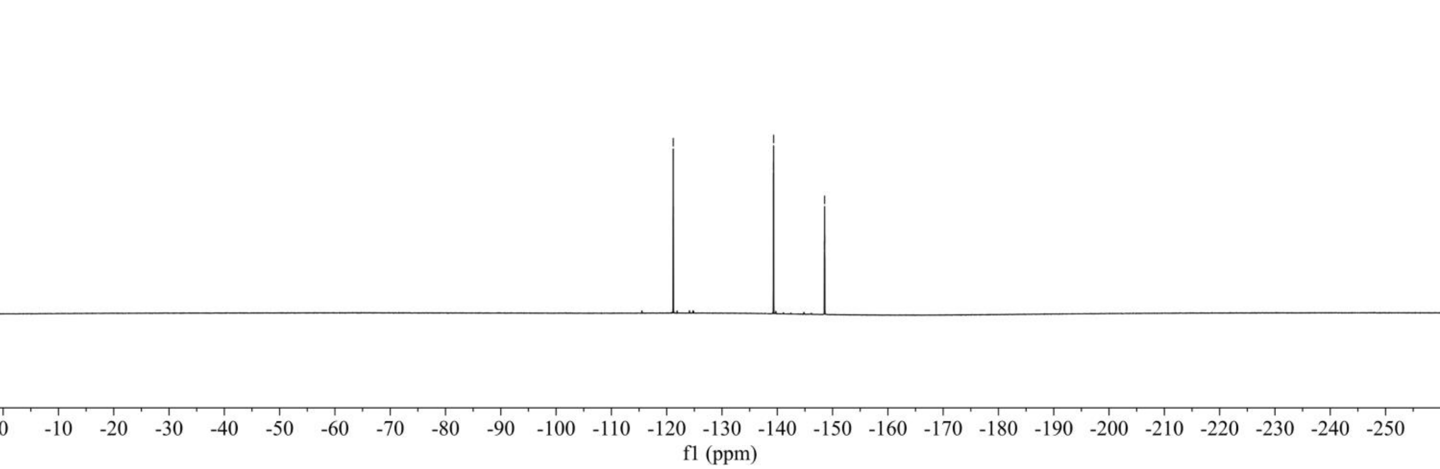

((*E*)-3-(2-(2,3,6-trifluoro-[1,1'-biphenyl]-4-yl)vinyl)pyrazolo[1,5-*a*]pyridin-2-ol (**11b**)

¹H NMR Spectrum (*DMF*)

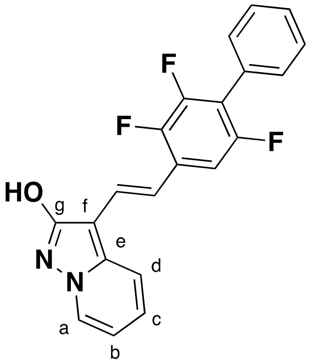

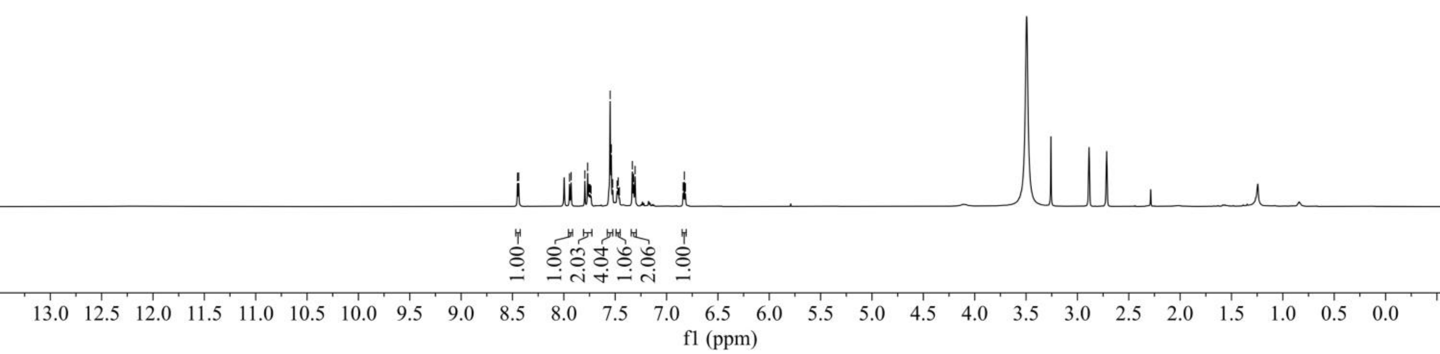

¹³C NMR Spectrum (*DMF*)

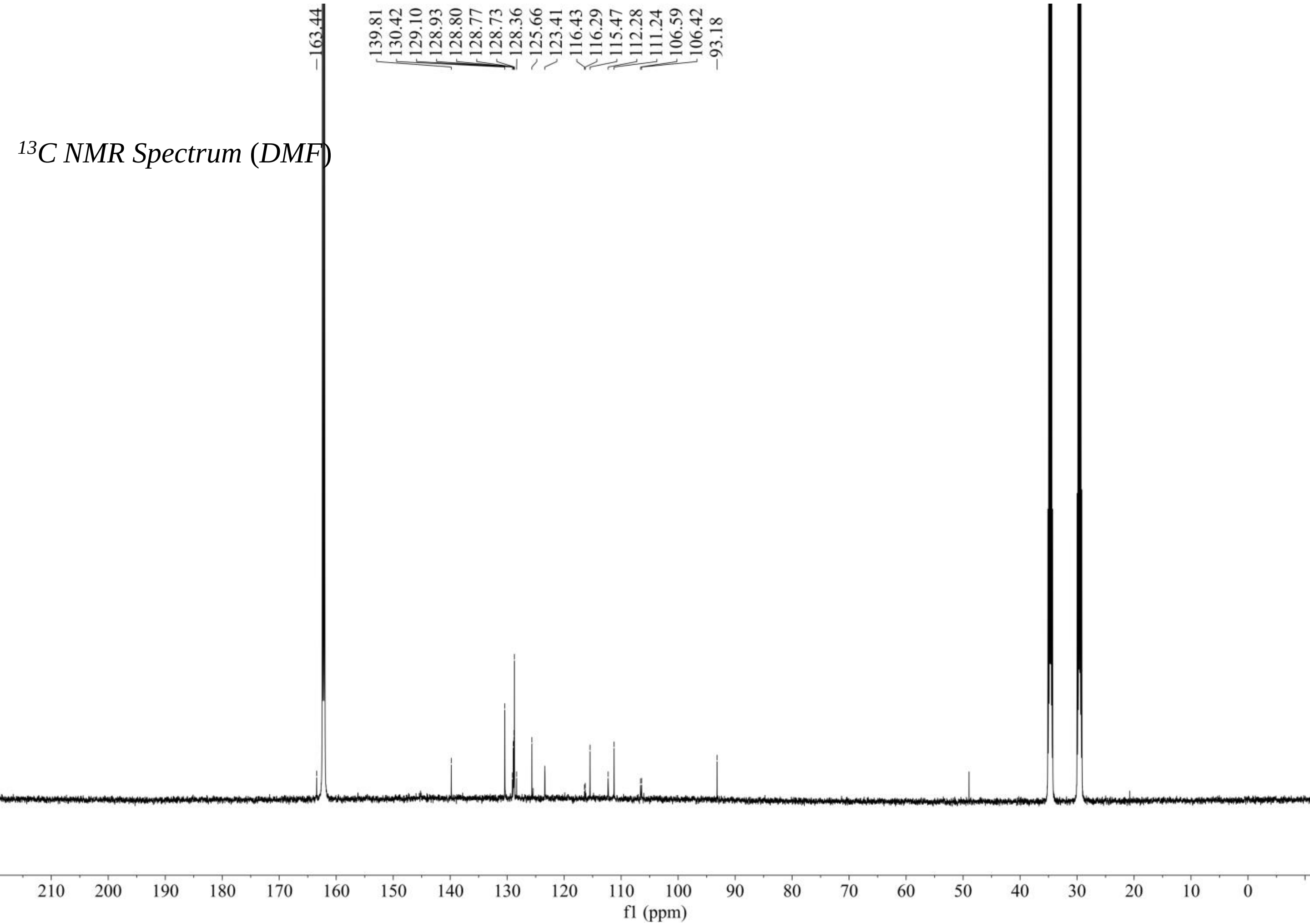

((E)-3-(2-(2,3,6-trifluoro-[1,1'-biphenyl]-4-yl)vinyl)pyrazolo[1,5-a]pyridin-2-ol (**11b**)

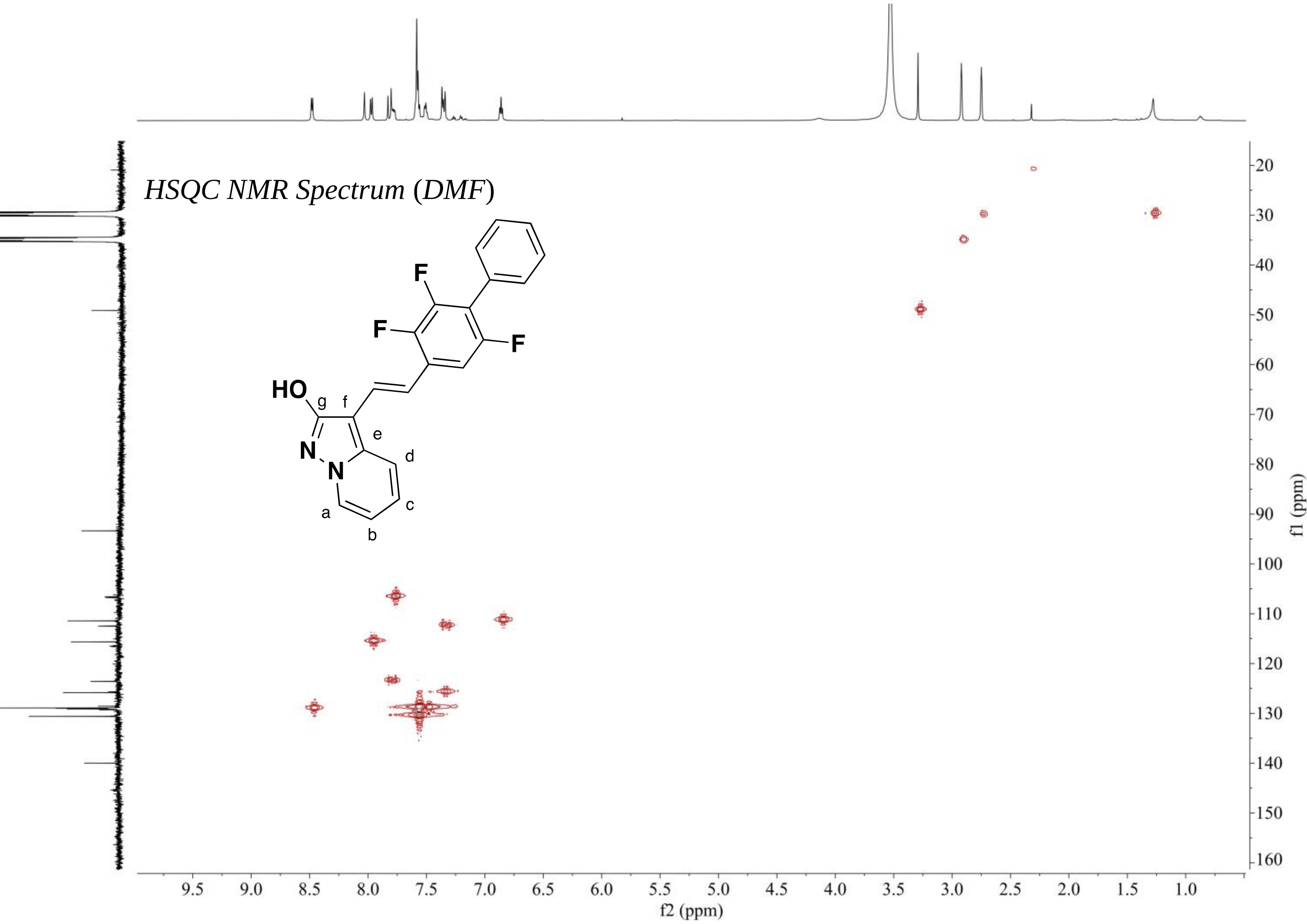

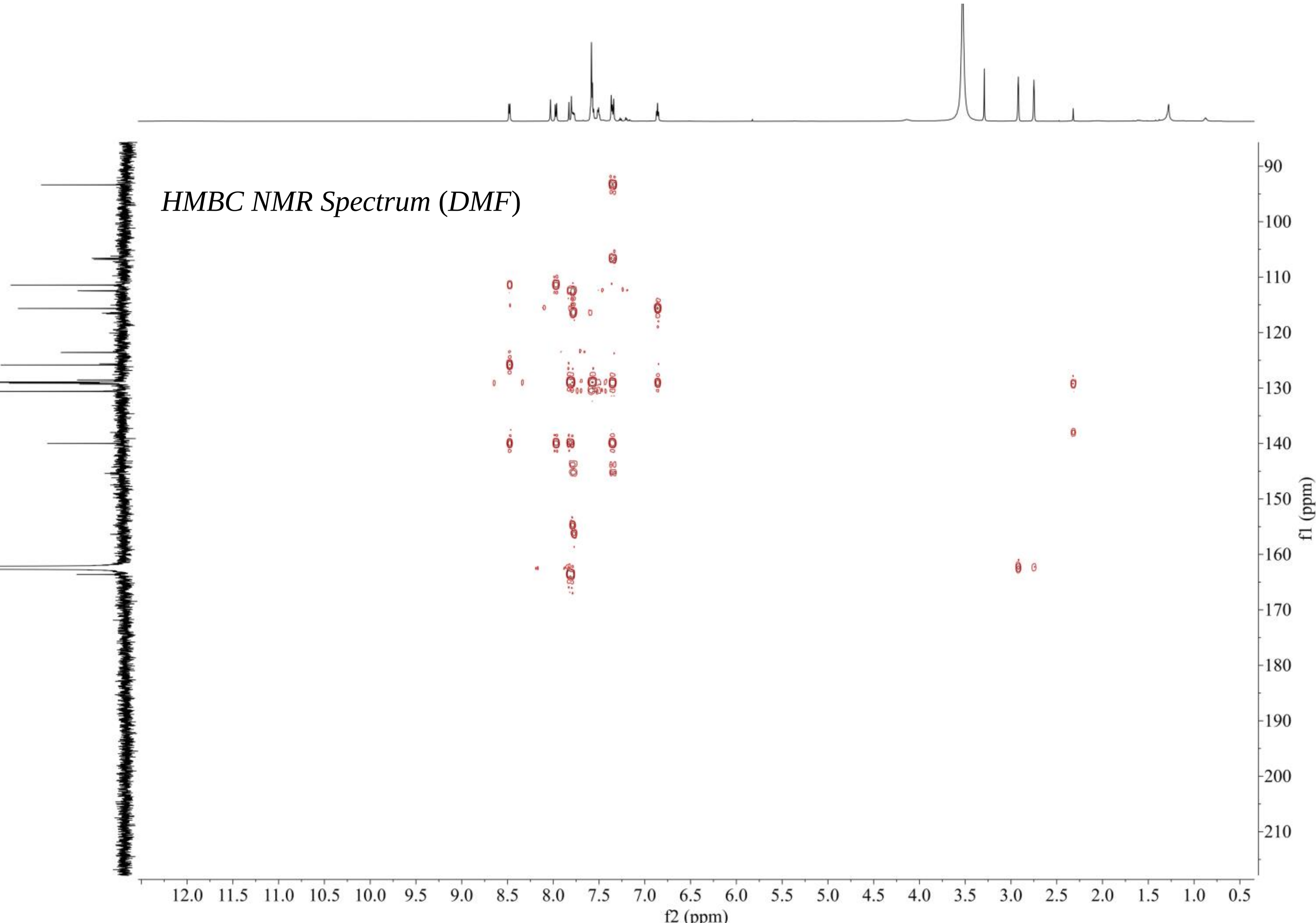

((E)-3-(2-(2,3,6-trifluoro-[1,1'-biphenyl]-4-yl)vinyl)pyrazolo[1,5-a]pyridin-2-ol (**11b**)

^{19}F NMR Spectrum (*DMF*)

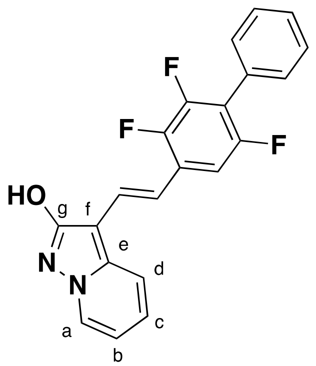

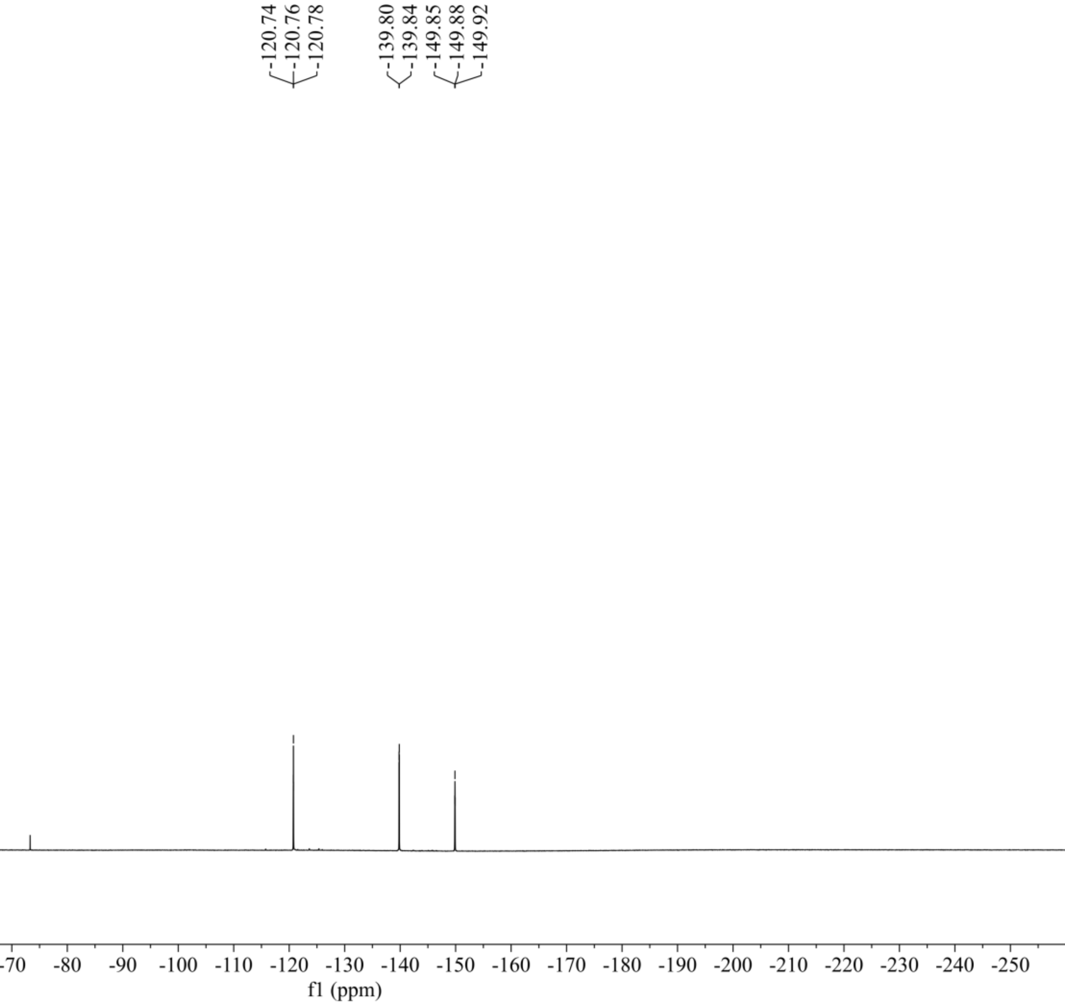

2,3,5,6-Tetrafluoro-[1,1'-biphenyl]-4-carbaldehyde (**39**)

^{1}H NMR Spectrum ($CDCl_3$)

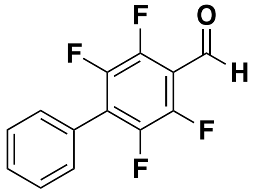

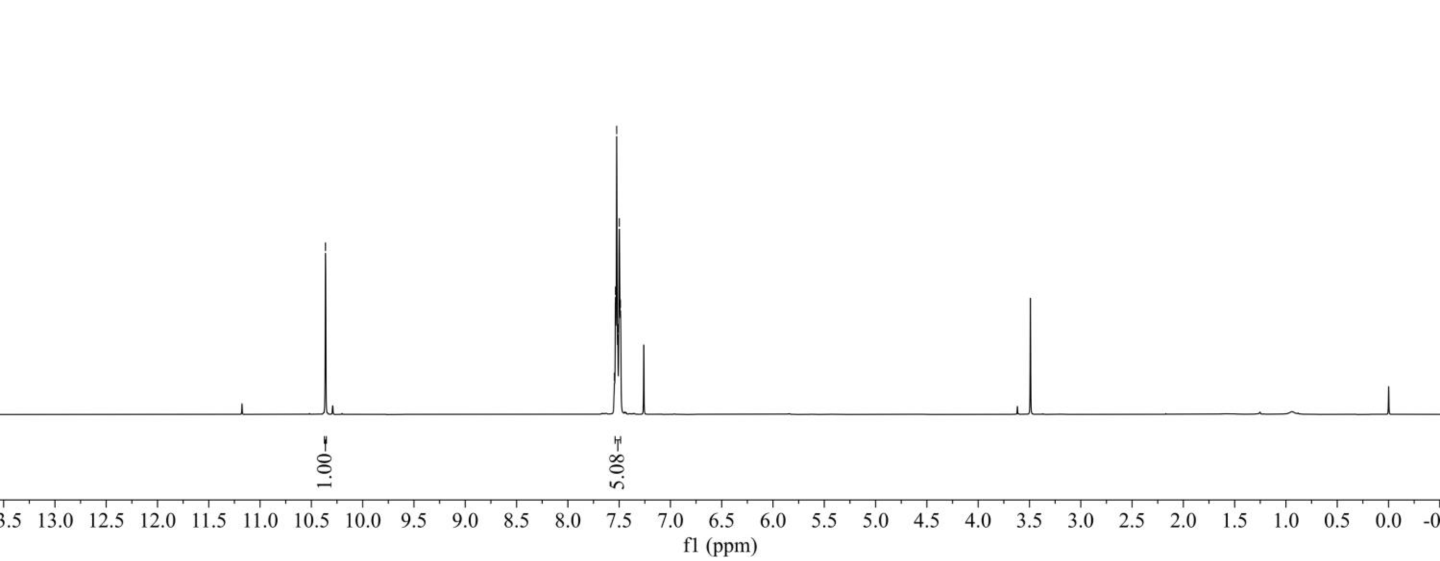

^{13}C NMR Spectrum ($CDCl_3$)

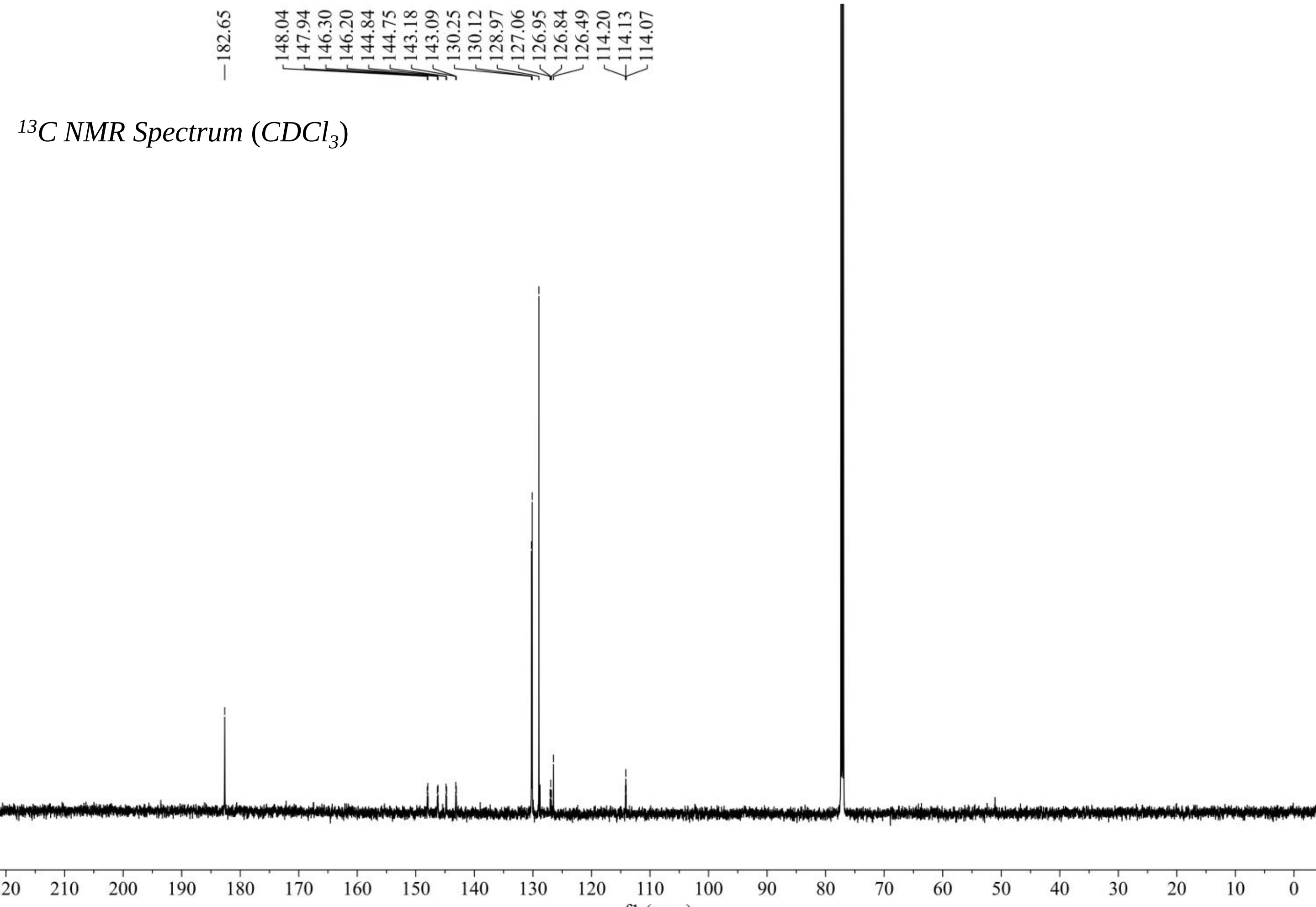

2,3,5,6-Tetrafluoro-[1,1'-biphenyl]-4-carbaldehyde (**39**)

^{19}F NMR Spectrum (*$CDCl_3$*)

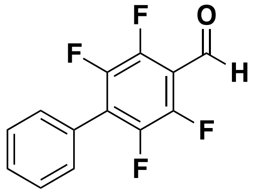

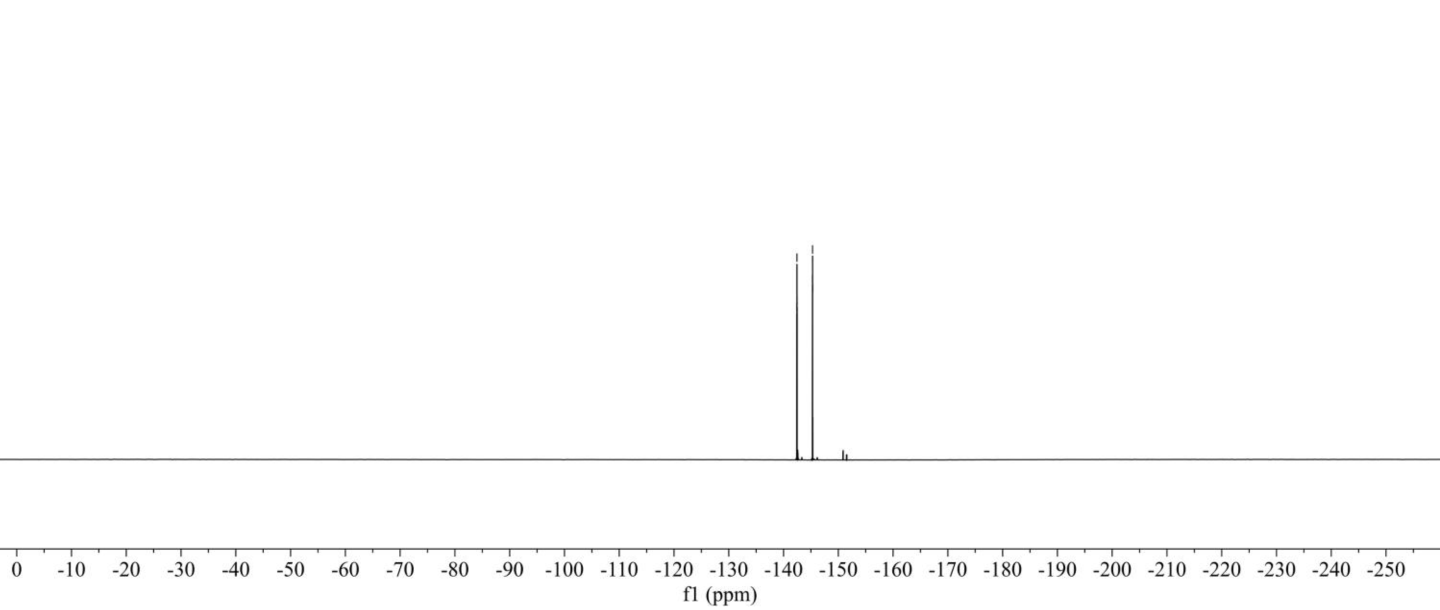

(2,3,5,6-Tetrafluoro-[1,1'-biphenyl]-4-yl)methanol (**38a**)

^{1}H NMR Spectrum (*CDCl$_3$*)

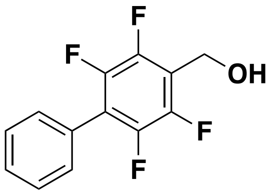

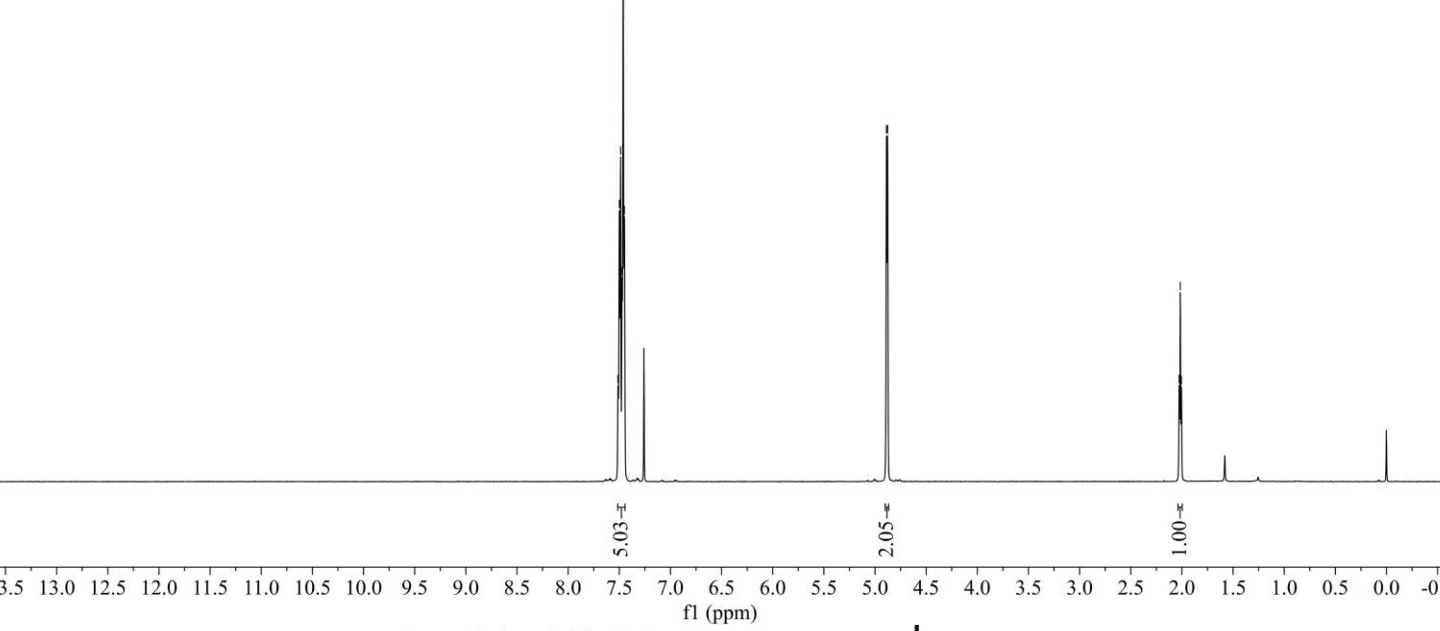

^{13}C NMR Spectrum (*CDCl$_3$*)

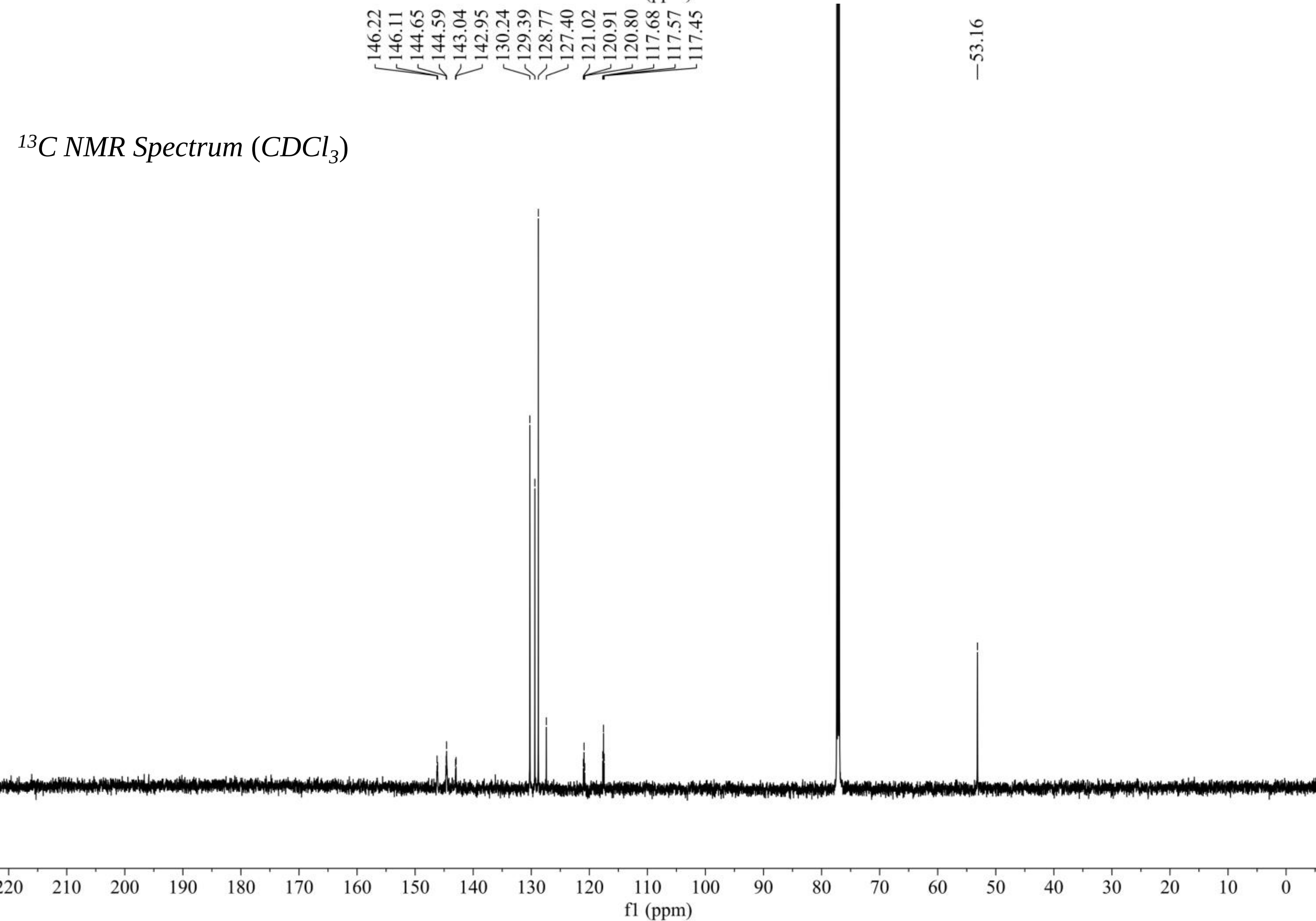

(2,3,5,6-Tetrafluoro-[1,1'-biphenyl]-4-yl)methanol (**38a**)

^{19}F NMR Spectrum (*$CDCl_3$*)

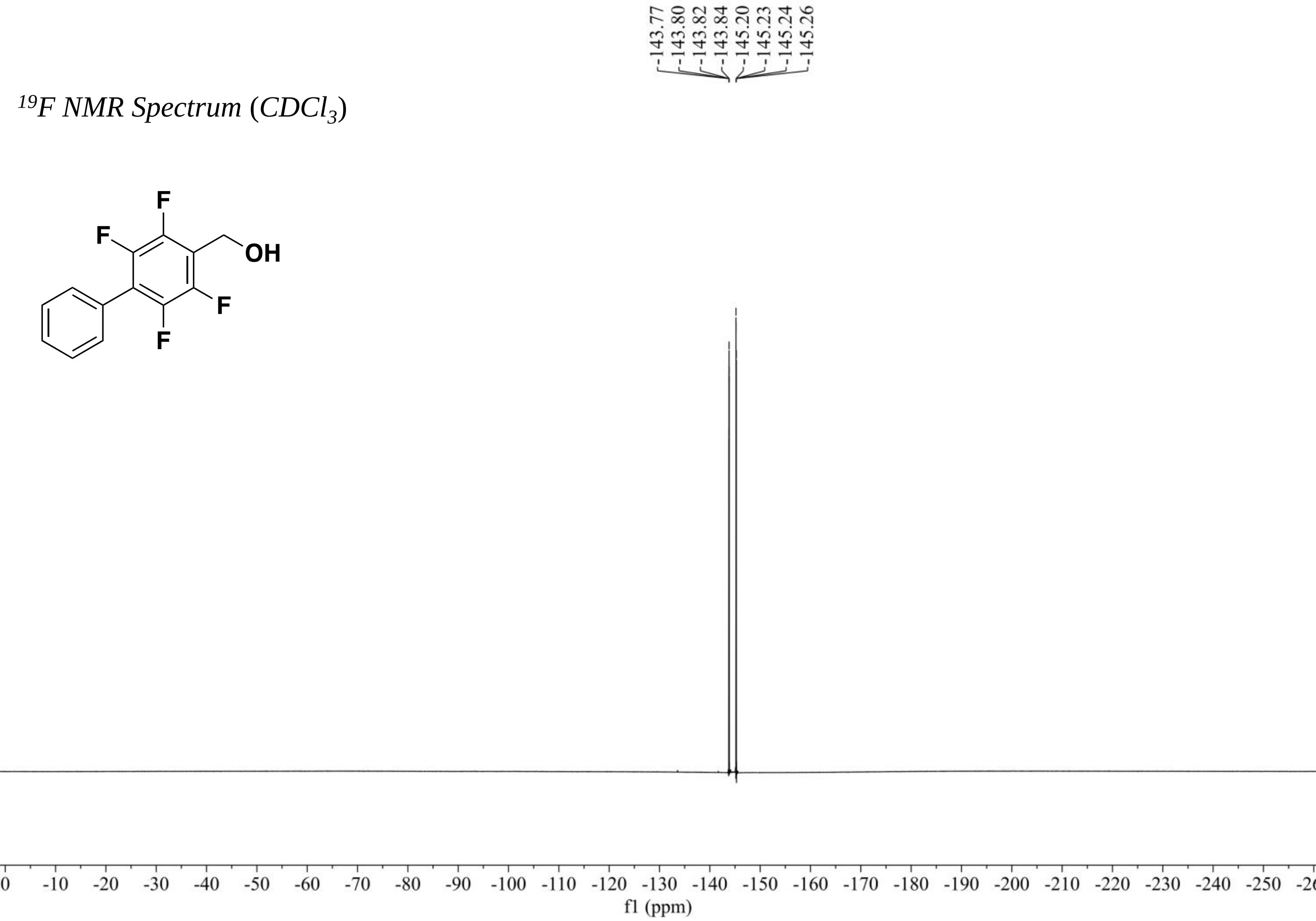

4-(Bromomethyl)-2,3,5,6-tetrafluoro-1,1'-biphenyl (**40a**)

^{1}H NMR Spectrum ($CDCl_3$)

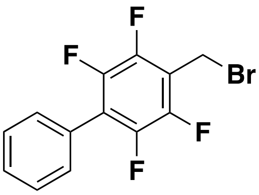

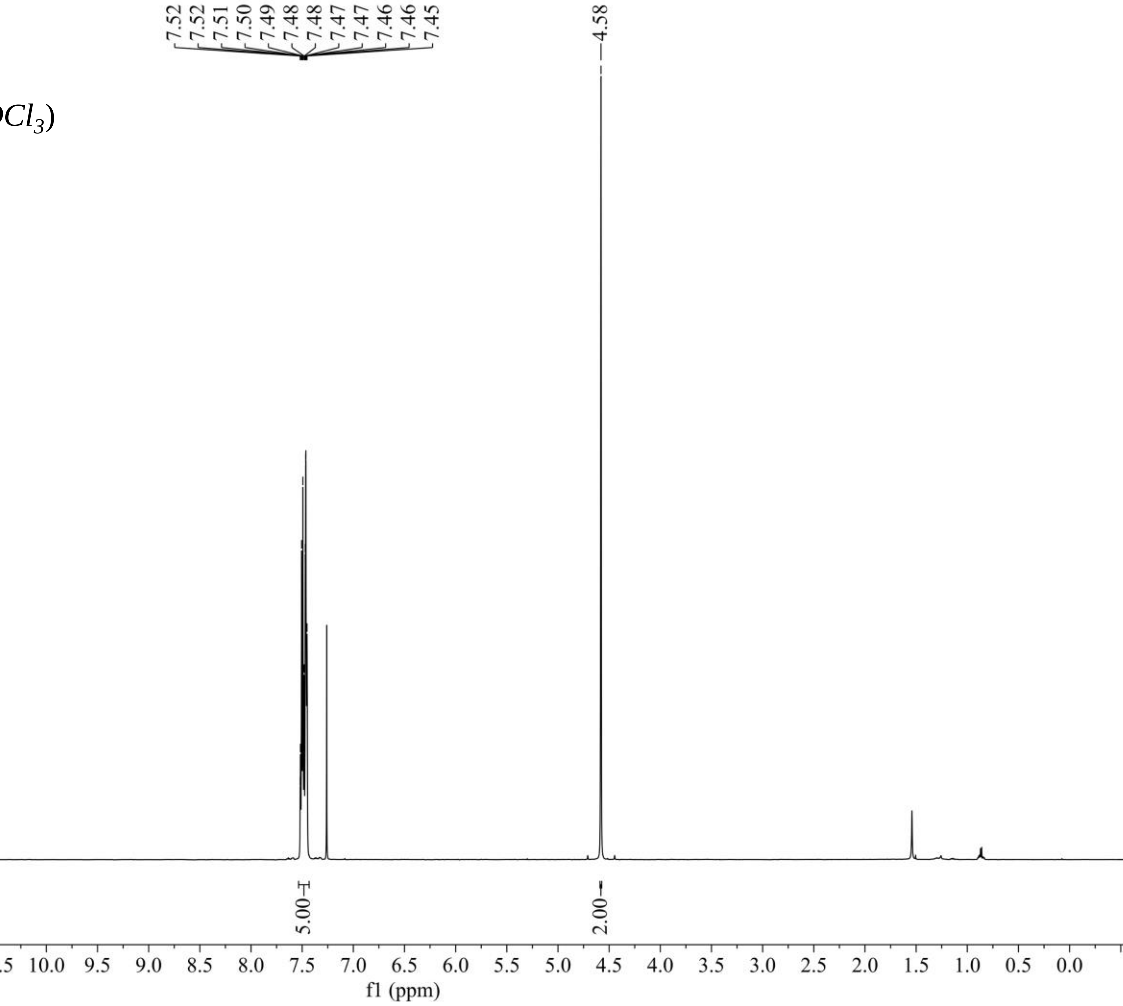

^{13}C NMR Spectrum ($CDCl_3$)

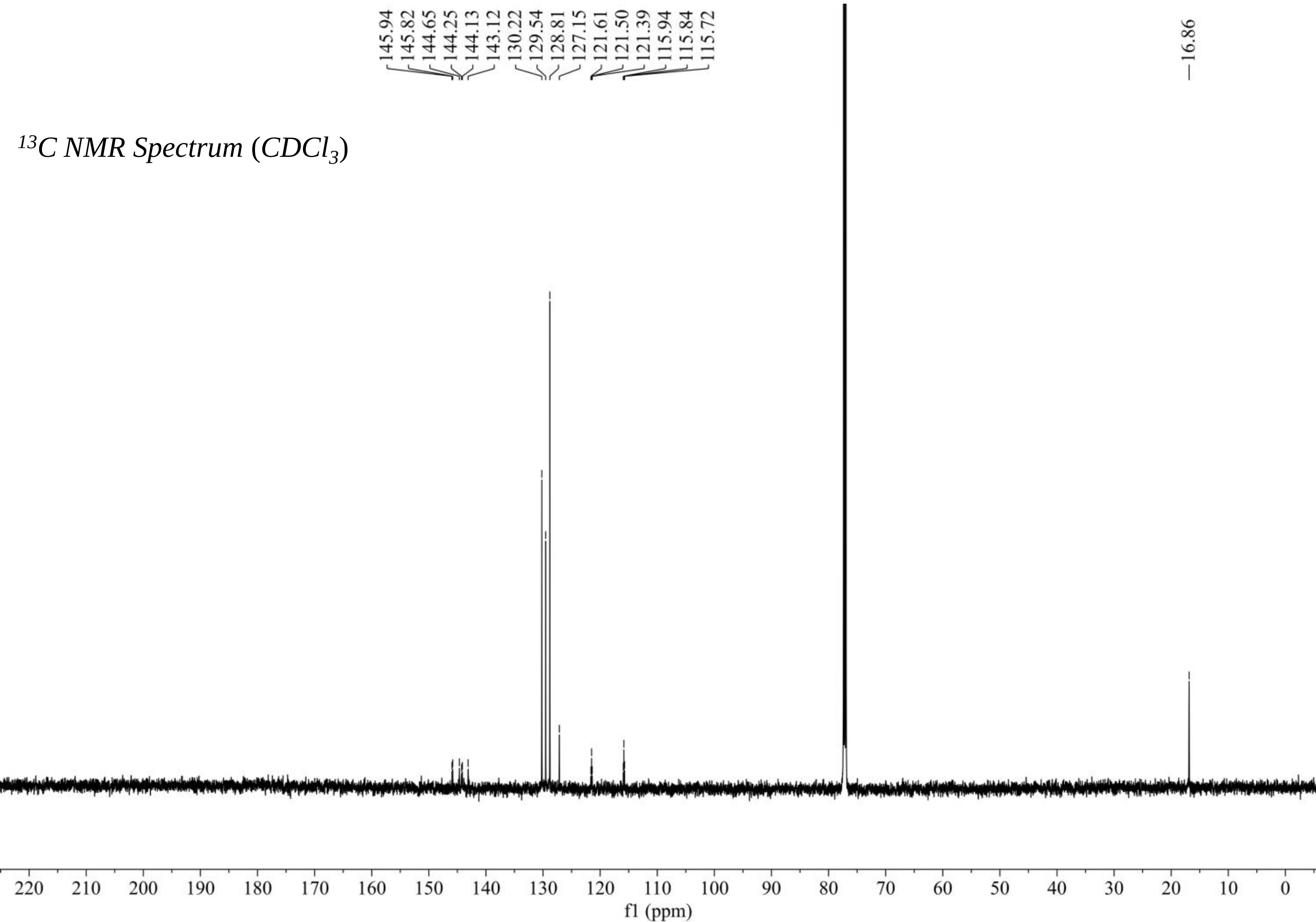

4-(Bromomethyl)-2,3,5,6-tetrafluoro-1,1'-biphenyl (**40a**)

^{19}F NMR Spectrum (*$CDCl_3$*)

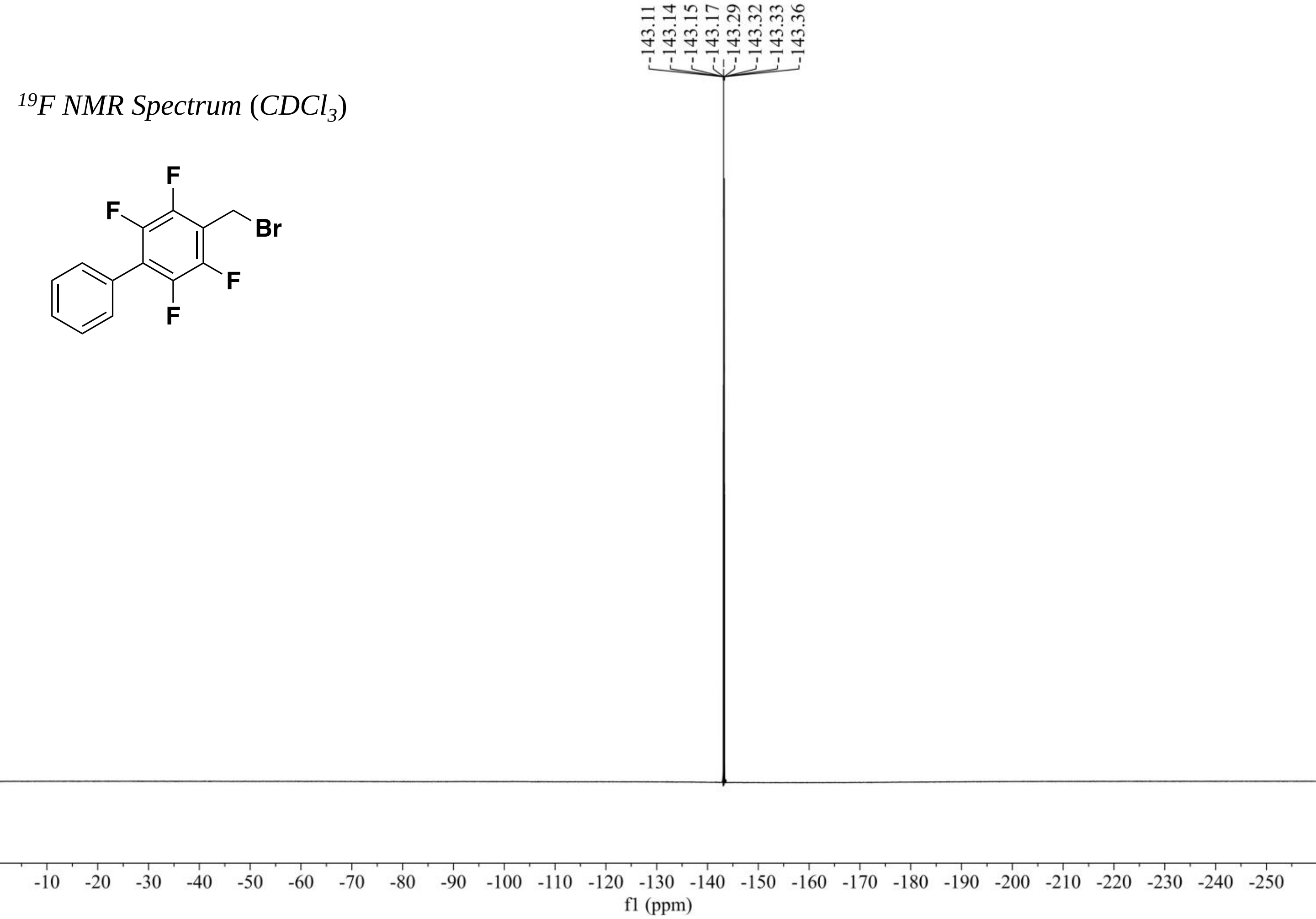

Triphenyl((2,3,5,6-tetrafluoro-[1,1'-biphenyl]-4-yl)methyl)phosphonium salt (**41a**)

^{1}H NMR Spectrum (*$CDCl_3$*)

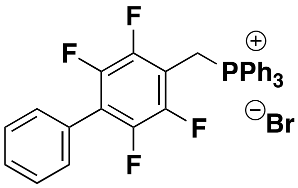

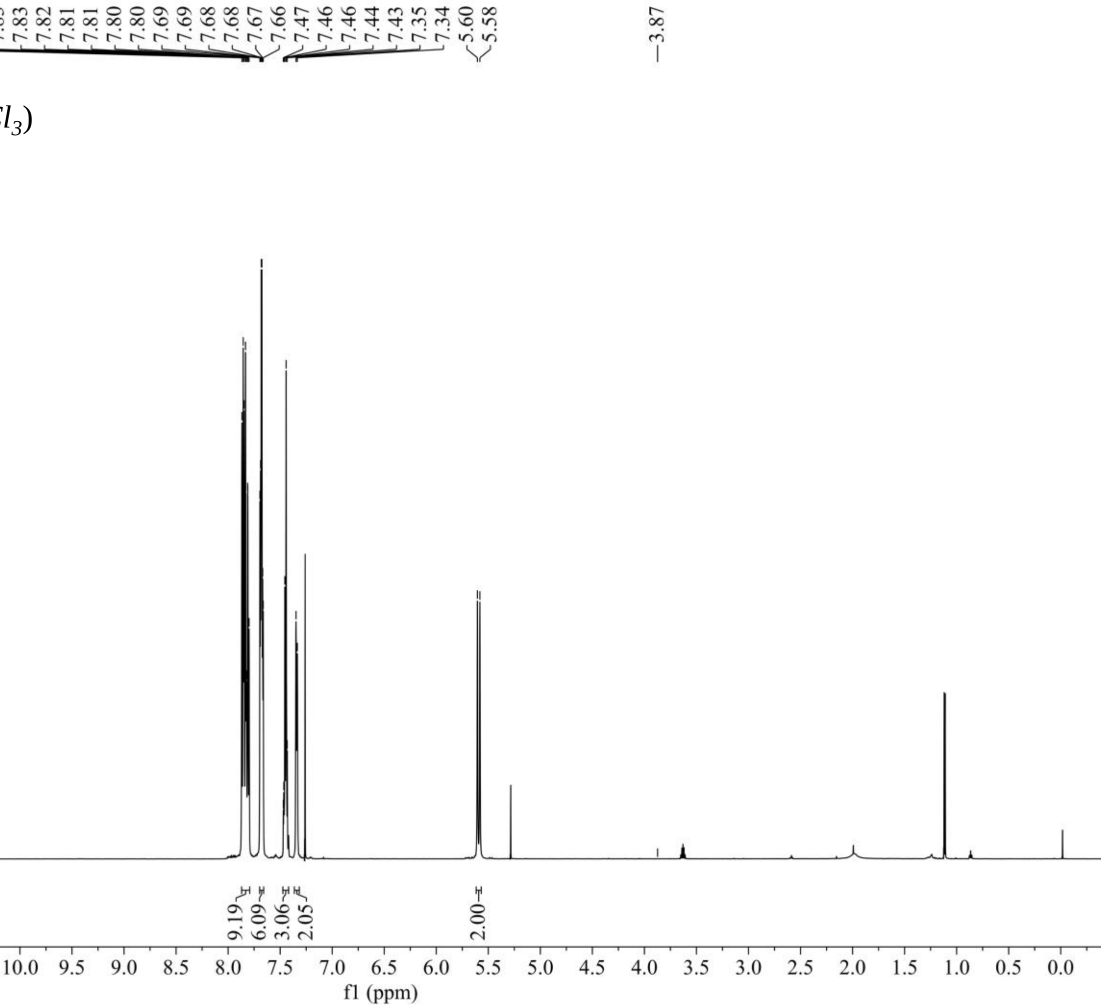

^{13}C NMR Spectrum (*$CDCl_3$*)

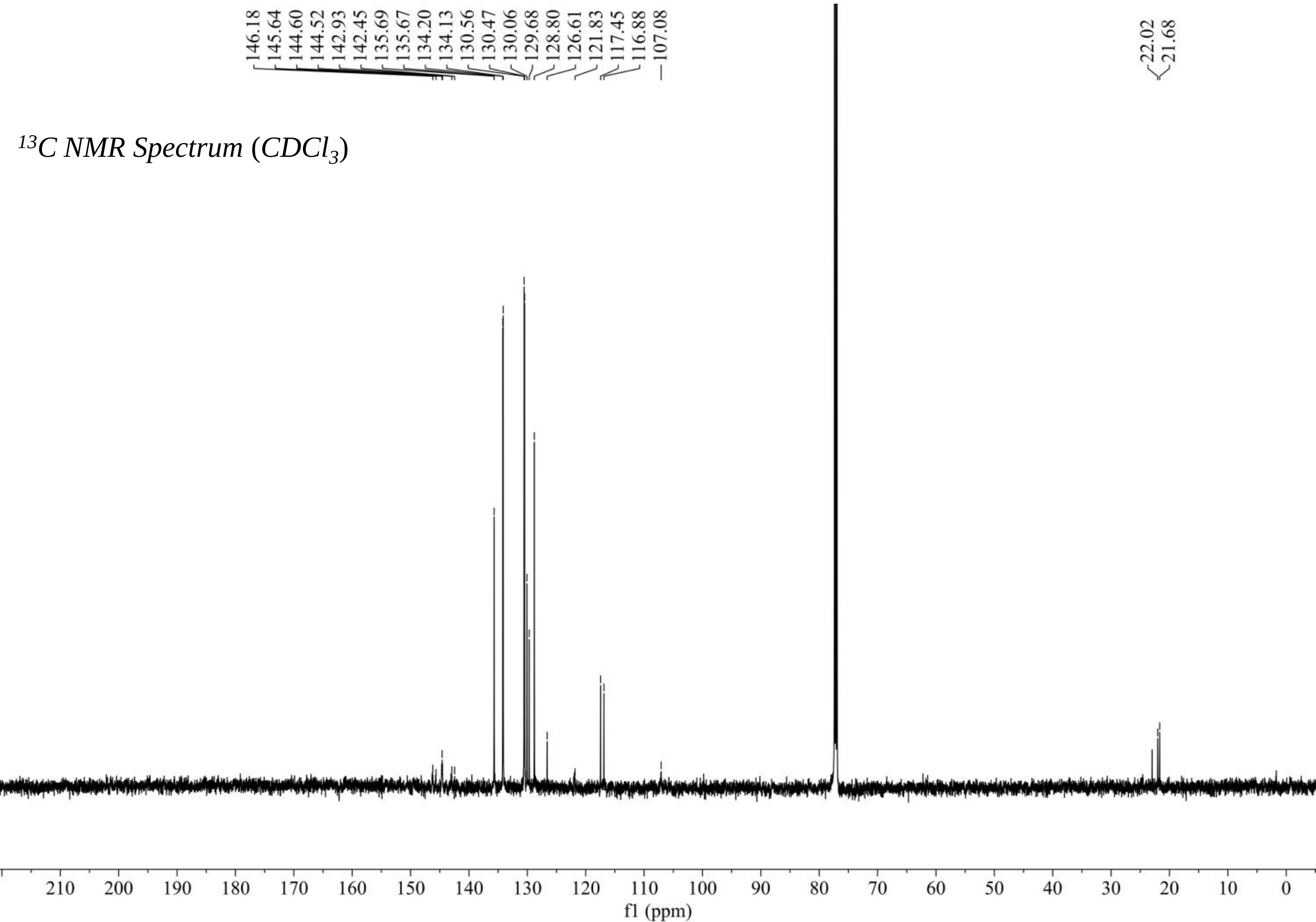

(E)-2-(Benzyloxy)-3-(2-(2,3,5,6-tetrafluoro-[1,1'-biphenyl]-4-yl)vinyl)pyrazolo[1,5-a]pyridine (**28a**)

^{1}H NMR Spectrum ($CDCl_3$)

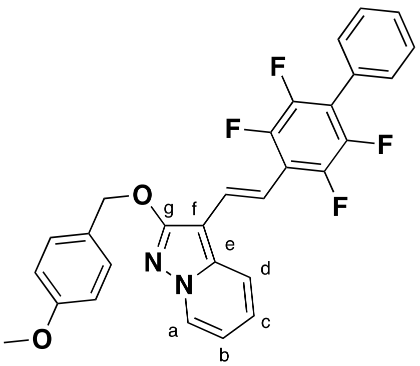

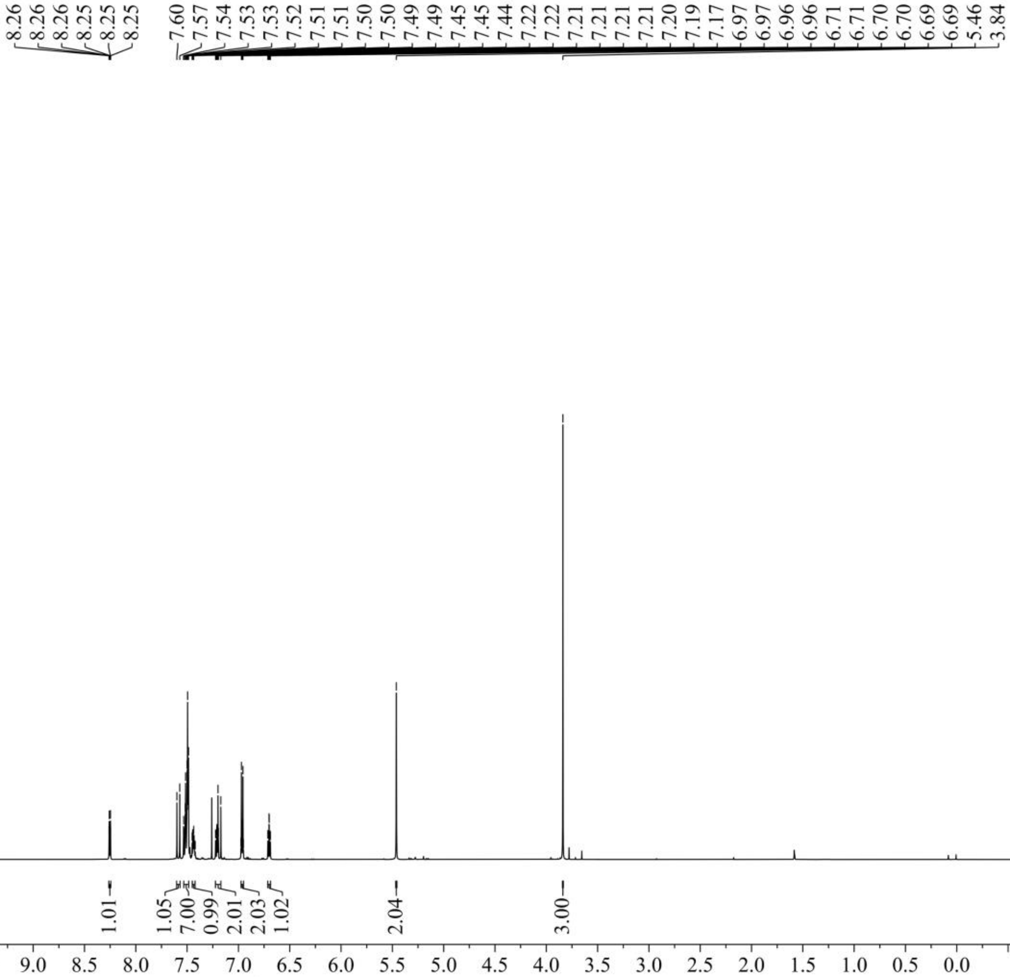

^{13}C NMR Spectrum ($CDCl_3$)

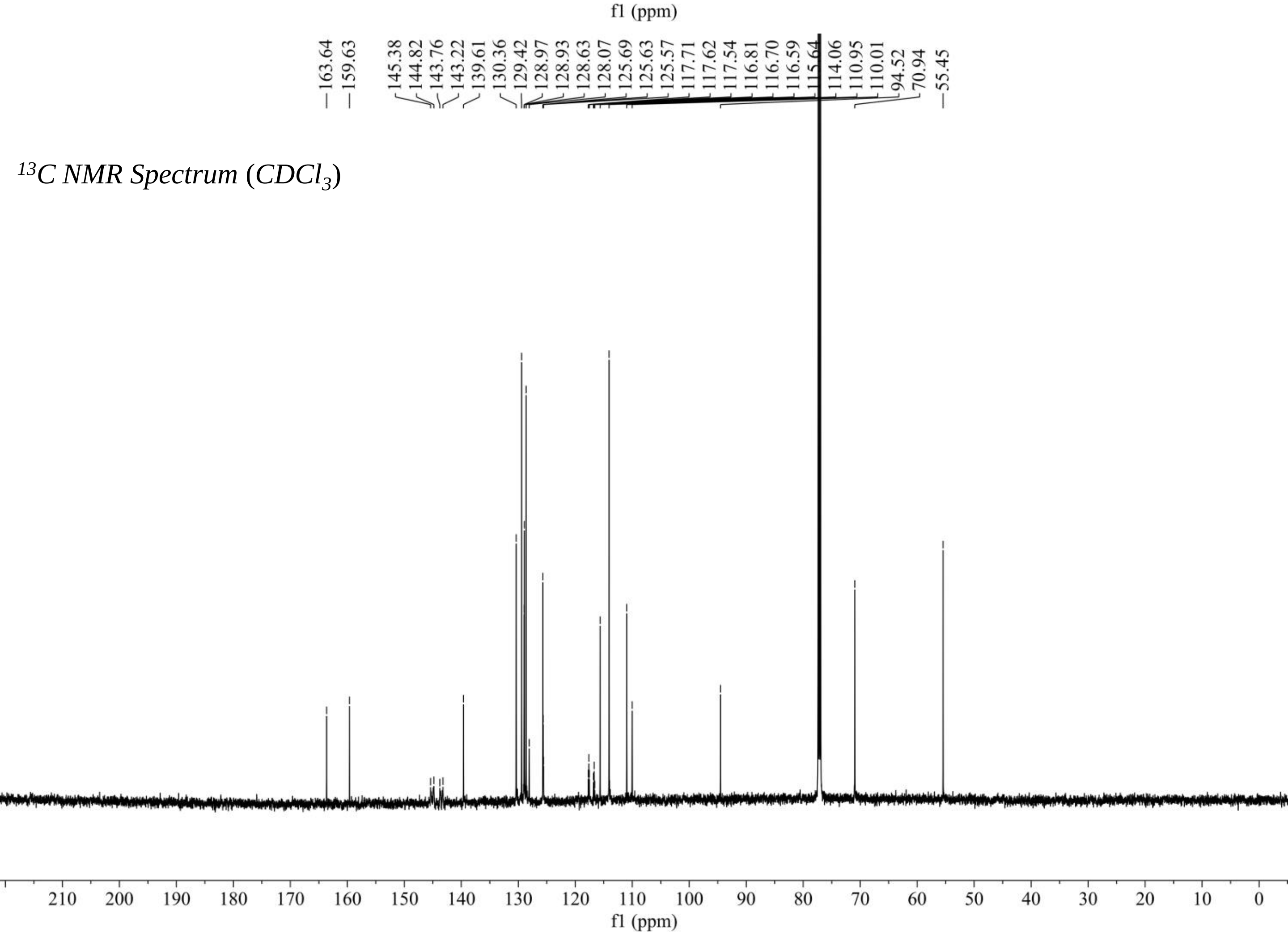

E)-2-(Benzyloxy)-3-(2-(2,3,5,6-tetrafluoro-[1,1'-biphenyl]-4-yl)vinyl)pyrazolo[1,5-a]pyridine (**28a**)

^{19}F NMR Spectrum ($CDCl_3$)

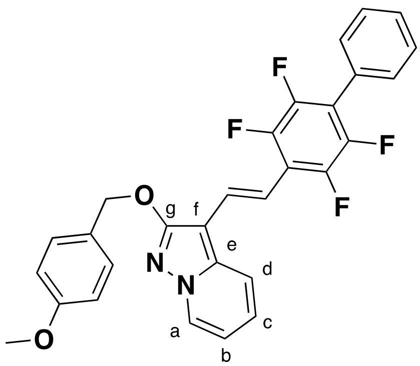

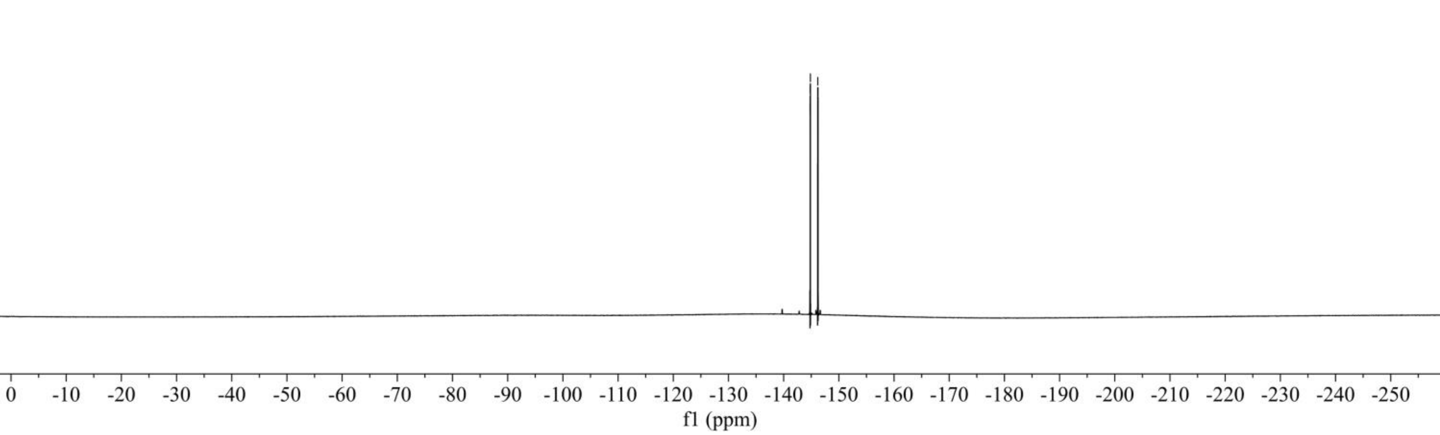

^{13}C NMR Spectrum ($CDCl_3$)

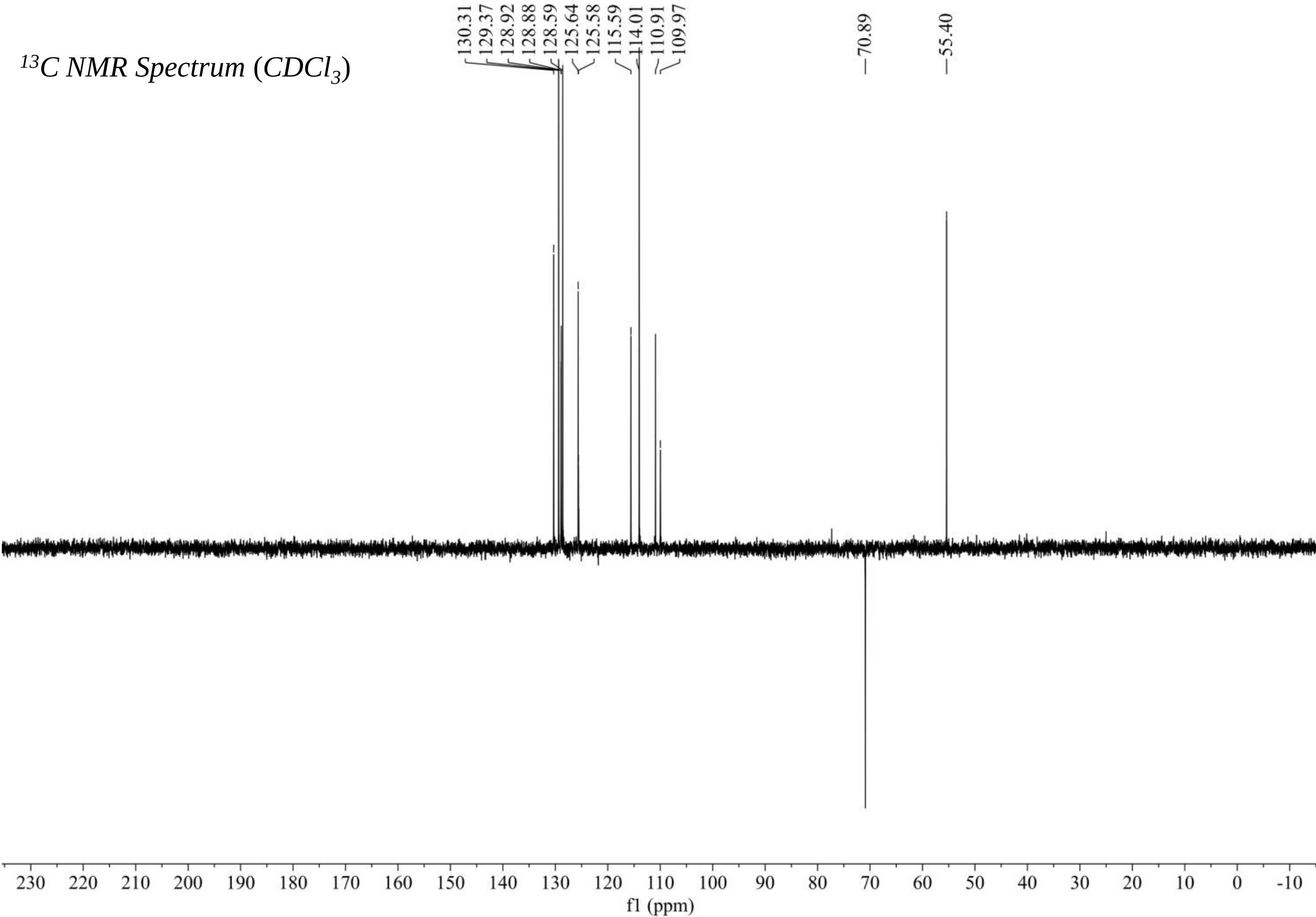

(E)-3-(2-(2,3,5,6-Tetrafluoro-[1,1'-biphenyl]-4-yl)vinyl)pyrazolo[1,5-a]pyridin-2-ol (**11a**)

^{1}H NMR Spectrum (DMSO)

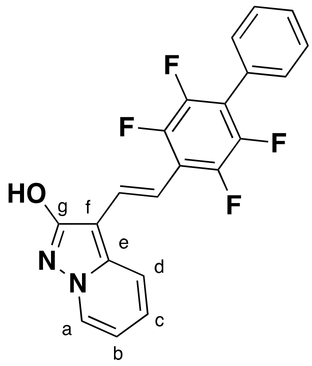

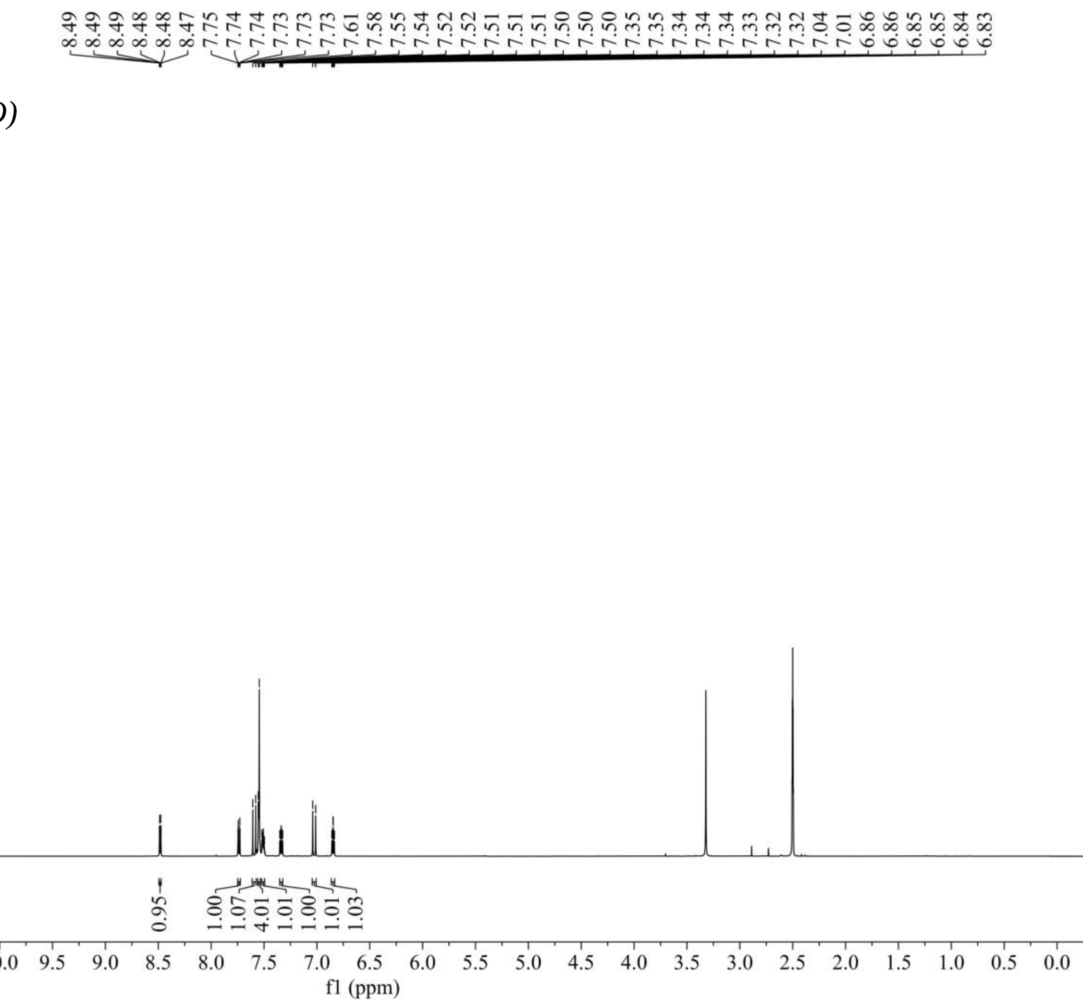

^{13}C NMR Spectrum (DMSO)

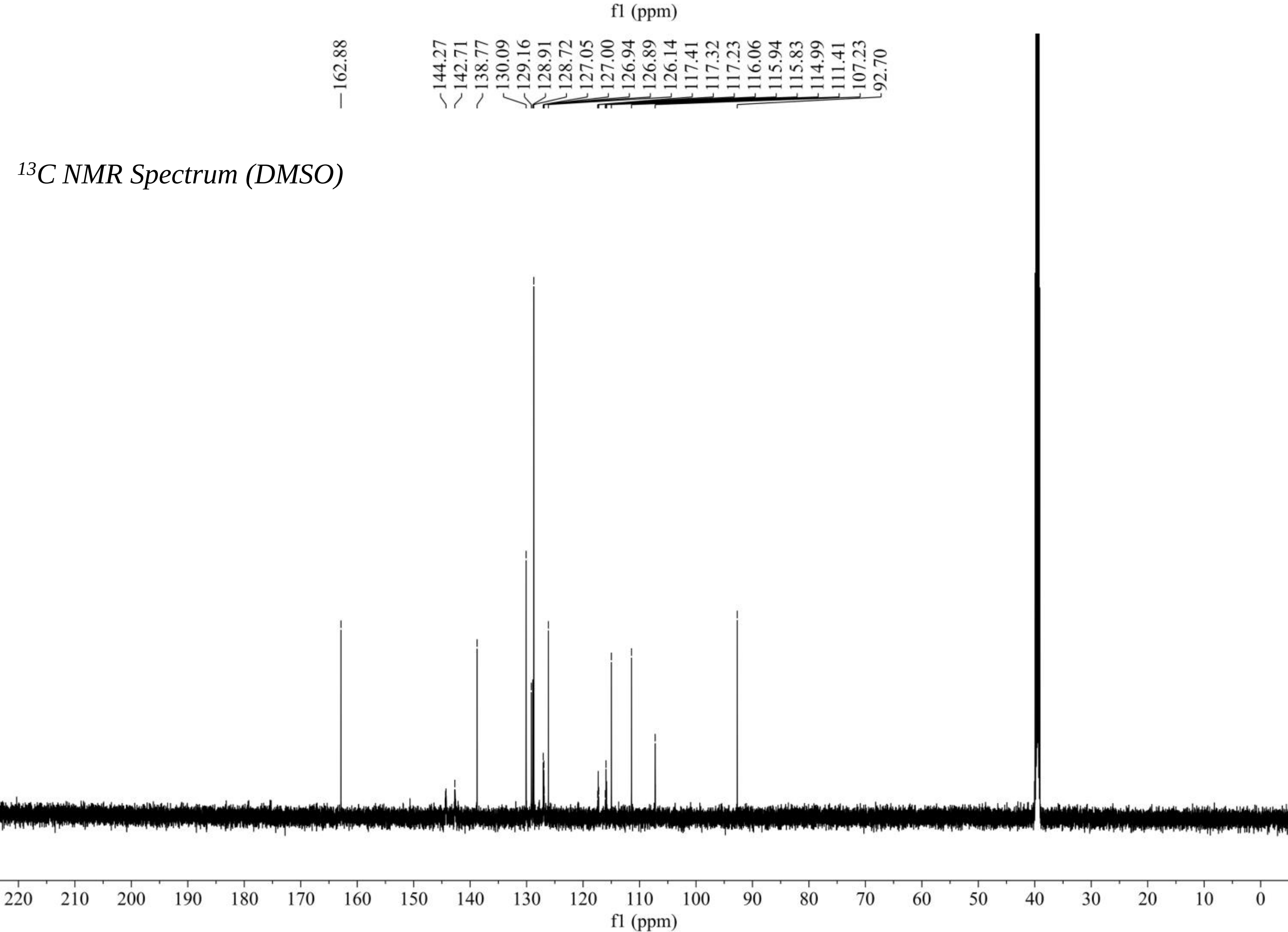

(E)-3-(2-(2,3,5,6-Tetrafluoro-[1,1'-biphenyl]-4-yl)vinyl)pyrazolo[1,5-a]pyridin-2-ol (**11a**)

^{19}F NMR Spectrum (DMSO)

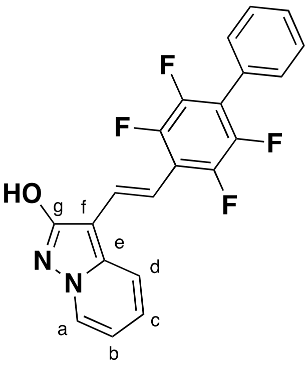

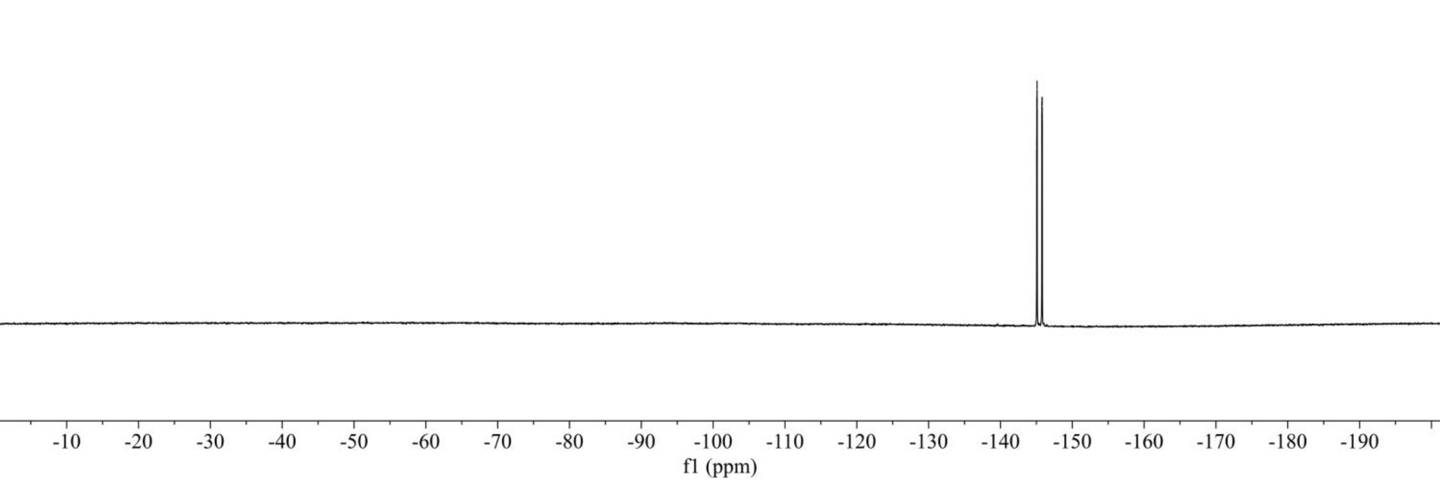

DEPT NMR Spectrum (DMSO)

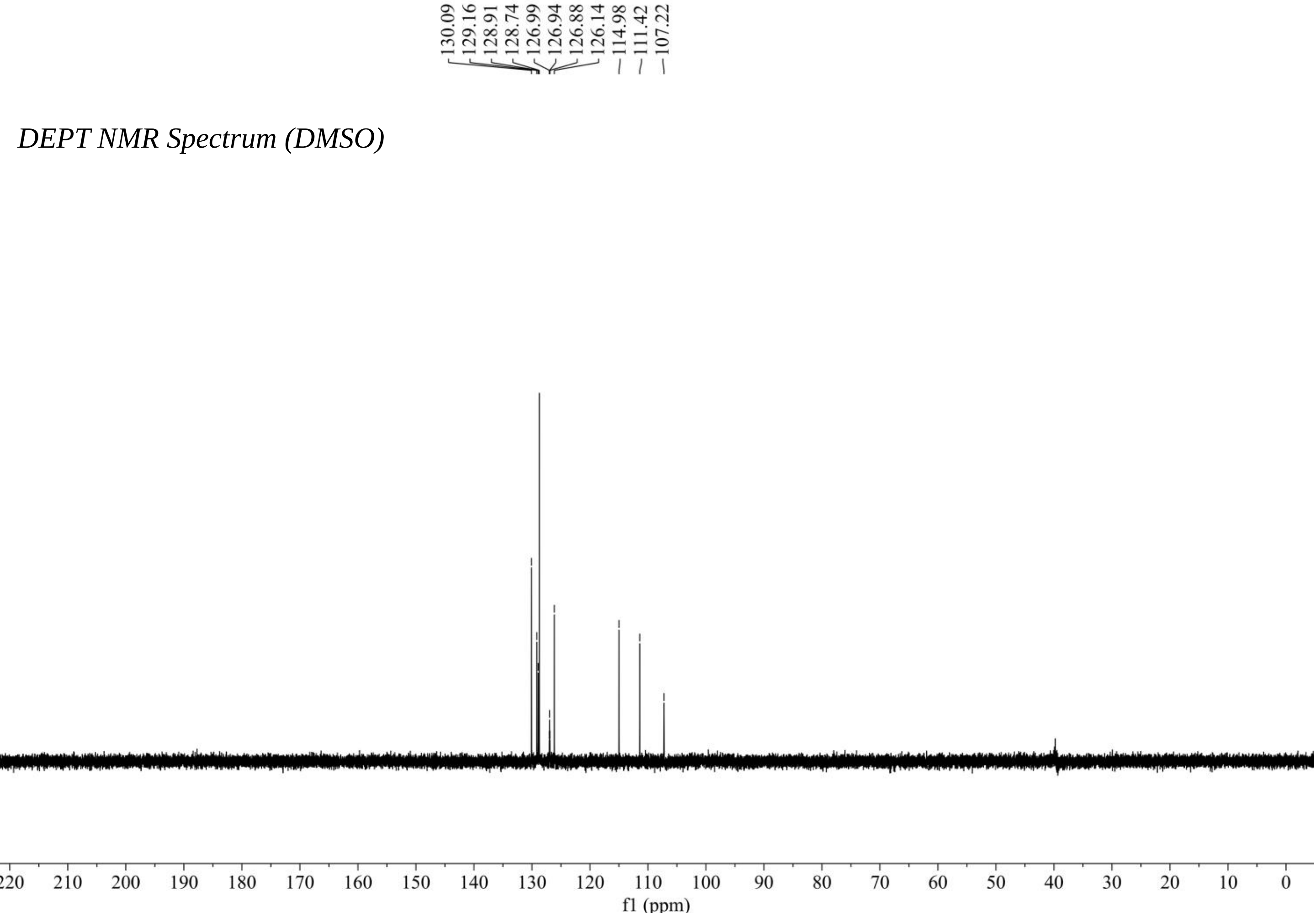

2-(Benzyloxy)-3-iodopyrazolo[1,5-a]pyridine (**29a**)

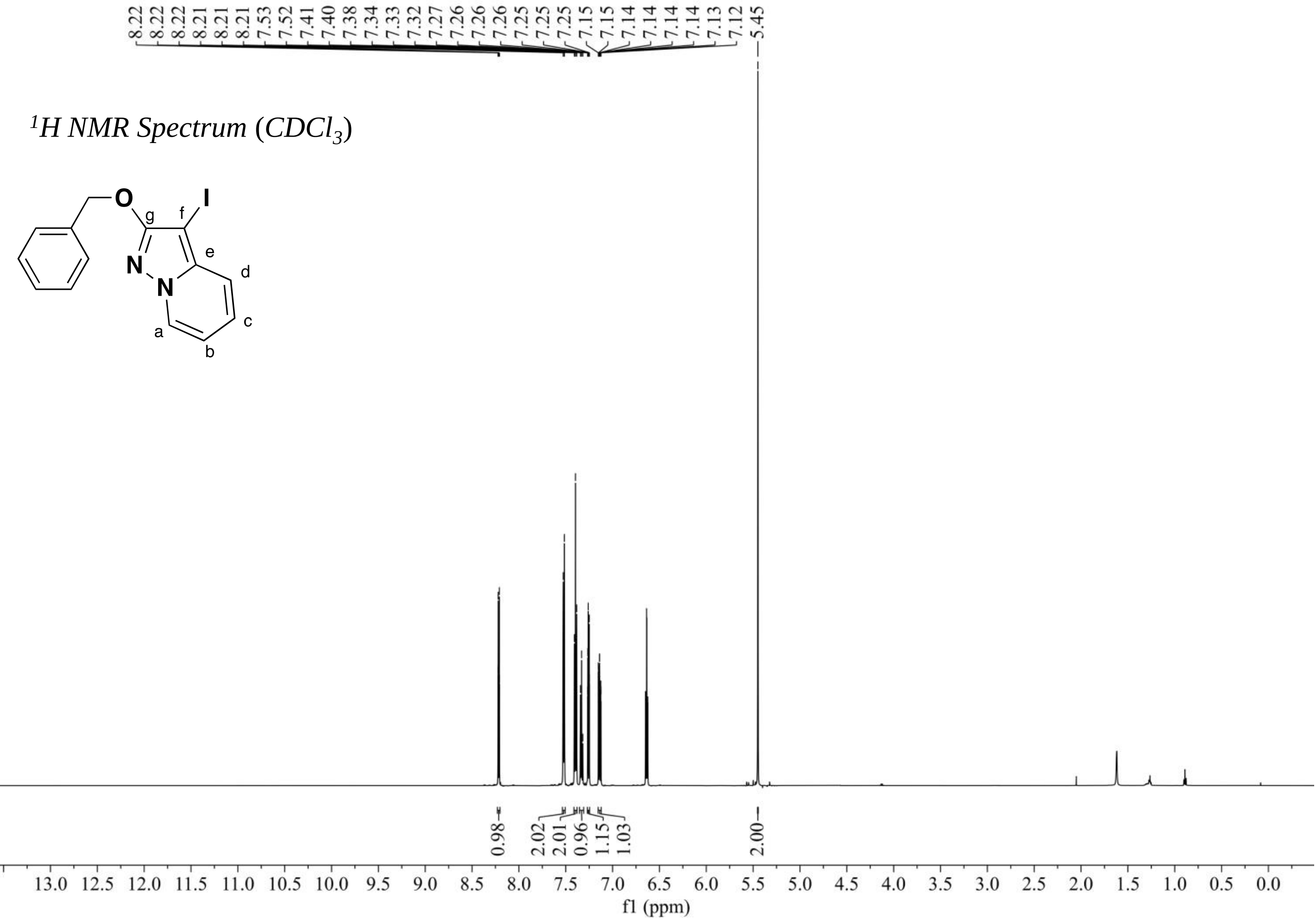

^{1}H NMR Spectrum (*CDCl$_3$*)

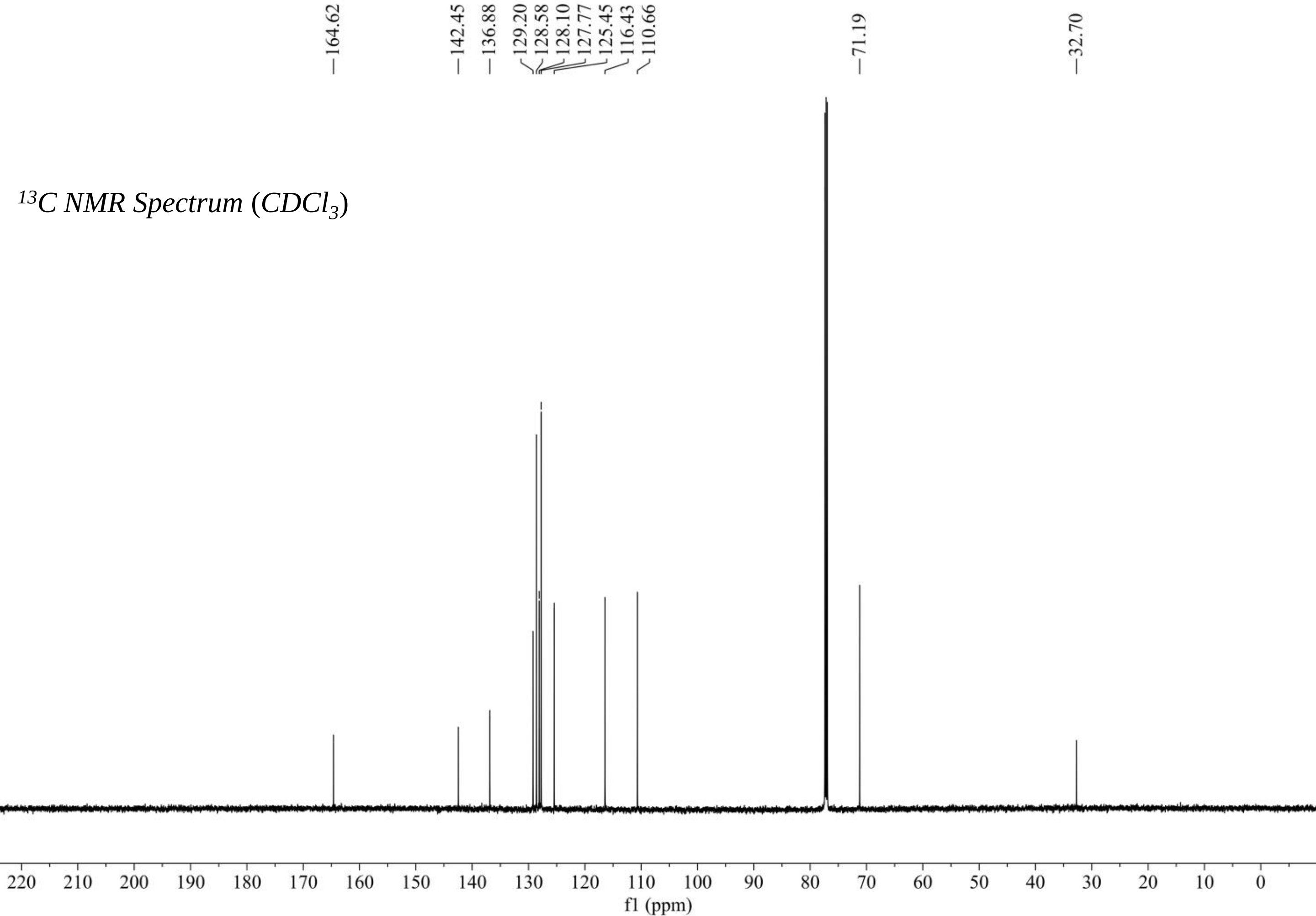

^{13}C NMR Spectrum (*CDCl$_3$*)

3-iodo-2-methoxypyrazolo[1,5-a]pyridine (**29b**)

^{1}H NMR Spectrum ($CDCl_3$)

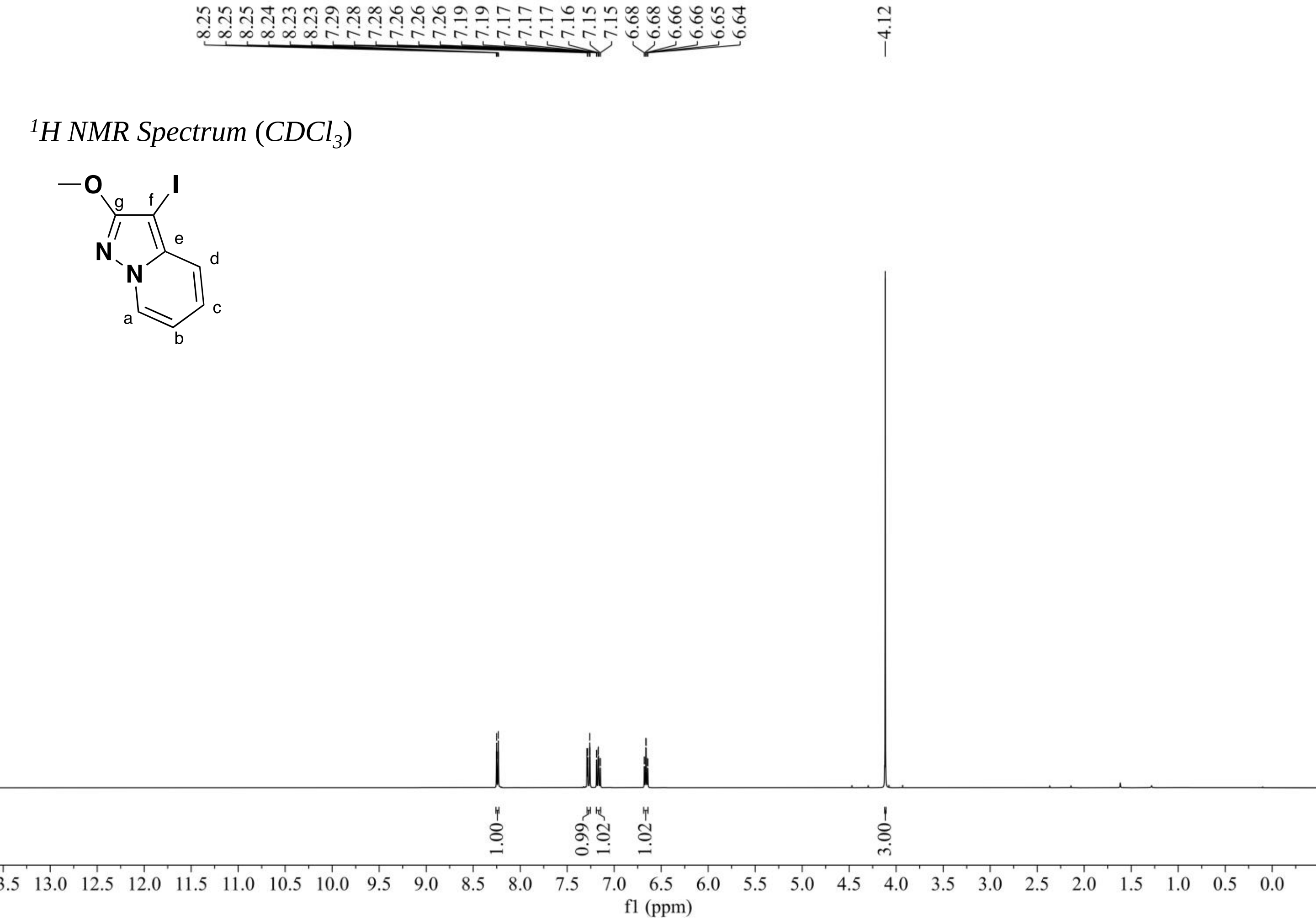

^{13}C NMR Spectrum ($CDCl_3$)

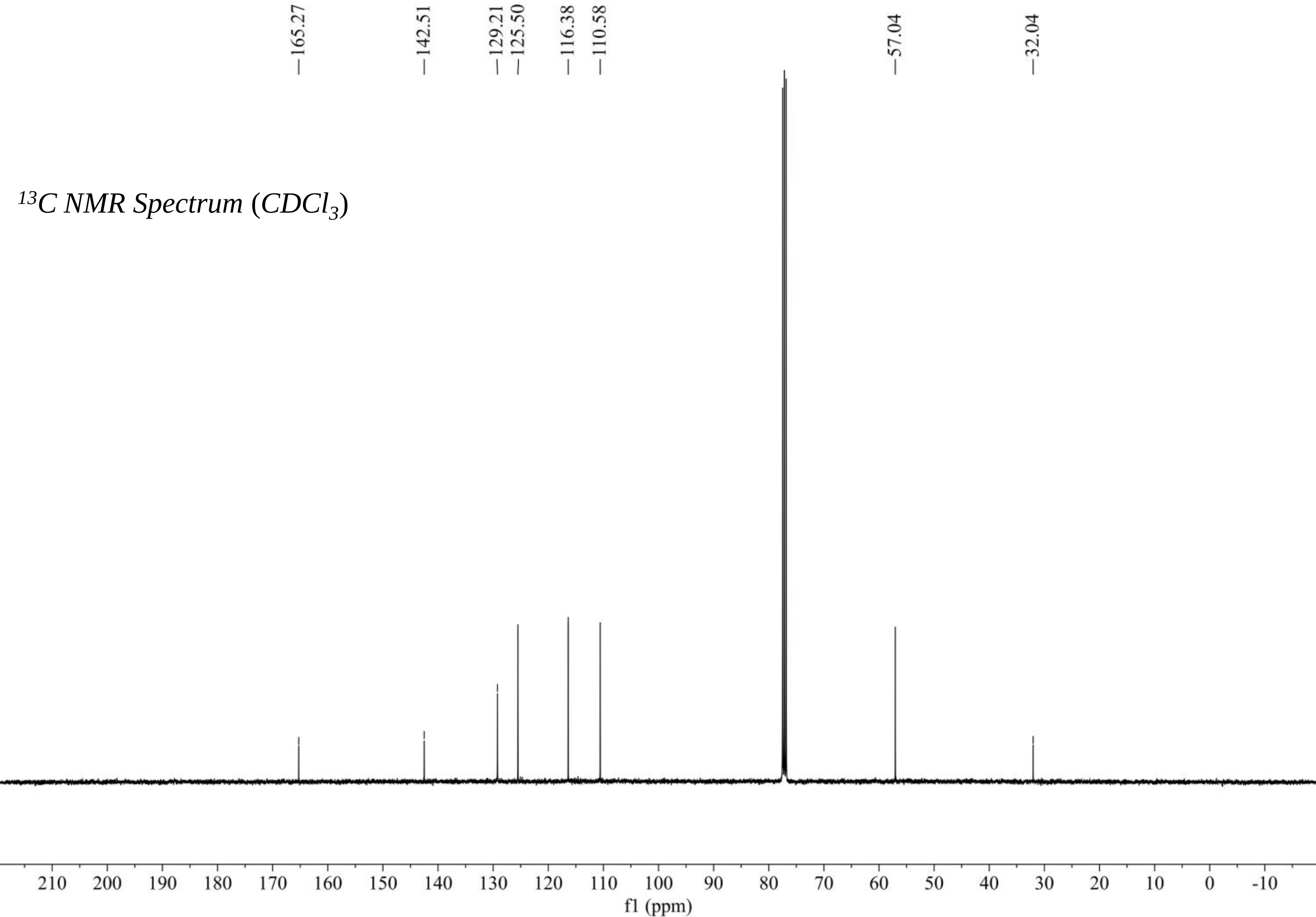

3-iodo-2-methoxypyrazolo[1,5-a]pyridine (**29b**)

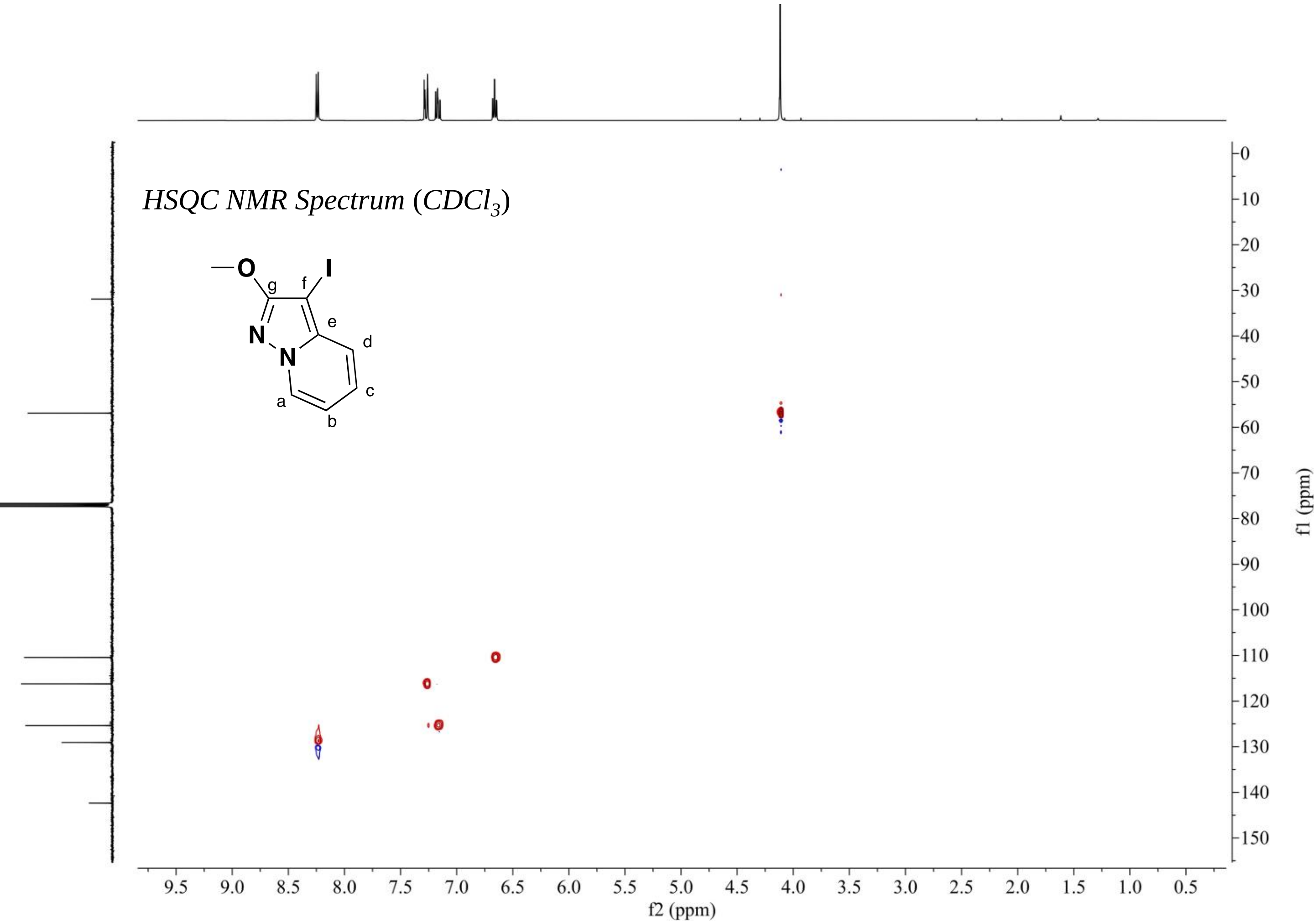

6-phenylquinolin-2-ol (**42**)

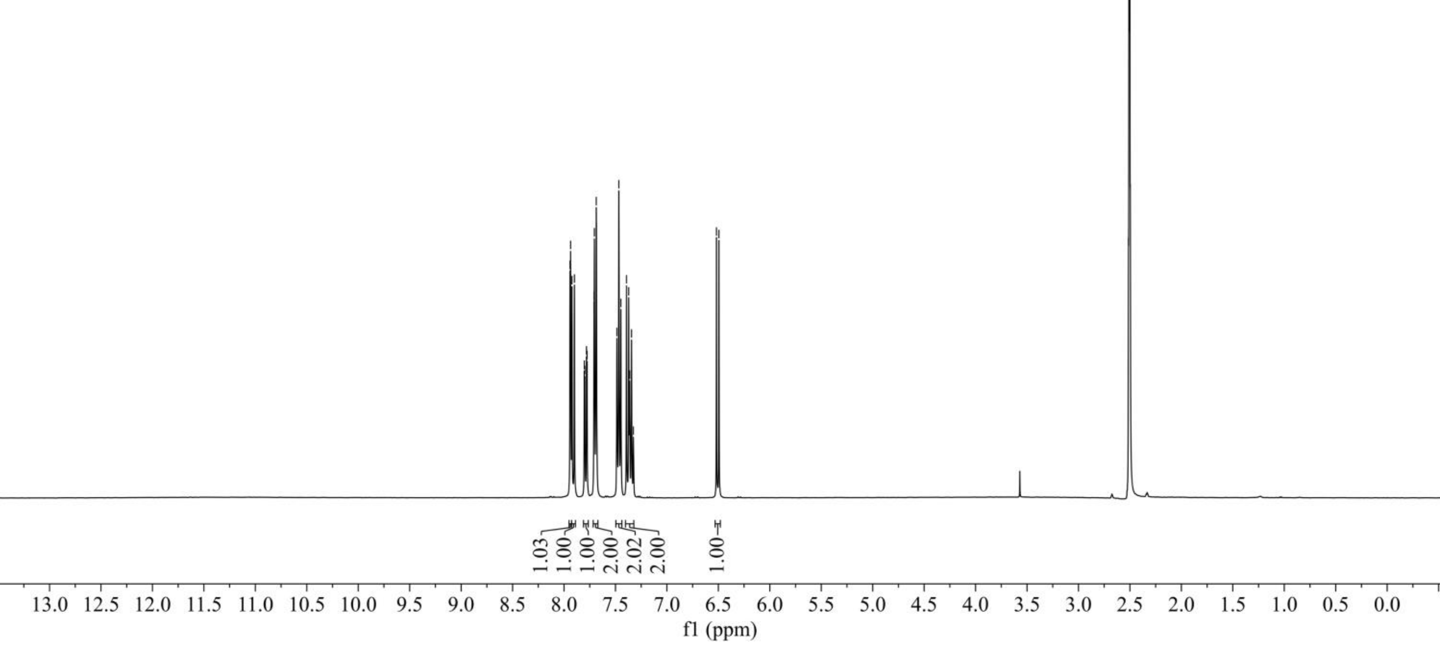

^{1}H NMR Spectrum (*DMSO*)

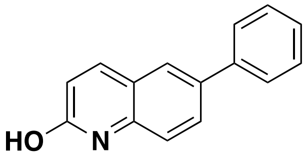

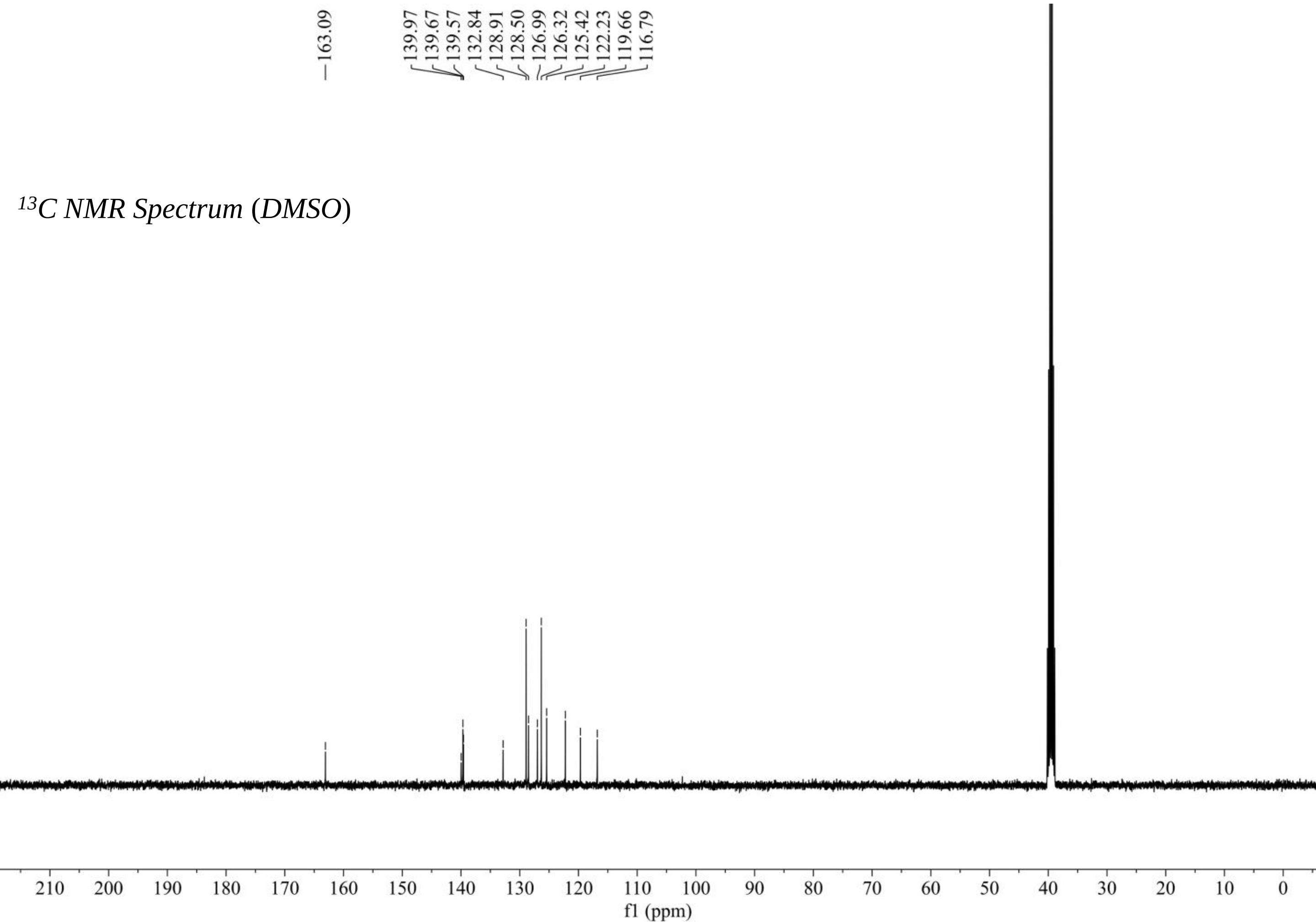

^{13}C NMR Spectrum (*DMSO*)

6-phenylquinolin-2-ol (**42**)

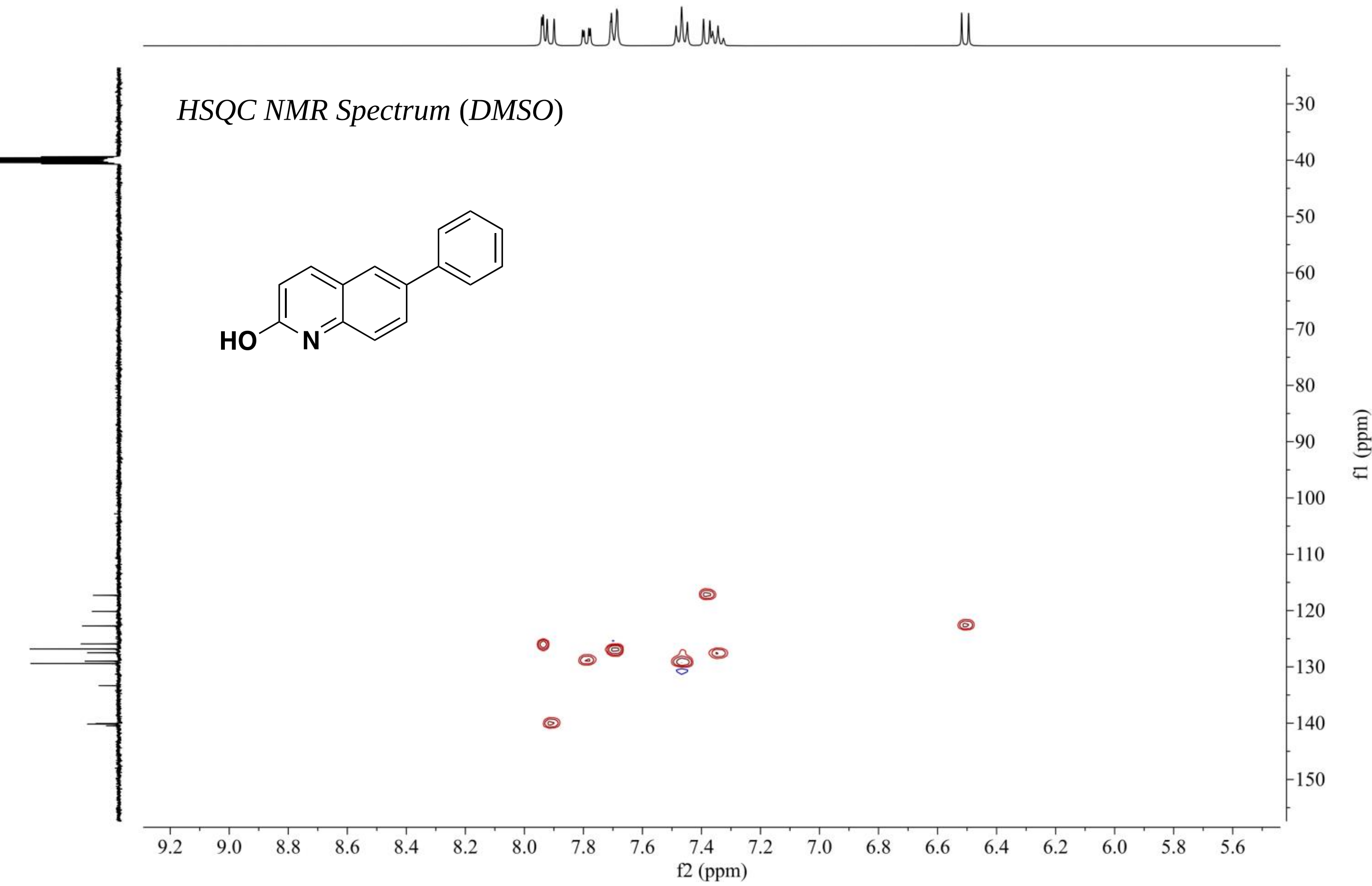

2-chloro-6-phenylquinoline (**43**)

^{1}H NMR Spectrum (*$CDCl_3$*)

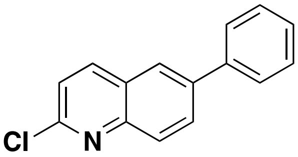

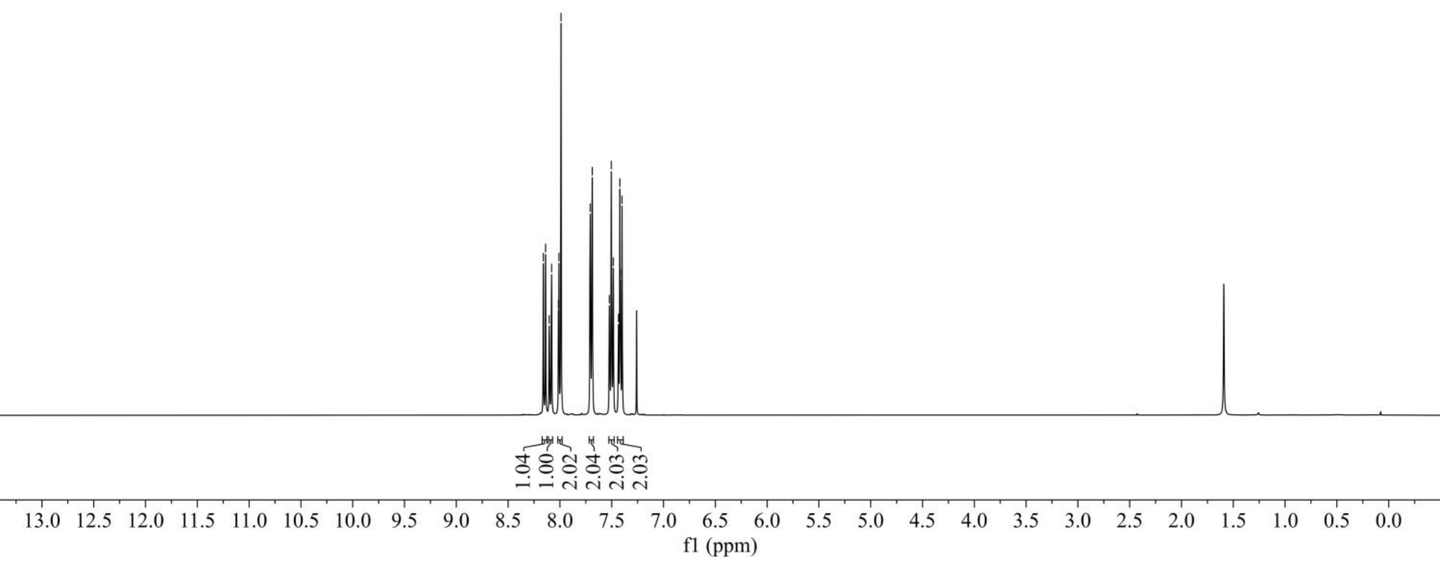

^{13}C NMR Spectrum (*$CDCl_3$*)

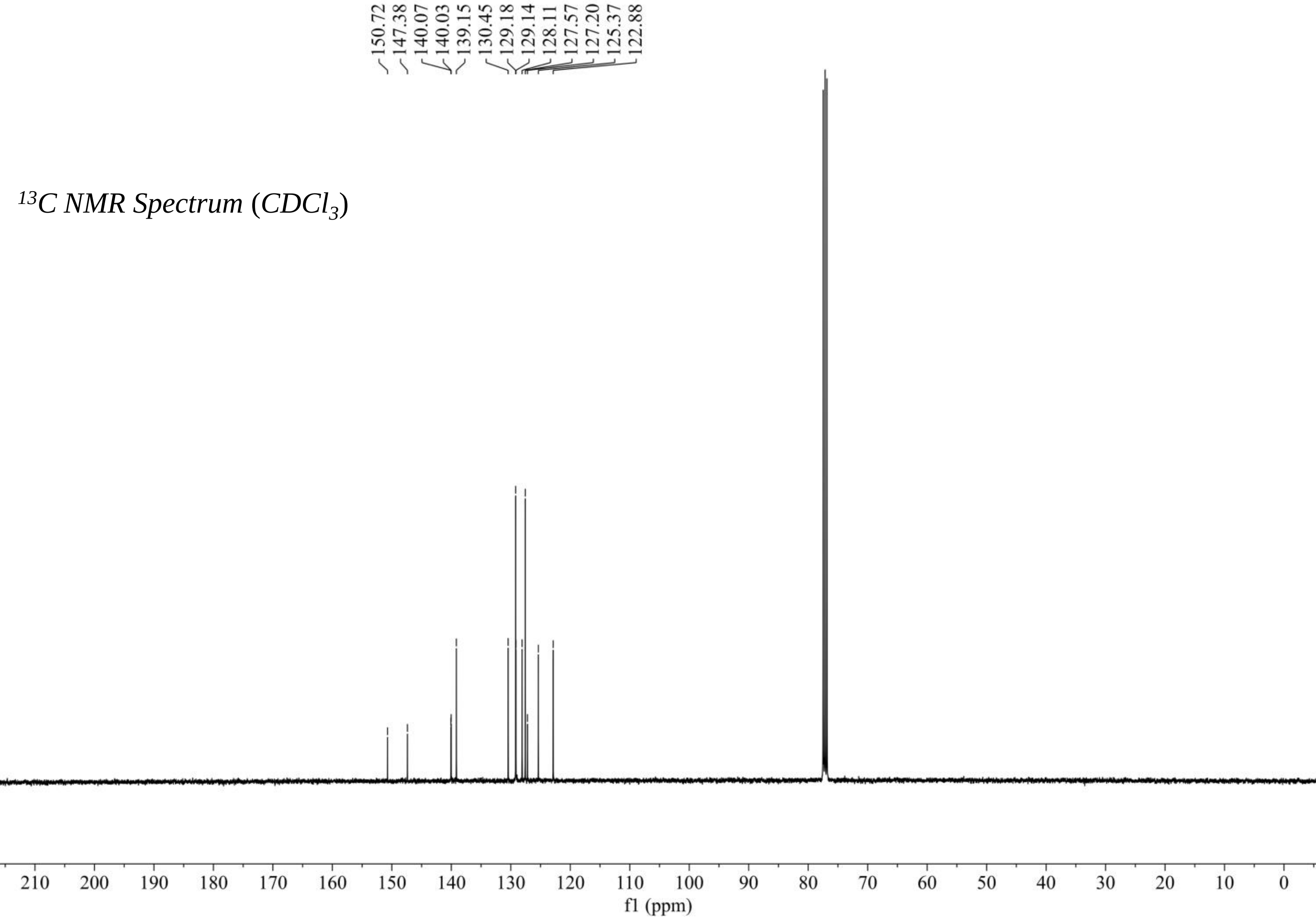

2-chloro-6-phenylquinoline (**43**)

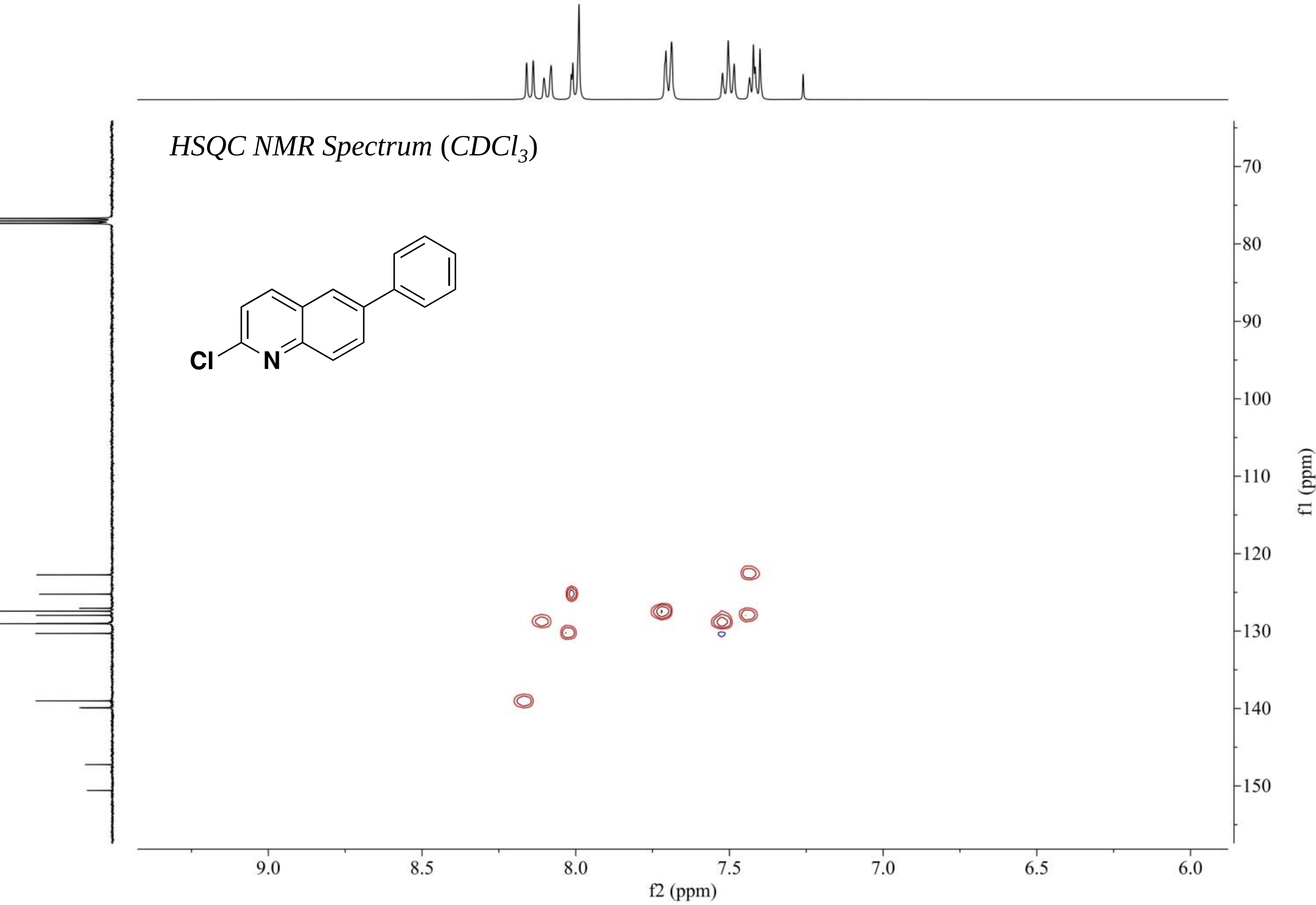

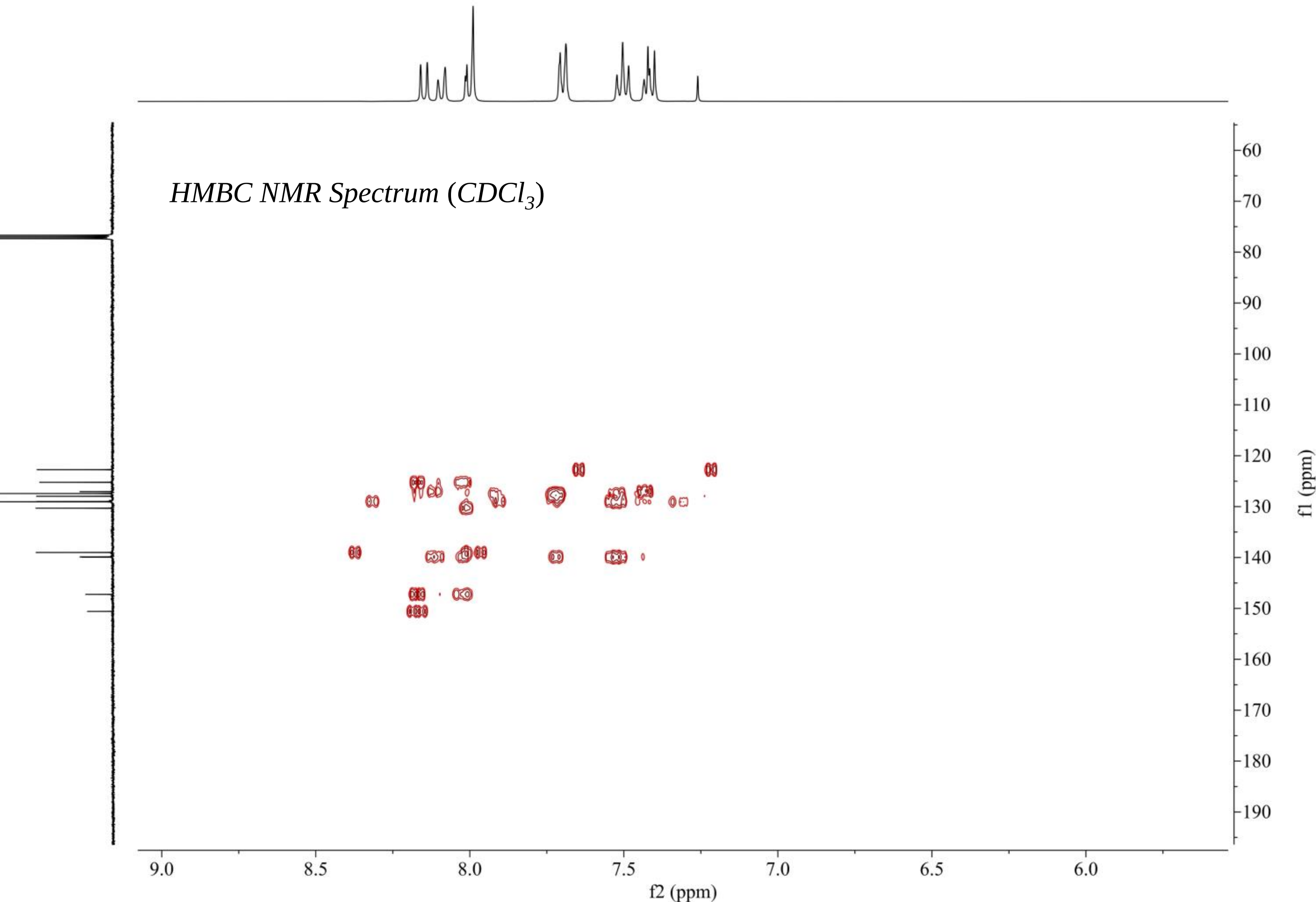

2-(2-methoxypyrazolo[1,5-a]pyridin-3-yl)-6-phenylquinoline (**31**)

^{1}H NMR Spectrum ($CDCl_3$)

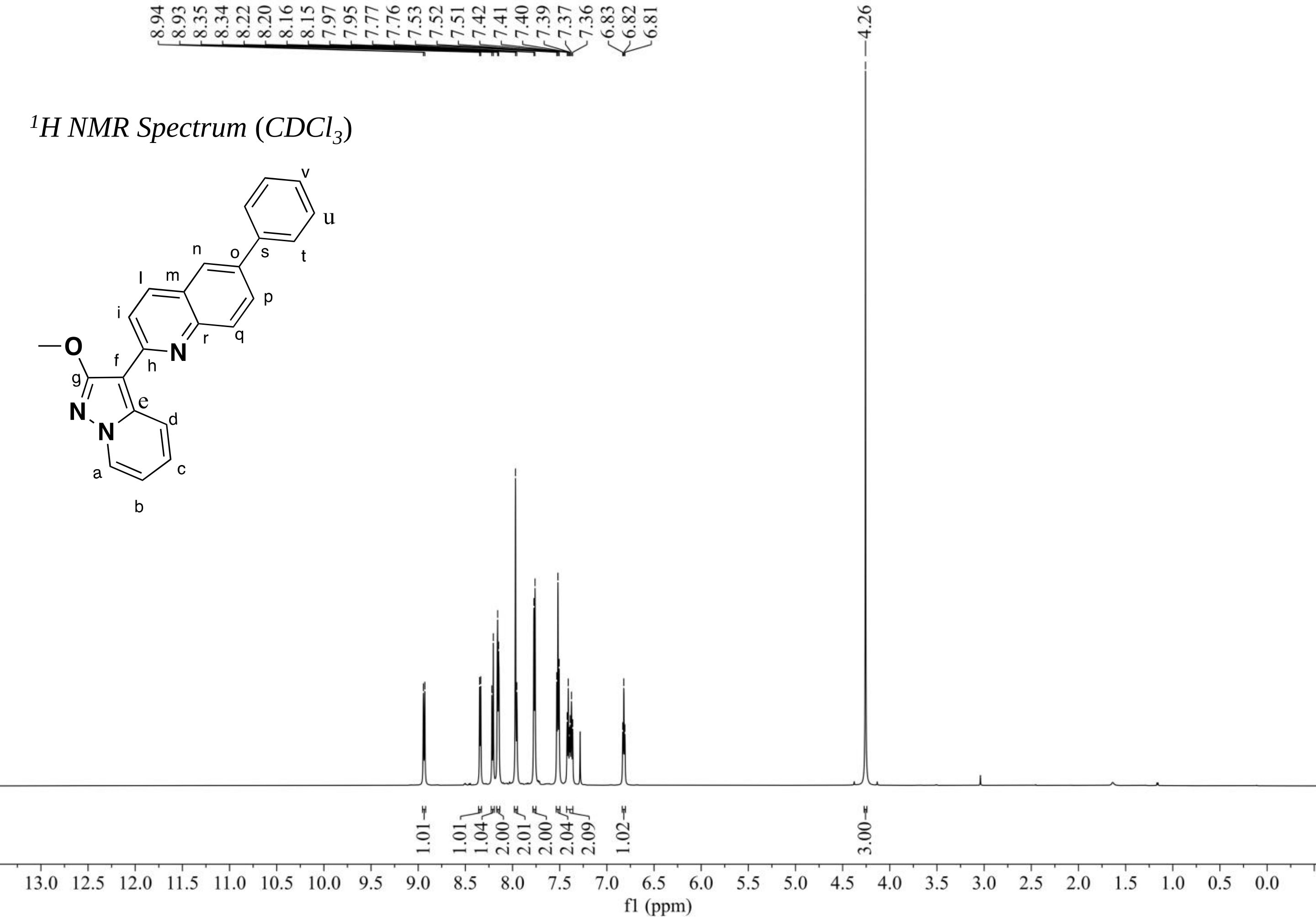

^{13}C NMR Spectrum ($CDCl_3$)

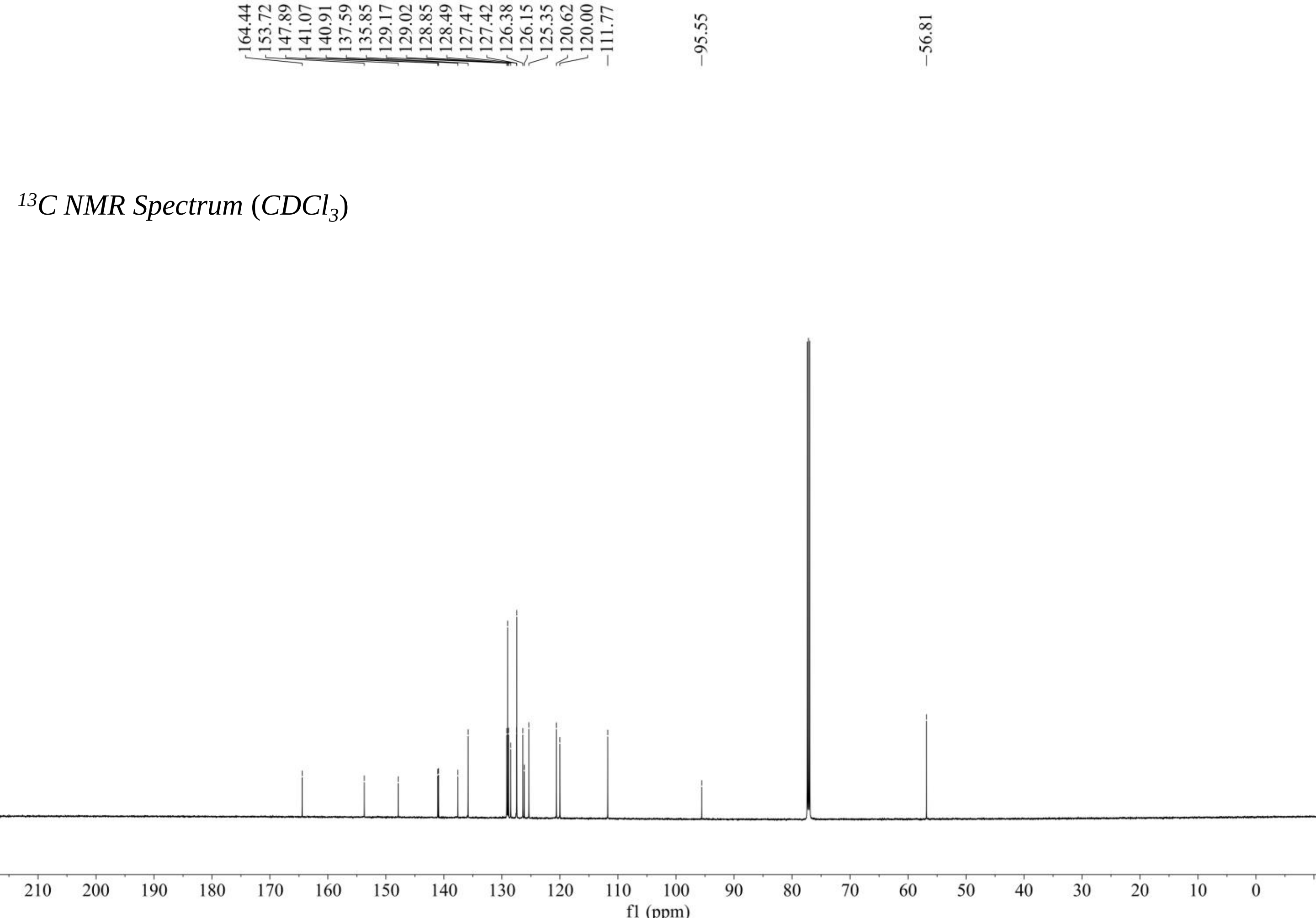

2-(2-methoxypyrazolo[1,5-a]pyridin-3-yl)-6-phenylquinoline (**31**)

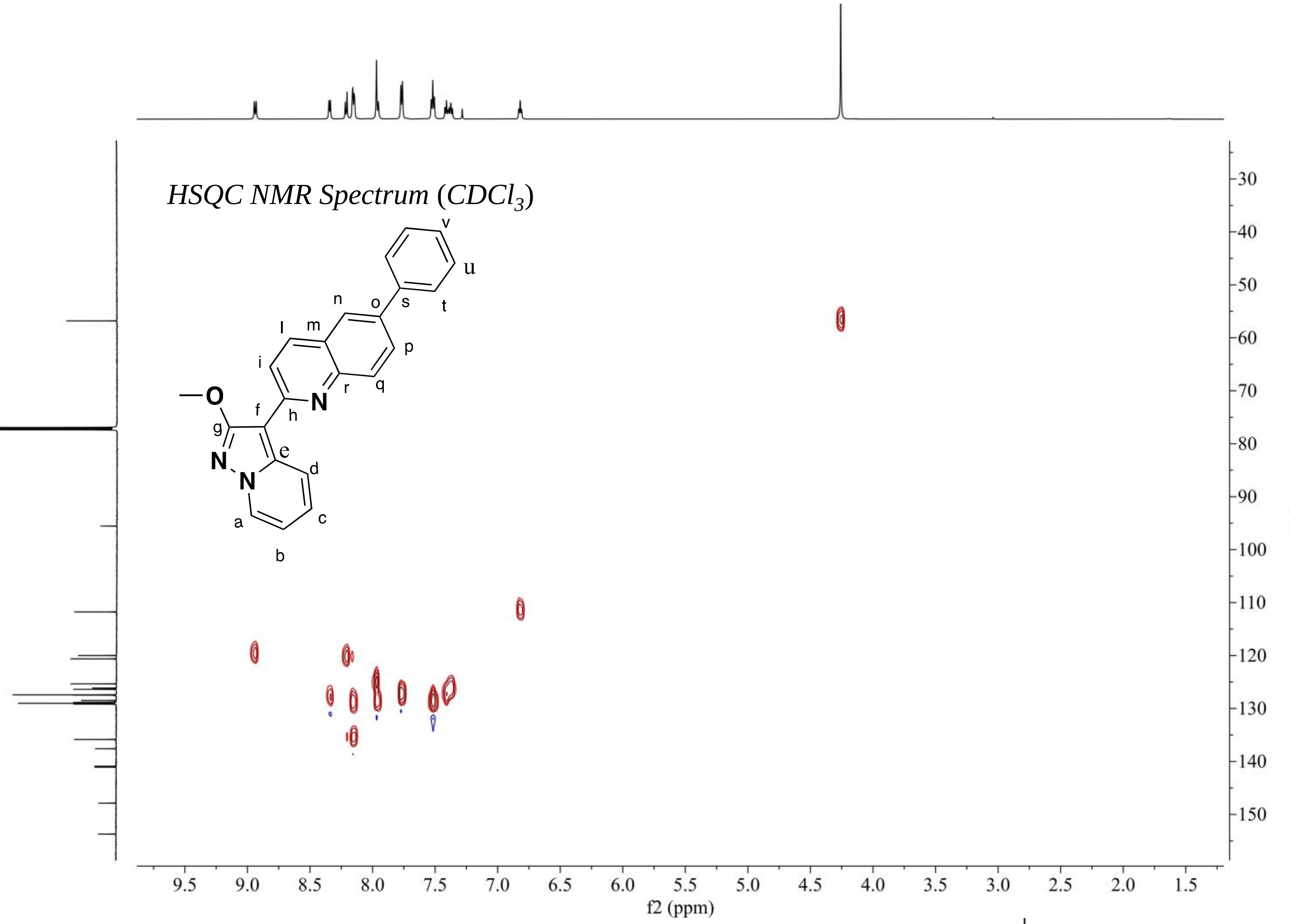

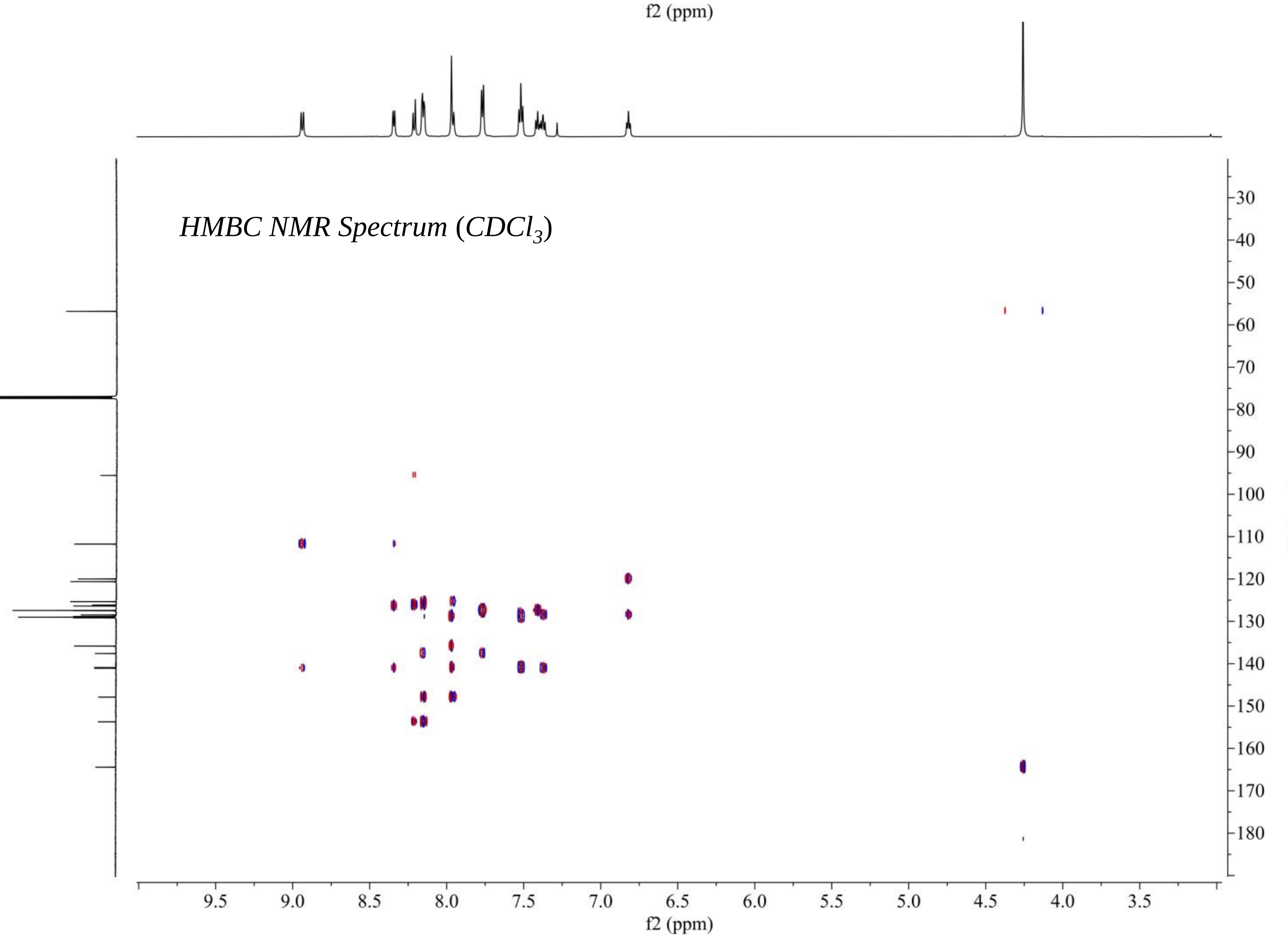

3-(6-phenylquinolin-2-yl)pyrazolo[1,5-a]pyridin-2-ol (**12**)

^{1}H NMR Spectrum (*DMSO*)

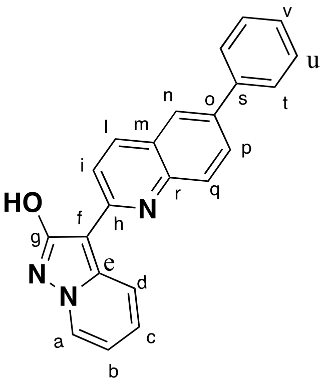

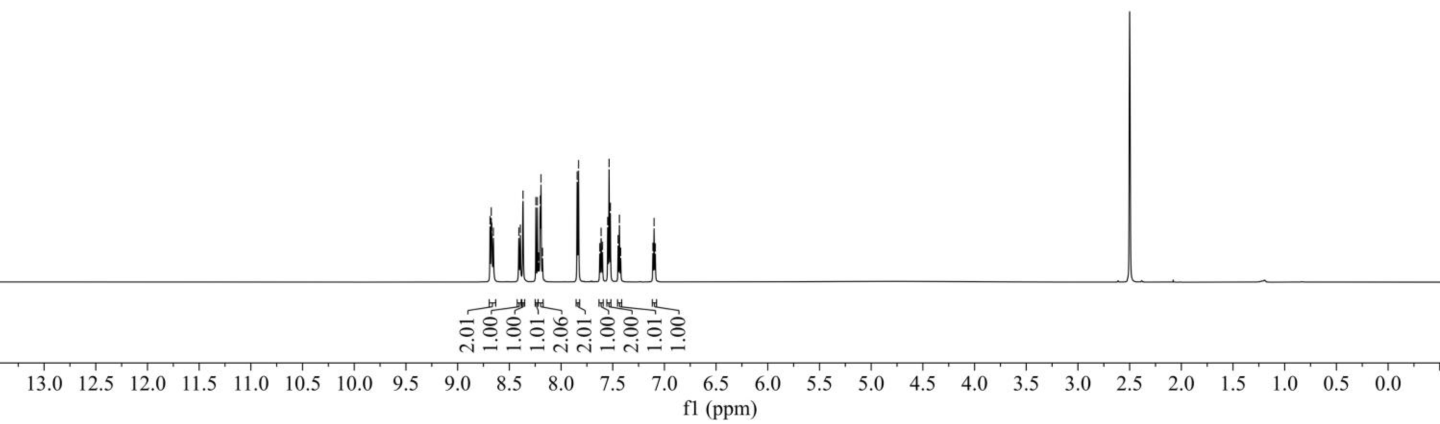

^{13}C NMR Spectrum (*DMSO*)

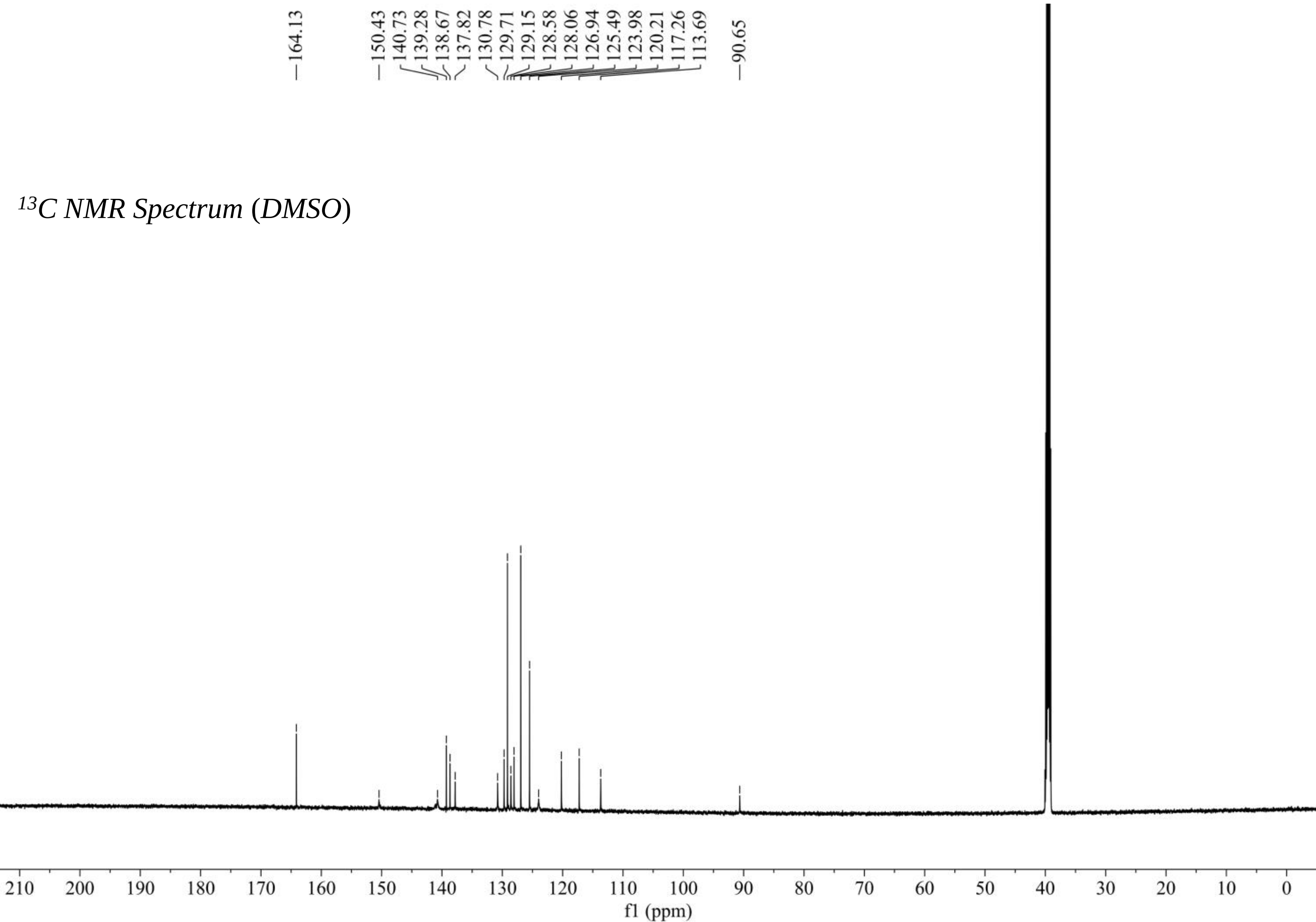

3-(6-phenylquinolin-2-yl)pyrazolo[1,5-a]pyridin-2-ol (**12**)

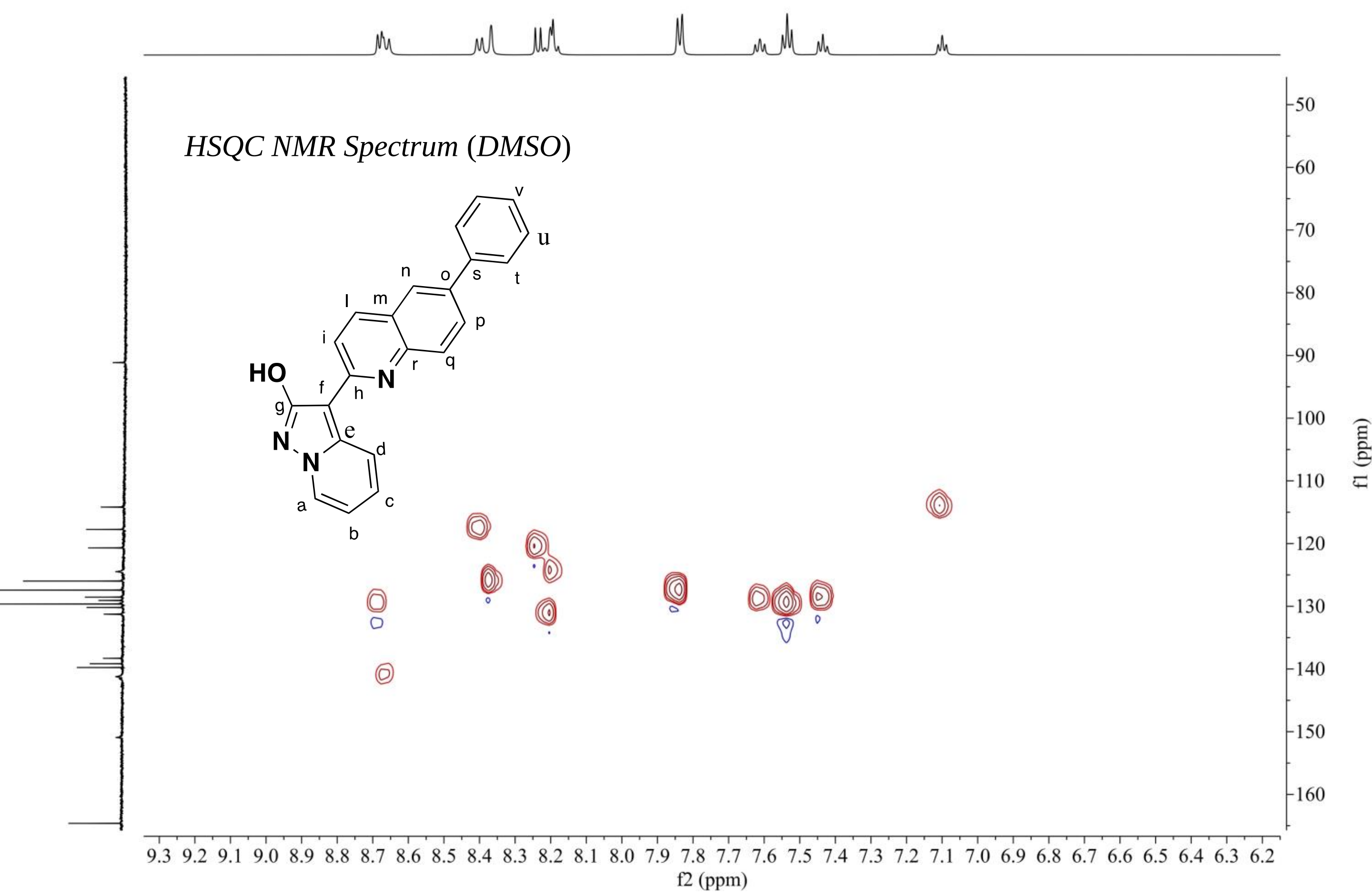

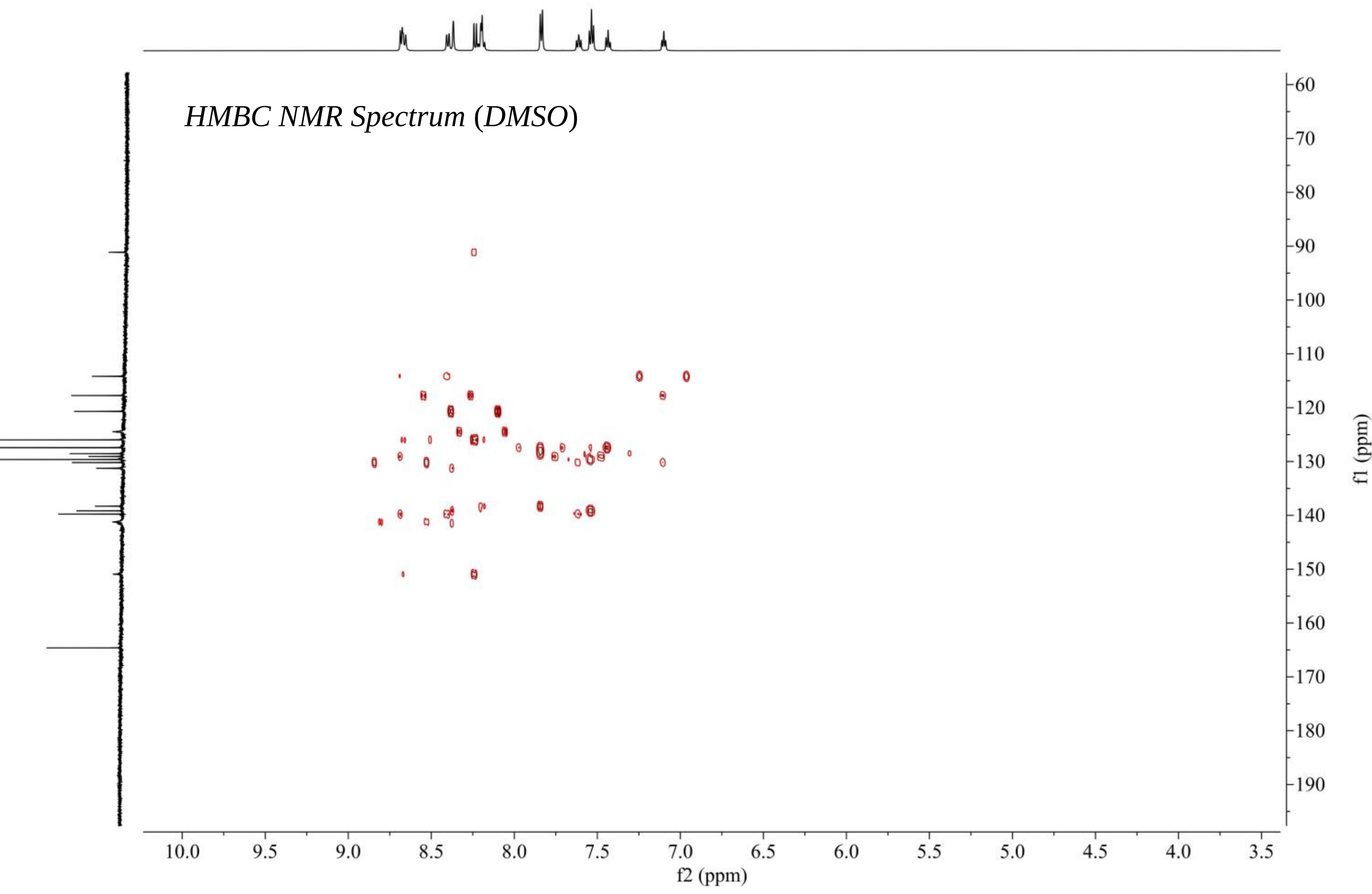

7-bromo-3-chloroquinoline (**44**)

^{1}H NMR Spectrum (*CDCl$_3$*)

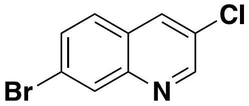

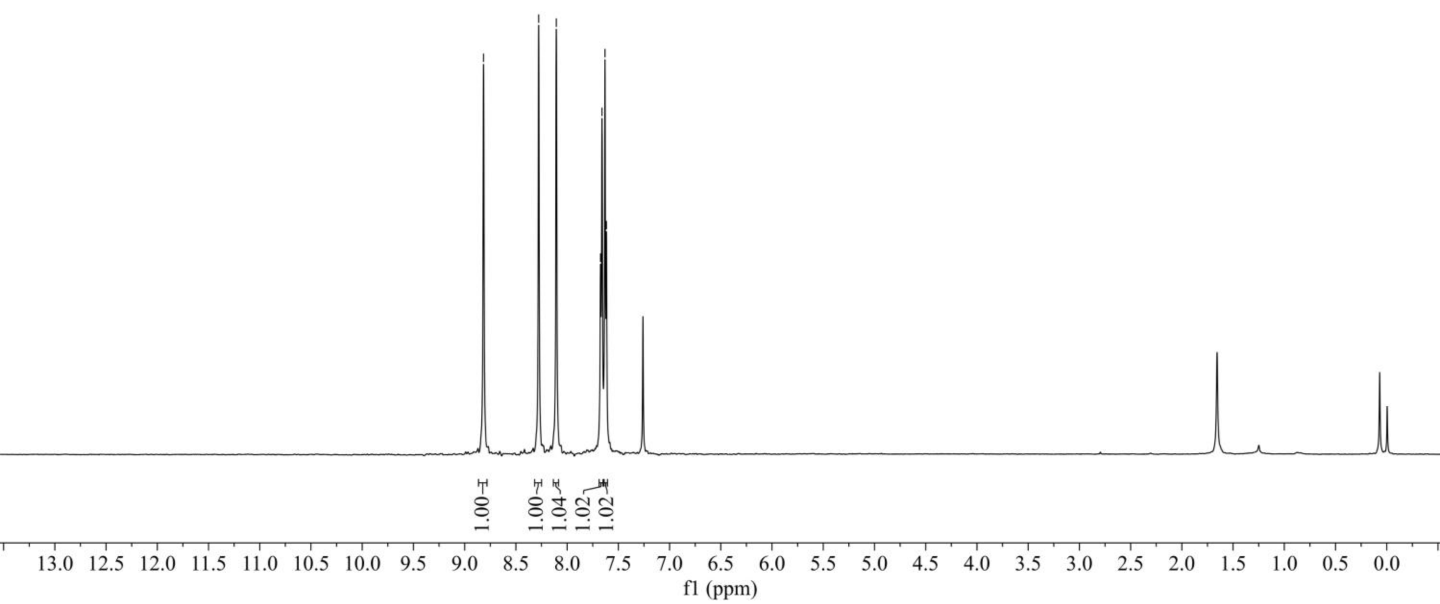

^{13}C NMR Spectrum (*CDCl$_3$*)

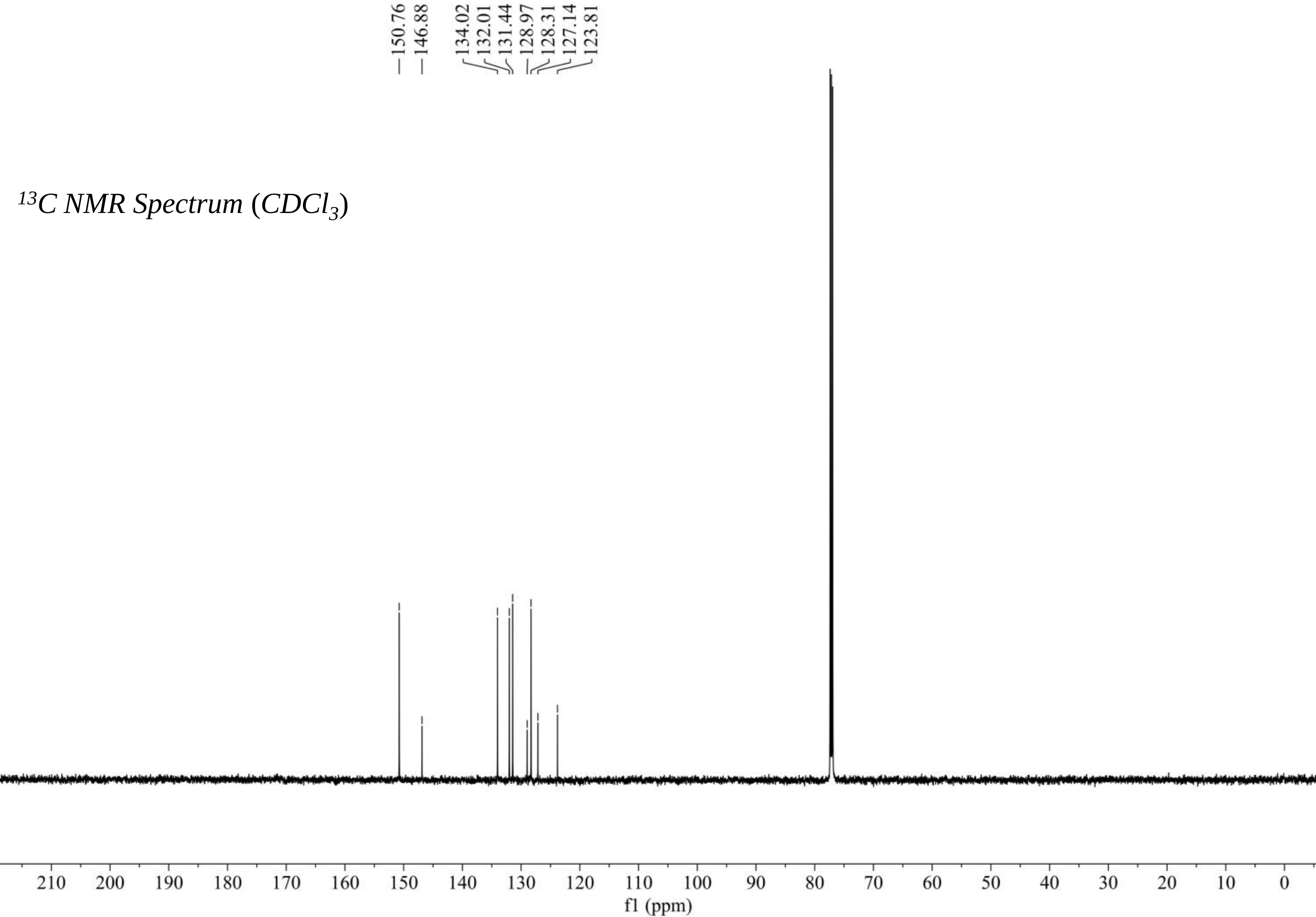

3-chloro-7-(2-methoxypyrazolo[1,5-a]pyridin-3-yl)quinoline (**32**)

^{1}H NMR Spectrum ($CDCl_3$)

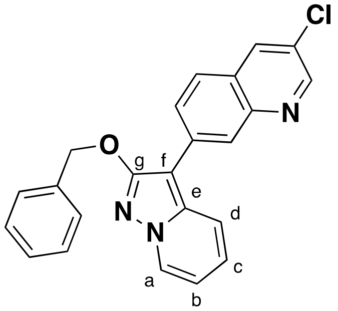

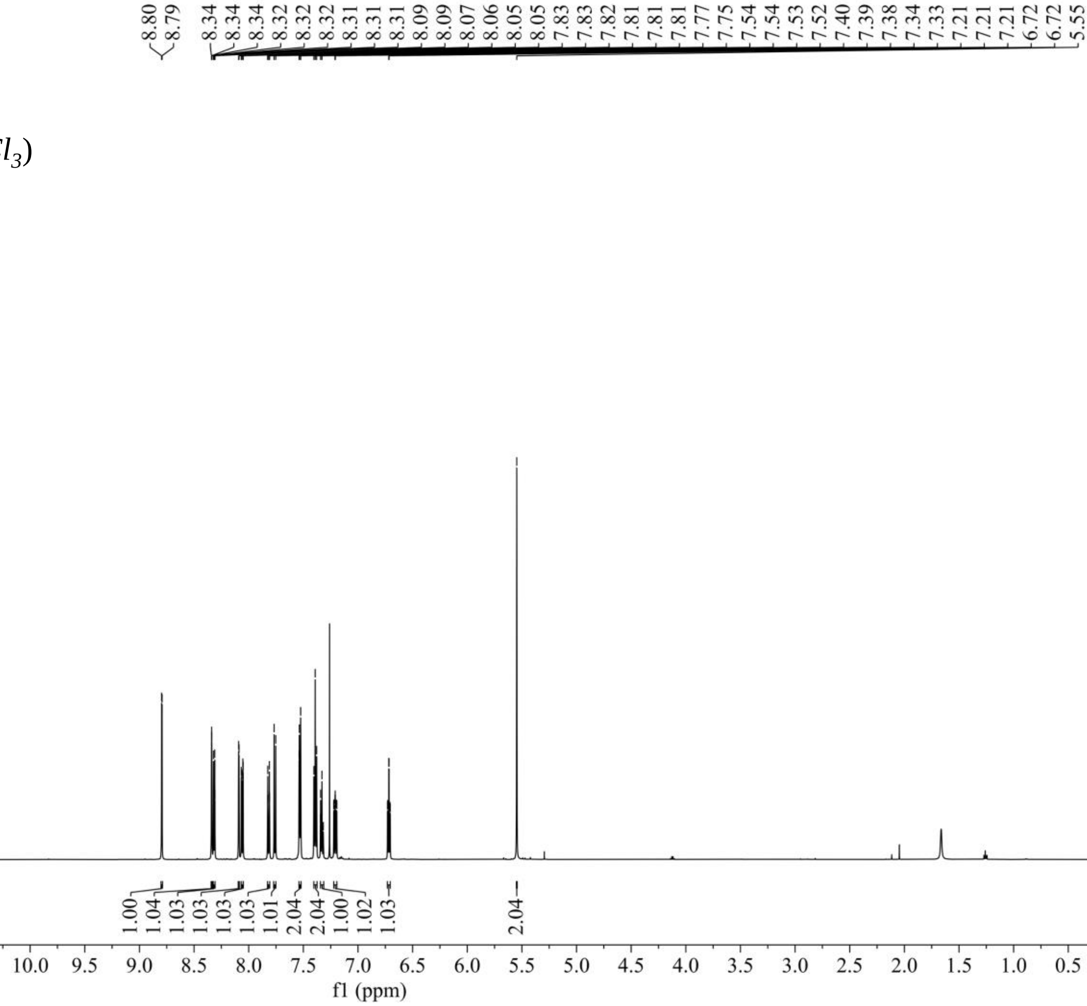

^{13}C NMR Spectrum ($CDCl_3$)

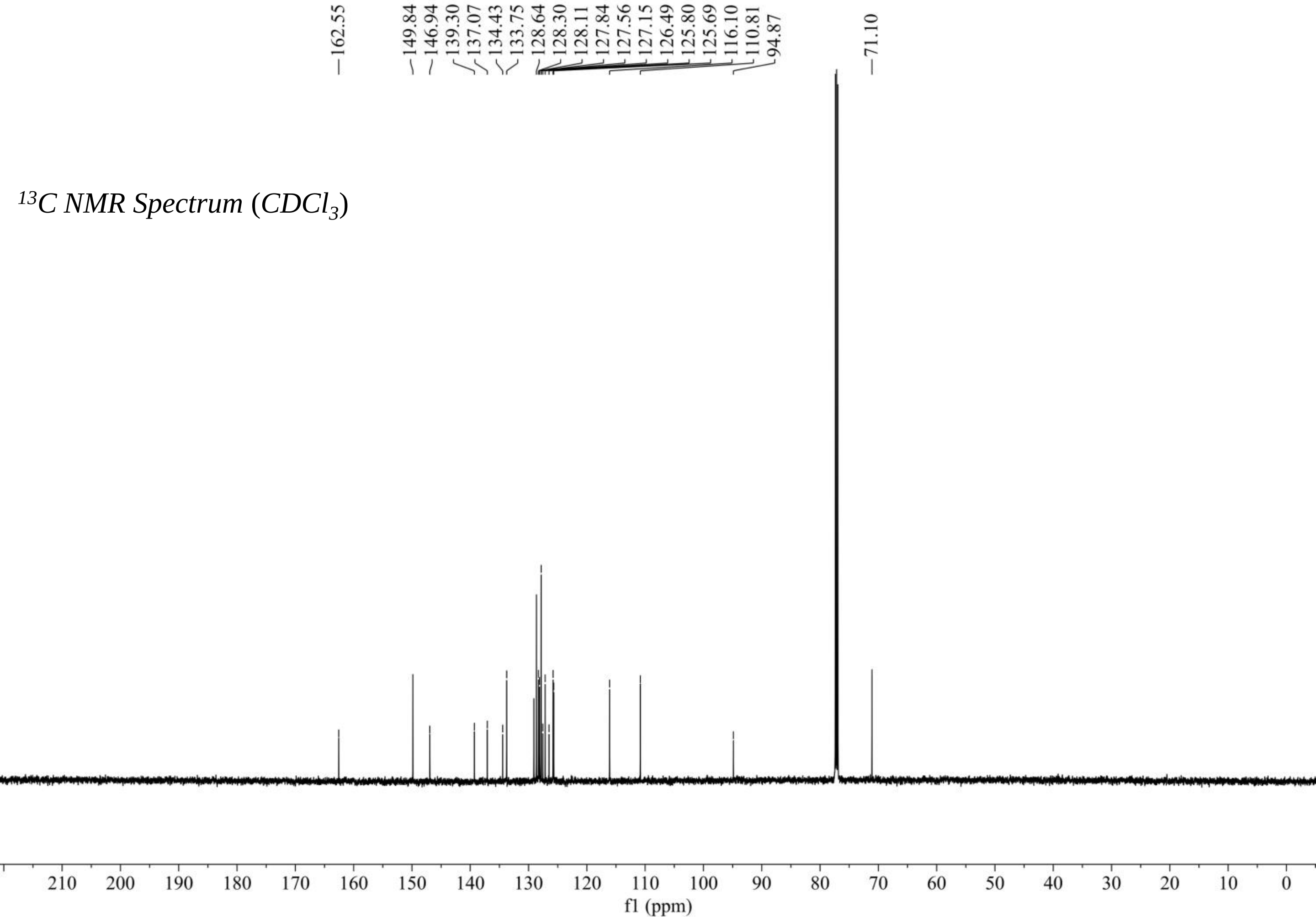

7-(2-(Benzyloxy)pyrazolo[1,5-a]pyridin-3-yl)-3-phenylquinoline (**33**)

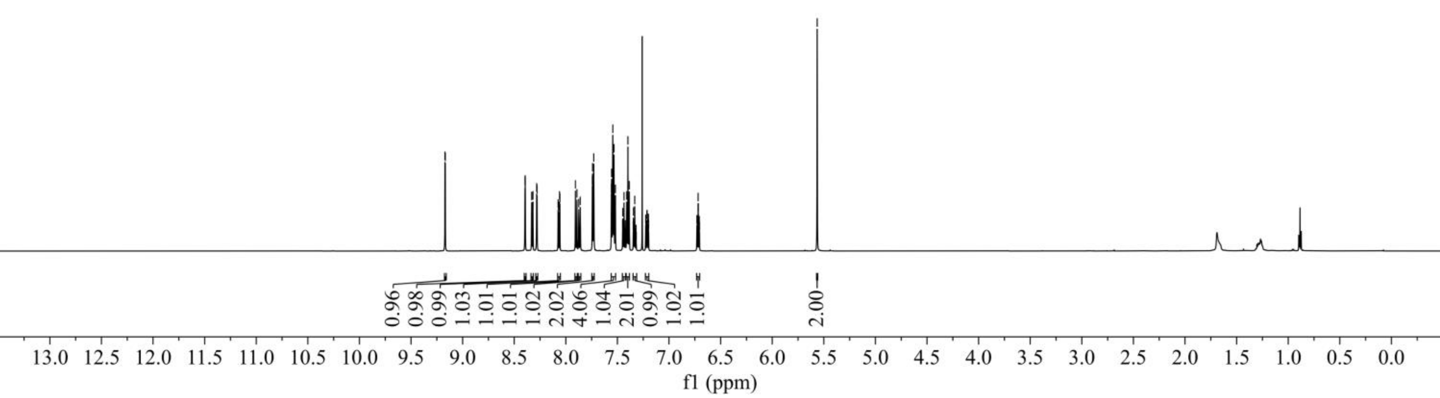

^{1}H NMR Spectrum ($CDCl_3$)

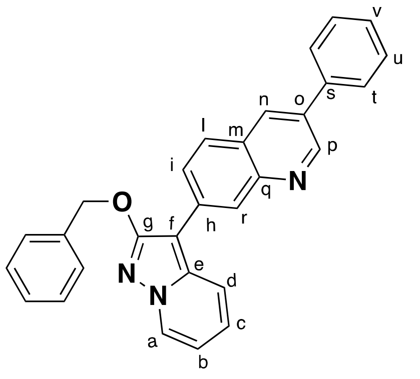

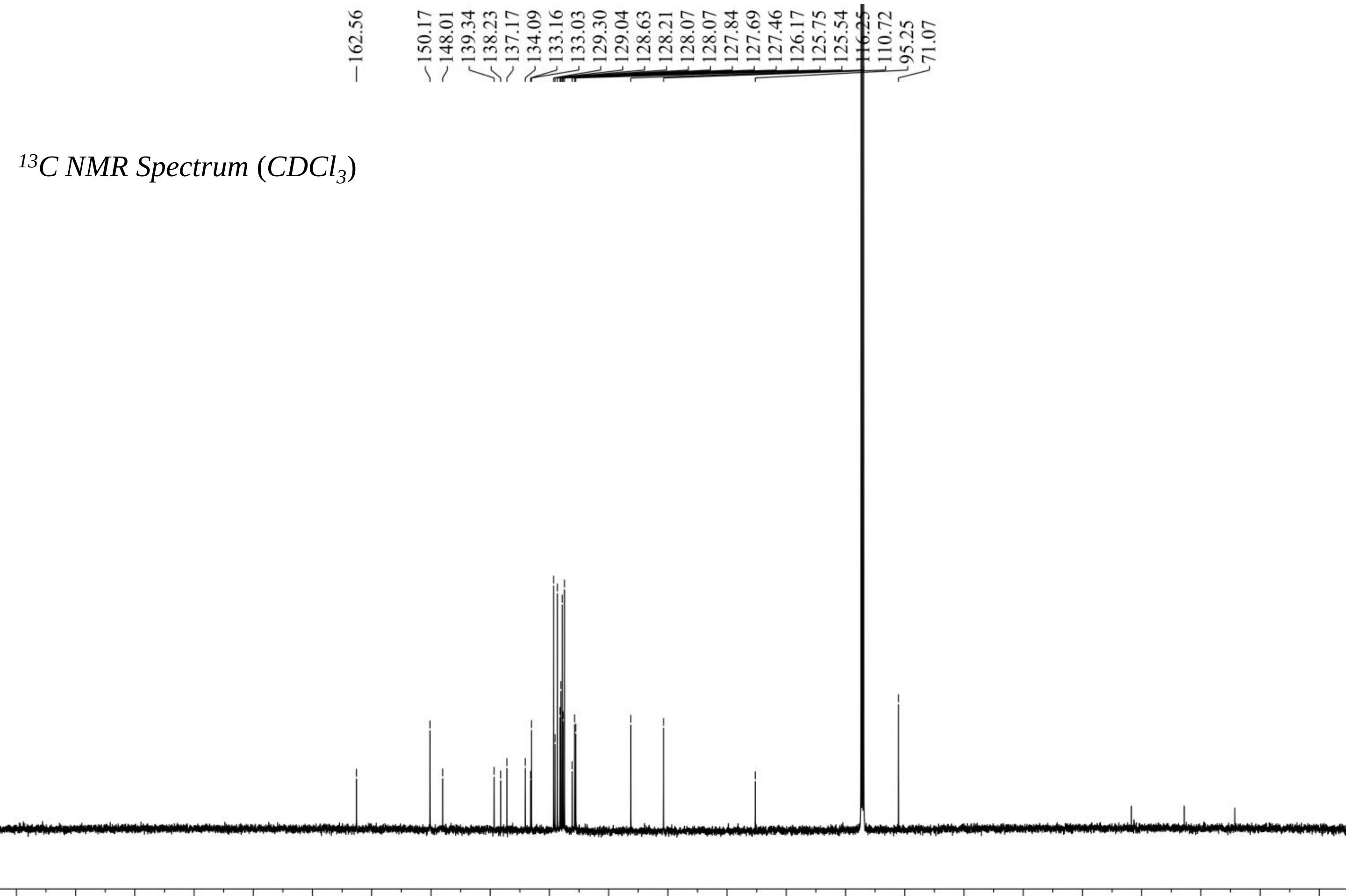

^{13}C NMR Spectrum ($CDCl_3$)

3-(3-phenylquinolin-7-yl)pyrazolo[1,5-a]pyridin-2-ol (**13**)

^{1}H NMR Spectrum (*DMSO*)

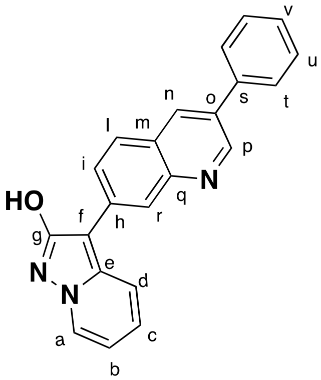

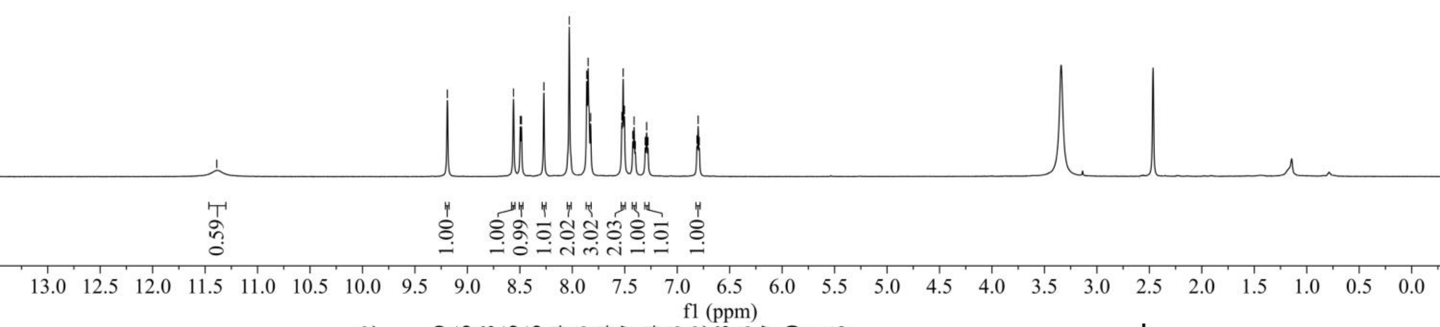

^{13}C NMR Spectrum (*DMSO*)

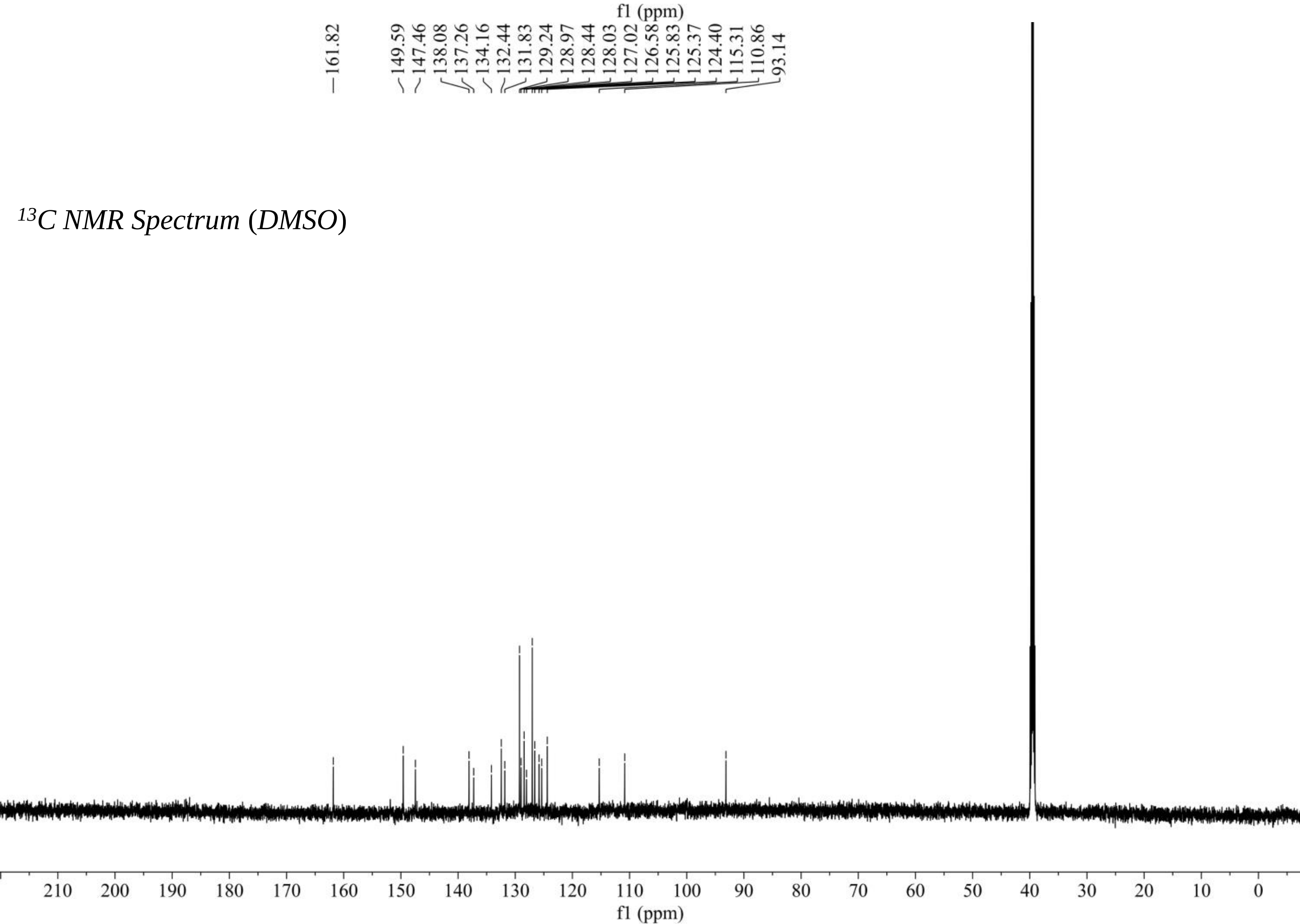

7-(2-(benzyloxy)pyrazolo[1,5-a]pyridin-3-yl)-3-(2,6-difluorophenyl)quinoline (**34**)

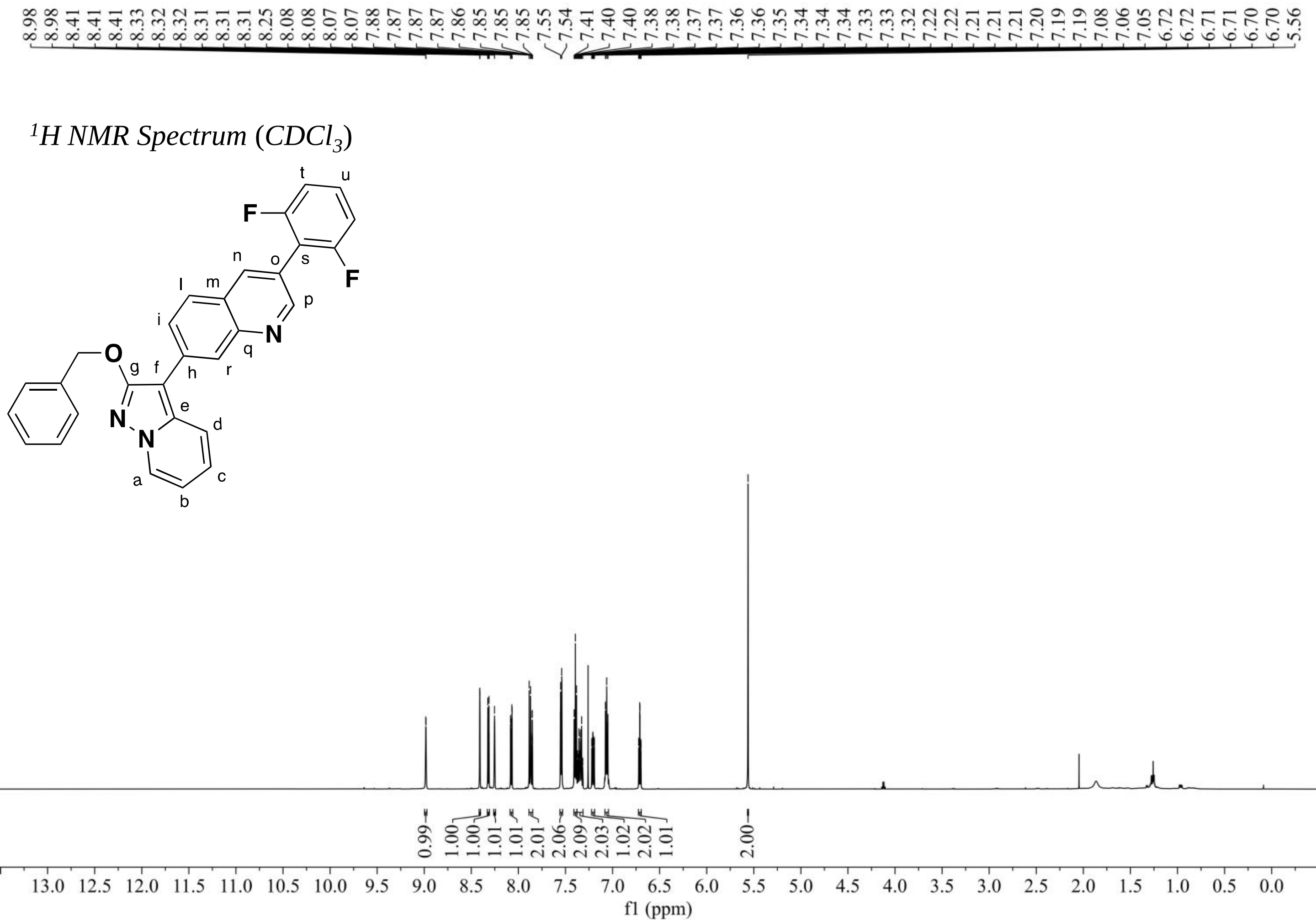

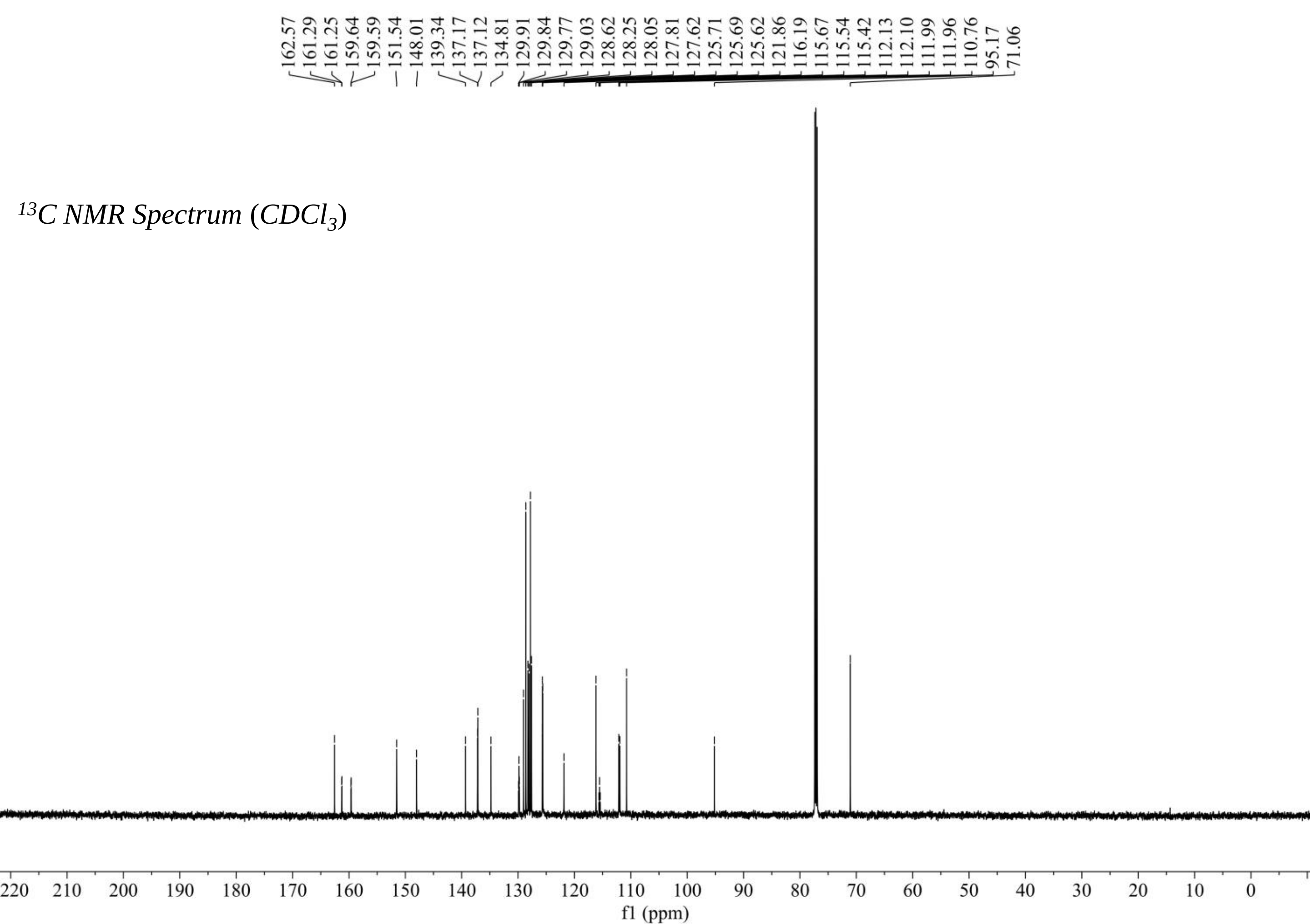

7-(2-(benzyloxy)pyrazolo[1,5-a]pyridin-3-yl)-3-(2,6-difluorophenyl)quinoline (**34**)

^{19}F NMR Spectrum (CDCl$_3$)

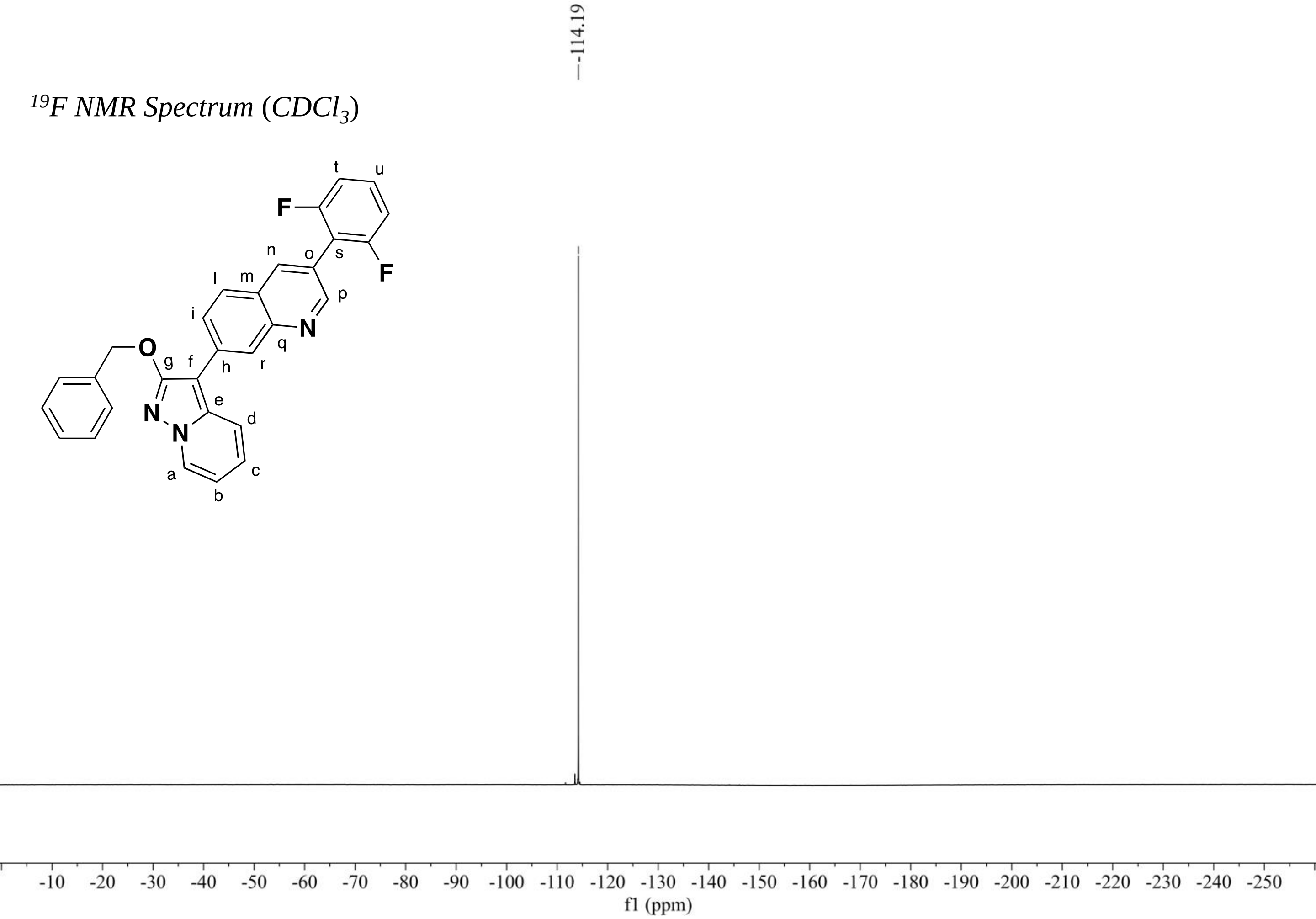

3-(3-(2,6-difluorophenyl)quinolin-7-yl)pyrazolo[1,5-a]pyridin-2-ol (**14**)

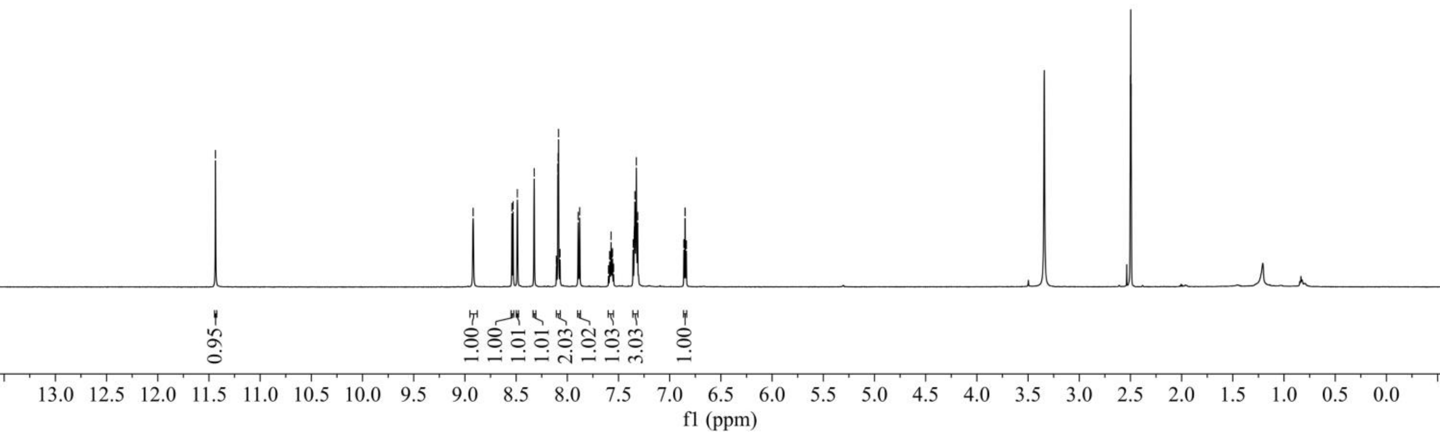

^{1}H NMR Spectrum (*DMSO*)

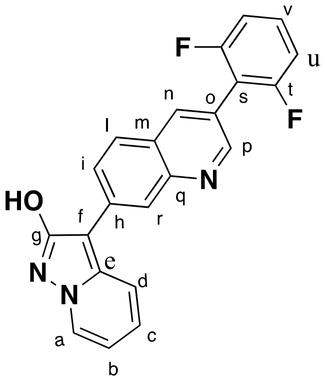

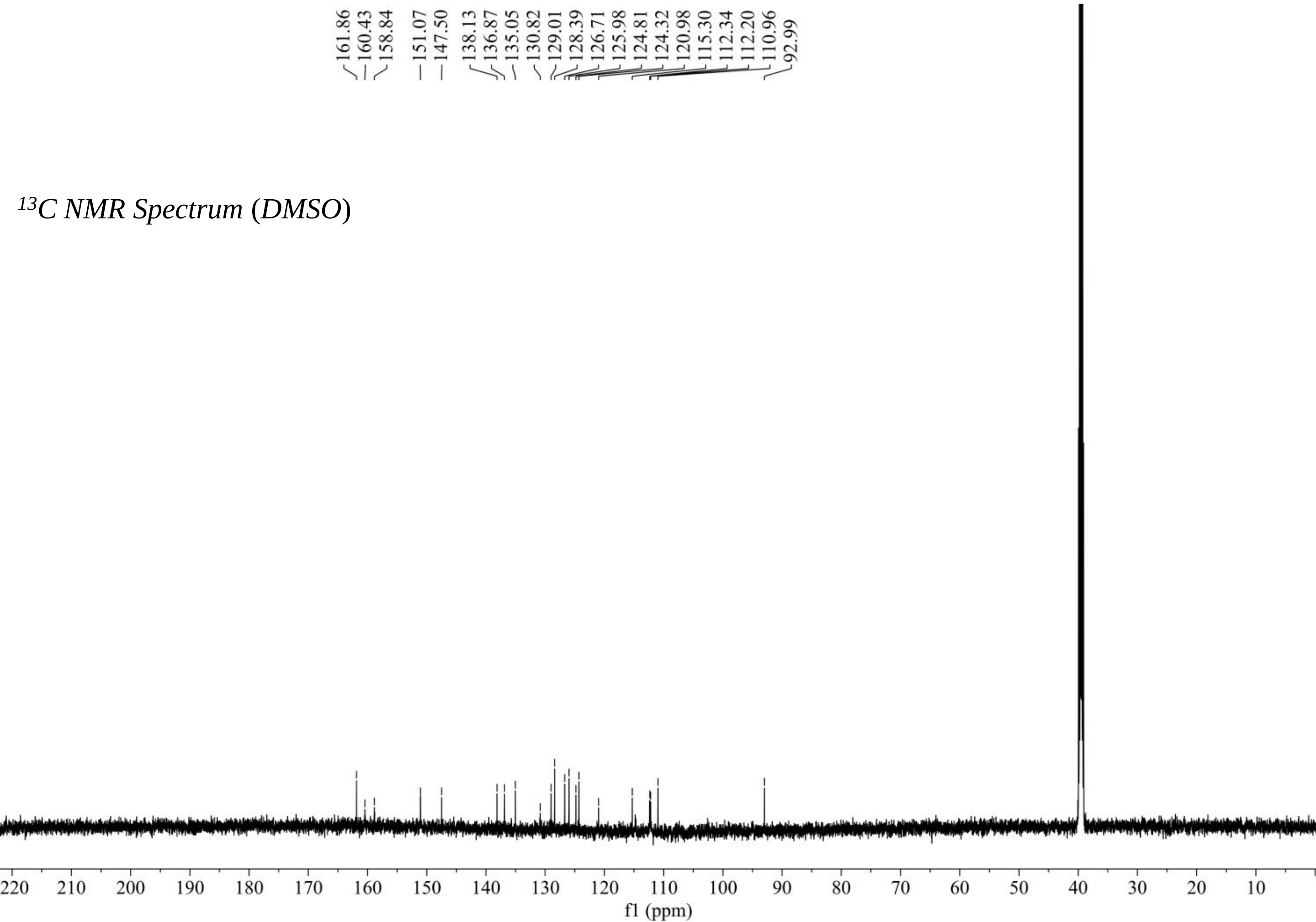

^{13}C NMR Spectrum (*DMSO*)

3-(3-(2,6-difluorophenyl)quinolin-7-yl)pyrazolo[1,5-a]pyridin-2-ol (**14**)

-114.79

^{19}F NMR Spectrum (*DMSO*)

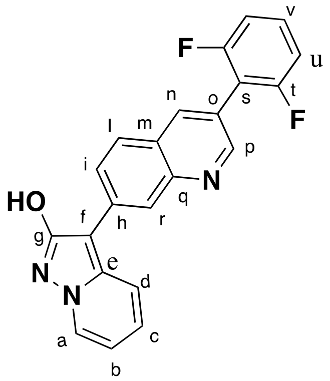

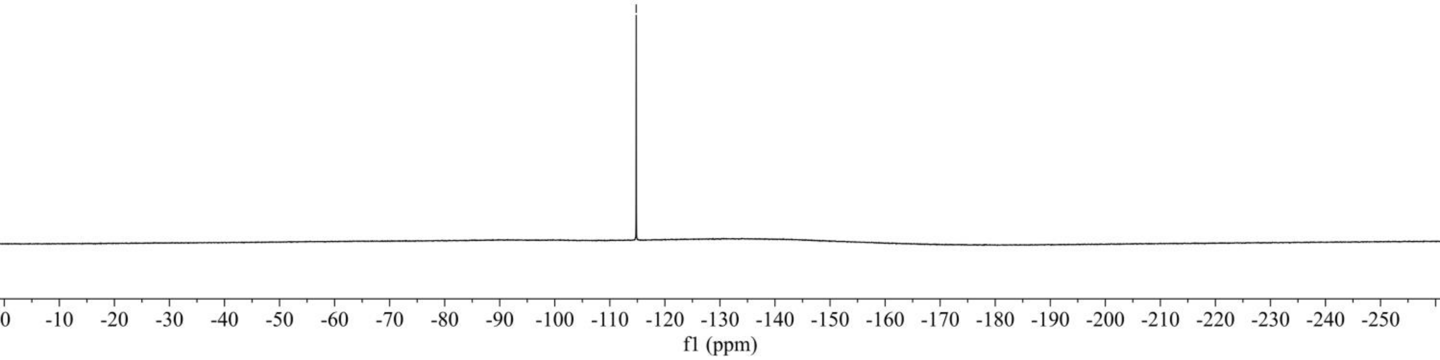

7-bromo-3-phenylquinoline (**45**)

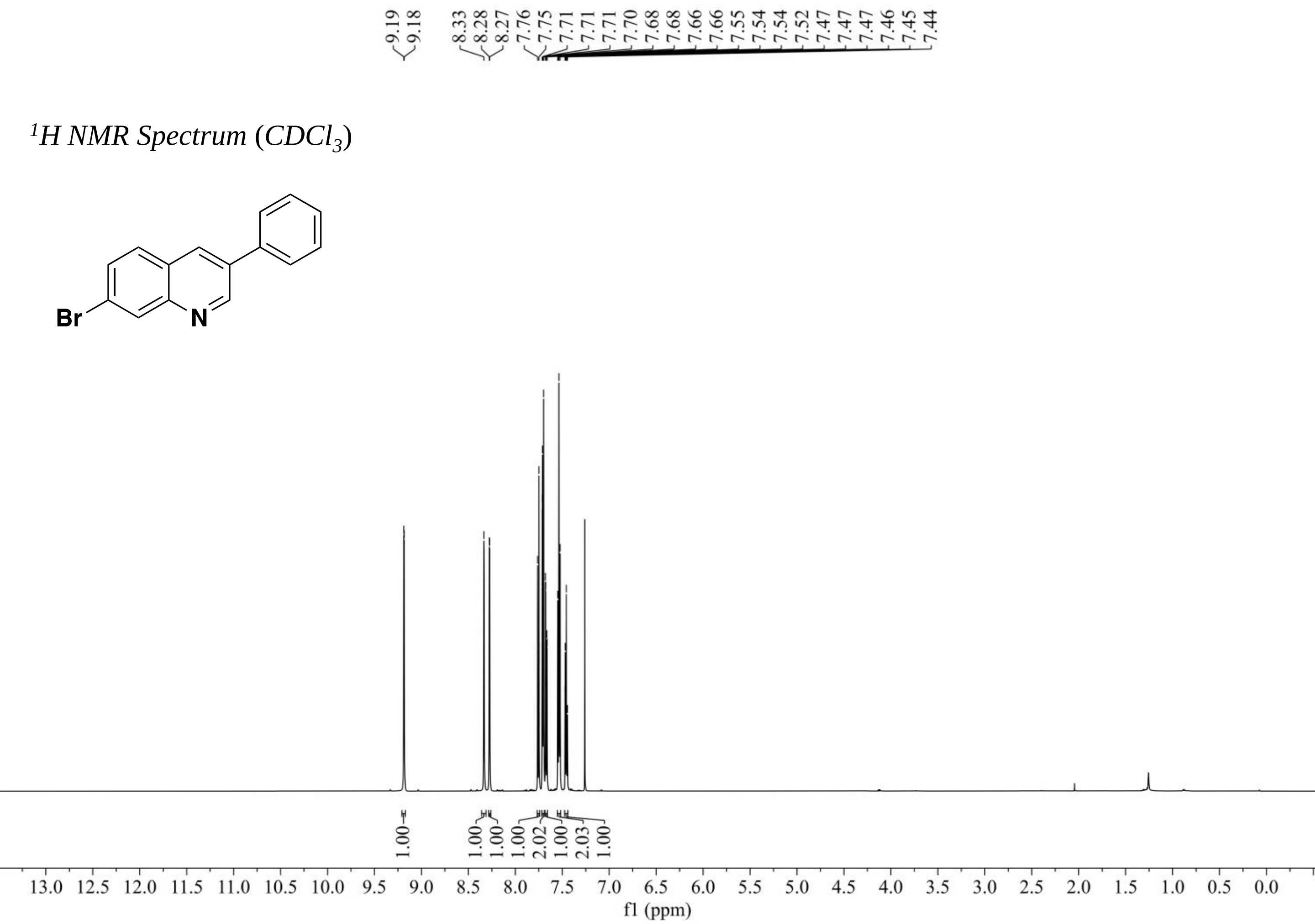

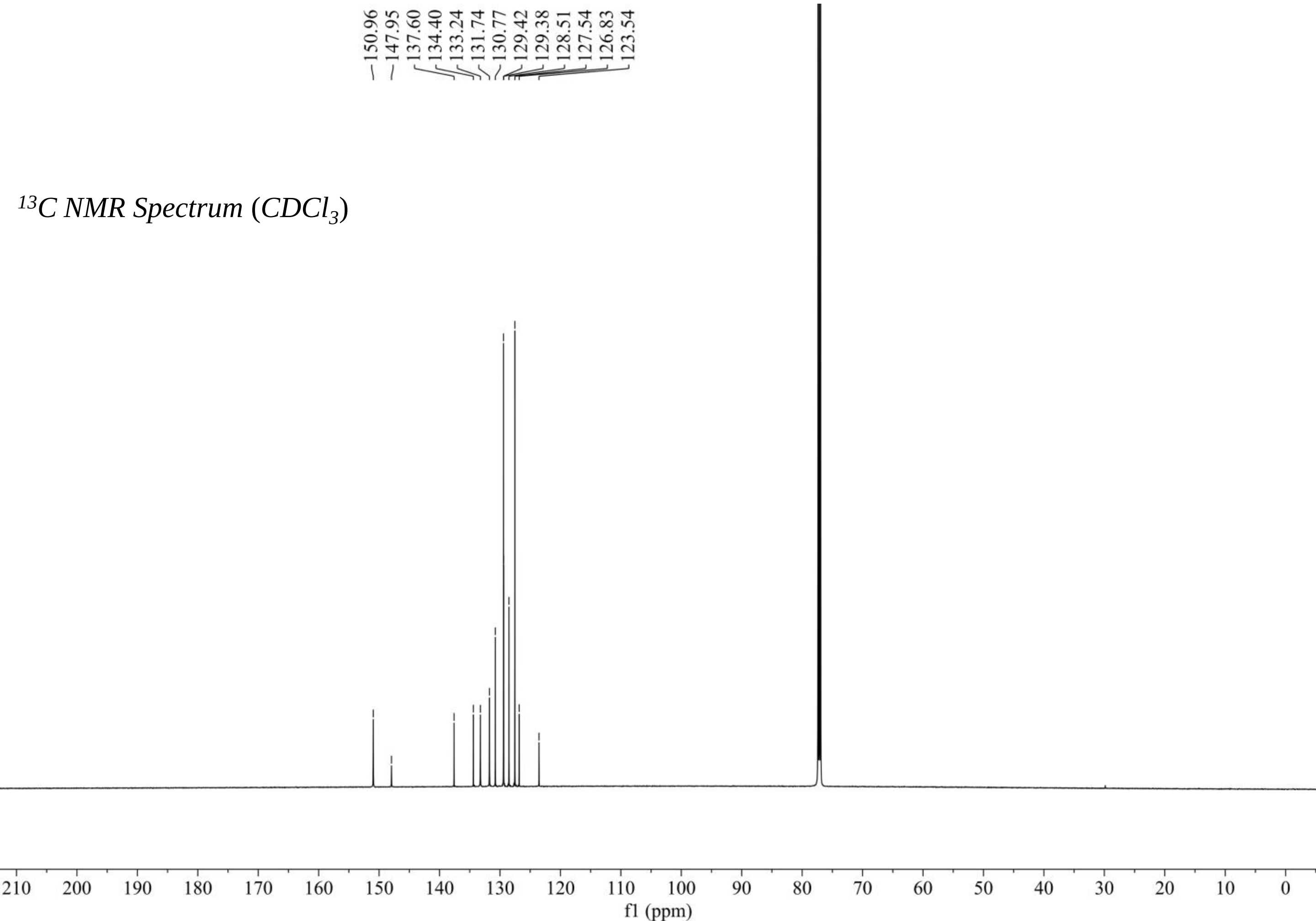

3-phenyl-7-(4,4,5,5-tetramethyl-1,3,2-dioxaborolan-2-yl)quinoline (**46**)

^{1}H NMR Spectrum ($CDCl_3$)

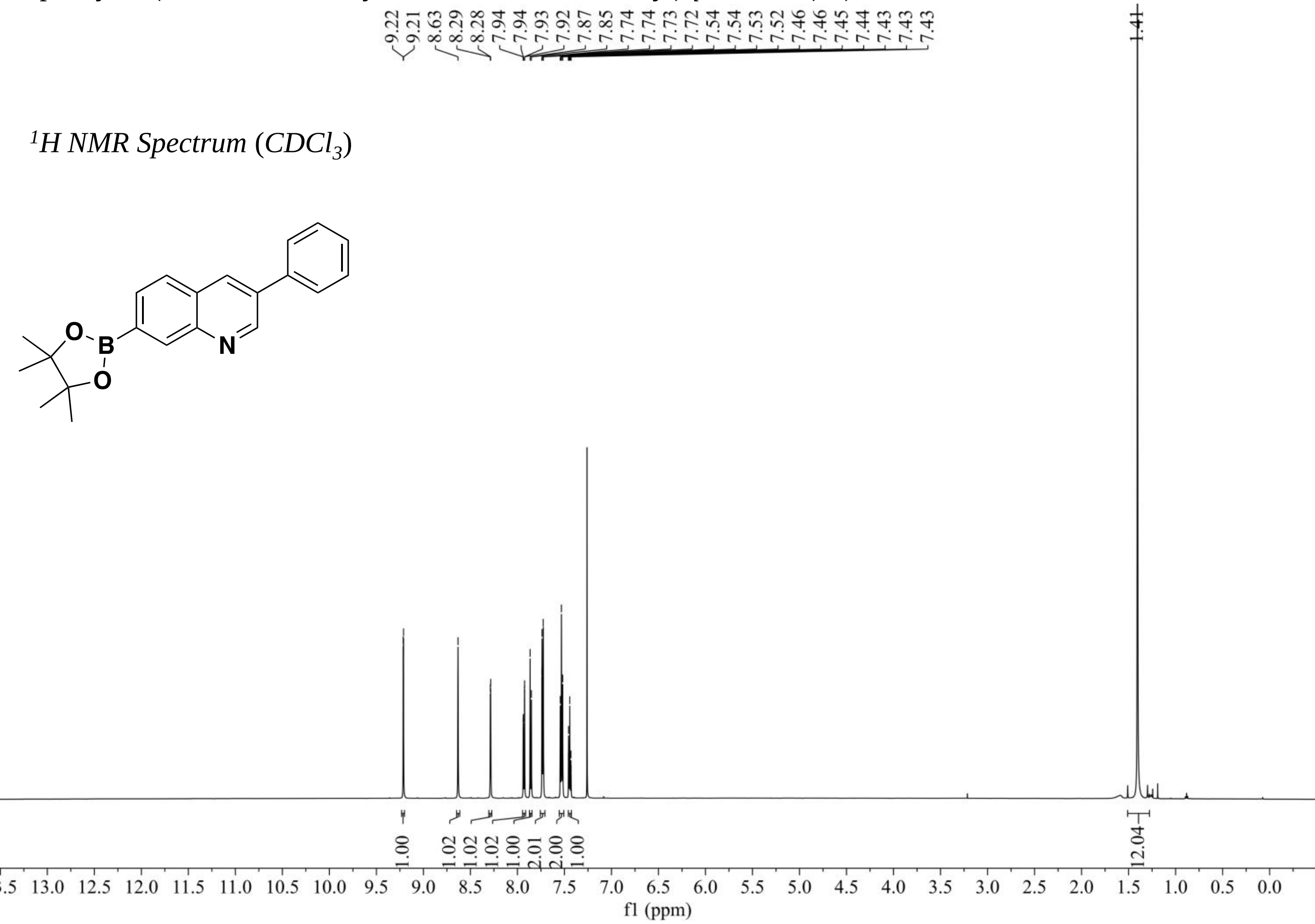

^{13}C NMR Spectrum ($CDCl_3$)

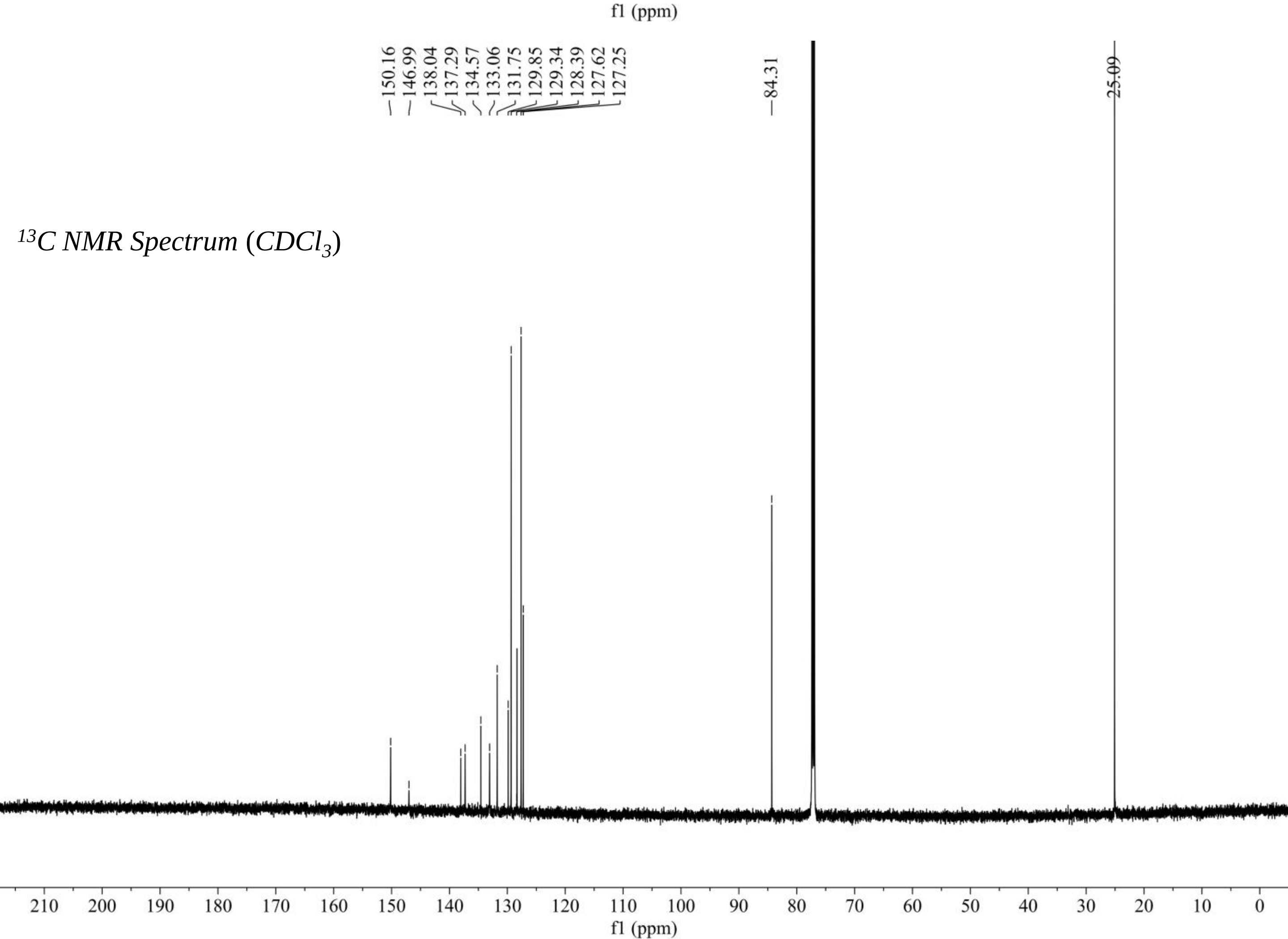

3-phenyl-7-(4,4,5,5-tetramethyl-1,3,2-dioxaborolan-2-yl)quinoline (**46**)

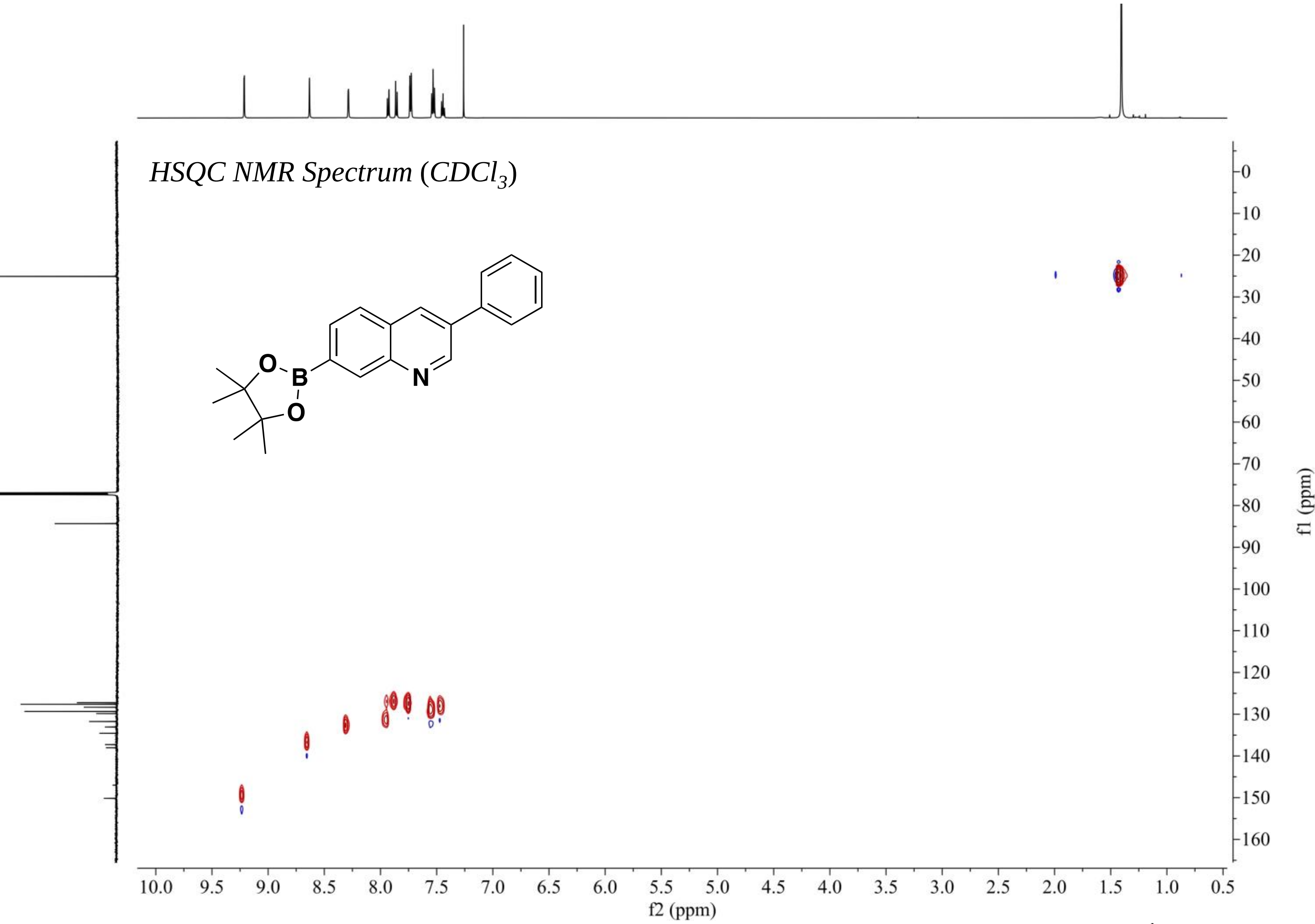

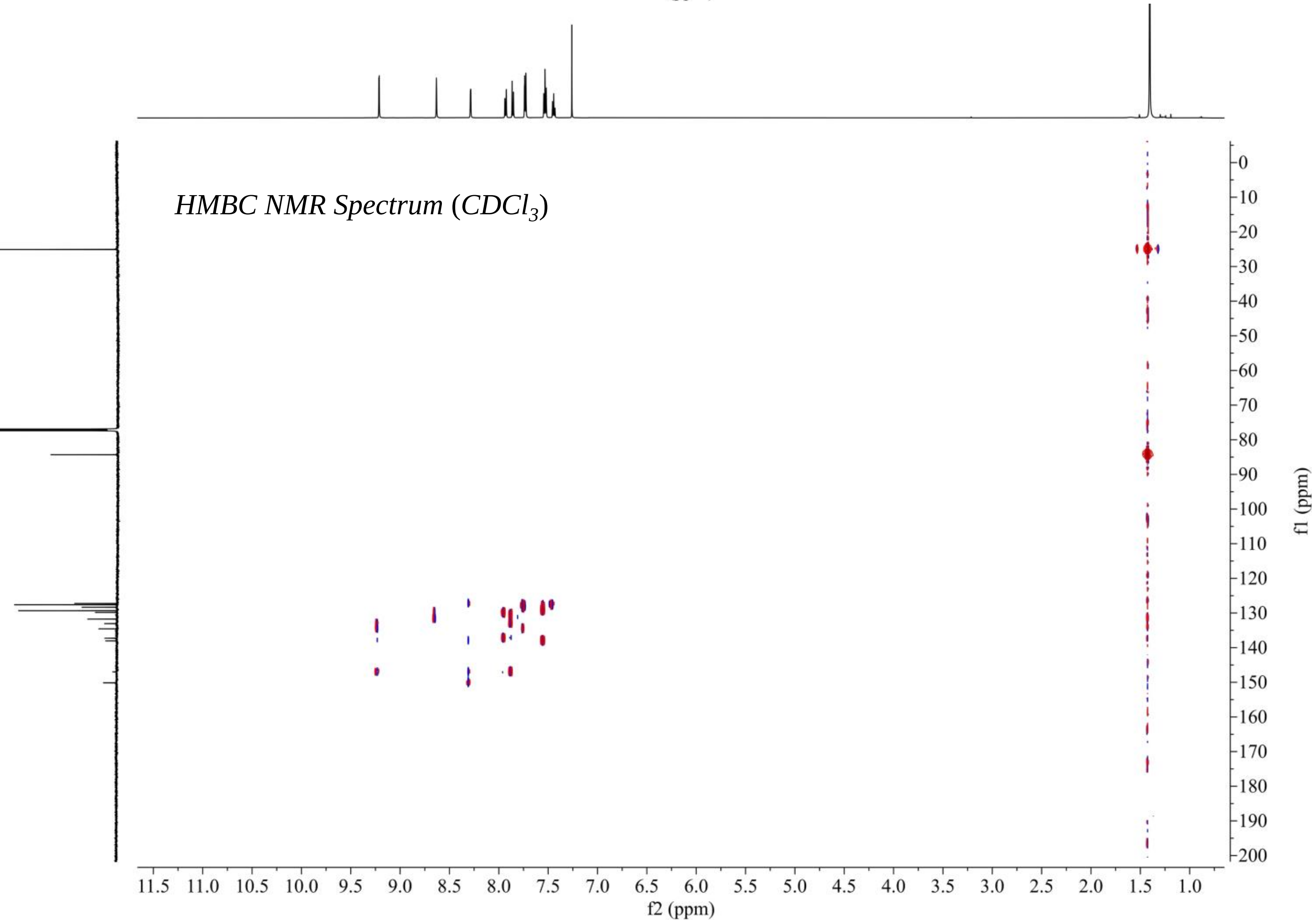

3-phenyl-7-(4,4,5,5-tetramethyl-1,3,2-dioxaborolan-2-yl)quinoline (**46**)

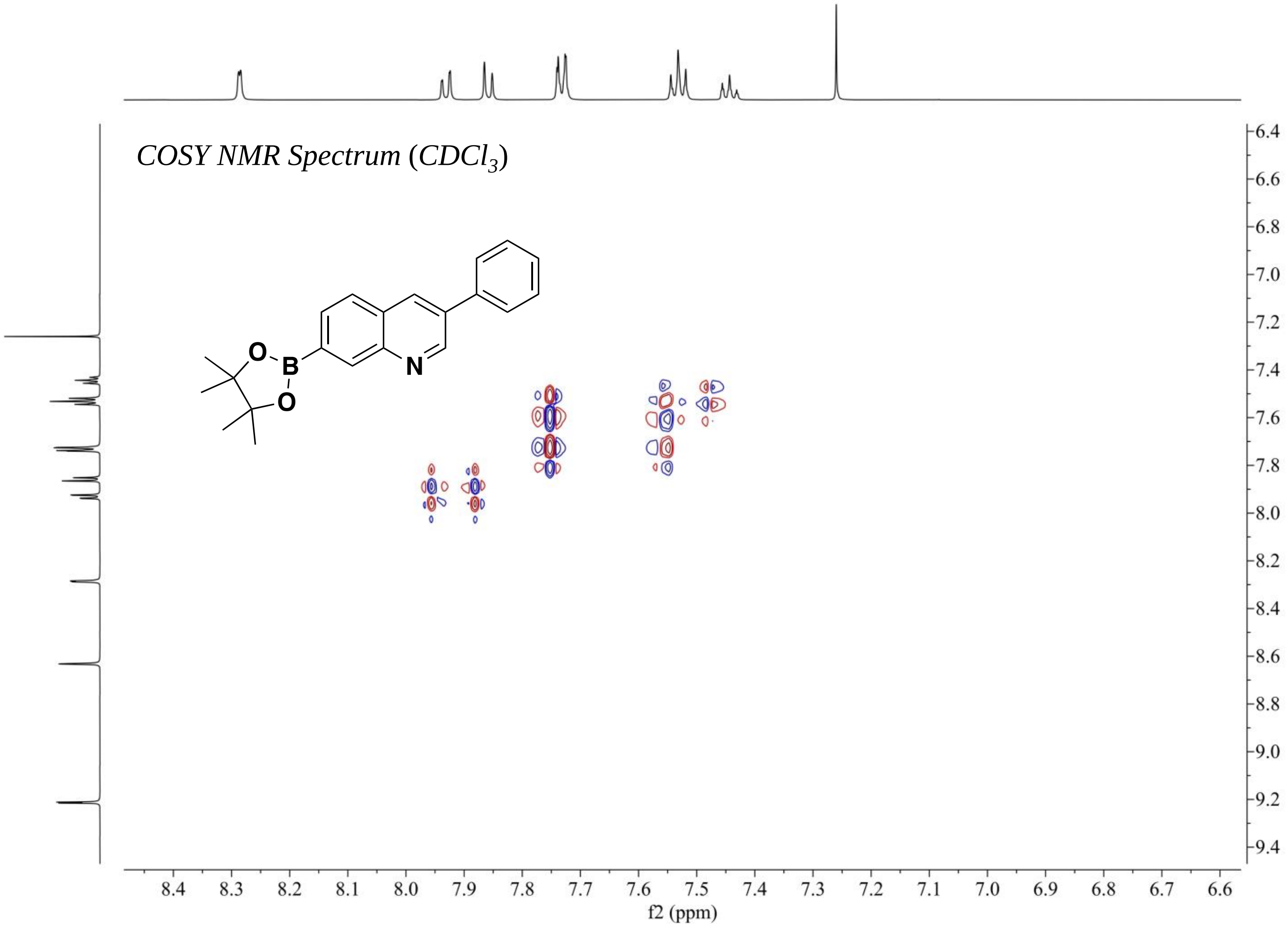

Ethyl 2-hydroxypyrazolo[1,5-a]pyridine-3-carboxylate (**2**)

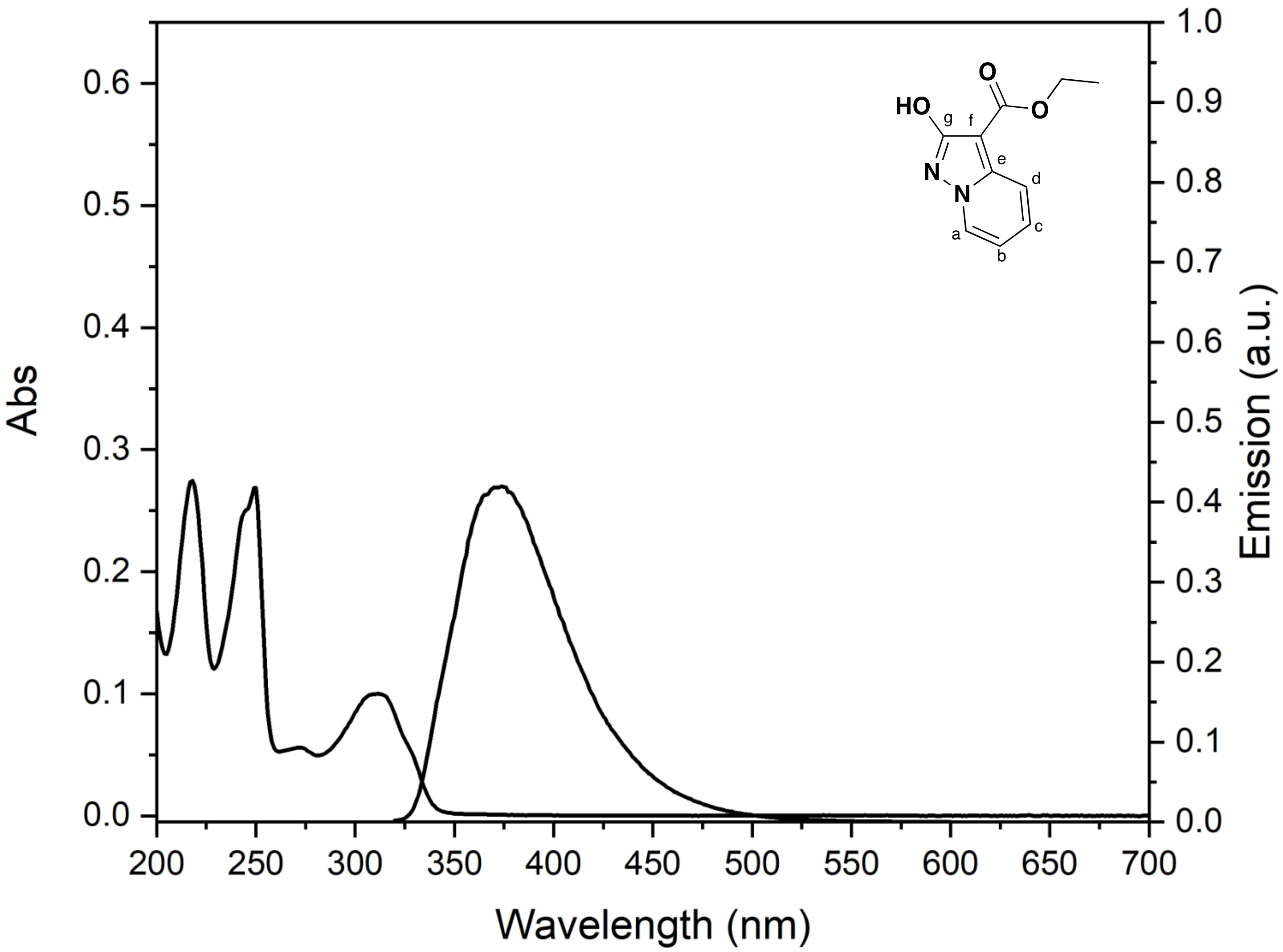

Ethyl 2-methoxypyrazolo[1,5-a]pyridine-3-carboxylate (**2a**)

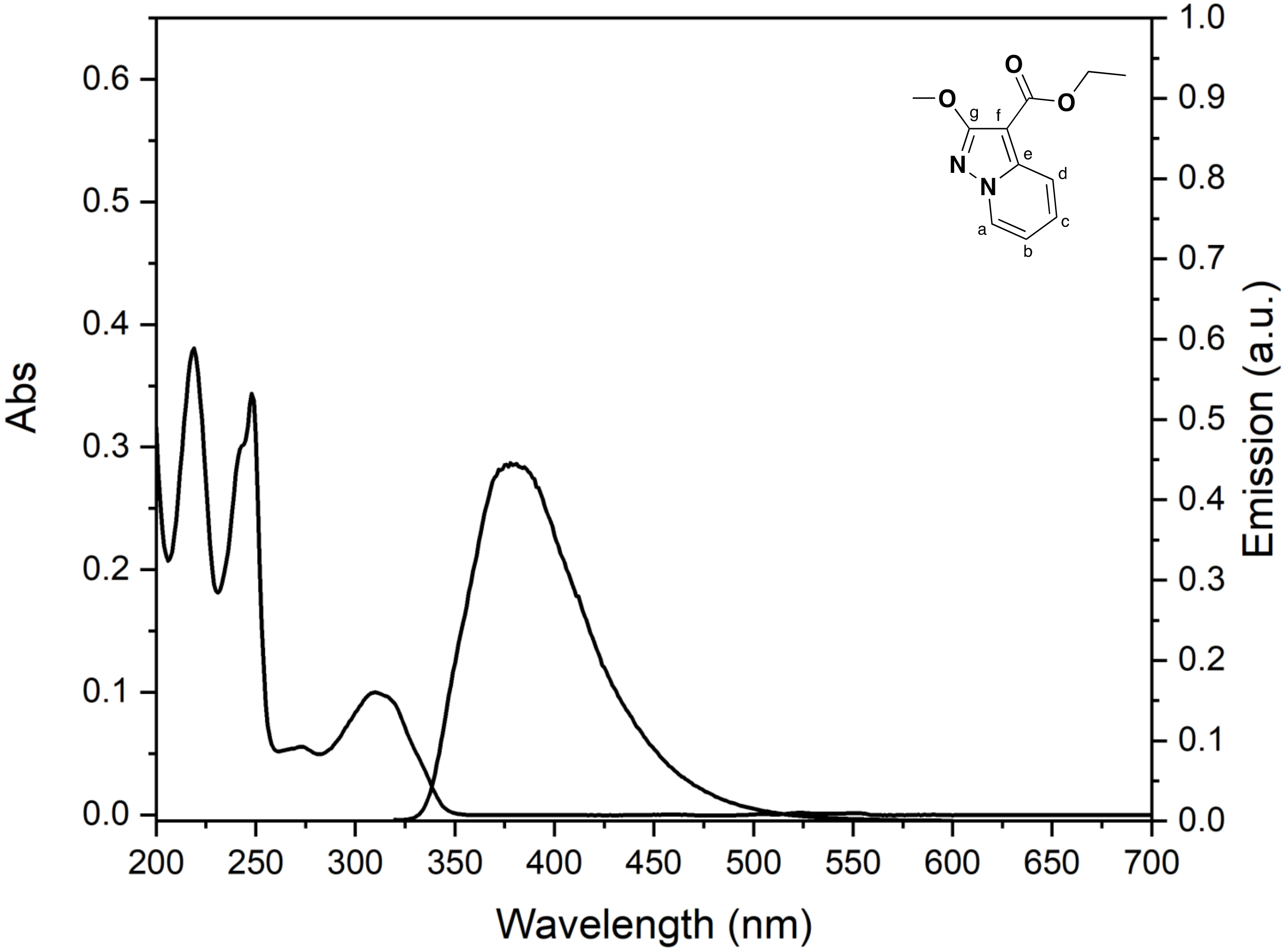

Ethyl 1-methyl-2-oxo-1,2-dihydropyrazolo[1,5-a]pyridine-3-carboxylate (**2b**)

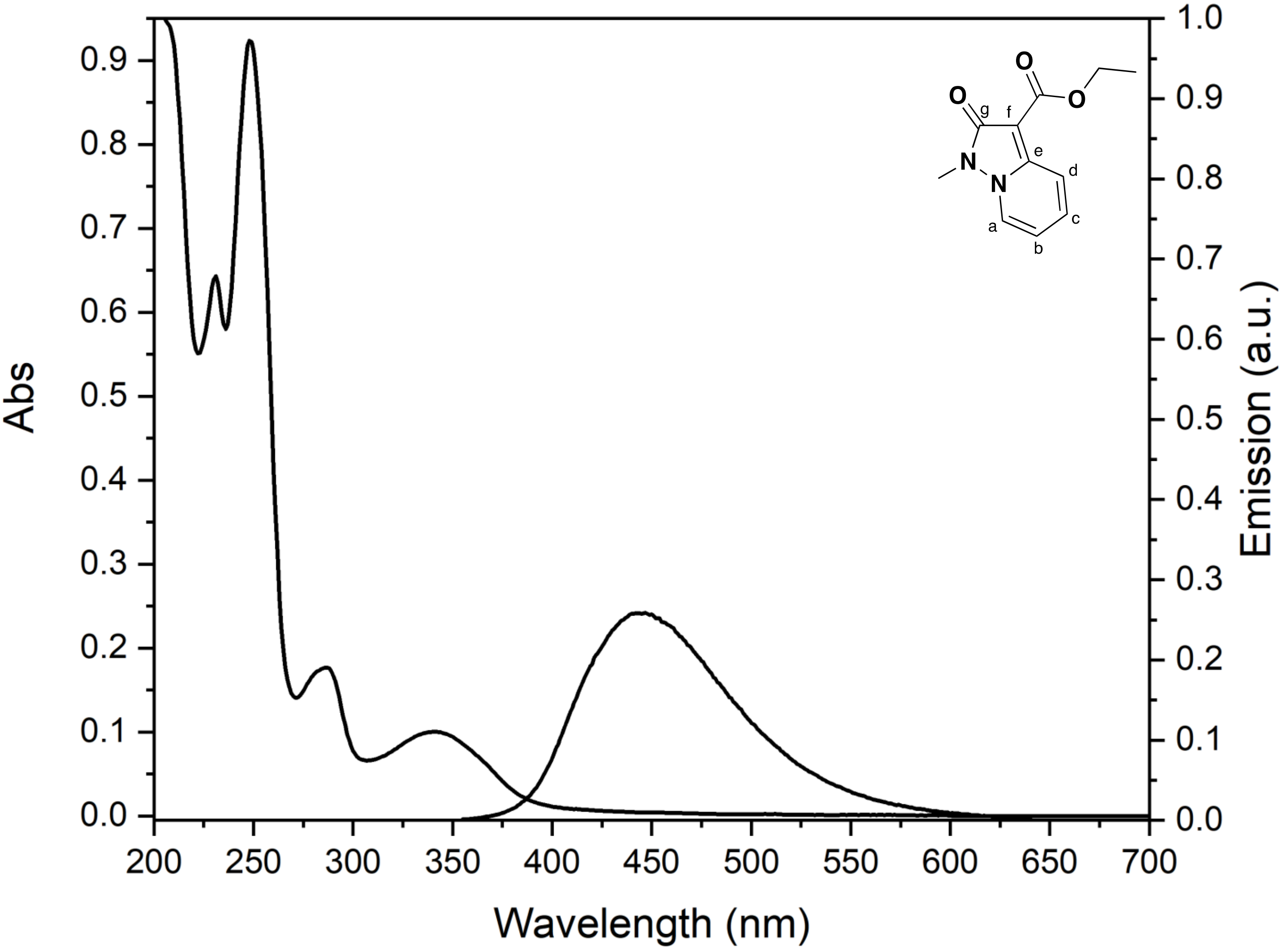

Ethyl 2-methoxypyrazolo[1,5-a]pyridine-3-carboxylate (**2c**)

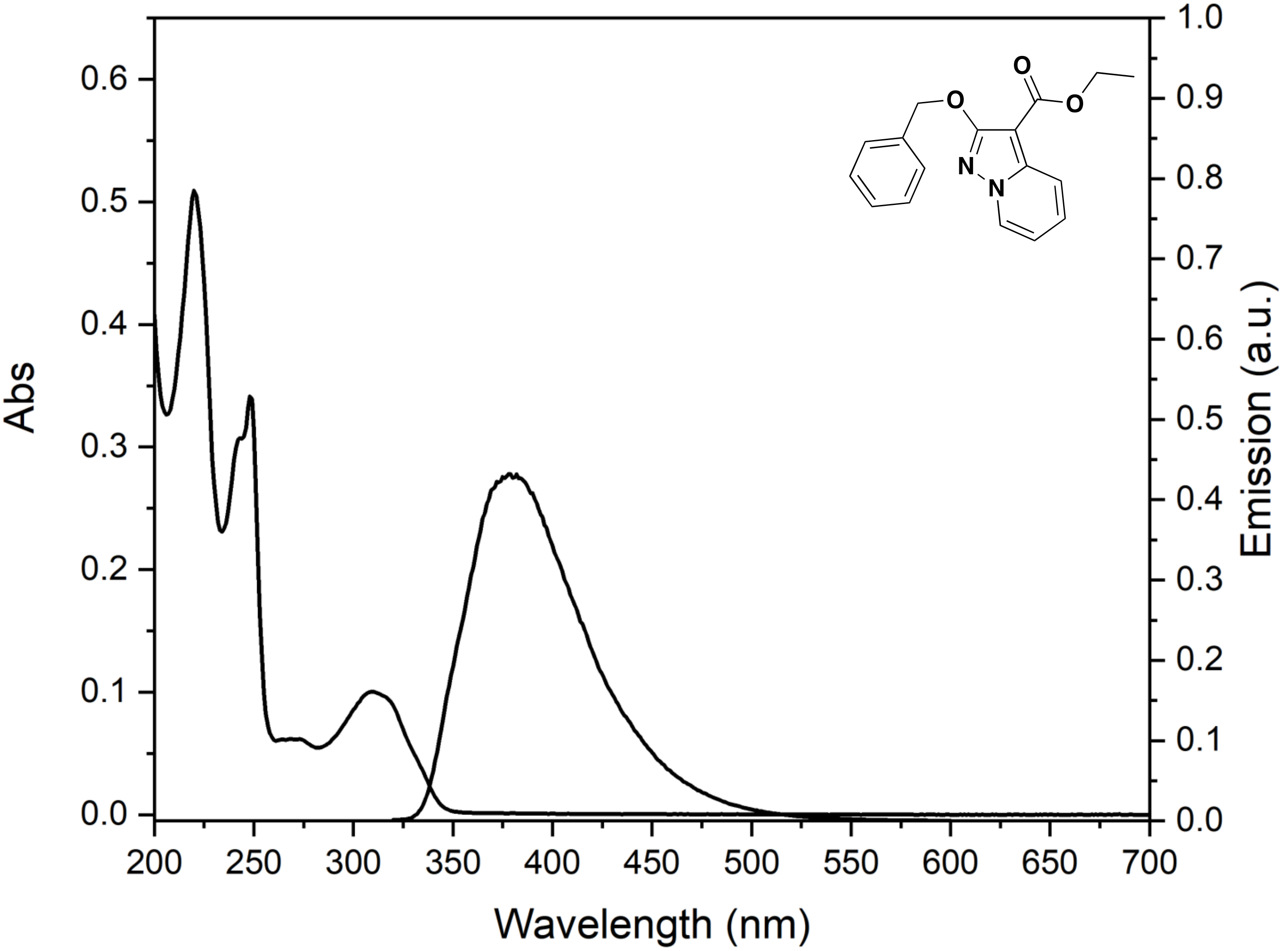

Ethyl 1-benzyl-2-oxo-1,2-dihydropyrazolo[1,5-a]pyridine-3-carboxylate (**2d**)

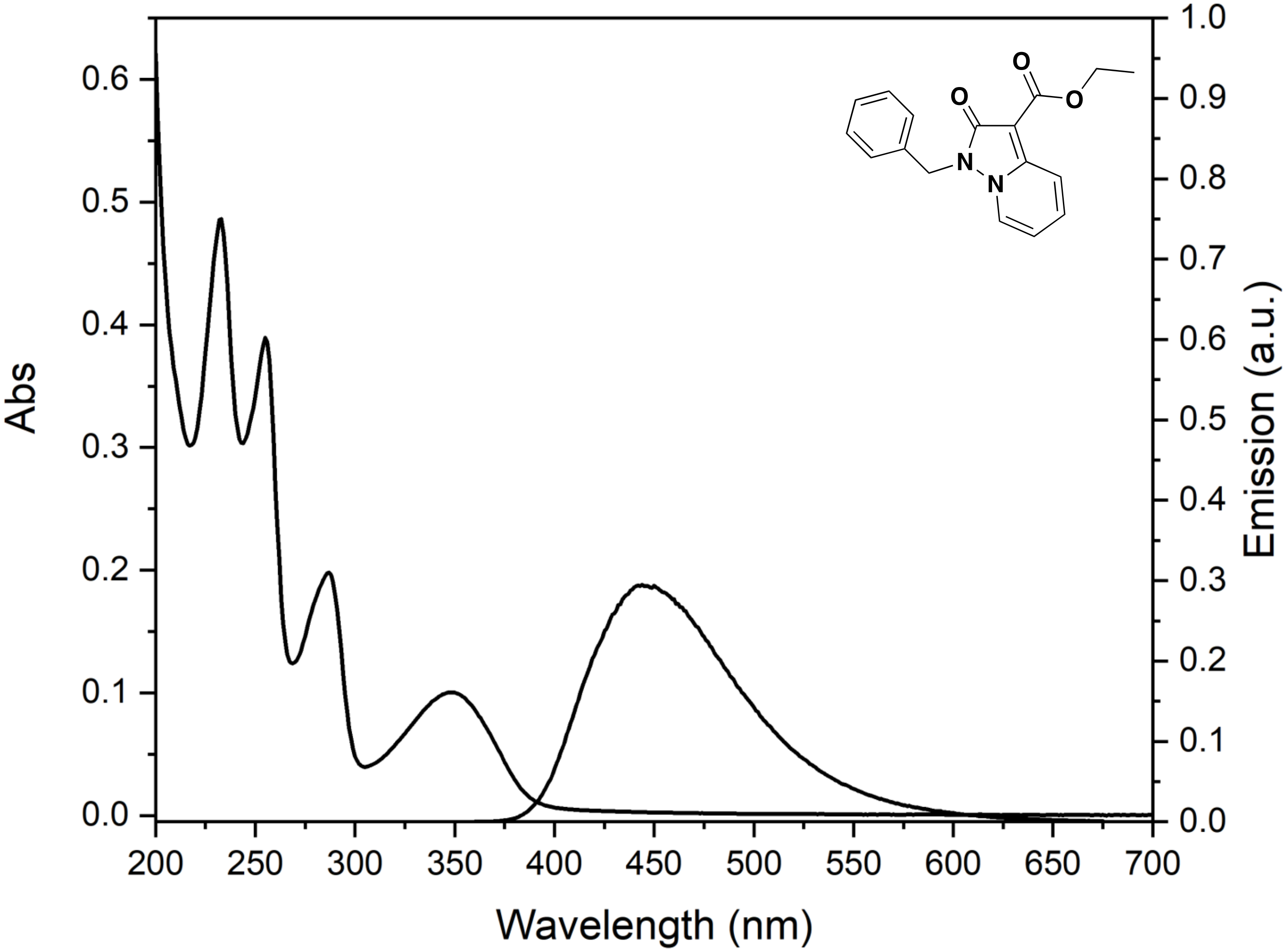

Pyrazolo[1,5-a]pyridin-2-ol (**1**)

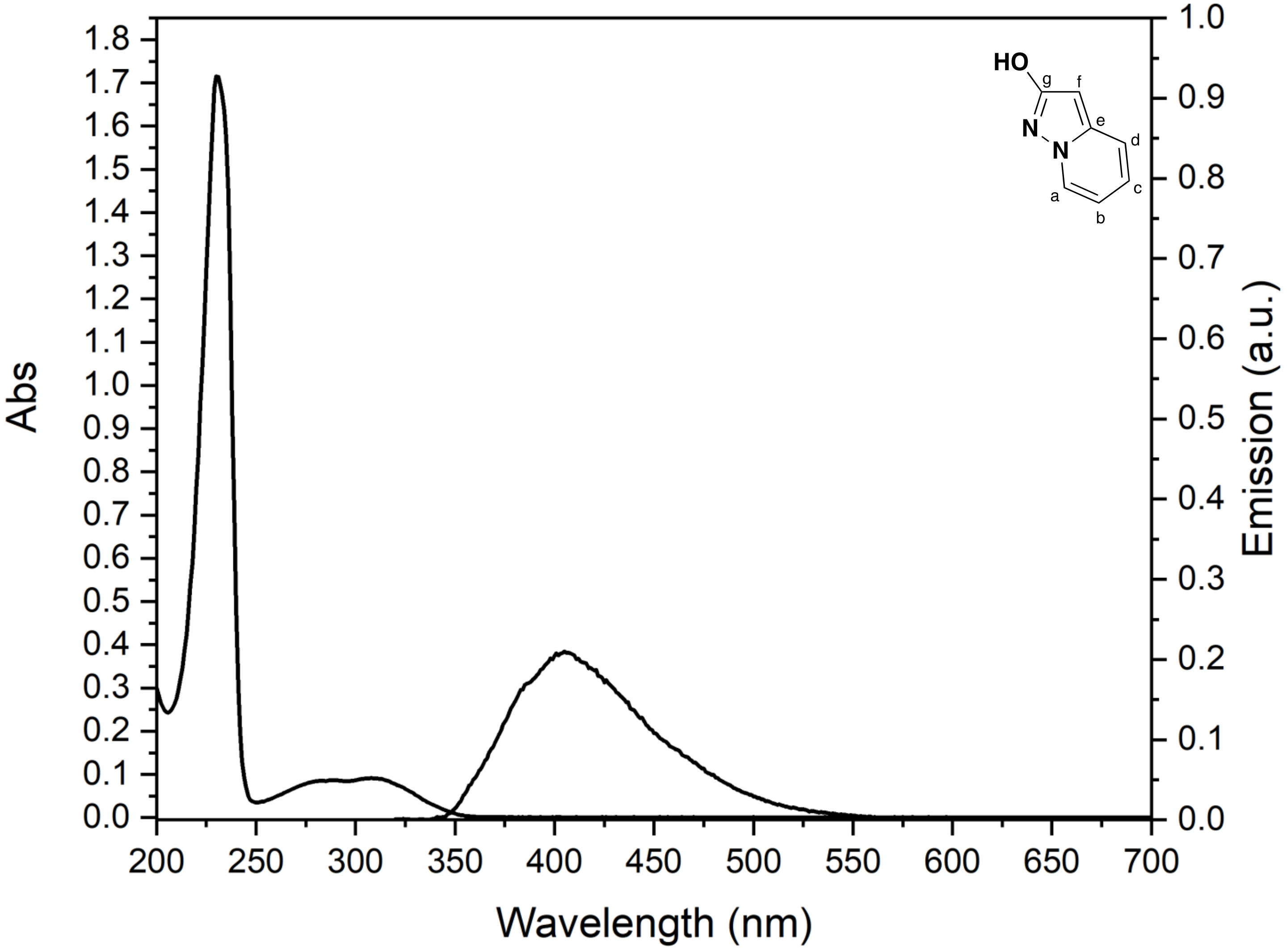

2-Methoxypyrazolo[1,5-a]pyridine (**1a**)

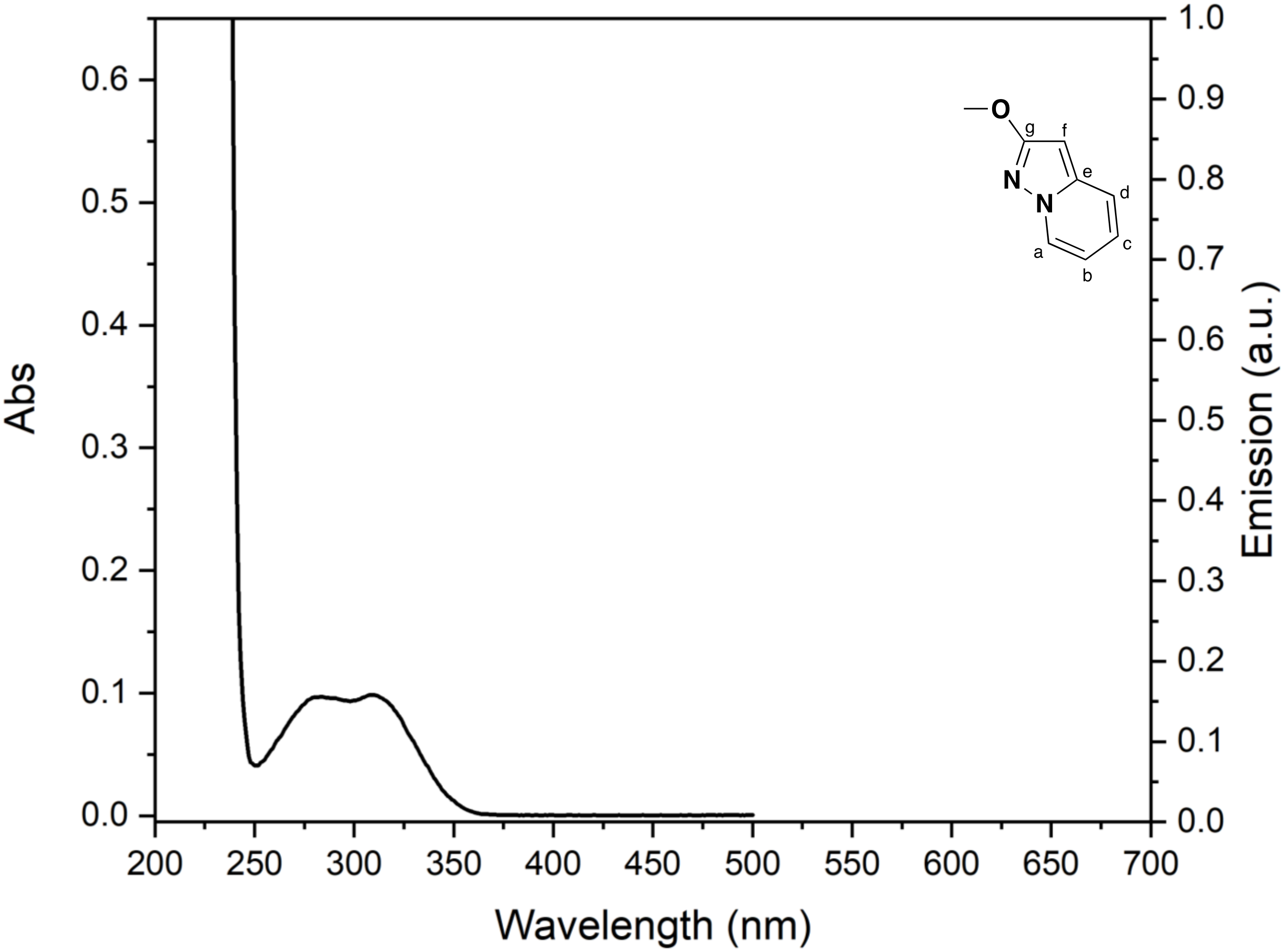

1-Methylpyrazolo[1,5-a]pyridin-2(1H)-one (**1b**)

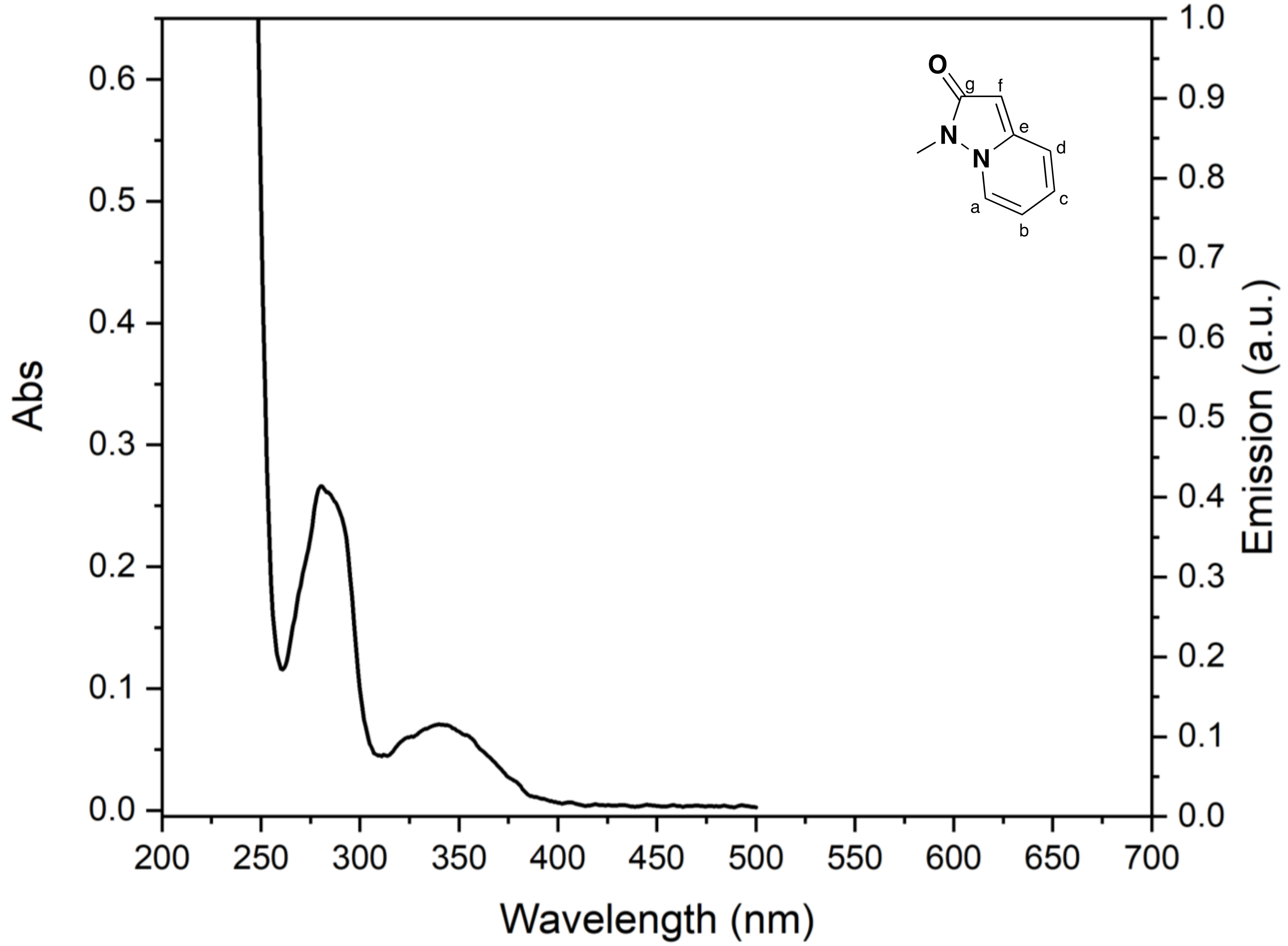

3-Nitropyrazolo[1,5-a]pyridin-2-ol (**3**)

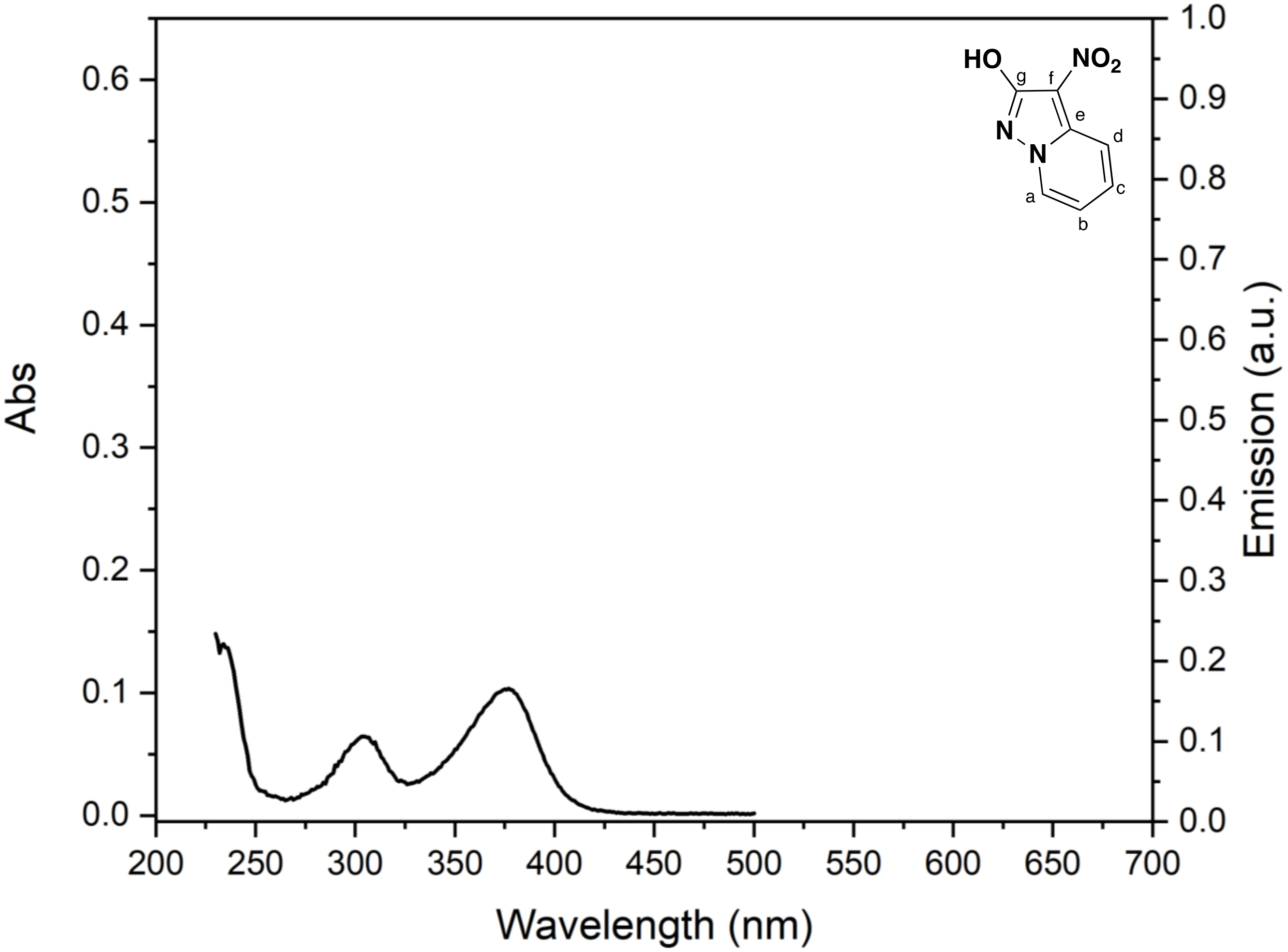

N-(2-Methoxypyrazolo[1,5-a]pyridin-3-yl)acetamide (**3a**)

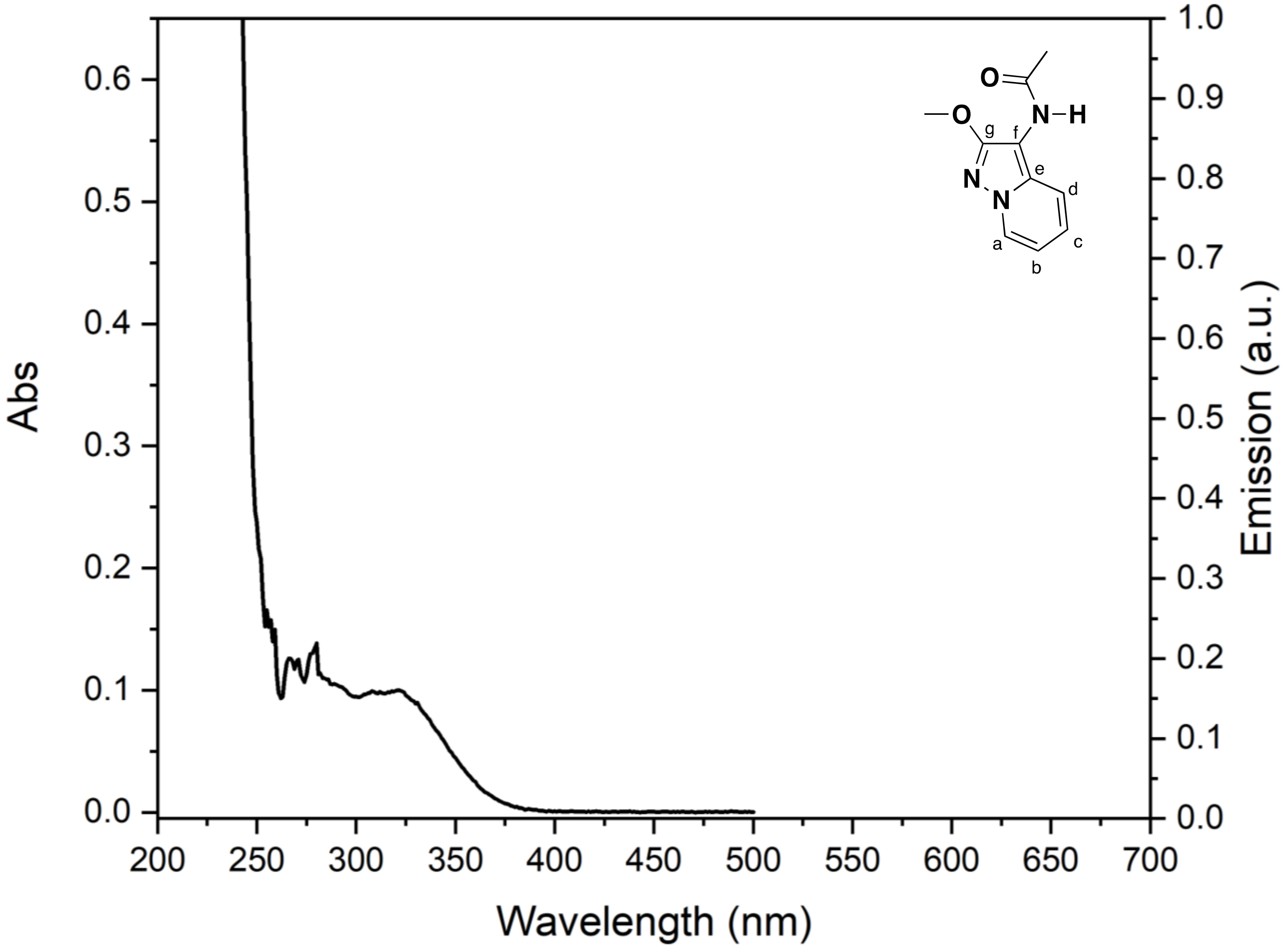

N-(2-Methoxypyrazolo[1,5-*a*]pyridin-3-yl)methanesulfonamide (**3b**)

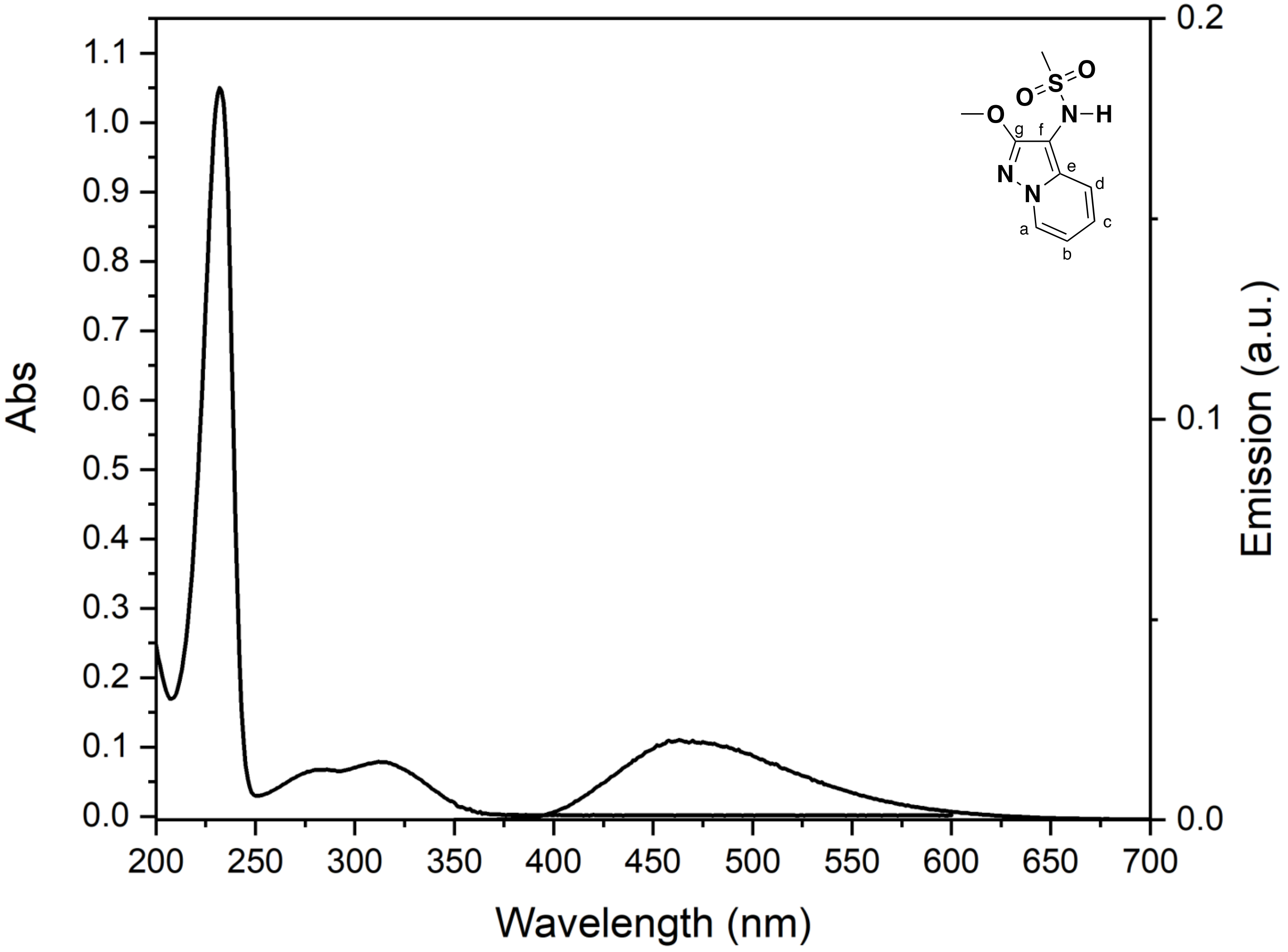

(E)-2-(2-methoxypyrazolo[1,5-a]pyridin-3-yl)vinyl)malononitrile (**4a**)

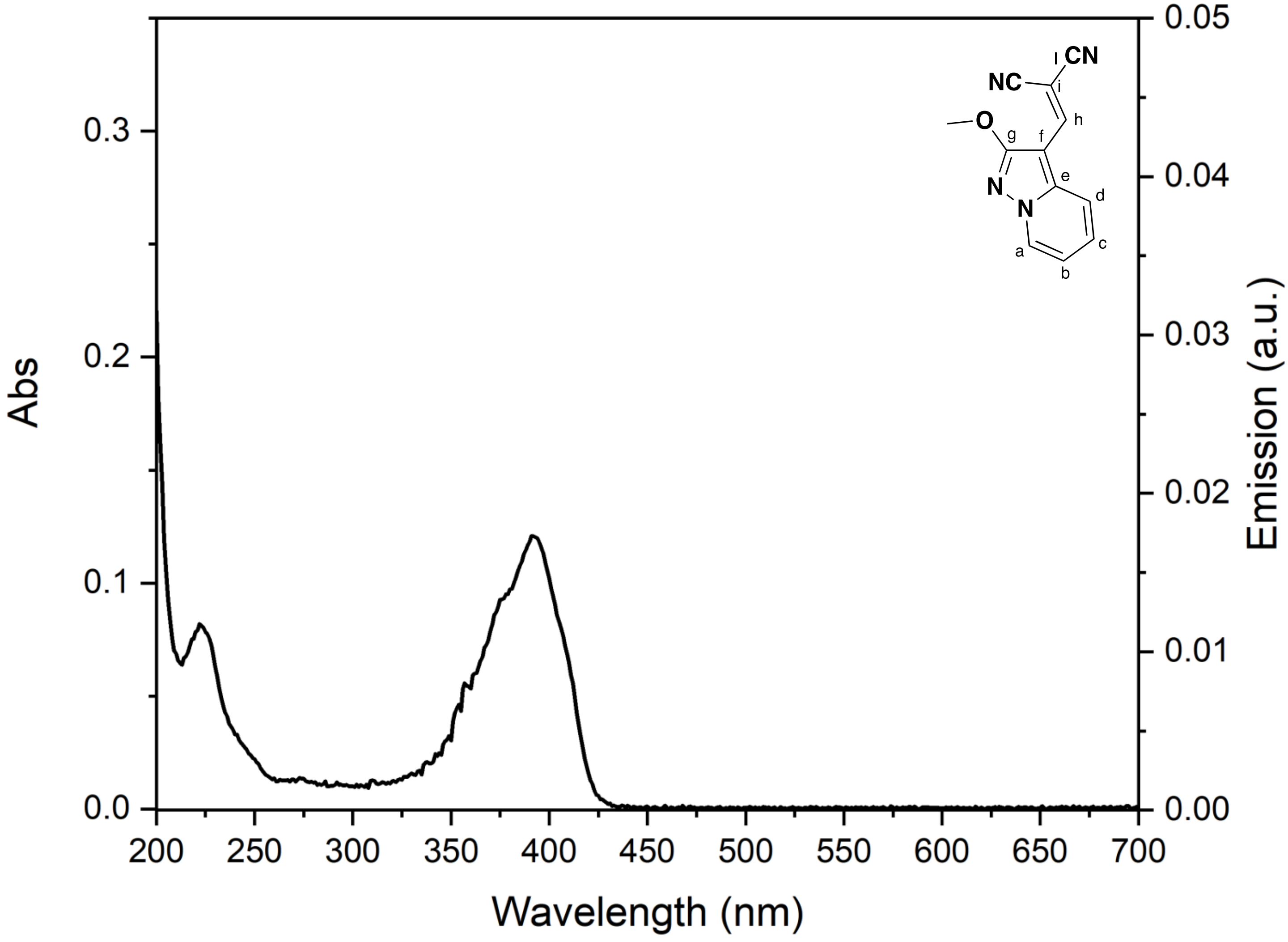

Ethyl (E)-2-cyano-4-(2-methoxypyrazolo[1,5-a]pyridin-3-yl)but-3-enoate (**4b**)

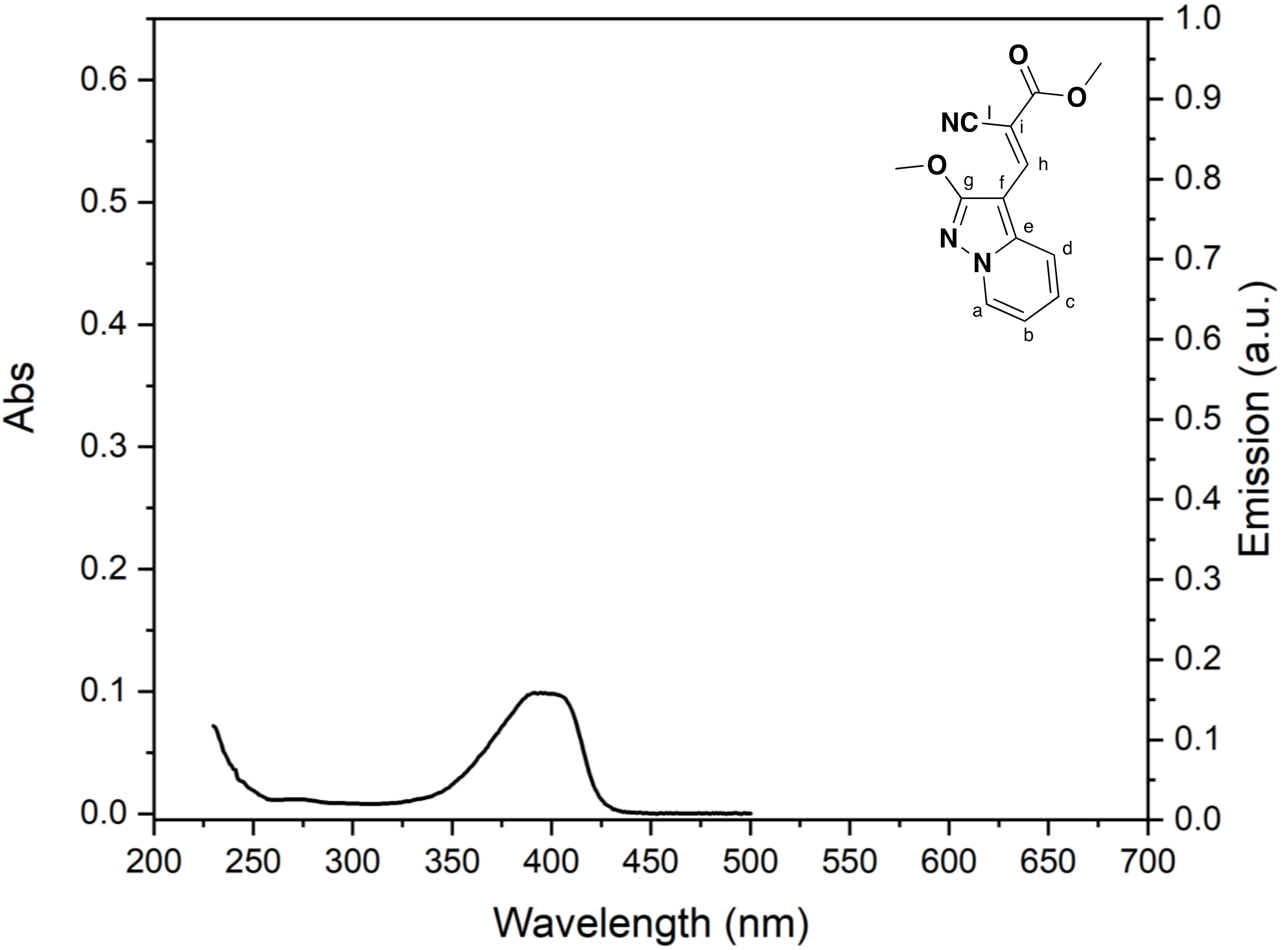

(E)-2-methoxy-3-styrylpyrazolo[1,5-a]pyridine (**5a**)

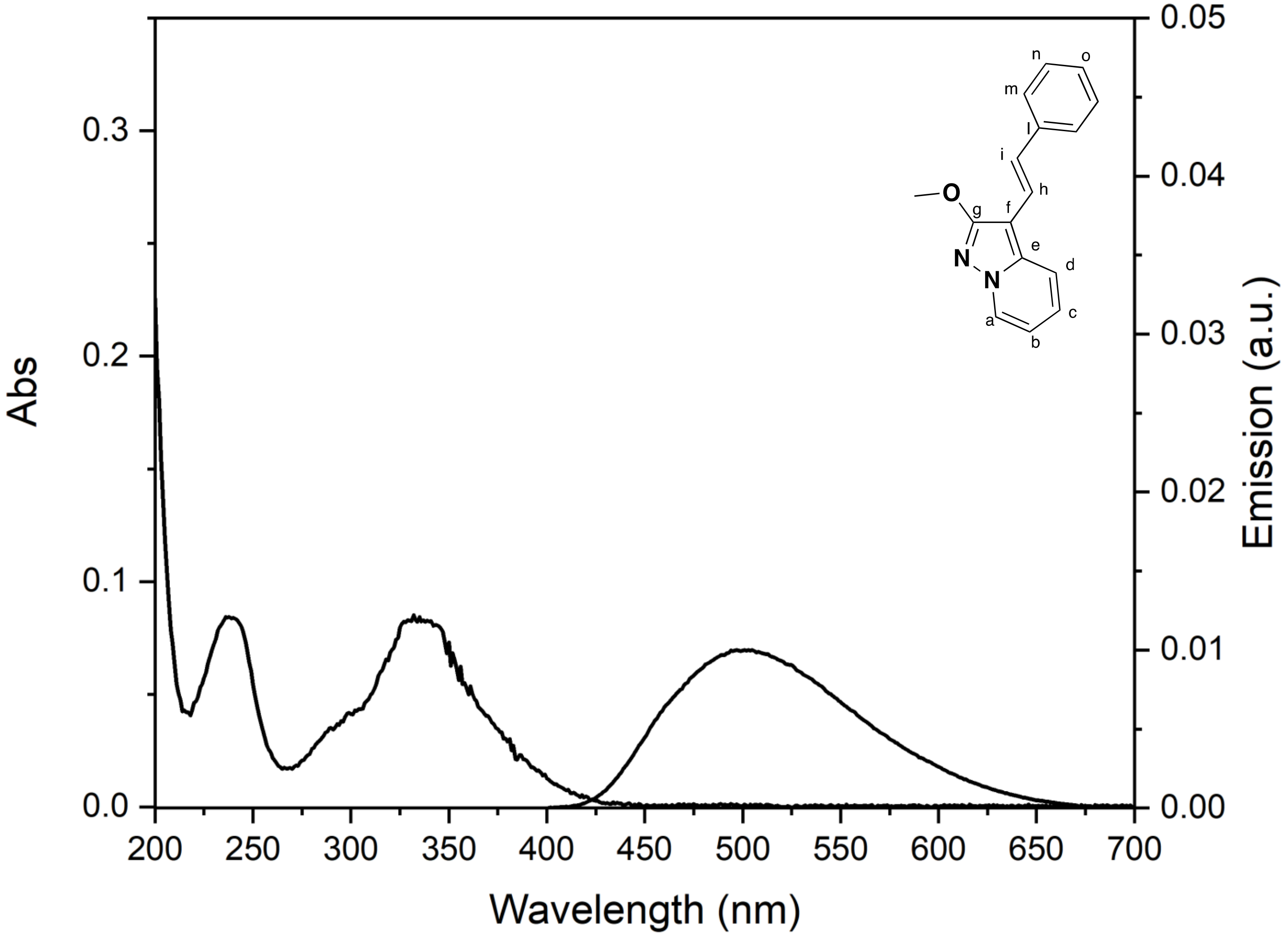

(E)-2-methoxy-3-(4-(methylthio)styryl)pyrazolo[1,5-a]pyridine (**5b**)

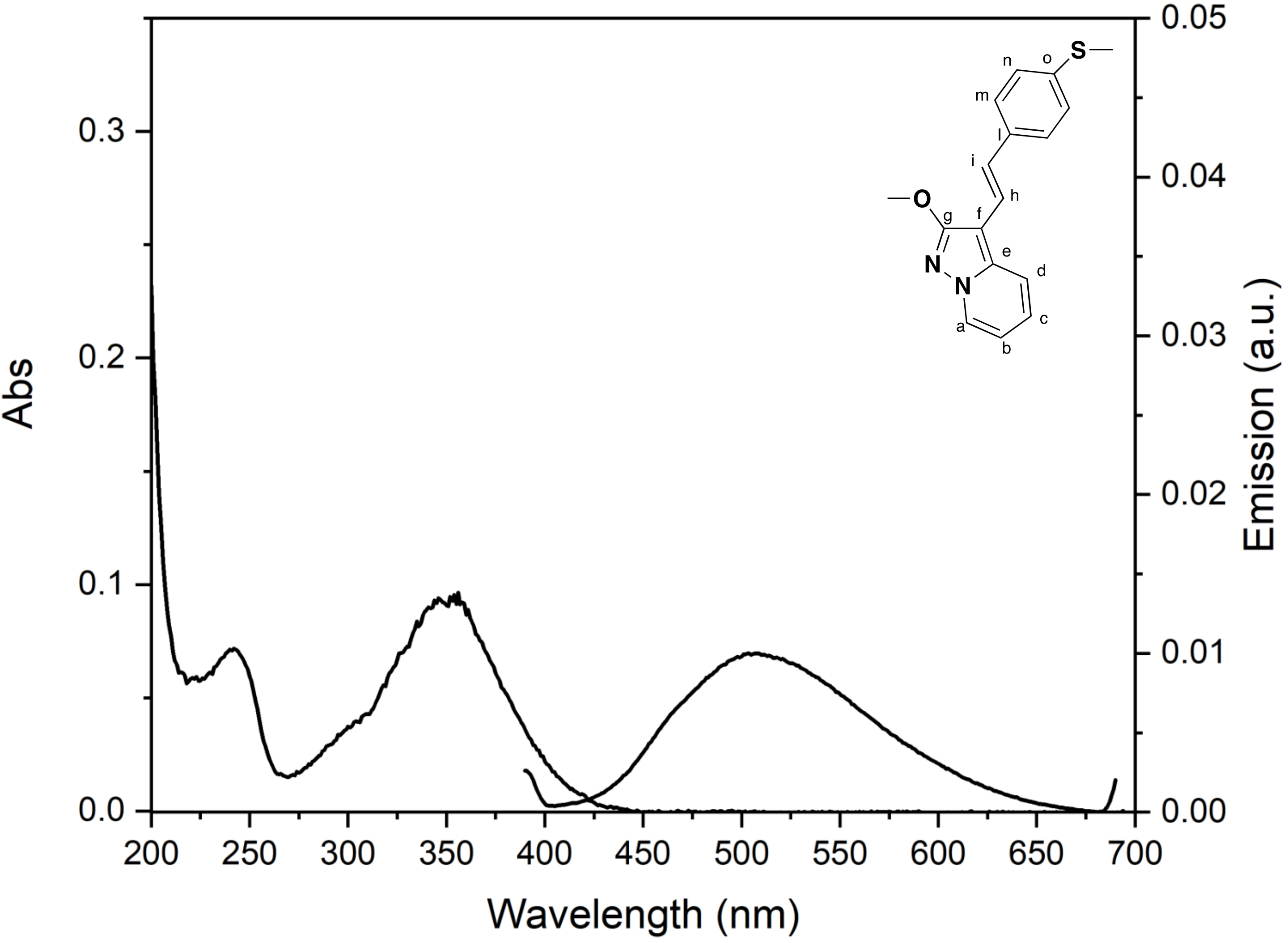

(E)-2-methoxy-3-(4-(methylsulfonyl)styryl)pyrazolo[1,5-a]pyridine (**5c**)

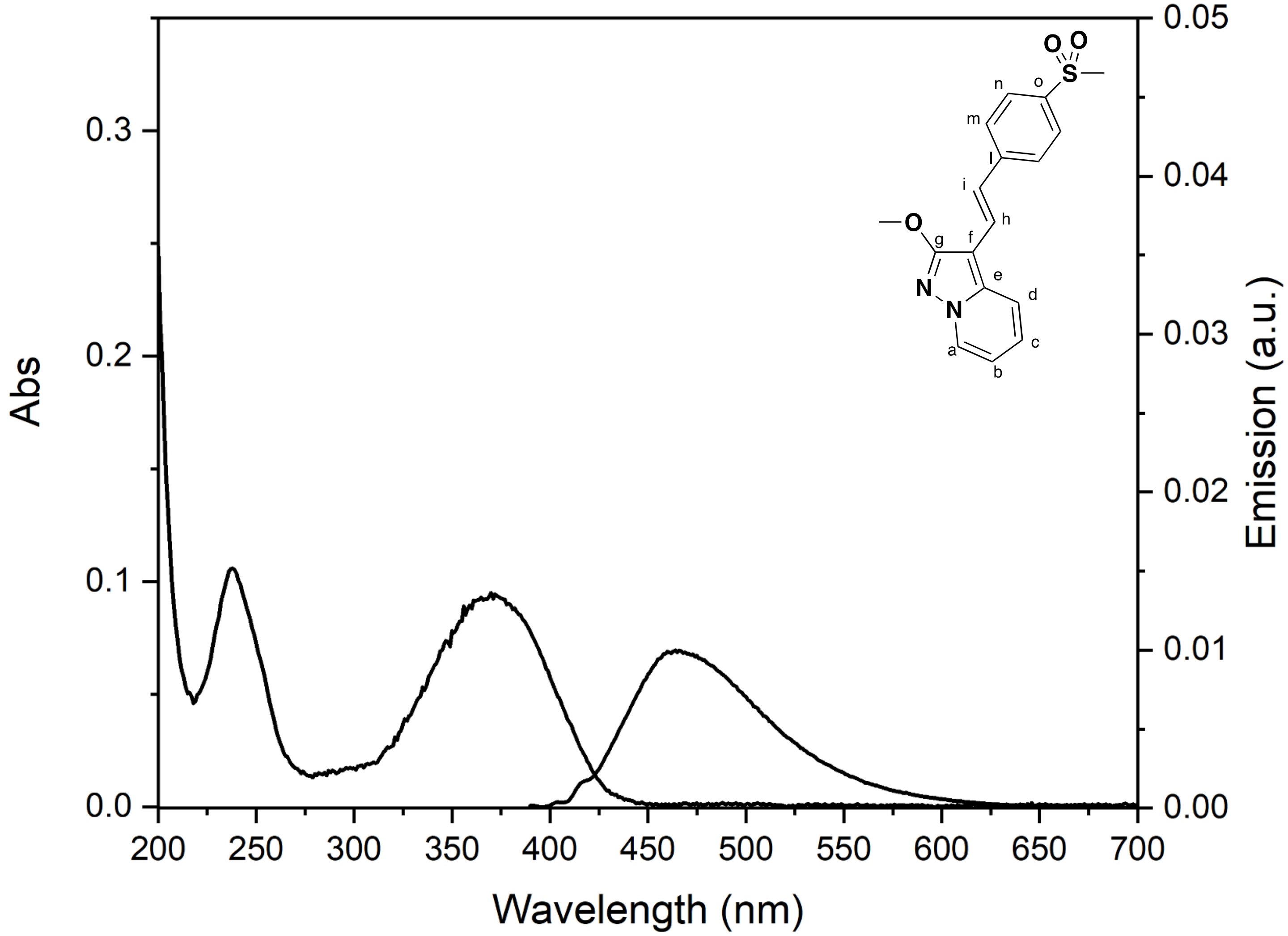

(E)-4-(2-(2-methoxypyrazolo[1,5-a]pyridin-3-yl)vinyl)benzonitrile (**5d**)

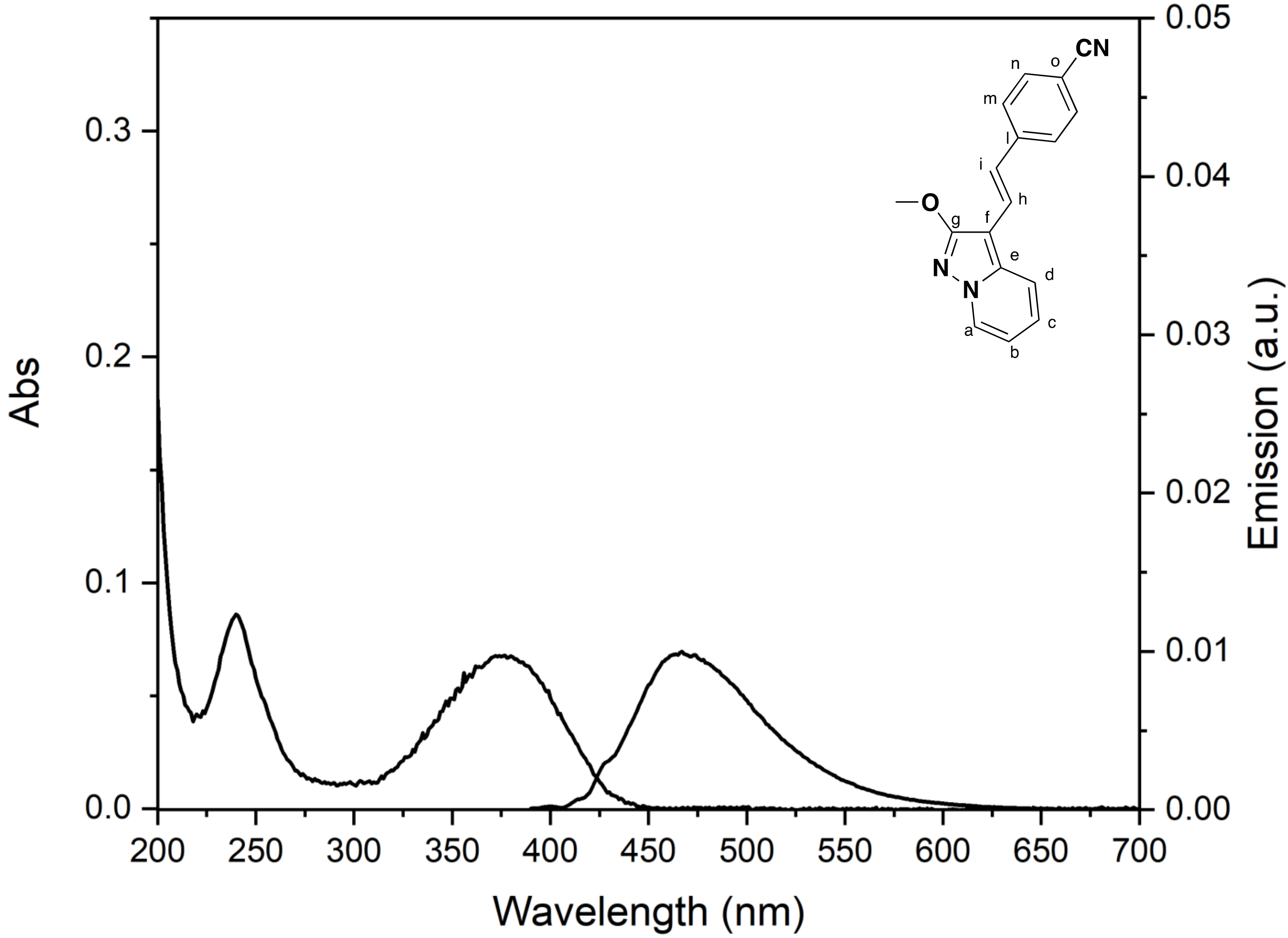

(E)-2-methoxy-3-(4-nitrostyryl)pyrazolo[1,5-a]pyridine (**5e**)

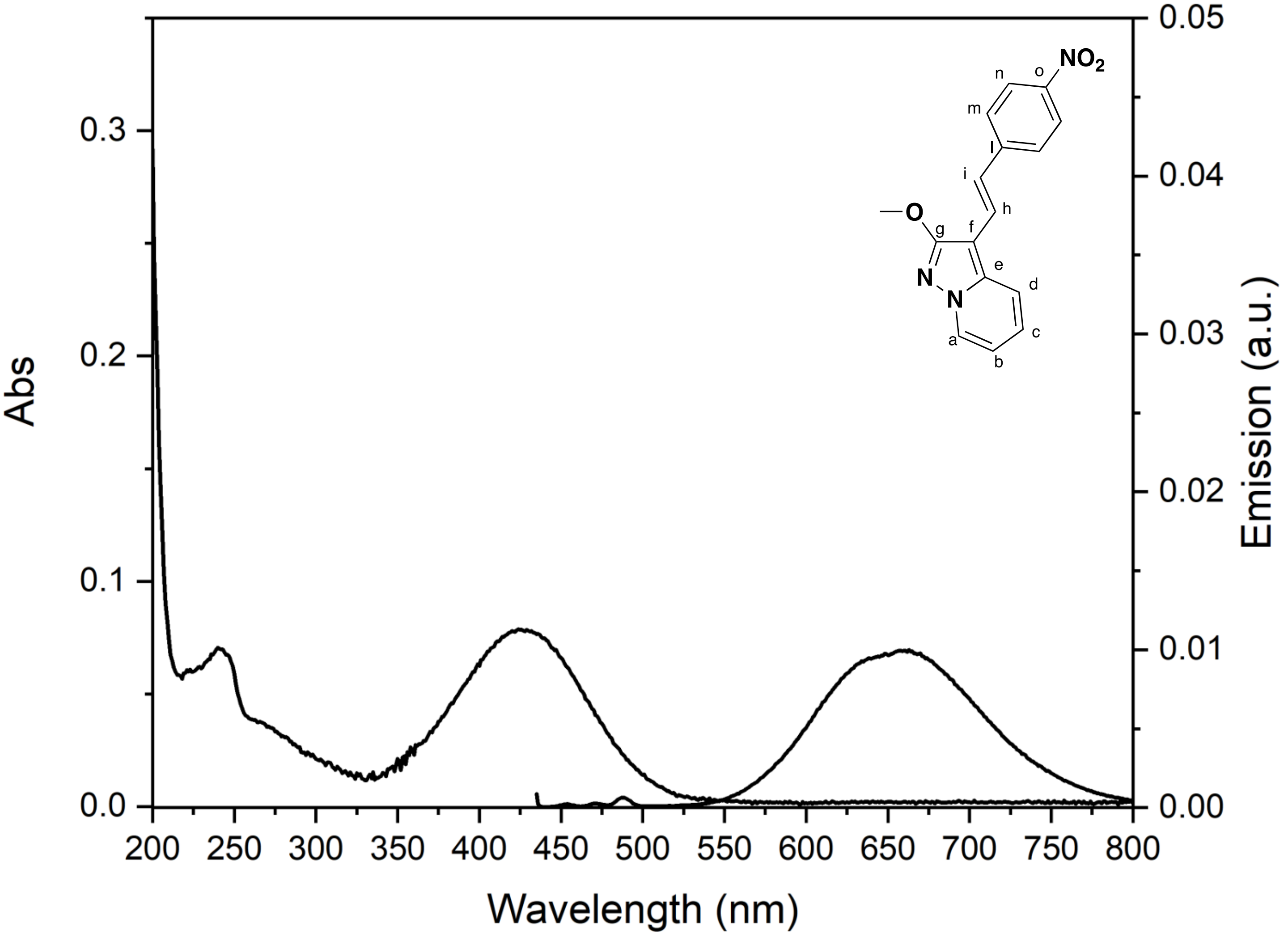

2-methoxy-3-(naphthalen-2-yl)pyrazolo[1,5-a]pyridine (**6**)

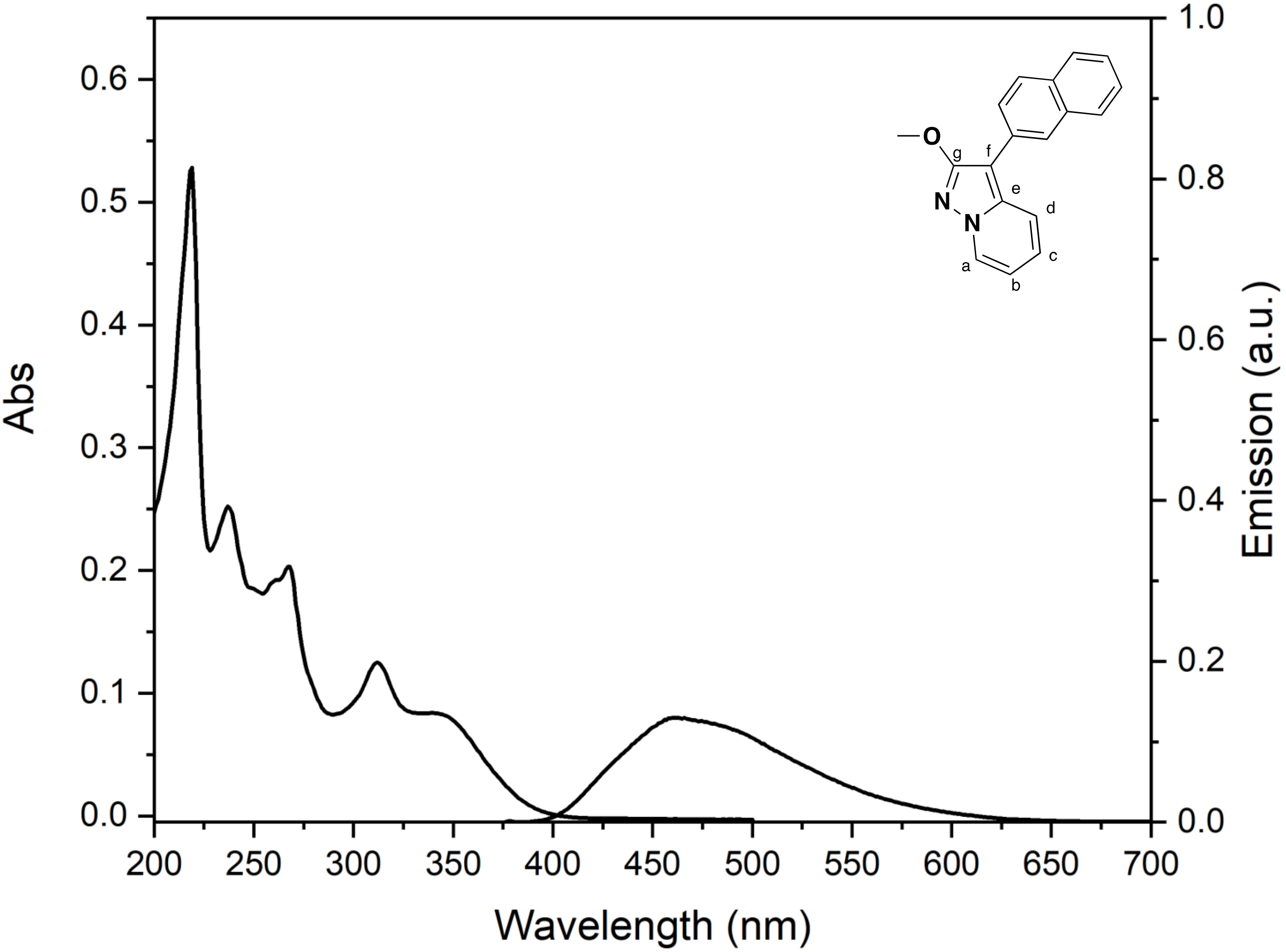

2-(2-methoxypyrazolo[1,5-a]pyridin-3-yl)quinoline (**7a**)

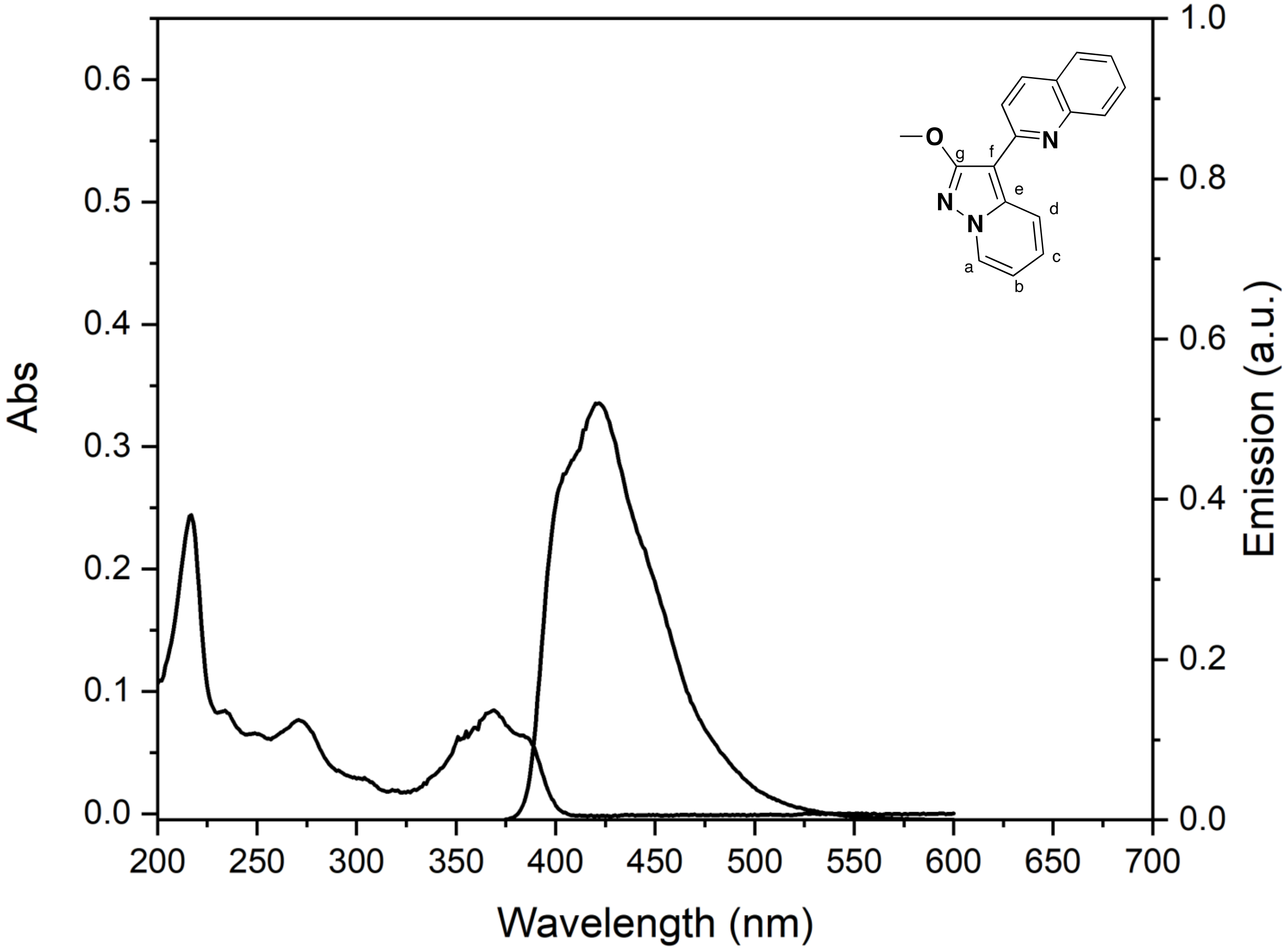

7-(2-methoxypyrazolo[1,5-a]pyridin-3-yl)quinoline (**7b**)

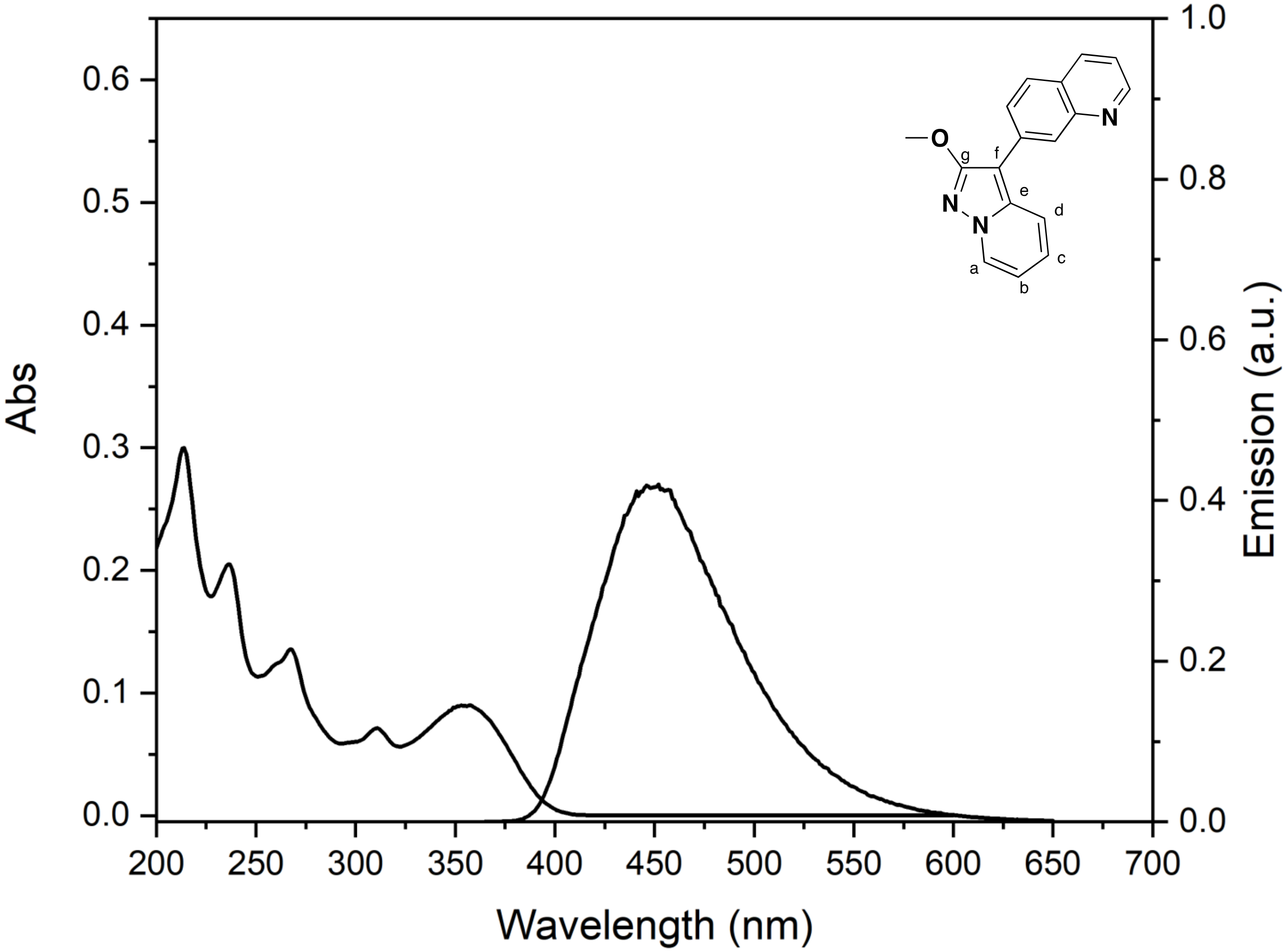

6-(2-methoxypyrazolo[1,5-a]pyridin-3-yl)quinoline (**7c**)

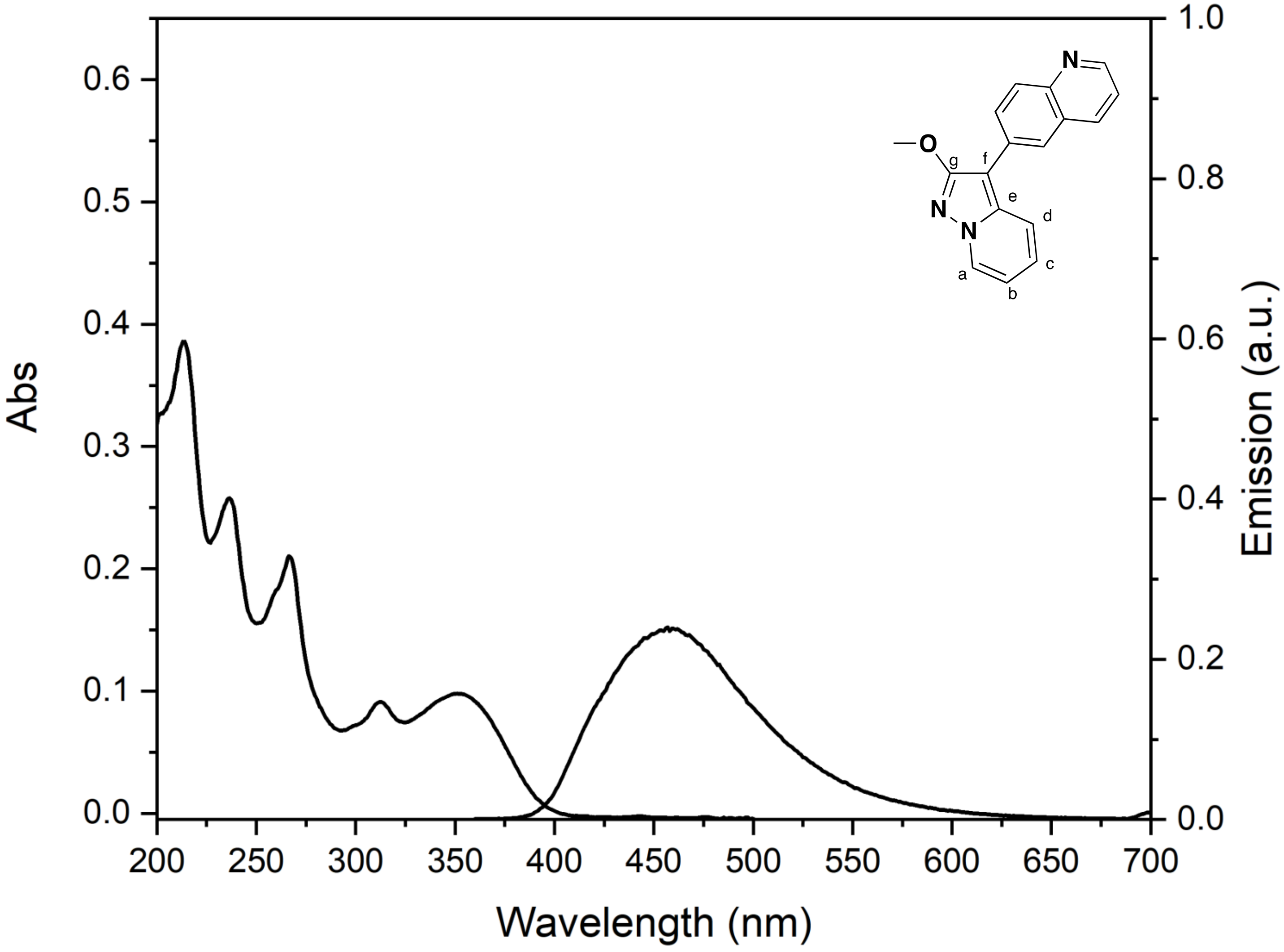

Ethyl 7-(dimethylamino)-2-hydroxy-6-nitropyrazolo[1,5-a]pyridine-3-carboxylate (**8b**)

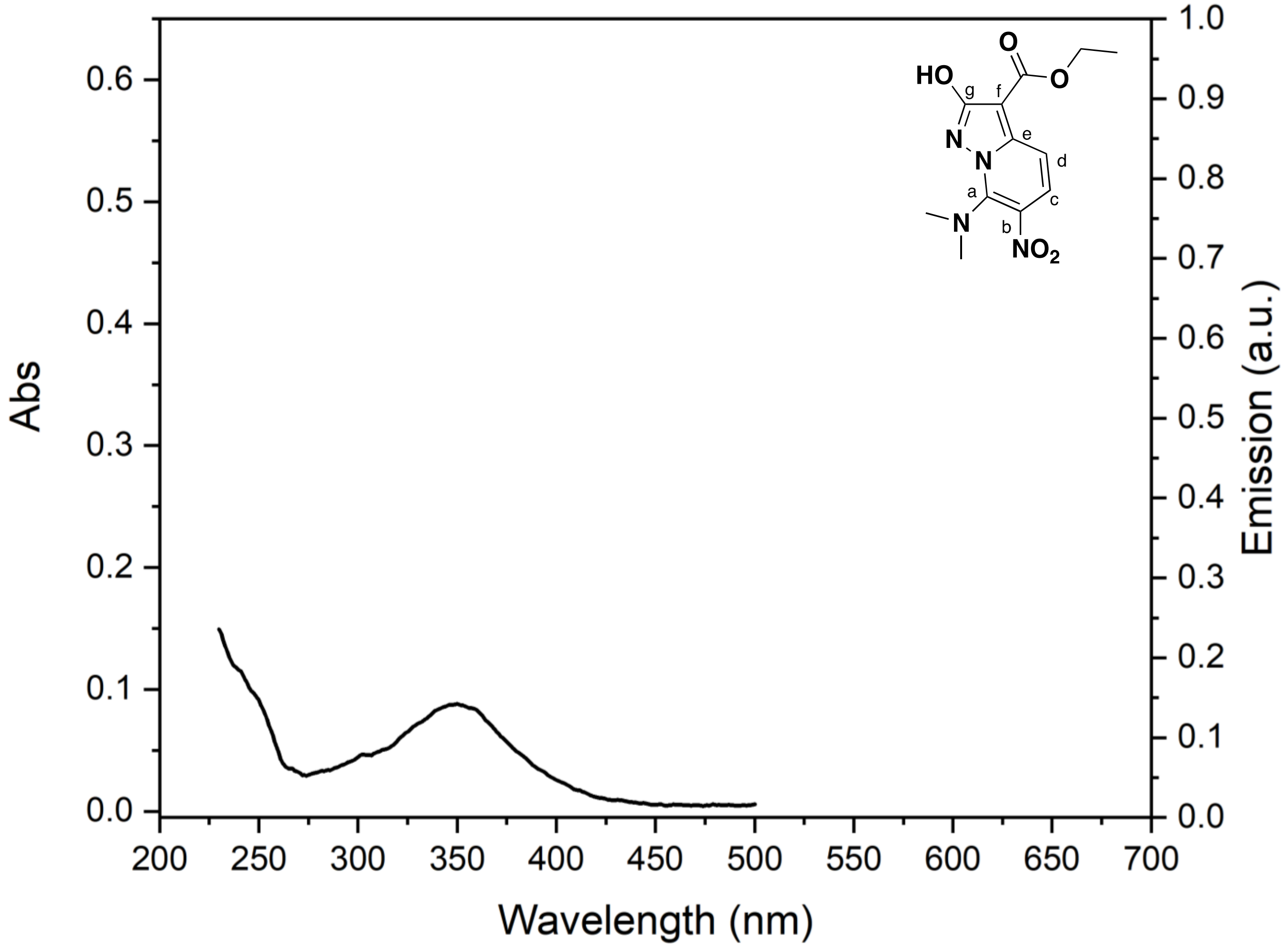

Ethyl 7-(dimethylamino)-2-hydroxy-4-nitropyrazolo[1,5-a]pyridine-3-carboxylate (**8c**)

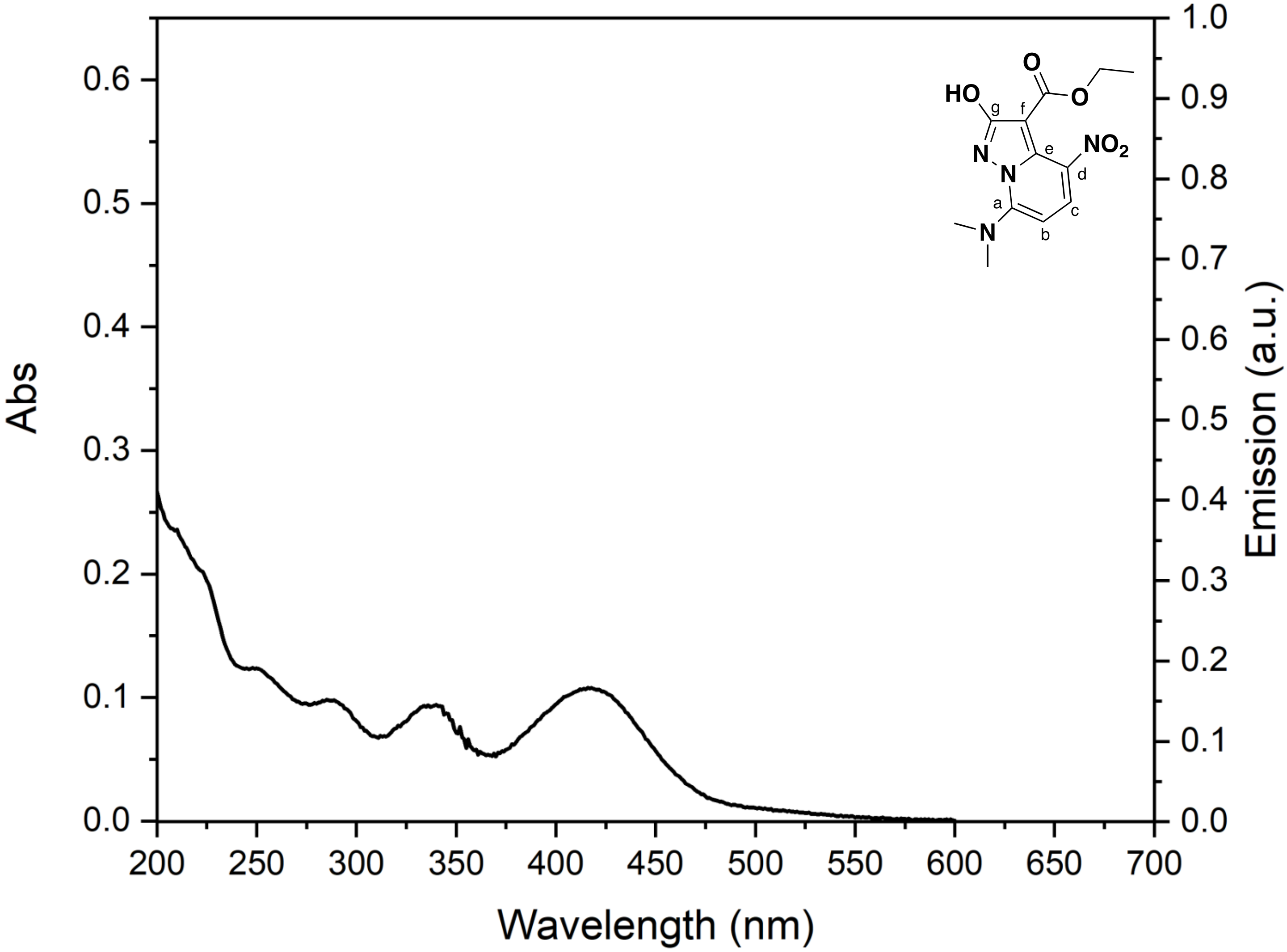

2-Methoxy-N,N-dimethyl-3-(quinolin-6-yl)pyrazolo[1,5-a]pyridin-7-amine (**9b**)

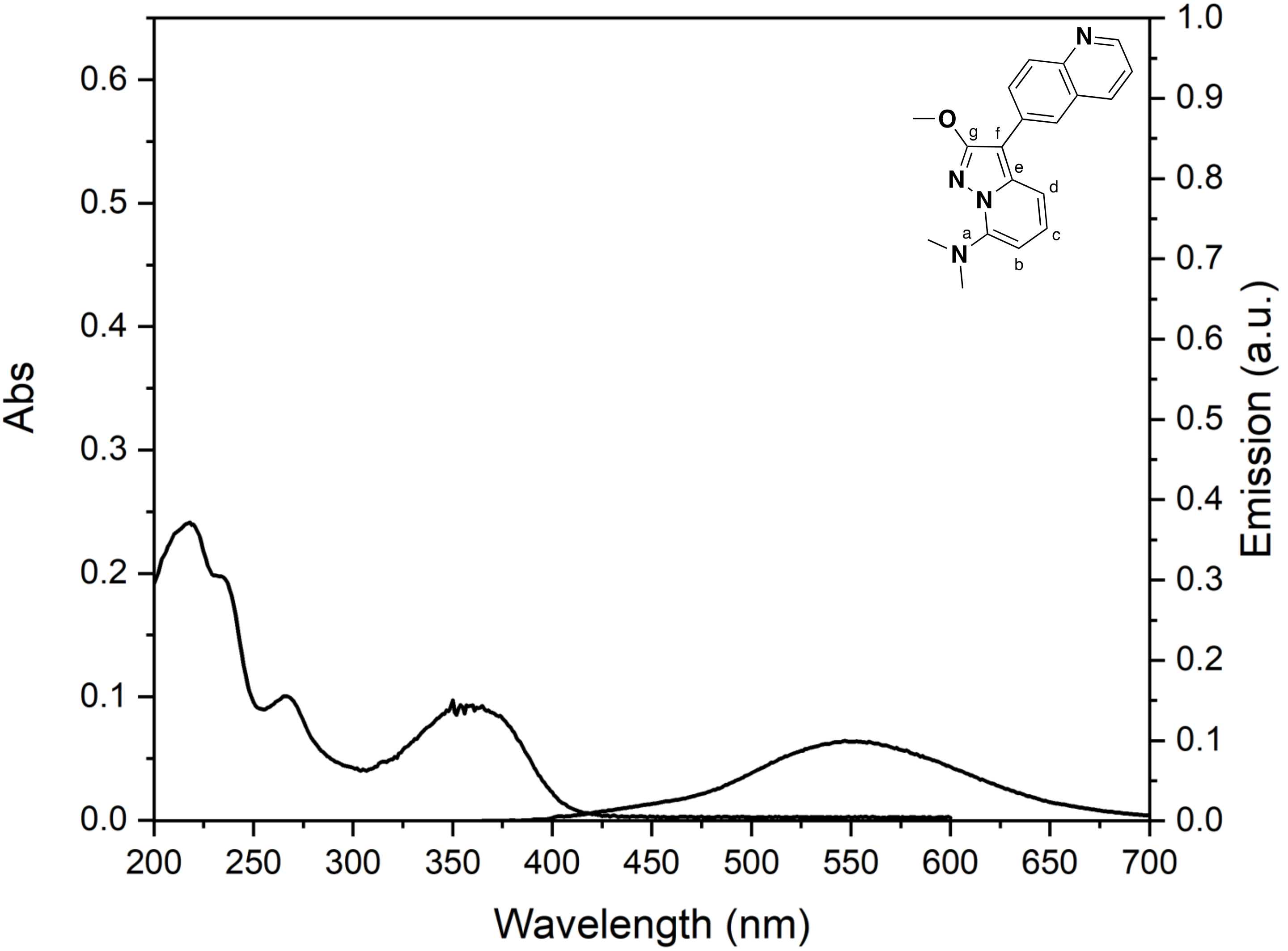

6-(7-chloro-2-methoxypyrazolo[1,5-a]pyridin-3-yl)quinoline (**9a**)

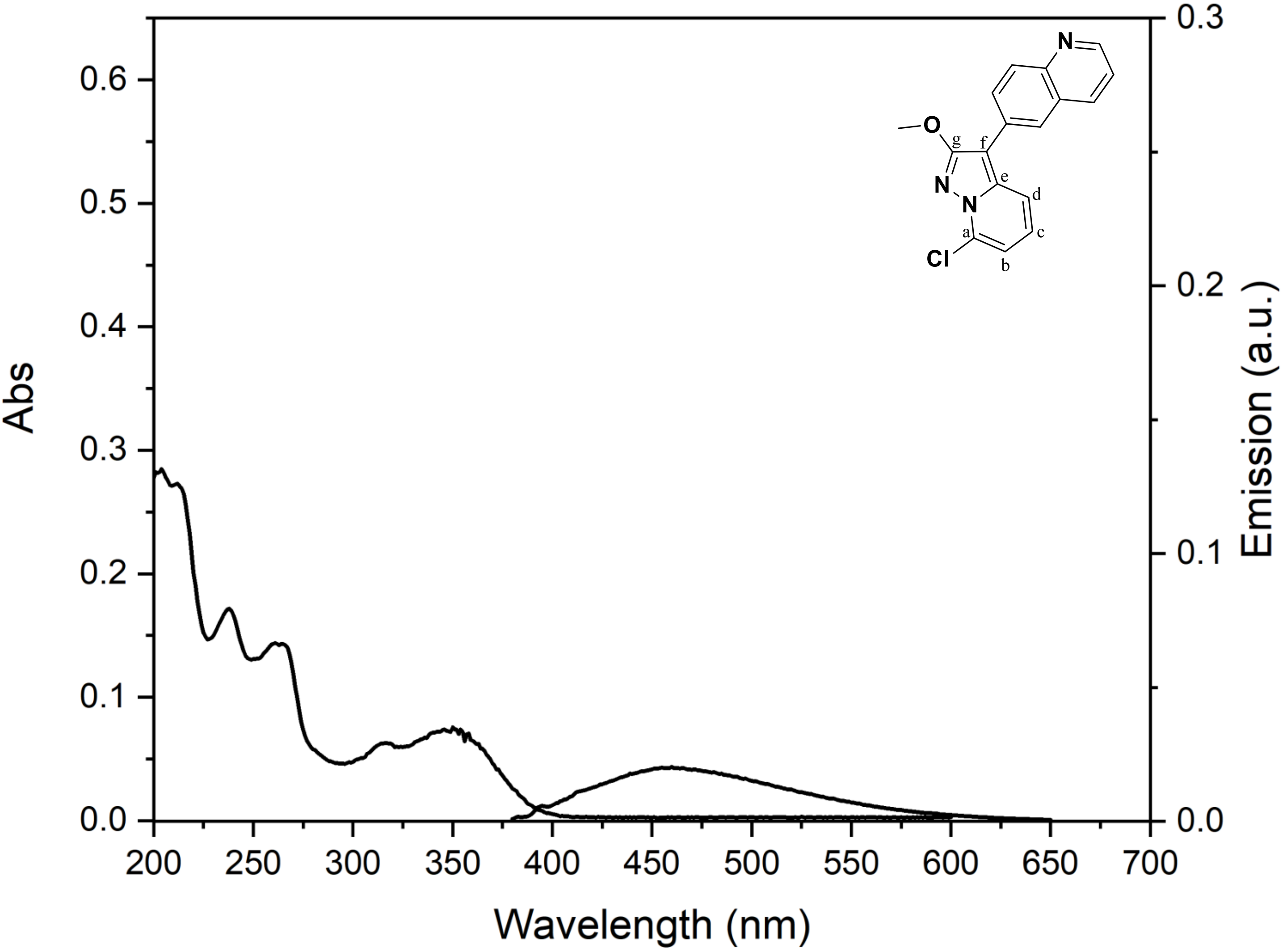

((E)-3-(2-(2,3,6-trifluoro-[1,1'-biphenyl]-4-yl)vinyl)pyrazolo[1,5-a]pyridin-2-ol (**11b**)

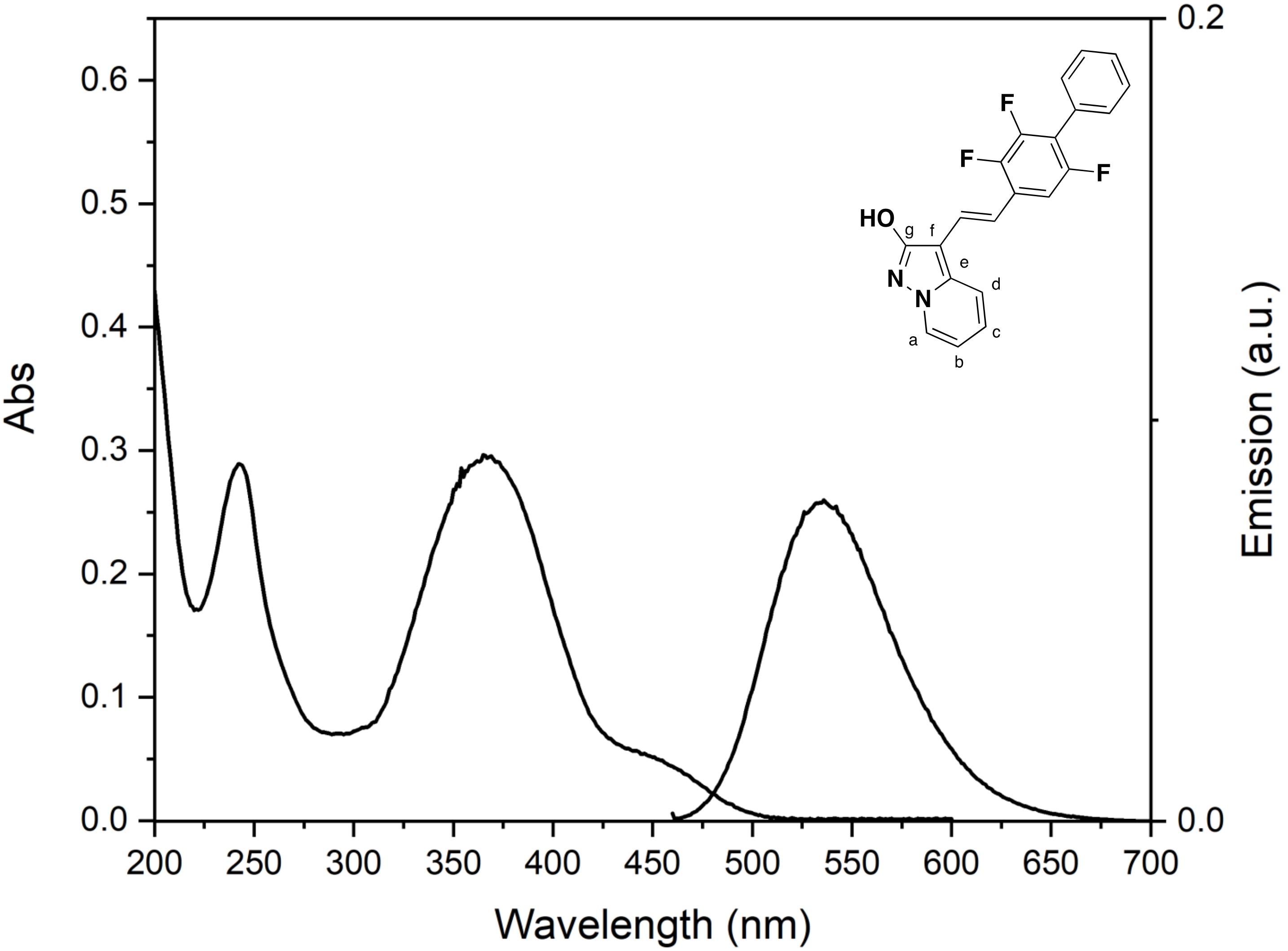

(E)-3-(2-(2,3,5,6-Tetrafluoro-[1,1'-biphenyl]-4-yl)vinyl)pyrazolo[1,5-a]pyridin-2-ol (**11a**)

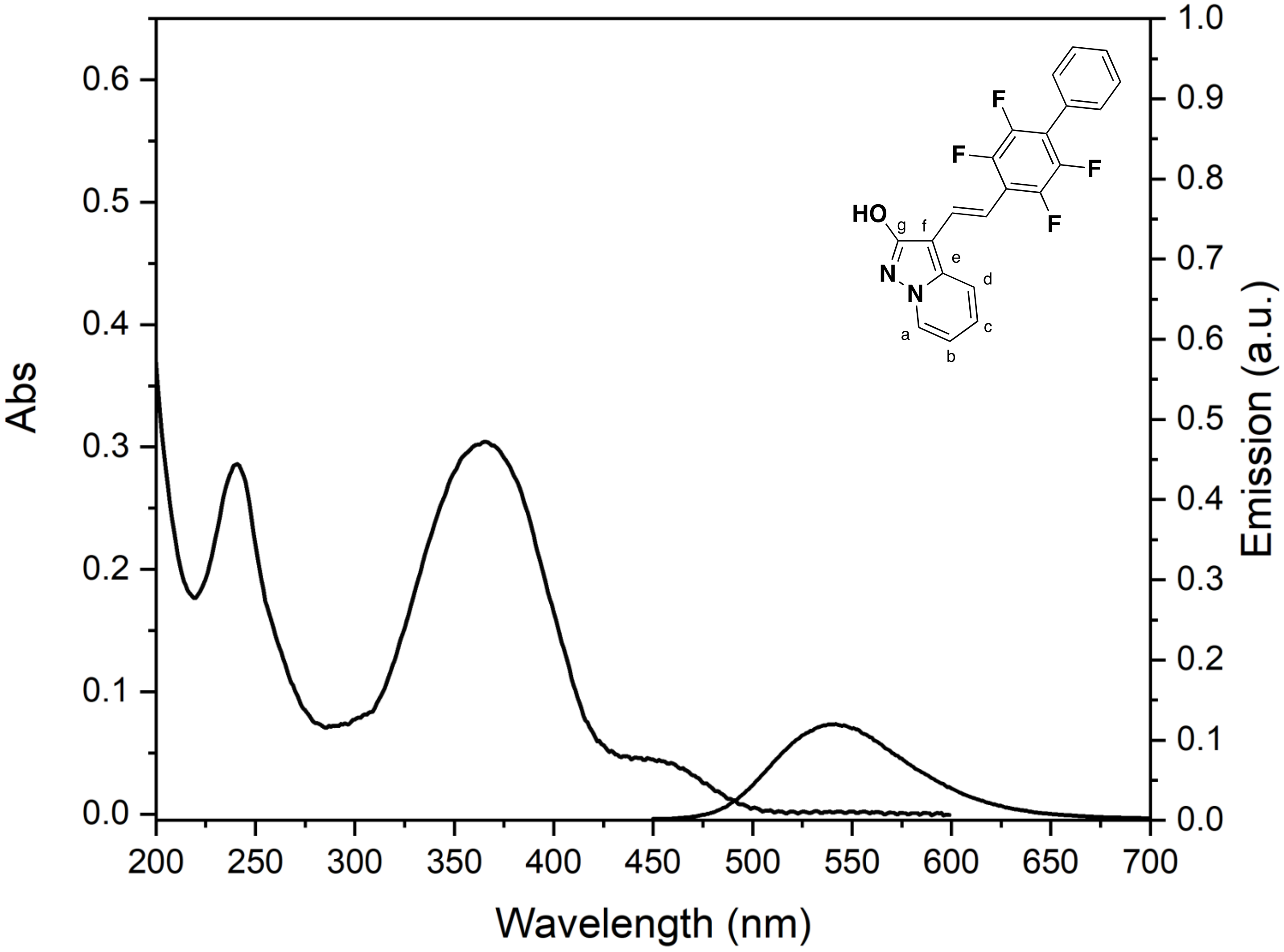

3-(6-phenylquinolin-2-yl)pyrazolo[1,5-a]pyridin-2-ol (**12**)

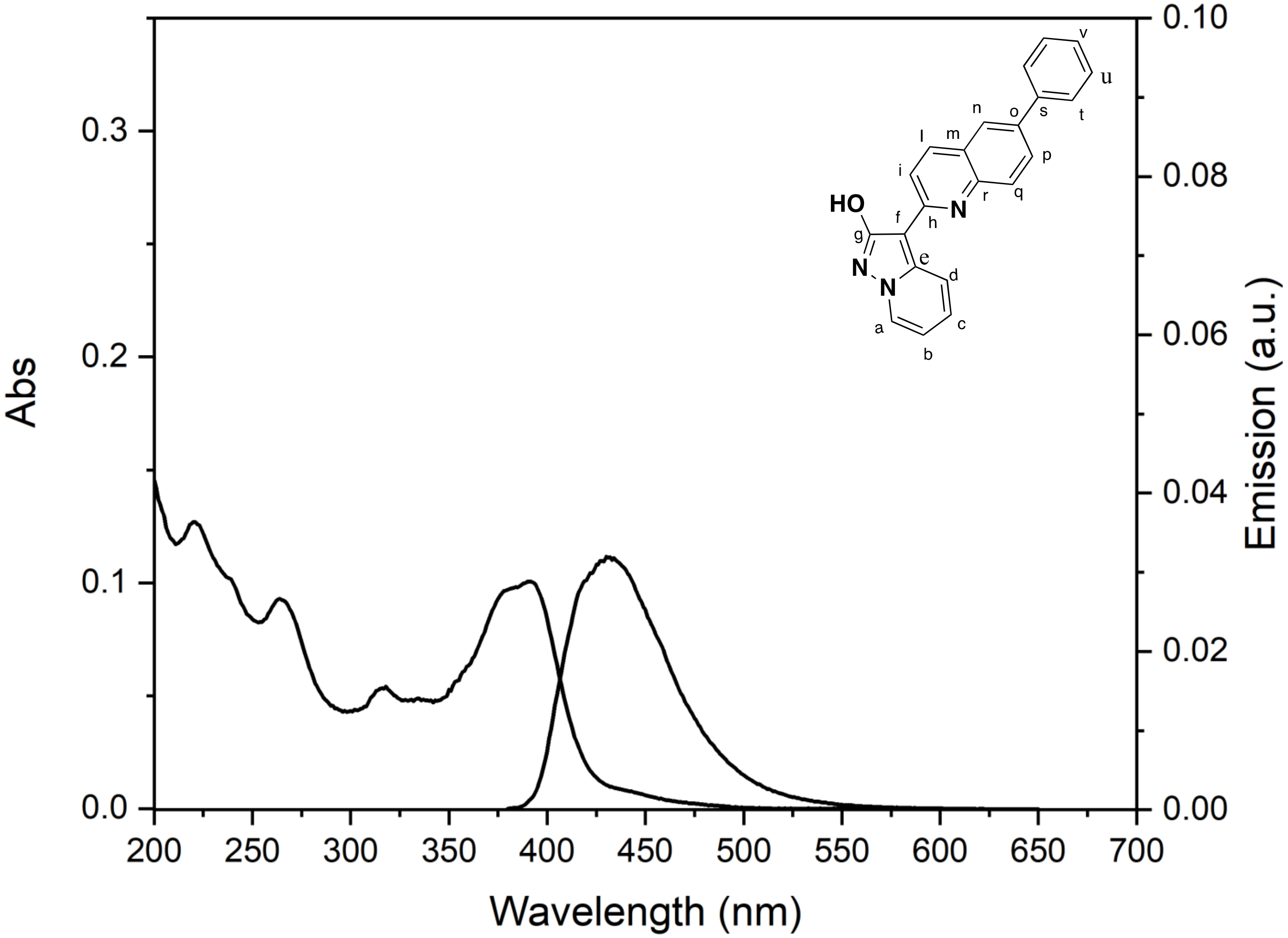

3-(3-phenylquinolin-7-yl)pyrazolo[1,5-a]pyridin-2-ol (**13**)

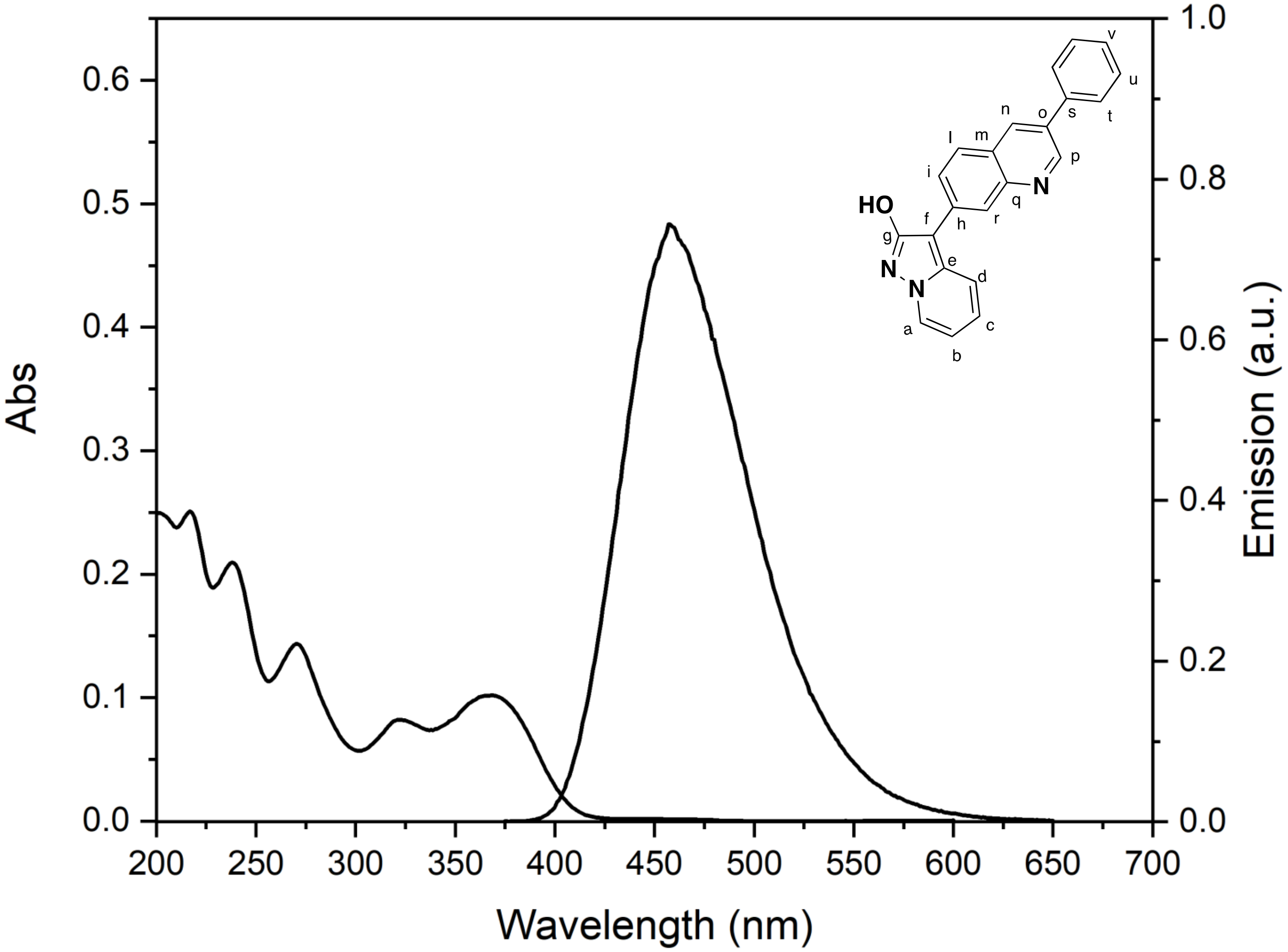

3-(3-(2,6-difluorophenyl)quinolin-7-yl)pyrazolo[1,5-a]pyridin-2-ol (**14**)

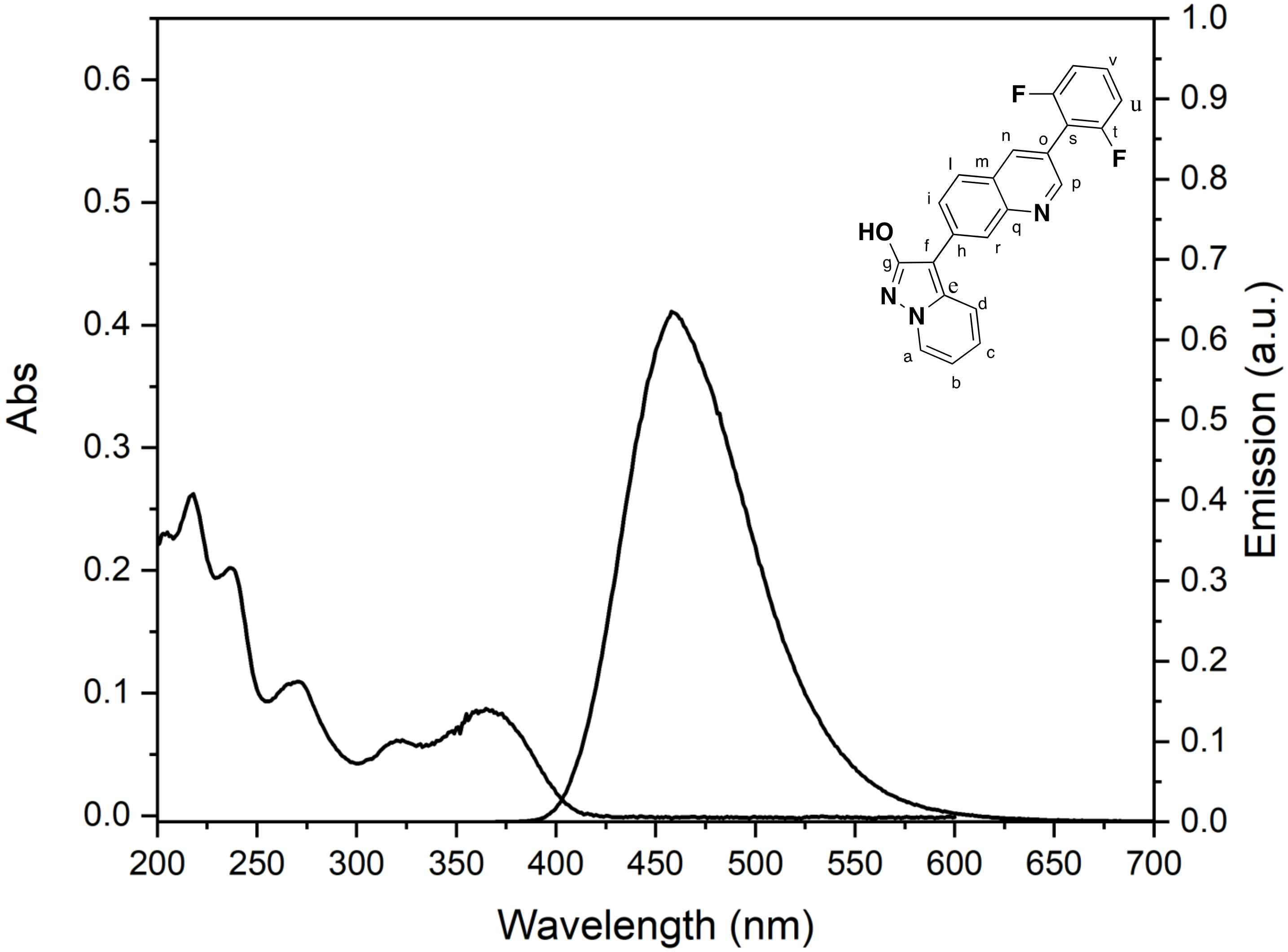

Bibliography

(1) Foschi, F.; Tagliabue, A.; Mihali, V.; Pilati, T.; Pecnikaj, I.; Penso, M. Memory of Chirality Approach to the Enantiodivergent Synthesis of Chiral Benzo[D]Sultams. *Org Lett* **2013**, *15* (14), 3686-3689.
(2) Li, D. K.; Cai, Q.; Zhou, R. R.; Wu, Y. D.; Wu, A. X. The Synthesis of 3-Arylquinolines from -Nitrobenzaldehydes and B-Nitrostyrenes Via an Iron-Promoted Reductive Cyclization. *ChemistrySelect* **2017**, *2* (3), 1048-1051.
